# Unveiling the Antimicrobial, Anti-Biofilm, and Anti-Quorum-Sensing Potential of *Paederia foetida* Linn. Leaf Extract against *Staphylococcus aureus*: An Integrated In Vitro–In Silico Investigation

**DOI:** 10.3390/antibiotics13070613

**Published:** 2024-07-01

**Authors:** Sirijan Santajit, Witawat Tunyong, Dararat Horpet, Asma Binmut, Thida Kong-Ngoen, Churaibhon Wisessaowapak, Techit Thavorasak, Pornpan Pumirat, Nitaya Indrawattana

**Affiliations:** 1 Department of Medical Technology, School of Allied Health Sciences, Walailak University, Tha Sala 80160, Thailand; sirijan.sa@wu.ac.th (S.S.); pdararat@wu.ac.th (D.H.); abilmud@gmail.com (A.B.); 2 Research Center in Tropical Pathobiology, Walailak University, Tha Sala 80160, Thailand; 3 Department of Microbiology and Immunology, Faculty of Tropical Medicine, Mahidol University, Bangkok 10400, Thailand; witawat.tun@mahidol.ac.th (W.T.); thida.kon@mahidol.ac.th (T.K.-N.); pornpan.pum@mahidol.ac.th (P.P.); 4 Department of Medicine and Pharmacology, University of California, San Diego, CA 92093, USA; chwisessaowapak@health.ucsd.edu; 5 Center of Research Excellence in Therapeutic Proteins and Antibody Engineering, Department of Parasitology, Faculty of Medicine Siriraj Hospital, Mahidol University, Bangkok 10700, Thailand; techit.tha@mahidol.edu; 6 Siriraj Center of Research Excellence in Allergy and Immunology, Department of Research, Faculty of Medicine Siriraj Hospital, Mahidol University, Bangkok 10700, Thailand

**Keywords:** antimicrobials, anti-biofilms, bioactive compounds, *Paederia foetida* Linn., molecular docking, quorum sensing

## Abstract

Antimicrobial resistance poses a global health threat, with *Staphylococcus aureus* emerging as a notorious pathogen capable of forming stubborn biofilms and regulating virulence through quorum sensing (QS). In the quest for novel therapeutic strategies, this groundbreaking study unveils the therapeutic potential of *Paederia foetida* Linn., an Asian medicinal plant containing various bioactive compounds, contributing to its antimicrobial activities, in the battle against *S. aureus*. Through a comprehensive approach, we investigated the effect of ethanolic *P. foetida* leaf extract on *S. aureus* biofilms, QS, and antimicrobial activity. The extract exhibited promising inhibitory effects against *S. aureus* including the biofilm-forming strain and MRSA. Real-time PCR analysis revealed significant downregulation of key virulence and biofilm genes, suggesting interference with QS. Biofilm assays quantified the extract’s ability to disrupt and prevent biofilm formation. LC-MS/MS analysis identified quercetin and kaempferol glycosides as potential bioactive constituents, while molecular docking studies explored their binding to the QS transcriptional regulator SarA. Computational ADMET predictions highlighted favorable intestinal absorption but potential P-glycoprotein interactions limiting oral bioavailability. While promising anti-virulence effects were demonstrated, the high molecular weights and excessive hydrogen bond donors/acceptors of the flavonoid glycosides raise concerns regarding drug-likeness and permeability. This integrated study offers valuable insights for developing novel anti-virulence strategies to combat antimicrobial resistance.

## 1. Introduction

*Staphylococcus aureus*, a member of the ESKAPE (*Enterococcus faecium*, *S. aureus*, *Klebsiella pneumoniae*, *Acinetobacter baumannii*, *Pseudomonas aeruginosa*, and *Enterobacter species*) group of pathogens, is a leading culprit of nosocomial infections [1]. The emergence of biofilm-forming, multidrug-resistant *S. aureus* strains, particularly methicillin-resistant *S. aureus* (MRSA), poses a formidable challenge in healthcare and agricultural settings [2]. *S. aureus* can cause a wide range of infections and exhibit a remarkable ability to develop resistance against conventional antibiotics [3,4]. Its pathogenicity is largely attributed to its ability to form robust biofilms, which are microbial communities encased in an extracellular matrix that enables evasion of host immune responses, antimicrobial resistance, and tenacious surface adhesion [5,6]. Additionally, the multilayered biofilms are facilitated by the *icaADBC* operon, which facilitates the synthesis of an extracellular matrix [7]. *S. aureus* employs quorum-sensing (QS) mechanisms to coordinate the expression of virulence factors, exacerbating its pathogenicity. Combating biofilm formation and disrupting QS signaling have emerged as critical objectives in mitigating the impact of *S. aureus* infections, underscoring the urgent need for novel therapeutic approaches tailored for healthcare and veterinary applications [8]. The QS mechanism in *S. aureus* is governed by two main cascades: the staphylococcal accessory regulator (Sar) and the accessory global regulator (Agr). Within the Agr QS system, oligopeptides serve as signaling molecules to modulate the expression of various virulence factors. SarA (Staphylococcal accessory regulator A) is a pivotal regulatory protein in the *S. aureus* QS cascade, significantly impacting bacterial pathogenicity and resistance mechanisms [9,10]. It controls the expression of numerous virulence factors, influencing the transcription of gene-encoding toxins, enzymes, and surface proteins, thus playing a crucial role in the bacteria’s ability to cause infections ranging from minor skin conditions to severe diseases such as pneumonia and sepsis. SarA promotes biofilm formation by upregulating genes responsible for extracellular polysaccharide production and repressing proteases that could degrade the biofilm matrix, thereby enhancing bacterial resistance to antibiotics and host immune responses. Furthermore, SarA contributes to antibiotic resistance by regulating stress response and cell wall synthesis genes, including the methicillin resistance gene *mecA*. Given its central role in virulence and biofilm formation, SarA is a promising target for new therapeutic strategies aimed at attenuating *S. aureus* pathogenicity and enhancing susceptibility to antibiotics [8,11].

Bioactive phytoconstituents have emerged as promising agents for combating infectious diseases caused by bacteria that form biofilms. These compounds, derived from plants, exert their therapeutic effects by targeting genes responsible for disease progression, particularly by disrupting virulence factors and inhibiting biofilm formation associated with quorum sensing (QS). Traditional medicine has a rich history of utilizing plants to combat microbial infections [12,13]. Among these botanical remedies, *Paederia foetida* (known as Sembukan in Indonesian and King’s Tonic in English), a climbing herb belonging to the *Rubiaceae* family, stands out as one of Asia’s indigenous medicinal plants, thriving in both temperate and tropical regions. Despite its bitter taste and unpleasant odor, the leaves of this plant are traditionally employed to address a range of ailments, including diarrhea, rheumatism, inflammation, piles, dysentery, and stomachache [14,15,16]. This traditional wisdom serves as a valuable resource for uncovering potential drugs. Notably, compounds such as quercetin, catechin, rosmarinic acid, limonoid, ichangin, apigenin, kaempferol, and naringenin have demonstrated efficacy against biofilm-associated infections [17]. Consequently, there is a pressing need to identify potent QS inhibitors (QSIs), preferably sourced from natural reservoirs. The secondary metabolites found in plants have shown promise in effectively treating various infections [18].

Exploring anti-quorum-sensing and anti-biofilm agents from natural sources, such as plant extracts and biologically synthesized agents, offers promising avenues for controlling *S. aureus* infections [19,20]. Molecular docking simulations predict binding modes and affinities between bioactive compounds and key proteins in bacterial QS pathways, guiding the design of novel, potent QS inhibitors [21,22]. This interdisciplinary approach aligns with the One Health initiative, recognizing the interconnectedness of animals, humans, and the environment [23,24]. The present study investigated the antimicrobial, anti-QS, and anti-biofilm formation abilities of *P. foetida* Linn. leaf extract against *S. aureus*, focusing on managing infections in healthcare and veterinary settings while embracing the One Health approach to combat this global threat.

## 2. Results

### 2.1. Qualitative Profiling and Identification of Bioactive Anti-Quorum-Sensing Compounds from the Ethanolic Crude Extract by LC-MS/MS

Bioactive anti-QS compounds against *S. aureus* present in the ethanolic crude extract of *P. foetida* Linn. were tentatively identified by LC-MS/MS analysis. Metabolites were putatively characterized based on accurate mass measurements of the protonated pseudomolecular [M + H]^+^ ions and their fragmentation patterns, compared to the literature data. Supplementary Data S2 revealed the metabolomic profile of the ethanolic crude extract of *P. foetida* Linn. Table 1 lists the retention times (RT), and PubChem database details of the MS/MS fragment ions detected of flavonoid glycosides, which show a promising antibacterial activity against *S. aureus*. Several quercetin glycosides were identified, including Isoquercetin, Quercetin 3-galactoside, Quercetin 3-β-D-glucoside, and Quercetin 3-O-glucoside. Kaempferol glycoside derivatives such as Kaempferol 3-α-D-galactoside and Kaempferol 3-α-D-glucoside were also tentatively characterized among the bioactive anti-QS metabolites present in the crude extract.

### 2.2. Antimicrobial Activity of Paederia Foetida Leaf Crude Extract against S. aureus

The minimal inhibitory concentration (MIC) of the crude extracts was determined against *S. aureus* ATCC 25923 using the broth microdilution method. The crude extract obtained from ethanolic *P. foetida* Linn. leaves exhibited varying minimum inhibitory concentrations (MICs) and minimum bactericidal concentrations (MBCs) against *S. aureus* ATCC 25923 and MRSA. Specifically, the MIC and MBC of the crude extract against *S. aureus* ATCC 25923 were 7.81 μg/mL and 31.25 μg/mL, respectively. For MRSA, the MIC and MBC values were found to be 15.63 μg/mL and 31.25 μg/mL, respectively. The lower MIC value for *S. aureus* ATCC 25923 compared to MRSA indicates that the crude extract is more effective in inhibiting the growth of the non-resistant strain. Additionally, the MIC value of Tetracycline (a positive control) against *S. aureus* ATCC 25923 and MRSA was 0.25 μg/mL and 64 μg/mL, respectively. This comparison shows that while the crude extract is effective, it is not as potent as Tetracycline against the non-resistant strain but is significantly more effective against MRSA compared to Tetracycline.

### 2.3. Impact of Crude Extract on S. aureus Morphology Assessed by SEM

**Figure 1:**
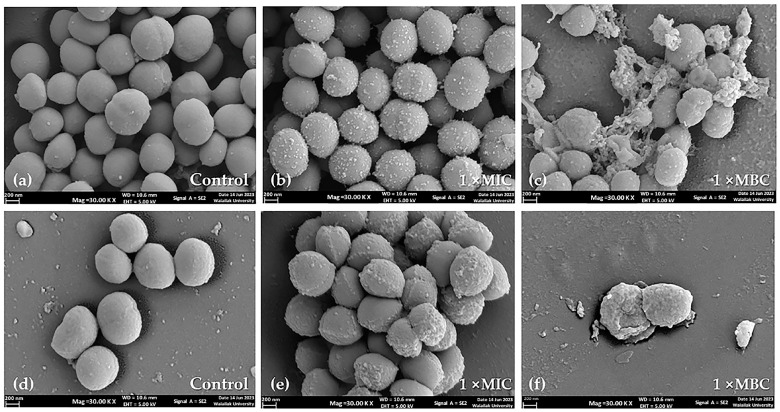
Scanning electron micrographs of *S. aureus* ATCC 25923 and MRSA cell morphology at 24 h treatment (20,000× magnification): (**a**) untreated control cells of *S. aureus* ATCC 25923, (**b**,**c**) *S. aureus* ATCC 25923 after treatment with PF crude extract at 1× MIC and 1× MBC, respectively; (**d**) untreated control of MRSA cells, (**e**,**f**) MRSA after treatment with PF crude extract at 1× MIC and 1× MBC, respectively. Scale bars indicate 200 nm.

SEM images revealed the effects of the *P. foetida* Linn. (PF) crude extract on the morphology of *S. aureus* ATCC 25923 (methicillin-sensitive *S. aureus*, MSSA) and methicillin-resistant *S. aureus* (MRSA) cells in a concentration-dependent manner. The control cells exposed to media containing DMSO (vehicle control) exhibited a slightly smooth, spherical shape, with some undergoing cell division (Figure 1a,d).

At 1× MIC (minimum inhibitory concentration) of the PF crude extract treatment, most cells appeared to have a wrinkled spherical cell surface with increased accumulation of extracellular matrix for both MSSA and MRSA strains (Figure 1b,e). When the concentration was increased to 1× MBC (minimum bactericidal concentration) of the PF crude extract, extensive damage to the cell surface and distortion of morphology were observed. Figure 1c,f show severe cell wall destruction, irregular dimpled surfaces, cell rupture, and leakage of intracellular contents, suggesting cell lysis.

The SEM images demonstrate that the PF crude extract caused concentration-dependent morphological changes and damage to both MSSA and MRSA cells, ultimately leading to cell lysis at higher concentrations.

### 2.4. P. foetida Extract Interfered with S. aureus Biofilm Formation at Sub-Inhibitory Concentrations

**Figure 2:**
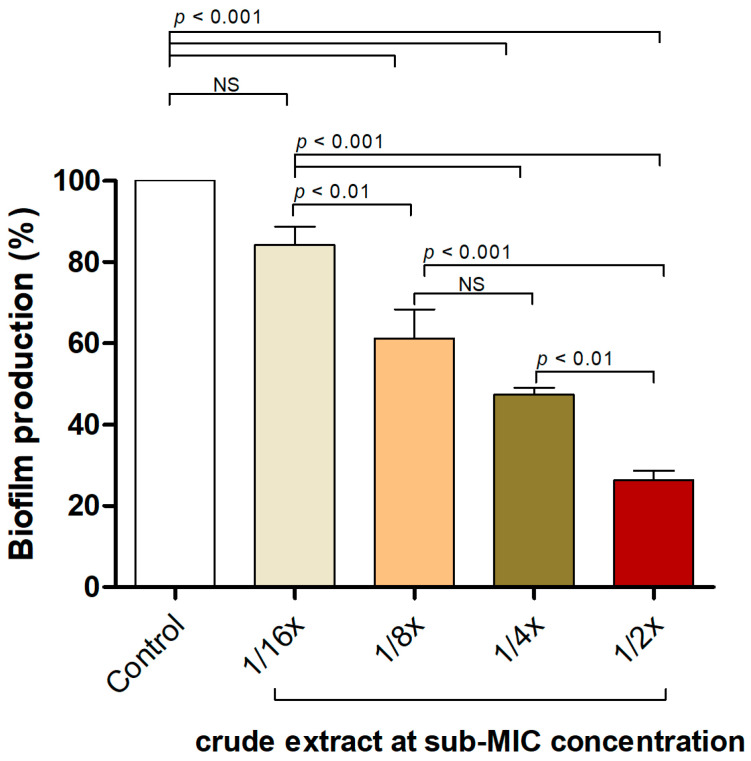
*P. foetida* Linn. extract interfered with biofilm formation in *S. aureus* ATCC 25923 using a static microtiter plate assay at sub-MIC level (1/2× MIC, 1/4× MIC, 1/8× MIC and 1/16× MIC). The data represent an average of triplicate experiments ± SD (n = 3) and with error bars indicating standard deviations. Non-significant results are denoted as ‘NS’.

The effectiveness of the *P. foetida* Linn. extract in inhibiting biofilm formation by the *S. aureus* ATCC 25923 strain was assessed using a static microtiter plate assay with crystal violet staining. Figure 2 illustrates the biofilm production percentages of *S. aureus* when exposed to various sub-minimum inhibitory concentrations (sub-MIC) of the crude extract. The control group (untreated) exhibited approximately 100% biofilm production. Treatment with the crude extract at 1/2×, 1/4× and 1/8× sub-MIC concentrations resulted in significant reductions in biofilm production, with *p*-values of <0.01, <0.001, and <0.01, respectively, indicating statistically significant differences compared to the control. Although the 1/16× sub-MIC concentration also reduced biofilm production, this reduction was not statistically significant (NS) compared to the control.

These findings suggest that the crude extract effectively disrupts and inhibits biofilm formation in *S. aureus*, even at sub-inhibitory concentrations, indicating its potential anti-biofilm and anti-quorum-sensing properties against *S. aureus*.

### 2.5. Interference of P. foetida Linn. in Quorum-Sensing Signaling of S. aurues

In *S. aureus* ATCC 25923, the quorum-sensing (QS) system governs the expression of virulence factors, including antibiotic resistance, bacterial transformation, and biofilm formation. Disrupting QS holds promise in mitigating bacterial pathogenesis. Thus, we investigated whether abundant compounds extracted from *P. foetida* Linn. ethanolic extract could interfere with the *S. aureus* QS system. We focused on evaluating their impact on the expression of *sarA*, *ica*, and *hla* genes, which play crucial roles in QS activation and biofilm initiation, respectively.

**Figure 3:**
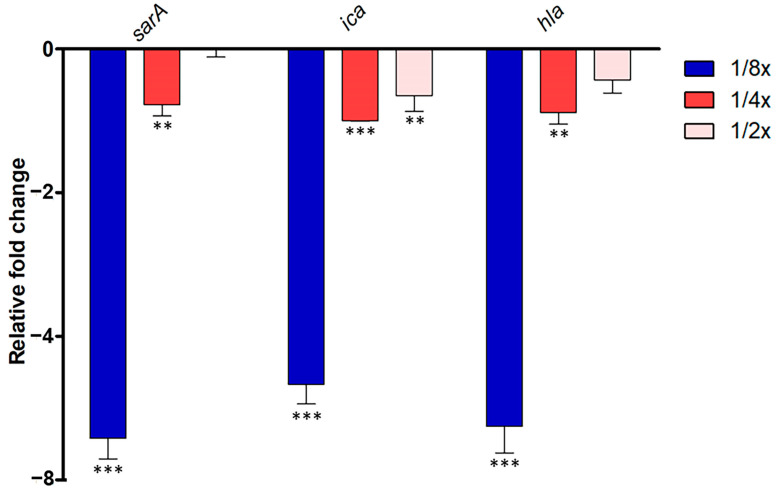
Quantitative Real-Time PCR (qRT-PCR) analysis of QS-related gene expression (sarA, ica, and hla) in *S. aureus* ATCC 25923 treated with different sub-MIC concentrations (1/2× MIC, 1/4× MIC and 1/8× MIC) of *P. foetida* Linn. ethanolic extract. The expression levels were normalized to an internal control gene (16S rRNA). Data are presented as mean ± standard deviation (SD) from three independent experiments. Bar graphs show the average fold change in gene expression compared to the non-treated control (calibrated as 1-fold). Error bars indicate standard deviation (n = 3). Statistical significance was determined by one-way ANOVA followed by Tukey’s post hoc test, with ** indicating *p* < 0.01, *** indicating *p* < 0.001.

The study observed significant downregulation of *sarA* gene expression in the 1/2× MIC condition, with about a 6.4-fold decrease, followed by moderate downregulation in the 1/4× MIC samples (1.7-fold decrease), while 1/8× MIC samples showed slight downregulation or no change. *Ica* gene expression was consistently downregulated across all samples compared to non-treatment, with the strongest downregulation observed in the 1/2× MIC condition (5.7-fold decrease), followed by moderate downregulation in the 1/4× MIC samples (2.0-fold decrease), and slight downregulation in the 1/8× MIC samples (1.6-fold decrease). Similarly, *hla* gene expression showed the strongest downregulation in the 1/2× MIC samples (6.3-fold decrease), moderate downregulation in the 1/4× MIC samples (1.8-fold decrease), and slight downregulation in the 1/8× MIC samples (1.4-fold decrease) (Figure 3).

### 2.6. Molecular Docking of Candidate Anti-QS Ligand with S. aureus QS Transcriptional Regulators and In Silico ADMET Evaluation

**Figure 4:**
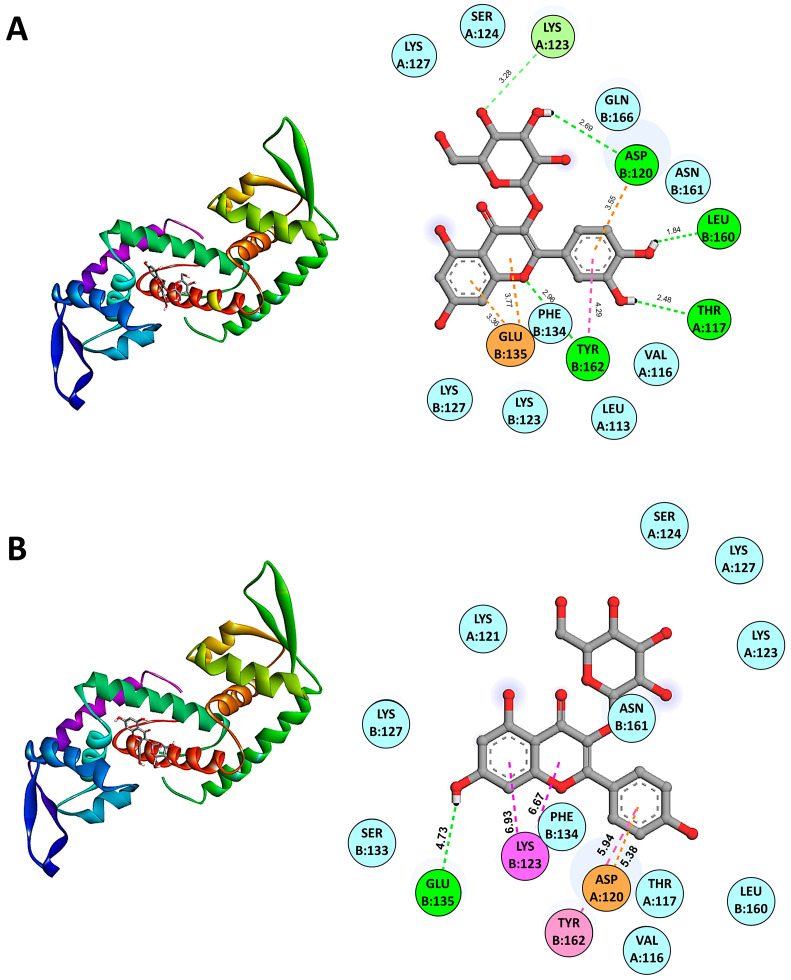
Predicted binding modes from molecular docking of SarA with representative anti-QS ligands including Isoquercetin (**A**) and Kaempferol 3-alpha-D-glucoside (**B**) are shown. The cartoon ribbon model (3D, left side of the panels) depicts the overall structure of SarA. Key interacting amino acids are labeled and highlighted with appropriate interaction colors. Hydrogen bonds and hydrophobic contacts are indicated with dotted lines (2D, right side of the panels). Anti-QS ligands are represented in stick model structures, while key amino acid residues of the protein receptor are displayed as ball models. The SarA–ligand interactive bonds are demonstrated as follows: green for hydrogen bonds, light pink for Pi–Pi stacked interactions, magenta for Pi–cation interactions, cyan for van der Waals forces, and orange for Pi–anion interactions.

The molecular docking results using AutoDock Vina version 1.2 indicated the binding affinities of various candidate anti-QS compounds with SarA (PDB: 2FNP). The binding energies for SarA in complex with anti-QS ligands were as follows: SarA-Isoquercetin (−7.0 kcal/mol), SarA-Quercetin 3-galactoside (−6.9 kcal/mol), SarA-Quercetin 3-beta-D-glucoside (−6.8 kcal/mol), SarA-Kaempferol 3-alpha-D-galactoside (−6.2 kcal/mol), and SarA-Kaempferol 3-alpha-D-glucoside (−6.6 kcal/mol). Among the compounds tested, Isoquercetin/Quercetin 3-O-glucoside (CID 5280804) demonstrated the strongest binding affinity among the Quercetin derivatives. While in the Kaempferol derivatives group, Kaempferol 3-alpha-D-glucoside exhibited lower binding affinities than Kaempferol 3-alpha-D-galactoside. These results suggest that Isoquercetin is the most promising candidate for anti-QS activity due to its higher binding affinities with SarA, potentially indicating stronger interactions and efficacy in disrupting QS mechanisms. Molecular docking analysis revealed the binding interactions between the SarA protein and the active ligand Isoquercetin (CID 5280804). The docking results indicated a predicted binding mode with key interactions highlighted in Figure 4A. The ligand forms van der Waals interactions with the residues LEU A:113, VAL A:116, SER A:124, LYS A:127, LYS B:123, LYS B:127, PHE B:134, ASN B:161, and GLN B:166. A pi–anion interaction is observed between the ligand’s aromatic ring system and the residue GLU B:135 at a distance of 3.36 and 3.77 Å, also with TYR B:162 (4.29 Å). The ligand forms carbon–hydrogen bonds with the residues GLN B:166 (3.32 Å) and PHE B:134 (3.64 Å). Hydrogen bonding interactions are predicted between the ligand and the residues THR A:117 (2.48 Å), ASP A:120 (3.55 Å), LYS B:127 (3.28 Å), LEU B:160 (1.84 Å) and, TYR B:162 (2.96 Å).

Figure 4B demonstrates the binding interactions between the SarA protein and the active ligand Kaempferol 3-alpha-D-glucoside (CID 44258798). Kaempferol 3-alpha-D-glucoside forms several van der Waals interactions with key residues in the active site of SarA including VAL A:116, THR A:117, LYS A:121, LYS A:123, SER A:124, LYS A:127, LYS B:127, SER B:133, PHE B:134, LEU B:160, and ASN B:161. Additionally, the ligand exhibits a pi–cation interaction with LYS B:123 at a distance of 6.93 and 6.67 Å, a pi–pi stacked interaction with TYR B:162 at 5.94 Å, and a pi–anion interaction with ASP A:161 at 5.38 Å. Hydrogen bonding interactions are also predicted with GLU B:135 (4.73 Å). These extensive interactions between representative anti-QS ligands and the SarA binding pocket suggest its potential as an effective inhibitor by disrupting the protein’s function. The docking results highlight key residues involved in stabilizing the binding complex through van der Waals forces, hydrogen bonds, and pi-interactions. The favorable binding affinity score and the predicted interaction distances align with the observed antimicrobial and anti-QS activities of this ligand in our experimental studies.

The absorption, distribution, metabolism, excretion, and toxicity (ADMET) properties of the candidate anti-quorum-sensing (anti-QS) compounds were evaluated computationally using ADMETlab 2.0 (https://admetmesh.scbdd.com, accessed on 21 May 2024). This widely used software platform incorporates updated predictive models and databases to forecast key pharmacokinetic parameters critical for drug development. The candidate anti-QS compounds were analyzed using the various ADMET prediction models in ADMETlab 2.0, which employ advanced algorithms to compute properties such as human intestinal absorption, blood–brain barrier penetration, cytochrome P450 inhibition, hepatotoxicity, and other toxicity endpoints.

The results from the in silico ADMET profiles of the identified flavonoid glycosides with potential anti-QS activity were summarized in Table S1. The quercetin glycoside derivatives (Isoquercetin, Quercetin 3-β-D-glucoside, Quercetin 3-O-glucoside, Quercetin 3-galactoside) showed good predicted human intestinal absorption, with over 90% absorbed fractions. However, they demonstrated relatively high efflux ratios, suggesting potential P-glycoprotein liability that could limit oral bioavailability. Minimal blood–brain barrier penetration was predicted for these quercetin glycosides. In terms of metabolism, they showed low risk for CYP inhibition but indicated possible hERG liability. Importantly, no hepatotoxicity or other significant toxicity risks were flagged for this class of compounds. The kaempferol glycosides (kaempferol 3-α-D-galactoside, kaempferol 3-α-D-glucoside) exhibited comparable ADMET profiles, with over 80% human intestinal absorption predicted and negligible blood–brain barrier penetration. Similar to the quercetin derivatives, high efflux ratios may limit oral exposure, while minimal CYP inhibition and no overt toxicity liabilities were observed. However, hERG inhibition was also indicated as a potential risk for this subclass. Overall, while the flavonoid glycoside hits demonstrated some favorable ADMET properties like lack of hepatotoxicity, the computational data suggest potential limitations in terms of P-glycoprotein and hERG liabilities that could impact bioavailability and safety. Further optimization may be required to improve the pharmacokinetic profiles of these anti-QS lead candidates.

The drug-likeness evaluation of anti-QS flavonoid glycosides is summarized in Table S2. All the quercetin glycoside derivatives violated Lipinski’s rule on molecular weight, exceeding 500 g/mol, while the kaempferol glycosides narrowly met this criterion. However, all compounds had eight or more H-bond donors and 11–12 H-bond acceptors, exceeding the recommended limits, which could compromise their membrane permeability. The calculated LogP values ranging from 0.45 to 1.08 were within the acceptable lipophilicity range for drug-likeness. However, the bioavailability scores of 0.55 predicted by SwissADME suggest potentially limited oral bioavailability for this series of compounds. While exhibiting some favorable physicochemical properties like moderate LogP values, the high molecular weights and excessive H-bond donors/acceptors of these flavonoid glycosides raise concerns regarding their drug-likeness and likelihood for oral absorption. Structural modifications to reduce molecular size and polarity may be required to improve the drug-likeness profiles of these anti-QS lead candidates.

## 3. Discussion

The rise of infectious diseases caused by *S. aureus* has created an urgent need for new antibacterial agents derived from natural sources. Medicinal plants could serve as a rich source of novel antibacterial compounds. This study evaluated the antibacterial potency of an extract from the leaves of *P. foetida* against bacterial strains, including biofilm-forming *S. aureus* and MRSA. The ethanolic *P. foetida* leaf crude extract exhibited numerous bioactive phytochemicals including flavonoid glycosides, promising antibacterial activity against *S. aureus*. The in vitro assay demonstrates that at a concentration of 7.81 μg/mL and 15.63 μg/mL, the ethanolic *P. foetida* leaf extract effectively inhibited the growth of *S. aureus* ATCC 25923 and MRSA, respectively. Supporting these findings, previous studies have also found that ethanolic extracts of *P. foetida* can inhibit the growth of several other bacteria. Namsena et al. (2019) reported that a 100 mg/mL *P. foetida* extract inhibited *Erwinia carotovora*, *Xanthomonas campestris*, and *Ralstonia solanacearum* [25]. Uddin et al. (2007) found that a 25 mg/mL extract was active against *Enterococcus faecalis*, *S. aureus*, *Shigella flexneri*, and *E. coli* [26]. These results suggest that both methanol and ethanol can be effective solvents for extracting antibacterial compounds from *P. foetida* [27]. 

The establishment of infection and biofilm formation in Gram-positive bacteria is orchestrated by the quorum-sensing (QS) system [28]. Treating biofilm-forming *S. aureus* infections remains a formidable challenge, largely due to the bacteria’s predominant resistance to conventional antibiotics. Our study corroborates these findings, with the *S. aureus* ATCC 25923 strain exhibiting resistance to numerous antibiotics compared to the MRSA strain, aligning with previous reports [29]. Plant-derived compounds offer a promising alternative, as they can target and disrupt various bacterial components, including the cytoplasmic membrane, cell wall, nucleic acids, porins, and enzymes. Notably, recent studies have highlighted the role of ethanolic *P. foetida* extract in inducing the lysis of bacterial cells and also inhibitory effects against drug-resistant strains. These findings underscore the potential of phytochemicals as novel therapeutic agents against multidrug-resistant biofilm-forming *S. aureus* infections.

The crude extract from *P. foetida* Linn. of this study demonstrated significant anti-biofilm and anti-quorum-sensing activities against *S. aureus*. The observed concentration-dependent effects on cell morphology, as revealed by the SEM images, suggest that the extract compromised the structural integrity of the bacterial cell wall and membrane, leading to cell lysis at higher concentrations. The wrinkled cell surfaces and increased extracellular matrix accumulation at lower concentrations (1× MIC) indicate potential interference with cell wall synthesis and biofilm formation pathways. The severe cell wall destruction, irregular dimpled surfaces, and leakage of intracellular contents at bactericidal concentrations (1× MBC) further highlight the extract’s potent antimicrobial properties. These findings are particularly promising given the growing concern over antibiotic resistance and the challenges posed by biofilm-associated infections. The ability of the *P. foetida* Linn. extract to inhibit biofilm formation and disrupt quorum-sensing signaling in *S. aureus*, coupled with its bactericidal activity, suggests potential therapeutic applications. Further investigations into the active compounds responsible for these effects, their mechanisms of action, and their efficacy in relevant infection models are warranted to fully explore the clinical potential of this natural extract in combating *S. aureus* infections, including those caused by multidrug-resistant strains. The results of our study align with previous research evaluating the anti-biofilm activities of plant extracts and their bioactive compounds against *S. aureus*. Notably, the ethanolic extract in prior studies inhibited bacterial growth at a concentration as low as 5 mg/mL. Similarly, 3-HBA demonstrated significant bactericidal activity against *S. aureus* at a concentration of 400 μg/mL. Our findings are consistent with those of Mostafa et al., who reported that ethanolic extracts of *Cuminum cyminum* inhibited MRSA at a concentration of 10 mg/mL. These comparisons suggest that our crude extract possesses a higher potential for anti-biofilm activity against *S. aureus* than previously studied extracts [30].

SarA is a key regulatory molecule in *S. aureus* that controls the expression of virulence factors, promotes biofilm formation, contributes to antibiotic resistance, and aids in bacterial adaptation and survival. Its central role in the pathogenesis of *S. aureus* makes it an important focus for understanding bacterial infection mechanisms and developing new treatments [31,32]. Molecular docking results of this study reveal that Isoquercetin and Quercetin 3-galactoside exhibit the highest binding affinities with SarA, suggesting they may be the most effective anti-QS compounds among those tested. Isoquercetin’s binding affinity of −7.0 kcal/mol and hyperoside’s −6.9 kcal/mol indicate robust interactions with SarA, potentially leading to significant inhibition of quorum-sensing pathways. In contrast, Kaempferol 3-alpha-D-glucoside and Kaempferol 3-alpha-D-galactoside, with lower binding affinities of −6.6 kcal/mol and −6.2 kcal/mol, respectively, may have comparatively weaker inhibitory effects. These findings highlight Isoquercetin and hyperoside as promising leads for further investigation and development as anti-quorum-sensing agents. Employing a molecular docking approach, we effectively identified the binding sites for anti-quorum-sensing (anti-QS) compounds. These results represent a significant breakthrough, showcasing the potential of small organic compounds to target the quorum-sensing regulator of *S. aureus*. This discovery opens avenues for pioneering antimicrobial strategies aimed at disrupting bacterial communication mechanisms. However, the crude extract is a complex mixture of diverse phytochemicals, and the observed bioactivities could potentially arise from the synergistic interactions between various components. Future studies should employ proper quantification methods, including calibration standards, to ensure accuracy. Additionally, the crude extract’s observed bioactivities may result from synergistic interactions between diverse phytochemicals, which should be further elucidated in vitro.

The data show that quercetin and kaempferol glycosides have well predicted human intestinal absorption (>80%). However, high efflux ratios suggest potential P-glycoprotein (P-gp) substrate liabilities that could limit oral bioavailability. Minimal blood–brain barrier penetration is expected based on the negative log BB values. While showing a low risk for CYP inhibition, these compounds indicate a possible hERG inhibition risk. Importantly, no overt hepatotoxicity concerns were flagged for this series by the ADMET models [33]. Despite potential P-gp liabilities and drug-likeness concerns that may limit oral bioavailability, the flavonoid glycosides (quercetin and kaempferol derivatives) show promise for several applications leveraging their predicted anti-QS or anti-virulence activity against *S. aureus*. Their good predicted human intestinal absorption, low hepatotoxicity risk, and minimal blood–brain barrier penetration make them attractive candidates for incorporation into anti-biofilm surface coatings or topical anti-virulence formulations. Moreover, when combined with conventional antibiotics, their anti-QS/anti-virulence effects could potentially re-sensitize resistant strains, reduce required antibiotic doses, and mitigate biofilm-associated tolerance. While not ideal for oral systemic delivery, these compounds could still find utility through localized/topical approaches, prodrug design, nanoformulations, or as adjuvants potentiating antibiotic therapy. Despite their drug-likeness limitations, the favorable in silico ADMET profiles rationalize further exploration of these flavonoid glycosides as anti-virulence agents against *S. aureus*, pending additional experimental validation of their bioactivities.

## 4. Materials and Methods

### 4.1. Location of Plant Collection and Bacterial Strains

**Figure 5:**
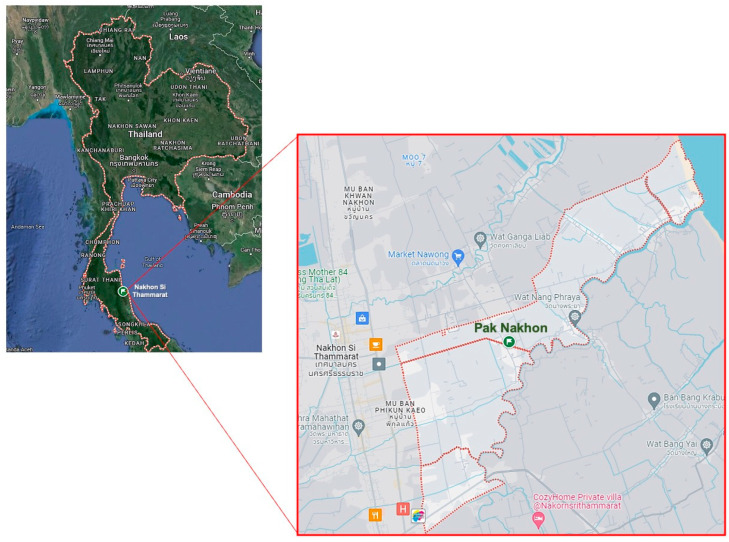
Map of Pak Nakhon Mueang Nakhon Si Thammarat, Southern Thailand. The map illustrates the plant collection site [Source: Google Maps].

Fresh leaves of *P. foetida* Linn. were collected from Pak Nakhon Mueng Nakhon Si Thammarat District, located in Nakhon Si Thammarat province, Thailand (8.47102° N 100.05395° E). The study area comprises diverse vegetation types, including tropical forests and agricultural lands (Figure 5). Bacterial strains, including *S. aureus* ATCC 25923 and methicillin-resistant *S. aureus* (MRSA) ATCC 43300 strains were purchased from American Type Culture Collection (Manassas, VA, USA). The bacteria were grown on Tryptic Soy Agar (TSA) (Difco, Claix, France) and incubated at 37 °C for 24 h. The pathogens were then inoculated in Tryptic Soy Broth (TSB) (Himedia, Nashik, India) at 37 °C for 18–24 h and stored in TSB containing 25% glycerol at −80 °C until use. For the experiment, bacterial suspensions were prepared by first adjusting the turbidity spectrophotometrically at 600 nm to achieve an optical density (OD) of 0.1. The adjusted suspension was then diluted in Tryptic Soy Broth (TSB) at a 1:4 ratio (volume/volume) to obtain an approximate concentration of 10^6^ colony-forming units per milliliter (CFU/mL) before use in the subsequent experiments.

### 4.2. Preparation of P. foetida Linn. Crude Extracts and LC-MS/MS Analysis

The crude extract was prepared using the maceration method modified from that previously described [34]. In brief, the freshly collected *P. foetida* Linn. leaves underwent a gentle wash with tap water. Subsequently, the dried leaves were ground, with around 10 g of leaves soaked in 100 mL of 95% ethanol (1:10 *w*/*v*) for 7 days at 28.5 °C, agitated at 120 rpm. Afterward, the supernatant was filtered through Whatman No. 1 filter paper (GE Healthcare Life Science, Buckinghamshire, UK) and then the mixture was concentrated using a rotary evaporator at 40 °C under reduced pressure. The resulting dry extract was air-dried at room temperature to remove any remaining solvent and maintain its weight. Then the crude extract was stored at 4 °C until use, and for subsequent analysis, it was dissolved in dimethyl sulfoxide (DMSO).

For metabolomics analysis of *P. foetida* Linn. crude extracts, liquid chromatography-tandem mass spectrometry (LC-MS/MS) was used. In brief, the crude extract samples were resuspended in 250 µL of 0.1% formic acid in deionized Water-LC-MS CHROMASOLV from Honeywell (Wunstorfer Strasse, Seelze, Germany). Afterward, the supernatant underwent filtration using a 0.45 µm pore size hydrophilic nylon syringe filter for LC-MS/MS analysis, with 100 µL of the prepared sample collected in an insert within LC glass vials. ESI-QTOF mass spectrometry was employed to identify active ingredients exhibiting specific activities. Separation was conducted using a DIONEX Ultimate 3000 HPLC (Dionex Softron, GmbH, Germering, Germany) system with an Acclaim PolarAdvantageII C18 (2.1 × 100 mm, 3 μm) column and an Acclaim PolarAdvantageII C18 (3 × 10 mm, 5 μm) guard column. The injection volume was 3 µL, and column and autosampler temperatures were maintained at 40 °C and 10 °C, respectively. The detection was performed with a Bruker compact QTOF mass spectrometer (Bruker, Bremen, Germany). MS signals were acquired in the *m*/*z* range of 50–1000 under positive ion mode [35]. The nebulizing gas pressure, the drying gas flow, and the drying gas temperature were set at 2 bars, 8 L/min, and 220 °C, respectively. The separation was performed at a flow rate of 0.3 mL/min under a gradient program in which mobile phases A (water with 0.1% formic acid) and B (acetonitrile with 0.1% formic acid) were employed. The gradient program was: 0–2 min, 99% A: 1% B; 2–17 min, 99–1% A: 1–99% B; 17–20 min, 99% B: 1% A. The flow rate was fixed at 0.25 mL/min. Subsequently, 20–20.1 min, 99% B to 99% A; 20.1–28.5 min, 99% A: 1% B at 0.35 mL/min flow rate; 28.5–30 min; 99% A: 1% B at 0.25 mL/min. Data processing utilized Compass Data Analysis 5.3 software (Bruker Daltonics, Bremen, Germany), and metabolite identification relied on matching MS/MS spectra and retention time in the Bruker MetaboBASE Personal Library 2.0 (Bruker Daltonics, Germany).

### 4.3. Minimal Inhibitory Concentration (MIC) and Minimal Bactericidal Concentration (MBC) of Ethanolic P. foetida Linn. Crude Extract against S. aureus

The antibacterial efficacy of the extract against both MSSA and MRSA strains was assessed with modification using the broth microdilution method as previously outlined [36]. In brief, the crude extract of *P. foetida* Linn. was diluted in a 96-well microtitre plate to final concentrations ranging from 62.50 μg/mL to 0.12 μg/mL in Mueller–Hinton broth (MHB) (Difco, Claix, France). Each well was inoculated with 100 μL of bacterial suspension (1 × 10^6^ CFU/mL) and then incubated at 37 °C for 18 h. tetracycline at 512 µg/mL served as the positive control, while broth with 1% DMSO was used as the negative control. To determine the minimum inhibitory concentration (MIC), 0.03% resazurin (Thermo Fisher Scientific, Lancashire, UK) was added to each well. The MIC was defined as the lowest concentration that completely inhibited bacterial growth, indicated by a blue color change [37]. Crude extract MBC was assessed by streaking cultures onto TSA plates. All experiments were conducted in triplicate.

### 4.4. Scanning Electron Microscopy Analysis and Imaging

The impact of *P. foetida* Linn. extract on the cell morphology of MSSA and MRSA was investigated using SEM, modified from previously mentioned [38] with slight adjustments. Initially, bacterial cells were cultured in Mueller–Hinton broth (MHB) and incubated at 37 °C for 18 h. Afterward, the bacterial cells (1 × 10^6^ CFU/mL) were exposed to the extract at concentrations of 1× MIC and 1× MBC in a centrifuge tube, followed by further incubation at 37 °C for 24 h. A final concentration of MHB containing 1% DMSO was maintained as a negative control. Also, the media supplemented tetracycline (Sigma-Aldrich, St. Louis, MA, USA) as a positive control. Samples were then centrifuged at 5000 rpm for 5 min, and the resulting bacterial pellet was air-dried on a sterile glass coverslip (0.5 cm × 0.5 cm). These specimens were fixed in 2.5% glutaraldehyde for 2 h, followed by dehydration using a graded ethanol series (20–100%) for 30 min at each step. Afterward, specimens were mounted on aluminum stubs and dried using a critical point dryer, then coated with gold particles. Finally, SEM imaging was conducted to observe the bacterial morphology post-extract treatment, utilizing SEM-Zeiss (Munich, Germany) at the Center for Scientific and Technological Equipment, Walailak University.

### 4.5. Biofilm Formation Inhibition Using Microtiter Plates Assay

To assess the biofilm inhibition potential of the crude extract against *S. aureus* ATCC 25923, a microtiter plate assay modified from the method previously mentioned by Gajewska et al. 2020 [39] was used. In brief, *S. aureus* ATCC 25923 was cultured in Luria–Bertani broth (LB) at 37 °C for 24 h. Subsequently, 200 microliters of a 1:100 dilution of overnight cultures adjusted to 0.5 McFarland turbidity, were mixed with varying concentrations of the extracts or fractions (1/2× MIC, 1/4× MIC, 1/8× MIC, and 1/16× MIC) in fresh LB supplemented with 1% (*w*/*v*) glucose were dispensed into wells, followed by incubation at 37 °C for 24 h. Sub-inhibitory concentrations relative to the minimum inhibitory concentration (MIC) were tested to assess the ability to interfere with biofilm formation without exerting a bactericidal effect. Wells containing biofilm were gently washed with phosphate-buffered saline (PBS) pH 7.4 and air-dried at room temperature. The biofilm was stained with a 0.4% (*w*/*v*) crystal violet solution (200 μL) for 15 min. Subsequently, wells were rinsed three times with distilled water to remove excess dye. The biofilms were dissolved in ethanol, and the optical density (OD) was measured at 570 nm.

### 4.6. QS Gene Expression Using Quantitative Reverse Transcription PCR (qRT-PCR)

The impact of crude extracts on the quorum-sensing (QS) system in *S. aureus* was assessed by measuring the transcriptional expression levels of representative QS-associated genes (*sarA*, *hla*, and *ica*) using qRT-PCR. In brief, *S. aureus* ATCC 25923 was grown in the presence of extract (varied concentrations from 1/2× MIC, 1/4× MIC, and 1/8× MIC) or without the test compound at 37 °C for 16 h. After incubation, the test tube walls were scraped with a sterile cell scraper to resuspend adhered cells. The total cell population (planktonic and biofilm) was harvested by centrifugation from the grown cultures (0.5 mL). Total RNA was extracted from bacterial cells using the RNeasy Plus Mini Kit (QIAGEN, Hilden, Germany) according to the manufacturer’s instructions. Briefly, bacterial cultures were centrifuged, and cell pellets were resuspended in RNAprotect^®^ Bacteria Reagent to stabilize RNA. After a brief incubation at room temperature, cells were lysed with a buffer containing β-mercaptoethanol and subjected to mechanical disruption. The lysate was then loaded onto RNeasy Mini spin columns, where RNA selectively bound to the silica membrane while contaminants were removed by washing with RW1 and RPE buffers. Columns were centrifuged to dry the membrane, and RNA was eluted in RNase-free water. RNA concentration and purity were determined by optical density measurements at OD260 using a spectrophotometer (Thermo Scientific NanoDrop, Wilmington, DE, USA) before proceeding with qRT-PCR analysis.

The qRT-PCR was employed to quantify the impact of the crude extract on the transcriptional regulation of virulence factors and biofilm formation genes controlled by the QS system in *S. aureus*. It should be noted that the assay was performed using the crude extract, which contains multiple components, rather than a single isolated compound. The sequences of primers used in this study are provided in Table 2. The reaction mixture, with a total volume of 20 μL, consisted of 10 μL 2× SYBR Green PCR Master Mix, forward and reverse primers (1 μL each), 4 μL of nuclease-free water, and 4 μL of 20× diluted cDNA. PCR conditions included an initial denaturation at 95 °C for 2 min, followed by 40 cycles of denaturation (95 °C for 15 s), annealing (55.8 °C for 15 s), and extension (72 °C for 20 s). To ensure the samples were free from contamination, negative controls containing nuclease-free water instead of RNA were run in parallel. The relative gene expression was analyzed using the 2^−ΔΔCt^ method with *16S rRNA* as a reference gene. 

### 4.7. In Silico Analysis of Anti-QS Activity of Candidate Bioactive Compounds

In silico molecular docking studies were performed to investigate the binding interactions between candidate anti-QS compounds and the QS receptors SarA of *S. aureus*. The structures of phytochemicals used for the study were retrieved from the NCBI-PubChem database (https://pubchem.ncbi.nlm.nih.gov/, accessed on 21 May 2024) and prepared using AutoDock, including two 2D-to-3D structure conversion, generation of three-dimensional (3D) coordinates, and energy minimization for optimization [40]. The 3D structures of *S. aureus* SarA (PDB ID: 2FNP) were downloaded from the Protein Data Bank (PDB) (https://www.rcsb.org, accessed on 21 May 2024), and protein structures were prepared using the dock prep method of AutoDock, removing water molecules and other components from the crystal configuration [41]. Hydrogen atoms were included in the existing carbon atoms to evaluate the structure and assign bond orders. The refined and optimized protein structures were processed using MGLTools version 1.5.7 to specify the location of the receptor site. Active site residues bound to the co-crystallized ligand were used as the centroid for receptor grid generation, with an automatic setting of the enclosing box [42]. For the molecular docking process, the 3D structure of the candidate anti-QS ligands (Isoquercetin/Quercetin 3-beta-D-glucoside; Quercetin 3-galactoside; Kaempferol 3-alpha-D-galactoside; and Kaempferol 3-alpha-D-glucoside) was separately docked into the receptor binding pocket of each transcriptional regulator using the AutoDock Vina docking software version 1.2 [40,43]. The conformation of the QS receptor–ligand complex with the lowest binding free energy (ΔG) at the optimal docking position was selected for further interaction analysis and visualization through the Discovery Studio Visualizer 3.5 program (Dassault Systèmes BIOVIA, San Diego, CA, USA).

The absorption, distribution, metabolism, excretion and toxicity (ADMET) properties of the candidate compounds were evaluated computationally using ADMETlab 2.0 (https://admetmesh.scbdd.com, accessed on 21 May 2024), a widely recognized platform for predicting these critical pharmacokinetic parameters. ADMETlab 2.0 incorporates significant updates to its functional modules, predictive models, explanations, and user interface, enhancing its capacity to assist medicinal chemists in accelerating the drug research and development process. The candidate anti-QS compounds were analyzed using the various ADMET prediction models available in the software, which employs advanced algorithms and databases to compute properties such as human intestinal absorption, blood–brain barrier penetration, cytochrome P450 inhibition, hepatotoxicity, and other toxicity endpoints. This in silico assessment provided valuable insights into the pharmacokinetic profiles and potential toxicity liabilities of the compounds, facilitating the prioritization and selection of promising candidates for further evaluation [44]. Moreover, we employed SwissADME to analyze the drug-likeness of the anti-quorum-sensing (anti-QS) flavonoid glycoside compounds. Initially, the chemical structures of these compounds, which included quercetins and kaempferol glycosides, were entered into the SwissADME web interface (http://www.swissadme.ch, accessed on 15 April 2024). This platform utilizes computational algorithms to predict essential pharmacokinetic parameters such as gastrointestinal absorption, blood–brain barrier permeability, and potential interactions with P-gc. Furthermore, SwissADME evaluates drug-likeness based on Lipinski’s rule of five and other established guidelines for oral bioavailability and drug safety. The outcomes generated by SwissADME were subsequently scrutinized to gauge the overall drug-likeness profile of the flavonoid glycoside compounds, thereby offering crucial insights into their potential as promising candidates for anti-QS therapeutics.

**Table 2:** Primer sequences for qRT-PCR analysis of quorum-sensing gene expression.

Genes	Forward Primer (5′ to 3′)	Reverse Primer (5′ to 3′)	PCR Product (bp)	References
*16S rRNA*	TGATCCTGGCTCAGGATGA	TTCGCTCGACTTGCATGTA	57	[45]
*sarA*	GCTGTATTGACATACATCAGCGAA	CGTTGTTTGCTTCAGTGATTCGT	250	[46]
*hla*	ACAATTTTAGAGAGCCCAACTGAT	TCCCCAATTTTGATTCACCAT	77	[45]
*ica*	TCGCACTCTTTATTGATAGTCGCTACGAG	TGCGACAAGAACTACTGCTGCGTTAAT	98	[46]

### 4.8. Statistical Analysis

The results are reported as mean values with standard deviations, based on three independent experiments. Statistical comparisons between the control and test groups were conducted using one-way ANOVA, followed by Tukey’s post hoc test, utilizing GraphPad Prism 5 software (La Jolla, CA, USA). A significance level of *p* < 0.05 was considered statistically significant.

## 5. Conclusions

In conclusion, this study highlights the therapeutic potential of *P. foetida* Linn. leaf extract against *S. aureus*. The ethanolic extract demonstrated inhibitory effects on *S. aureus* growth, biofilm formation, and quorum-sensing (QS) regulation. Real-time PCR analysis revealed the extract’s ability to downregulate key virulence and biofilm genes, while LC-MS/MS analysis identified quercetin and kaempferol glycosides as potential bioactive constituents. Molecular docking studies explored their binding to the QS transcriptional regulator SarA. Despite concerns regarding drug-likeness and permeability, this integrated in vitro–in silico investigation offers valuable insights for developing novel anti-virulence strategies to combat antimicrobial resistance in *S. aureus*. Further experimental validation is warranted to confirm its efficacy and explore its clinical applications in addressing the global antibiotic resistance crisis and reducing the burden of healthcare-associated infections.

## Figures and Tables

**Table 1:** PubChem database details of candidate anti-QS bioactive compounds in ethanolic crude extract of *P. foetida* Linn. identified by LC-MS/MS in the positive ionization mode.

No.	Name of Bioactive Compounds and PubChem CID	Structure of the Bioactive Compounds	Molecular Formula	XLogP	Hydrogen Bond Donor	Hydrogen Bond Acceptor	Retention Time (min)	Mass-to-Charge Ratio (*m*/*z*)
1.	Isoquercetin (CID 5280804)	* 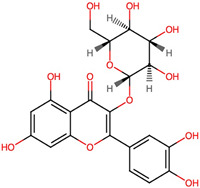 *	C_21_H_20_O_12_	0.4	8	12	9.22	303.0497
2.	Quercetin 3-galactoside (CID 5281643)	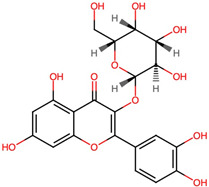	C_21_H_20_O_12_	0.4	8	12	9.22	303.0493
3.	Quercetin 3-beta-D-glucoside (CID 44259136)	* 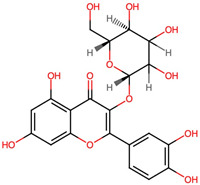 *	C_21_H_20_O_12_	0.4	8	12	9.22	303.0503
4.	Kaempferol 3-alpha-D-galactoside (CID 44258736)	* 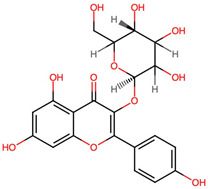 *	C_21_H_20_O_11_	0.7	7	11	9.60	287.0536
5.	Kaempferol 3-alpha-D-glucoside (CID 44258798)	* 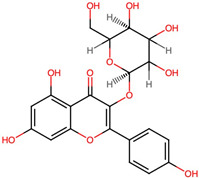 *	C_21_H_20_O_11_	0.7	7	11	9.63	287.0532

## Data Availability

Data are contained within the article and Supplementary Materials.

## Supplementary Materials

The following supporting information can be downloaded at: https://www.mdpi.com/article/10.3390/antibiotics13070613/s1. Data S1: Tables S1 and S2; Data S2: Compound Spectrum List Report.
