# Supplementary material for: Unveiling the Antimicrobial, Anti-Biofilm, and Anti-Quorum-Sensing Potential of Paederia foetida Linn. Leaf Extract against Staphylococcus aureus: An Integrated In Vitro–In Silico Investigation
Source: Antibiotics (Basel). 2024 Jul 1;13(7):613. doi: 10.3390/antibiotics13070613 (PMC11273848; doi:10.3390/antibiotics13070613)
Supplement: Supplementary file 1 [file antibiotics-13-00613-s001.zip › SupplementaryDataS1.pdf]

Supplementary data

Unveiling the Antimicrobial, Anti-Biofilm, and Anti-Quorum-Sensing Potential of *Paederia foetida* Linn. Leaf Extract against *Staphylococcus aureus*: An Integrated In Vitro–In Silico Investigation

Sirijan Santajit [1,2], Witawat Tunyong [3], Dararat Horpet [1], Asma Binmut [1], Thida Kong-Ngoen [3], Churaibhon Wisessaowapak [4], Techit Thavorasak [5], Pornpan Pumirat [3] and Nitaya Indrawattana [3,6,*]

[1] Department of Medical Technology, School of Allied Health Sciences, Walailak University, Tha Sala 80160, Thailand; sirijan.sa@wu.ac.th (S.S.); pdararat@wu.ac.th (D.H.); abilmud@gmail.com (A.B.)
[2] Research Center in Tropical Pathobiology, Walailak University, Tha Sala 80160, Thailand
[3] Department of Microbiology and Immunology, Faculty of Tropical Medicine, Mahidol University, Bangkok 10400, Thailand; witawat.tun@mahidol.ac.th (W.T.); thida.kon@mahidol.ac.th (T.K.-N.); pornpan.pum@mahidol.ac.th (P.P.)
[4] Department of Medicine and Pharmacology, University of California, San Diego, CA 92093, USA; chwisessaowapak@health.ucsd.edu
[5] Center of Research Excellence in Therapeutic Proteins and Antibody Engineering, Department of Parasitology, Faculty of Medicine Siriraj Hospital, Mahidol University, Bangkok 10700, Thailand; techit.tha@mahidol.edu
[6] Siriraj Center of Research Excellence in Allergy and Immunology, Department of Research, Faculty of Medicine Siriraj Hospital, Mahidol University, Bangkok 10700, Thailand
* Correspondence: nitaya.ind@mahidol.ac.th; Tel.: +66-2-354-9100 (ext. 1598)

TableS1: *In silico* ADMET prediction for flavonoid glycosides with anti-QS potential

Property	Isoquercetin (CID 5280804)#	Quercetin 3-galactoside (CID 5281643)	Quercetin 3-beta-D-glucoside (CID 44259136)	Kaempferol 3-α-D-galactoside (CID 44258736)	Kaempferol 3-α-D-glucoside (CID 44258798)
**Solubility (LogS)**	-4.2 log mol/L	-4.1 log mol/L	-3.27 log mol/L	-4.3 log mol/L	-4.3 log mol/L
**Human Intestinal Absorption**	Medium	Medium	Medium	Medium	Medium
**Caco-2 Permeability**	Low	Low	Low	Low	Low
**Plasma Protein Binding (PPB)**	95%	94%	69.89%	95%	95%
**Blood-Brain Barrier (BBB)**	Low	Low	Low	Low	Low
**hERG Inhibition**	Low risk	Low risk	Low risk	Low risk	Low risk
**Hepatotoxicity**	Low Risk	Low Risk	Low risk	Low Risk	Low Risk
**LD50 (Acute Toxicity)**	2500 mg/kg	2400 mg/kg	2110 mg/kg	2600 mg/kg	2600 mg/kg

TableS2: Physicochemical Properties and Bioavailability Parameters for Anti-QS Flavonoid Glycosides

Compound	MW (g/mol)#	LogP	H-Bond Donors	H-Bond Acceptors	Lipinski Violations	Bioavailability Score
Isoquercetin	464.38	1.08	8	12	1 (MW>500)	0.55
Quercetin 3-galactoside	464.38	1.04	8	12	1 (MW>500)	0.55
Quercetin 3-β-D-glucoside	464.38	1.08	8	12	1 (MW>500)	0.55
Kaempferol 3-α-D-galactoside	448.38	0.45	8	11	0	0.55
Kaempferol 3-α-D-glucoside	448.38	0.45	8	11	0	0.55

MW: Molecular Weight

Lipinski Violations: Violations of Lipinski's rule of five.
