# Supplementary material for: Unveiling the Antimicrobial, Anti-Biofilm, and Anti-Quorum-Sensing Potential of Paederia foetida Linn. Leaf Extract against Staphylococcus aureus: An Integrated In Vitro–In Silico Investigation
Source: Antibiotics (Basel). 2024 Jul 1;13(7):613. doi: 10.3390/antibiotics13070613 (PMC11273848; doi:10.3390/antibiotics13070613)
Supplement: Supplementary file 1 [file antibiotics-13-00613-s001.zip › SupplementaryDataS2.pdf]

Compound Spectrum List Report

Analysis Info

		Acquisition Date	6/23/2023 5:27:59 PM
Analysis Name	D:\Data\Supitcha\metabolites26062023\PF_EtOH_pos_MSMS_RA7_01_12675.d		
Method	msms_positive_210666.m	Operator	tof-user
Sample Name	PF_EtOH_pos_MSMS	Instrument	compact 8255754.20128
Comment			

Acquisition Parameter

Source Type	ESI	Ion Polarity	Positive	Set Nebulizer	0.3 Bar
Focus	Not active	Set Capillary	4500 V	Set Dry Heater	200 °C
Scan Begin	20 m/z	Set End Plate Offset	-500 V	Set Dry Gas	4.0 l/min
Scan End	1000 m/z	Set Charging Voltage	2000 V	Set Divert Valve	Waste
		Set Corona	0 nA	Set APCI Heater	0 °C

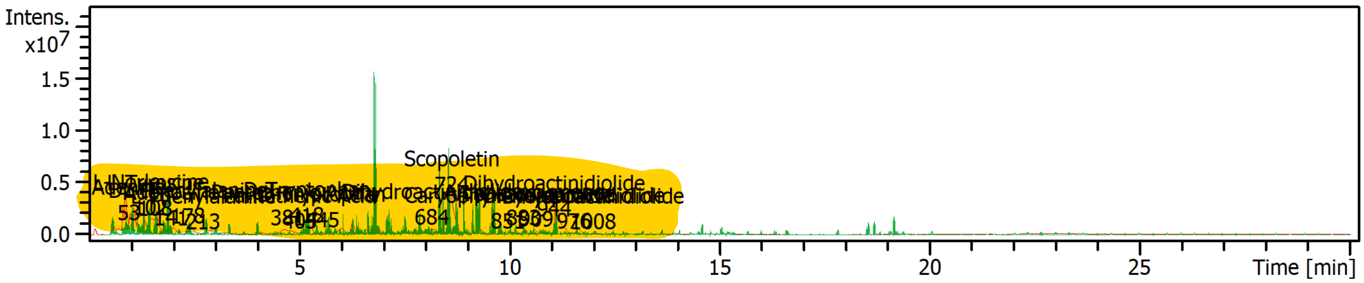

#	RT [min]	I	MS/MS	Purity'	Compound Name	Library
28	0.71	2500775	x	960	Choline	Bruker MetaboBASE Personal Library 2.0
43	0.86	192822	x	885	Glycerophosphocholine	Bruker MetaboBASE Personal Library 2.0
51	0.93	73280	x	787	Kojibiose	Bruker MetaboBASE Personal Library 2.0
53	0.94	2016456	x	782	Adenine	Bruker MetaboBASE Personal Library 2.0
72	1.14	102806	x	620	Guanine	Bruker MetaboBASE Personal Library 2.0
73	1.15	2123971	x	940	L-Norleucine	Bruker MetaboBASE Personal Library 2.0
78	1.19	1410278	x	630	Niacinamide	Bruker MetaboBASE Personal Library 2.0
86	1.31	351868	x	479	.alpha.-D-Glucose	Bruker MetaboBASE Personal Library 2.0
			x	420	D-Tagatose	Bruker MetaboBASE Personal Library 2.0
			x	410	L-(-)Sorbose	Bruker MetaboBASE Personal Library 2.0
90	1.36	73280	x	732	Kojibiose	Bruker MetaboBASE Personal Library 2.0
102	1.47	2123971	x	943	L-Norleucine	Bruker MetaboBASE Personal Library 2.0
			x	806	L-Isoleucine	Bruker MetaboBASE Personal Library 2.0
			x	794	L-Alloisoleucine	Bruker MetaboBASE Personal Library 2.0
			x	733	L-Leucine	Bruker MetaboBASE Personal Library 2.0
104	1.54	1410278	x	723	Isonicotinic acid	Bruker MetaboBASE Personal Library 2.0
108	1.56	1864357	x	704	L-Tyrosine	Bruker MetaboBASE Personal Library 2.0
127	1.78	381677	x	965	2-Pyrrolidinone	Bruker MetaboBASE Personal Library 2.0
130	1.80	83958	x	972	Uridine	Bruker MetaboBASE Personal Library 2.0
			x	815	Azacitidine	Bruker MetaboBASE Personal Library 2.0
			x	808	Ribavirin	Bruker MetaboBASE Personal Library 2.0
141	1.95	691931	x	991	Adenosine	Bruker MetaboBASE Personal Library 2.0
			x	990	Vidarabine	Bruker MetaboBASE Personal Library 2.0
168	2.21	107882	x	957	2-Pyrrolidinone	Bruker MetaboBASE Personal Library 2.0
178	2.34	1026229	x	952	DL-Phenylalanine	Bruker MetaboBASE Personal Library 2.0
			x	951	L-Phenylalanine	Bruker MetaboBASE Personal Library 2.0
213	2.71	1026229	x	960	L-Phenylalanine	Bruker MetaboBASE Personal Library 2.0
			x	958	DL-Phenylalanine	Bruker MetaboBASE Personal Library 2.0
262	3.34	28991	x	583	1,2,3-Trihydroxybenzene	Bruker MetaboBASE Personal Library 2.0
289	3.67	27741	x	977	2'-O-Methyladenosine	Bruker MetaboBASE Personal Library 2.0
373	4.63	152969	x	791	4-METHYLDAPHNETIN	Bruker MetaboBASE Personal Library 2.0
381	4.72	408910	x	804	Pantothenic Acid	Bruker MetaboBASE Personal Library 2.0
405	5.01	408910	x	677	Pantothenic Acid	Bruker MetaboBASE Personal Library 2.0
418	5.17	798327	x	739	D-Tryptophan	Bruker MetaboBASE Personal Library 2.0
			x	653	L-Tryptophan	Bruker MetaboBASE Personal Library 2.0
			x	629	DL-Tryptophan	Bruker MetaboBASE Personal Library 2.0
445	5.55	798327	x	729	D-Tryptophan	Bruker MetaboBASE Personal Library 2.0
			x	644	L-Tryptophan	Bruker MetaboBASE Personal Library 2.0

Compound Spectrum List Report

#	RT [min]	I	MS/MS	Purity'	Compound Name	Library
			x	623	DL-Tryptophan	Bruker MetaboBASE Personal Library 2.0
480	5.89	31207	x	819	4-METHYLDAPHNETIN	Bruker MetaboBASE Personal Library 2.0
482	5.92	26376	x	585	2,6-Dihydroxynaphthalene	Bruker MetaboBASE Personal Library 2.0
497	6.12	38672	x	909	Acetyl-DL-Valine	Bruker MetaboBASE Personal Library 2.0
518	6.34	52953	x	804	Norharman	Bruker MetaboBASE Personal Library 2.0
545	6.58	239751	x	497	p-Coumaric acid	Bruker MetaboBASE Personal Library 2.0
546	6.61	148603	x	666	PHENACYLAMINE	Bruker MetaboBASE Personal Library 2.0
554	6.68	229741	x	546	Methyl cinnamate	Bruker MetaboBASE Personal Library 2.0
579	6.92	92486	x	704	m-Coumaric acid	Bruker MetaboBASE Personal Library 2.0
			x	657	p-Coumaric acid	Bruker MetaboBASE Personal Library 2.0
591	7.08	81336	x	951	Chlorogenic Acid	Bruker MetaboBASE Personal Library 2.0
615	7.34	204403	x	768	Scytalone	Bruker MetaboBASE Personal Library 2.0
			x	621	Ferulic acid	Bruker MetaboBASE Personal Library 2.0
623	7.46	41327	x	803	m-Coumaric acid	Bruker MetaboBASE Personal Library 2.0
			x	770	p-Coumaric acid	Bruker MetaboBASE Personal Library 2.0
631	7.53	204403	x	748	Scytalone	Bruker MetaboBASE Personal Library 2.0
			x	618	Ferulic acid	Bruker MetaboBASE Personal Library 2.0
648	7.73	72690	x	662	Sabinol	Bruker MetaboBASE Personal Library 2.0
			x	628	Thujone	Bruker MetaboBASE Personal Library 2.0
			x	623	(1S,4R)-p-Mentha-2,8-dien-1-ol	Bruker MetaboBASE Personal Library 2.0
			x	546	(4R,6R)-Cis-Carveol	Bruker MetaboBASE Personal Library 2.0
649	7.75	115296	x	843	p-Mentha-1,3,8-triene	Bruker MetaboBASE Personal Library 2.0
			x	810	(S)-(-)-Perillyl alcohol	Bruker MetaboBASE Personal Library 2.0
653	7.80	67359	x	602	FRAXETIN	Bruker MetaboBASE Personal Library 2.0
659	7.85	233387	x	948	Chlorogenic Acid	Bruker MetaboBASE Personal Library 2.0
671	7.99	115296	x	435	p-Mentha-1,3,8-triene	Bruker MetaboBASE Personal Library 2.0
684	8.16	497133	x	450	Dihydroactinidiolide	Bruker MetaboBASE Personal Library 2.0
713	8.51	39453	x	869	o-Xylene	Bruker MetaboBASE Personal Library 2.0
			x	763	m-Xylene	Bruker MetaboBASE Personal Library 2.0
724	8.64	432280	x	729	Scopoletin	Bruker MetaboBASE Personal Library 2.0
728	8.69	149233	x	465	Methyl jasmonate	Bruker MetaboBASE Personal Library 2.0
730	8.70	137209	x	673	Sabinol	Bruker MetaboBASE Personal Library 2.0
			x	668	(1S,4R)-p-Mentha-2,8-dien-1-ol	Bruker MetaboBASE Personal Library 2.0
			x	657	Dihydrocarvone	Bruker MetaboBASE Personal Library 2.0
			x	616	Thujone	Bruker MetaboBASE Personal Library 2.0
			x	553	(4R,6R)-Cis-Carveol	Bruker MetaboBASE Personal Library 2.0
			x	545	(1R,4R)-Dihydrocarvone	Bruker MetaboBASE Personal Library 2.0
			x	529	(-)-Trans-Pinocarveol	Bruker MetaboBASE Personal Library 2.0
743	8.86	39453	x	908	o-Xylene	Bruker MetaboBASE Personal Library 2.0
747	8.91	34504	x	949	Rutin	Bruker MetaboBASE Personal Library 2.0
749	8.95	169554	x	740	Alpha-curcumene	Bruker MetaboBASE Personal Library 2.0
750	8.96	149742	x	860	p-Mentha-1,3,8-triene	Bruker MetaboBASE Personal Library 2.0
			x	824	(S)-(-)-Perillyl alcohol	Bruker MetaboBASE Personal Library 2.0
752	8.98	137209	x	657	Sabinol	Bruker MetaboBASE Personal Library 2.0
			x	627	(1S,4R)-p-Mentha-2,8-dien-1-ol	Bruker MetaboBASE Personal Library 2.0
			x	610	Dihydrocarvone	Bruker MetaboBASE Personal Library 2.0
			x	602	Thujone	Bruker MetaboBASE Personal Library 2.0
			x	546	(4R,6R)-Cis-Carveol	Bruker MetaboBASE Personal Library 2.0
			x	509	(-)-Trans-Pinocarveol	Bruker MetaboBASE Personal Library 2.0
			x	503	(1R,4R)-Dihydrocarvone	Bruker MetaboBASE Personal Library 2.0
764	9.09	40349	x	789	p-Coumaric acid	Bruker MetaboBASE Personal Library 2.0
			x	779	m-Coumaric acid	Bruker MetaboBASE Personal Library 2.0
772	9.16	101471	x	480	Capsidiol	Bruker MetaboBASE Personal Library 2.0
778	9.22	71703	x	970	Quercetin 3-.beta.-D-glucoside	Bruker MetaboBASE Personal Library 2.0
			x	975	Delphinidin 3-O-galactoside	Bruker MetaboBASE Personal Library 2.0
			x	981	Quercetin 3-O-glucoside	Bruker MetaboBASE Personal Library 2.0
			x	976	Myrtillin	Bruker MetaboBASE Personal Library 2.0
			x	973	Isoquercitrin	Bruker MetaboBASE Personal Library 2.0
			x	973	Quercetin 3-galactoside	Bruker MetaboBASE Personal Library 2.0
783	9.27	169554	x	549	Alpha-curcumene	Bruker MetaboBASE Personal Library 2.0
803	9.45	58713	x	478	(R)-8-Acetoxycarvotanacetone	Bruker MetaboBASE Personal Library 2.0
805	9.48	47039	x	797	Limonene-1,2-epoxide	Bruker MetaboBASE Personal Library 2.0
			x	659	Sabinol	Bruker MetaboBASE Personal Library 2.0
			x	641	Dihydrocarvone	Bruker MetaboBASE Personal Library 2.0

Compound Spectrum List Report

#	RT [min]	I	MS/MS	Purity'	Compound Name	Library
			x	622	(1S,4R)-p-Mentha-2,8-dien-1-ol	Bruker MetaboBASE Personal Library 2.0
			x	614	Thujone	Bruker MetaboBASE Personal Library 2.0
			x	577	(-)-Trans-Pinocarveol	Bruker MetaboBASE Personal Library 2.0
			x	571	(4R,6R)-Cis-Carveol	Bruker MetaboBASE Personal Library 2.0
			x	556	(1R,4R)-Dihydrocarvone	Bruker MetaboBASE Personal Library 2.0
811	9.53	56593	x	779	p-Coumaric acid	Bruker MetaboBASE Personal Library 2.0
			x	766	m-Coumaric acid	Bruker MetaboBASE Personal Library 2.0
817	9.59	104059	x	970	Petunidin 3-galactoside	Bruker MetaboBASE Personal Library 2.0
			x	969	Petunidin 3-glucoside	Bruker MetaboBASE Personal Library 2.0
			x	963	Isorhamnetin 3-glucoside	Bruker MetaboBASE Personal Library 2.0
818	9.59	98650	x	964	Chrysanthemin	Bruker MetaboBASE Personal Library 2.0
			x	969	Astragalin	Bruker MetaboBASE Personal Library 2.0
			x	980	Kaempferol 3-alpha-D-glucoside	Bruker MetaboBASE Personal Library 2.0
			x	970	KAEMPFEROL-7-O-GLUCOSIDE	Bruker MetaboBASE Personal Library 2.0
			x	975	Kaempferol 3-alpha-D-galactoside	Bruker MetaboBASE Personal Library 2.0
821	9.62	169554	x	703	Alpha-curcumene	Bruker MetaboBASE Personal Library 2.0
833	9.76	40317	x	666	Limonene-1,2-epoxide	Bruker MetaboBASE Personal Library 2.0
			x	563	(R)-(+)-Pulegone	Bruker MetaboBASE Personal Library 2.0
			x	575	Sabinol	Bruker MetaboBASE Personal Library 2.0
			x	566	(1S,4R)-p-Mentha-2,8-dien-1-ol	Bruker MetaboBASE Personal Library 2.0
			x	563	(-)-Trans-Pinocarveol	Bruker MetaboBASE Personal Library 2.0
			x	552	(4R,6R)-Cis-Carveol	Bruker MetaboBASE Personal Library 2.0
			x	546	Dihydrocarvone	Bruker MetaboBASE Personal Library 2.0
			x	494	(1R,4R)-Dihydrocarvone	Bruker MetaboBASE Personal Library 2.0
			x	473	Thujone	Bruker MetaboBASE Personal Library 2.0
842	9.86	81130	x	302	Achillin	Bruker MetaboBASE Personal Library 2.0
848	9.94	338582	x	553	Megastigmatrienone	Bruker MetaboBASE Personal Library 2.0
851	9.97	538263	x	766	Caryophyllene epoxide	Bruker MetaboBASE Personal Library 2.0
			x	726	Spathulenol	Bruker MetaboBASE Personal Library 2.0
			x	688	Longifolenaldehyde	Bruker MetaboBASE Personal Library 2.0
			x	683	Hinesol	Bruker MetaboBASE Personal Library 2.0
856	10.03	37796	x	541	CRASSIN ACETATE	Bruker MetaboBASE Personal Library 2.0
			x	366	8-Iso-PGE2	Bruker MetaboBASE Personal Library 2.0
857	10.04	311425	x	755	Alpha-curcumene	Bruker MetaboBASE Personal Library 2.0
864	10.12	28489	x	510	(+)-Abscisic Acid	Bruker MetaboBASE Personal Library 2.0
			x	471	(.plusminus.)Abscisic Acid	Bruker MetaboBASE Personal Library 2.0
883	10.34	441471	x	755	Alpha-curcumene	Bruker MetaboBASE Personal Library 2.0
888	10.40	338582	x	582	Megastigmatrienone	Bruker MetaboBASE Personal Library 2.0
909	10.65	441471	x	715	Alpha-curcumene	Bruker MetaboBASE Personal Library 2.0
912	10.66	27805	x	579	Traumatic Acid	Bruker MetaboBASE Personal Library 2.0
916	10.75	32253	x	427	Cymathere lactone	Bruker MetaboBASE Personal Library 2.0
925	10.83	159399	x	564	Megastigmatrienone	Bruker MetaboBASE Personal Library 2.0
926	10.84	88267	x	765	9(S)-HpOTrE	Bruker MetaboBASE Personal Library 2.0
			x	587	13(S)-HpOTrE	Bruker MetaboBASE Personal Library 2.0
			x	553	9-OxoOTrE	Bruker MetaboBASE Personal Library 2.0
			x	534	13-Epi-12-oxo Phytodienoic Acid	Bruker MetaboBASE Personal Library 2.0
			x	534	12-OPDA	Bruker MetaboBASE Personal Library 2.0
944	11.07	2827105	x	767	Dihydroactinidiolide	Bruker MetaboBASE Personal Library 2.0
946	11.08	80913	x	417	Megastigmatrienone	Bruker MetaboBASE Personal Library 2.0
957	11.22	156538	x	231	Sequiterpene Lactone 326	Bruker MetaboBASE Personal Library 2.0
962	11.34	33252	x	599	13(S)-HpODE	Bruker MetaboBASE Personal Library 2.0
			x	528	(.plusminus.)13-HpODE	Bruker MetaboBASE Personal Library 2.0
972	11.49	17291	x	256	Tetrahydrocortisone	Bruker MetaboBASE Personal Library 2.0
973	11.49	43927	x	502	9(S)-HpOTrE	Bruker MetaboBASE Personal Library 2.0
			x	518	13(S)-HpOTrE	Bruker MetaboBASE Personal Library 2.0
976	11.55	2827105	x	672	Dihydroactinidiolide	Bruker MetaboBASE Personal Library 2.0
978	11.57	118477	x	276	(.plusminus.)Abscisic Acid	Bruker MetaboBASE Personal Library 2.0
982	11.60	29178	x	443	Confertifoline	Bruker MetaboBASE Personal Library 2.0
984	11.63	18408	x	550	Megastigmatrienone	Bruker MetaboBASE Personal Library 2.0
999	11.89	46971	x	684	Traumatic Acid	Bruker MetaboBASE Personal Library 2.0
1000	11.90	10416	x	479	Isoalantolactone	Bruker MetaboBASE Personal Library 2.0
			x	420	Alantolactone	Bruker MetaboBASE Personal Library 2.0
1008	12.00	638180	x	779	Dihydroactinidiolide	Bruker MetaboBASE Personal Library 2.0
1018	12.15	95332	x	734	Alpha-curcumene	Bruker MetaboBASE Personal Library 2.0

Compound Spectrum List Report

#	RT [min]	I	MS/MS	Purity'	Compound Name	Library
1033	12.33	19556	x	498	Megastigmatrienone	Bruker MetaboBASE Personal Library 2.0
1037	12.39	43128	x	501	2,3-Dinor Prostaglandin E1	Bruker MetaboBASE Personal Library 2.0
1038	12.40	63035	x	673	Dihydroactinidiolide	Bruker MetaboBASE Personal Library 2.0
1040	12.42	95332	x	749	Alpha-curcumene	Bruker MetaboBASE Personal Library 2.0
1045	12.51	19556	x	492	Megastigmatrienone	Bruker MetaboBASE Personal Library 2.0
1047	12.52	16047	x	467	RETUSIN 7-METHYL ETHER	Bruker MetaboBASE Personal Library 2.0
			x	467	7-HYDROXY-8,4'-DIMETHOXYISOFLAVONE	Bruker MetaboBASE Personal Library 2.0
1076	12.80	37727	x	632	Dihydroactinidiolide	Bruker MetaboBASE Personal Library 2.0
1078	12.82	11622	x	847	Isorhamnetin	Bruker MetaboBASE Personal Library 2.0
1080	12.84	9024	x	591	9(S)-HpOTrE	Bruker MetaboBASE Personal Library 2.0
1101	13.08	9709	x	374	9(S)-HpOTrE	Bruker MetaboBASE Personal Library 2.0
1107	13.15	17719	x	551	Megastigmatrienone	Bruker MetaboBASE Personal Library 2.0
1109	13.22	9709	x	487	9(S)-HpOTrE	Bruker MetaboBASE Personal Library 2.0
			x	423	13(S)-HpOTrE	Bruker MetaboBASE Personal Library 2.0
1114	13.27	24209	x	636	Dihydroactinidiolide	Bruker MetaboBASE Personal Library 2.0
1126	13.39	8033	x	834	Wogonin	Bruker MetaboBASE Personal Library 2.0
1131	13.50	12796	x	474	Megastigmatrienone	Bruker MetaboBASE Personal Library 2.0
1135	13.54	55694	x	534	Dihydroactinidiolide	Bruker MetaboBASE Personal Library 2.0
1150	13.76	4579	x	751	Isonicotinic acid	Bruker MetaboBASE Personal Library 2.0
1152	13.78	74740	x	470	Dihydroactinidiolide	Bruker MetaboBASE Personal Library 2.0
1172	14.09	5970	x	473	1-Monopalmitin	Bruker MetaboBASE Personal Library 2.0
1175	14.11	74740	x	368	Dihydroactinidiolide	Bruker MetaboBASE Personal Library 2.0
1194	14.42	5362	x	373	11.beta.-Hydroxyandrost-4-ene-3,17-dione	Bruker MetaboBASE Personal Library 2.0
1205	14.54	54075	x	342	Dihydroactinidiolide	Bruker MetaboBASE Personal Library 2.0
1208	14.57	10971	x	697	13(S)-HOTrE	Bruker MetaboBASE Personal Library 2.0
			x	684	9(S)-HOTrE	Bruker MetaboBASE Personal Library 2.0
			x	488	9,12-Octadecadiynoic Acid	Bruker MetaboBASE Personal Library 2.0
			x	447	Stearidonic Acid	Bruker MetaboBASE Personal Library 2.0
1211	14.59	18476	x	972	PC(16:0/0:0)[U] / PC(16:0/0:0)[rac]	Bruker MetaboBASE Personal Library 2.0
1214	14.64	6702	x	970	(R)-Ar-Turmerone	Bruker MetaboBASE Personal Library 2.0
1230	14.85	5862	x	338	Androsterone	Bruker MetaboBASE Personal Library 2.0
			x	328	Etiocholanolone	Bruker MetaboBASE Personal Library 2.0
			x	322	Epiandrosterone	Bruker MetaboBASE Personal Library 2.0
1251	15.17	3842	x	434	Niacin (Nicotinic acid)	Bruker MetaboBASE Personal Library 2.0
1279	15.56	5939	x	329	Dihydroactinidiolide	Bruker MetaboBASE Personal Library 2.0
1283	15.60	3721	x	258	Niacin (Nicotinic acid)	Bruker MetaboBASE Personal Library 2.0
			x	406	Isonicotinic acid	Bruker MetaboBASE Personal Library 2.0
1284	15.61	6174	x	614	(.plusminus.)10-HDoHE	Bruker MetaboBASE Personal Library 2.0
			x	636	(.plusminus.)7-HDoHE	Bruker MetaboBASE Personal Library 2.0
			x	618	(.plusminus.)11-HDoHE	Bruker MetaboBASE Personal Library 2.0
			x	585	(.plusminus.)14-HDoHE	Bruker MetaboBASE Personal Library 2.0
			x	578	(.plusminus.)13-HDoHE	Bruker MetaboBASE Personal Library 2.0
			x	562	(.plusminus.)20-HDoHE	Bruker MetaboBASE Personal Library 2.0
			x	555	(.plusminus.)8-HDoHE	Bruker MetaboBASE Personal Library 2.0
			x	554	17(R)-HDoHE	Bruker MetaboBASE Personal Library 2.0
			x	534	(.plusminus.)17-HDoHE	Bruker MetaboBASE Personal Library 2.0
			x	541	(.plusminus.)16-HDoHE	Bruker MetaboBASE Personal Library 2.0
			x	527	(.plusminus.)4-HDoHE	Bruker MetaboBASE Personal Library 2.0
			x	537	17(S)-HDoHE	Bruker MetaboBASE Personal Library 2.0
1347	16.59	8053	x	658	DEHYDRO (11,12)URSOLIC ACID LACTONE	Bruker MetaboBASE Personal Library 2.0
1378	17.18	30507	x	541	9(S)-HODE	Bruker MetaboBASE Personal Library 2.0
			x	567	13(R)-HODE	Bruker MetaboBASE Personal Library 2.0
			x	554	13(S)-HODE	Bruker MetaboBASE Personal Library 2.0
1386	17.37	3803	x	281	1-Monopalmitin	Bruker MetaboBASE Personal Library 2.0
1410	17.80	11483	x	698	DEHYDRO (11,12)URSOLIC ACID LACTONE	Bruker MetaboBASE Personal Library 2.0
1415	17.99	6689	x	592	Linoelaidic Acid	Bruker MetaboBASE Personal Library 2.0
			x	585	Linoleic acid	Bruker MetaboBASE Personal Library 2.0
			x	535	10E,12Z-Octadecadienoic acid	Bruker MetaboBASE Personal Library 2.0
1441	18.52	45160	x	966	Bis(2-ethylhexyl) phthalate	Bruker MetaboBASE Personal Library 2.0
1457	18.73	3297	x	740	N-Methyl-a-aminoisobutyric acid	Bruker MetaboBASE Personal Library 2.0
			x	589	L-Valine	Bruker MetaboBASE Personal Library 2.0
1465	18.81	45160	x	968	Bis(2-ethylhexyl) phthalate	Bruker MetaboBASE Personal Library 2.0
1495	19.25	15414	x	966	Bis(2-ethylhexyl) phthalate	Bruker MetaboBASE Personal Library 2.0
1540	20.02	53789	x	548	13E-Docosenamide	Bruker MetaboBASE Personal Library 2.0

Compound Spectrum List Report

#	RT [min]	I	MS/MS	Purity'	Compound Name	Library
			x	506	13Z-Docosenamide	Bruker MetaboBASE Personal Library 2.0
1652	21.70	8605	x	494	Guanine	Bruker MetaboBASE Personal Library 2.0
1673	21.99	50849	x	226	Dihydroactinidiolide	Bruker MetaboBASE Personal Library 2.0
1691	22.26	53243	x	597	Dihydroactinidiolide	Bruker MetaboBASE Personal Library 2.0
1695	22.31	53243	x	630	Dihydroactinidiolide	Bruker MetaboBASE Personal Library 2.0
1710	22.51	6142	x	102	Thujone	Bruker MetaboBASE Personal Library 2.0
1717	22.64	53243	x	603	Dihydroactinidiolide	Bruker MetaboBASE Personal Library 2.0
1724	22.77	8994	x	727	Isonicotinic acid	Bruker MetaboBASE Personal Library 2.0
1743	23.05	44104	x	411	Dihydroactinidiolide	Bruker MetaboBASE Personal Library 2.0
1747	23.12	8967	x	278	Megastigmatrienone	Bruker MetaboBASE Personal Library 2.0
1750	23.16	8722	x	660	Isonicotinic acid	Bruker MetaboBASE Personal Library 2.0
			x	430	Niacin (Nicotinic acid)	Bruker MetaboBASE Personal Library 2.0
1760	23.32	8722	x	690	Isonicotinic acid	Bruker MetaboBASE Personal Library 2.0
1767	23.40	5451	x	243	Limonene-1,2-epoxide	Bruker MetaboBASE Personal Library 2.0
			x	215	Dihydrocarvone	Bruker MetaboBASE Personal Library 2.0
1783	23.63	7712	x	282	Megastigmatrienone	Bruker MetaboBASE Personal Library 2.0
1784	23.63	8722	x	754	Isonicotinic acid	Bruker MetaboBASE Personal Library 2.0
1806	23.93	16374	x	510	1,2,3-Trihydroxybenzene	Bruker MetaboBASE Personal Library 2.0
1814	24.02	7607	x	329	Megastigmatrienone	Bruker MetaboBASE Personal Library 2.0
1848	24.50	6241	x	310	Megastigmatrienone	Bruker MetaboBASE Personal Library 2.0
1856	24.61	4268	x	293	Limonene-1,2-epoxide	Bruker MetaboBASE Personal Library 2.0
1894	25.12	6296	x	674	Isonicotinic acid	Bruker MetaboBASE Personal Library 2.0
1936	25.66	12833	x	216	Carboxyibuprofen	Bruker MetaboBASE Personal Library 2.0
1969	26.02	6116	x	600	Isonicotinic acid	Bruker MetaboBASE Personal Library 2.0
1989	26.31	5962	x	756	Isonicotinic acid	Bruker MetaboBASE Personal Library 2.0
2008	26.57	16795	x	647	1,2,3-Trihydroxybenzene	Bruker MetaboBASE Personal Library 2.0
2013	26.63	5962	x	764	Isonicotinic acid	Bruker MetaboBASE Personal Library 2.0
2035	26.91	5962	x	565	Isonicotinic acid	Bruker MetaboBASE Personal Library 2.0
2076	27.46	5762	x	463	Isonicotinic acid	Bruker MetaboBASE Personal Library 2.0
2167	28.71	5695	x	701	Isonicotinic acid	Bruker MetaboBASE Personal Library 2.0
2175	28.80	13450	x	589	1,2,3-Trihydroxybenzene	Bruker MetaboBASE Personal Library 2.0
2193	29.09	5695	x	622	Isonicotinic acid	Bruker MetaboBASE Personal Library 2.0
2216	29.43	12142	x	468	1,2,3-Trihydroxybenzene	Bruker MetaboBASE Personal Library 2.0
2222	29.51	4952	x	648	Isonicotinic acid	Bruker MetaboBASE Personal Library 2.0
2249	29.82	5682	x	726	Isonicotinic acid	Bruker MetaboBASE Personal Library 2.0

Cmpd 28, AutoMSn(104.1073), 0.71 min

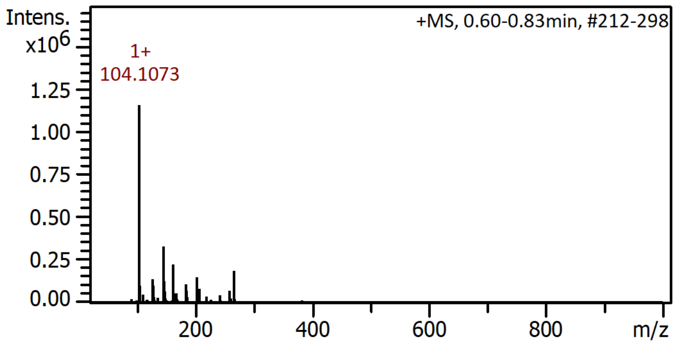

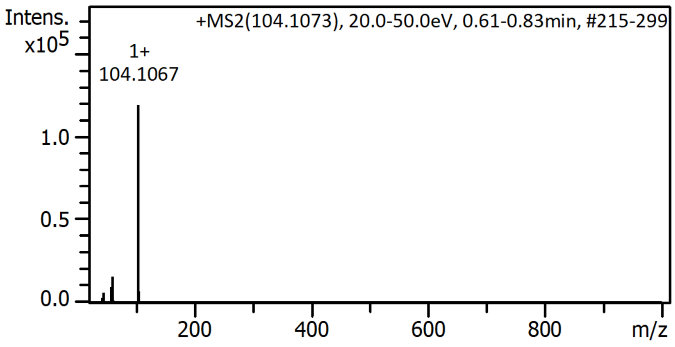

#	m/z	Res.	S/N	I	I %	FWHM
1	104.1073	8435	192270.5	1153623	100.0	0.0123
2	105.1104	8766	16654.6	99928	8.7	0.0120
3	127.0241	9567	22601.7	135610	11.8	0.0133
4	128.0209	8861	16654.1	99925	8.7	0.0144
5	145.0350	9584	55505.9	333035	28.9	0.0151
6	146.0336	8428	20763.5	124581	10.8	0.0173
7	163.0466	8295	37898.9	227394	19.7	0.0197
8	185.0663	10302	17614.6	105688	9.2	0.0180
9	203.0528	10369	24512.3	147074	12.7	0.0196
10	266.1601	11434	31081.4	186488	16.2	0.0233

#	m/z	Res.	S/N	I	I %	FWHM
1	42.0344	5345	683.5	2734	2.3	0.0079
2	44.0507	4087	1161.0	4644	3.9	0.0108
3	45.0345	4641	1446.7	5787	4.9	0.0097
4	58.0660	4473	2406.4	9626	8.1	0.0130
5	59.0732	6993	962.6	3851	3.2	0.0084
6	60.0824	2924	3932.5	15730	13.2	0.0205
7	61.0843	7285	341.8	1367	1.2	0.0084
8	88.0757	8607	196.4	786	0.7	0.0102
9	104.1067	7852	29721.2	118885	100.0	0.0133
10	105.1098	9118	1670.3	6681	5.6	0.0115

Compound Spectrum List Report

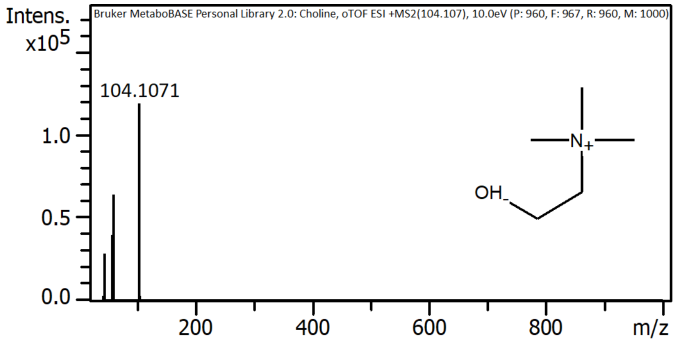

#	m/z	Res.	S/N	I	I %	FWHM
1	42.0345	2827	4.0	2853	2.4	0.0149
2	44.0498	2963	22.5	16049	13.5	0.0149
3	45.0339	3029	40.3	28770	24.2	0.0149
4	45.0556	3031	10.0	7133	6.0	0.0149
5	45.0571	3031	5.0	3567	3.0	0.0149
6	58.0665	3906	55.8	39826	33.5	0.0149
7	59.0737	3973	6.5	4637	3.9	0.0149
8	60.0818	4041	90.0	64198	54.1	0.0149
9	104.1071	7003	166.5	118766	100.0	0.0149
10	104.2005	7009	3.8	2734	2.3	0.0149

Cmpd 43, AutoMSn(258.1093), 0.86 min

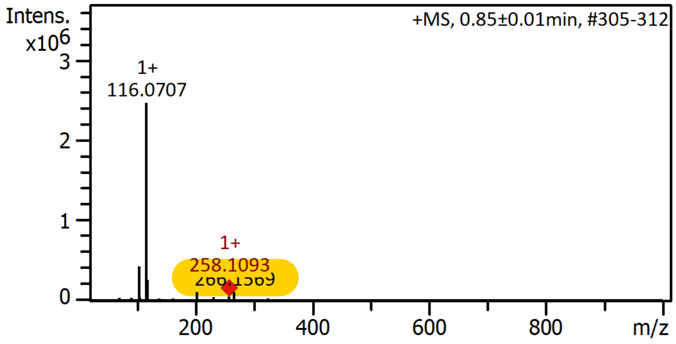

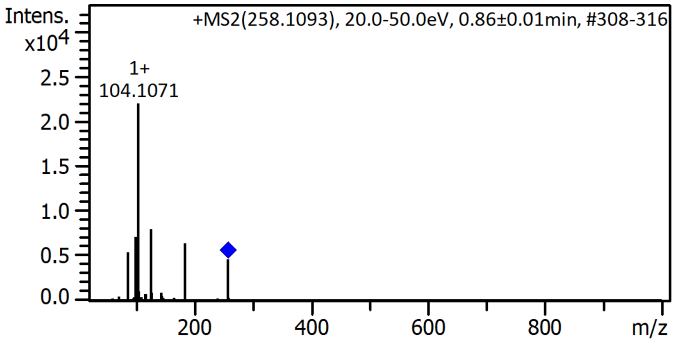

#	m/z	Res.	S/N	I	I %	FWHM
1	70.0658	4369	2085.6	37541	1.5	0.0160
2	104.0699	9277	2875.2	51754	2.1	0.0112
3	104.1066	8323	23466.7	422401	17.2	0.0125
4	116.0707	8175	136788.3	2462189	100.0	0.0142
5	117.0737	9060	12039.9	216718	8.8	0.0129
6	118.0856	8977	14171.0	255079	10.4	0.0132
7	203.0519	10917	5831.1	104959	4.3	0.0186
8	231.1347	10853	2432.2	43779	1.8	0.0213
9	258.1093	11531	2740.1	49323	2.0	0.0224
10	266.1569	11467	6147.0	110646	4.5	0.0232

#	m/z	Res.	S/N	I	I %	FWHM
1	86.0968	7786	228.1	5474	24.9	0.0111
2	98.9840	9660	299.6	7191	32.7	0.0102
3	104.1071	9080	916.0	21983	100.0	0.0115
4	105.1103	11257	43.4	1041	4.7	0.0093
5	116.0683	10511	31.1	747	3.4	0.0110
6	124.9998	10694	333.4	8001	36.4	0.0117
7	126.0644	11592	38.2	917	4.2	0.0109
8	143.0108	9871	36.2	869	4.0	0.0145
9	184.0711	10954	269.8	6476	29.5	0.0168
10	258.1126	11596	194.4	4666	21.2	0.0223

Compound Spectrum List Report

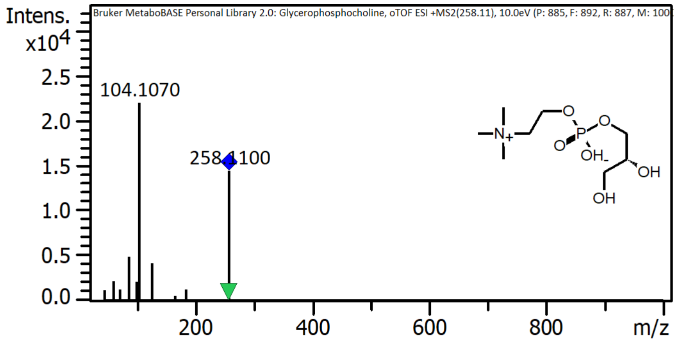

#	m/z	Res.	S/N	I	I %	FWHM
1	45.0334	4240	2.3	1187	5.4	0.0106
2	60.0814	5657	4.3	2198	10.0	0.0106
3	71.0735	6692	2.4	1231	5.6	0.0106
4	86.0970	8107	9.7	4902	22.3	0.0106
5	98.9840	9320	4.2	2110	9.6	0.0106
6	104.1070	9802	43.4	21961	100.0	0.0106
7	124.9995	11770	8.4	4243	19.3	0.0106
8	166.0624	15636	1.0	506	2.3	0.0106
9	184.0727	17332	2.4	1209	5.5	0.0106
10	258.1100	24303	28.5	14421	65.7	0.0106

Cmpd 51, AutoMSn(325.1136), 0.93 min

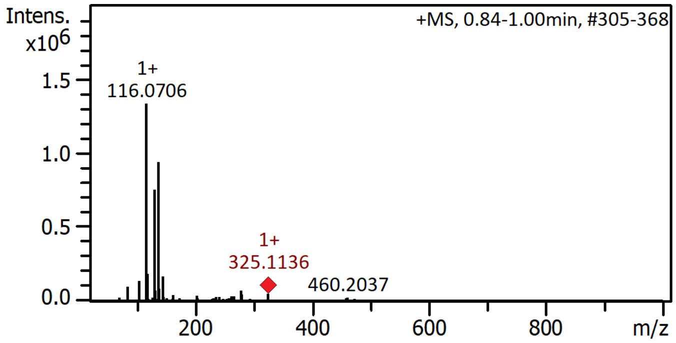

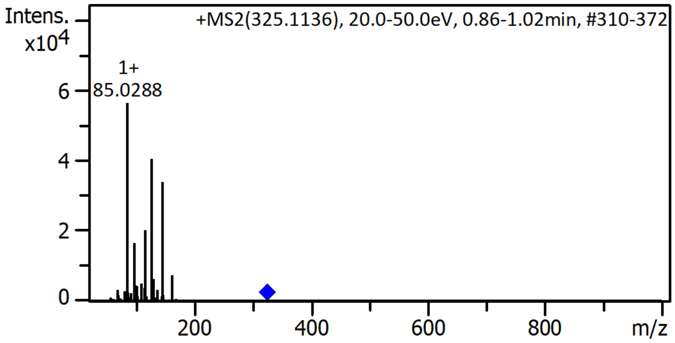

#	m/z	Res.	S/N	I	I %	FWHM
1	84.0812	8334	16498.9	98993	7.4	0.0101
2	104.1065	8311	22832.9	136997	10.3	0.0125
3	116.0706	8375	222261.9	1333572	100.0	0.0139
4	117.0735	9186	18997.8	113987	8.5	0.0127
5	118.0859	9059	30329.1	181975	13.6	0.0130
6	130.0866	9337	126175.4	757052	56.8	0.0139
7	136.0621	9551	156602.6	939616	70.5	0.0142
8	137.0644	8953	14290.6	85744	6.4	0.0153
9	144.1024	10273	27794.1	166765	12.5	0.0140
10	278.1248	11994	11774.6	70648	5.3	0.0232

#	m/z	Res.	S/N	I	I %	FWHM
1	85.0288	8513	7191.8	56336	100.0	0.0100
2	97.0285	9052	2137.5	16744	29.7	0.0107
3	99.0442	9874	569.6	4462	7.9	0.0100
4	101.0235	10301	546.8	4284	7.6	0.0098
5	109.0289	9751	631.8	4949	8.8	0.0112
6	116.0710	9907	2581.2	20219	35.9	0.0117
7	127.0392	10047	5181.0	40585	72.0	0.0126
8	130.0865	9874	801.3	6277	11.1	0.0132
9	145.0496	10419	4339.6	33994	60.3	0.0139
10	163.0602	11122	930.7	7290	12.9	0.0147

Compound Spectrum List Report

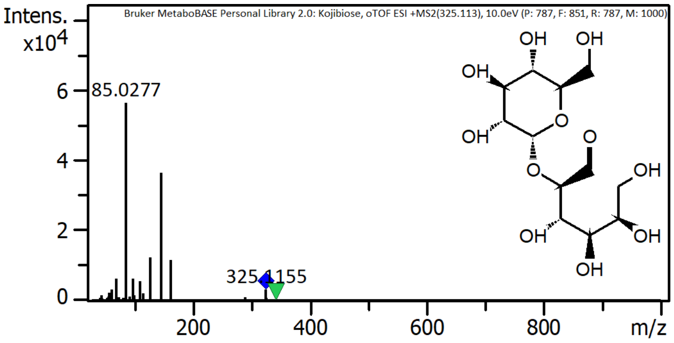

#	m/z	Res.	S/N	I	I %	FWHM
1	61.0283	6863	11.4	3211	5.7	0.0089
2	69.0341	7763	22.0	6197	11.0	0.0089
3	85.0277	9562	199.8	56279	100.0	0.0089
4	97.0275	10911	22.4	6310	11.2	0.0089
5	109.0273	12261	19.6	5521	9.8	0.0089
6	127.0378	14286	17.0	4789	8.5	0.0089
7	127.0384	14286	44.0	12394	22.0	0.0089
8	145.0484	16312	129.4	36449	64.8	0.0089
9	163.0595	18337	41.4	11662	20.7	0.0089
10	325.1155	36561	11.2	3155	5.6	0.0089

Cmpd 53, AutoMSn(136.0623), 0.94 min

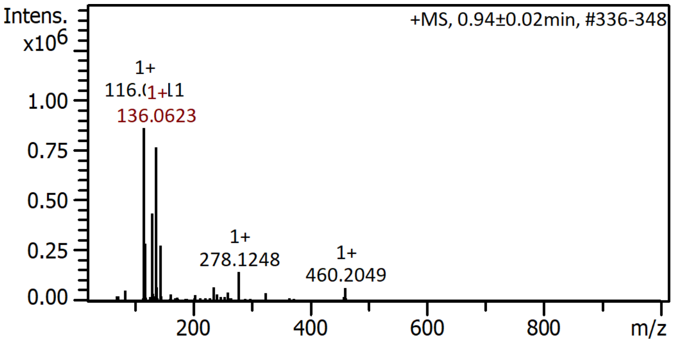

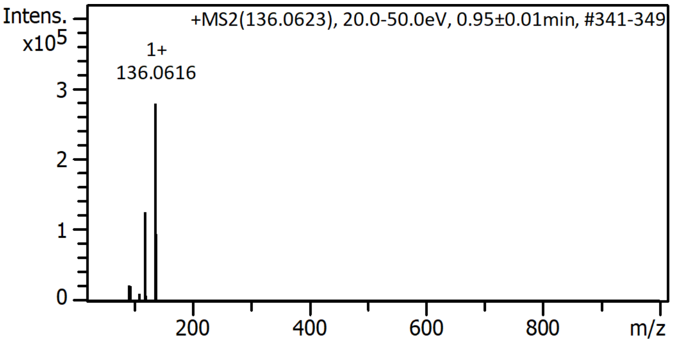

#	m/z	Res.	S/N	I	I %	FWHM
1	116.0711	8875	81994.4	983932	100.0	0.0131
2	117.0742	9431	6936.6	83239	8.5	0.0124
3	118.0869	8886	24074.9	288899	29.4	0.0133
4	130.0867	9373	36522.2	438267	44.5	0.0139
5	136.0623	9591	63739.4	764873	77.7	0.0142
6	137.0646	9560	5636.6	67640	6.9	0.0143
7	144.1027	10039	23285.6	279427	28.4	0.0144
8	236.1506	10923	5760.9	69131	7.0	0.0216
9	278.1248	12100	11970.0	143640	14.6	0.0230
10	460.2049	12307	5433.1	65198	6.6	0.0374

#	m/z	Res.	S/N	I	I %	FWHM
1	65.0131	8675	329.7	2638	0.9	0.0075
2	92.0244	9001	2797.9	22383	8.0	0.0102
3	94.0401	9277	2648.8	21190	7.6	0.0101
4	109.0505	9435	1266.1	10129	3.6	0.0116
5	112.0500	10321	334.1	2673	1.0	0.0109
6	119.0348	9523	15788.7	126310	45.3	0.0125
7	120.0379	9878	943.8	7550	2.7	0.0122
8	136.0616	8657	34850.7	278806	100.0	0.0157
9	137.0464	9486	11897.8	95182	34.1	0.0144
10	138.0489	9187	776.0	6208	2.2	0.0150

Compound Spectrum List Report

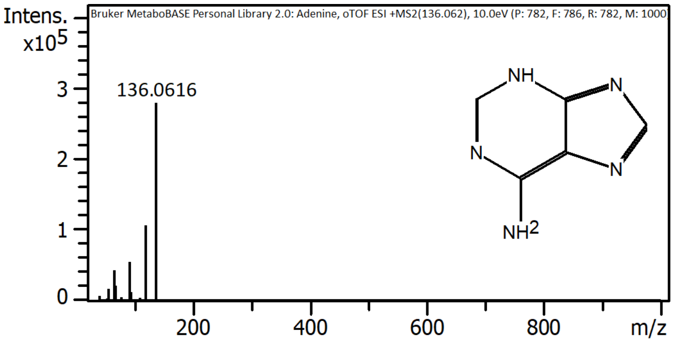

#	m/z	Res.	S/N	I	I %	FWHM
1	40.0197	2661	3.3	6413	2.3	0.0150
2	55.0298	3660	8.7	17007	6.1	0.0150
3	65.0136	4324	22.0	42936	15.4	0.0150
4	67.0297	4458	10.7	20910	7.5	0.0150
5	77.0137	5122	2.7	5297	1.9	0.0150
6	92.0241	6120	28.6	55761	20.0	0.0150
7	94.0385	6254	2.0	3903	1.4	0.0150
8	94.0408	6254	6.1	11989	4.3	0.0150
9	119.0349	7916	54.7	106783	38.3	0.0150
10	136.0616	9049	142.7	278527	100.0	0.0150

Cmpd 72, AutoMSn(152.0566), 1.14 min

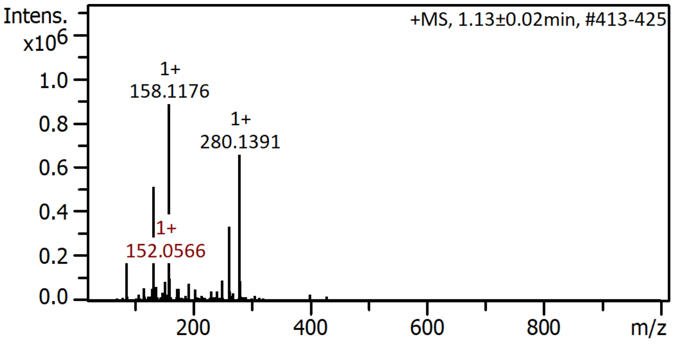

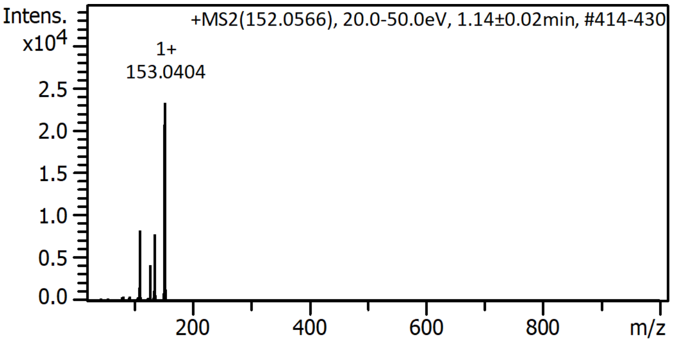

#	m/z	Res.	S/N	I	I %	FWHM
1	86.0965	7934	21795.8	261550	29.5	0.0109
2	132.1018	9250	42738.1	512857	57.8	0.0143
3	152.0566	10760	7059.6	84715	9.6	0.0141
4	158.1176	9801	73888.6	886663	100.0	0.0161
5	159.1206	10715	8186.3	98236	11.1	0.0148
6	193.0707	11506	6378.2	76539	8.6	0.0168
7	250.1291	11590	7662.4	91949	10.4	0.0216
8	262.1284	11842	28014.3	336172	37.9	0.0221
9	280.1391	11133	54860.6	658327	74.2	0.0252
10	281.1426	11878	7228.1	86737	9.8	0.0237

#	m/z	Res.	S/N	I	I %	FWHM
1	109.0494	8587	96.2	1507	6.5	0.0127
2	110.0343	9466	530.7	8314	35.8	0.0116
3	128.0445	9570	263.7	4132	17.8	0.0134
4	134.0498	11632	74.2	1163	5.0	0.0115
5	135.0308	10563	498.6	7811	33.6	0.0128
6	136.0303	7491	42.8	671	2.9	0.0182
7	151.0854	7732	56.7	888	3.8	0.0195
8	152.0566	10337	1320.9	20694	89.1	0.0147
9	153.0404	10433	1482.6	23228	100.0	0.0147
10	154.0383	8374	84.2	1319	5.7	0.0184

Compound Spectrum List Report

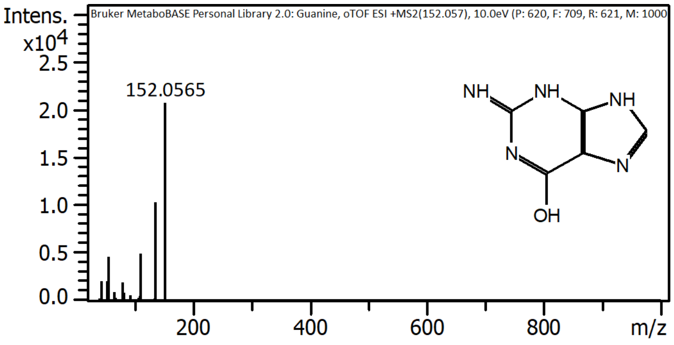

#	m/z	Res.	S/N	I	I %	FWHM
1	43.0292	2857	8.3	2049	9.9	0.0151
2	53.0137	3520	8.3	2049	9.9	0.0151
3	55.0294	3654	18.8	4656	22.5	0.0151
4	65.0142	4316	3.7	931	4.5	0.0151
5	80.0248	5313	7.8	1925	9.3	0.0151
6	82.0406	5447	3.4	848	4.1	0.0151
7	93.0083	6175	2.3	579	2.8	0.0151
8	110.0350	7305	20.1	4987	24.1	0.0151
9	135.0299	8965	41.6	10326	49.9	0.0151
10	152.0565	10095	83.3	20673	100.0	0.0151

Cmpd 73, AutoMSn(132.1020), 1.15 min

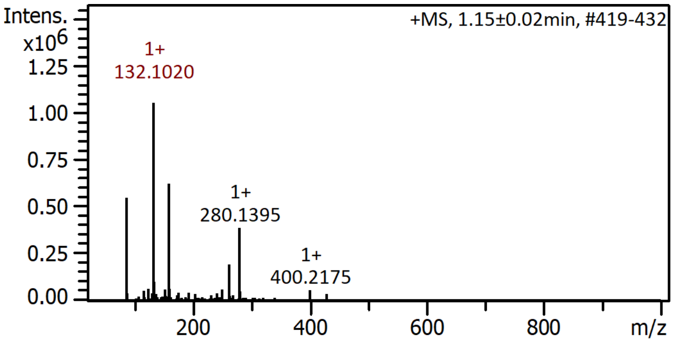

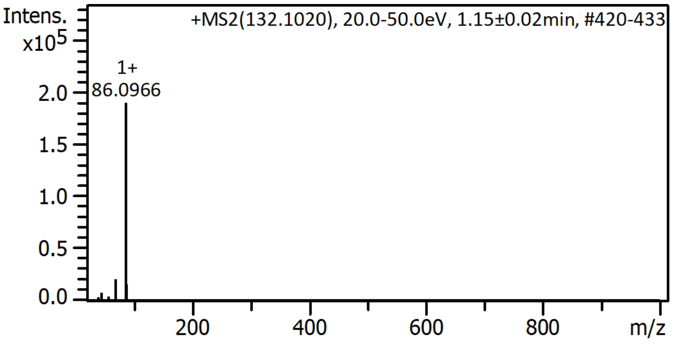

#	m/z	Res.	S/N	I	I %	FWHM
1	86.0967	7963	45615.4	547385	52.1	0.0108
2	123.0492	8357	5182.1	62185	5.9	0.0147
3	132.1020	9230	87532.1	1050386	100.0	0.0143
4	133.1053	9957	8264.7	99177	9.4	0.0134
5	152.0568	10621	4841.7	58100	5.5	0.0143
6	158.1177	9873	51896.6	622759	59.3	0.0160
7	159.1211	10742	5380.2	64562	6.1	0.0148
8	250.1294	11795	4888.9	58666	5.6	0.0212
9	262.1288	12024	15947.7	191373	18.2	0.0218
10	280.1395	11380	32505.6	390067	37.1	0.0246

#	m/z	Res.	S/N	I	I %	FWHM
1	39.0243	5520	449.4	3595	1.9	0.0071
2	44.0503	4256	937.5	7500	4.0	0.0104
3	45.0341	6827	164.3	1315	0.7	0.0066
4	56.0494	6828	502.7	4022	2.1	0.0082
5	57.0573	7158	445.0	3560	1.9	0.0080
6	58.0654	5837	205.4	1643	0.9	0.0099
7	69.0703	5112	2566.5	20532	10.8	0.0135
8	70.0718	5906	217.3	1738	0.9	0.0119
9	86.0966	6303	23669.9	189359	100.0	0.0137
10	87.0997	8284	1940.1	15521	8.2	0.0105

Compound Spectrum List Report

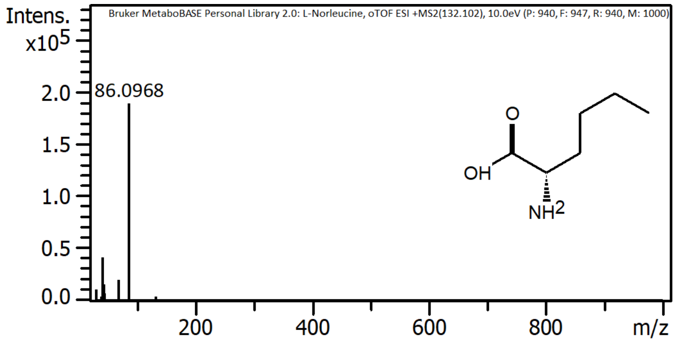

#	m/z	Res.	S/N	I	I %	FWHM
1	30.0337	2185	11.4	10793	5.7	0.0137
2	39.0234	2839	4.2	3977	2.1	0.0137
3	41.0394	2986	44.0	41659	22.0	0.0137
4	43.0550	3132	16.6	15717	8.3	0.0137
5	44.0505	3205	7.2	6817	3.6	0.0137
6	69.0704	5025	21.0	19883	10.5	0.0137
7	86.0968	6264	199.8	189170	100.0	0.0137
8	86.1813	6270	4.6	4355	2.3	0.0137
9	86.2093	6272	1.4	1326	0.7	0.0137
10	132.1024	9611	4.0	3787	2.0	0.0137

Cmpd 78, AutoMSn(123.0549), 1.19 min

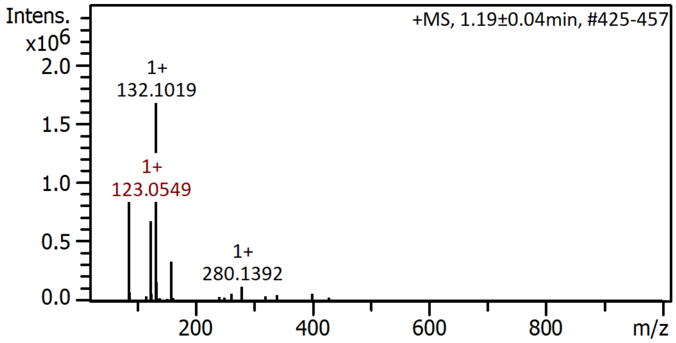

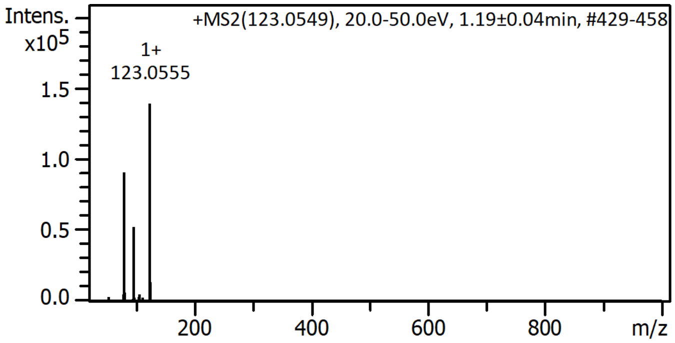

#	m/z	Res.	S/N	I	I %	FWHM
1	86.0966	7911	151056.1	906337	54.2	0.0109
2	87.0999	8429	12163.0	72978	4.4	0.0103
3	123.0549	8918	113332.6	679996	40.7	0.0138
4	124.0580	9172	10351.1	62107	3.7	0.0135
5	132.1019	9213	278710.9	1672266	100.0	0.0143
6	133.1054	9813	26477.9	158868	9.5	0.0136
7	158.1177	10180	56323.8	337943	20.2	0.0155
8	262.1287	11686	10069.0	60414	3.6	0.0224
9	280.1392	11610	20055.3	120332	7.2	0.0241
10	400.2182	13092	10681.1	64087	3.8	0.0306

#	m/z	Res.	S/N	I	I %	FWHM
1	53.0389	7276	752.5	3010	2.2	0.0073
2	78.0343	8347	1165.5	4662	3.4	0.0093
3	80.0499	6522	22727.4	90910	65.4	0.0123
4	81.0527	8129	1546.4	6186	4.4	0.0100
5	96.0445	8497	13075.5	52302	37.6	0.0113
6	105.0431	8558	771.2	3085	2.2	0.0123
7	106.0295	9932	1184.3	4737	3.4	0.0107
8	111.0314	10813	644.7	2579	1.9	0.0103
9	123.0555	8530	34756.5	139026	100.0	0.0144
10	124.0564	8302	3293.4	13174	9.5	0.0149

Compound Spectrum List Report

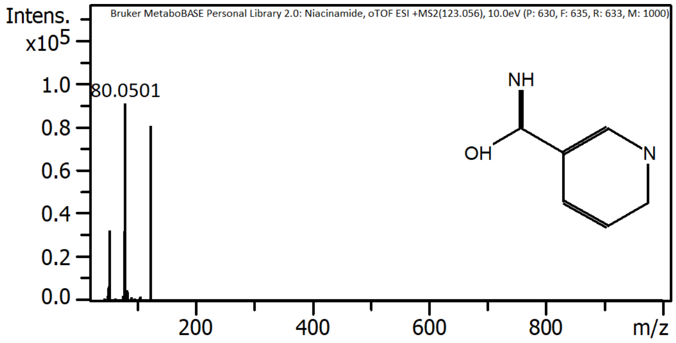

#	m/z	Res.	S/N	I	I %	FWHM
1	51.0235	3291	12.6	5727	6.3	0.0155
2	52.0301	3356	14.6	6636	7.3	0.0155
3	53.0387	3421	56.8	25818	28.4	0.0155
4	53.0390	3421	71.6	32546	35.8	0.0155
5	75.9833	4901	4.8	2182	2.4	0.0155
6	78.0343	5033	70.6	32091	35.3	0.0155
7	80.0501	5163	199.8	90819	100.0	0.0155
8	82.9898	5353	10.6	4818	5.3	0.0155
9	83.4941	5385	8.6	3909	4.3	0.0155
10	123.0553	7937	177.0	80455	88.6	0.0155

Cmpd 86, AutoMSn(163.0605), 1.31 min

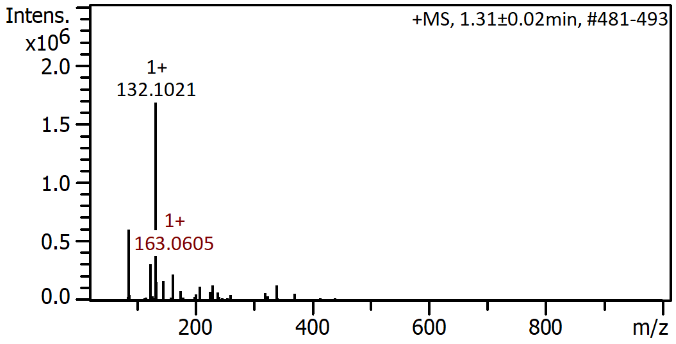

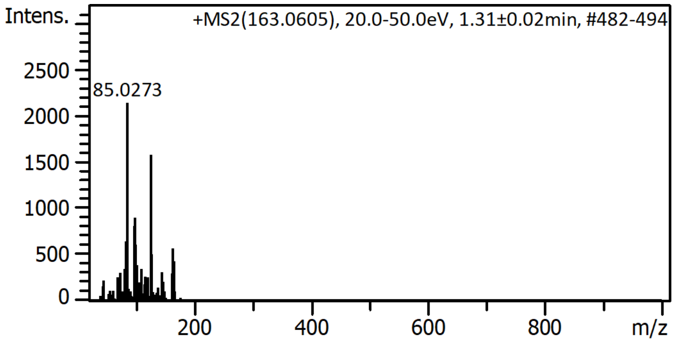

#	m/z	Res.	S/N	I	I %	FWHM
1	86.0968	7717	50434.8	605218	36.0	0.0112
2	123.0555	9322	25642.2	307706	18.3	0.0132
3	132.1021	9103	140019.6	1680236	100.0	0.0145
4	133.1052	9632	12739.4	152873	9.1	0.0138
5	145.0500	10057	13876.6	166519	9.9	0.0144
6	163.0605	10329	18345.9	220151	13.1	0.0158
7	176.0927	10421	6344.6	76135	4.5	0.0169
8	209.1028	11025	9733.0	116796	7.0	0.0190
9	231.0847	11027	10855.4	130265	7.8	0.0210
10	340.1969	12134	10620.8	127450	7.6	0.0280

#	m/z	Res.	S/N	I	I %	FWHM
1	83.0133	8821	45.0	645	30.2	0.0094
2	85.0273	9061	149.1	2137	100.0	0.0094
3	97.0288	8481	56.6	811	38.0	0.0114
4	98.0591	8987	62.8	900	42.1	0.0109
5	99.0445	9541	42.5	609	28.5	0.0104
6	101.0220	11525	26.7	383	17.9	0.0088
7	126.0543	8753	110.0	1576	73.8	0.0144
8	127.0416	12562	35.5	509	23.8	0.0101
9	164.0717	10392	39.5	567	26.5	0.0158
10	166.0714	9632	30.1	431	20.2	0.0172

Compound Spectrum List Report

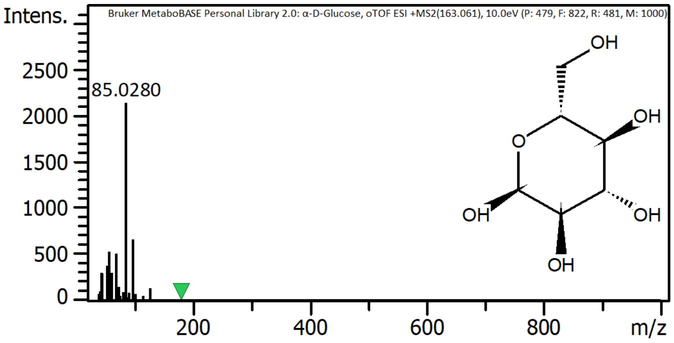

#	m/z	Res.	S/N	I	I %	FWHM
1	43.0176	5327	7.9	303	14.2	0.0081
2	45.0314	5576	7.7	295	13.8	0.0081
3	45.0335	5576	5.9	229	10.7	0.0081
4	53.0381	6567	9.8	376	17.6	0.0081
5	55.0191	6813	5.8	224	10.5	0.0081
6	57.0327	7062	13.8	532	24.9	0.0081
7	61.0282	7557	7.8	301	14.1	0.0081
8	69.0315	8548	13.4	517	24.2	0.0081
9	85.0280	10528	55.5	2135	100.0	0.0081
10	97.0275	12014	17.3	665	31.1	0.0081

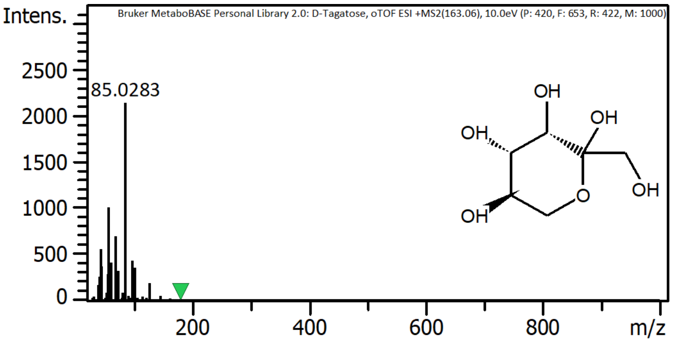

#	m/z	Res.	S/N	I	I %	FWHM
1	43.0182	5327	52.6	562	26.3	0.0081
2	45.0328	5576	34.4	368	17.2	0.0081
3	45.0340	5576	31.2	333	15.6	0.0081
4	57.0336	7062	94.6	1011	47.3	0.0081
5	61.0291	7557	38.8	415	19.4	0.0081
6	69.0336	8548	65.8	703	32.9	0.0081
7	73.0279	9043	29.8	318	14.9	0.0081
8	85.0283	10528	199.8	2135	100.0	0.0081
9	97.0277	12014	41.2	440	20.6	0.0081
10	101.0229	12509	33.0	353	16.5	0.0081

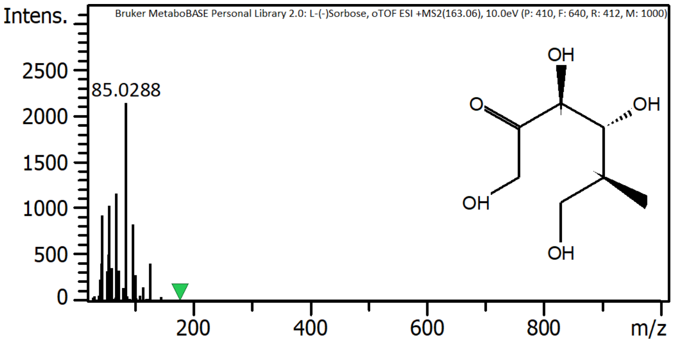

#	m/z	Res.	S/N	I	I %	FWHM
1	43.0175	5327	38.6	412	19.3	0.0081
2	43.0190	5327	36.8	393	18.4	0.0081
3	45.0342	5576	86.8	927	43.4	0.0081
4	55.0178	6812	47.2	504	23.6	0.0081
5	57.0340	7062	96.4	1030	48.2	0.0081
6	69.0334	8548	55.2	590	27.6	0.0081
7	69.0339	8548	108.6	1160	54.4	0.0081
8	85.0288	10529	199.8	2135	100.0	0.0081
9	97.0284	12014	78.2	835	39.1	0.0081
10	127.0381	15730	38.2	408	19.1	0.0081

Cmpd 90, AutoMSn(325.1152), 1.36 min

Compound Spectrum List Report

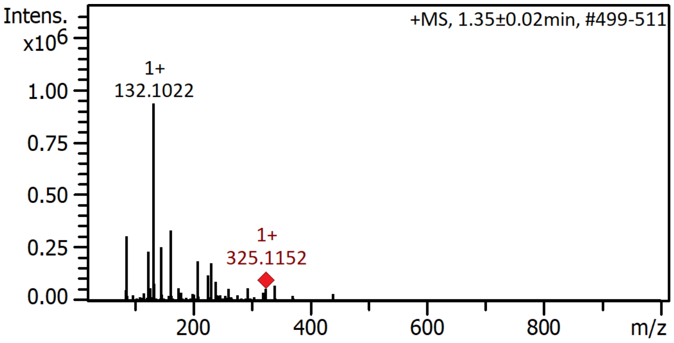

#	m/z	Res.	S/N	I	I %	FWHM
1	86.0969	7821	25519.2	306231	32.8	0.0110
2	123.0555	9505	19547.2	234567	25.1	0.0129
3	132.1022	9233	77915.1	934981	100.0	0.0143
4	133.1054	10151	6662.9	79955	8.6	0.0131
5	145.0501	9899	21419.6	257035	27.5	0.0147
6	163.0605	10826	27848.3	334180	35.7	0.0151
7	209.1028	11343	15702.9	188435	20.2	0.0184
8	226.1294	11594	10042.7	120513	12.9	0.0195
9	231.0848	11837	14924.7	179096	19.2	0.0195
10	239.1137	12043	7449.0	89388	9.6	0.0199

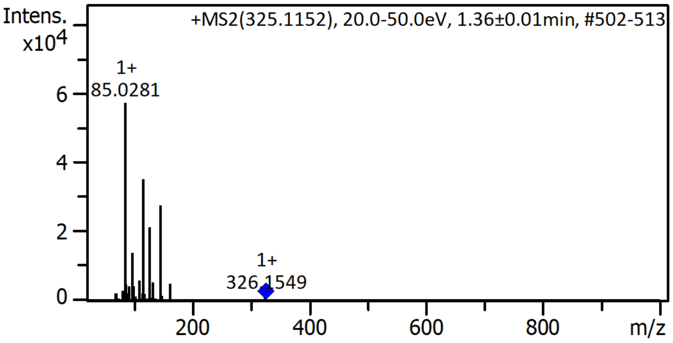

#	m/z	Res.	S/N	I	I %	FWHM
1	85.0281	8582	3574.0	57184	100.0	0.0099
2	86.0963	9090	299.9	4798	8.4	0.0095
3	97.0276	9262	875.0	14000	24.5	0.0105
4	99.0439	8130	259.5	4153	7.3	0.0122
5	109.0279	10291	368.1	5889	10.3	0.0106
6	116.0703	10491	2202.4	35239	61.6	0.0111
7	127.0384	10218	1333.9	21342	37.3	0.0124
8	132.1014	11855	333.6	5338	9.3	0.0111
9	145.0486	10341	1721.7	27548	48.2	0.0140
10	163.0596	10695	303.8	4861	8.5	0.0152

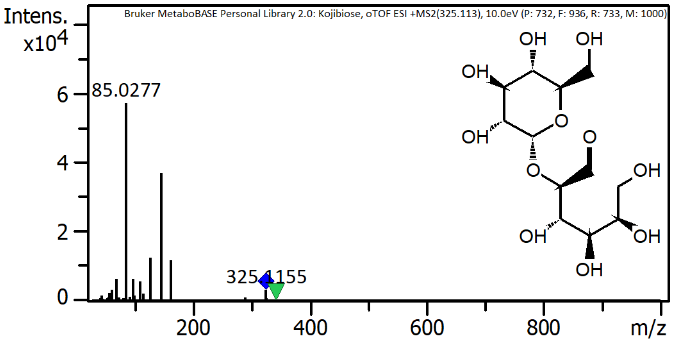

#	m/z	Res.	S/N	I	I %	FWHM
1	61.0283	6846	11.4	3260	5.7	0.0089
2	69.0341	7744	22.0	6290	11.0	0.0089
3	85.0277	9538	199.8	57127	100.0	0.0089
4	97.0275	10884	22.4	6405	11.2	0.0089
5	109.0273	12230	19.6	5604	9.8	0.0089
6	127.0378	14250	17.0	4861	8.5	0.0089
7	127.0384	14250	44.0	12581	22.0	0.0089
8	145.0484	16271	129.4	36998	64.8	0.0089
9	163.0595	18291	41.4	11837	20.7	0.0089
10	325.1155	36470	11.2	3202	5.6	0.0089

Cmpd 102, AutoMSn(132.1021), 1.47 min

Compound Spectrum List Report

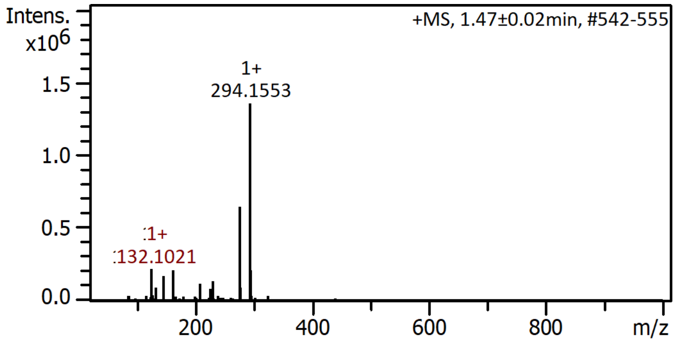

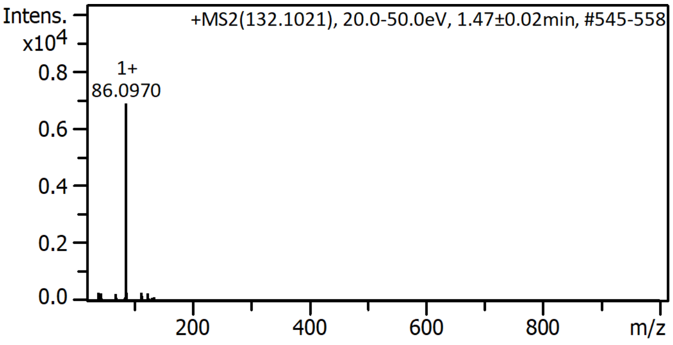

#	m/z	Res.	S/N	I	I %	FWHM
1	124.0398	9765	18158.1	217898	16.1	0.0127
2	132.1021	10016	7653.6	91843	6.8	0.0132
3	145.0500	10265	14208.1	170497	12.6	0.0141
4	163.0605	10903	17404.2	208850	15.4	0.0150
5	209.1024	11649	9740.0	116880	8.6	0.0179
6	231.0846	11702	11136.3	133635	9.9	0.0197
7	276.1446	11467	54127.2	649526	47.9	0.0241
8	277.1481	12065	7387.6	88651	6.5	0.0230
9	294.1553	11157	112885.2	1354622	100.0	0.0264
10	295.1588	11883	17463.8	209566	15.5	0.0248

#	m/z	Res.	S/N	I	I %	FWHM
1	39.0204	6942	18.0	282	4.1	0.0056
2	43.0553	9058	9.6	151	2.2	0.0048
3	43.0669	8875	10.8	170	2.5	0.0049
4	44.0479	8216	16.7	261	3.8	0.0054
5	69.0705	11012	15.6	244	3.5	0.0063
6	86.0970	8047	438.5	6869	100.0	0.0107
7	87.0975	10733	17.4	273	4.0	0.0081
8	112.0171	10815	17.3	271	3.9	0.0104
9	113.0236	9274	10.3	162	2.4	0.0122
10	123.0777	8945	16.1	252	3.7	0.0138

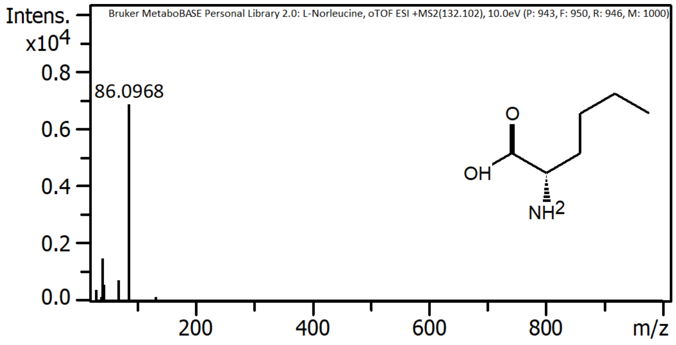

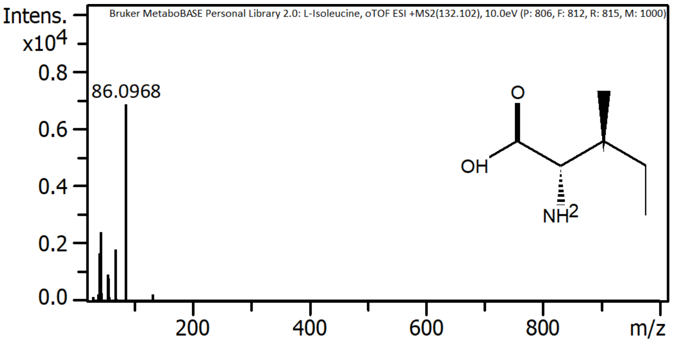

#	m/z	Res.	S/N	I	I %	FWHM
1	30.0337	2887	11.4	392	5.7	0.0104
2	39.0234	3751	4.2	144	2.1	0.0104
3	41.0394	3945	44.0	1511	22.0	0.0104
4	43.0550	4139	16.6	570	8.3	0.0104
5	44.0505	4234	7.2	247	3.6	0.0104
6	69.0704	6640	21.0	721	10.5	0.0104
7	86.0968	8276	199.8	6862	100.0	0.0104
8	86.1813	8284	4.6	158	2.3	0.0104
9	86.2093	8287	1.4	48	0.7	0.0104
10	132.1024	12699	4.0	137	2.0	0.0104

#	m/z	Res.	S/N	I	I %	FWHM
1	39.0232	3751	4.7	227	3.3	0.0104
2	41.0390	3945	34.9	1676	24.4	0.0104
3	42.0348	4041	4.9	234	3.4	0.0104
4	44.0496	4234	50.1	2411	35.1	0.0104
5	45.0336	4329	5.7	275	4.0	0.0104
6	56.0501	5388	19.1	920	13.4	0.0104
7	57.0581	5485	16.3	783	11.4	0.0104
8	69.0704	6640	38.0	1827	26.6	0.0104
9	86.0968	8276	142.7	6862	100.0	0.0104
10	132.1001	12698	5.0	240	3.5	0.0104

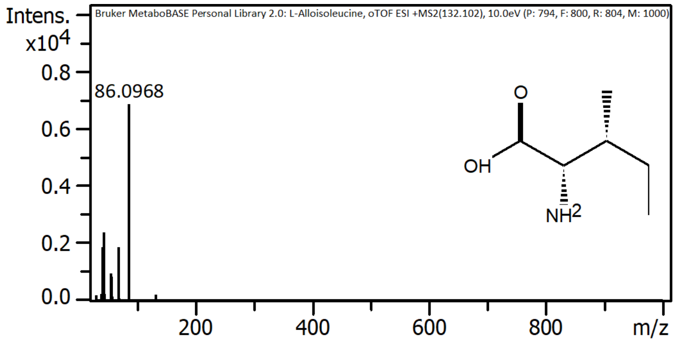

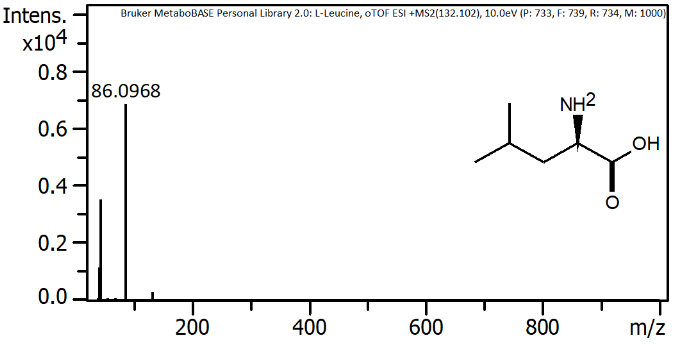

Compound Spectrum List Report

#	m/z	Res.	S/N	I	I %	FWHM
1	39.0241	3751	7.0	240	3.5	0.0104
2	41.0394	3945	55.0	1889	27.5	0.0104
3	42.0347	4041	5.8	199	2.9	0.0104
4	44.0504	4234	69.8	2397	34.9	0.0104
5	45.0341	4329	7.0	240	3.5	0.0104
6	56.0503	5388	27.4	941	13.7	0.0104
7	57.0580	5485	24.4	838	12.2	0.0104
8	69.0705	6640	54.6	1875	27.3	0.0104
9	86.0968	8276	199.8	6862	100.0	0.0104
10	132.1026	12699	6.0	206	3.0	0.0104

#	m/z	Res.	S/N	I	I %	FWHM
1	39.0227	3751	1.3	82	1.2	0.0104
2	41.0388	3945	18.6	1147	16.7	0.0104
3	43.0547	4139	37.2	2301	33.5	0.0104
4	44.0498	4234	57.3	3545	51.7	0.0104
5	55.0172	5289	1.2	76	1.1	0.0104
6	55.0542	5292	1.0	62	0.9	0.0104
7	69.0699	6639	1.3	82	1.2	0.0104
8	86.0968	8276	111.0	6862	100.0	0.0104
9	132.1014	12699	4.9	302	4.4	0.0104

Cmpd 104, AutoMSn(124.0398), 1.54 min

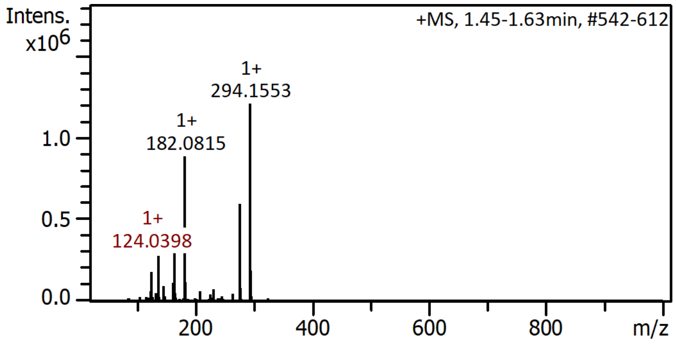

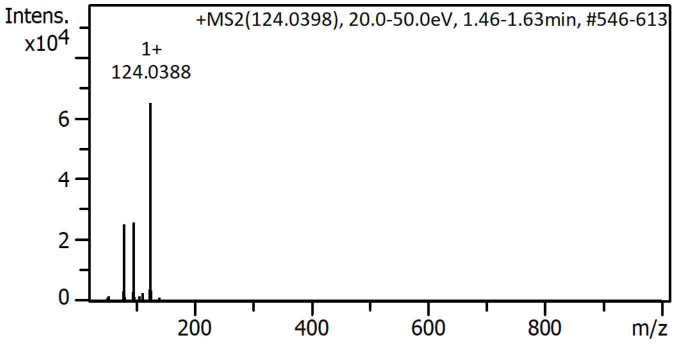

#	m/z	Res.	S/N	I	I %	FWHM
1	124.0398	9666	29904.9	179429	14.9	0.0128
2	136.0760	9486	46504.6	279028	23.1	0.0143
3	145.0499	10291	15667.3	94004	7.8	0.0141
4	163.0605	10892	18855.4	113133	9.4	0.0150
5	165.0550	9941	73771.7	442630	36.7	0.0166
6	182.0815	9902	147675.0	886050	73.4	0.0184
7	183.0849	10883	19450.2	116701	9.7	0.0168
8	276.1447	11390	99306.4	595838	49.4	0.0242
9	294.1553	11120	201193.2	1207159	100.0	0.0265
10	295.1589	11663	30705.3	184232	15.3	0.0253

#	m/z	Res.	S/N	I	I %	FWHM
1	53.0393	4934	218.6	1640	2.5	0.0107
2	78.0343	7381	435.0	3263	5.0	0.0106
3	80.0493	8044	3366.7	25251	38.9	0.0100
4	95.0489	9343	416.2	3121	4.8	0.0102
5	96.0441	8854	3446.1	25846	39.8	0.0108
6	106.0734	9995	202.3	1517	2.3	0.0106
7	111.0314	8897	358.2	2687	4.1	0.0125
8	123.0552	10040	512.9	3847	5.9	0.0123
9	124.0388	9932	8661.6	64962	100.0	0.0125
10	125.0422	9343	469.0	3518	5.4	0.0134

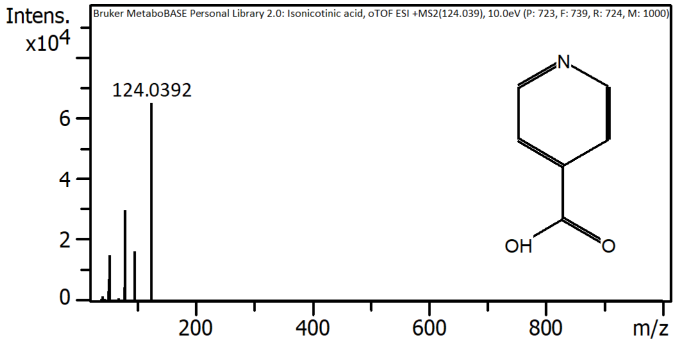

#	m/z	Res.	S/N	I	I %	FWHM
1	41.0392	3061	4.8	1559	2.4	0.0134
2	51.0224	3806	10.2	3313	5.1	0.0134
3	52.0307	3881	22.4	7276	11.2	0.0134
4	53.0390	3956	46.8	15201	23.4	0.0134
5	78.0343	5820	13.8	4482	6.9	0.0134
6	78.0345	5820	6.8	2209	3.4	0.0134
7	79.0421	5896	24.2	7860	12.1	0.0134
8	80.0500	5971	92.0	29883	46.0	0.0134
9	96.0449	7164	50.6	16435	25.3	0.0134
10	124.0392	9252	199.8	64897	100.0	0.0134

Compound Spectrum List Report

Cmpd 108, AutoMSn(182.0815), 1.56 min

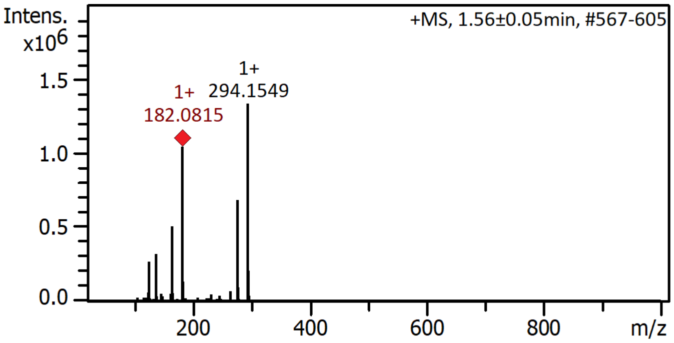

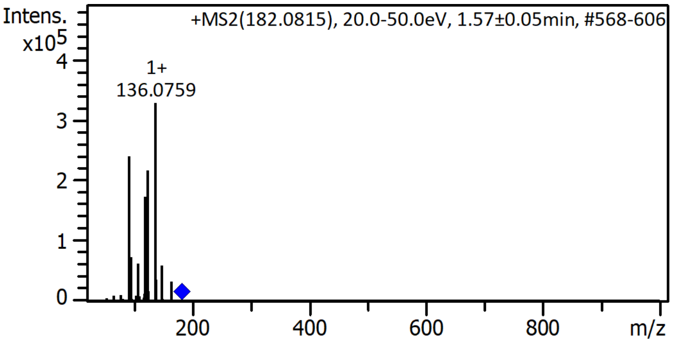

#	m/z	Res.	S/N	I	I %	FWHM
1	124.0397	9300	44787.3	268724	20.1	0.0133
2	136.0757	9517	54063.5	324381	24.3	0.0143
3	165.0549	10022	84697.4	508185	38.0	0.0165
4	182.0815	9884	174459.8	1046759	78.3	0.0184
5	183.0846	10706	22125.1	132751	9.9	0.0171
6	264.1446	11906	11228.2	67369	5.0	0.0222
7	276.1446	11056	114114.3	684686	51.2	0.0250
8	277.1482	11870	15496.4	92979	7.0	0.0233
9	294.1549	10890	222830.9	1336985	100.0	0.0270
10	295.1587	11437	34358.1	206149	15.4	0.0258

#	m/z	Res.	S/N	I	I %	FWHM
1	91.0544	7936	60084.2	240337	73.2	0.0115
2	92.0578	8582	4347.4	17390	5.3	0.0107
3	95.0494	8644	18400.4	73602	22.4	0.0110
4	107.0491	9386	15845.3	63381	19.3	0.0114
5	119.0495	8516	43399.0	173596	52.9	0.0140
6	123.0443	8348	54322.3	217289	66.2	0.0147
7	136.0759	8391	82063.8	328255	100.0	0.0162
8	137.0785	9669	8937.1	35749	10.9	0.0142
9	147.0443	10413	14760.6	59043	18.0	0.0141
10	165.0553	10876	8215.9	32864	10.0	0.0152

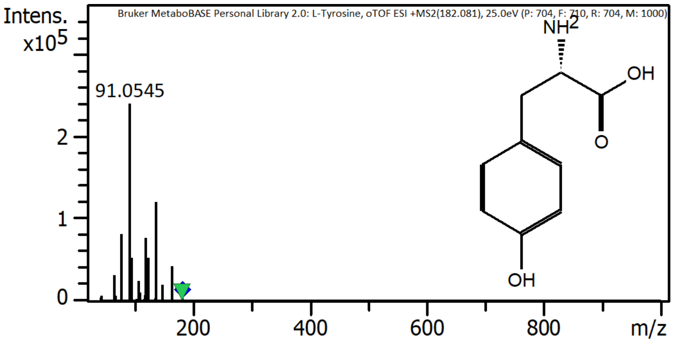

#	m/z	Res.	S/N	I	I %	FWHM
1	65.0397	4119	26.4	31724	13.2	0.0158
2	77.0392	4879	68.4	82195	34.2	0.0158
3	91.0545	5766	199.8	240096	100.0	0.0158
4	95.0485	6019	22.2	26677	11.1	0.0158
5	95.0500	6019	44.4	53355	22.2	0.0158
6	107.0481	6779	20.4	24514	10.2	0.0158
7	119.0490	7539	64.4	77388	32.2	0.0158
8	123.0439	7792	44.0	52874	22.0	0.0158
9	136.0751	8617	100.4	120649	50.3	0.0158
10	165.0546	10452	35.2	42299	17.6	0.0158

Cmpd 127, AutoMSn(86.0602), 1.78 min

Compound Spectrum List Report

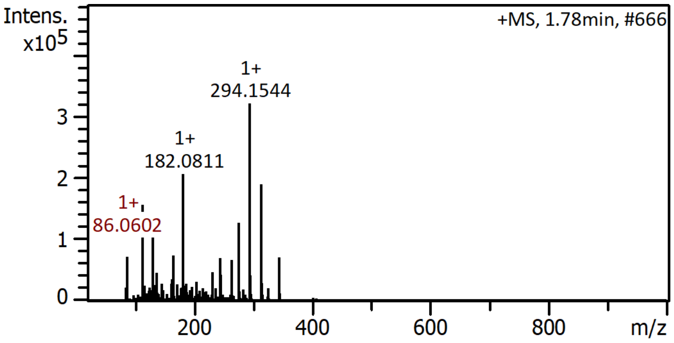

#	m/z	Res.	S/N	I	I %	FWHM
1	86.0602	8809	2005.9	72212	22.5	0.0098
2	113.0346	9729	4344.9	156416	48.6	0.0116
3	130.0499	10467	3624.9	130497	40.6	0.0124
4	165.0553	11562	2054.2	73950	23.0	0.0143
5	182.0811	11374	5751.8	207065	64.4	0.0160
6	245.0772	11633	1949.9	70198	21.8	0.0211
7	276.1441	12319	3544.8	127614	39.7	0.0224
8	294.1544	12177	8934.4	321640	100.0	0.0242
9	314.0916	12351	5265.4	189555	58.9	0.0254
10	344.1344	12254	1981.0	71315	22.2	0.0281

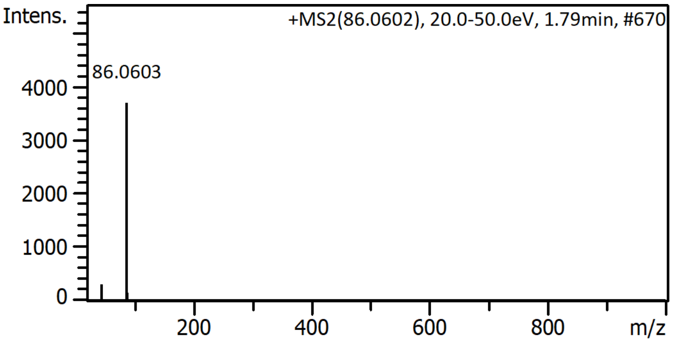

#	m/z	Res.	S/N	I	I %	FWHM
1	44.0115	4664	2.1	309	8.4	0.0094
2	86.0603	8108	25.6	3691	100.0	0.0106
3	88.0661	13015	1.0	144	3.9	0.0068

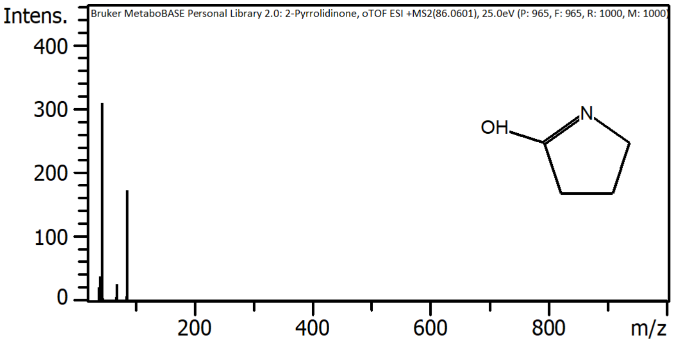

#	m/z	Res.	S/N	I	I %	FWHM
1	39.0240	3638	11.7	22	7.0	0.0107
2	41.0393	3825	20.5	38	12.3	0.0107
3	43.0206	4010	4.5	8	2.7	0.0107
4	43.0549	4013	3.7	7	2.2	0.0107
5	44.0143	4103	166.5	309	100.0	0.0107
6	44.0494	4106	12.0	22	7.2	0.0107
7	68.0498	6343	3.5	6	2.1	0.0107
8	69.0339	6435	14.2	26	8.5	0.0107
9	85.0760	7930	4.0	7	2.4	0.0107
10	86.0604	8022	93.5	173	56.2	0.0107

Cmpd 130, AutoMSn(245.0766), 1.80 min

Compound Spectrum List Report

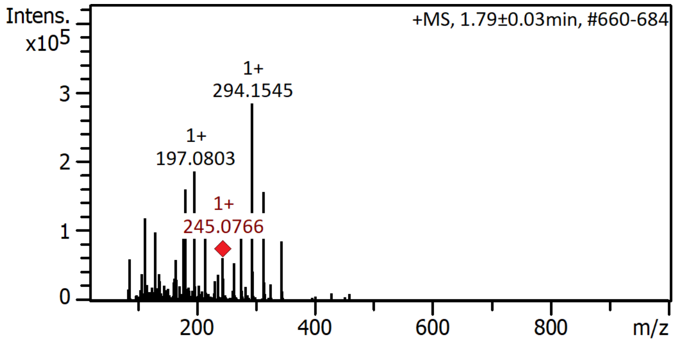

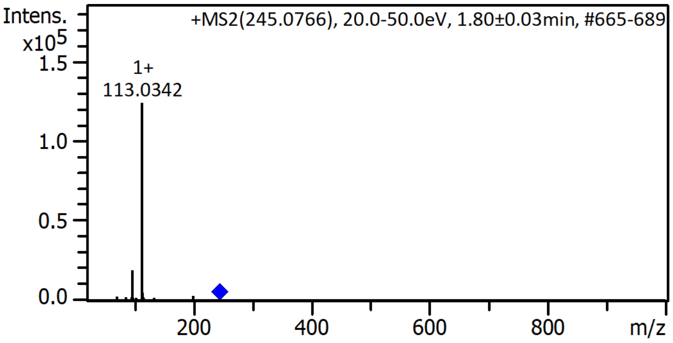

#	m/z	Res.	S/N	I	I %	FWHM
1	113.0346	9631	6618.5	119134	41.9	0.0117
2	130.0502	10078	5470.4	98467	34.6	0.0129
3	179.0695	11505	5353.8	96369	33.9	0.0156
4	182.0817	10613	8918.1	160526	56.5	0.0172
5	197.0803	10600	10340.5	186130	65.5	0.0186
6	215.0911	11379	6040.1	108723	38.3	0.0189
7	276.1443	12126	6323.8	113829	40.1	0.0228
8	294.1545	11881	15789.4	284210	100.0	0.0248
9	314.0915	12241	8688.8	156399	55.0	0.0257
10	344.1325	12211	4774.6	85942	30.2	0.0282

#	m/z	Res.	S/N	I	I %	FWHM
1	70.0289	8645	108.9	2613	2.1	0.0081
2	85.0277	10366	91.1	2186	1.8	0.0082
3	95.0250	9011	90.4	2169	1.8	0.0105
4	96.0083	8887	786.9	18885	15.3	0.0108
5	97.0287	9224	80.8	1940	1.6	0.0105
6	113.0342	9624	5158.4	123801	100.0	0.0117
7	114.0375	9720	214.6	5151	4.2	0.0117
8	115.0401	8517	87.5	2100	1.7	0.0135
9	200.1287	10932	126.1	3026	2.4	0.0183
10	246.1335	15598	71.9	1726	1.4	0.0158

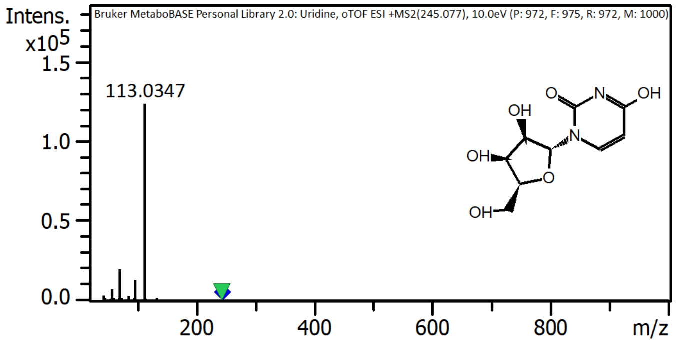

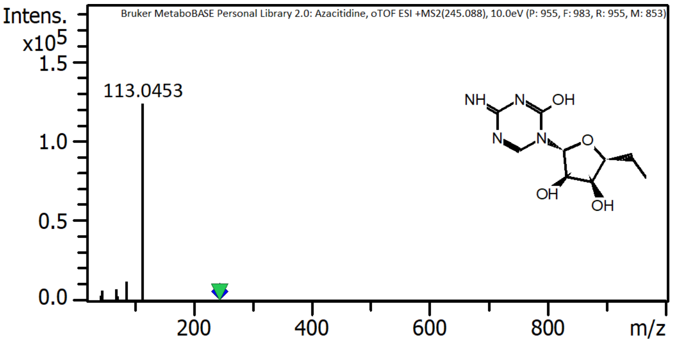

#	m/z	Res.	S/N	I	I %	FWHM
1	43.0189	3628	5.8	3590	2.9	0.0119
2	45.0339	3798	2.6	1609	1.3	0.0119
3	57.0341	4810	12.2	7552	6.1	0.0119
4	61.0296	5147	3.2	1981	1.6	0.0119
5	69.0352	5822	3.0	1857	1.5	0.0119
6	70.0295	5906	32.0	19808	16.0	0.0119
7	73.0292	6159	3.2	1981	1.6	0.0119
8	85.0287	7171	4.6	2847	2.3	0.0119
9	96.0081	8097	21.2	13123	10.6	0.0119
10	113.0347	9533	199.8	123677	100.0	0.0119

#	m/z	Res.	S/N	I	I %	FWHM
1	43.0281	3629	4.3	3219	2.6	0.0119
2	45.0451	3799	9.0	6685	5.4	0.0119
3	55.0183	4640	1.2	867	0.7	0.0119
4	57.0341	4810	1.5	1114	0.9	0.0119
5	69.0093	5820	9.8	7304	5.9	0.0119
6	71.0225	5990	4.0	2971	2.4	0.0119
7	86.0345	7256	16.7	12380	10.0	0.0119
8	113.0453	9534	166.5	123677	100.0	0.0119
9	245.0988	20671	1.0	743	0.6	0.0119

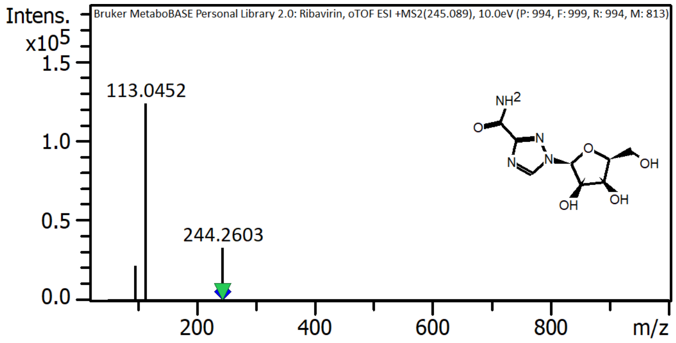

Compound Spectrum List Report

#	m/z	Res.	S/N	I	I %	FWHM
1	96.0190	8098	1.0	21789	17.6	0.0119
2	113.0452	9534	5.7	123677	100.0	0.0119
3	244.2603	20600	1.5	33302	26.9	0.0119

Cmpd 141, AutoMSn(268.1040), 1.95 min

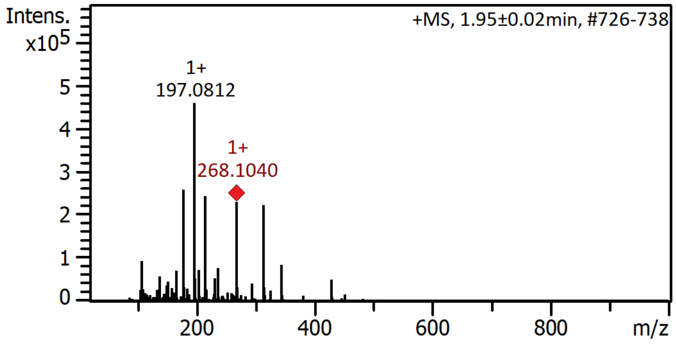

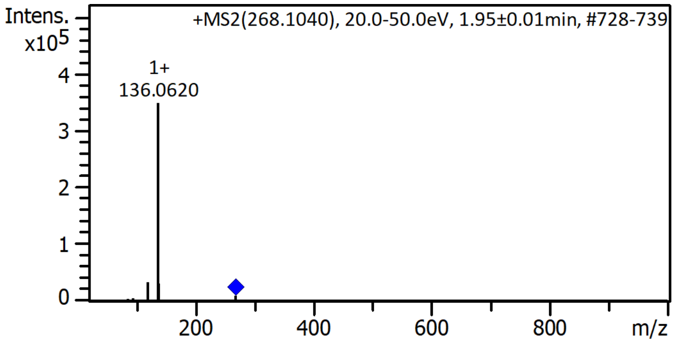

#	m/z	Res.	S/N	I	I %	FWHM
1	107.0493	9475	7855.6	94268	20.5	0.0113
2	167.0701	11029	5909.4	70913	15.4	0.0151
3	179.0705	11027	21540.2	258482	56.3	0.0162
4	197.0812	10482	38258.0	459096	100.0	0.0188
5	204.1233	11312	6039.6	72475	15.8	0.0180
6	215.0919	11337	20313.6	243763	53.1	0.0190
7	237.0743	11081	6379.2	76550	16.7	0.0214
8	268.1040	12071	19281.2	231375	50.4	0.0222
9	314.0920	12420	18678.3	224140	48.8	0.0253
10	344.1345	12513	7004.3	84052	18.3	0.0275

#	m/z	Res.	S/N	I	I %	FWHM
1	85.0284	8882	333.0	4440	1.3	0.0096
2	92.0253	8720	186.8	2490	0.7	0.0106
3	94.0400	8461	376.9	5025	1.4	0.0111
4	97.0288	8905	155.7	2076	0.6	0.0109
5	109.0507	9709	244.3	3257	0.9	0.0112
6	119.0352	10204	2502.4	33365	9.6	0.0117
7	133.0506	10815	164.7	2196	0.6	0.0123
8	136.0620	9337	26109.1	348121	100.0	0.0146
9	137.0609	7832	2318.0	30907	8.9	0.0175
10	268.1037	11638	681.7	9089	2.6	0.0230

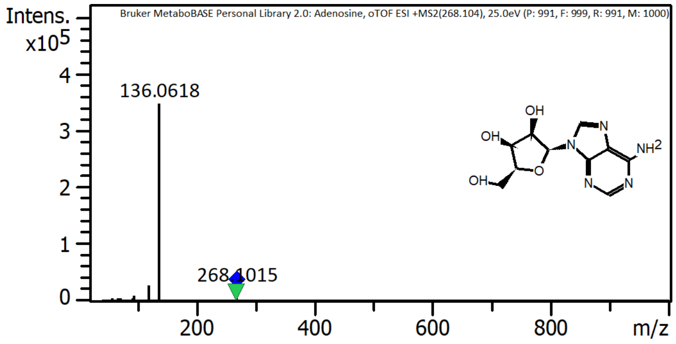

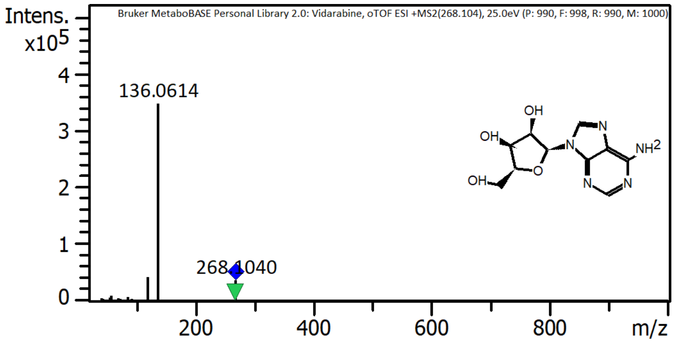

#	m/z	Res.	S/N	I	I %	FWHM
1	41.0310	2803	1.2	2089	0.6	0.0146
2	57.0346	3896	2.6	4526	1.3	0.0146
3	67.0291	4579	2.6	4526	1.3	0.0146
4	69.0336	4716	1.8	3133	0.9	0.0146
5	71.0127	4851	2.6	4526	1.3	0.0146
6	92.0221	6286	2.6	4526	1.3	0.0146
7	94.0401	6424	5.2	9051	2.6	0.0146
8	119.0367	8131	15.6	27153	7.8	0.0146
9	136.0618	9294	199.8	347773	100.0	0.0146
10	268.1015	18314	13.4	23324	6.7	0.0146

#	m/z	Res.	S/N	I	I %	FWHM
1	41.0389	2803	2.3	4874	1.4	0.0146
2	43.0182	2939	2.0	4177	1.2	0.0146
3	55.0167	3758	3.0	6266	1.8	0.0146
4	57.0333	3896	4.3	9051	2.6	0.0146
5	69.0358	4716	2.2	4526	1.3	0.0146
6	73.0308	4989	1.8	3829	1.1	0.0146
7	85.0289	5808	3.3	6962	2.0	0.0146
8	119.0357	8131	20.3	42471	12.2	0.0146
9	136.0614	9294	166.5	347773	100.0	0.0146
10	268.1040	18314	17.5	36553	10.5	0.0146

Cmpd 168, AutoMSn(86.0603), 2.21 min

Compound Spectrum List Report

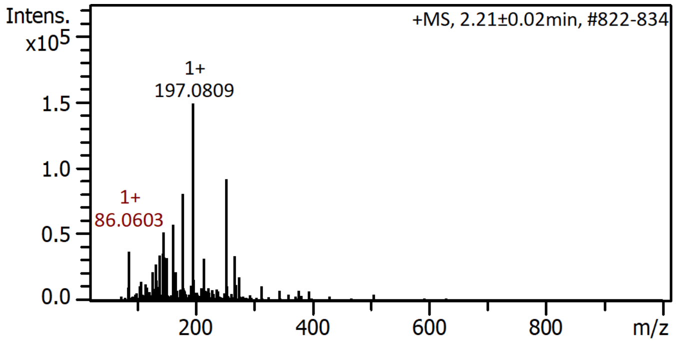

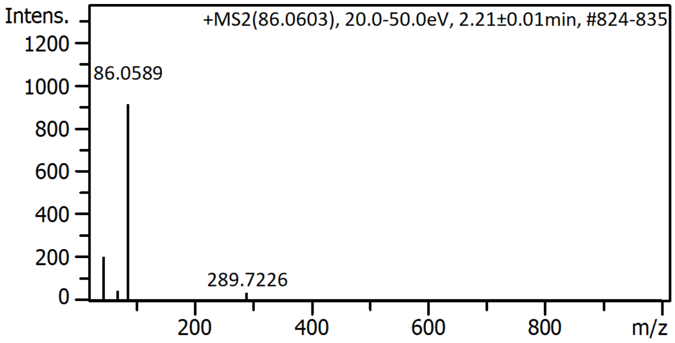

#	m/z	Res.	S/N	I	I %	FWHM
1	86.0603	8437	3132.5	37590	25.3	0.0102
2	139.0752	10140	2893.9	34727	23.3	0.0137
3	144.0478	10344	3009.0	36108	24.3	0.0139
4	145.0496	10470	5303.8	63646	42.8	0.0139
5	146.0812	10272	2756.2	33074	22.2	0.0142
6	163.0600	10782	4811.3	57735	38.8	0.0151
7	179.0703	11006	6728.9	80747	54.3	0.0163
8	197.0809	11095	12395.1	148742	100.0	0.0178
9	253.1282	11735	7671.8	92062	61.9	0.0216
10	268.1048	12335	2837.4	34048	22.9	0.0217

#	m/z	Res.	S/N	I	I %	FWHM
1	44.0133	6037	5.6	207	22.7	0.0073
2	69.0384	12067	1.2	45	4.9	0.0057
3	86.0589	9772	24.7	912	100.0	0.0088
4	289.7226	23694	1.0	37	4.1	0.0122

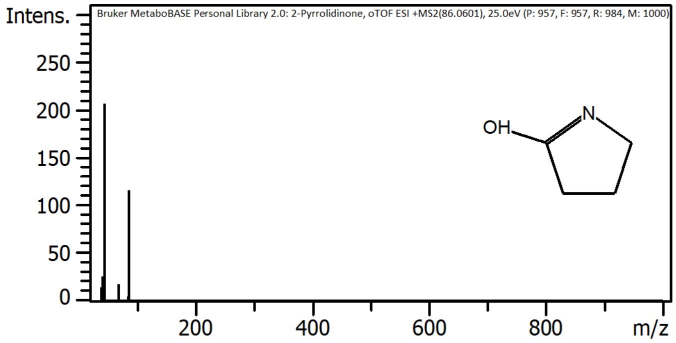

#	m/z	Res.	S/N	I	I %	FWHM
1	39.0240	4373	11.7	14	7.0	0.0089
2	41.0393	4599	20.5	25	12.3	0.0089
3	43.0206	4821	4.5	6	2.7	0.0089
4	43.0549	4825	3.7	5	2.2	0.0089
5	44.0143	4932	166.5	206	100.0	0.0089
6	44.0494	4936	12.0	15	7.2	0.0089
7	68.0498	7626	3.5	4	2.1	0.0089
8	69.0339	7736	14.2	18	8.5	0.0089
9	85.0760	9534	4.0	5	2.4	0.0089
10	86.0604	9644	93.5	116	56.2	0.0089

Cmpd 178, AutoMSn(166.0863), 2.34 min

Compound Spectrum List Report

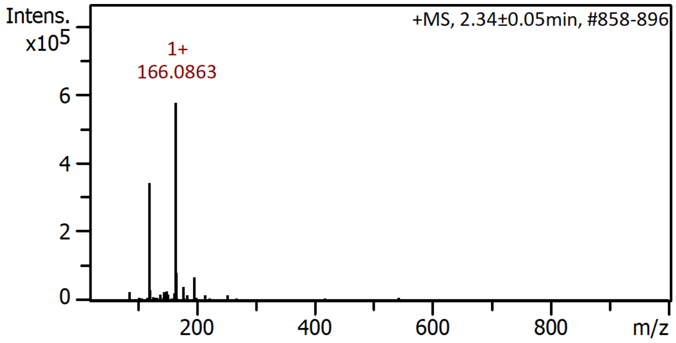

#	m/z	Res.	S/N	I	I %	FWHM
1	86.0602	8885	4258.5	25551	4.4	0.0097
2	120.0808	9396	57175.4	343052	59.6	0.0128
3	121.0838	9764	5262.3	31574	5.5	0.0124
4	145.0497	10419	4238.8	25433	4.4	0.0139
5	146.0812	11078	4327.4	25964	4.5	0.0132
6	149.0600	10232	4614.0	27684	4.8	0.0146
7	166.0863	9871	95995.8	575975	100.0	0.0168
8	167.0870	9308	13548.6	81292	14.1	0.0180
9	179.0706	11103	6614.7	39688	6.9	0.0161
10	197.0809	11442	11235.6	67413	11.7	0.0172

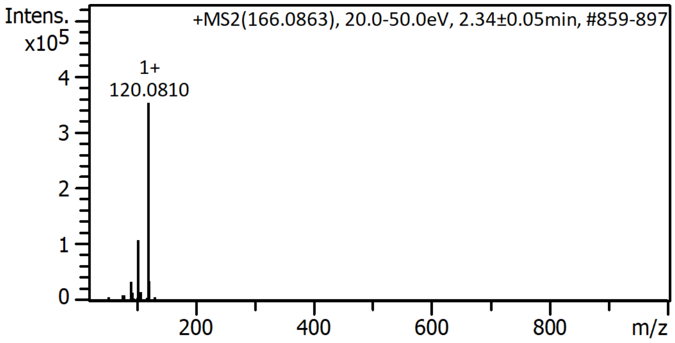

#	m/z	Res.	S/N	I	I %	FWHM
1	77.0385	7999	2037.4	9169	2.6	0.0096
2	79.0546	8054	2066.4	9299	2.6	0.0098
3	91.0543	8740	7583.0	34123	9.7	0.0104
4	93.0698	8824	3245.0	14602	4.1	0.0105
5	103.0543	9077	24252.1	109134	31.0	0.0114
6	104.0574	8891	1950.6	8778	2.5	0.0117
7	107.0492	9516	3510.8	15799	4.5	0.0112
8	120.0810	8155	78230.5	352037	100.0	0.0147
9	121.0840	9796	7810.2	35146	10.0	0.0124
10	131.0494	10119	1489.5	6703	1.9	0.0130

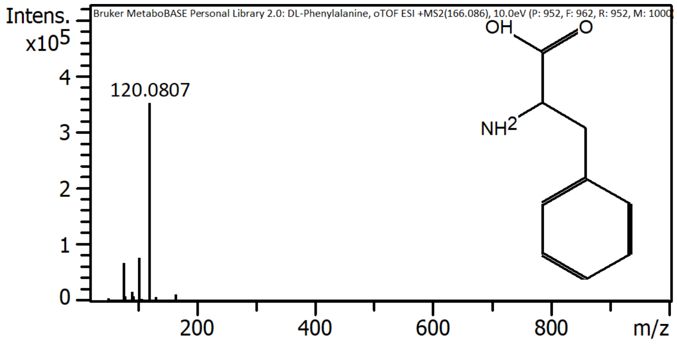

#	m/z	Res.	S/N	I	I %	FWHM
1	51.0237	3624	3.2	5633	1.6	0.0141
2	77.0394	5472	39.0	68647	19.5	0.0141
3	79.0544	5615	5.0	8801	2.5	0.0141
4	91.0550	6467	9.2	16194	4.6	0.0141
5	93.0690	6610	4.8	8449	2.4	0.0141
6	103.0544	7319	44.0	77448	22.0	0.0141
7	107.0495	7603	2.0	3520	1.0	0.0141
8	120.0807	8529	199.8	351685	100.0	0.0141
9	131.0487	9308	4.4	7745	2.2	0.0141
10	166.0879	11796	6.6	11617	3.3	0.0141

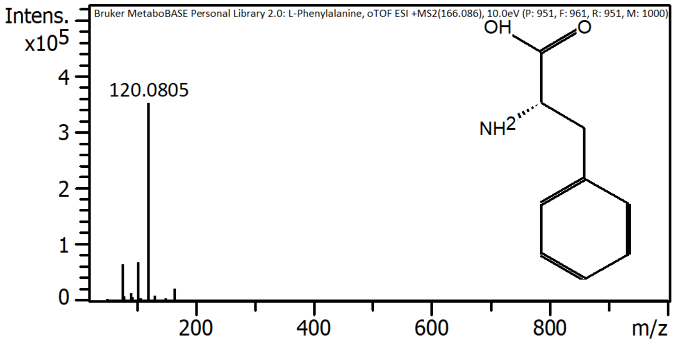

#	m/z	Res.	S/N	I	I %	FWHM
1	77.0391	5472	36.8	64775	18.4	0.0141
2	79.0546	5615	5.0	8801	2.5	0.0141
3	91.0542	6467	8.2	14434	4.1	0.0141
4	93.0702	6610	4.0	7041	2.0	0.0141
5	103.0542	7319	39.8	70055	19.9	0.0141
6	107.0486	7603	2.8	4929	1.4	0.0141
7	120.0805	8529	199.8	351685	100.0	0.0141
8	131.0482	9308	5.2	9153	2.6	0.0141
9	149.0586	10587	2.6	4576	1.3	0.0141
10	166.0842	11796	12.8	22530	6.4	0.0141

Cmpd 213, AutoMSn(166.0861), 2.71 min

Compound Spectrum List Report

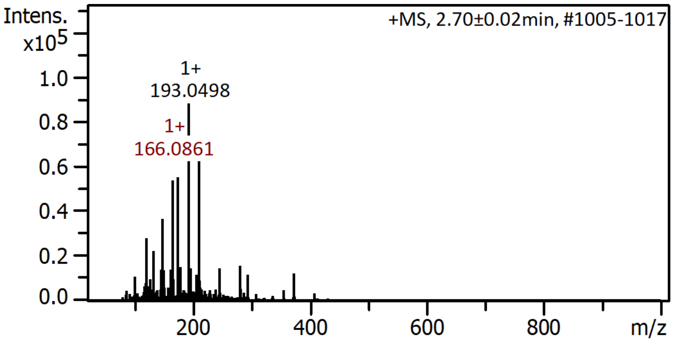

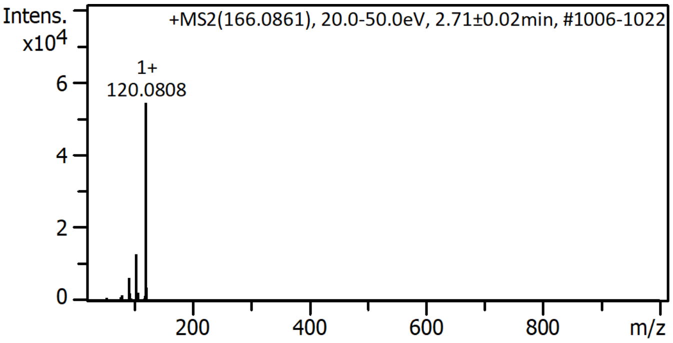

#	m/z	Res.	S/N	I	I %	FWHM
1	120.0807	10416	2340.0	28080	31.8	0.0115
2	132.1019	10673	1878.2	22538	25.6	0.0124
3	147.0441	10654	3057.2	36686	41.6	0.0138
4	166.0861	11195	4487.1	53845	61.1	0.0148
5	175.0394	10955	4600.8	55210	62.6	0.0160
6	179.0704	11574	1246.6	14959	17.0	0.0155
7	193.0498	11678	7348.3	88180	100.0	0.0165
8	197.0818	10734	1212.6	14551	16.5	0.0184
9	211.0602	11653	5819.9	69839	79.2	0.0181
10	281.1065	11857	1290.6	15487	17.6	0.0237

#	m/z	Res.	S/N	I	I %	FWHM
1	77.0391	8597	53.2	940	1.7	0.0090
2	79.0538	7323	82.3	1454	2.7	0.0108
3	91.0540	8984	352.2	6223	11.5	0.0101
4	93.0704	11050	109.4	1932	3.6	0.0084
5	103.0547	9606	733.5	12958	23.9	0.0107
6	104.0560	7992	54.8	969	1.8	0.0130
7	107.0497	9786	119.5	2111	3.9	0.0109
8	119.0495	10256	73.0	1289	2.4	0.0116
9	120.0808	10050	3071.6	54265	100.0	0.0119
10	121.0834	11272	203.9	3602	6.6	0.0107

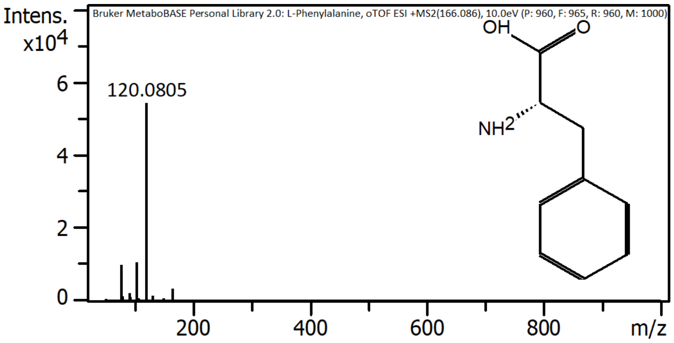

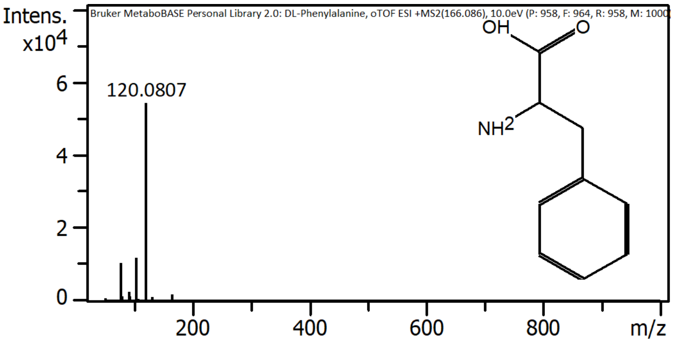

#	m/z	Res.	S/N	I	I %	FWHM
1	77.0391	6395	36.8	9985	18.4	0.0120
2	79.0546	6563	5.0	1357	2.5	0.0120
3	91.0542	7559	8.2	2225	4.1	0.0120
4	93.0702	7726	4.0	1085	2.0	0.0120
5	103.0542	8555	39.8	10799	19.9	0.0120
6	107.0486	8887	2.8	760	1.4	0.0120
7	120.0805	9968	199.8	54210	100.0	0.0120
8	131.0482	10879	5.2	1411	2.6	0.0120
9	149.0586	12374	2.6	705	1.3	0.0120
10	166.0842	13787	12.8	3473	6.4	0.0120

#	m/z	Res.	S/N	I	I %	FWHM
1	51.0237	4236	3.2	868	1.6	0.0120
2	77.0394	6395	39.0	10582	19.5	0.0120
3	79.0544	6563	5.0	1357	2.5	0.0120
4	91.0550	7559	9.2	2496	4.6	0.0120
5	93.0690	7726	4.8	1302	2.4	0.0120
6	103.0544	8555	44.0	11938	22.0	0.0120
7	107.0495	8887	2.0	543	1.0	0.0120
8	120.0807	9968	199.8	54210	100.0	0.0120
9	131.0487	10879	4.4	1194	2.2	0.0120
10	166.0879	13788	6.6	1791	3.3	0.0120

Cmpd 262, AutoMSn(127.0395), 3.34 min

Compound Spectrum List Report

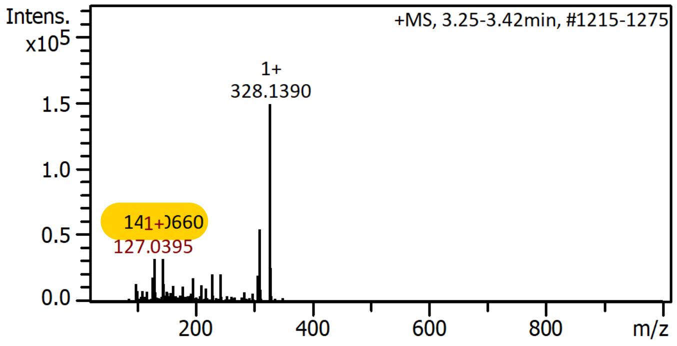

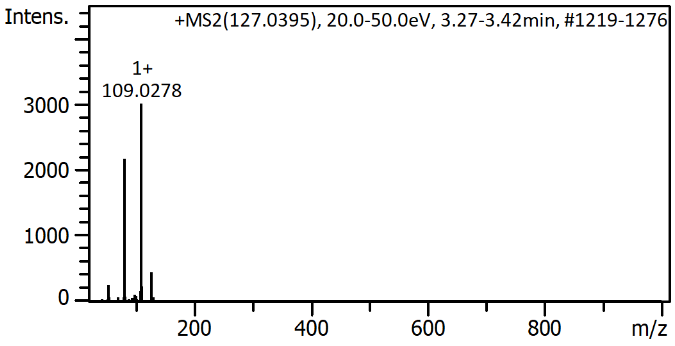

#	m/z	Res.	S/N	I	I %	FWHM
1	127.0395	9925	3007.9	18047	12.1	0.0128
2	130.1591	10368	7041.8	42251	28.4	0.0126
3	144.0660	10374	8368.4	50211	33.7	0.0139
4	197.1170	11370	2903.3	17420	11.7	0.0173
5	229.0710	11912	3399.2	20395	13.7	0.0192
6	244.1547	11917	3426.5	20559	13.8	0.0205
7	307.1690	12969	3289.8	19739	13.3	0.0237
8	310.1289	12106	9099.9	54600	36.7	0.0256
9	328.1390	12584	24796.0	148776	100.0	0.0261
10	329.1423	12136	4195.0	25170	16.9	0.0271

#	m/z	Res.	S/N	I	I %	FWHM
1	53.0385	8710	22.8	250	8.3	0.0061
2	81.0340	9404	197.3	2171	72.2	0.0086
3	82.0753	5034	6.8	75	2.5	0.0163
4	98.0618	13181	9.5	104	3.5	0.0074
5	100.0791	7018	7.8	86	2.9	0.0143
6	108.0460	8676	14.2	157	5.2	0.0125
7	109.0278	10169	273.2	3005	100.0	0.0107
8	110.0323	10501	20.9	230	7.6	0.0105
9	126.0531	14340	9.6	106	3.5	0.0088
10	127.0371	11421	40.1	441	14.7	0.0111

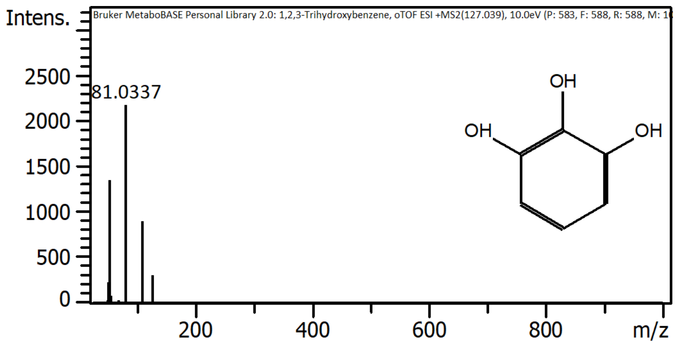

#	m/z	Res.	S/N	I	I %	FWHM
1	50.0138	4600	3.2	35	1.6	0.0109
2	51.0231	4693	21.2	230	10.6	0.0109
3	52.0328	4786	2.2	24	1.1	0.0109
4	53.0388	4878	124.4	1350	62.3	0.0109
5	55.0182	5061	7.6	82	3.8	0.0109
6	68.9975	6346	2.8	30	1.4	0.0109
7	81.0337	7453	199.8	2169	100.0	0.0109
8	81.1130	7461	6.6	72	3.3	0.0109
9	109.0274	10028	82.8	899	41.4	0.0109
10	127.0386	11685	28.4	308	14.2	0.0109

Cmpd 289, AutoMSn(282.1205), 3.67 min

Compound Spectrum List Report

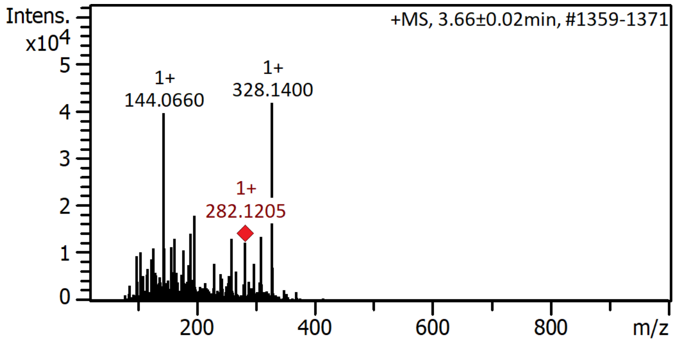

#	m/z	Res.	S/N	I	I %	FWHM
1	127.0394	10289	932.3	11187	26.8	0.0123
2	144.0660	10316	3297.4	39569	94.8	0.0140
3	157.0844	10946	953.6	11444	27.4	0.0144
4	163.0608	10578	1094.2	13130	31.5	0.0154
5	190.1078	11852	1186.7	14240	34.1	0.0160
6	197.1174	11527	1500.9	18011	43.2	0.0171
7	260.1501	11954	1098.5	13182	31.6	0.0218
8	282.1205	11311	1029.2	12351	29.6	0.0249
9	310.1302	11448	1126.5	13518	32.4	0.0271
10	328.1400	13155	3476.7	41720	100.0	0.0249

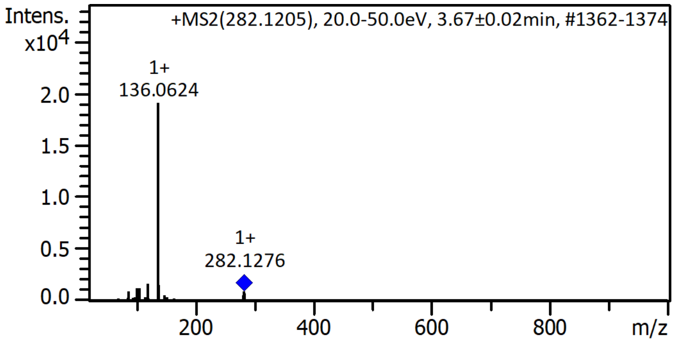

#	m/z	Res.	S/N	I	I %	FWHM
1	87.0813	8245	55.2	883	4.6	0.0106
2	101.0608	10586	73.4	1174	6.2	0.0095
3	105.0908	8938	76.5	1225	6.4	0.0118
4	119.0341	8642	102.4	1638	8.6	0.0138
5	136.0624	9939	1192.8	19085	100.0	0.0137
6	137.0636	7932	93.7	1499	7.9	0.0173
7	281.1060	8317	34.0	544	2.9	0.0338
8	282.1276	10202	55.3	884	4.6	0.0277
9	282.1688	8324	51.2	819	4.3	0.0339
10	283.1726	9630	43.5	696	3.6	0.0294

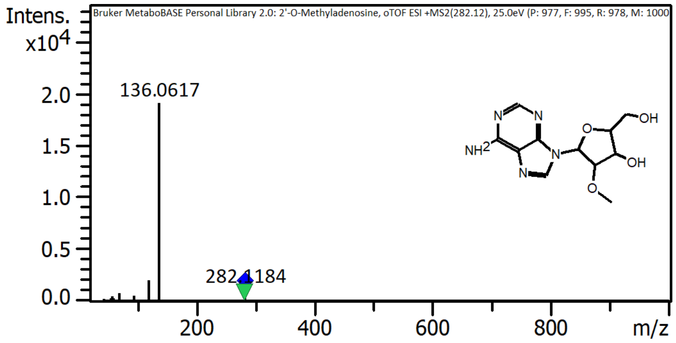

#	m/z	Res.	S/N	I	I %	FWHM
1	43.0188	3040	1.8	210	1.1	0.0141
2	45.0338	3183	1.5	172	0.9	0.0141
3	55.0185	3888	2.2	248	1.3	0.0141
4	57.0335	4031	4.0	458	2.4	0.0141
5	59.0498	4173	2.7	305	1.6	0.0141
6	69.0339	4879	6.7	763	4.0	0.0141
7	94.0397	6646	4.5	515	2.7	0.0141
8	119.0351	8413	17.7	2023	10.6	0.0141
9	136.0617	9616	166.5	19066	100.0	0.0141
10	282.1184	19938	10.0	1145	6.0	0.0141

Cmpd 373, AutoMSn(193.0499), 4.63 min

Compound Spectrum List Report

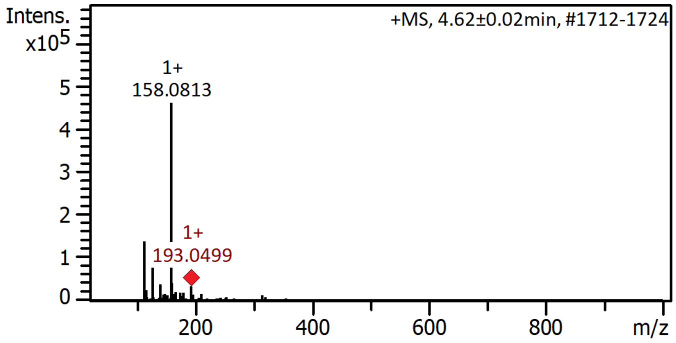

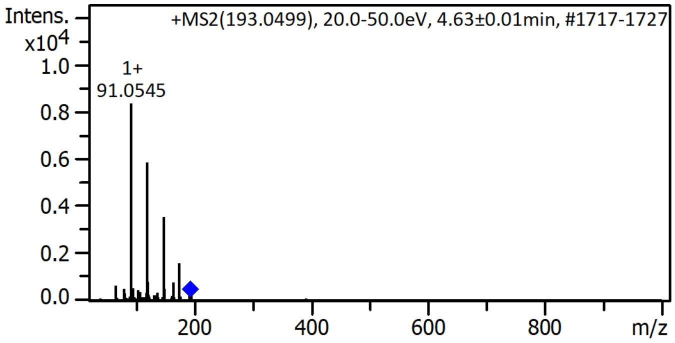

#	m/z	Res.	S/N	I	I %	FWHM
1	112.0756	10032	11545.4	138544	30.1	0.0112
2	116.0704	10056	2104.2	25250	5.5	0.0115
3	127.0392	9968	10432.0	125184	27.2	0.0127
4	140.0707	10193	3108.5	37302	8.1	0.0137
5	158.0813	9783	38378.5	460542	100.0	0.0162
6	159.0843	11297	3373.6	40484	8.8	0.0141
7	167.0705	11334	1727.3	20727	4.5	0.0147
8	175.0395	11358	1560.9	18731	4.1	0.0154
9	180.0641	10443	1548.0	18576	4.0	0.0172
10	193.0499	11344	2786.0	33432	7.3	0.0170

#	m/z	Res.	S/N	I	I %	FWHM
1	65.0379	8883	38.9	622	7.5	0.0073
2	79.0546	10379	31.0	497	6.0	0.0076
3	91.0545	8802	521.6	8346	100.0	0.0103
4	92.0590	8648	42.5	680	8.1	0.0106
5	95.0480	9805	33.0	528	6.3	0.0097
6	119.0494	10383	366.2	5860	70.2	0.0115
7	120.0540	8894	49.3	789	9.4	0.0135
8	147.0453	11172	222.2	3555	42.6	0.0132
9	165.0534	12728	47.5	760	9.1	0.0130
10	175.0383	14252	100.1	1601	19.2	0.0123

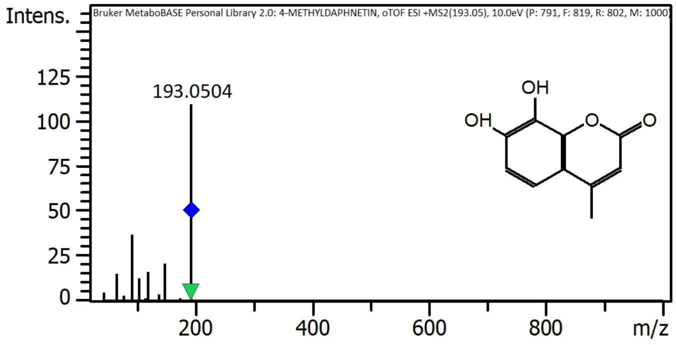

#	m/z	Res.	S/N	I	I %	FWHM
1	43.0190	4798	8.6	5	4.3	0.0090
2	65.0389	7254	27.6	15	13.8	0.0090
3	77.0394	8593	5.8	3	2.9	0.0090
4	91.0544	10156	67.6	37	33.8	0.0090
5	103.0547	11494	22.8	12	11.4	0.0090
6	119.0492	13278	29.4	16	14.7	0.0090
7	137.0605	15287	7.0	4	3.5	0.0090
8	147.0452	16401	38.8	21	19.4	0.0090
9	193.0494	21532	83.2	45	41.6	0.0090
10	193.0504	21532	199.8	109	100.0	0.0090

Cmpd 381, AutoMSn(220.1183), 4.72 min

Compound Spectrum List Report

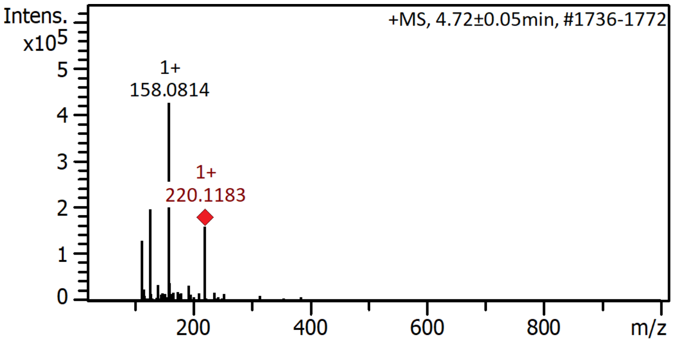

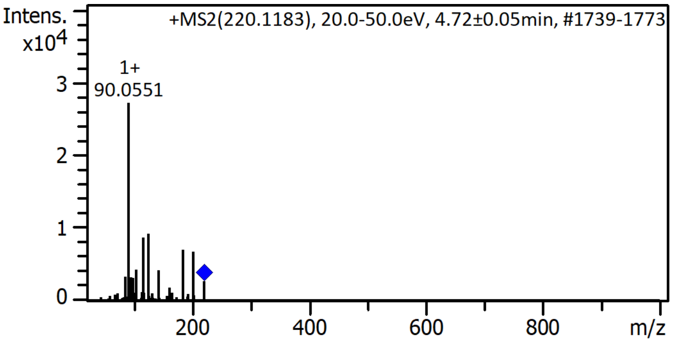

#	m/z	Res.	S/N	I	I %	FWHM
1	112.0756	9732	21593.6	129561	30.5	0.0115
2	116.0706	9918	3973.6	23841	5.6	0.0117
3	127.0399	8950	32931.8	197591	46.5	0.0142
4	140.0707	10311	5524.8	33149	7.8	0.0136
5	158.0814	9785	70852.5	425115	100.0	0.0162
6	159.0844	10366	6321.6	37930	8.9	0.0153
7	175.0393	11020	3003.6	18022	4.2	0.0159
8	193.0501	11422	5460.0	32760	7.7	0.0169
9	220.1183	11279	26783.1	160699	37.8	0.0195
10	221.1221	10672	3211.3	19268	4.5	0.0207

#	m/z	Res.	S/N	I	I %	FWHM
1	85.0649	8349	461.6	3308	12.2	0.0102
2	90.0551	8876	3791.4	27172	100.0	0.0101
3	95.0487	8773	449.5	3221	11.9	0.0108
4	98.0238	9228	433.8	3109	11.4	0.0106
5	103.0751	10159	599.7	4298	15.8	0.0101
6	116.0346	9885	1221.0	8750	32.2	0.0117
7	124.0756	9849	1295.0	9281	34.2	0.0126
8	142.0863	10636	577.2	4137	15.2	0.0134
9	184.0972	10730	981.9	7037	25.9	0.0172
10	202.1075	11448	949.0	6801	25.0	0.0177

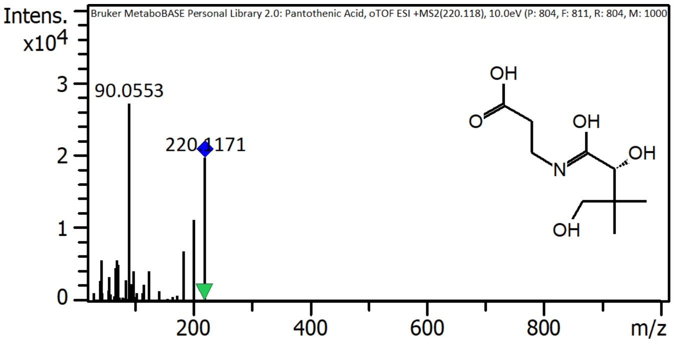

#	m/z	Res.	S/N	I	I %	FWHM
1	43.0181	4664	34.5	5625	20.7	0.0092
2	67.0548	7270	28.0	4565	16.8	0.0092
3	70.0293	7593	35.0	5706	21.0	0.0092
4	72.0453	7811	30.7	5000	18.4	0.0092
5	90.0553	9764	166.5	27145	100.0	0.0092
6	98.0235	10628	25.2	4103	15.1	0.0092
7	124.0761	13453	25.2	4103	15.1	0.0092
8	184.0964	19960	42.2	6875	25.3	0.0092
9	202.1071	21913	68.8	11222	41.3	0.0092
10	220.1171	23865	120.8	19700	72.6	0.0092

Cmpd 405, AutoMSn(220.1180), 5.01 min

Compound Spectrum List Report

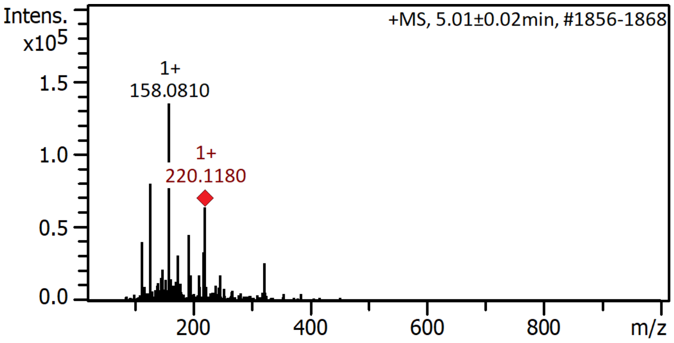

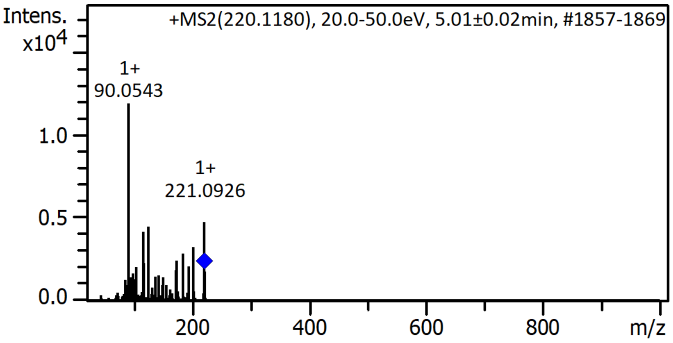

#	m/z	Res.	S/N	I	I %	FWHM
1	112.0757	9529	3362.1	40345	29.9	0.0118
2	127.0392	9609	6670.9	80051	59.3	0.0132
3	147.0440	10027	1781.2	21374	15.8	0.0147
4	158.0810	10496	11241.3	134896	100.0	0.0151
5	175.0390	10995	2598.9	31186	23.1	0.0159
6	193.0499	11646	3785.6	45427	33.7	0.0166
7	218.1390	11000	2794.5	33534	24.9	0.0198
8	220.1180	10859	5332.4	63989	47.4	0.0203
9	247.0817	11510	1455.9	17470	13.0	0.0215
10	322.2000	11065	2132.3	25588	19.0	0.0291

#	m/z	Res.	S/N	I	I %	FWHM
1	90.0543	9000	758.1	11878	100.0	0.0100
2	103.0744	10505	129.9	2035	17.1	0.0098
3	116.0338	10287	267.1	4185	35.2	0.0113
4	117.0550	9928	145.4	2278	19.2	0.0118
5	124.0752	10292	286.2	4484	37.8	0.0121
6	173.1358	10469	157.1	2461	20.7	0.0165
7	184.0959	8319	183.8	2879	24.2	0.0221
8	194.0811	12625	131.4	2059	17.3	0.0154
9	202.1089	10831	206.9	3241	27.3	0.0187
10	221.0926	10574	302.9	4746	40.0	0.0209

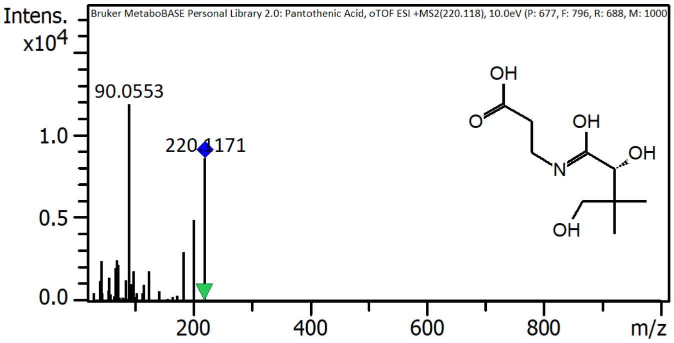

#	m/z	Res.	S/N	I	I %	FWHM
1	43.0181	4828	34.5	2459	20.7	0.0089
2	67.0548	7525	28.0	1995	16.8	0.0089
3	70.0293	7859	35.0	2494	21.0	0.0089
4	72.0453	8085	30.7	2185	18.4	0.0089
5	90.0553	10107	166.5	11866	100.0	0.0089
6	98.0235	11001	25.2	1794	15.1	0.0089
7	124.0761	13925	25.2	1794	15.1	0.0089
8	184.0964	20661	42.2	3005	25.3	0.0089
9	202.1071	22682	68.8	4905	41.3	0.0089
10	220.1171	24703	120.8	8611	72.6	0.0089

Cmpd 418, AutoMSn(205.0968), 5.17 min

Compound Spectrum List Report

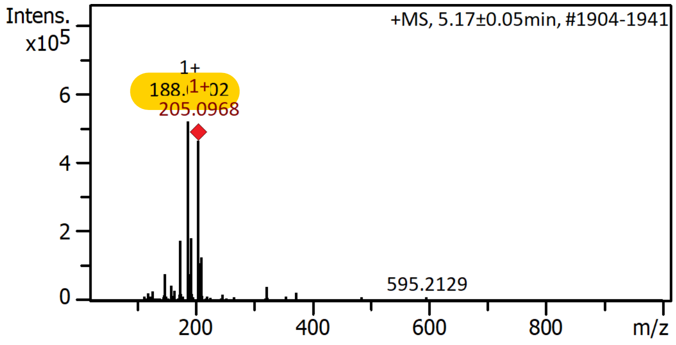

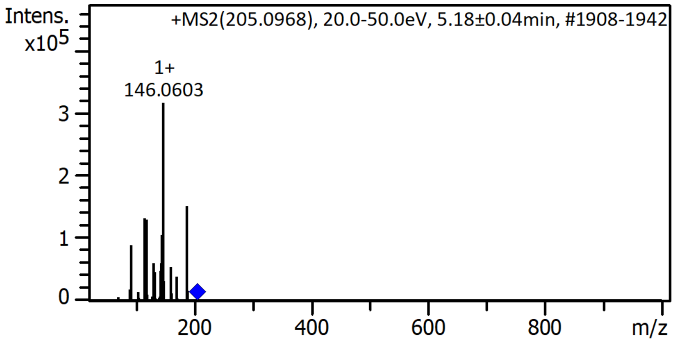

#	m/z	Res.	S/N	I	I %	FWHM
1	147.0438	10592	12744.2	76465	13.3	0.0139
2	158.0806	10687	7111.5	42669	7.4	0.0148
3	175.0388	10982	29367.2	176203	30.7	0.0159
4	188.0702	10383	95581.2	573487	100.0	0.0181
5	189.0736	11010	12887.2	77323	13.5	0.0172
6	193.0491	10839	30584.4	183506	32.0	0.0178
7	205.0968	10462	77390.2	464341	81.0	0.0196
8	206.1066	6058	18207.5	109245	19.0	0.0340
9	211.0597	11628	21192.2	127153	22.2	0.0182
10	322.1997	11312	6531.3	39188	6.8	0.0285

#	m/z	Res.	S/N	I	I %	FWHM
1	91.0543	8774	16289.5	89592	28.3	0.0104
2	115.0541	9543	24106.9	132588	42.0	0.0121
3	117.0578	9394	18208.7	100148	31.7	0.0125
4	118.0649	9530	23717.8	130448	41.3	0.0124
5	130.0651	10150	10900.1	59950	19.0	0.0128
6	143.0727	10443	10928.3	60106	19.0	0.0137
7	144.0806	10181	19185.1	105518	33.4	0.0142
8	146.0603	9155	57459.5	316028	100.0	0.0160
9	159.0919	10785	9698.7	53343	16.9	0.0148
10	188.0707	10454	27555.4	151555	48.0	0.0180

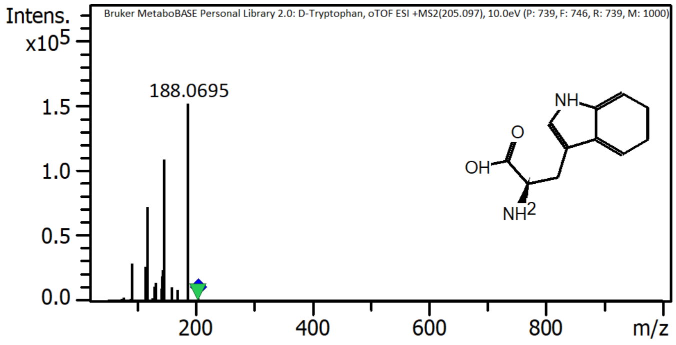

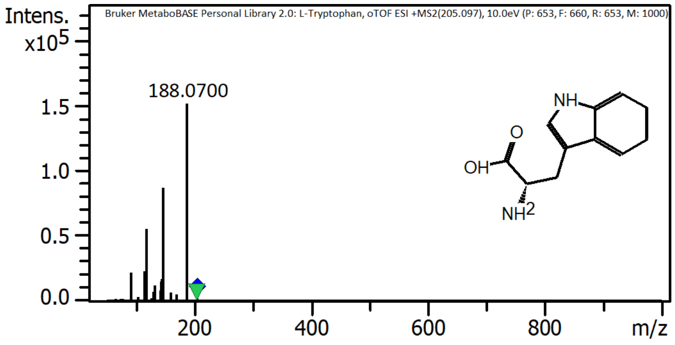

#	m/z	Res.	S/N	I	I %	FWHM
1	91.0537	6048	38.0	28795	19.0	0.0151
2	115.0539	7642	34.4	26067	17.2	0.0151
3	117.0579	7776	26.6	20157	13.3	0.0151
4	118.0646	7842	95.6	72443	47.8	0.0151
5	130.0640	8639	14.6	11063	7.3	0.0151
6	132.0804	8773	18.8	14246	9.4	0.0151
7	143.0722	9503	24.8	18793	12.4	0.0151
8	144.0801	9570	31.2	23643	15.6	0.0151
9	146.0603	9702	143.8	108968	72.0	0.0151
10	188.0695	12492	199.8	151403	100.0	0.0151

#	m/z	Res.	S/N	I	I %	FWHM
1	91.0542	6048	24.2	21975	14.5	0.0151
2	115.0537	7642	25.2	22885	15.1	0.0151
3	117.0565	7775	19.3	17580	11.6	0.0151
4	118.0654	7842	61.2	55621	36.7	0.0151
5	132.0802	8773	13.0	11821	7.8	0.0151
6	142.0630	9436	9.0	8184	5.4	0.0151
7	143.0725	9504	16.5	15004	9.9	0.0151
8	144.0804	9570	18.8	17126	11.3	0.0151
9	146.0593	9702	96.2	87447	57.8	0.0151
10	188.0700	12492	166.5	151403	100.0	0.0151

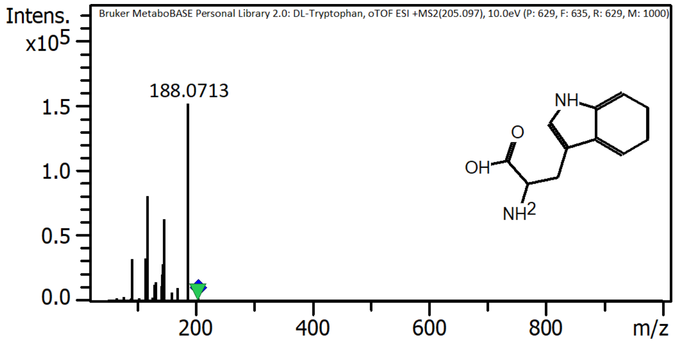

Compound Spectrum List Report

#	m/z	Res.	S/N	I	I %	FWHM
1	91.0550	6048	43.0	32584	21.5	0.0151
2	115.0545	7642	43.6	33039	21.8	0.0151
3	117.0595	7776	30.8	23339	15.4	0.0151
4	118.0650	7842	106.8	80930	53.5	0.0151
5	132.0806	8773	19.4	14701	9.7	0.0151
6	143.0735	9504	27.0	20460	13.5	0.0151
7	144.0802	9570	37.0	28038	18.5	0.0151
8	146.0592	9702	83.2	63047	41.6	0.0151
9	146.0616	9702	69.4	52589	34.7	0.0151
10	188.0713	12493	199.8	151403	100.0	0.0151

Cmpd 445, AutoMSn(205.0969), 5.55 min

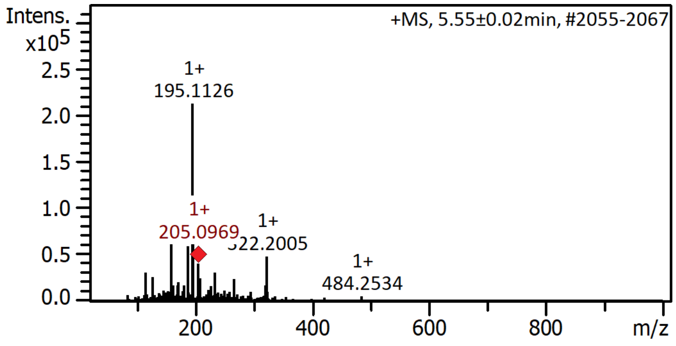

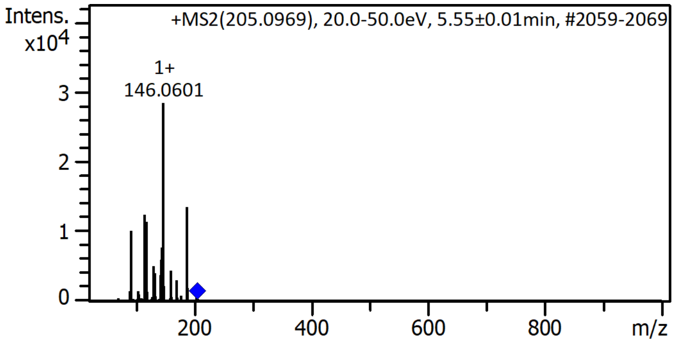

#	m/z	Res.	S/N	I	I %	FWHM
1	114.0911	9785	2572.2	30867	14.5	0.0117
2	127.0391	10225	2150.6	25807	12.1	0.0124
3	158.0810	10547	6487.4	77848	36.6	0.0150
4	188.0716	11057	4936.0	59232	27.9	0.0170
5	195.1126	11162	17718.1	212617	100.0	0.0175
6	196.1129	10876	6885.1	82621	38.9	0.0180
7	197.1165	11603	2397.2	28767	13.5	0.0170
8	205.0969	11127	3436.9	41243	19.4	0.0184
9	234.1481	11541	2549.6	30595	14.4	0.0203
10	322.2005	11860	4071.5	48858	23.0	0.0272

#	m/z	Res.	S/N	I	I %	FWHM
1	91.0544	9210	631.8	10108	35.6	0.0099
2	115.0540	10385	776.3	12421	43.8	0.0111
3	117.0584	8953	658.8	10541	37.1	0.0131
4	118.0652	9420	716.9	11470	40.4	0.0125
5	130.0663	10513	312.5	5000	17.6	0.0124
6	143.0725	11059	376.1	6018	21.2	0.0129
7	144.0807	11108	486.0	7776	27.4	0.0130
8	146.0601	10019	1774.4	28391	100.0	0.0146
9	159.0911	10399	271.3	4340	15.3	0.0153
10	188.0705	10813	847.5	13561	47.8	0.0174

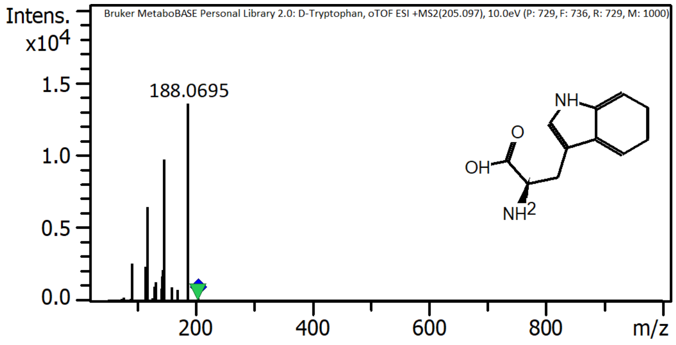

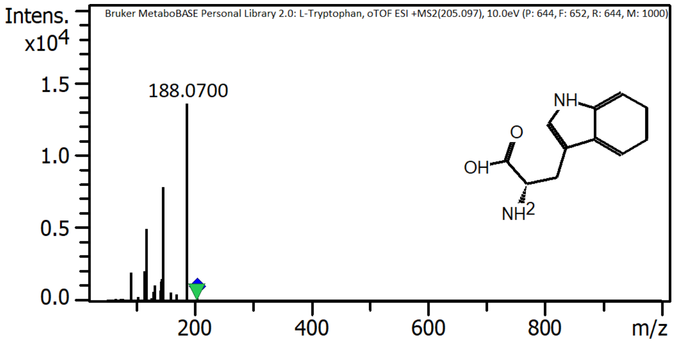

#	m/z	Res.	S/N	I	I %	FWHM
1	91.0537	6276	38.0	2577	19.0	0.0145
2	115.0539	7930	34.4	2332	17.2	0.0145
3	117.0579	8068	26.6	1804	13.3	0.0145
4	118.0646	8138	95.6	6482	47.8	0.0145
5	130.0640	8965	14.6	990	7.3	0.0145
6	132.0804	9104	18.8	1275	9.4	0.0145
7	143.0722	9861	24.8	1682	12.4	0.0145
8	144.0801	9931	31.2	2115	15.6	0.0145
9	146.0603	10067	143.8	9750	72.0	0.0145
10	188.0695	12963	199.8	13547	100.0	0.0145

#	m/z	Res.	S/N	I	I %	FWHM
1	91.0542	6276	24.2	1966	14.5	0.0145
2	115.0537	7930	25.2	2048	15.1	0.0145
3	117.0565	8068	19.3	1573	11.6	0.0145
4	118.0654	8138	61.2	4977	36.7	0.0145
5	132.0802	9104	13.0	1058	7.8	0.0145
6	142.0630	9792	9.0	732	5.4	0.0145
7	143.0725	9862	16.5	1343	9.9	0.0145
8	144.0804	9931	18.8	1532	11.3	0.0145
9	146.0593	10067	96.2	7825	57.8	0.0145
10	188.0700	12963	166.5	13547	100.0	0.0145

Compound Spectrum List Report

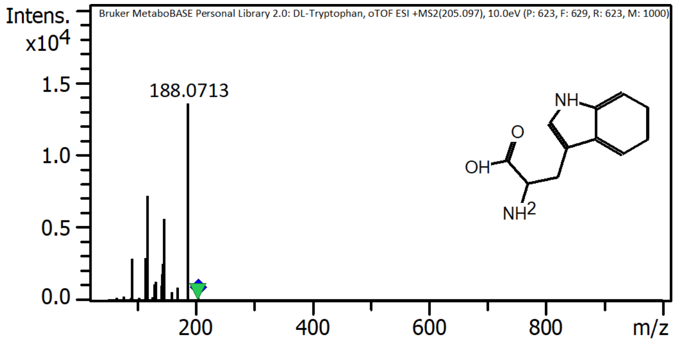

#	m/z	Res.	S/N	I	I %	FWHM
1	91.0550	6276	43.0	2916	21.5	0.0145
2	115.0545	7930	43.6	2956	21.8	0.0145
3	117.0595	8069	30.8	2088	15.4	0.0145
4	118.0650	8138	106.8	7241	53.5	0.0145
5	132.0806	9104	19.4	1315	9.7	0.0145
6	143.0735	9862	27.0	1831	13.5	0.0145
7	144.0802	9931	37.0	2509	18.5	0.0145
8	146.0592	10067	83.2	5641	41.6	0.0145
9	146.0616	10068	69.4	4706	34.7	0.0145
10	188.0713	12963	199.8	13547	100.0	0.0145

Cmpd 480, AutoMSn(193.0502), 5.89 min

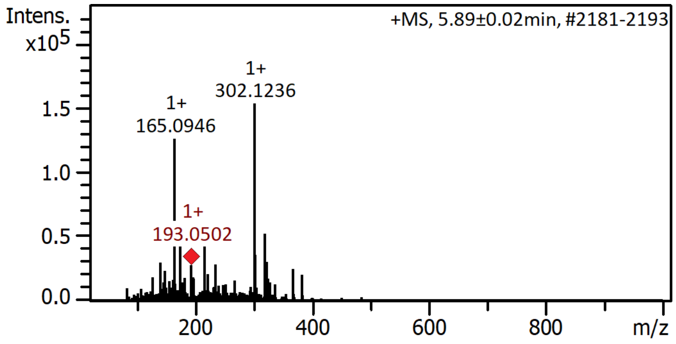

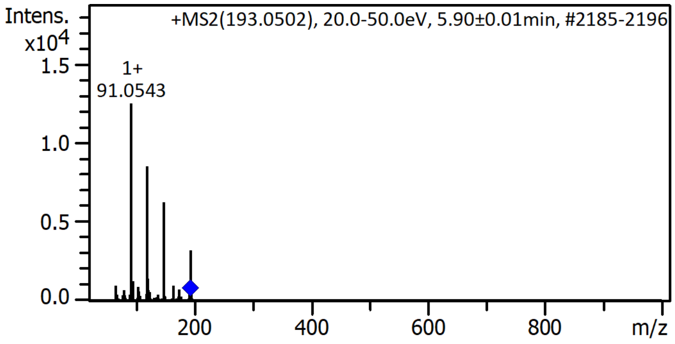

#	m/z	Res.	S/N	I	I %	FWHM
1	140.0712	10009	2521.6	30259	19.7	0.0140
2	165.0946	10716	10519.3	126232	82.3	0.0154
3	175.0394	11556	3899.3	46791	30.5	0.0151
4	193.0502	11546	2288.2	27458	17.9	0.0167
5	216.0868	11434	3906.4	46877	30.5	0.0189
6	235.0606	11508	2350.7	28208	18.4	0.0204
7	302.1236	12234	12787.2	153447	100.0	0.0247
8	303.1298	11738	3013.4	36161	23.6	0.0258
9	319.1510	12584	4369.9	52438	34.2	0.0254
10	322.2010	12779	2544.2	30530	19.9	0.0252

#	m/z	Res.	S/N	I	I %	FWHM
1	91.0543	9095	750.2	12503	100.0	0.0100
2	92.0560	10004	64.8	1080	8.6	0.0092
3	95.0499	9383	73.0	1217	9.7	0.0101
4	119.0492	9997	511.2	8520	68.1	0.0119
5	120.0557	8139	64.0	1067	8.5	0.0148
6	120.0784	10401	82.9	1381	11.0	0.0115
7	147.0442	10440	375.2	6253	50.0	0.0141
8	165.0542	12909	56.8	947	7.6	0.0128
9	192.1014	13084	60.1	1002	8.0	0.0147
10	194.1905	12334	193.7	3229	25.8	0.0157

Compound Spectrum List Report

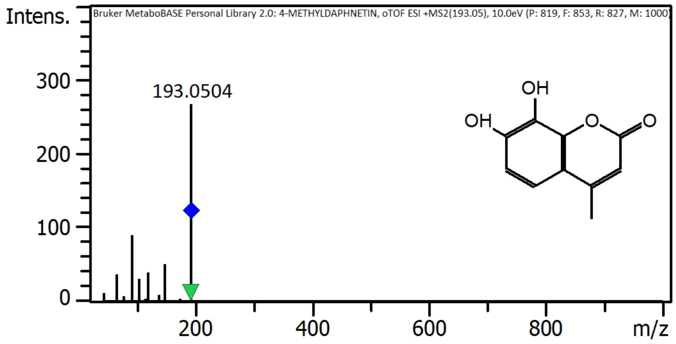

#	m/z	Res.	S/N	I	I %	FWHM
1	43.0190	4762	8.6	12	4.3	0.0090
2	65.0389	7199	27.6	37	13.8	0.0090
3	77.0394	8528	5.8	8	2.9	0.0090
4	91.0544	10079	67.6	90	33.8	0.0090
5	103.0547	11407	22.8	31	11.4	0.0090
6	119.0492	13178	29.4	39	14.7	0.0090
7	137.0605	15171	7.0	9	3.5	0.0090
8	147.0452	16277	38.8	52	19.4	0.0090
9	193.0494	21369	83.2	111	41.6	0.0090
10	193.0504	21369	199.8	267	100.0	0.0090

Cmpd 482, AutoMSn(161.0602), 5.92 min

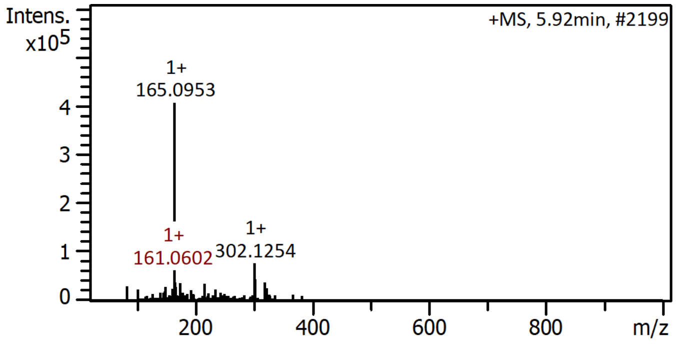

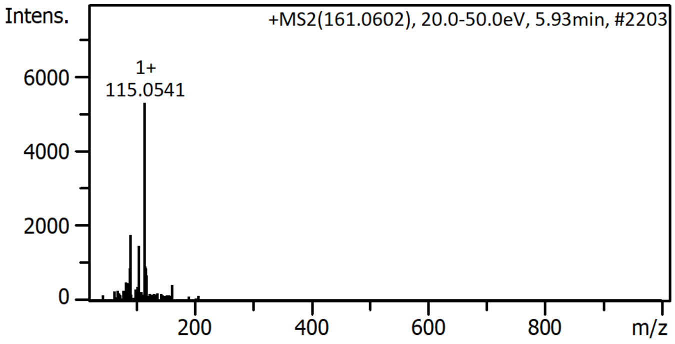

#	m/z	Res.	S/N	I	I %	FWHM
1	83.0860	8918	802.1	28875	7.1	0.0093
2	149.0602	10896	776.8	27966	6.9	0.0137
3	165.0953	10106	11260.4	405373	100.0	0.0163
4	166.0967	10488	1021.9	36787	9.1	0.0158
5	167.0929	8676	771.4	27772	6.9	0.0193
6	175.0399	11570	1009.2	36331	9.0	0.0151
7	216.0882	11271	974.3	35076	8.7	0.0192
8	302.1254	12399	2145.4	77234	19.1	0.0244
9	303.1336	12239	1215.4	43754	10.8	0.0248
10	319.1523	13029	1025.9	36934	9.1	0.0245

#	m/z	Res.	S/N	I	I %	FWHM
1	83.0153	9935	9.0	495	9.4	0.0084
2	86.0619	9712	8.7	478	9.0	0.0089
3	89.0380	6784	15.7	863	16.3	0.0131
4	91.0538	10595	32.1	1765	33.4	0.0086
5	105.0704	7633	26.9	1481	28.0	0.0138
6	115.0541	10139	96.2	5291	100.0	0.0113
7	116.0638	4497	16.7	921	17.4	0.0258
8	117.0449	13123	8.8	486	9.2	0.0089
9	117.0680	6828	15.5	855	16.2	0.0171
10	118.0835	10114	12.3	675	12.8	0.0117

Compound Spectrum List Report

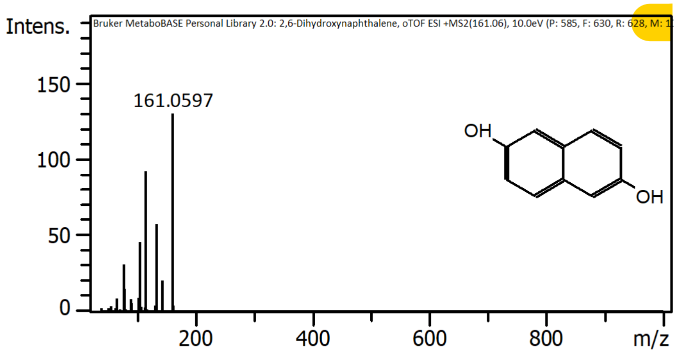

#	m/z	Res.	S/N	I	I %	FWHM
1	77.0396	6658	48.0	31	24.0	0.0116
2	79.0539	6832	23.2	15	11.6	0.0116
3	105.0692	9080	71.0	46	35.5	0.0116
4	105.0719	9081	31.8	21	15.9	0.0116
5	115.0537	9943	141.8	92	71.0	0.0116
6	133.0635	11500	88.8	58	44.4	0.0116
7	133.0652	11500	50.6	33	25.3	0.0116
8	143.0457	12362	27.8	18	13.9	0.0116
9	143.0486	12363	31.6	21	15.8	0.0116
10	161.0597	13919	199.8	130	100.0	0.0116

Cmpd 497, AutoMSn(160.0968), 6.12 min

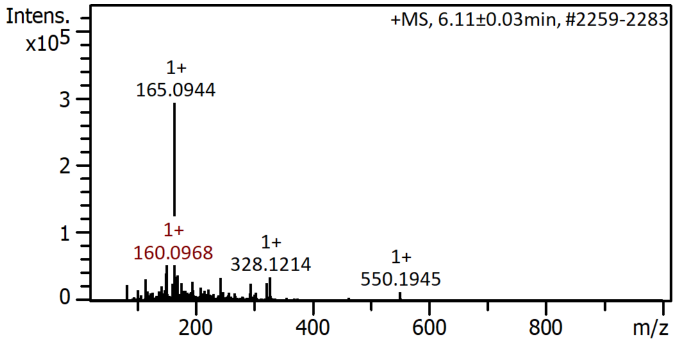

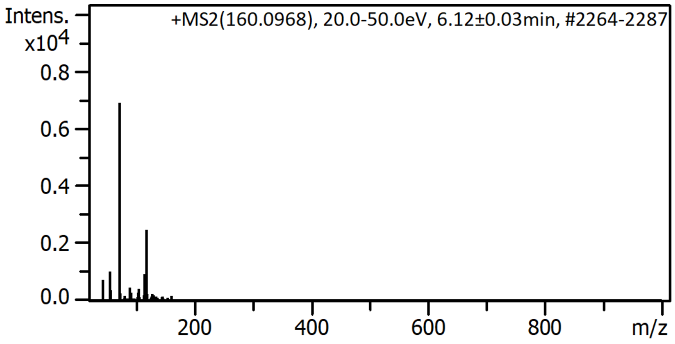

#	m/z	Res.	S/N	I	I %	FWHM
1	114.0913	10023	3525.4	31728	10.8	0.0114
2	149.0597	10472	4472.0	40248	13.8	0.0142
3	151.0756	10923	10686.6	96179	32.9	0.0138
4	165.0944	10216	32518.7	292669	100.0	0.0162
5	166.0971	10258	3068.4	27616	9.4	0.0162
6	167.0789	5818	3373.7	30363	10.4	0.0287
7	169.0864	10796	3953.1	35578	12.2	0.0157
8	170.0820	10244	4183.8	37655	12.9	0.0166
9	244.1545	11724	3757.3	33816	11.6	0.0208
10	328.1214	12940	3808.8	34279	11.7	0.0254

#	m/z	Res.	S/N	I	I %	FWHM
1	43.0180	6738	59.0	723	10.5	0.0064
2	55.0550	5066	82.9	1016	14.7	0.0109
3	57.0593	7919	29.8	365	5.3	0.0072
4	72.0808	7735	562.7	6893	100.0	0.0093
5	89.0403	9373	36.8	451	6.5	0.0095
6	105.0719	11535	33.3	408	5.9	0.0091
7	114.0913	10864	76.3	935	13.6	0.0105
8	115.0548	11555	38.5	472	6.8	0.0100
9	117.0589	12048	49.0	601	8.7	0.0097
10	118.0862	10060	202.6	2481	36.0	0.0117

Compound Spectrum List Report

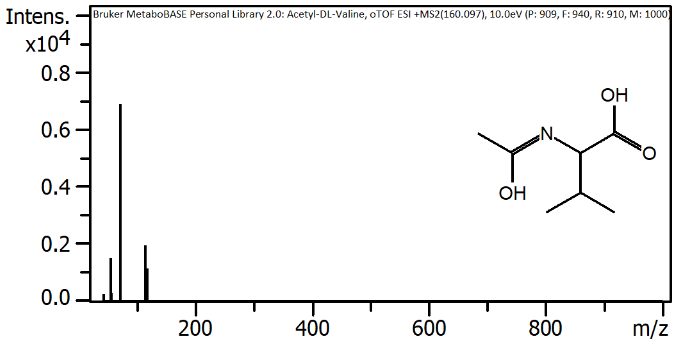

#	m/z	Res.	S/N	I	I %	FWHM
1	43.0191	5276	7.6	262	3.8	0.0082
2	43.0204	5276	2.4	83	1.2	0.0082
3	55.0549	6752	36.2	1248	18.1	0.0082
4	55.0551	6752	44.4	1530	22.2	0.0082
5	56.0517	6875	2.4	83	1.2	0.0082
6	57.0573	6998	8.8	303	4.4	0.0082
7	72.0813	8841	199.8	6886	100.0	0.0082
8	72.1586	8850	4.0	138	2.0	0.0082
9	114.0910	13993	57.4	1978	28.7	0.0082
10	118.0860	14483	33.0	1137	16.5	0.0082

Cmpd 518, AutoMSn(169.0787), 6.34 min

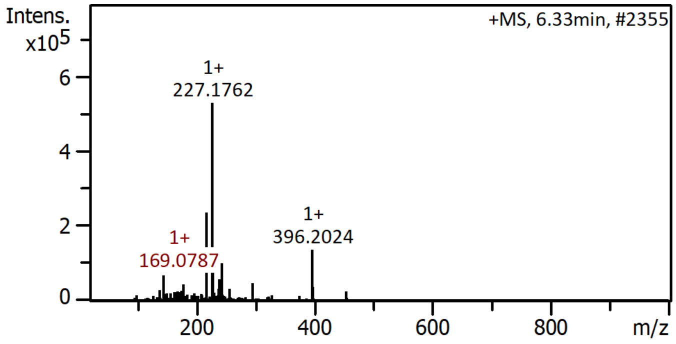

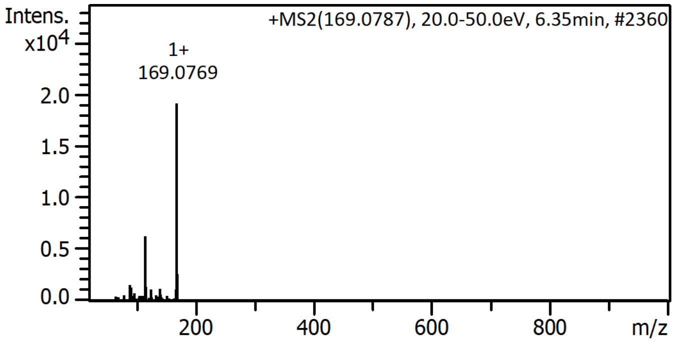

#	m/z	Res.	S/N	I	I %	FWHM
1	144.0830	9798	1879.4	67657	12.8	0.0147
2	179.0714	11294	1191.2	42883	8.1	0.0159
3	217.0977	11425	6551.6	235856	44.6	0.0190
4	227.1762	10955	14677.8	528401	100.0	0.0207
5	228.1793	11168	2145.4	77234	14.6	0.0204
6	239.0933	11770	1587.3	57142	10.8	0.0203
7	244.1546	12490	2833.8	102018	19.3	0.0195
8	295.1046	11089	1285.1	46262	8.8	0.0266
9	396.2024	12612	3818.9	137482	26.0	0.0314
10	397.2049	11507	1012.8	36460	6.9	0.0345

#	m/z	Res.	S/N	I	I %	FWHM
1	89.0404	10609	27.7	1498	7.8	0.0084
2	91.0557	10723	23.6	1274	6.7	0.0085
3	96.0811	11519	13.3	720	3.8	0.0083
4	115.0546	9726	116.5	6289	32.9	0.0118
5	116.0557	5529	24.9	1345	7.0	0.0210
6	124.0767	11893	20.6	1114	5.8	0.0104
7	140.0520	10436	21.3	1148	6.0	0.0134
8	168.0673	9228	19.9	1077	5.6	0.0182
9	169.0769	11002	353.7	19102	100.0	0.0154
10	170.0818	12454	48.0	2593	13.6	0.0137

Compound Spectrum List Report

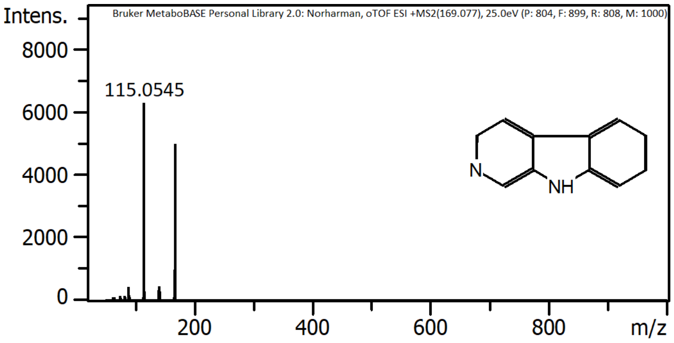

#	m/z	Res.	S/N	I	I %	FWHM
1	81.9904	5162	5.0	157	2.5	0.0159
2	89.0399	5605	13.6	428	6.8	0.0159
3	90.0065	5666	5.8	182	2.9	0.0159
4	115.0545	7243	199.8	6283	100.0	0.0159
5	116.0502	7306	9.4	296	4.7	0.0159
6	140.0485	8817	10.6	333	5.3	0.0159
7	141.0573	8880	14.6	459	7.3	0.0159
8	142.0650	8944	9.2	289	4.6	0.0159
9	168.0681	10581	31.0	975	15.5	0.0159
10	169.0761	10644	158.8	4993	79.5	0.0159

Cmpd 545, AutoMSn(165.0551), 6.58 min

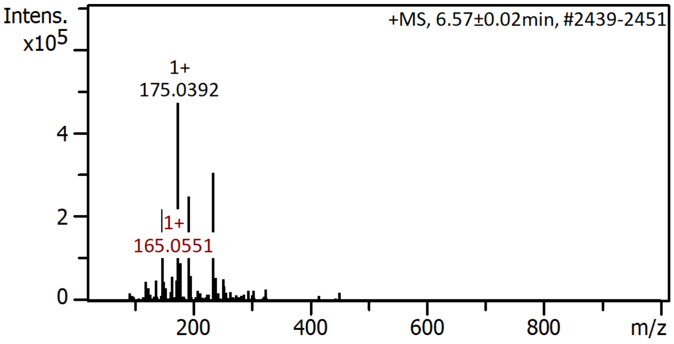

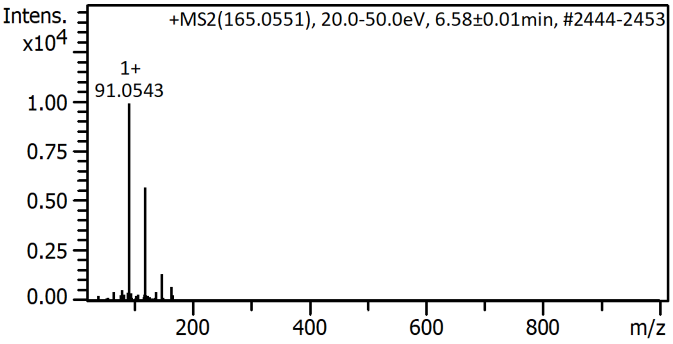

#	m/z	Res.	S/N	I	I %	FWHM
1	147.0442	10723	18208.1	218497	46.4	0.0137
2	165.0551	10779	4706.4	56477	12.0	0.0153
3	175.0392	10354	39266.1	471193	100.0	0.0169
4	176.0428	11128	4299.9	51599	11.0	0.0158
5	179.0722	10784	7463.1	89557	19.0	0.0166
6	193.0500	11124	20787.3	249447	52.9	0.0174
7	197.0812	11451	4891.5	58698	12.5	0.0172
8	235.0606	11576	25418.4	305021	64.7	0.0203
9	239.0920	12089	4565.7	54789	11.6	0.0198
10	252.1230	12092	4305.4	51664	11.0	0.0208

#	m/z	Res.	S/N	I	I %	FWHM
1	65.0391	8250	26.8	447	4.5	0.0079
2	79.0527	10202	31.5	525	5.3	0.0077
3	89.0374	10637	23.2	386	3.9	0.0084
4	91.0543	8809	592.6	9877	100.0	0.0103
5	92.0578	9866	60.5	1008	10.2	0.0093
6	93.0711	7972	21.5	359	3.6	0.0117
7	119.0490	9556	340.4	5673	57.4	0.0125
8	138.0537	11919	25.7	429	4.3	0.0116
9	147.0441	11537	80.2	1337	13.5	0.0127
10	165.0557	10750	41.4	690	7.0	0.0154

Compound Spectrum List Report

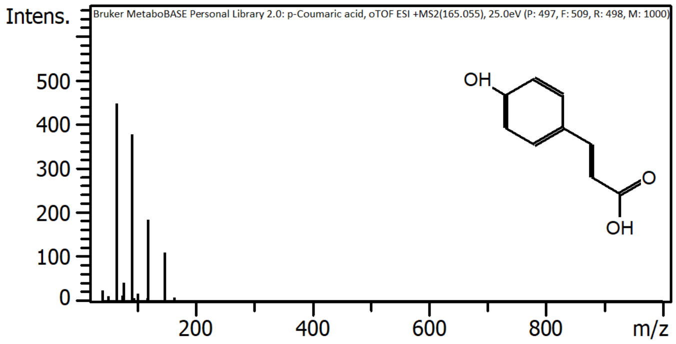

#	m/z	Res.	S/N	I	I %	FWHM
1	41.0378	4282	11.6	26	5.8	0.0096
2	65.0397	6786	199.8	447	100.0	0.0096
3	77.0372	8038	19.2	43	9.6	0.0096
4	91.0543	9500	168.6	377	84.4	0.0096
5	101.0308	10541	8.2	18	4.1	0.0096
6	119.0491	12421	83.2	186	41.6	0.0096
7	119.0503	12421	6.4	14	3.2	0.0096
8	119.0759	12424	6.6	15	3.3	0.0096
9	147.0434	15342	50.0	112	25.0	0.0096
10	147.0437	15342	45.2	101	22.6	0.0096

Cmpd 546, AutoMSn(136.0755), 6.61 min

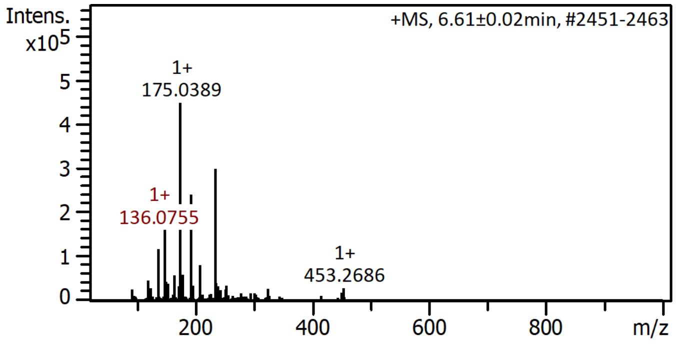

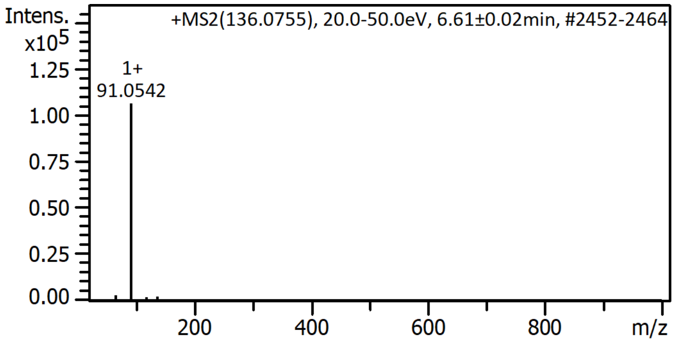

#	m/z	Res.	S/N	I	I %	FWHM
1	119.0489	10158	3849.5	46194	10.3	0.0117
2	136.0755	10614	9885.3	118623	26.5	0.0128
3	147.0441	10456	17943.4	215320	48.1	0.0141
4	165.0546	10910	4716.7	56600	12.6	0.0151
5	175.0389	10354	37308.7	447704	100.0	0.0169
6	176.0423	11352	4148.9	49786	11.1	0.0155
7	179.0718	10929	4867.6	58411	13.0	0.0164
8	193.0497	10820	20084.1	241009	53.8	0.0178
9	209.1532	11491	6679.3	80151	17.9	0.0182
10	235.0601	11524	24936.8	299241	66.8	0.0204

#	m/z	Res.	S/N	I	I %	FWHM
1	39.0238	4397	33.2	521	0.5	0.0089
2	65.0383	7638	183.0	2868	2.7	0.0085
3	89.0367	8168	38.8	607	0.6	0.0109
4	91.0542	8341	6773.0	106110	100.0	0.0109
5	92.0576	9773	548.2	8589	8.1	0.0094
6	93.0674	6490	28.0	439	0.4	0.0143
7	118.0636	9976	112.9	1769	1.7	0.0118
8	119.0460	10416	52.3	819	0.8	0.0114
9	132.0791	9808	45.5	713	0.7	0.0135
10	136.0731	11259	145.7	2282	2.2	0.0121

Compound Spectrum List Report

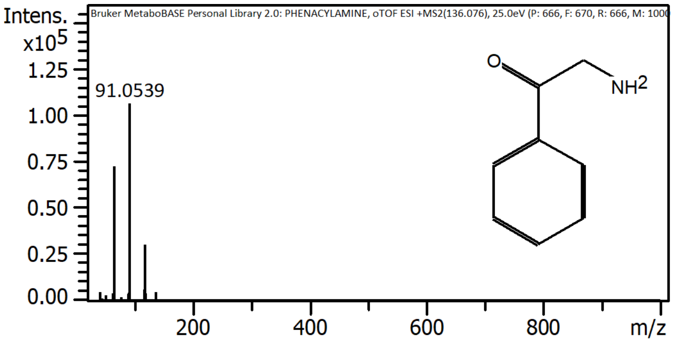

#	m/z	Res.	S/N	I	I %	FWHM
1	41.0391	3808	7.2	4563	4.3	0.0108
2	63.0229	5847	6.0	3820	3.6	0.0108
3	65.0386	6034	113.8	72473	68.4	0.0108
4	89.0387	8261	5.7	3608	3.4	0.0108
5	90.0452	8354	6.7	4244	4.0	0.0108
6	91.0539	8448	166.5	106004	100.0	0.0108
7	117.0558	10860	9.7	6154	5.8	0.0108
8	118.0642	10954	48.0	30560	28.8	0.0108
9	119.0490	11045	6.2	3926	3.7	0.0108
10	136.0767	12625	7.2	4563	4.3	0.0108

Cmpd 554, AutoMSn(163.0690), 6.68 min

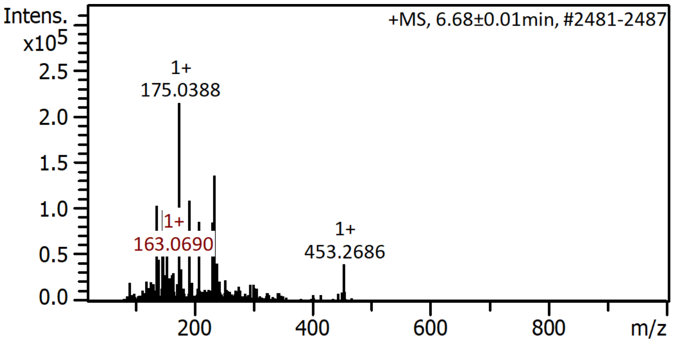

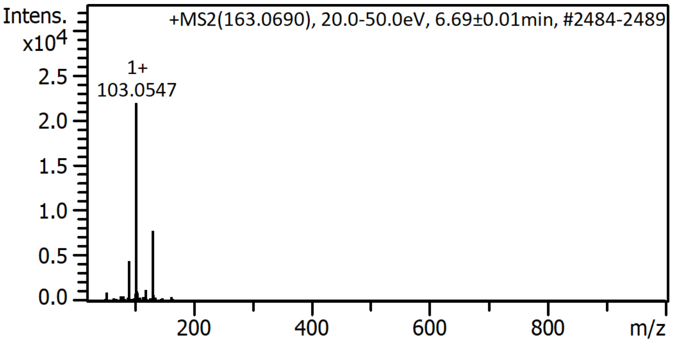

#	m/z	Res.	S/N	I	I %	FWHM
1	136.0751	10650	5755.8	103604	48.4	0.0128
2	140.0707	10556	2507.3	45132	21.1	0.0133
3	146.0597	10527	2957.6	53236	24.8	0.0139
4	147.0440	10460	5493.8	98888	46.2	0.0141
5	153.1269	10892	3629.4	65330	30.5	0.0141
6	175.0388	11077	11902.6	214248	100.0	0.0158
7	193.0495	10900	6043.5	108784	50.8	0.0177
8	209.1531	11587	4778.0	86004	40.1	0.0181
9	231.1124	11746	4736.0	85248	39.8	0.0197
10	235.0601	12081	7570.4	136268	63.6	0.0195

#	m/z	Res.	S/N	I	I %	FWHM
1	53.0378	7923	38.2	956	4.4	0.0067
2	77.0393	10076	21.9	549	2.5	0.0076
3	91.0539	9952	179.4	4484	20.5	0.0091
4	102.0454	10489	31.8	795	3.6	0.0097
5	103.0547	9446	875.4	21886	100.0	0.0109
6	104.0583	8414	45.3	1132	5.2	0.0124
7	105.0445	8714	35.3	883	4.0	0.0121
8	119.0495	12878	50.3	1259	5.8	0.0092
9	131.0495	11209	312.7	7817	35.7	0.0117
10	132.0506	12234	27.9	697	3.2	0.0108

Compound Spectrum List Report

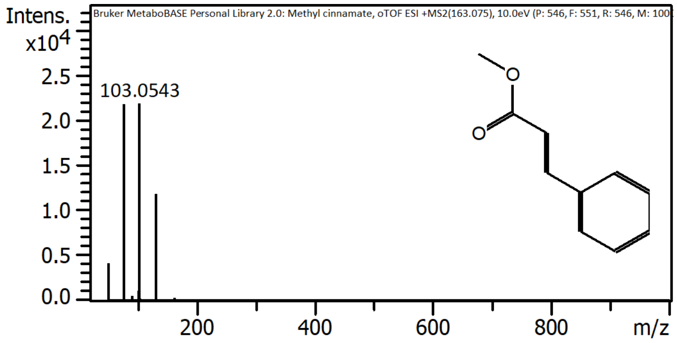

#	m/z	Res.	S/N	I	I %	FWHM
1	51.0236	4923	38.0	4158	19.0	0.0104
2	77.0387	7434	198.6	21733	99.4	0.0104
3	77.1180	7441	6.4	700	3.2	0.0104
4	91.0534	8786	4.8	525	2.4	0.0104
5	102.0456	9847	9.8	1072	4.9	0.0104
6	103.0543	9944	199.8	21864	100.0	0.0104
7	103.1467	9953	6.8	744	3.4	0.0104
8	131.0487	12645	108.4	11862	54.3	0.0104
9	131.1514	12655	2.2	241	1.1	0.0104
10	163.0744	15736	2.6	285	1.3	0.0104

Cmpd 579, AutoMSn(165.0545), 6.92 min

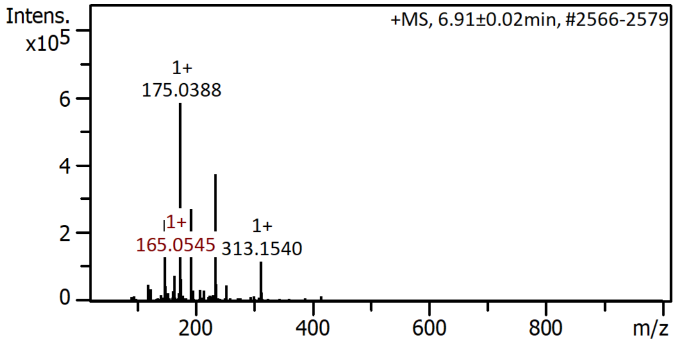

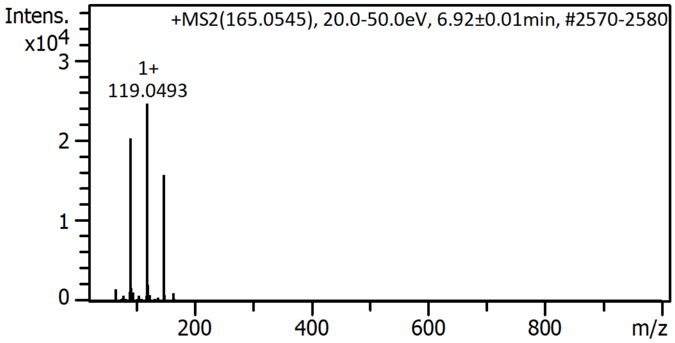

#	m/z	Res.	S/N	I	I %	FWHM
1	119.0486	10203	3989.3	47871	8.2	0.0117
2	147.0438	10650	20023.6	240283	41.2	0.0138
3	165.0545	11205	6266.1	75193	12.9	0.0147
4	175.0388	10133	48589.8	583078	100.0	0.0173
5	176.0424	10886	5416.8	65002	11.1	0.0162
6	193.0493	10987	22678.6	272144	46.7	0.0176
7	235.0598	11100	31139.3	373672	64.1	0.0212
8	236.0634	11646	4173.7	50084	8.6	0.0203
9	253.0705	11972	3817.6	45811	7.9	0.0211
10	313.1540	12043	9816.7	117800	20.2	0.0260

#	m/z	Res.	S/N	I	I %	FWHM
1	65.0391	8245	94.5	1480	6.0	0.0079
2	89.0402	6601	75.0	1175	4.8	0.0135
3	91.0546	8829	1295.0	20289	82.5	0.0103
4	92.0593	9209	104.0	1629	6.6	0.0100
5	95.0509	10027	69.5	1089	4.4	0.0095
6	119.0493	10124	1569.5	24589	100.0	0.0118
7	120.0516	10916	127.3	1994	8.1	0.0110
8	147.0438	10889	1003.9	15728	64.0	0.0135
9	148.0463	7901	47.5	744	3.0	0.0187
10	165.0557	11316	61.3	961	3.9	0.0146

Compound Spectrum List Report

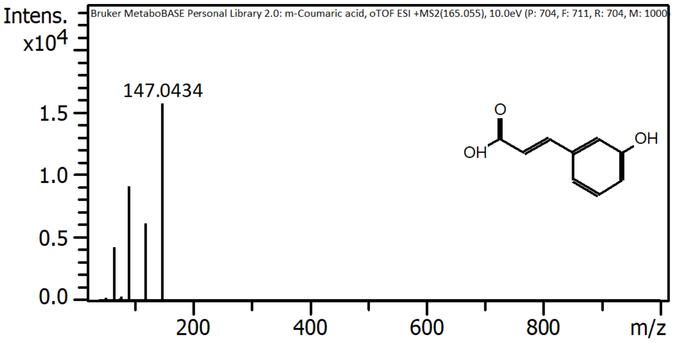

#	m/z	Res.	S/N	I	I %	FWHM
1	41.0374	3392	1.8	142	0.9	0.0121
2	51.0216	4217	2.6	204	1.3	0.0121
3	65.0383	5375	54.8	4310	27.4	0.0121
4	75.0213	6200	2.4	189	1.2	0.0121
5	77.0383	6367	4.2	330	2.1	0.0121
6	91.0538	7526	116.4	9154	58.3	0.0121
7	101.0376	8351	1.8	142	0.9	0.0121
8	103.0502	8517	1.4	110	0.7	0.0121
9	119.0481	9839	78.6	6181	39.3	0.0121
10	147.0434	12153	199.8	15713	100.0	0.0121

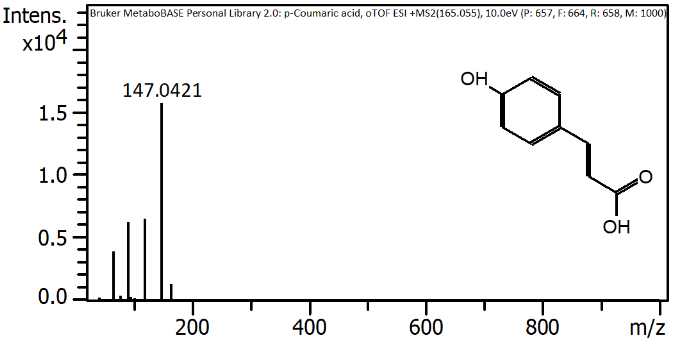

#	m/z	Res.	S/N	I	I %	FWHM
1	65.0376	5375	3.7	409	2.6	0.0121
2	65.0395	5375	35.7	3932	25.0	0.0121
3	91.0513	7525	7.7	849	5.4	0.0121
4	91.0545	7526	56.9	6260	39.8	0.0121
5	119.0489	9839	59.4	6543	41.6	0.0121
6	119.0503	9839	18.4	2029	12.9	0.0121
7	147.0421	12153	142.7	15713	100.0	0.0121
8	147.0455	12153	32.3	3555	22.6	0.0121
9	164.9162	13630	11.6	1274	8.1	0.0121
10	165.0509	13641	6.1	676	4.3	0.0121

Cmpd 591, AutoMSn(355.1031), 7.08 min

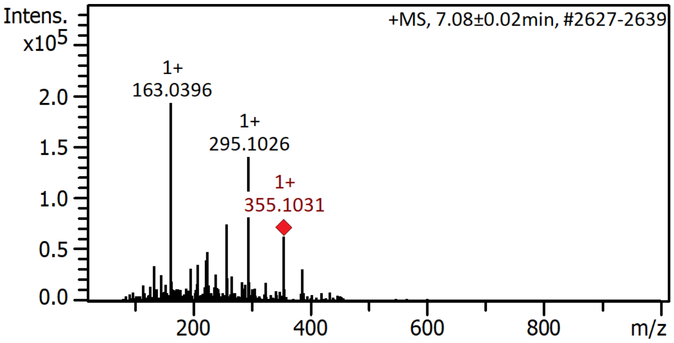

#	m/z	Res.	S/N	I	I %	FWHM
1	133.0506	9696	2829.3	33952	17.6	0.0137
2	163.0396	10491	16057.8	192693	100.0	0.0155
3	197.1173	10833	2630.8	31569	16.4	0.0182
4	209.0444	11655	2943.4	35320	18.3	0.0179
5	223.1331	11682	3335.8	40029	20.8	0.0191
6	225.1489	11776	4048.3	48580	25.2	0.0191
7	258.1703	11698	6246.7	74960	38.9	0.0221
8	295.1026	11985	11691.1	140293	72.8	0.0246
9	355.1031	12667	5262.4	63149	32.8	0.0280
10	387.2017	12441	2571.7	30860	16.0	0.0311

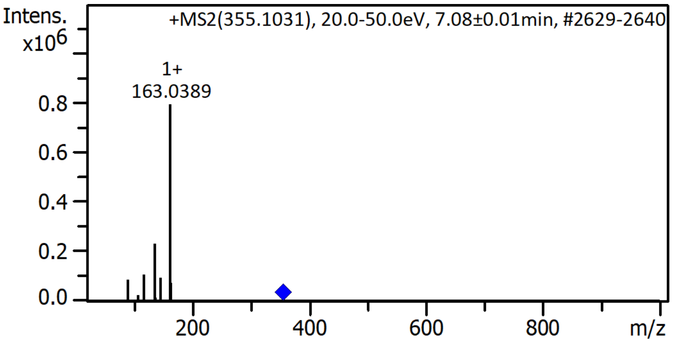

#	m/z	Res.	S/N	I	I %	FWHM
1	89.0384	8732	5475.9	85789	10.8	0.0102
2	107.0486	9506	1541.0	24143	3.0	0.0113
3	117.0332	9816	6901.4	108123	13.6	0.0119
4	118.0366	10159	415.4	6508	0.8	0.0116
5	135.0436	9998	14813.8	232083	29.3	0.0135
6	136.0472	10374	979.2	15340	1.9	0.0131
7	145.0282	10501	6006.9	94107	11.9	0.0138
8	146.0316	10428	446.7	6998	0.9	0.0140
9	163.0389	9417	50563.4	792160	100.0	0.0173
10	164.0420	10913	4738.8	74241	9.4	0.0150

Compound Spectrum List Report

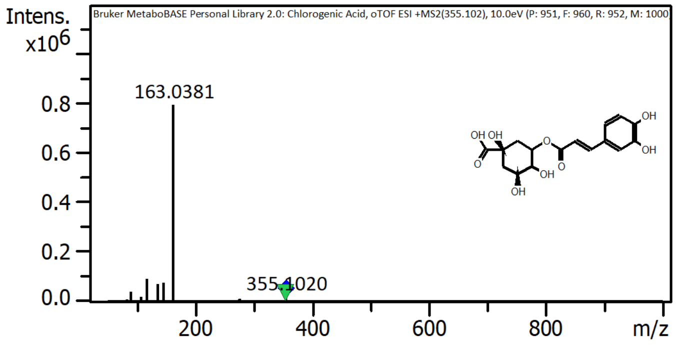

#	m/z	Res.	S/N	I	I %	FWHM
1	83.0488	4918	2.0	9506	1.2	0.0169
2	89.0390	5272	8.5	40400	5.1	0.0169
3	107.0477	6339	4.3	20596	2.6	0.0169
4	117.0328	6930	19.2	91098	11.5	0.0169
5	135.0432	7996	14.8	70502	8.9	0.0169
6	145.0278	8587	16.2	76840	9.7	0.0169
7	145.0293	8588	2.3	11090	1.4	0.0169
8	163.0381	9654	166.5	791368	100.0	0.0169
9	276.0422	16345	2.7	12675	1.6	0.0169
10	355.1020	21026	4.3	20596	2.6	0.0169

Cmpd 615, AutoMSn(195.0653), 7.34 min

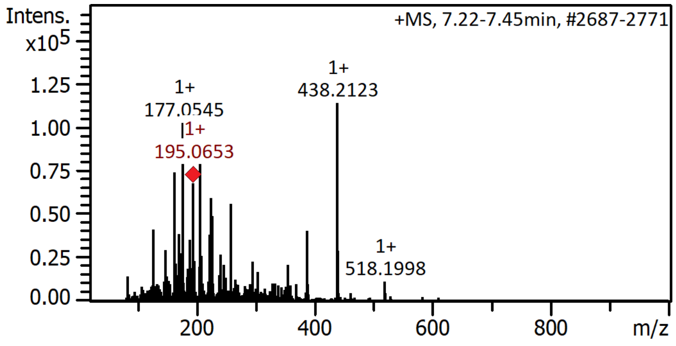

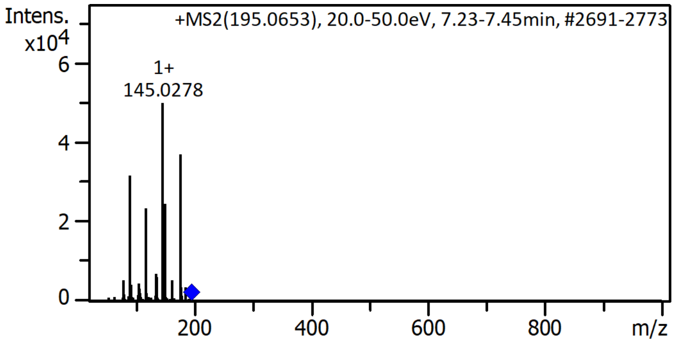

#	m/z	Res.	S/N	I	I %	FWHM
1	126.0911	10254	6894.9	41369	36.3	0.0123
2	163.0396	10661	12334.0	74004	65.0	0.0153
3	177.0545	11145	17049.3	102296	89.9	0.0159
4	195.0653	11644	11284.4	67706	59.5	0.0168
5	207.1381	11549	14844.0	89064	78.3	0.0179
6	225.1486	11780	9922.0	59532	52.3	0.0191
7	227.1281	11616	8189.7	49138	43.2	0.0196
8	258.1696	11848	9342.7	56056	49.3	0.0218
9	387.2012	12660	6795.4	40772	35.8	0.0306
10	438.2123	12696	18969.6	113818	100.0	0.0345

#	m/z	Res.	S/N	I	I %	FWHM
1	78.0464	8194	693.0	5197	10.4	0.0095
2	89.0382	8854	4214.8	31611	63.5	0.0101
3	105.0342	8340	600.6	4504	9.0	0.0126
4	117.0330	9783	3119.6	23397	47.0	0.0120
5	134.0354	10636	913.1	6848	13.8	0.0126
6	135.0431	10947	797.4	5981	12.0	0.0123
7	145.0278	10809	6638.7	49790	100.0	0.0134
8	149.0591	10746	3281.4	24610	49.4	0.0139
9	163.0385	10547	693.8	5204	10.5	0.0155
10	177.0540	11359	4924.2	36932	74.2	0.0156

Compound Spectrum List Report

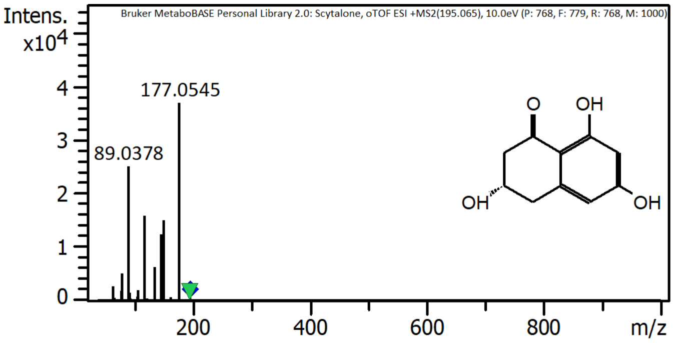

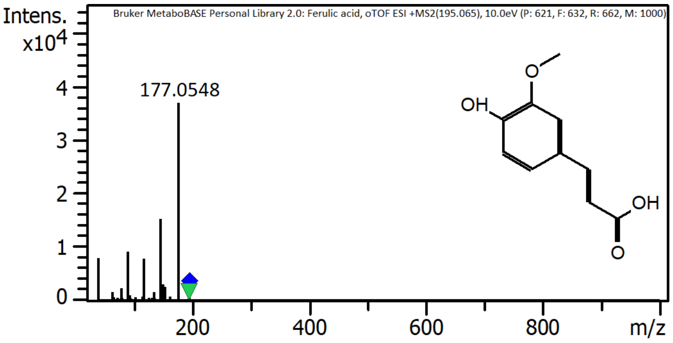

#	m/z	Res.	S/N	I	I %	FWHM
1	63.0231	4480	14.8	2733	7.4	0.0141
2	78.0455	5548	27.4	5060	13.7	0.0141
3	89.0378	6330	136.0	25114	68.1	0.0141
4	117.0319	8320	86.2	15918	43.1	0.0141
5	134.0353	9529	34.2	6315	17.1	0.0141
6	145.0283	10310	27.8	5134	13.9	0.0141
7	145.0285	10310	67.2	12409	33.6	0.0141
8	149.0569	10597	81.6	15068	40.8	0.0141
9	177.0524	12587	65.4	12077	32.7	0.0141
10	177.0545	12587	199.8	36895	100.0	0.0141

#	m/z	Res.	S/N	I	I %	FWHM
1	38.9646	2770	43.8	8088	21.9	0.0141
2	89.0394	6330	17.2	3176	8.6	0.0141
3	89.0410	6330	50.0	9233	25.0	0.0141
4	116.9778	8316	37.8	6980	18.9	0.0141
5	117.0344	8320	43.0	7940	21.5	0.0141
6	145.0280	10310	83.2	15364	41.6	0.0141
7	145.0308	10310	41.8	7719	20.9	0.0141
8	149.0589	10597	16.2	2991	8.1	0.0141
9	177.0542	12587	21.0	3878	10.5	0.0141
10	177.0548	12587	199.8	36895	100.0	0.0141

Cmpd 623, AutoMSn(165.0541), 7.46 min

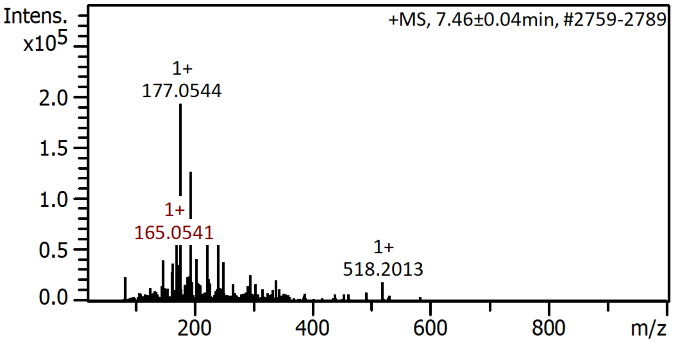

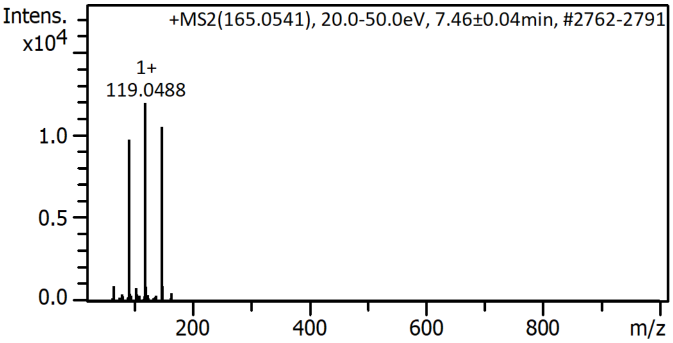

#	m/z	Res.	S/N	I	I %	FWHM
1	147.0439	10855	5604.8	40354	20.9	0.0135
2	165.0541	11015	5070.7	36509	18.9	0.0150
3	171.1013	10755	8053.1	57983	30.0	0.0159
4	175.0387	10971	4949.9	35639	18.5	0.0160
5	177.0544	11084	26812.3	193048	100.0	0.0160
6	195.0649	11550	17621.4	126874	65.7	0.0169
7	205.1220	11453	5799.3	41755	21.6	0.0179
8	223.1327	11514	10122.4	72881	37.8	0.0194
9	241.1429	12111	7695.8	55410	28.7	0.0199
10	250.0896	12027	5361.1	38600	20.0	0.0208

#	m/z	Res.	S/N	I	I %	FWHM
1	65.0390	6070	93.9	901	7.6	0.0107
2	91.0540	8904	1013.4	9728	81.7	0.0102
3	92.0561	9182	87.3	838	7.0	0.0100
4	103.0531	10794	84.0	807	6.8	0.0095
5	104.0566	9903	58.6	563	4.7	0.0105
6	119.0488	10095	1240.3	11906	100.0	0.0118
7	120.0538	9906	89.4	858	7.2	0.0121
8	120.0790	10164	88.8	852	7.2	0.0118
9	147.0442	10879	1094.4	10506	88.2	0.0135
10	148.0466	11241	92.5	888	7.5	0.0132

Compound Spectrum List Report

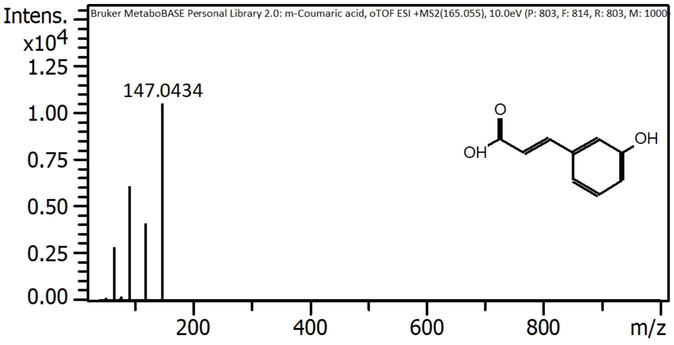

#	m/z	Res.	S/N	I	I %	FWHM
1	41.0374	3437	1.8	95	0.9	0.0119
2	51.0216	4273	2.6	137	1.3	0.0119
3	65.0383	5447	54.8	2879	27.4	0.0119
4	75.0213	6283	2.4	126	1.2	0.0119
5	77.0383	6452	4.2	221	2.1	0.0119
6	91.0538	7626	116.4	6115	58.3	0.0119
7	101.0376	8462	1.8	95	0.9	0.0119
8	103.0502	8631	1.4	74	0.7	0.0119
9	119.0481	9971	78.6	4129	39.3	0.0119
10	147.0434	12316	199.8	10496	100.0	0.0119

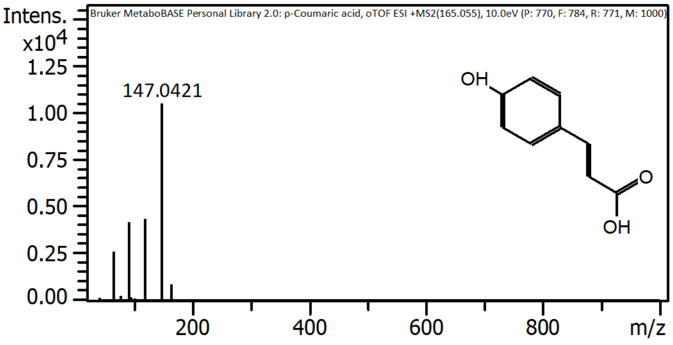

#	m/z	Res.	S/N	I	I %	FWHM
1	65.0376	5447	3.7	273	2.6	0.0119
2	65.0395	5447	35.7	2627	25.0	0.0119
3	91.0513	7626	7.7	567	5.4	0.0119
4	91.0545	7626	56.9	4181	39.8	0.0119
5	119.0489	9971	59.4	4371	41.6	0.0119
6	119.0503	9971	18.4	1355	12.9	0.0119
7	147.0421	12316	142.7	10496	100.0	0.0119
8	147.0455	12316	32.3	2374	22.6	0.0119
9	164.9162	13813	11.6	851	8.1	0.0119
10	165.0509	13824	6.1	452	4.3	0.0119

Cmpd 631, AutoMSn(195.0652), 7.53 min

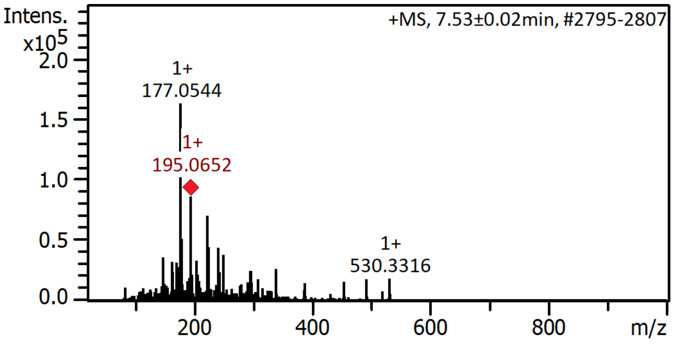

#	m/z	Res.	S/N	I	I %	FWHM
1	147.0434	10948	3005.3	36063	22.1	0.0134
2	163.0441	8623	2698.2	32378	19.9	0.0189
3	177.0544	11286	13582.4	162989	100.0	0.0157
4	180.1020	11168	4274.4	51293	31.5	0.0161
5	195.0652	11278	7174.4	86093	52.8	0.0173
6	205.1222	10763	2807.9	33695	20.7	0.0191
7	223.1334	11720	5867.9	70415	43.2	0.0190
8	225.1479	12053	3733.1	44798	27.5	0.0187
9	241.1438	11307	3656.4	43877	26.9	0.0213
10	250.0897	11041	3184.8	38218	23.4	0.0227

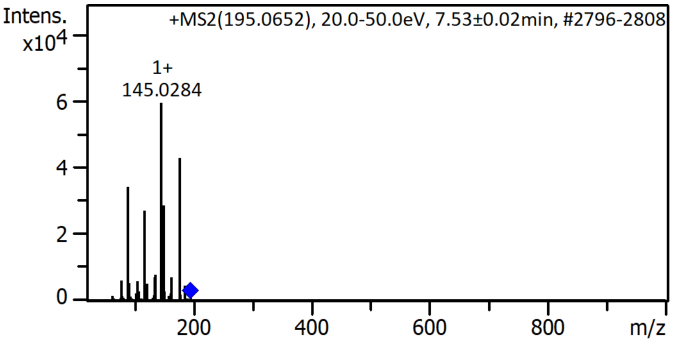

#	m/z	Res.	S/N	I	I %	FWHM
1	78.0467	8072	387.6	6073	10.2	0.0097
2	89.0385	8893	2188.8	34292	57.7	0.0100
3	105.0342	8275	370.6	5805	9.8	0.0127
4	117.0332	10134	1739.8	27257	45.9	0.0115
5	134.0364	10537	445.0	6972	11.7	0.0127
6	135.0441	10907	491.5	7700	13.0	0.0124
7	145.0284	10764	3792.4	59414	100.0	0.0135
8	149.0599	11173	1832.8	28713	48.3	0.0133
9	163.0394	11342	452.9	7095	11.9	0.0144
10	177.0546	11548	2742.1	42960	72.3	0.0153

Compound Spectrum List Report

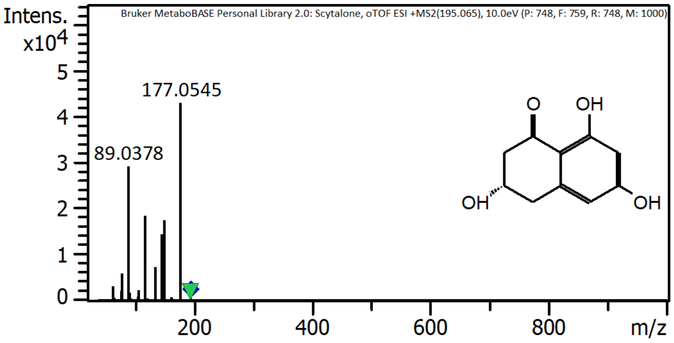

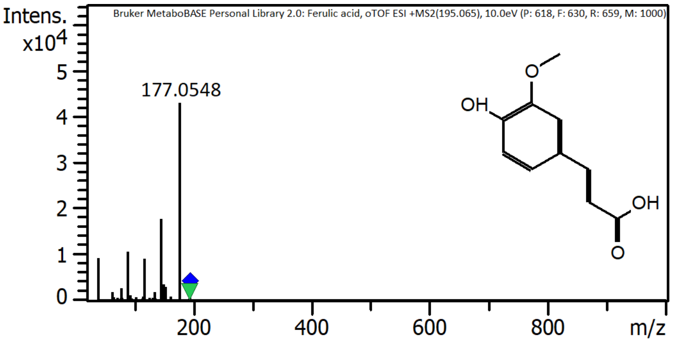

#	m/z	Res.	S/N	I	I %	FWHM
1	63.0231	4510	14.8	3179	7.4	0.0140
2	78.0455	5585	27.4	5886	13.7	0.0140
3	89.0378	6371	136.0	29213	68.1	0.0140
4	117.0319	8374	86.2	18516	43.1	0.0140
5	134.0353	9591	34.2	7346	17.1	0.0140
6	145.0283	10378	27.8	5971	13.9	0.0140
7	145.0285	10378	67.2	14435	33.6	0.0140
8	149.0569	10666	81.6	17528	40.8	0.0140
9	177.0524	12669	65.4	14048	32.7	0.0140
10	177.0545	12669	199.8	42917	100.0	0.0140

#	m/z	Res.	S/N	I	I %	FWHM
1	38.9646	2788	43.8	9408	21.9	0.0140
2	89.0394	6371	17.2	3695	8.6	0.0140
3	89.0410	6371	50.0	10740	25.0	0.0140
4	116.9778	8370	37.8	8119	18.9	0.0140
5	117.0344	8374	43.0	9236	21.5	0.0140
6	145.0280	10378	83.2	17871	41.6	0.0140
7	145.0308	10378	41.8	8979	20.9	0.0140
8	149.0589	10666	16.2	3480	8.1	0.0140
9	177.0542	12669	21.0	4511	10.5	0.0140
10	177.0548	12669	199.8	42917	100.0	0.0140

Cmpd 648, AutoMSn(153.1267), 7.73 min

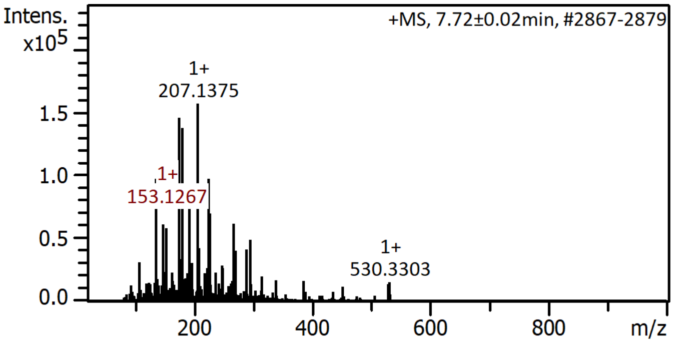

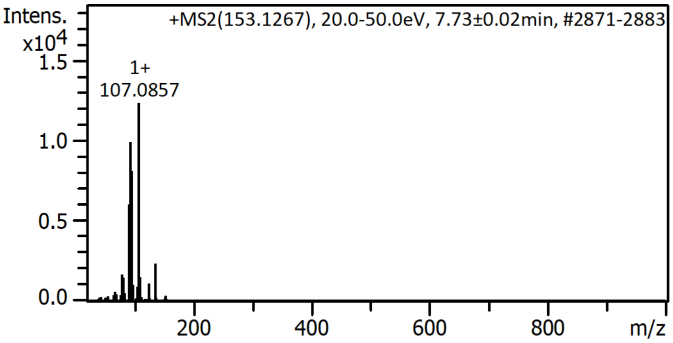

#	m/z	Res.	S/N	I	I %	FWHM
1	135.1163	10294	8125.0	97500	62.1	0.0131
2	147.0438	10642	5105.2	61262	39.0	0.0138
3	153.1267	10683	4841.4	58097	37.0	0.0143
4	175.0386	10795	12159.9	145919	92.9	0.0162
5	181.1217	10677	11480.5	137766	87.7	0.0170
6	193.0488	11630	6829.6	81956	52.2	0.0166
7	207.1375	11077	13085.4	157025	100.0	0.0187
8	225.1479	11293	8134.7	97616	62.2	0.0199
9	227.1276	11382	5826.1	69913	44.5	0.0200
10	267.0318	11869	5145.5	61746	39.3	0.0225

#	m/z	Res.	S/N	I	I %	FWHM
1	79.0543	8359	102.1	1634	13.3	0.0095
2	81.0690	9111	90.9	1454	11.8	0.0089
3	91.0541	9096	375.9	6014	48.8	0.0100
4	93.0697	9344	619.4	9911	80.4	0.0100
5	95.0850	9541	507.6	8121	65.9	0.0100
6	97.0645	7822	63.9	1022	8.3	0.0124
7	107.0857	9695	770.2	12323	100.0	0.0110
8	109.0993	11144	94.2	1507	12.2	0.0098
9	124.0411	9825	67.9	1086	8.8	0.0126
10	135.1160	9989	144.9	2319	18.8	0.0135

Compound Spectrum List Report

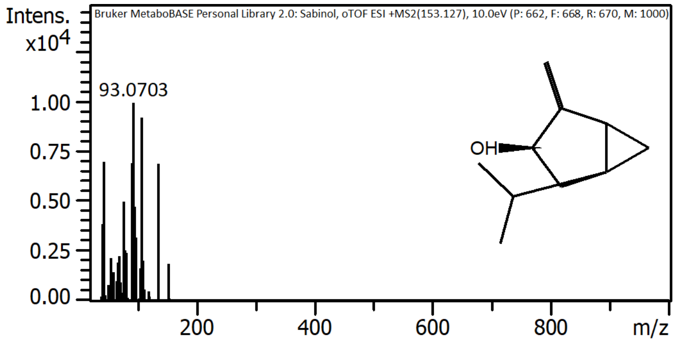

#	m/z	Res.	S/N	I	I %	FWHM
1	41.0394	3656	77.6	3845	38.8	0.0112
2	43.0552	3836	140.6	6967	70.4	0.0112
3	77.0387	6863	100.6	4985	50.4	0.0112
4	79.0545	7042	51.2	2537	25.6	0.0112
5	91.0541	8111	139.2	6898	69.7	0.0112
6	93.0703	8291	199.8	9901	100.0	0.0112
7	95.0852	8471	95.6	4737	47.8	0.0112
8	97.0647	8647	64.4	3191	32.2	0.0112
9	107.0848	9539	185.0	9167	92.6	0.0112
10	135.1154	12037	138.4	6858	69.3	0.0112

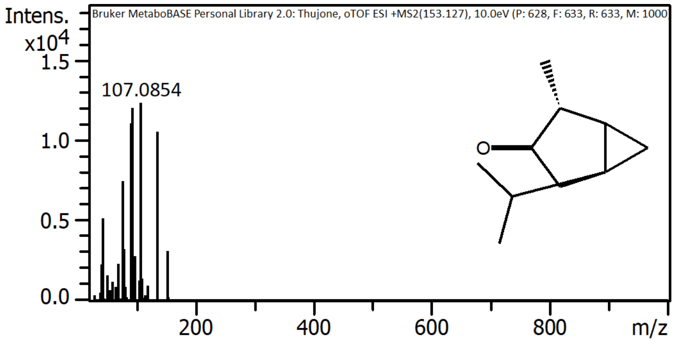

#	m/z	Res.	S/N	I	I %	FWHM
1	43.0183	3832	25.8	2859	23.2	0.0112
2	43.0554	3836	46.4	5151	41.8	0.0112
3	77.0392	6863	67.3	7468	60.7	0.0112
4	79.0547	7042	29.3	3253	26.4	0.0112
5	91.0543	8111	99.7	11054	89.8	0.0112
6	93.0699	8291	108.2	12003	97.5	0.0112
7	97.0654	8647	25.4	2822	22.9	0.0112
8	107.0854	9540	111.0	12311	100.0	0.0112
9	135.1174	12037	95.1	10549	85.7	0.0112
10	153.1277	13641	28.2	3130	25.4	0.0112

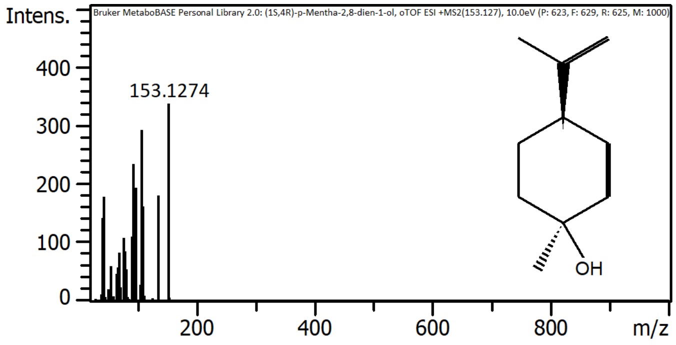

#	m/z	Res.	S/N	I	I %	FWHM
1	41.0397	3656	84.2	142	42.1	0.0112
2	43.0186	3832	105.8	179	53.0	0.0112
3	93.0699	8291	138.8	235	69.5	0.0112
4	95.0853	8471	103.0	174	51.6	0.0112
5	95.0854	8471	101.2	171	50.7	0.0112
6	97.0648	8647	114.6	194	57.4	0.0112
7	107.0856	9540	173.2	293	86.7	0.0112
8	109.1015	9719	95.8	162	47.9	0.0112
9	135.1169	12037	106.8	180	53.5	0.0112
10	153.1274	13641	199.8	338	100.0	0.0112

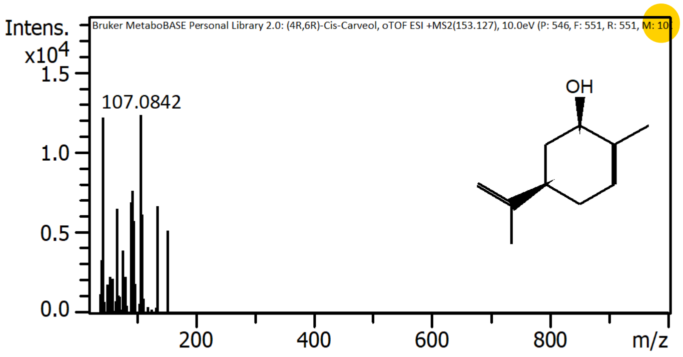

#	m/z	Res.	S/N	I	I %	FWHM
1	43.0180	3832	105.6	10413	84.6	0.0112
2	43.0181	3832	123.4	12163	98.8	0.0112
3	67.0537	5973	65.9	6494	52.8	0.0112
4	91.0537	8111	70.3	6926	56.3	0.0112
5	93.0688	8291	77.3	7616	61.9	0.0112
6	95.0838	8470	58.2	5743	46.6	0.0112
7	107.0842	9539	124.9	12311	100.0	0.0112
8	109.1001	9719	62.4	6149	49.9	0.0112
9	135.1153	12037	67.6	6667	54.2	0.0112
10	153.1251	13641	52.3	5151	41.8	0.0112

Cmpd 649, AutoMSn(135.1164), 7.75 min

Compound Spectrum List Report

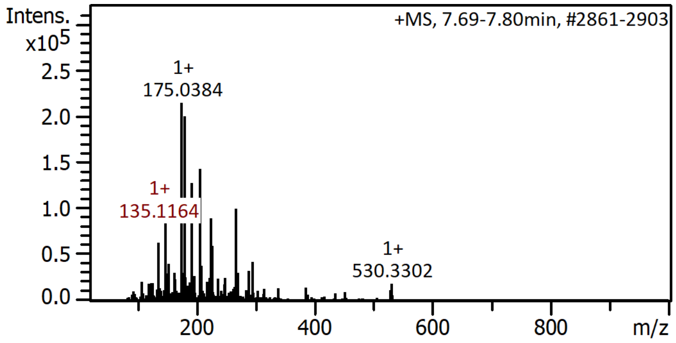

#	m/z	Res.	S/N	I	I %	FWHM
1	135.1164	10134	8822.4	63521	29.7	0.0133
2	147.0436	10812	12330.8	88782	41.5	0.0136
3	175.0384	10596	29731.7	214068	100.0	0.0165
4	181.1217	10287	27797.1	200139	93.5	0.0176
5	193.0491	11126	17748.3	127788	59.7	0.0174
6	207.1376	11295	19851.2	142929	66.8	0.0183
7	225.1482	11393	12479.1	89850	42.0	0.0198
8	227.1276	11545	8355.6	60161	28.1	0.0197
9	267.0316	11666	13863.1	99814	46.6	0.0229
10	295.1020	12245	5899.8	42479	19.8	0.0241

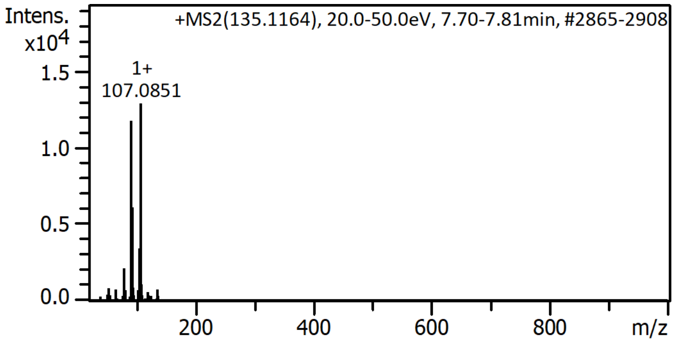

#	m/z	Res.	S/N	I	I %	FWHM
1	53.0401	4942	88.8	835	6.5	0.0107
2	79.0541	8333	222.4	2090	16.2	0.0095
3	91.0539	9046	1249.2	11743	91.1	0.0101
4	92.0591	9371	95.3	896	7.0	0.0098
5	93.0698	9029	650.5	6115	47.4	0.0103
6	94.0742	7806	91.4	859	6.7	0.0121
7	105.0699	10360	365.4	3435	26.7	0.0101
8	107.0851	9475	1371.0	12887	100.0	0.0113
9	108.0897	10559	112.2	1055	8.2	0.0102
10	135.1154	10452	76.6	720	5.6	0.0129

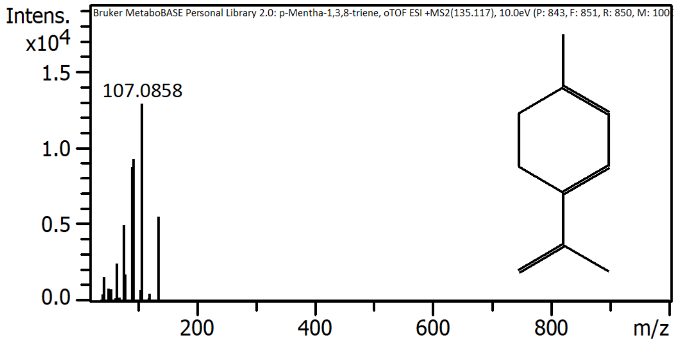

#	m/z	Res.	S/N	I	I %	FWHM
1	43.0556	3706	24.6	1585	12.3	0.0116
2	51.0243	4391	12.8	825	6.4	0.0116
3	65.0401	5598	38.4	2474	19.2	0.0116
4	77.0394	6630	77.6	5000	38.8	0.0116
5	79.0540	6804	26.6	1714	13.3	0.0116
6	79.0545	6804	20.4	1314	10.2	0.0116
7	91.0549	7837	136.0	8763	68.1	0.0116
8	93.0709	8010	144.2	9292	72.2	0.0116
9	107.0858	9216	199.8	12874	100.0	0.0116
10	135.1169	11629	86.2	5554	43.1	0.0116

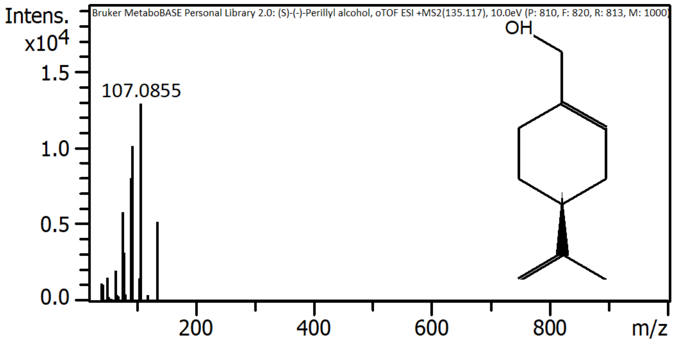

#	m/z	Res.	S/N	I	I %	FWHM
1	41.0395	3532	11.3	1160	9.0	0.0116
2	51.0237	4391	14.8	1521	11.8	0.0116
3	65.0391	5598	19.3	1985	15.4	0.0116
4	77.0390	6630	56.4	5812	45.1	0.0116
5	79.0547	6804	31.1	3209	24.9	0.0116
6	91.0542	7837	78.1	8055	62.6	0.0116
7	93.0700	8010	98.4	10142	78.8	0.0116
8	105.0694	9043	14.3	1469	11.4	0.0116
9	107.0855	9216	124.9	12874	100.0	0.0116
10	135.1164	11629	50.3	5181	40.2	0.0116

Cmpd 653, AutoMSn(209.0443), 7.80 min

Compound Spectrum List Report

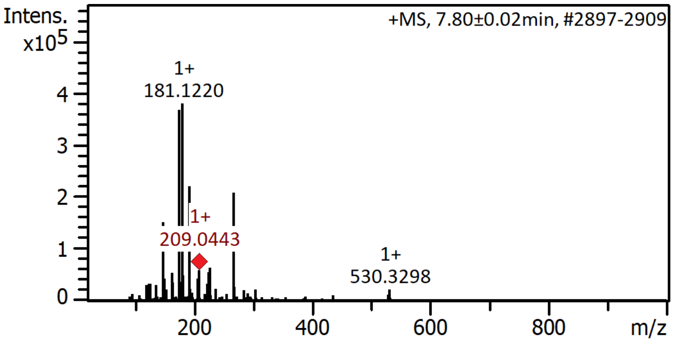

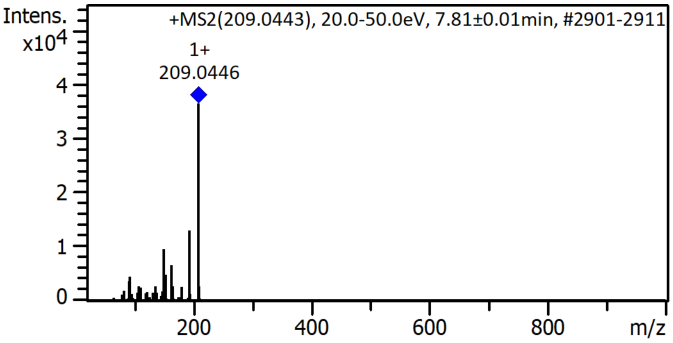

#	m/z	Res.	S/N	I	I %	FWHM
1	147.0439	10784	12634.0	151608	39.8	0.0136
2	163.1113	10932	4442.9	53315	14.0	0.0149
3	175.0386	10670	30680.7	368168	96.7	0.0164
4	181.1220	10433	31711.4	380537	100.0	0.0174
5	182.1252	11279	4021.6	48259	12.7	0.0161
6	193.0493	11101	18461.8	221542	58.2	0.0174
7	209.0443	11362	4921.5	59058	15.5	0.0184
8	225.1487	11347	4559.8	54717	14.4	0.0198
9	227.1283	11699	5266.8	63202	16.6	0.0194
10	267.0320	11748	17402.6	208831	54.9	0.0227

#	m/z	Res.	S/N	I	I %	FWHM
1	91.0540	9043	230.1	3606	9.9	0.0101
2	92.0256	8648	281.9	4417	12.1	0.0106
3	107.0494	10778	167.2	2619	7.2	0.0099
4	135.0435	11066	171.7	2690	7.4	0.0122
5	149.0235	11064	614.7	9630	26.4	0.0135
6	153.0545	9560	303.8	4760	13.0	0.0160
7	163.0381	10540	421.1	6598	18.1	0.0155
8	194.0197	11546	830.1	13004	35.6	0.0168
9	209.0446	11673	2330.9	36517	100.0	0.0179
10	210.0480	11001	172.4	2701	7.4	0.0191

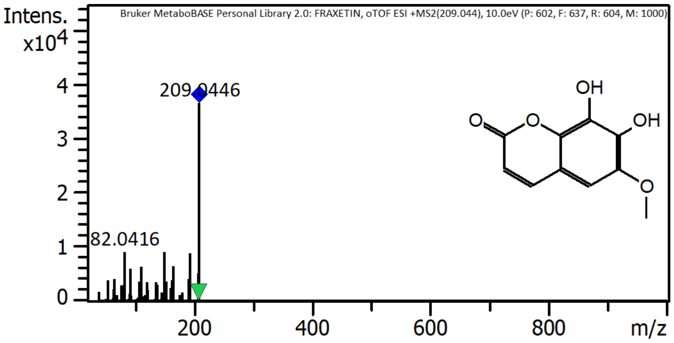

#	m/z	Res.	S/N	I	I %	FWHM
1	65.0396	3361	22.2	4053	11.1	0.0194
2	82.0416	4240	50.0	9129	25.0	0.0194
3	92.0266	4756	32.8	5989	16.4	0.0194
4	110.0360	5686	34.4	6281	17.2	0.0194
5	149.0222	7701	50.0	9129	25.0	0.0194
6	166.0276	8580	35.0	6391	17.5	0.0194
7	194.0198	10026	49.0	8947	24.5	0.0194
8	208.1358	10756	28.4	5185	14.2	0.0194
9	209.0437	10803	83.2	15191	41.6	0.0194
10	209.0446	10803	199.8	36481	100.0	0.0194

Cmpd 659, AutoMSn(355.1023), 7.85 min

Compound Spectrum List Report

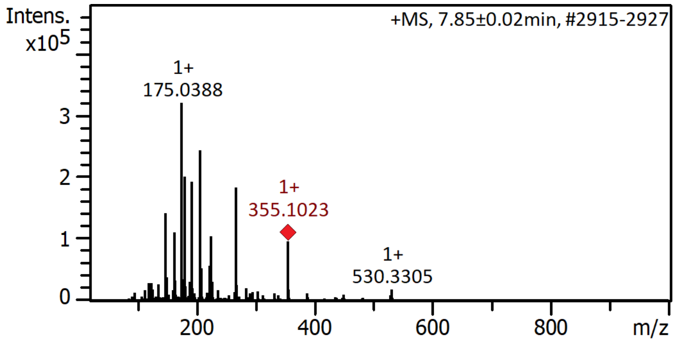

#	m/z	Res.	S/N	I	I %	FWHM
1	147.0438	10463	11837.6	142051	44.4	0.0141
2	163.0389	10867	9281.5	111378	34.8	0.0150
3	175.0388	10679	26642.1	319706	100.0	0.0164
4	181.1221	10975	16788.6	201463	63.0	0.0165
5	193.0493	11233	16114.9	193379	60.5	0.0172
6	207.1380	11273	20315.9	243791	76.3	0.0184
7	223.1323	11916	4715.0	56580	17.7	0.0187
8	225.1483	11920	8779.8	105357	33.0	0.0189
9	267.0317	12249	15327.8	183934	57.5	0.0218
10	355.1023	11739	8047.9	96575	30.2	0.0303

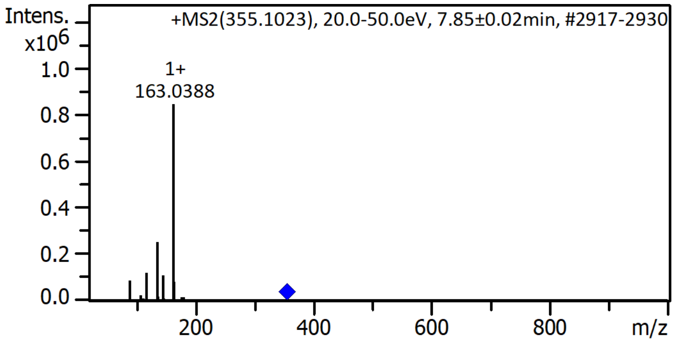

#	m/z	Res.	S/N	I	I %	FWHM
1	89.0384	8867	5720.2	87709	10.4	0.0100
2	107.0489	9595	1506.5	23099	2.7	0.0112
3	117.0333	9747	7746.6	118782	14.1	0.0120
4	135.0439	9811	16604.2	254598	30.2	0.0138
5	136.0477	10719	1182.5	18132	2.1	0.0127
6	145.0283	10485	7052.1	108133	12.8	0.0138
7	163.0388	9538	55051.8	844127	100.0	0.0171
8	164.0422	10780	5250.7	80510	9.5	0.0152
9	177.0543	11379	914.8	14026	1.7	0.0156
10	181.0493	11428	919.0	14091	1.7	0.0158

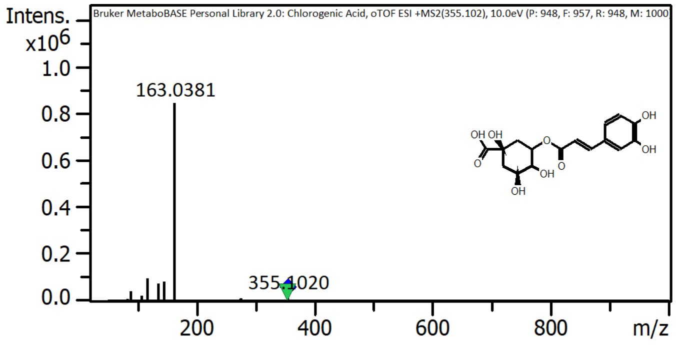

#	m/z	Res.	S/N	I	I %	FWHM
1	83.0488	4939	2.0	10130	1.2	0.0168
2	89.0390	5296	8.5	43050	5.1	0.0168
3	107.0477	6367	4.3	21947	2.6	0.0168
4	117.0328	6961	19.2	97075	11.5	0.0168
5	135.0432	8032	14.8	75127	8.9	0.0168
6	145.0278	8626	16.2	81880	9.7	0.0168
7	145.0293	8626	2.3	11818	1.4	0.0168
8	163.0381	9697	166.5	843283	100.0	0.0168
9	276.0422	16418	2.7	13506	1.6	0.0168
10	355.1020	21120	4.3	21947	2.6	0.0168

Cmpd 671, AutoMSn(135.1164), 7.99 min

Compound Spectrum List Report

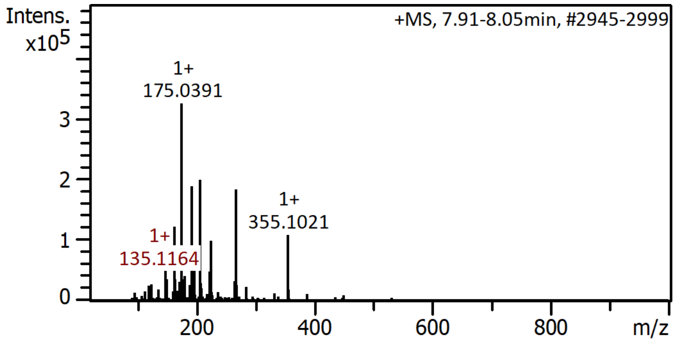

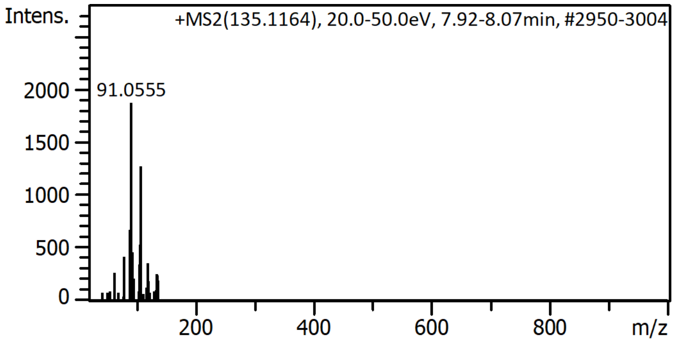

#	m/z	Res.	S/N	I	I %	FWHM
1	147.0442	10363	6999.9	125998	38.7	0.0142
2	163.0389	10886	6837.9	123082	37.8	0.0150
3	175.0391	10515	18094.8	325706	100.0	0.0166
4	193.0497	10874	10526.9	189485	58.2	0.0178
5	197.1175	11724	3346.0	60228	18.5	0.0168
6	207.1377	10810	11098.9	199780	61.3	0.0192
7	223.1334	11942	2686.3	48353	14.8	0.0187
8	225.1483	11468	5540.8	99735	30.6	0.0196
9	267.0324	12174	10202.9	183652	56.4	0.0219
10	355.1021	12808	6044.3	108797	33.4	0.0277

#	m/z	Res.	S/N	I	I %	FWHM
1	63.0248	5091	6.6	264	14.1	0.0124
2	79.0550	11465	10.5	420	22.5	0.0069
3	89.0381	9809	16.9	676	36.2	0.0091
4	91.0555	9397	46.7	1869	100.0	0.0097
5	93.0684	12165	11.6	465	24.9	0.0077
6	105.0392	9269	8.5	341	18.2	0.0113
7	106.0665	5393	13.3	532	28.4	0.0197
8	107.0853	6973	31.7	1269	67.9	0.0154
9	119.0407	9910	8.4	338	18.1	0.0120
10	119.0829	5431	8.9	358	19.2	0.0219

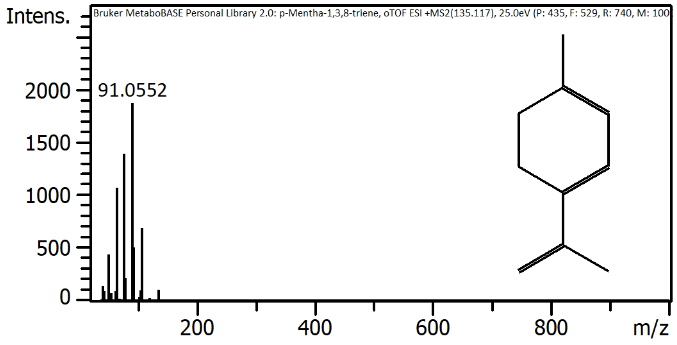

#	m/z	Res.	S/N	I	I %	FWHM
1	41.0396	4320	15.0	140	7.5	0.0095
2	51.0236	5371	47.4	443	23.7	0.0095
3	65.0402	6847	115.0	1074	57.6	0.0095
4	77.0392	8110	148.8	1390	74.5	0.0095
5	78.0450	8216	12.6	118	6.3	0.0095
6	79.0516	8322	23.0	215	11.5	0.0095
7	79.0546	8322	11.8	110	5.9	0.0095
8	91.0552	9586	199.8	1867	100.0	0.0095
9	93.0708	9798	54.4	508	27.2	0.0095
10	107.0859	11273	74.0	691	37.0	0.0095

Cmpd 684, AutoMSn(181.1229), 8.16 min

Compound Spectrum List Report

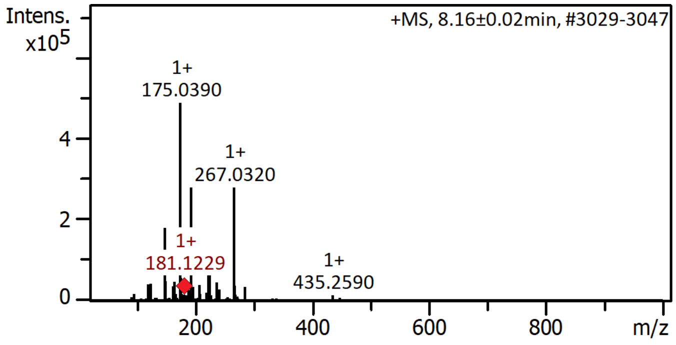

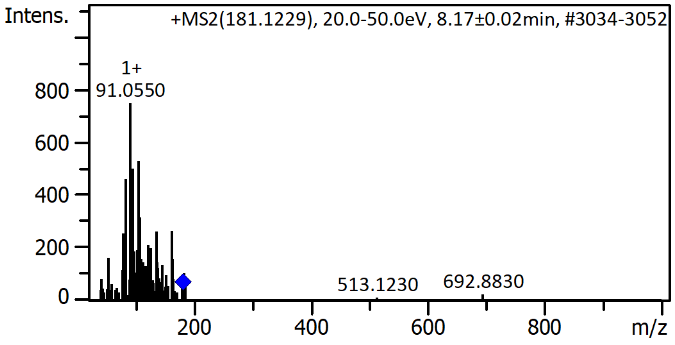

#	m/z	Res.	S/N	I	I %	FWHM
1	147.0439	10741	20084.1	180757	37.1	0.0137
2	149.0594	10583	5374.2	48368	9.9	0.0141
3	165.0545	11016	5123.0	46107	9.5	0.0150
4	175.0390	10360	54168.7	487518	100.0	0.0169
5	176.0425	11255	6175.9	55583	11.4	0.0156
6	193.0495	10808	30998.7	278988	57.2	0.0179
7	223.1330	11467	8678.1	78103	16.0	0.0195
8	225.1484	11262	8367.7	75310	15.4	0.0200
9	237.1851	11325	5001.7	45015	9.2	0.0209
10	267.0320	12144	31125.1	280126	57.5	0.0220

#	m/z	Res.	S/N	I	I %	FWHM
1	79.0545	8625	21.4	256	34.2	0.0092
2	83.0492	9520	38.5	462	61.8	0.0087
3	91.0550	10522	62.4	748	100.0	0.0087
4	93.0690	10992	34.5	414	55.3	0.0085
5	95.0850	10995	41.8	501	67.0	0.0086
6	105.0696	12096	44.1	530	70.8	0.0087
7	107.0467	12712	18.4	221	29.5	0.0084
8	107.0844	8642	26.3	316	42.2	0.0124
9	135.1133	14008	21.9	263	35.1	0.0096
10	162.0908	13742	22.0	265	35.3	0.0118

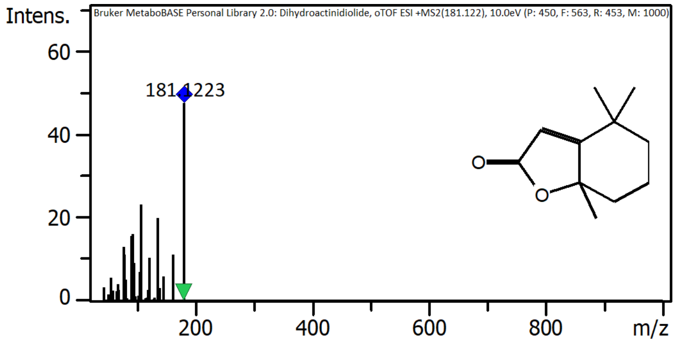

#	m/z	Res.	S/N	I	I %	FWHM
1	77.0390	10757	46.0	13	27.6	0.0072
2	79.0547	11039	39.7	11	23.8	0.0072
3	91.0545	12714	55.2	16	33.1	0.0072
4	93.0701	12996	57.0	16	34.2	0.0072
5	95.0855	13277	32.3	9	19.4	0.0072
6	107.0854	14953	81.8	23	49.1	0.0072
7	121.1003	16910	36.8	10	22.1	0.0072
8	135.1165	18867	70.5	20	42.3	0.0072
9	163.1114	22776	39.5	11	23.7	0.0072
10	181.1223	25291	166.5	47	100.0	0.0072

Cmpd 713, AutoMSn(107.0852), 8.51 min

Compound Spectrum List Report

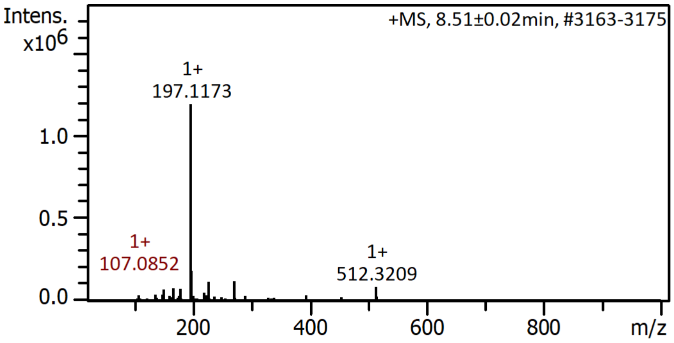

#	m/z	Res.	S/N	I	I %	FWHM
1	149.1324	10488	5653.6	67843	5.7	0.0142
2	167.1430	11138	6367.2	76407	6.4	0.0150
3	179.0707	11097	5990.0	71880	6.0	0.0161
4	179.1061	11153	6097.9	73175	6.1	0.0161
5	197.1173	10397	99386.1	1192634	100.0	0.0190
6	198.1212	11023	14990.3	179883	15.1	0.0180
7	219.1000	11020	4126.4	49516	4.2	0.0199
8	227.0737	11595	9541.3	114496	9.6	0.0196
9	271.0638	11949	9987.9	119855	10.0	0.0227
10	512.3209	13729	7038.4	84461	7.1	0.0373

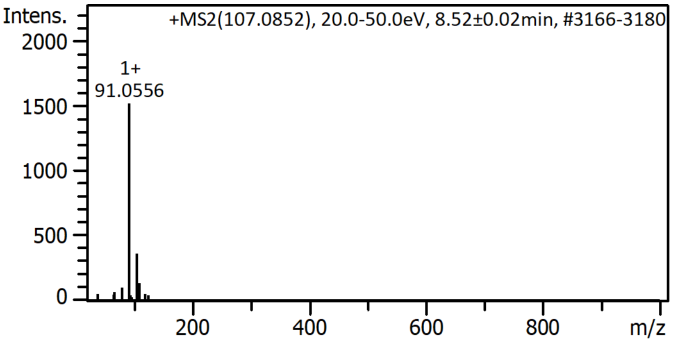

#	m/z	Res.	S/N	I	I %	FWHM
1	37.9927	8348	1.9	50	3.3	0.0046
2	66.0291	10427	2.5	65	4.3	0.0063
3	79.0529	11986	3.8	101	6.7	0.0066
4	91.0556	7608	57.6	1517	100.0	0.0120
5	92.0600	10259	7.7	203	13.4	0.0090
6	105.0697	10574	13.9	365	24.1	0.0099
7	106.0717	13688	2.2	57	3.7	0.0077
8	107.0902	13217	4.4	117	7.7	0.0081
9	109.0712	11735	5.2	136	8.9	0.0093
10	119.0606	14711	1.9	51	3.4	0.0081

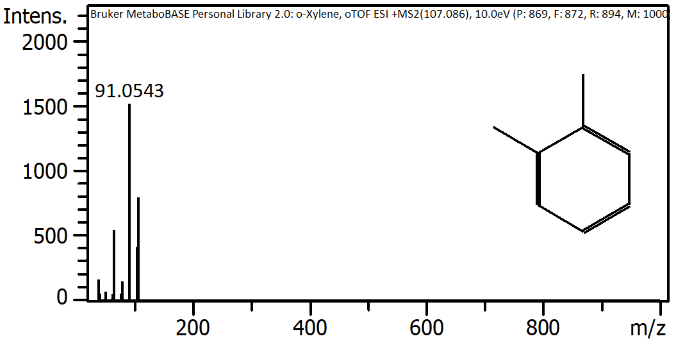

#	m/z	Res.	S/N	I	I %	FWHM
1	39.0237	3929	20.4	155	10.2	0.0099
2	39.0238	3929	21.8	165	10.9	0.0099
3	65.0393	6549	63.6	482	31.8	0.0099
4	65.0394	6549	72.4	549	36.2	0.0099
5	79.0542	7960	15.2	115	7.6	0.0099
6	79.0545	7960	19.6	149	9.8	0.0099
7	91.0543	9168	199.8	1515	100.0	0.0099
8	92.0575	9269	10.0	76	5.0	0.0099
9	105.0700	10579	55.6	422	27.8	0.0099
10	107.0849	10782	105.2	798	52.7	0.0099

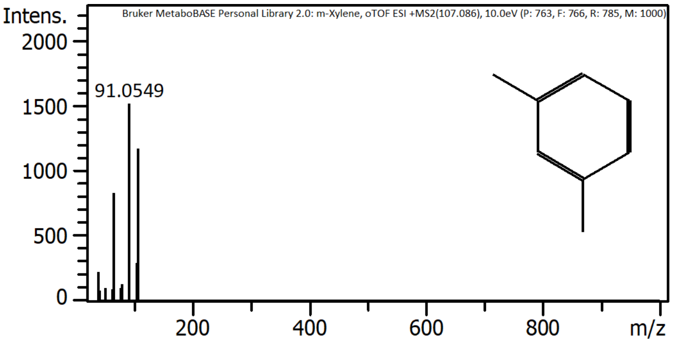

#	m/z	Res.	S/N	I	I %	FWHM
1	39.0236	3929	13.4	223	14.7	0.0099
2	41.0392	4132	4.7	79	5.2	0.0099
3	51.0239	5137	6.1	102	6.7	0.0099
4	63.0235	6346	5.4	90	5.9	0.0099
5	65.0393	6549	49.8	831	54.9	0.0099
6	77.0388	7757	6.1	102	6.7	0.0099
7	79.0546	7960	7.7	129	8.5	0.0099
8	91.0549	9168	90.8	1515	100.0	0.0099
9	105.0699	10579	17.9	299	19.7	0.0099
10	107.0858	10782	70.4	1174	77.5	0.0099

Cmpd 724, AutoMSn(193.0494), 8.64 min

Compound Spectrum List Report

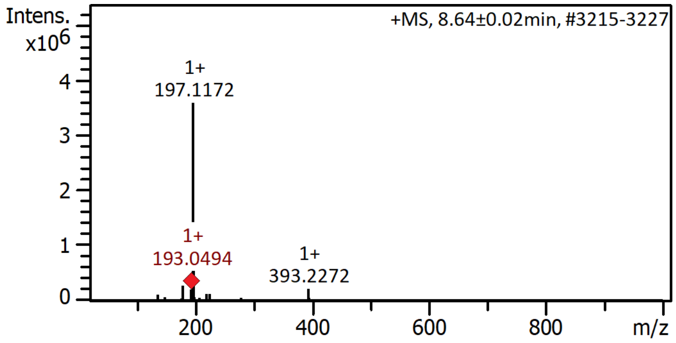

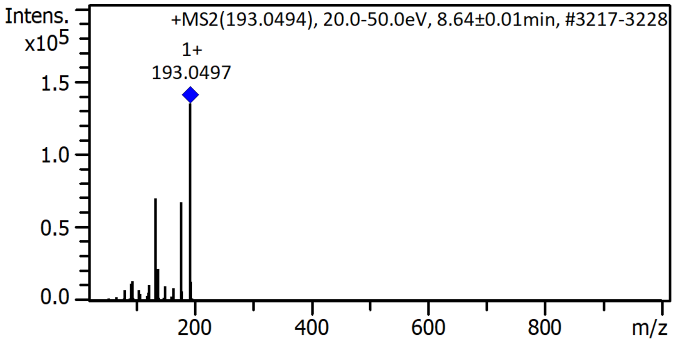

#	m/z	Res.	S/N	I	I %	FWHM
1	135.1165	10474	8739.0	104868	2.9	0.0129
2	147.0441	11092	5531.2	66374	1.9	0.0133
3	179.1065	11151	22447.4	269369	7.5	0.0161
4	193.0494	11101	16583.4	199000	5.6	0.0174
5	197.1172	9152	298216.4	3578597	100.0	0.0215
6	198.1205	10565	46930.9	563171	15.7	0.0188
7	199.1228	10680	5259.8	63117	1.8	0.0186
8	219.0994	11467	10102.8	121233	3.4	0.0191
9	225.1486	11633	10213.3	122560	3.4	0.0194
10	393.2272	12828	17697.0	212364	5.9	0.0307

#	m/z	Res.	S/N	I	I %	FWHM
1	91.0540	8731	845.5	11556	8.6	0.0104
2	94.0413	8936	981.8	13418	10.0	0.0105
3	122.0361	10541	788.0	10769	8.0	0.0116
4	133.0288	10049	5124.9	70040	52.0	0.0132
5	137.0604	10001	1570.7	21466	15.9	0.0137
6	150.0311	10895	719.9	9839	7.3	0.0138
7	165.0551	11472	630.5	8617	6.4	0.0144
8	178.0264	11573	4936.8	67470	50.1	0.0154
9	193.0497	11096	9854.4	134676	100.0	0.0174
10	194.0539	11325	934.3	12769	9.5	0.0171

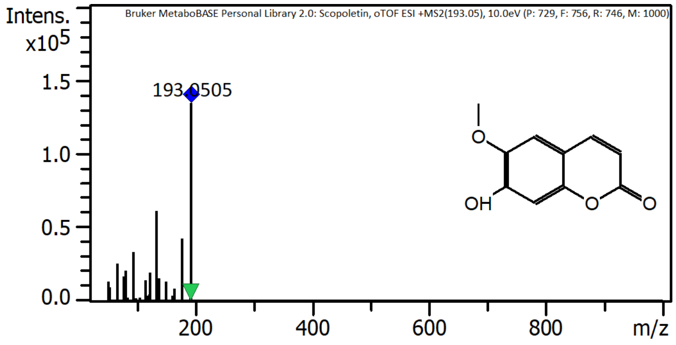

#	m/z	Res.	S/N	I	I %	FWHM
1	66.0472	3658	37.6	25319	18.8	0.0181
2	77.0390	4266	24.8	16700	12.4	0.0181
3	81.0336	4488	30.8	20740	15.4	0.0181
4	94.0395	5208	50.0	33669	25.0	0.0181
5	122.0369	6758	28.8	19393	14.4	0.0181
6	133.0273	7367	91.2	61412	45.6	0.0181
7	137.0590	7590	23.2	15622	11.6	0.0181
8	178.0237	9859	63.8	42962	31.9	0.0181
9	193.0464	10691	83.2	56025	41.6	0.0181
10	193.0505	10691	199.8	134542	100.0	0.0181

Cmpd 728, AutoMSn(225.1485), 8.69 min

Compound Spectrum List Report

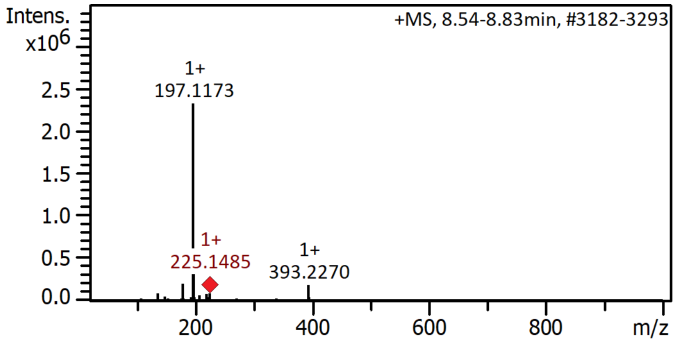

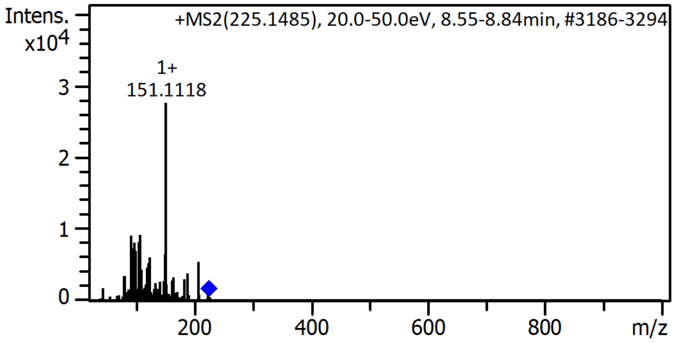

#	m/z	Res.	S/N	I	I %	FWHM
1	135.1166	10323	14855.1	89130	3.8	0.0131
2	147.0441	10569	8445.2	50671	2.2	0.0139
3	179.1064	10678	33171.6	199030	8.6	0.0168
4	197.1173	8002	387532.9	2325198	100.0	0.0246
5	198.1204	10501	67142.9	402857	17.3	0.0189
6	199.1234	10695	7485.6	44914	1.9	0.0186
7	207.1381	11153	11290.9	67745	2.9	0.0186
8	219.0995	11349	13297.6	79785	3.4	0.0193
9	225.1485	11559	14802.0	88812	3.8	0.0195
10	393.2270	11991	31096.0	186576	8.0	0.0328

#	m/z	Res.	S/N	I	I %	FWHM
1	91.0537	9311	1186.5	9097	33.0	0.0098
2	93.0698	9095	958.2	7346	26.6	0.0102
3	97.0647	9179	1065.5	8169	29.6	0.0106
4	99.0436	9269	914.8	7013	25.4	0.0107
5	105.0696	9862	1070.7	8209	29.8	0.0107
6	107.0855	9356	1206.2	9248	33.5	0.0114
7	123.0806	9738	798.6	6123	22.2	0.0126
8	149.0960	11549	851.8	6531	23.7	0.0129
9	151.1118	11113	3597.7	27582	100.0	0.0136
10	207.1376	12173	720.5	5524	20.0	0.0170

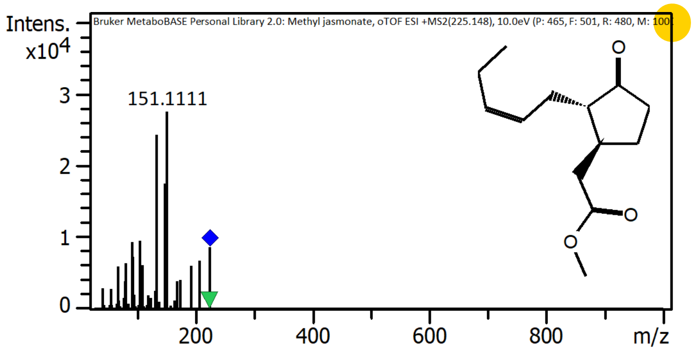

#	m/z	Res.	S/N	I	I %	FWHM
1	81.0696	5437	47.0	6482	23.5	0.0149
2	91.0540	6107	68.0	9378	34.0	0.0149
3	93.0700	6242	53.4	7364	26.7	0.0149
4	105.0700	7047	69.6	9599	34.8	0.0149
5	109.0647	7315	45.2	6234	22.6	0.0149
6	133.1008	8927	176.4	24327	88.3	0.0149
7	147.1164	9867	127.0	17515	63.6	0.0149
8	151.1111	10135	199.8	27555	100.0	0.0149
9	207.1360	13893	49.4	6813	24.7	0.0149
10	225.1463	15101	63.2	8716	31.6	0.0149

Cmpd 730, AutoMSn(153.1276), 8.70 min

Compound Spectrum List Report

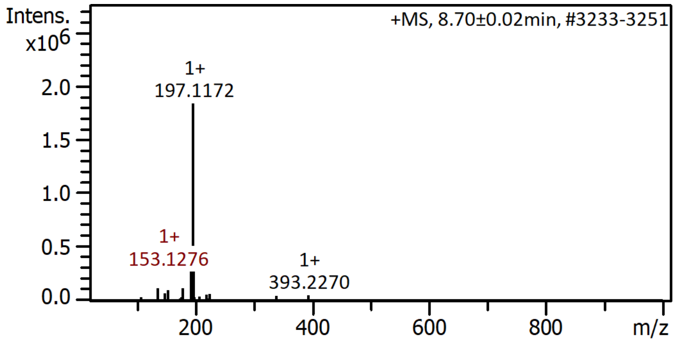

#	m/z	Res.	S/N	I	I %	FWHM
1	135.1167	10540	13018.8	117169	6.4	0.0128
2	147.0444	10908	7746.7	69721	3.8	0.0135
3	153.1276	10463	11014.6	99131	5.4	0.0146
4	179.1066	11212	13050.3	117453	6.4	0.0160
5	193.0499	10796	38580.0	347220	18.9	0.0179
6	197.1172	10244	204324.3	1838919	100.0	0.0192
7	198.1208	10863	30308.7	272779	14.8	0.0182
8	219.0996	11718	6287.5	56587	3.1	0.0187
9	225.1490	11298	7036.0	63324	3.4	0.0199
10	393.2270	12870	5416.0	48744	2.7	0.0306

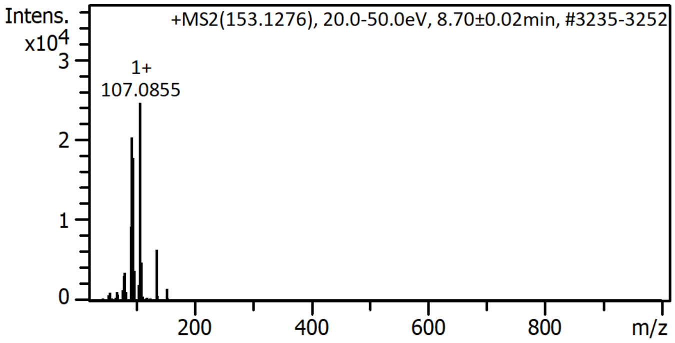

#	m/z	Res.	S/N	I	I %	FWHM
1	79.0543	8207	266.7	3067	12.5	0.0096
2	81.0699	8954	301.6	3469	14.1	0.0091
3	91.0542	9288	807.3	9284	37.8	0.0098
4	93.0696	9218	1764.2	20288	82.6	0.0101
5	95.0855	9152	1544.8	17765	72.3	0.0104
6	97.0646	10510	321.2	3694	15.0	0.0092
7	105.0691	10201	167.0	1921	7.8	0.0103
8	107.0855	9605	2135.2	24555	100.0	0.0111
9	109.1011	9507	421.2	4844	19.7	0.0115
10	135.1168	10779	556.5	6400	26.1	0.0125

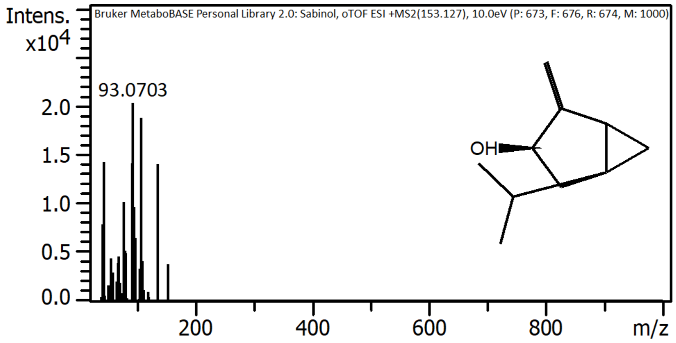

#	m/z	Res.	S/N	I	I %	FWHM
1	41.0394	3602	77.6	7872	38.8	0.0114
2	43.0552	3779	140.6	14262	70.4	0.0114
3	77.0387	6761	100.6	10205	50.4	0.0114
4	79.0545	6938	51.2	5194	25.6	0.0114
5	91.0541	7991	139.2	14120	69.7	0.0114
6	93.0703	8168	199.8	20268	100.0	0.0114
7	95.0852	8345	95.6	9698	47.8	0.0114
8	97.0647	8519	64.4	6533	32.2	0.0114
9	107.0848	9398	185.0	18766	92.6	0.0114
10	135.1154	11858	138.4	14039	69.3	0.0114

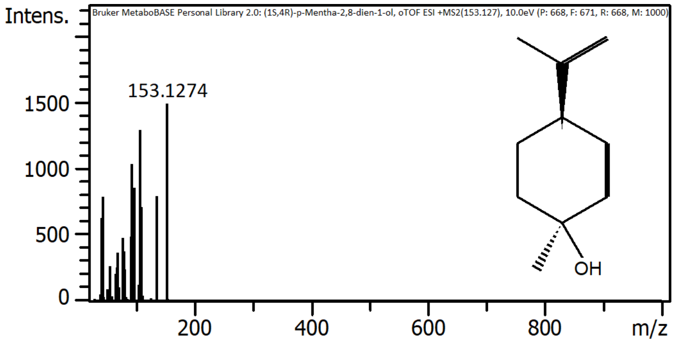

#	m/z	Res.	S/N	I	I %	FWHM
1	41.0397	3602	84.2	628	42.1	0.0114
2	43.0186	3775	105.8	789	53.0	0.0114
3	93.0699	8168	138.8	1035	69.5	0.0114
4	95.0853	8345	103.0	768	51.6	0.0114
5	95.0854	8345	101.2	755	50.7	0.0114
6	97.0648	8519	114.6	854	57.4	0.0114
7	107.0856	9398	173.2	1291	86.7	0.0114
8	109.1015	9575	95.8	714	47.9	0.0114
9	135.1169	11858	106.8	796	53.5	0.0114
10	153.1274	13439	199.8	1490	100.0	0.0114

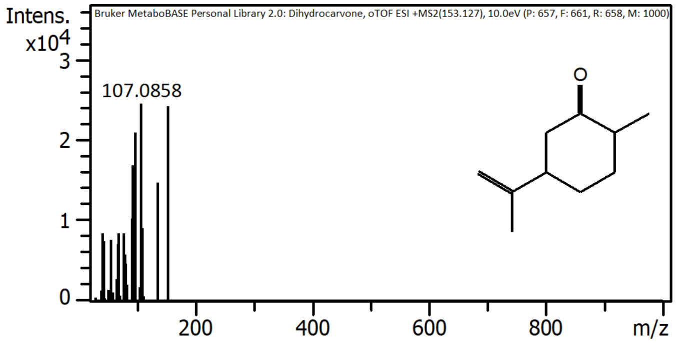

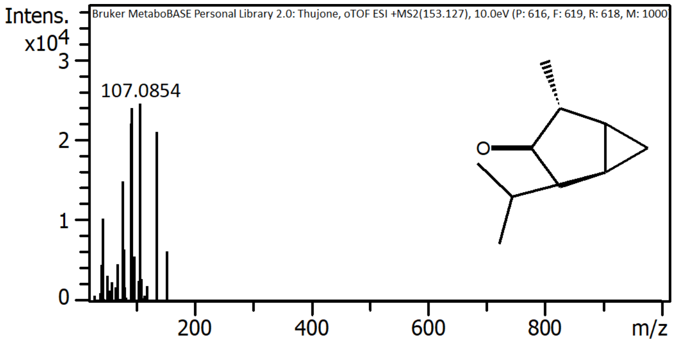

Compound Spectrum List Report

#	m/z	Res.	S/N	I	I %	FWHM
1	41.0400	3602	69.2	8496	34.6	0.0114
2	69.0705	6062	68.8	8447	34.4	0.0114
3	91.0547	7991	84.0	10313	42.0	0.0114
4	93.0704	8168	137.8	16918	69.0	0.0114
5	95.0860	8345	88.6	10878	44.3	0.0114
6	97.0651	8519	170.6	20945	85.4	0.0114
7	107.0858	9398	199.8	24530	100.0	0.0114
8	109.1011	9575	73.8	9061	36.9	0.0114
9	135.1169	11858	119.8	14708	60.0	0.0114
10	153.1274	13439	197.0	24187	98.6	0.0114

#	m/z	Res.	S/N	I	I %	FWHM
1	43.0183	3775	25.8	5697	23.2	0.0114
2	43.0554	3779	46.4	10264	41.8	0.0114
3	77.0392	6761	67.3	14880	60.7	0.0114
4	79.0547	6938	29.3	6483	26.4	0.0114
5	91.0543	7991	99.7	22026	89.8	0.0114
6	93.0699	8168	108.2	23917	97.5	0.0114
7	97.0654	8519	25.4	5623	22.9	0.0114
8	107.0854	9398	111.0	24530	100.0	0.0114
9	135.1174	11858	95.1	21019	85.7	0.0114
10	153.1277	13439	28.2	6237	25.4	0.0114

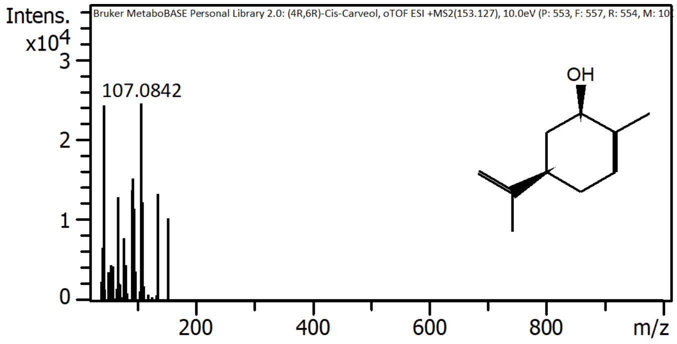

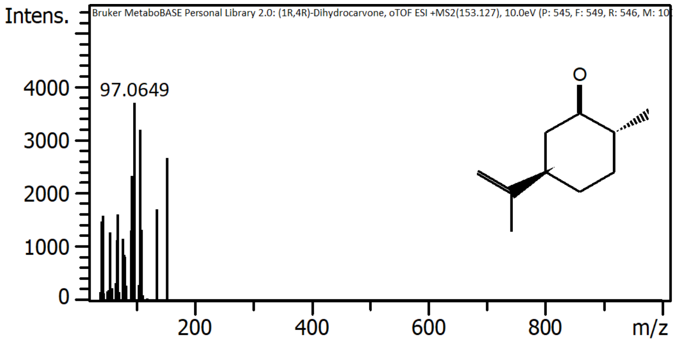

#	m/z	Res.	S/N	I	I %	FWHM
1	43.0180	3775	105.6	20749	84.6	0.0114
2	43.0181	3775	123.4	24236	98.8	0.0114
3	67.0537	5885	65.9	12940	52.8	0.0114
4	91.0537	7991	70.3	13800	56.3	0.0114
5	93.0688	8168	77.3	15175	61.9	0.0114
6	95.0838	8345	58.3	11443	46.6	0.0114
7	107.0842	9398	124.9	24530	100.0	0.0114
8	109.1001	9575	62.4	12253	49.9	0.0114
9	135.1153	11858	67.6	13284	54.2	0.0114
10	153.1251	13438	52.3	10264	41.8	0.0114

#	m/z	Res.	S/N	I	I %	FWHM
1	41.0396	3602	80.6	1489	40.3	0.0114
2	43.0187	3775	86.2	1592	43.1	0.0114
3	69.0702	6062	87.8	1622	43.9	0.0114
4	93.0699	8168	126.2	2331	63.2	0.0114
5	95.0856	8345	81.4	1504	40.7	0.0114
6	97.0649	8519	199.8	3691	100.0	0.0114
7	107.0853	9398	172.6	3188	86.4	0.0114
8	109.1008	9575	72.0	1330	36.0	0.0114
9	135.1166	11858	92.6	1710	46.3	0.0114
10	153.1271	13439	144.0	2660	72.1	0.0114

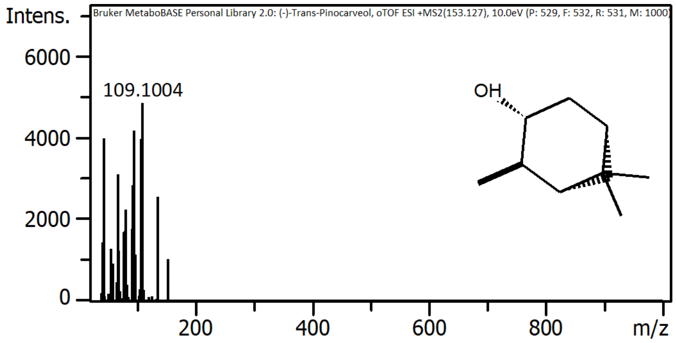

#	m/z	Res.	S/N	I	I %	FWHM
1	43.0184	3775	164.4	3981	82.3	0.0114
2	67.0543	5885	128.2	3105	64.2	0.0114
3	79.0546	6938	71.6	1734	35.8	0.0114
4	81.0692	7115	92.6	2243	46.3	0.0114
5	91.0546	7991	73.2	1773	36.6	0.0114
6	93.0700	8168	117.6	2848	58.9	0.0114
7	95.0860	8345	172.0	4165	86.1	0.0114
8	107.0861	9398	164.0	3972	82.1	0.0114
9	109.1004	9575	199.8	4839	100.0	0.0114
10	135.1162	11858	105.8	2562	53.0	0.0114

Cmpd 743, AutoMSn(107.0850), 8.86 min

Compound Spectrum List Report

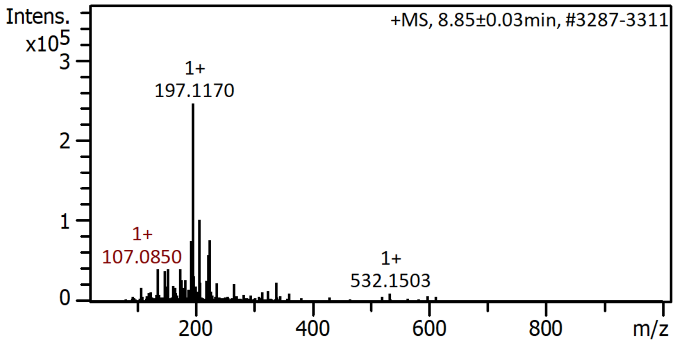

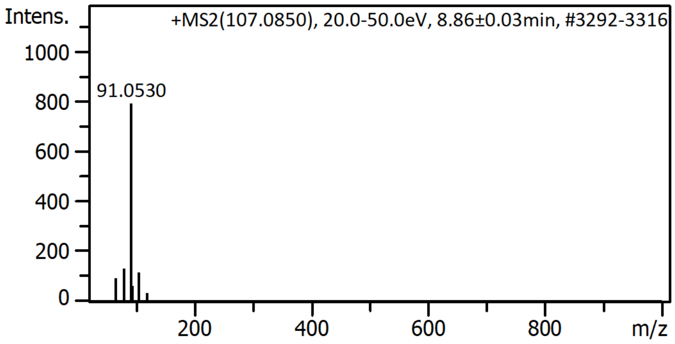

#	m/z	Res.	S/N	I	I %	FWHM
1	135.1165	10449	2244.6	40402	16.4	0.0129
2	147.0442	10957	2117.2	38110	15.5	0.0134
3	153.1273	10320	2503.4	45062	18.3	0.0148
4	175.0393	11313	2309.7	41575	16.9	0.0155
5	193.0491	11584	4216.8	75902	30.9	0.0167
6	197.1170	10964	13653.2	245758	100.0	0.0180
7	198.1200	11125	1758.4	31652	12.9	0.0178
8	207.1378	11490	5650.7	101712	41.4	0.0180
9	223.0592	11397	3222.9	58013	23.6	0.0196
10	225.1481	11607	4271.9	76894	31.3	0.0194

#	m/z	Res.	S/N	I	I %	FWHM
1	65.0359	11122	2.7	95	12.0	0.0058
2	79.0525	12594	3.8	132	16.7	0.0063
3	80.0505	12993	1.1	40	5.0	0.0062
4	91.0530	8047	22.6	790	100.0	0.0113
5	94.0397	13723	1.8	62	7.8	0.0069
6	105.0709	11819	3.3	117	14.8	0.0089
7	119.0556	16498	1.0	35	4.4	0.0072

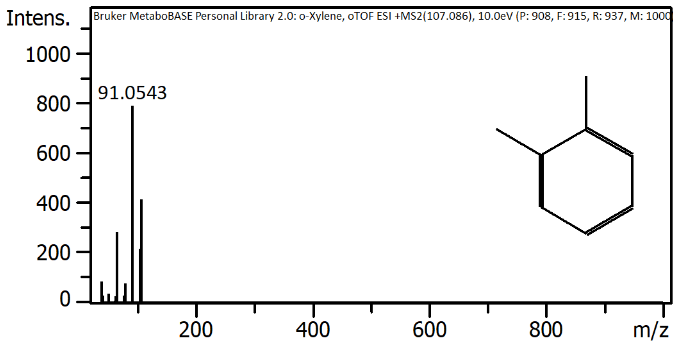

#	m/z	Res.	S/N	I	I %	FWHM
1	39.0237	4182	20.4	81	10.2	0.0093
2	39.0238	4182	21.8	86	10.9	0.0093
3	65.0393	6969	63.6	251	31.8	0.0093
4	65.0394	6969	72.4	286	36.2	0.0093
5	79.0542	8471	15.2	60	7.6	0.0093
6	79.0545	8471	19.6	77	9.8	0.0093
7	91.0543	9757	199.8	789	100.0	0.0093
8	92.0575	9865	10.0	39	5.0	0.0093
9	105.0700	11259	55.6	219	27.8	0.0093
10	107.0849	11475	105.2	415	52.7	0.0093

Cmpd 747, AutoMSn(611.1603), 8.91 min

Compound Spectrum List Report

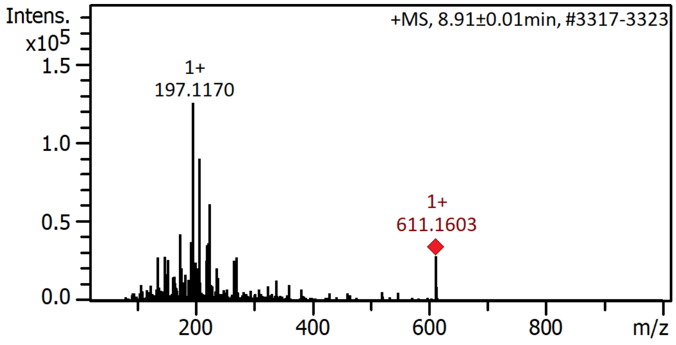

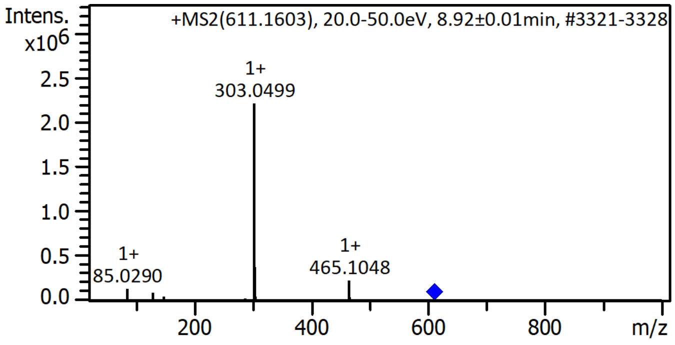

#	m/z	Res.	S/N	I	I %	FWHM
1	147.0442	10629	1558.1	28045	22.4	0.0138
2	175.0384	11003	2342.4	42164	33.7	0.0159
3	193.0494	10986	2078.9	37420	29.9	0.0176
4	197.1170	10741	6948.8	125079	100.0	0.0184
5	207.1379	11086	5009.6	90174	72.1	0.0187
6	221.1888	11990	1959.6	35273	28.2	0.0184
7	223.0593	10423	1757.5	31635	25.3	0.0214
8	223.1327	11504	2045.8	36824	29.4	0.0194
9	225.1485	11326	3409.9	61378	49.1	0.0199
10	611.1603	12654	1584.0	28512	22.8	0.0483

#	m/z	Res.	S/N	I	I %	FWHM
1	71.0500	6942	784.3	18824	0.9	0.0102
2	85.0290	7715	5629.7	135113	6.1	0.0110
3	129.0553	9784	3641.2	87388	3.9	0.0132
4	147.0660	10139	2065.1	49562	2.2	0.0145
5	287.0569	11072	982.8	23587	1.1	0.0259
6	303.0499	11027	92181.6	2212359	100.0	0.0275
7	304.0540	12136	15695.1	376683	17.0	0.0251
8	305.0554	11942	1920.7	46098	2.1	0.0255
9	465.1048	12465	9516.8	228403	10.3	0.0373
10	466.1083	12775	1682.5	40380	1.8	0.0365

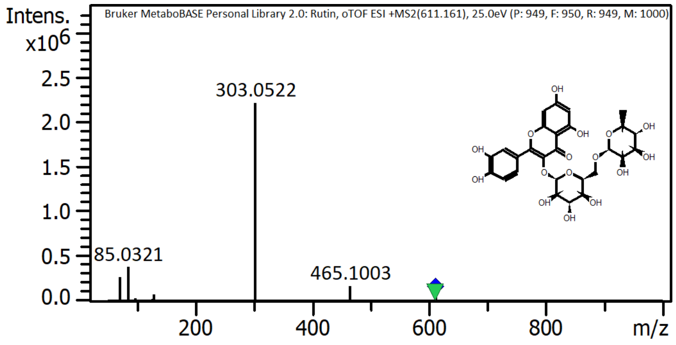

#	m/z	Res.	S/N	I	I %	FWHM
1	71.0529	2600	24.0	265483	12.0	0.0273
2	85.0316	3112	6.4	70795	3.2	0.0273
3	85.0321	3112	34.4	380526	17.2	0.0273
4	129.0578	4723	6.6	73008	3.3	0.0273
5	303.0479	11089	50.0	553090	25.0	0.0273
6	303.0519	11090	83.2	920341	41.6	0.0273
7	303.0522	11090	199.8	2210147	100.0	0.0273
8	303.2075	11095	3.4	37610	1.7	0.0273
9	465.1003	17019	15.2	168139	7.6	0.0273
10	611.1564	22364	6.0	66371	3.0	0.0273

Cmpd 749, AutoMSn(203.1798), 8.95 min

Compound Spectrum List Report

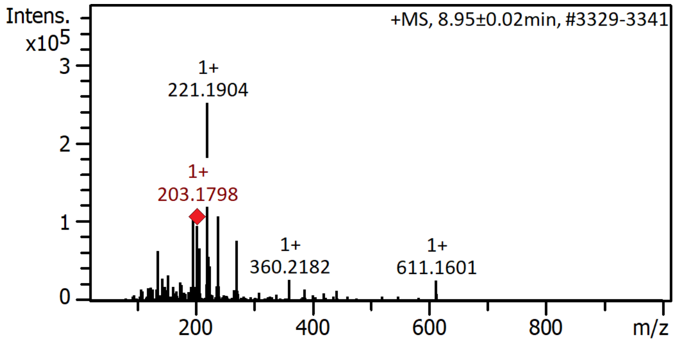

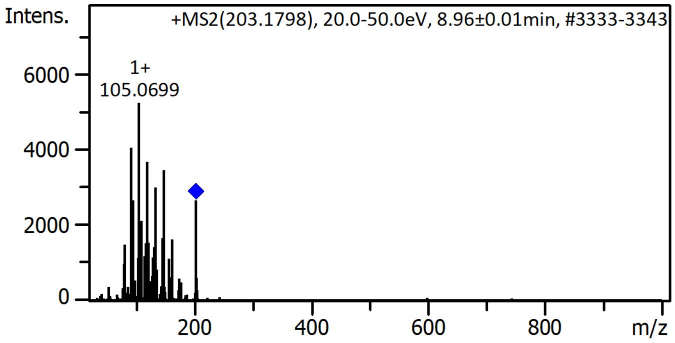

#	m/z	Res.	S/N	I	I %	FWHM
1	135.1169	10372	5343.3	64120	25.6	0.0130
2	197.1176	11277	8737.8	104854	41.8	0.0175
3	203.1798	11642	7928.7	95144	37.9	0.0175
4	207.1384	11367	5591.4	67097	26.7	0.0182
5	221.1904	11338	20909.8	250917	100.0	0.0195
6	222.1942	11344	3313.1	39758	15.8	0.0196
7	223.1334	11597	4689.6	56275	22.4	0.0192
8	225.1484	11603	3608.4	43301	17.3	0.0194
9	239.2011	11856	8984.4	107813	43.0	0.0202
10	271.1366	11303	6373.8	76485	30.5	0.0240

#	m/z	Res.	S/N	I	I %	FWHM
1	91.0540	9470	258.3	4046	77.4	0.0096
2	95.0856	8225	169.6	2656	50.8	0.0116
3	105.0699	9606	333.5	5225	100.0	0.0109
4	107.0839	9218	122.8	1923	36.8	0.0116
5	109.0996	8246	135.6	2124	40.7	0.0132
6	119.0851	9701	234.3	3670	70.2	0.0123
7	133.1006	10156	191.5	3000	57.4	0.0131
8	145.1002	9801	106.2	1664	31.8	0.0148
9	147.1167	8507	220.4	3452	66.1	0.0173
10	203.1792	11126	170.0	2663	51.0	0.0183

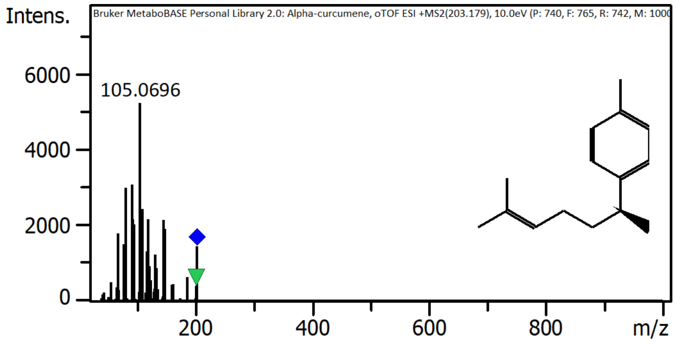

#	m/z	Res.	S/N	I	I %	FWHM
1	81.0697	7589	114.8	2999	57.5	0.0107
2	91.0545	8523	118.0	3083	59.1	0.0107
3	93.0690	8712	82.8	2163	41.4	0.0107
4	95.0851	8901	78.0	2038	39.0	0.0107
5	105.0696	9835	199.8	5220	100.0	0.0107
6	107.0844	10024	93.4	2440	46.7	0.0107
7	109.1014	10213	93.4	2440	46.7	0.0107
8	119.0845	11147	82.8	2163	41.4	0.0107
9	145.0992	13582	82.2	2148	41.1	0.0107
10	147.1150	13771	73.2	1912	36.6	0.0107

Cmpd 750, AutoMSn(135.1169), 8.96 min

Compound Spectrum List Report

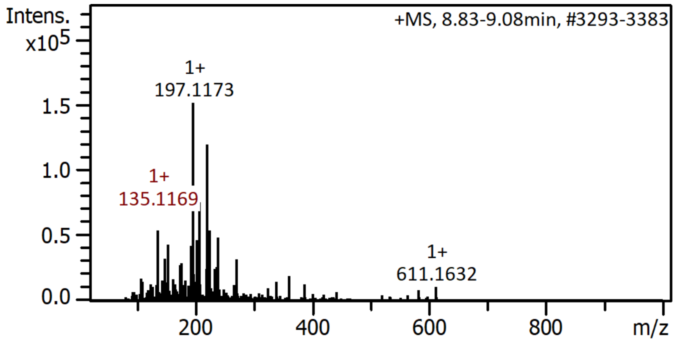

#	m/z	Res.	S/N	I	I %	FWHM
1	135.1169	10294	10554.4	54280	35.9	0.0131
2	153.1274	10917	8460.9	43513	28.8	0.0140
3	193.0496	11301	8181.7	42077	27.8	0.0171
4	197.1173	11244	29421.2	151309	100.0	0.0175
5	203.1799	11409	9044.1	46513	30.7	0.0178
6	207.1382	11585	14685.4	75525	49.9	0.0179
7	221.1905	11142	23253.7	119590	79.0	0.0199
8	223.0600	11500	6712.8	34523	22.8	0.0194
9	225.1488	11589	10515.5	54080	35.7	0.0194
10	239.2012	11929	9507.8	48897	32.3	0.0201

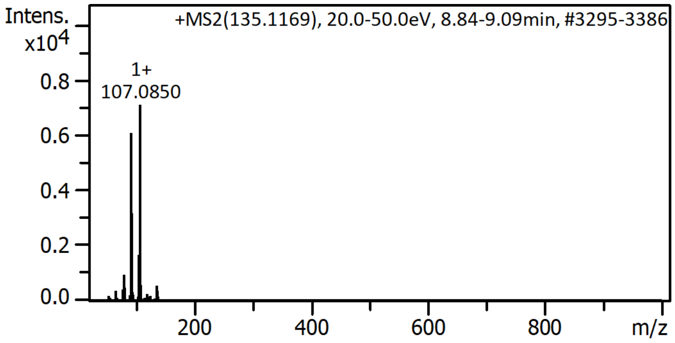

#	m/z	Res.	S/N	I	I %	FWHM
1	79.0549	8721	135.1	927	13.0	0.0091
2	81.0696	9491	68.7	471	6.6	0.0085
3	91.0541	9463	887.1	6083	85.6	0.0096
4	92.0612	6135	60.6	415	5.8	0.0150
5	93.0697	9569	466.0	3195	45.0	0.0097
6	105.0696	10857	245.5	1683	23.7	0.0097
7	106.0729	10007	75.4	517	7.3	0.0106
8	107.0850	9582	1036.6	7108	100.0	0.0112
9	108.0883	9424	81.7	560	7.9	0.0115
10	135.1161	12016	78.5	539	7.6	0.0112

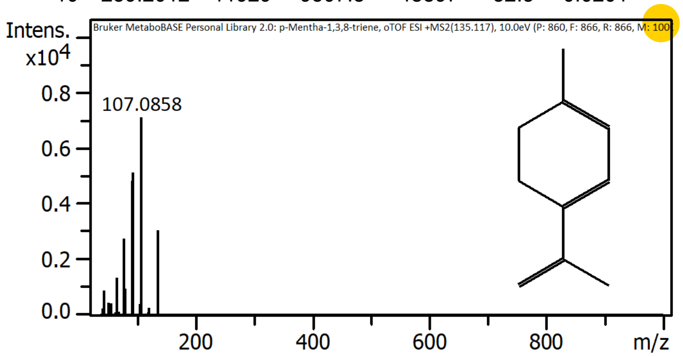

#	m/z	Res.	S/N	I	I %	FWHM
1	43.0556	3893	24.6	874	12.3	0.0111
2	51.0243	4613	12.8	455	6.4	0.0111
3	65.0401	5880	38.4	1365	19.2	0.0111
4	77.0394	6965	77.6	2758	38.8	0.0111
5	79.0540	7147	26.6	945	13.3	0.0111
6	79.0545	7147	20.4	725	10.2	0.0111
7	91.0549	8232	136.0	4834	68.1	0.0111
8	93.0709	8414	144.2	5125	72.2	0.0111
9	107.0858	9681	199.8	7101	100.0	0.0111
10	135.1169	12216	86.2	3064	43.1	0.0111

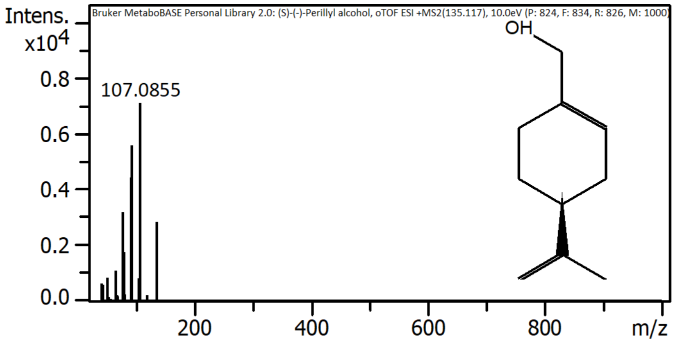

#	m/z	Res.	S/N	I	I %	FWHM
1	41.0395	3710	11.3	640	9.0	0.0111
2	51.0237	4613	14.8	839	11.8	0.0111
3	65.0391	5880	19.3	1095	15.4	0.0111
4	77.0390	6965	56.4	3206	45.1	0.0111
5	79.0547	7147	31.1	1770	24.9	0.0111
6	91.0542	8232	78.1	4443	62.6	0.0111
7	93.0700	8414	98.4	5594	78.8	0.0111
8	105.0694	9499	14.3	810	11.4	0.0111
9	107.0855	9681	124.9	7101	100.0	0.0111
10	135.1164	12216	50.3	2857	40.2	0.0111

Cmpd 752, AutoMSn(153.1272), 8.98 min

Compound Spectrum List Report

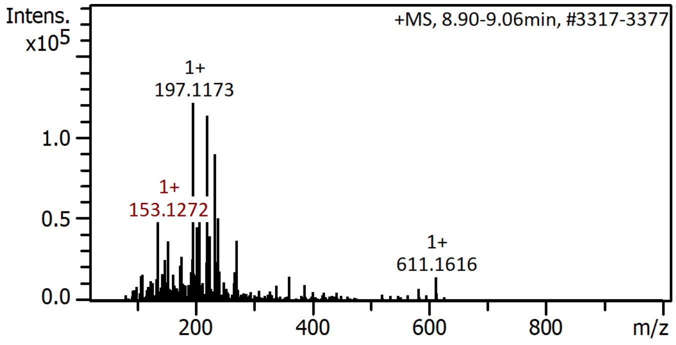

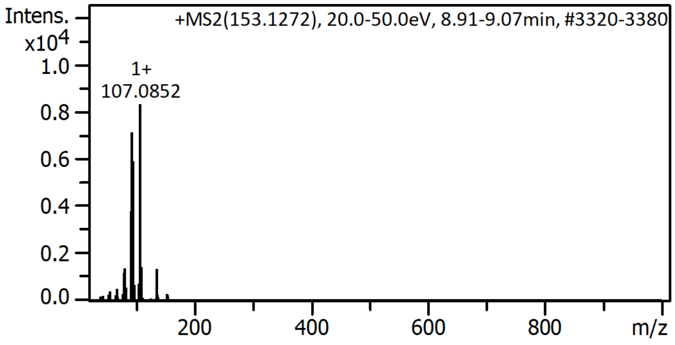

#	m/z	Res.	S/N	I	I %	FWHM
1	135.1167	10464	6978.4	50244	41.5	0.0129
2	153.1272	10781	5061.8	36445	30.1	0.0142
3	197.1173	11171	16829.6	121173	100.0	0.0176
4	203.1795	11622	6287.9	45273	37.4	0.0175
5	207.1381	11285	7965.6	57352	47.3	0.0184
6	221.1903	11515	15752.6	113419	93.6	0.0192
7	225.1489	11531	5521.4	39754	32.8	0.0195
8	234.1129	11564	12462.1	89727	74.0	0.0202
9	239.2010	11769	7045.3	50726	41.9	0.0203
10	271.1370	11536	5147.8	37064	30.6	0.0235

#	m/z	Res.	S/N	I	I %	FWHM
1	79.0530	8977	124.0	1190	14.3	0.0088
2	81.0702	7327	143.7	1379	16.6	0.0111
3	91.0544	8852	396.0	3801	45.7	0.0103
4	93.0697	8858	742.5	7128	85.6	0.0105
5	95.0853	9130	615.7	5910	71.0	0.0104
6	105.0696	7619	74.9	719	8.6	0.0138
7	107.0852	10355	867.2	8325	100.0	0.0103
8	108.0872	11216	71.4	686	8.2	0.0096
9	109.1014	8673	148.9	1429	17.2	0.0126
10	135.1164	10741	140.5	1348	16.2	0.0126

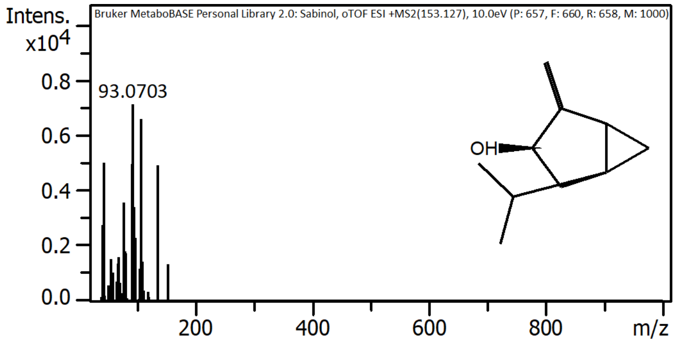

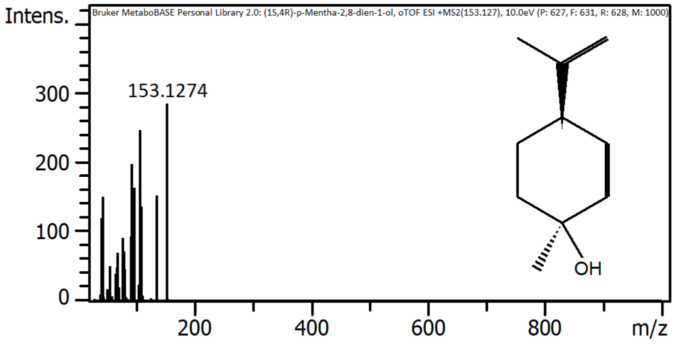

#	m/z	Res.	S/N	I	I %	FWHM
1	41.0394	3548	77.6	2766	38.8	0.0116
2	43.0552	3722	140.6	5011	70.4	0.0116
3	77.0387	6660	100.6	3585	50.4	0.0116
4	79.0545	6835	51.2	1825	25.6	0.0116
5	91.0541	7872	139.2	4961	69.7	0.0116
6	93.0703	8046	199.8	7121	100.0	0.0116
7	95.0852	8221	95.6	3407	47.8	0.0116
8	97.0647	8392	64.4	2295	32.2	0.0116
9	107.0848	9258	185.0	6593	92.6	0.0116
10	135.1154	11682	138.4	4933	69.3	0.0116

#	m/z	Res.	S/N	I	I %	FWHM
1	41.0397	3548	84.2	120	42.1	0.0116
2	43.0186	3719	105.8	151	53.0	0.0116
3	93.0699	8046	138.8	198	69.5	0.0116
4	95.0853	8221	103.0	147	51.6	0.0116
5	95.0854	8221	101.2	144	50.7	0.0116
6	97.0648	8392	114.6	163	57.4	0.0116
7	107.0856	9258	173.2	247	86.7	0.0116
8	109.1015	9432	95.8	137	47.9	0.0116
9	135.1169	11682	106.8	152	53.5	0.0116
10	153.1274	13239	199.8	285	100.0	0.0116

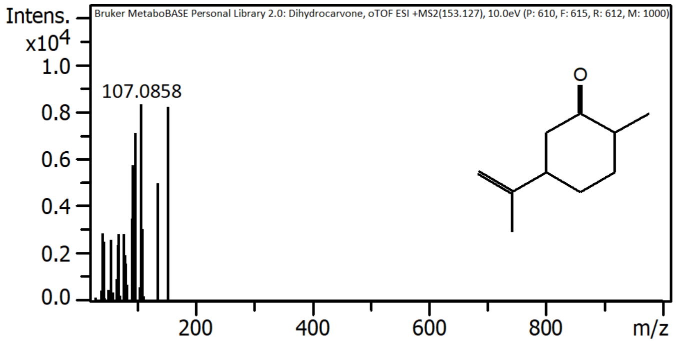

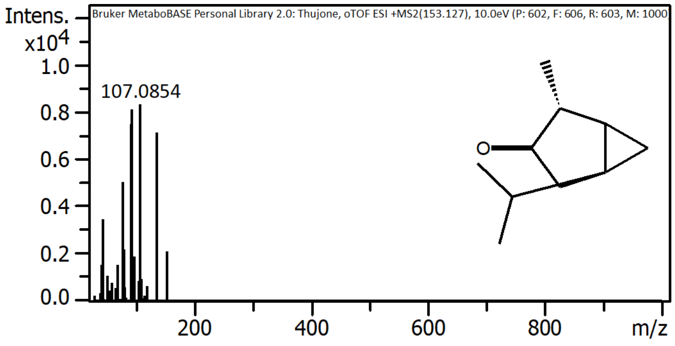

Compound Spectrum List Report

#	m/z	Res.	S/N	I	I %	FWHM
1	41.0400	3548	69.2	2881	34.6	0.0116
2	69.0705	5972	68.8	2864	34.4	0.0116
3	91.0547	7872	84.0	3497	42.0	0.0116
4	93.0704	8046	137.8	5736	69.0	0.0116
5	95.0860	8221	88.6	3688	44.3	0.0116
6	97.0651	8392	170.6	7101	85.4	0.0116
7	107.0858	9258	199.8	8317	100.0	0.0116
8	109.1011	9432	73.8	3072	36.9	0.0116
9	135.1169	11682	119.8	4987	60.0	0.0116
10	153.1274	13239	197.0	8200	98.6	0.0116

#	m/z	Res.	S/N	I	I %	FWHM
1	43.0183	3719	25.8	1931	23.2	0.0116
2	43.0554	3722	46.4	3480	41.8	0.0116
3	77.0392	6660	67.3	5045	60.7	0.0116
4	79.0547	6835	29.3	2198	26.4	0.0116
5	91.0543	7872	99.7	7468	89.8	0.0116
6	93.0699	8046	108.2	8109	97.5	0.0116
7	97.0654	8392	25.4	1906	22.9	0.0116
8	107.0854	9258	111.0	8317	100.0	0.0116
9	135.1174	11682	95.1	7126	85.7	0.0116
10	153.1277	13239	28.2	2115	25.4	0.0116

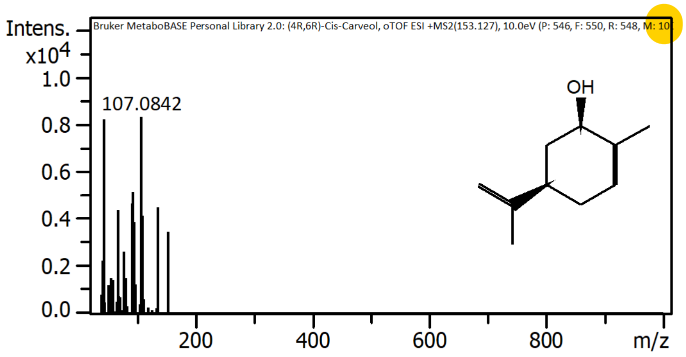

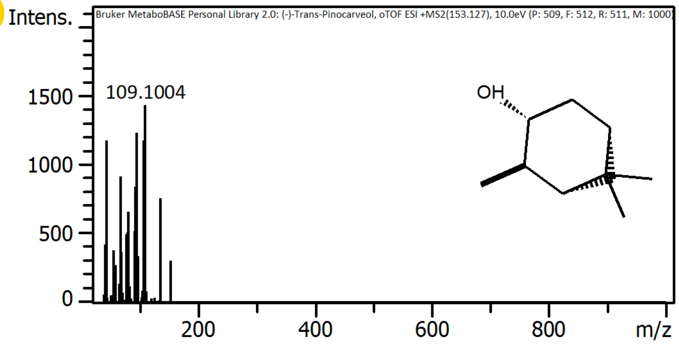

#	m/z	Res.	S/N	I	I %	FWHM
1	43.0180	3719	105.6	7035	84.6	0.0116
2	43.0181	3719	123.4	8217	98.8	0.0116
3	67.0537	5797	65.9	4387	52.8	0.0116
4	91.0537	7872	70.3	4679	56.3	0.0116
5	93.0688	8046	77.3	5145	61.9	0.0116
6	95.0838	8221	58.3	3880	46.6	0.0116
7	107.0842	9258	124.9	8317	100.0	0.0116
8	109.1001	9432	62.4	4154	49.9	0.0116
9	135.1153	11682	67.6	4504	54.2	0.0116
10	153.1251	13239	52.3	3480	41.8	0.0116

#	m/z	Res.	S/N	I	I %	FWHM
1	43.0184	3719	164.4	1175	82.3	0.0116
2	67.0543	5797	128.2	916	64.2	0.0116
3	79.0546	6835	71.6	512	35.8	0.0116
4	81.0692	7009	92.6	662	46.3	0.0116
5	91.0546	7872	73.2	523	36.6	0.0116
6	93.0700	8046	117.6	840	58.9	0.0116
7	95.0860	8221	172.0	1229	86.1	0.0116
8	107.0861	9258	164.0	1172	82.1	0.0116
9	109.1004	9432	199.8	1428	100.0	0.0116
10	135.1162	11682	105.8	756	53.0	0.0116

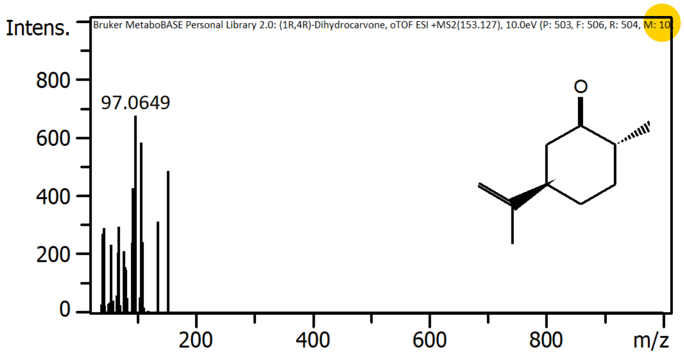

#	m/z	Res.	S/N	I	I %	FWHM
1	41.0396	3548	80.6	272	40.3	0.0116
2	43.0187	3719	86.2	291	43.1	0.0116
3	69.0702	5972	87.8	297	43.9	0.0116
4	93.0699	8046	126.2	427	63.2	0.0116
5	95.0856	8221	81.4	275	40.7	0.0116
6	97.0649	8392	199.8	675	100.0	0.0116
7	107.0853	9258	172.6	583	86.4	0.0116
8	109.1008	9432	72.0	243	36.0	0.0116
9	135.1166	11682	92.6	313	46.3	0.0116
10	153.1271	13239	144.0	487	72.1	0.0116

Cmpd 764, AutoMSn(165.0545), 9.09 min

Compound Spectrum List Report

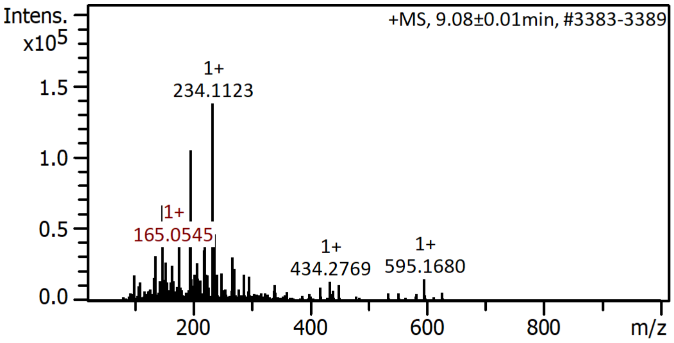

#	m/z	Res.	S/N	I	I %	FWHM
1	135.1168	9720	1750.3	31506	22.9	0.0139
2	147.0439	9891	3694.4	66500	48.4	0.0149
3	177.0547	11026	2353.5	42363	30.8	0.0161
4	195.1374	10654	2435.8	43845	31.9	0.0183
5	197.1174	11148	5838.3	105090	76.4	0.0177
6	219.1748	10941	1971.6	35488	25.8	0.0200
7	221.1893	10917	2245.0	40410	29.4	0.0203
8	234.1123	11094	7637.5	137475	100.0	0.0211
9	237.1851	11578	2577.4	46393	33.7	0.0205
10	268.1180	11963	1701.7	30631	22.3	0.0224

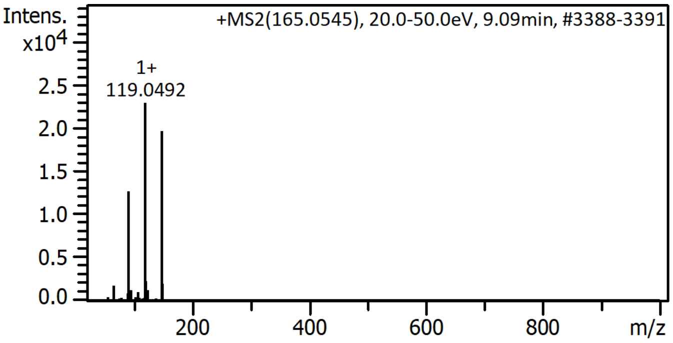

#	m/z	Res.	S/N	I	I %	FWHM
1	65.0384	8416	70.6	1730	7.6	0.0077
2	89.0401	6060	34.8	854	3.7	0.0147
3	91.0547	8572	518.2	12695	55.4	0.0106
4	95.0507	6075	50.5	1237	5.4	0.0156
5	107.0810	8586	41.4	1015	4.4	0.0125
6	119.0492	9483	935.1	22911	100.0	0.0126
7	120.0535	9400	93.3	2286	10.0	0.0128
8	123.0436	12609	48.9	1199	5.2	0.0098
9	147.0447	10468	802.0	19648	85.8	0.0140
10	148.0476	13026	79.8	1956	8.5	0.0114

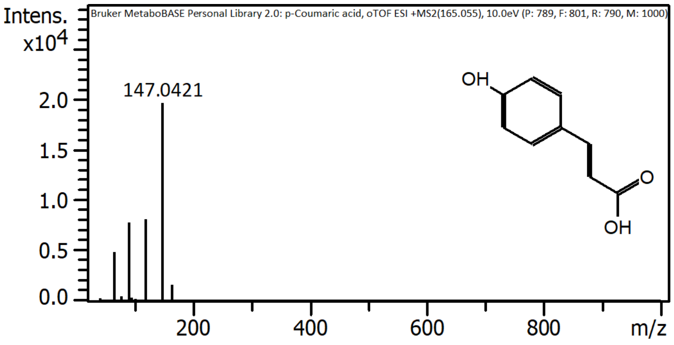

#	m/z	Res.	S/N	I	I %	FWHM
1	65.0376	5251	3.7	511	2.6	0.0124
2	65.0395	5251	35.7	4912	25.0	0.0124
3	91.0513	7351	7.7	1061	5.4	0.0124
4	91.0545	7351	56.9	7820	39.8	0.0124
5	119.0489	9611	59.4	8174	41.6	0.0124
6	119.0503	9611	18.4	2535	12.9	0.0124
7	147.0421	11871	142.7	19628	100.0	0.0124
8	147.0455	11871	32.3	4440	22.6	0.0124
9	164.9162	13314	11.6	1591	8.1	0.0124
10	165.0509	13325	6.1	845	4.3	0.0124

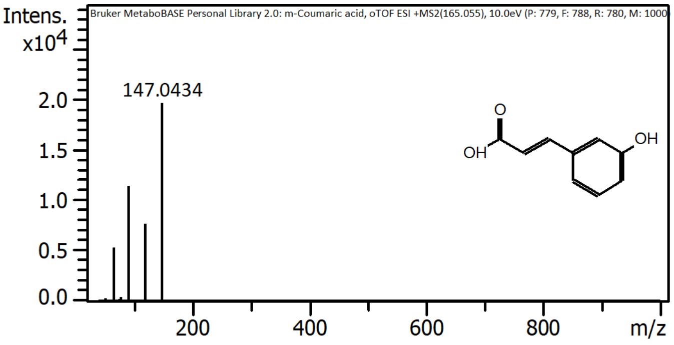

#	m/z	Res.	S/N	I	I %	FWHM
1	41.0374	3313	1.8	177	0.9	0.0124
2	51.0216	4119	2.6	255	1.3	0.0124
3	65.0383	5251	54.8	5384	27.4	0.0124
4	75.0213	6057	2.4	236	1.2	0.0124
5	77.0383	6219	4.2	413	2.1	0.0124
6	91.0538	7351	116.4	11435	58.3	0.0124
7	101.0376	8157	1.8	177	0.9	0.0124
8	103.0502	8319	1.4	138	0.7	0.0124
9	119.0481	9611	78.6	7722	39.3	0.0124
10	147.0434	11871	199.8	19628	100.0	0.0124

Cmpd 772, AutoMSn(219.1738), 9.16 min

Compound Spectrum List Report

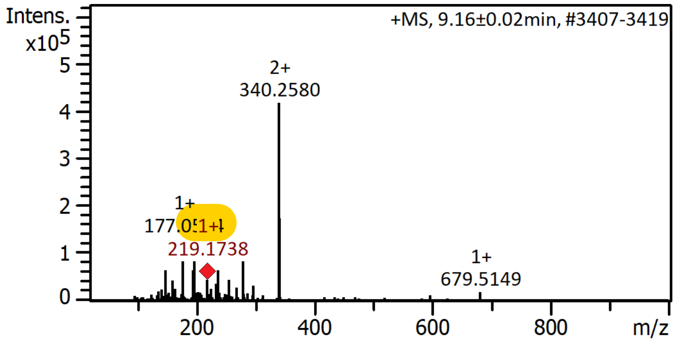

#	m/z	Res.	S/N	I	I %	FWHM
1	147.0436	9155	5376.8	64522	15.5	0.0161
2	177.0544	9308	11002.3	132027	31.7	0.0190
3	195.0651	9742	5336.4	64037	15.4	0.0200
4	197.1166	9444	7686.3	92236	22.1	0.0209
5	219.1738	9664	3704.3	44452	10.7	0.0227
6	237.1845	9282	5384.3	64611	15.5	0.0256
7	255.1950	9571	3608.2	43299	10.4	0.0267
8	279.1620	9585	8218.3	98619	23.7	0.0291
9	340.2580	10521	34728.9	416747	100.0	0.0323
10	340.7591	10687	14573.7	174885	42.0	0.0319

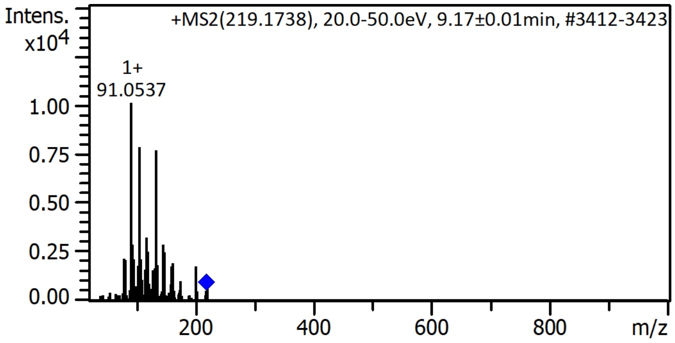

#	m/z	Res.	S/N	I	I %	FWHM
1	79.0533	7745	133.9	2186	21.6	0.0102
2	91.0537	7707	618.6	10104	100.0	0.0118
3	93.0689	9570	177.6	2901	28.7	0.0097
4	105.0693	9423	482.0	7873	77.9	0.0112
5	117.0667	6425	198.5	3242	32.1	0.0182
6	119.0485	7766	156.0	2547	25.2	0.0153
7	119.0844	8607	148.0	2417	23.9	0.0138
8	133.0998	10079	471.5	7701	76.2	0.0132
9	145.0991	9762	176.3	2880	28.5	0.0149
10	147.0432	9992	153.5	2507	24.8	0.0147

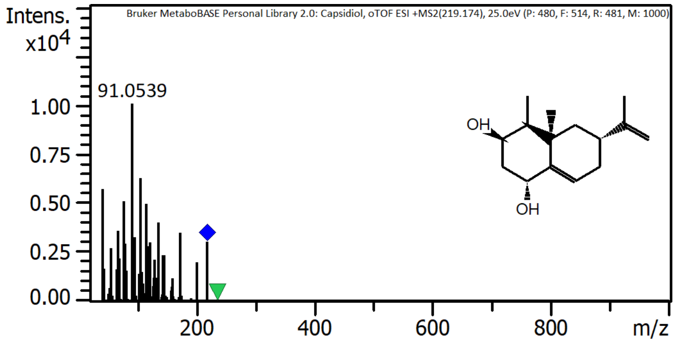

#	m/z	Res.	S/N	I	I %	FWHM
1	41.0395	4140	113.4	5729	56.8	0.0099
2	67.0547	6764	71.2	3597	35.6	0.0099
3	77.0403	7771	101.4	5123	50.8	0.0099
4	91.0539	9185	199.8	10094	100.0	0.0099
5	95.0865	9592	64.8	3274	32.4	0.0099
6	105.0698	10599	124.8	6305	62.5	0.0099
7	115.0555	11606	98.4	4971	49.2	0.0099
8	135.0825	13626	80.2	4052	40.1	0.0099
9	173.1334	17465	69.4	3506	34.7	0.0099
10	219.1758	22109	60.6	3061	30.3	0.0099

Cmpd 778, AutoMSn(465.1028), 9.22 min

Compound Spectrum List Report

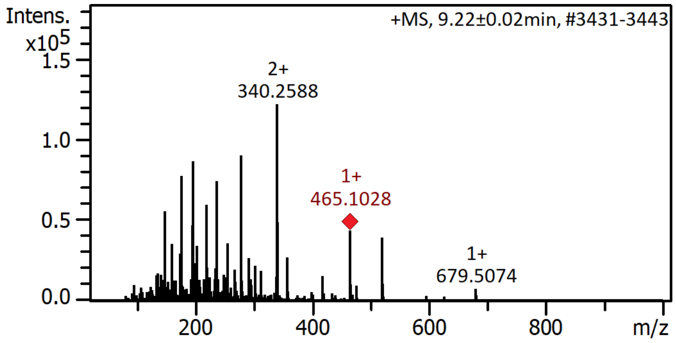

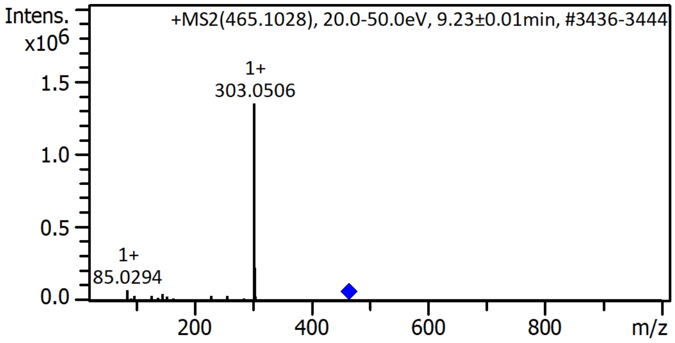

#	m/z	Res.	S/N	I	I %	FWHM
1	147.0438	9638	4642.2	55706	45.7	0.0153
2	177.0547	10460	6447.0	77364	63.4	0.0169
3	195.0646	10063	3895.9	46751	38.3	0.0194
4	197.1168	10433	7206.2	86474	70.9	0.0189
5	219.1736	10897	4964.0	59568	48.8	0.0201
6	237.1842	10138	6190.1	74281	60.9	0.0234
7	279.1619	10786	7515.9	90190	73.9	0.0259
8	340.2588	12758	10164.6	121975	100.0	0.0267
9	340.7606	12348	4088.1	49057	40.2	0.0276
10	465.1028	9988	3659.8	43917	36.0	0.0466

#	m/z	Res.	S/N	I	I %	FWHM
1	85.0294	8050	4571.5	71620	5.3	0.0106
2	97.0293	8013	1965.5	30792	2.3	0.0121
3	127.0397	9204	2122.9	33259	2.5	0.0138
4	145.0507	9748	2904.1	45497	3.4	0.0149
5	153.0190	9235	1777.3	27845	2.1	0.0166
6	229.0509	9846	2216.3	34722	2.6	0.0233
7	257.0464	10056	1982.2	31055	2.3	0.0256
8	303.0506	9150	86079.1	1348572	100.0	0.0331
9	304.0557	10132	14368.7	225109	16.7	0.0300
10	305.0576	10686	1687.7	26441	2.0	0.0285

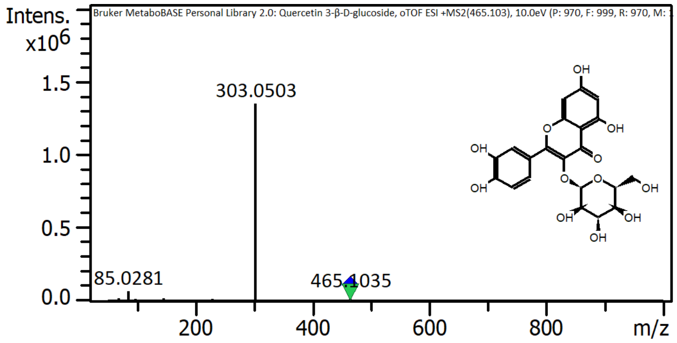

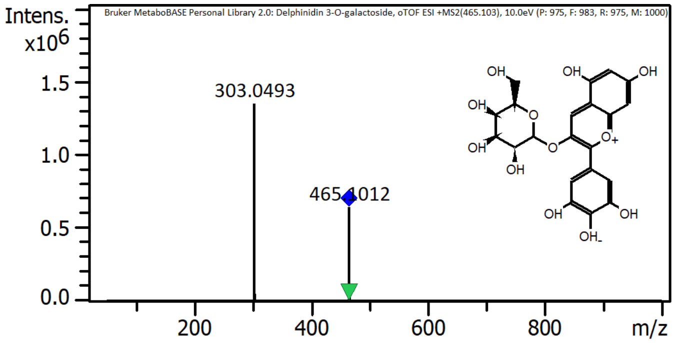

#	m/z	Res.	S/N	I	I %	FWHM
1	69.0336	2107	3.0	20229	1.5	0.0328
2	85.0281	2595	9.8	66080	4.9	0.0328
3	97.0274	2962	2.4	16183	1.2	0.0328
4	137.0234	4182	1.4	9440	0.7	0.0328
5	145.0485	4427	3.2	21577	1.6	0.0328
6	153.0177	4671	1.6	10789	0.8	0.0328
7	165.0183	5037	1.4	9440	0.7	0.0328
8	229.0514	6991	2.0	13486	1.0	0.0328
9	303.0503	9250	199.8	1347224	100.0	0.0328
10	465.1035	14196	7.4	49897	3.7	0.0328

#	m/z	Res.	S/N	I	I %	FWHM
1	303.0493	9250	199.8	1347224	100.0	0.0328
2	303.2098	9255	3.2	21577	1.6	0.0328
3	303.2921	9257	1.6	10789	0.8	0.0328
4	303.3422	9259	1.2	8091	0.6	0.0328
5	304.0507	9281	2.0	13486	1.0	0.0328
6	304.0557	9281	7.4	49897	3.7	0.0328
7	465.1012	14196	95.8	645966	47.9	0.0328
8	465.2854	14202	3.0	20229	1.5	0.0328
9	465.3525	14204	1.6	10789	0.8	0.0328
10	466.1037	14227	5.2	35063	2.6	0.0328

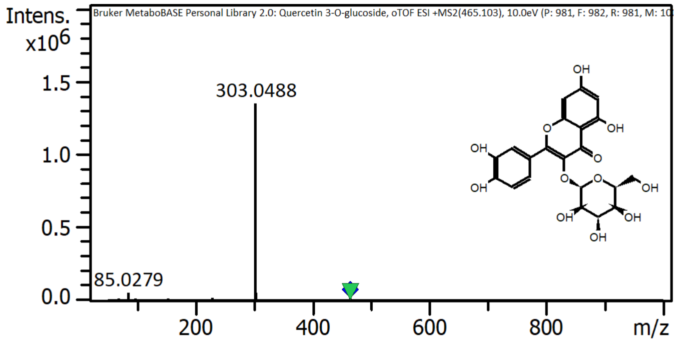

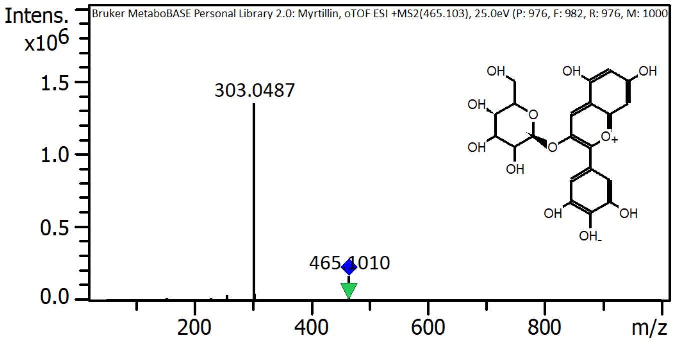

Compound Spectrum List Report

#	m/z	Res.	S/N	I	I %	FWHM
1	69.0327	2107	2.2	14834	1.1	0.0328
2	85.0279	2595	8.0	53943	4.0	0.0328
3	97.0270	2962	2.2	14834	1.1	0.0328
4	127.0393	3878	1.8	12137	0.9	0.0328
5	153.0176	4671	2.0	13486	1.0	0.0328
6	229.0479	6991	2.8	18880	1.4	0.0328
7	303.0488	9250	199.8	1347224	100.0	0.0328
8	303.1999	9255	4.0	26971	2.0	0.0328
9	304.0538	9281	7.8	52594	3.9	0.0328
10	465.1035	14196	2.2	14834	1.1	0.0328

#	m/z	Res.	S/N	I	I %	FWHM
1	153.0138	4670	2.0	13486	1.0	0.0328
2	205.0448	6259	1.6	10789	0.8	0.0328
3	229.0498	6991	2.2	14834	1.1	0.0328
4	257.0447	7846	5.4	36411	2.7	0.0328
5	303.0487	9250	199.8	1347224	100.0	0.0328
6	303.2046	9255	3.8	25623	1.9	0.0328
7	303.2805	9257	1.8	12137	0.9	0.0328
8	303.5001	9264	1.4	9440	0.7	0.0328
9	304.0490	9281	6.8	45851	3.4	0.0328
10	465.1010	14196	24.8	167223	12.4	0.0328

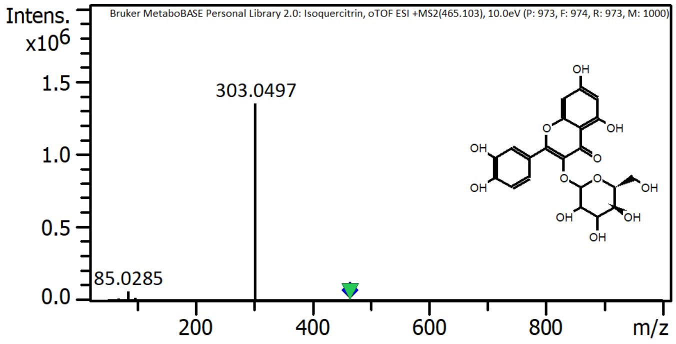

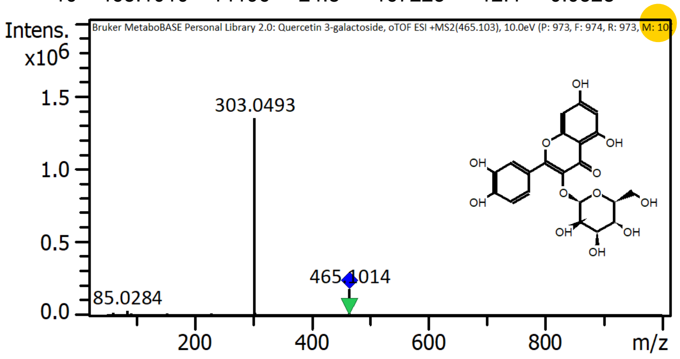

#	m/z	Res.	S/N	I	I %	FWHM
1	69.0335	2107	2.0	13486	1.0	0.0328
2	85.0285	2595	9.4	63383	4.7	0.0328
3	97.0291	2962	2.6	17531	1.3	0.0328
4	153.0170	4671	1.8	12137	0.9	0.0328
5	229.0512	6991	1.8	12137	0.9	0.0328
6	257.0448	7846	1.6	10789	0.8	0.0328
7	303.0497	9250	199.8	1347224	100.0	0.0328
8	303.2006	9255	2.2	14834	1.1	0.0328
9	304.0534	9281	1.8	12137	0.9	0.0328
10	465.1006	14196	1.8	12137	0.9	0.0328

#	m/z	Res.	S/N	I	I %	FWHM
1	61.0272	1863	3.2	21577	1.6	0.0328
2	85.0282	2595	3.2	21577	1.6	0.0328
3	85.0284	2595	4.6	31017	2.3	0.0328
4	85.0286	2595	3.0	20229	1.5	0.0328
5	91.0394	2779	2.4	16183	1.2	0.0328
6	229.0473	6991	2.4	16183	1.2	0.0328
7	303.0493	9250	199.8	1347224	100.0	0.0328
8	303.2047	9255	2.8	18880	1.4	0.0328
9	304.0505	9281	2.6	17531	1.3	0.0328
10	465.1014	14196	27.0	182057	13.5	0.0328

Cmpd 783, AutoMSn(203.1814), 9.27 min

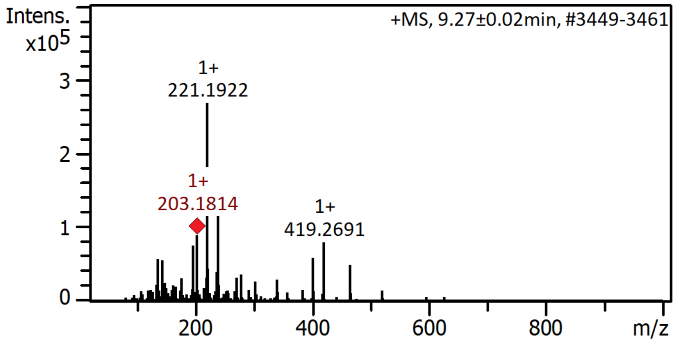

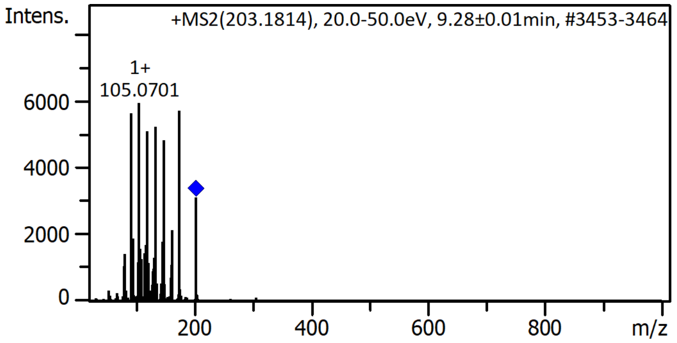

#	m/z	Res.	S/N	I	I %	FWHM
1	135.1180	8810	4840.4	58085	21.7	0.0153
2	143.1085	9057	4647.0	55764	20.8	0.0158
3	197.1183	9370	6354.2	76250	28.4	0.0210
4	203.1814	9834	7519.1	90229	33.6	0.0207
5	221.1922	9175	22350.4	268205	100.0	0.0241
6	222.1954	9979	3650.4	43805	16.3	0.0223
7	239.2031	10102	12321.3	147856	55.1	0.0237
8	401.2588	9750	4923.8	59086	22.0	0.0412
9	419.2691	10285	6663.2	79959	29.8	0.0408
10	465.1047	10686	4083.5	49002	18.3	0.0435

#	m/z	Res.	S/N	I	I %	FWHM
1	91.0528	8623	358.9	5623	94.8	0.0106
2	95.0859	7594	120.1	1881	31.7	0.0125
3	105.0701	8209	378.7	5932	100.0	0.0128
4	119.0855	8760	325.2	5095	85.9	0.0136
5	133.1013	8938	334.0	5233	88.2	0.0149
6	145.1009	9774	114.0	1786	30.1	0.0148
7	147.1164	8618	308.1	4827	81.4	0.0171
8	161.1324	9059	137.1	2148	36.2	0.0178
9	175.0359	9357	364.4	5710	96.2	0.0187
10	203.1776	10306	198.4	3109	52.4	0.0197

Compound Spectrum List Report

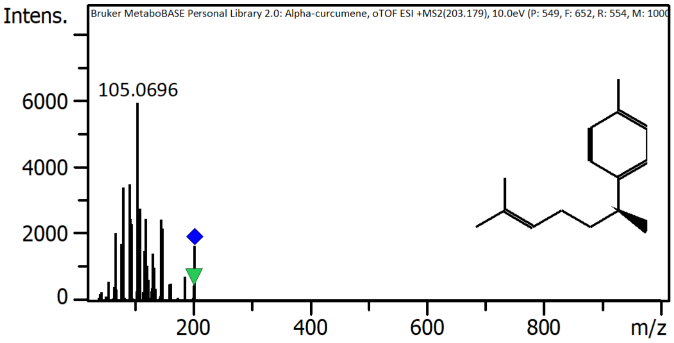

#	m/z	Res.	S/N	I	I %	FWHM
1	81.0697	7059	114.8	3405	57.5	0.0115
2	91.0545	7929	118.0	3500	59.1	0.0115
3	93.0690	8104	82.8	2456	41.4	0.0115
4	95.0851	8280	78.0	2314	39.0	0.0115
5	105.0696	9149	199.8	5926	100.0	0.0115
6	107.0844	9325	93.4	2770	46.7	0.0115
7	109.1014	9500	93.4	2770	46.7	0.0115
8	119.0845	10370	82.8	2456	41.4	0.0115
9	145.0992	12635	82.2	2438	41.1	0.0115
10	147.1150	12810	73.2	2171	36.6	0.0115

Cmpd 803, AutoMSn(211.1325), 9.45 min

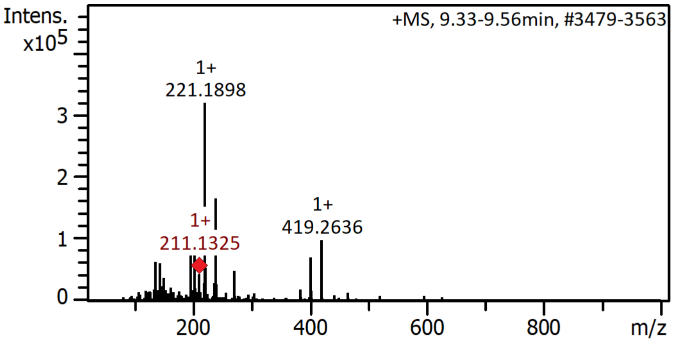

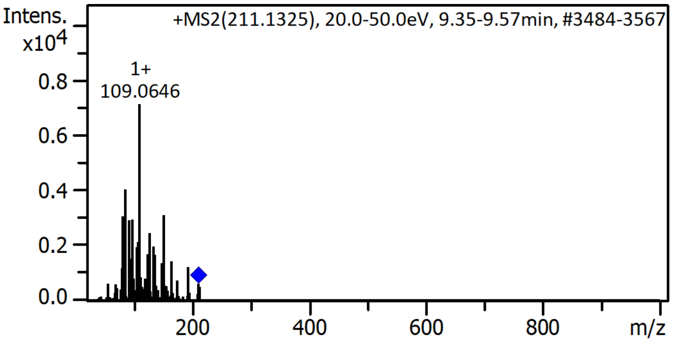

#	m/z	Res.	S/N	I	I %	FWHM
1	135.1166	10277	8812.7	63452	19.9	0.0131
2	143.1065	10325	8594.9	61884	19.4	0.0139
3	197.1174	10881	10892.4	78426	24.6	0.0181
4	203.1791	11162	16506.9	118850	37.2	0.0182
5	221.1898	10854	44349.6	319317	100.0	0.0204
6	222.1933	11283	7376.8	53113	16.6	0.0197
7	239.2004	11209	23028.4	165804	51.9	0.0213
8	271.1360	11117	6694.3	48199	15.1	0.0244
9	401.2531	12181	9816.8	70681	22.1	0.0329
10	419.2636	12358	13748.8	98991	31.0	0.0339

#	m/z	Res.	S/N	I	I %	FWHM
1	81.0700	7486	319.9	3071	43.2	0.0108
2	83.0498	9317	291.9	2803	39.4	0.0089
3	85.0645	9476	420.9	4040	56.8	0.0090
4	91.0542	9244	305.3	2931	41.2	0.0098
5	97.0647	9523	308.6	2963	41.6	0.0102
6	107.0850	8777	222.7	2138	30.0	0.0122
7	109.0646	9488	741.2	7116	100.0	0.0115
8	127.0751	10425	258.0	2476	34.8	0.0122
9	133.1005	11181	207.3	1990	28.0	0.0119
10	151.1110	11175	325.2	3122	43.9	0.0135

Compound Spectrum List Report

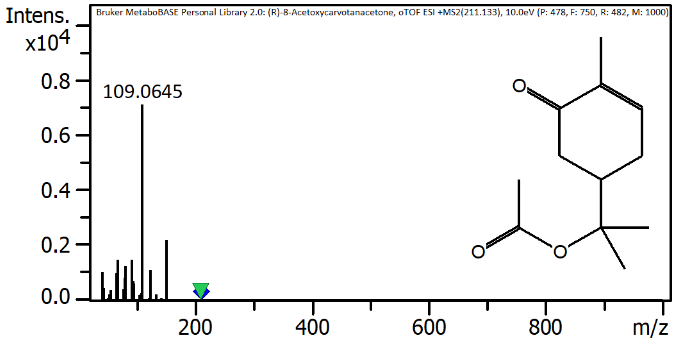

#	m/z	Res.	S/N	I	I %	FWHM
1	41.0391	3761	29.2	1039	14.6	0.0109
2	65.0384	5960	27.4	975	13.7	0.0109
3	67.0548	6145	42.0	1494	21.0	0.0109
4	79.0545	7244	23.4	833	11.7	0.0109
5	81.0700	7429	35.0	1245	17.5	0.0109
6	91.0541	8344	41.8	1487	20.9	0.0109
7	109.0645	9994	199.8	7108	100.0	0.0109
8	109.0964	9997	73.8	2626	36.9	0.0109
9	123.0795	11279	30.8	1096	15.4	0.0109
10	151.1114	13847	62.4	2220	31.2	0.0109

Cmpd 805, AutoMSn(153.1269), 9.48 min

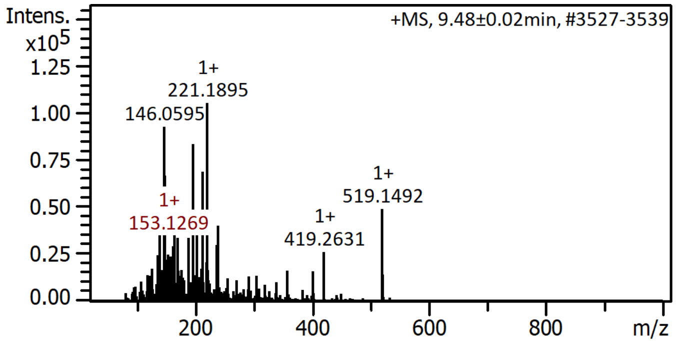

#	m/z	Res.	S/N	I	I %	FWHM
1	139.1113	10351	4886.1	58634	55.8	0.0134
2	146.0595	10526	7720.7	92648	88.2	0.0139
3	147.0443	10171	5559.2	66711	63.5	0.0145
4	165.0542	10735	3110.6	37327	35.5	0.0154
5	197.1163	11317	6960.9	83530	79.5	0.0174
6	203.1786	11777	3174.5	38094	36.3	0.0173
7	213.1478	11647	5742.8	68913	65.6	0.0183
8	221.1895	11588	8753.2	105038	100.0	0.0191
9	239.1997	12199	3352.9	40235	38.3	0.0196
10	519.1492	11741	4098.4	49181	46.8	0.0442

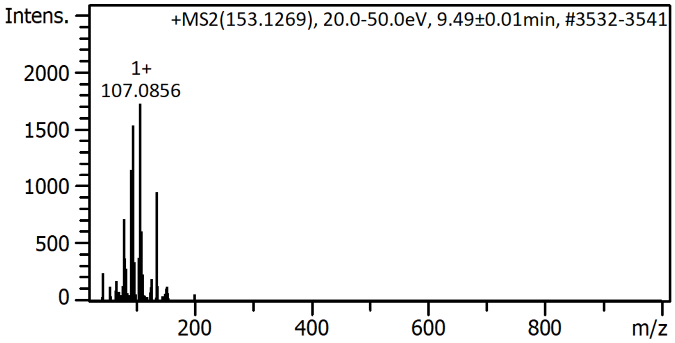

#	m/z	Res.	S/N	I	I %	FWHM
1	79.0545	8132	42.2	718	41.7	0.0097
2	81.0698	9973	22.3	379	22.0	0.0081
3	91.0540	8695	67.5	1148	66.7	0.0105
4	93.0704	9824	65.9	1121	65.1	0.0095
5	95.0860	8313	90.3	1534	89.1	0.0114
6	97.0664	9835	20.4	346	20.1	0.0099
7	105.0686	7675	22.5	383	22.2	0.0137
8	107.0856	10332	101.3	1722	100.0	0.0104
9	109.1002	10495	36.1	613	35.6	0.0104
10	135.1157	9988	56.0	952	55.3	0.0135

Compound Spectrum List Report

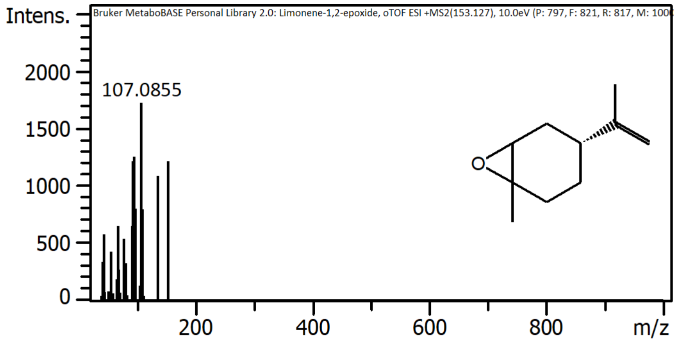

#	m/z	Res.	S/N	I	I %	FWHM
1	43.0190	4038	67.8	584	33.9	0.0107
2	67.0548	6294	76.2	656	38.1	0.0107
3	91.0542	8547	75.8	653	37.9	0.0107
4	93.0702	8736	141.2	1216	70.7	0.0107
5	95.0856	8926	145.8	1256	73.0	0.0107
6	97.0651	9111	93.6	806	46.8	0.0107
7	107.0855	10052	199.8	1721	100.0	0.0107
8	109.1017	10241	92.8	799	46.4	0.0107
9	135.1167	12683	126.4	1089	63.3	0.0107
10	153.1271	14374	141.0	1214	70.6	0.0107

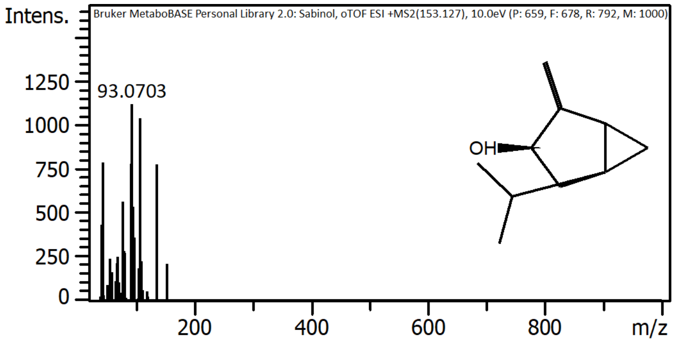

#	m/z	Res.	S/N	I	I %	FWHM
1	41.0394	3852	77.6	435	38.8	0.0107
2	43.0552	4042	140.6	788	70.4	0.0107
3	77.0387	7232	100.6	564	50.4	0.0107
4	79.0545	7421	51.2	287	25.6	0.0107
5	91.0541	8547	139.2	780	69.7	0.0107
6	93.0703	8736	199.8	1120	100.0	0.0107
7	95.0852	8926	95.6	536	47.8	0.0107
8	97.0647	9111	64.4	361	32.2	0.0107
9	107.0848	10052	185.0	1037	92.6	0.0107
10	135.1154	12683	138.4	776	69.3	0.0107

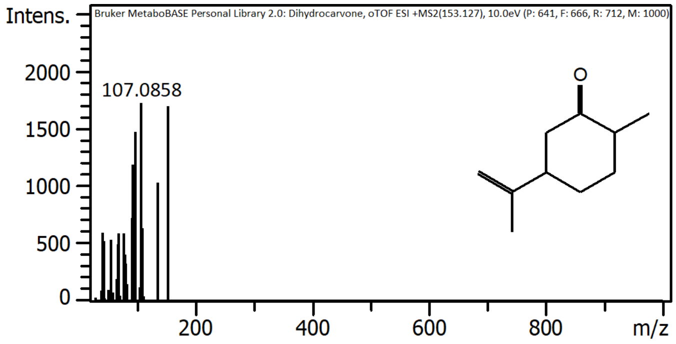

#	m/z	Res.	S/N	I	I %	FWHM
1	41.0400	3852	69.2	596	34.6	0.0107
2	69.0705	6484	68.8	592	34.4	0.0107
3	91.0547	8547	84.0	723	42.0	0.0107
4	93.0704	8736	137.8	1187	69.0	0.0107
5	95.0860	8926	88.6	763	44.3	0.0107
6	97.0651	9111	170.6	1469	85.4	0.0107
7	107.0858	10052	199.8	1721	100.0	0.0107
8	109.1011	10241	73.8	636	36.9	0.0107
9	135.1169	12683	119.8	1032	60.0	0.0107
10	153.1274	14374	197.0	1696	98.6	0.0107

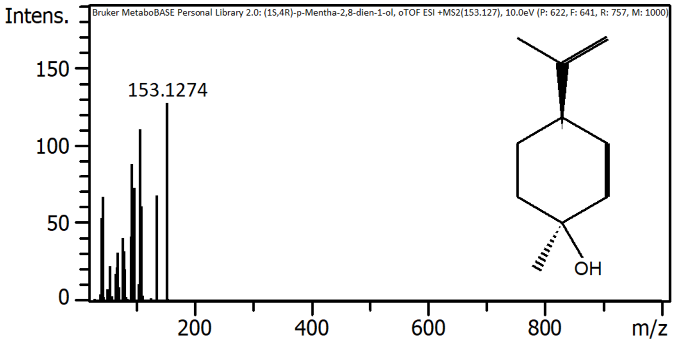

#	m/z	Res.	S/N	I	I %	FWHM
1	41.0397	3852	84.2	54	42.1	0.0107
2	43.0186	4038	105.8	67	53.0	0.0107
3	93.0699	8736	138.8	88	69.5	0.0107
4	95.0853	8926	103.0	66	51.6	0.0107
5	95.0854	8926	101.2	64	50.7	0.0107
6	97.0648	9111	114.6	73	57.4	0.0107
7	107.0856	10052	173.2	110	86.7	0.0107
8	109.1015	10241	95.8	61	47.9	0.0107
9	135.1169	12683	106.8	68	53.5	0.0107
10	153.1274	14374	199.8	127	100.0	0.0107

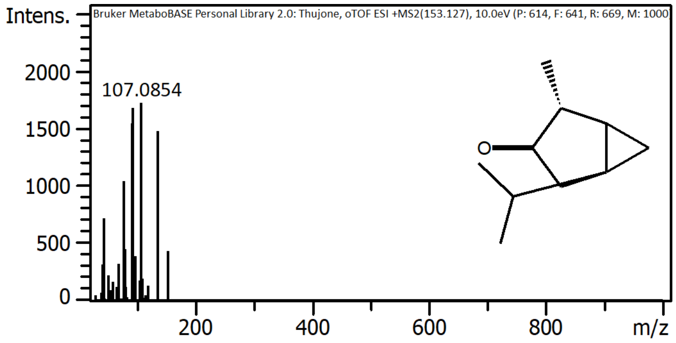

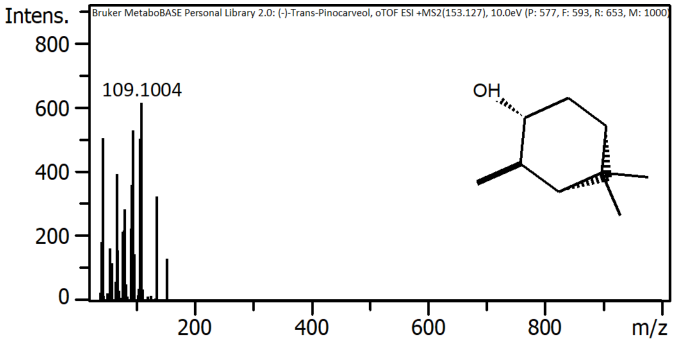

Compound Spectrum List Report

#	m/z	Res.	S/N	I	I %	FWHM
1	43.0183	4038	25.8	400	23.2	0.0107
2	43.0554	4042	46.4	720	41.8	0.0107
3	77.0392	7232	67.3	1044	60.7	0.0107
4	79.0547	7421	29.3	455	26.4	0.0107
5	91.0543	8547	99.7	1545	89.8	0.0107
6	93.0699	8736	108.2	1678	97.5	0.0107
7	97.0654	9111	25.4	394	22.9	0.0107
8	107.0854	10052	111.0	1721	100.0	0.0107
9	135.1174	12683	95.1	1474	85.7	0.0107
10	153.1277	14374	28.2	437	25.4	0.0107

#	m/z	Res.	S/N	I	I %	FWHM
1	43.0184	4038	164.4	504	82.3	0.0107
2	67.0543	6294	128.2	393	64.2	0.0107
3	79.0546	7421	71.6	219	35.8	0.0107
4	81.0692	7610	92.6	284	46.3	0.0107
5	91.0546	8547	73.2	224	36.6	0.0107
6	93.0700	8736	117.6	360	58.9	0.0107
7	95.0860	8926	172.0	527	86.1	0.0107
8	107.0861	10052	164.0	503	82.1	0.0107
9	109.1004	10241	199.8	612	100.0	0.0107
10	135.1162	12683	105.8	324	53.0	0.0107

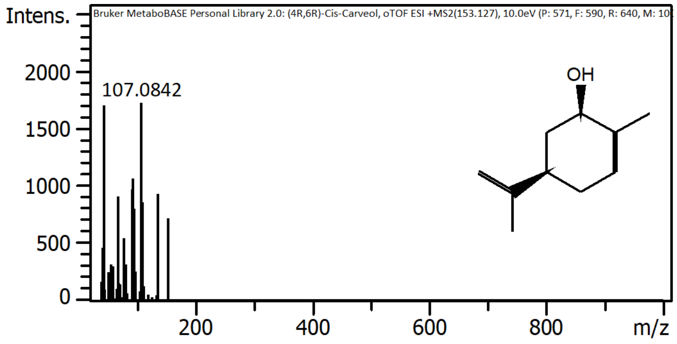

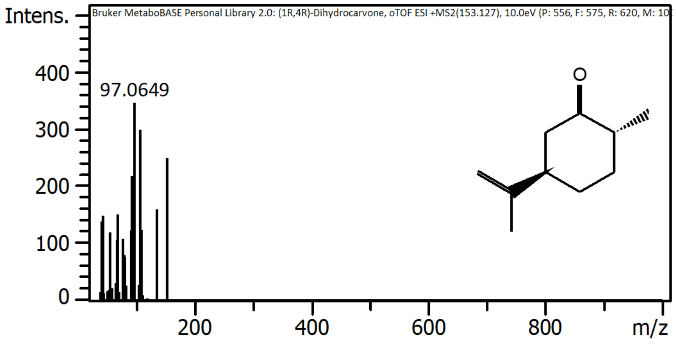

#	m/z	Res.	S/N	I	I %	FWHM
1	43.0180	4038	105.6	1455	84.6	0.0107
2	43.0181	4038	123.4	1700	98.8	0.0107
3	67.0537	6294	65.9	908	52.8	0.0107
4	91.0537	8547	70.3	968	56.3	0.0107
5	93.0688	8736	77.3	1064	61.9	0.0107
6	95.0838	8925	58.3	803	46.6	0.0107
7	107.0842	10052	124.9	1721	100.0	0.0107
8	109.1001	10241	62.4	859	49.9	0.0107
9	135.1153	12683	67.6	932	54.2	0.0107
10	153.1251	14374	52.3	720	41.8	0.0107

#	m/z	Res.	S/N	I	I %	FWHM
1	41.0396	3852	80.6	139	40.3	0.0107
2	43.0187	4038	86.2	149	43.1	0.0107
3	69.0702	6484	87.8	152	43.9	0.0107
4	93.0699	8736	126.2	218	63.2	0.0107
5	95.0856	8926	81.4	141	40.7	0.0107
6	97.0649	9111	199.8	346	100.0	0.0107
7	107.0853	10052	172.6	299	86.4	0.0107
8	109.1008	10241	72.0	125	36.0	0.0107
9	135.1166	12683	92.6	160	46.3	0.0107
10	153.1271	14374	144.0	249	72.1	0.0107

Cmpd 811, AutoMSn(165.0543), 9.53 min

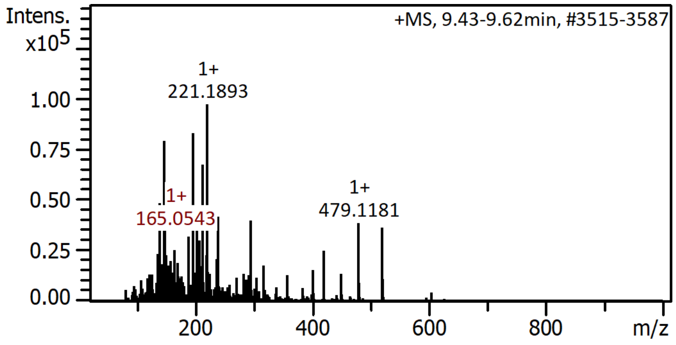

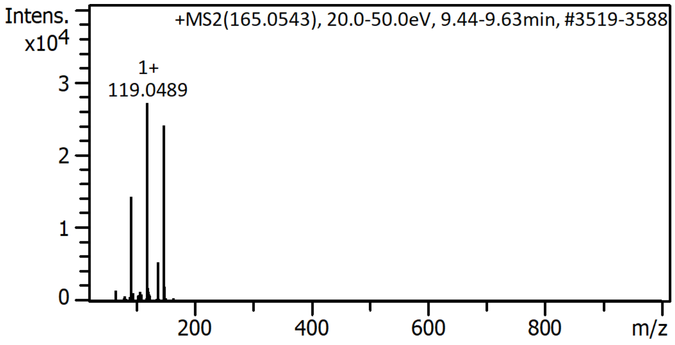

#	m/z	Res.	S/N	I	I %	FWHM
1	139.1113	10296	6750.3	48602	50.0	0.0135
2	146.0597	10600	11009.2	79266	81.6	0.0138
3	147.0446	10532	6518.5	46933	48.3	0.0140
4	197.1169	11556	11510.9	82878	85.3	0.0171
5	203.1790	12155	5542.6	39907	41.1	0.0167
6	213.1481	11396	9351.2	67329	69.3	0.0187
7	221.1893	11632	13494.6	97161	100.0	0.0190
8	239.2002	11737	5816.1	41876	43.1	0.0204
9	295.1018	11655	5561.4	40042	41.2	0.0253
10	479.1181	13469	5369.6	38661	39.8	0.0356

#	m/z	Res.	S/N	I	I %	FWHM
1	65.0378	8969	152.1	1460	5.4	0.0073
2	91.0541	9181	1496.3	14365	53.0	0.0099
3	107.0867	8991	135.1	1297	4.8	0.0119
4	119.0489	10340	2821.7	27089	100.0	0.0115
5	119.0869	8648	121.2	1164	4.3	0.0138
6	120.0536	11253	183.3	1760	6.5	0.0107
7	121.1010	10114	133.0	1276	4.7	0.0120
8	137.0956	11217	563.1	5406	20.0	0.0122
9	147.0439	10831	2502.2	24021	88.7	0.0136
10	148.0461	11003	207.2	1990	7.3	0.0135

Compound Spectrum List Report

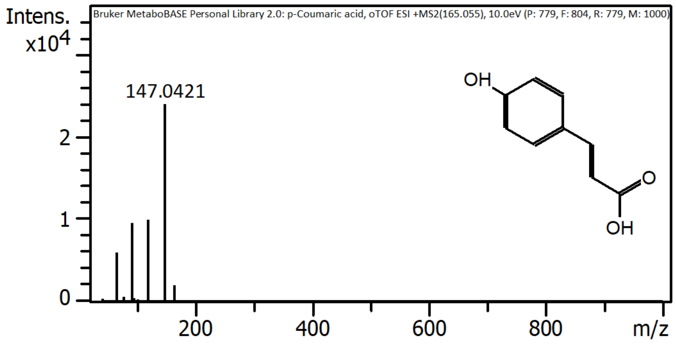

#	m/z	Res.	S/N	I	I %	FWHM
1	65.0376	5606	3.7	625	2.6	0.0116
2	65.0395	5606	35.7	6005	25.0	0.0116
3	91.0513	7849	7.7	1297	5.4	0.0116
4	91.0545	7849	56.9	9561	39.8	0.0116
5	119.0489	10262	59.4	9993	41.6	0.0116
6	119.0503	10262	18.4	3099	12.9	0.0116
7	147.0421	12675	142.7	23997	100.0	0.0116
8	147.0455	12676	32.3	5429	22.6	0.0116
9	164.9162	14216	11.6	1946	8.1	0.0116
10	165.0509	14228	6.1	1033	4.3	0.0116

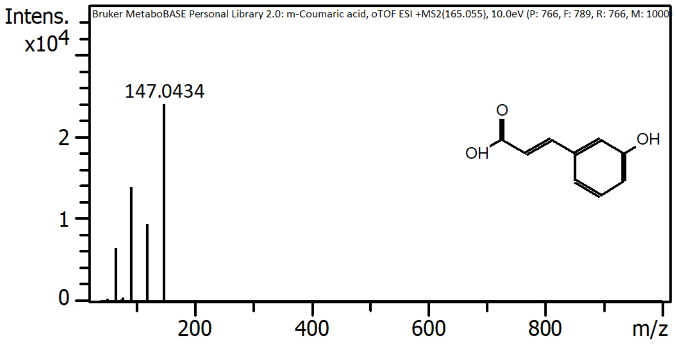

#	m/z	Res.	S/N	I	I %	FWHM
1	41.0374	3537	1.8	216	0.9	0.0116
2	51.0216	4398	2.6	312	1.3	0.0116
3	65.0383	5606	54.8	6582	27.4	0.0116
4	75.0213	6467	2.4	288	1.2	0.0116
5	77.0383	6641	4.2	504	2.1	0.0116
6	91.0538	7849	116.4	13980	58.3	0.0116
7	101.0376	8710	1.8	216	0.9	0.0116
8	103.0502	8883	1.4	168	0.7	0.0116
9	119.0481	10262	78.6	9440	39.3	0.0116
10	147.0434	12675	199.8	23997	100.0	0.0116

Cmpd 817, AutoMSn(479.1176), 9.59 min

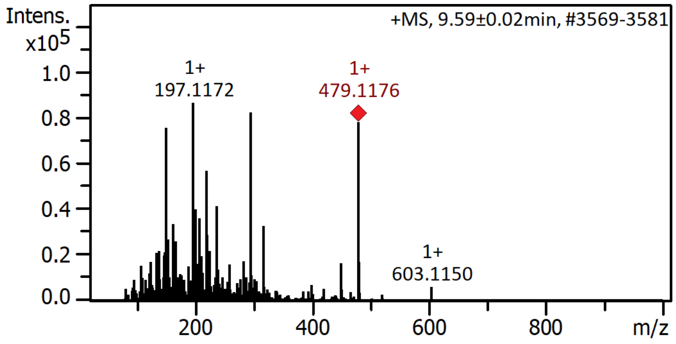

#	m/z	Res.	S/N	I	I %	FWHM
1	149.1324	10671	6302.8	75633	87.6	0.0140
2	163.0480	10167	2798.7	33584	38.9	0.0160
3	197.1172	11551	7192.4	86308	100.0	0.0171
4	201.1637	11489	3333.7	40005	46.4	0.0175
5	207.1381	11243	3006.6	36079	41.8	0.0184
6	219.1745	11900	4729.8	56758	65.8	0.0184
7	237.1850	11919	3462.6	41552	48.1	0.0199
8	295.1021	12118	6843.7	82124	95.2	0.0244
9	317.0655	12464	2724.1	32689	37.9	0.0254
10	479.1176	13467	6496.8	77962	90.3	0.0356

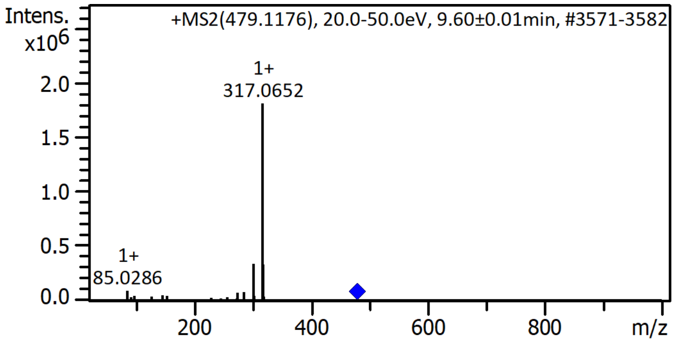

#	m/z	Res.	S/N	I	I %	FWHM
1	85.0286	8042	5701.6	89325	4.9	0.0106
2	97.0283	9397	2863.9	44868	2.5	0.0103
3	145.0492	10565	3116.0	48817	2.7	0.0137
4	153.0179	10826	2901.0	45448	2.5	0.0141
5	274.0475	11559	4542.2	71161	3.9	0.0237
6	285.0395	12015	5130.4	80377	4.4	0.0237
7	302.0420	12092	21360.1	334642	18.5	0.0250
8	303.0457	12044	2833.6	44392	2.4	0.0252
9	317.0652	10962	115656.3	1811949	100.0	0.0289
10	318.0689	11740	21241.4	332782	18.4	0.0271

Compound Spectrum List Report

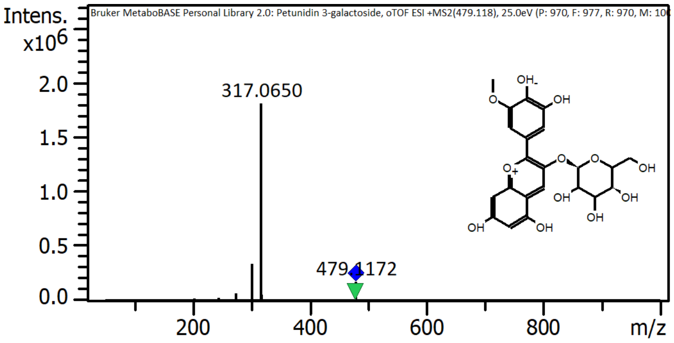

#	m/z	Res.	S/N	I	I %	FWHM
1	203.0368	7138	2.0	18119	1.0	0.0284
2	245.0446	8615	3.2	28991	1.6	0.0284
3	246.0517	8650	1.6	14496	0.8	0.0284
4	274.0453	9634	7.4	67042	3.7	0.0284
5	302.0403	10619	37.0	335211	18.5	0.0284
6	317.0650	11147	199.8	1810137	100.0	0.0284
7	317.2299	11153	3.4	30803	1.7	0.0284
8	318.0660	11182	6.4	57982	3.2	0.0284
9	318.0676	11182	1.8	16308	0.9	0.0284
10	479.1172	16844	19.0	172135	9.5	0.0284

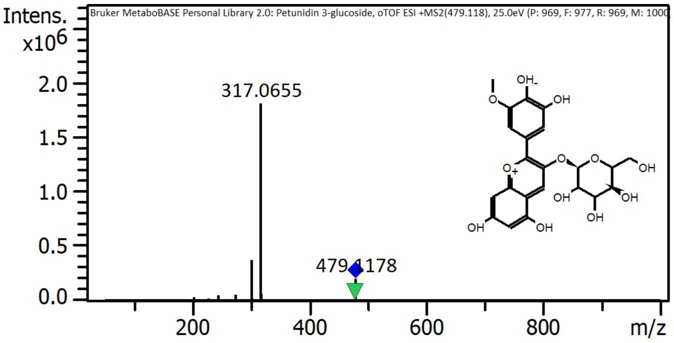

#	m/z	Res.	S/N	I	I %	FWHM
1	203.0339	7138	3.4	30803	1.7	0.0284
2	228.0370	8017	2.2	19931	1.1	0.0284
3	245.0415	8615	5.8	52547	2.9	0.0284
4	274.0498	9635	6.0	54358	3.0	0.0284
5	302.0436	10619	42.0	380509	21.0	0.0284
6	302.1861	10624	1.6	14496	0.8	0.0284
7	303.0350	10654	2.0	18119	1.0	0.0284
8	317.0655	11147	199.8	1810137	100.0	0.0284
9	318.0699	11182	6.6	59794	3.3	0.0284
10	479.1178	16844	22.6	204750	11.3	0.0284

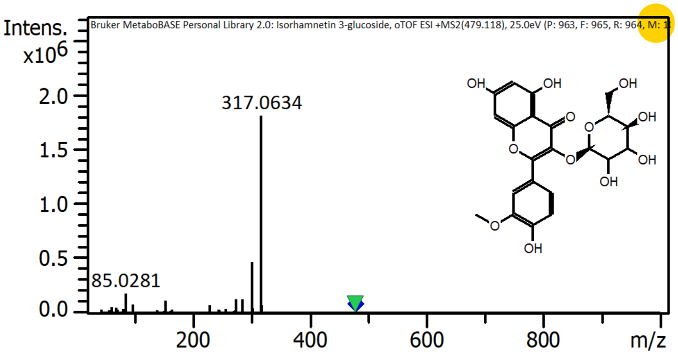

#	m/z	Res.	S/N	I	I %	FWHM
1	61.0292	2146	6.2	56170	3.1	0.0284
2	85.0281	2989	20.0	181195	10.0	0.0284
3	97.0252	3411	8.6	77914	4.3	0.0284
4	153.0153	5379	12.4	112341	6.2	0.0284
5	229.0428	8052	7.8	70666	3.9	0.0284
6	274.0455	9634	14.2	128648	7.1	0.0284
7	285.0384	10021	13.8	125025	6.9	0.0284
8	302.0396	10619	52.2	472919	26.1	0.0284
9	317.0634	11147	199.8	1810137	100.0	0.0284
10	318.0669	11182	8.0	72478	4.0	0.0284

Cmpd 818, AutoMSn(449.1078), 9.59 min

Compound Spectrum List Report

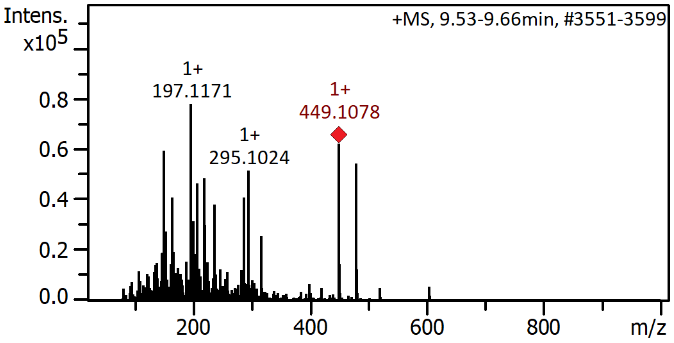

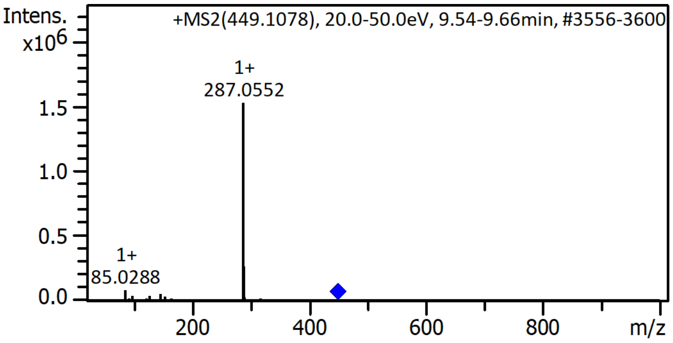

#	m/z	Res.	S/N	I	I %	FWHM
1	149.1323	10713	6611.5	59504	76.4	0.0139
2	165.1274	10707	4533.2	40799	52.4	0.0154
3	197.1171	11337	8657.8	77920	100.0	0.0174
4	207.1380	11186	5161.6	46455	59.6	0.0185
5	219.1740	11810	5379.9	48419	62.1	0.0186
6	237.1842	12195	4244.0	38196	49.0	0.0194
7	287.0555	12489	4544.6	40901	52.5	0.0230
8	295.1024	11824	5727.2	51545	66.2	0.0250
9	449.1078	12694	6915.2	62237	79.9	0.0354
10	479.1181	13186	6023.4	54211	69.6	0.0363

#	m/z	Res.	S/N	I	I %	FWHM
1	85.0288	8084	6924.4	81361	5.3	0.0105
2	97.0289	9273	3290.0	38658	2.5	0.0105
3	121.0287	9654	1525.0	17919	1.2	0.0125
4	127.0396	10263	3353.3	39401	2.6	0.0124
5	145.0504	9991	4383.6	51508	3.4	0.0145
6	153.0184	10627	2893.3	33996	2.2	0.0144
7	165.0183	11097	1472.2	17299	1.1	0.0149
8	287.0552	10878	129925.6	1526626	100.0	0.0264
9	288.0593	11657	22494.9	264315	17.3	0.0247
10	289.0612	11284	2390.9	28093	1.8	0.0256

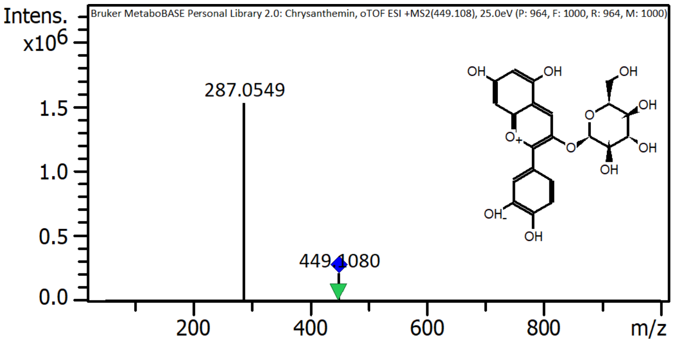

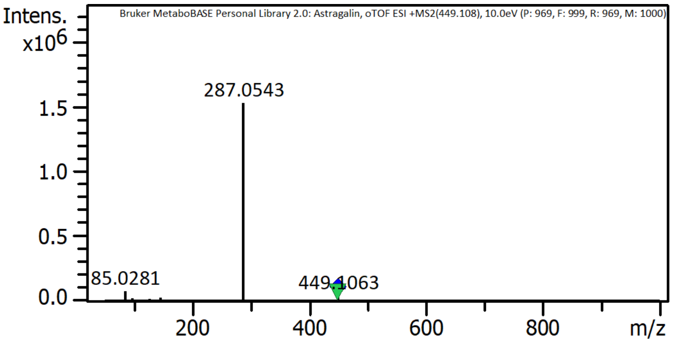

#	m/z	Res.	S/N	I	I %	FWHM
1	213.0530	8048	1.0	12213	0.8	0.0265
2	287.0549	10843	124.9	1525099	100.0	0.0265
3	449.1080	16964	17.5	213728	14.0	0.0265

#	m/z	Res.	S/N	I	I %	FWHM
1	69.0337	2608	1.5	13740	0.9	0.0265
2	85.0281	3212	8.2	74805	4.9	0.0265
3	97.0271	3665	1.8	16793	1.1	0.0265
4	97.0274	3665	2.5	22899	1.5	0.0265
5	127.0381	4799	1.7	15266	1.0	0.0265
6	145.0487	5479	3.2	29006	1.9	0.0265
7	153.0188	5780	1.0	9160	0.6	0.0265
8	287.0543	10843	166.5	1525099	100.0	0.0265
9	287.2057	10849	2.8	25953	1.7	0.0265
10	449.1063	16964	4.7	42746	2.8	0.0265

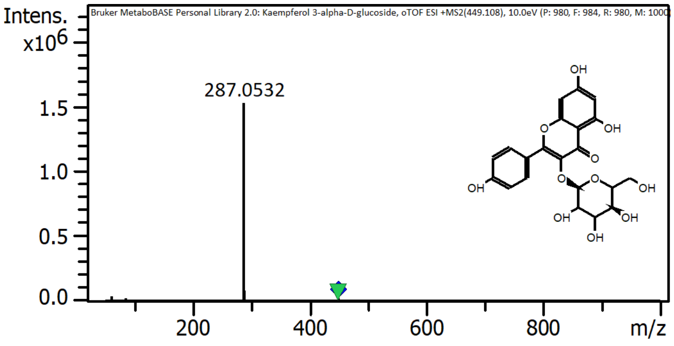

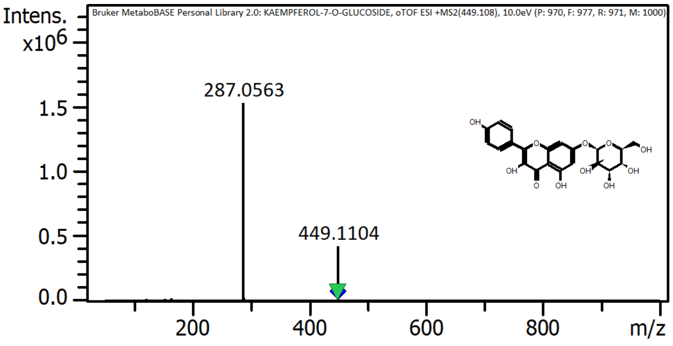

Compound Spectrum List Report

#	m/z	Res.	S/N	I	I %	FWHM
1	61.0282	2305	5.0	38166	2.5	0.0265
2	85.0287	3212	3.2	24426	1.6	0.0265
3	91.0382	3439	1.8	13740	0.9	0.0265
4	287.0532	10843	199.8	1525099	100.0	0.0265
5	287.2480	10850	2.0	15266	1.0	0.0265
6	287.2854	10852	1.8	13740	0.9	0.0265
7	287.3405	10854	1.4	10686	0.7	0.0265
8	287.4398	10858	2.4	18320	1.2	0.0265
9	288.0561	10881	10.8	82438	5.4	0.0265
10	449.0982	16964	2.6	19846	1.3	0.0265

#	m/z	Res.	S/N	I	I %	FWHM
1	121.0281	4572	2.0	15266	1.0	0.0265
2	153.0240	5780	2.4	18320	1.2	0.0265
3	165.0167	6233	2.6	19846	1.3	0.0265
4	287.0534	10843	66.6	508366	33.3	0.0265
5	287.0563	10843	199.8	1525099	100.0	0.0265
6	287.2001	10849	2.6	19846	1.3	0.0265
7	288.0556	10881	2.2	16793	1.1	0.0265
8	288.0632	10881	3.8	29006	1.9	0.0265
9	449.1104	16965	55.8	425929	27.9	0.0265
10	449.3516	16974	2.0	15266	1.0	0.0265

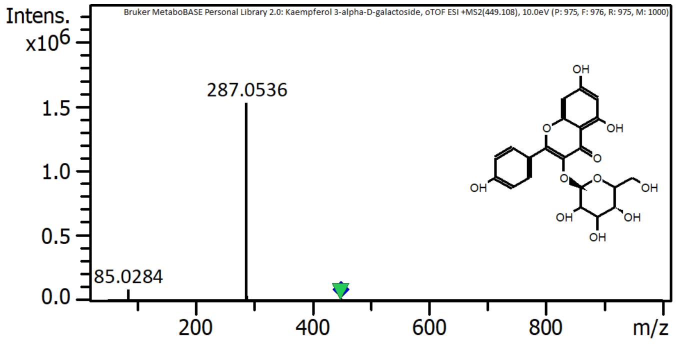

#	m/z	Res.	S/N	I	I %	FWHM
1	61.0271	2305	1.6	12213	0.8	0.0265
2	69.0320	2608	1.8	13740	0.9	0.0265
3	85.0284	3212	11.0	83964	5.5	0.0265
4	153.0168	5780	1.8	13740	0.9	0.0265
5	165.0177	6233	1.8	13740	0.9	0.0265
6	287.0536	10843	199.8	1525099	100.0	0.0265
7	287.2015	10849	5.6	42746	2.8	0.0265
8	287.2515	10851	2.4	18320	1.2	0.0265
9	288.0584	10881	4.8	36639	2.4	0.0265
10	449.1080	16964	2.4	18320	1.2	0.0265

Cmpd 821, AutoMSn(203.1788), 9.62 min

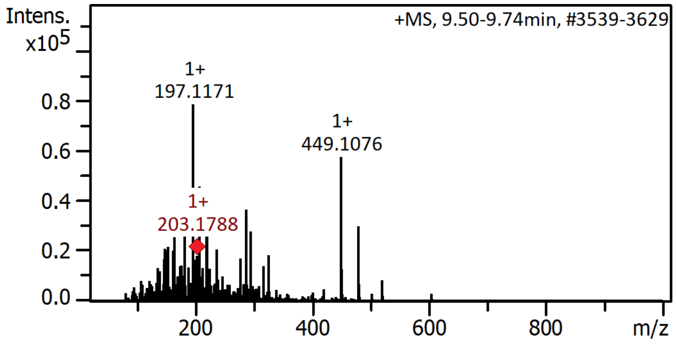

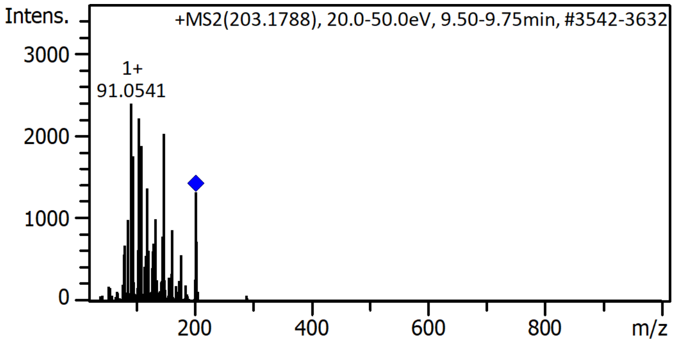

#	m/z	Res.	S/N	I	I %	FWHM
1	165.1272	10703	3577.5	25758	32.8	0.0154
2	182.1181	10747	4495.7	32369	41.2	0.0169
3	197.1171	11389	10918.3	78612	100.0	0.0173
4	207.1381	11157	6358.1	45778	58.2	0.0186
5	219.1740	11906	4890.6	35213	44.8	0.0184
6	221.1898	11259	3820.3	27506	35.0	0.0196
7	287.0553	12010	5101.1	36728	46.7	0.0239
8	295.0987	9428	3871.9	27877	35.5	0.0313
9	449.1076	12976	7989.6	57525	73.2	0.0346
10	479.1187	12659	4167.9	30009	38.2	0.0378

#	m/z	Res.	S/N	I	I %	FWHM
1	86.0970	7913	102.9	988	41.3	0.0109
2	91.0541	9887	249.3	2393	100.0	0.0092
3	95.0857	9562	183.0	1757	73.4	0.0099
4	105.0701	10639	230.5	2213	92.5	0.0099
5	107.0860	9357	117.2	1125	47.0	0.0114
6	109.1010	9709	196.0	1881	78.6	0.0112
7	119.0862	10230	142.8	1371	57.3	0.0116
8	133.1018	11584	103.7	995	41.6	0.0115
9	147.1169	8249	211.0	2026	84.7	0.0178
10	203.1816	12894	137.4	1319	55.1	0.0158

Compound Spectrum List Report

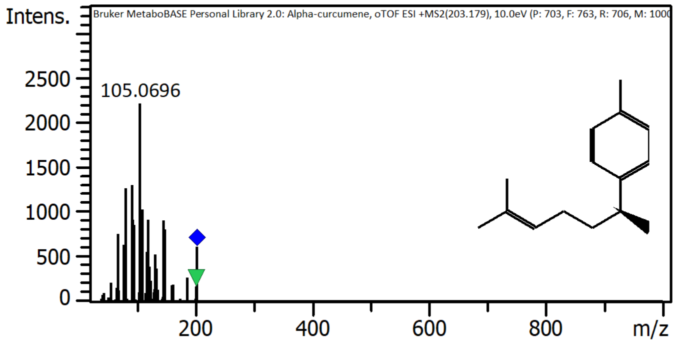

#	m/z	Res.	S/N	I	I %	FWHM
1	81.0697	9633	114.8	1270	57.5	0.0084
2	91.0545	10820	118.0	1305	59.1	0.0084
3	93.0690	11059	82.8	916	41.4	0.0084
4	95.0851	11299	78.0	863	39.0	0.0084
5	105.0696	12485	199.8	2210	100.0	0.0084
6	107.0844	12724	93.4	1033	46.7	0.0084
7	109.1014	12964	93.4	1033	46.7	0.0084
8	119.0845	14150	82.8	916	41.4	0.0084
9	145.0992	17241	82.2	909	41.1	0.0084
10	147.1150	17481	73.2	810	36.6	0.0084

Cmpd 833, AutoMSn(153.1275), 9.76 min

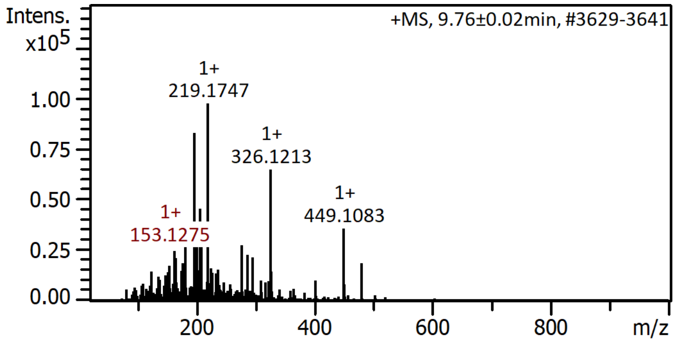

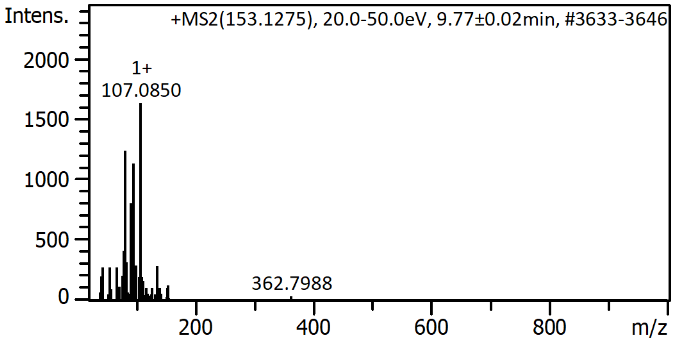

#	m/z	Res.	S/N	I	I %	FWHM
1	163.0401	10865	2065.8	24790	25.4	0.0150
2	182.1180	10689	2978.8	35746	36.6	0.0170
3	197.1175	11074	6907.3	82887	84.9	0.0178
4	201.1641	11599	2912.8	34954	35.8	0.0173
5	207.1384	10977	3812.3	45748	46.9	0.0189
6	209.1537	12148	2825.9	33911	34.7	0.0172
7	219.1747	11674	8136.0	97632	100.0	0.0188
8	277.0705	12234	2307.8	27693	28.4	0.0226
9	326.1213	12621	5416.9	65002	66.6	0.0258
10	449.1083	13775	2988.0	35856	36.7	0.0326

#	m/z	Res.	S/N	I	I %	FWHM
1	55.0541	5027	15.5	274	16.8	0.0110
2	79.0525	8928	23.5	416	25.5	0.0089
3	81.0704	7312	70.0	1237	75.9	0.0111
4	83.0487	10967	18.1	319	19.6	0.0076
5	91.0553	10931	45.5	803	49.3	0.0083
6	93.0698	8992	43.7	772	47.4	0.0104
7	95.0849	9374	64.1	1133	69.5	0.0101
8	98.9857	9309	16.4	290	17.8	0.0106
9	107.0850	6521	92.2	1630	100.0	0.0164
10	135.1220	11246	16.0	282	17.3	0.0120

Compound Spectrum List Report

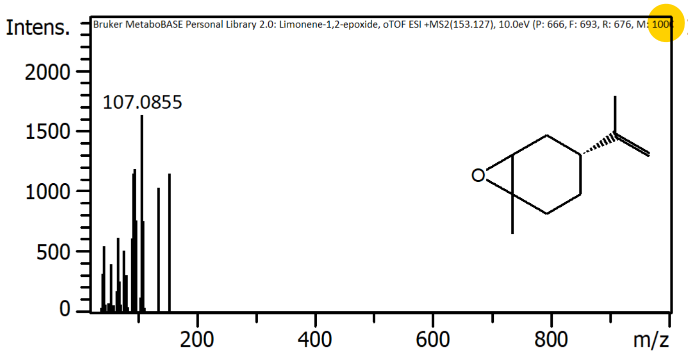

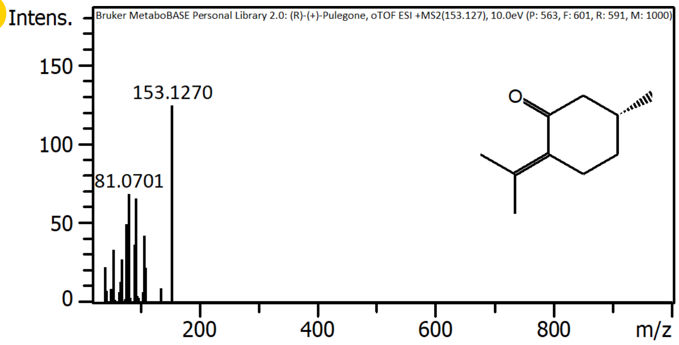

#	m/z	Res.	S/N	I	I %	FWHM
1	43.0190	3855	67.8	552	33.9	0.0112
2	67.0548	6009	76.2	621	38.1	0.0112
3	91.0542	8159	75.8	618	37.9	0.0112
4	93.0702	8340	141.2	1151	70.7	0.0112
5	95.0856	8521	145.8	1188	73.0	0.0112
6	97.0651	8698	93.6	763	46.8	0.0112
7	107.0855	9596	199.8	1628	100.0	0.0112
8	109.1017	9777	92.8	756	46.4	0.0112
9	135.1167	12108	126.4	1030	63.3	0.0112
10	153.1271	13722	141.0	1149	70.6	0.0112

#	m/z	Res.	S/N	I	I %	FWHM
1	41.0396	3678	11.2	22	17.9	0.0112
2	55.0550	4934	16.8	33	26.9	0.0112
3	69.0704	6189	13.9	28	22.3	0.0112
4	77.0389	6904	25.1	50	40.1	0.0112
5	79.0546	7084	15.4	31	24.7	0.0112
6	81.0701	7265	34.5	69	55.3	0.0112
7	91.0544	8159	18.5	37	29.6	0.0112
8	93.0700	8340	33.1	66	53.0	0.0112
9	107.0854	9596	21.3	42	34.1	0.0112
10	153.1270	13722	62.4	124	100.0	0.0112

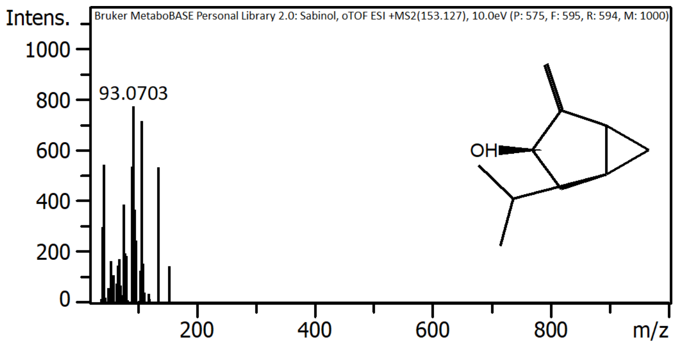

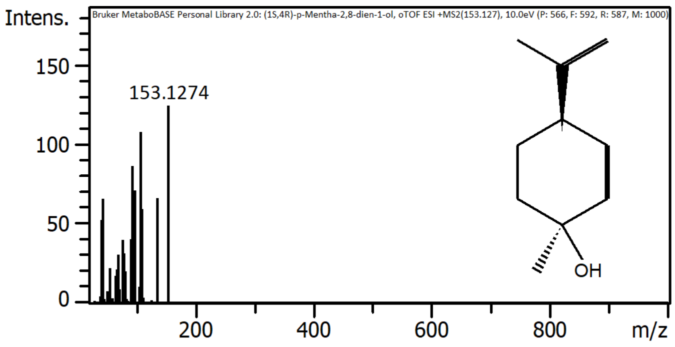

#	m/z	Res.	S/N	I	I %	FWHM
1	41.0394	3678	77.6	299	38.8	0.0112
2	43.0552	3858	140.6	542	70.4	0.0112
3	77.0387	6904	100.6	388	50.4	0.0112
4	79.0545	7084	51.2	198	25.6	0.0112
5	91.0541	8159	139.2	537	69.7	0.0112
6	93.0703	8340	199.8	771	100.0	0.0112
7	95.0852	8521	95.6	369	47.8	0.0112
8	97.0647	8698	64.4	248	32.2	0.0112
9	107.0848	9596	185.0	714	92.6	0.0112
10	135.1154	12108	138.4	534	69.3	0.0112

#	m/z	Res.	S/N	I	I %	FWHM
1	41.0397	3678	84.2	52	42.1	0.0112
2	43.0186	3855	105.8	66	53.0	0.0112
3	93.0699	8340	138.8	86	69.5	0.0112
4	95.0853	8521	103.0	64	51.6	0.0112
5	95.0854	8521	101.2	63	50.7	0.0112
6	97.0648	8698	114.6	71	57.4	0.0112
7	107.0856	9596	173.2	108	86.7	0.0112
8	109.1015	9777	95.8	60	47.9	0.0112
9	135.1169	12108	106.8	66	53.5	0.0112
10	153.1274	13722	199.8	124	100.0	0.0112

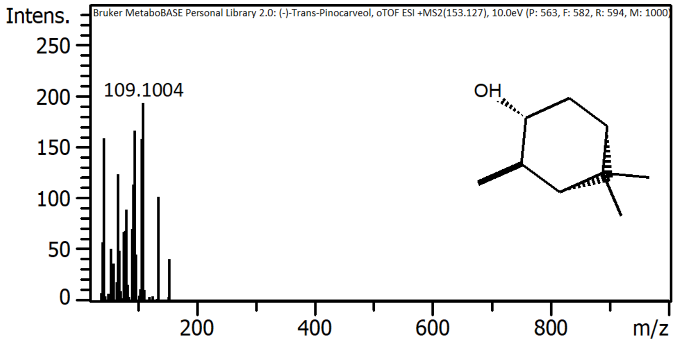

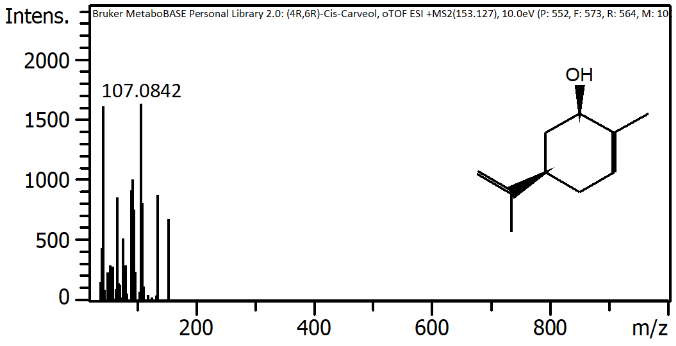

Compound Spectrum List Report

#	m/z	Res.	S/N	I	I %	FWHM
1	43.0184	3855	164.4	159	82.3	0.0112
2	67.0543	6009	128.2	124	64.2	0.0112
3	79.0546	7084	71.6	69	35.8	0.0112
4	81.0692	7265	92.6	89	46.3	0.0112
5	91.0546	8160	73.2	71	36.6	0.0112
6	93.0700	8340	117.6	113	58.9	0.0112
7	95.0860	8521	172.0	166	86.1	0.0112
8	107.0861	9596	164.0	158	82.1	0.0112
9	109.1004	9777	199.8	193	100.0	0.0112
10	135.1162	12108	105.8	102	53.0	0.0112

#	m/z	Res.	S/N	I	I %	FWHM
1	43.0180	3855	105.6	1377	84.6	0.0112
2	43.0181	3855	123.4	1608	98.8	0.0112
3	67.0537	6009	65.9	859	52.8	0.0112
4	91.0537	8159	70.3	916	56.3	0.0112
5	93.0688	8340	77.3	1007	61.9	0.0112
6	95.0838	8521	58.3	759	46.6	0.0112
7	107.0842	9596	124.9	1628	100.0	0.0112
8	109.1001	9777	62.4	813	49.9	0.0112
9	135.1153	12108	67.6	882	54.2	0.0112
10	153.1251	13722	52.3	681	41.8	0.0112

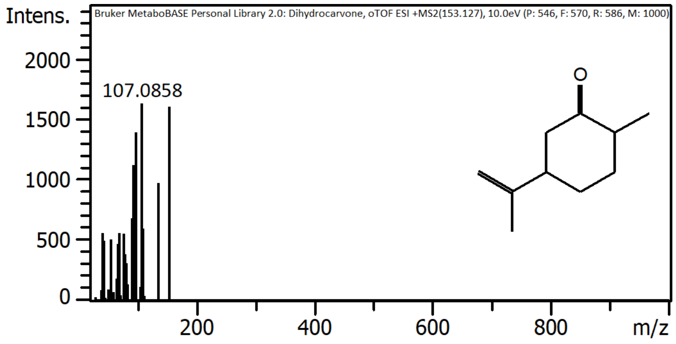

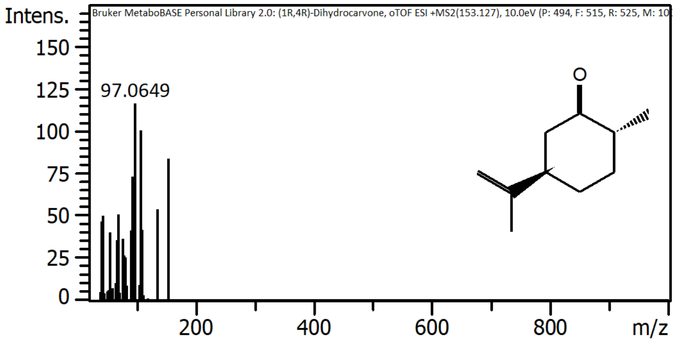

#	m/z	Res.	S/N	I	I %	FWHM
1	41.0400	3678	69.2	564	34.6	0.0112
2	69.0705	6189	68.8	561	34.4	0.0112
3	91.0547	8160	84.0	684	42.0	0.0112
4	93.0704	8340	137.8	1123	69.0	0.0112
5	95.0860	8521	88.6	722	44.3	0.0112
6	97.0651	8698	170.6	1390	85.4	0.0112
7	107.0858	9596	199.8	1628	100.0	0.0112
8	109.1011	9777	73.8	601	36.9	0.0112
9	135.1169	12108	119.8	976	60.0	0.0112
10	153.1274	13722	197.0	1605	98.6	0.0112

#	m/z	Res.	S/N	I	I %	FWHM
1	41.0396	3678	80.6	47	40.3	0.0112
2	43.0187	3855	86.2	50	43.1	0.0112
3	69.0702	6189	87.8	51	43.9	0.0112
4	93.0699	8340	126.2	73	63.2	0.0112
5	95.0856	8521	81.4	47	40.7	0.0112
6	97.0649	8698	199.8	116	100.0	0.0112
7	107.0853	9596	172.6	100	86.4	0.0112
8	109.1008	9777	72.0	42	36.0	0.0112
9	135.1166	12108	92.6	54	46.3	0.0112
10	153.1271	13722	144.0	84	72.1	0.0112

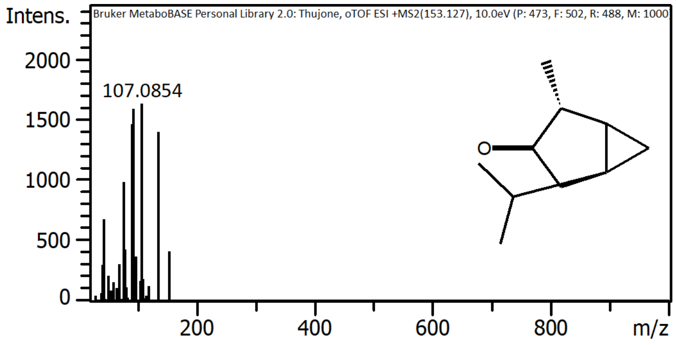

#	m/z	Res.	S/N	I	I %	FWHM
1	43.0183	3855	25.8	378	23.2	0.0112
2	43.0554	3858	46.4	681	41.8	0.0112
3	77.0392	6904	67.3	988	60.7	0.0112
4	79.0547	7084	29.3	430	26.4	0.0112
5	91.0543	8159	99.7	1462	89.8	0.0112
6	93.0699	8340	108.2	1587	97.5	0.0112
7	97.0654	8698	25.4	373	22.9	0.0112
8	107.0854	9596	111.0	1628	100.0	0.0112
9	135.1174	12108	95.1	1395	85.7	0.0112
10	153.1277	13722	28.2	414	25.4	0.0112

Cmpd 842, AutoMSn(247.1330), 9.86 min

Compound Spectrum List Report

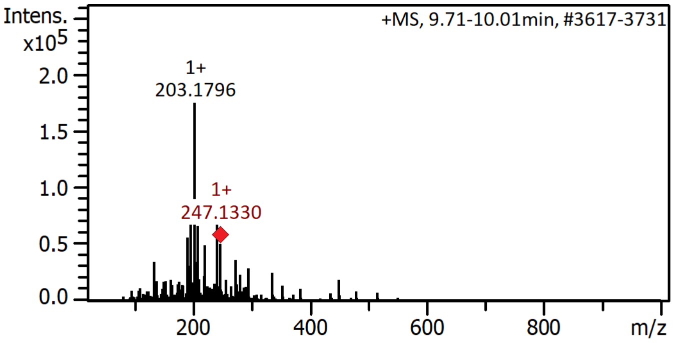

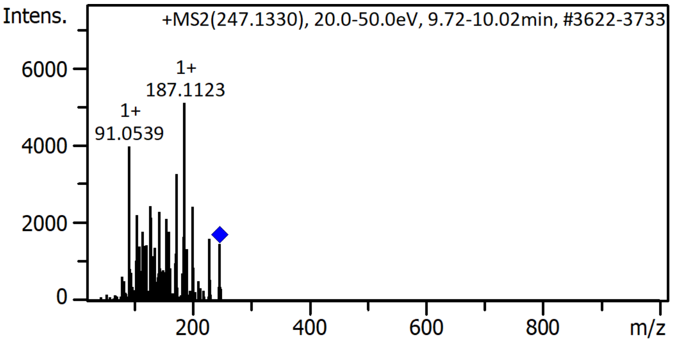

#	m/z	Res.	S/N	I	I %	FWHM
1	191.1432	11245	6230.9	56079	32.1	0.0170
2	197.1174	11156	8713.5	78421	44.9	0.0177
3	203.1796	11384	19402.6	174623	100.0	0.0178
4	207.1377	11405	3878.3	34905	20.0	0.0182
5	209.1539	10921	7373.2	66359	38.0	0.0192
6	221.1903	11113	5512.6	49614	28.4	0.0199
7	241.0975	11979	4672.8	42055	24.1	0.0201
8	241.1434	11814	7853.4	70681	40.5	0.0204
9	247.1330	11597	5605.1	50446	28.9	0.0213
10	273.1625	11967	4068.6	36618	21.0	0.0228

#	m/z	Res.	S/N	I	I %	FWHM
1	91.0539	9648	337.9	3970	78.0	0.0094
2	105.0695	9175	188.3	2212	43.4	0.0115
3	115.0541	11019	152.8	1795	35.3	0.0104
4	128.0612	10693	208.8	2454	48.2	0.0120
5	129.0689	10581	182.7	2147	42.2	0.0122
6	143.0851	12055	195.7	2299	45.2	0.0119
7	155.0724	7711	179.6	2111	41.4	0.0201
8	173.1317	11402	278.9	3277	64.4	0.0152
9	187.1123	11448	433.4	5092	100.0	0.0163
10	201.1276	11182	207.3	2436	47.8	0.0180

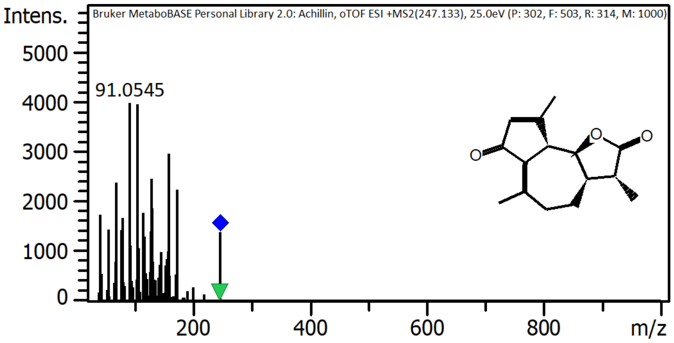

#	m/z	Res.	S/N	I	I %	FWHM
1	41.0396	2367	39.9	1743	43.9	0.0173
2	69.0344	3982	54.5	2382	60.1	0.0173
3	79.0541	4559	38.5	1679	42.3	0.0173
4	91.0545	5252	90.8	3966	100.0	0.0173
5	105.0699	6060	90.3	3942	99.4	0.0173
6	115.0530	6636	40.6	1775	44.7	0.0173
7	129.0694	7444	56.3	2458	62.0	0.0173
8	130.0773	7502	42.8	1870	47.1	0.0173
9	158.0721	9117	67.6	2954	74.5	0.0173
10	173.0968	9983	51.4	2243	56.6	0.0173

Cmpd 848, AutoMSn(191.1432), 9.94 min

Compound Spectrum List Report

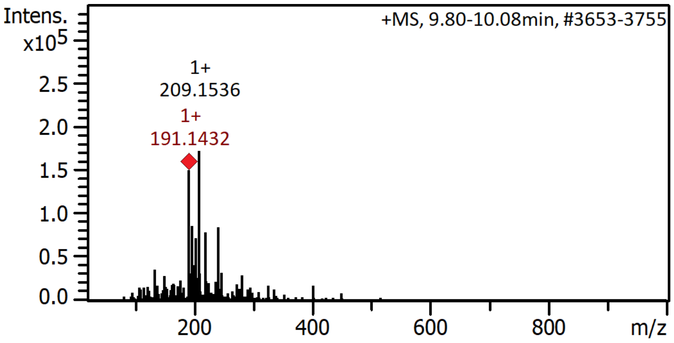

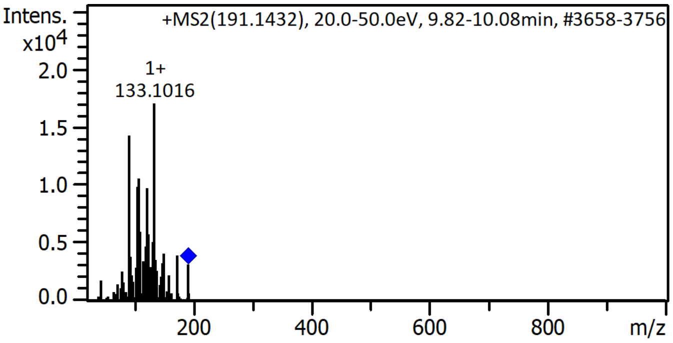

#	m/z	Res.	S/N	I	I %	FWHM
1	133.1008	10032	5917.7	35506	15.6	0.0133
2	191.1432	10980	24979.6	149877	66.0	0.0174
3	197.1172	11335	14365.3	86192	38.0	0.0174
4	201.1636	11420	6766.5	40599	17.9	0.0176
5	203.1799	11548	12080.3	72482	31.9	0.0176
6	209.1536	11295	37832.4	226995	100.0	0.0185
7	219.1743	11729	13121.5	78729	34.7	0.0187
8	241.0975	11813	14149.4	84896	37.4	0.0204
9	241.1429	11993	12077.1	72463	31.9	0.0201
10	247.1330	11482	5317.3	31904	14.1	0.0215

#	m/z	Res.	S/N	I	I %	FWHM
1	91.0545	8873	1990.6	14266	83.8	0.0103
2	105.0697	9335	1373.8	9846	57.8	0.0113
3	107.0856	10051	1475.3	10573	62.1	0.0107
4	109.1008	9840	832.7	5968	35.1	0.0111
5	119.0857	9226	662.2	4746	27.9	0.0129
6	121.1012	10512	1356.1	9719	57.1	0.0115
7	123.0804	9549	802.9	5754	33.8	0.0129
8	131.0857	11087	713.7	5115	30.0	0.0118
9	133.1016	10022	2375.7	17026	100.0	0.0133
10	149.1327	10163	576.6	4132	24.3	0.0147

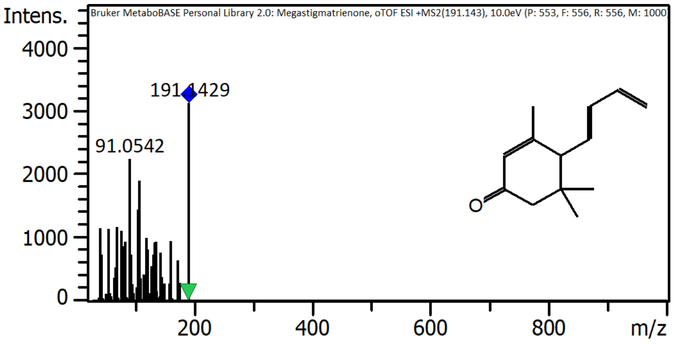

#	m/z	Res.	S/N	I	I %	FWHM
1	41.0394	3051	73.8	1152	36.9	0.0135
2	55.0544	4093	73.4	1146	36.7	0.0135
3	69.0337	5132	75.4	1177	37.7	0.0135
4	77.0389	5727	71.2	1111	35.6	0.0135
5	91.0542	6769	144.0	2248	72.1	0.0135
6	105.0700	7811	93.0	1452	46.5	0.0135
7	107.0850	7961	122.0	1904	61.1	0.0135
8	119.0855	8853	64.4	1005	32.2	0.0135
9	161.0963	11976	60.8	949	30.4	0.0135
10	191.1429	14210	199.8	3119	100.0	0.0135

Cmpd 851, AutoMSn(221.1897), 9.97 min

Compound Spectrum List Report

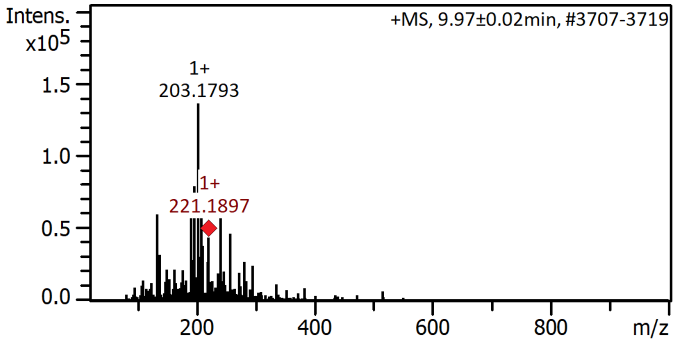

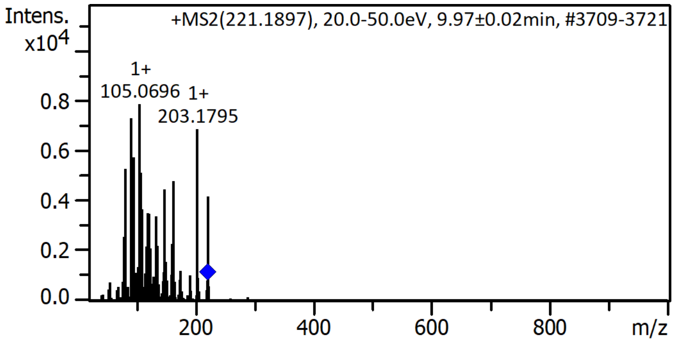

#	m/z	Res.	S/N	I	I %	FWHM
1	133.1012	10224	4976.9	59723	43.9	0.0130
2	137.0959	10096	2656.3	31876	23.4	0.0136
3	191.1432	11442	5857.6	70292	51.7	0.0167
4	197.1173	11439	6594.3	79132	58.2	0.0172
5	203.1793	11633	11334.9	136019	100.0	0.0175
6	209.1537	11233	7110.8	85329	62.7	0.0186
7	211.1689	10817	3192.5	38310	28.2	0.0195
8	221.1897	11491	3659.0	43908	32.3	0.0192
9	241.1439	11733	5904.5	70854	52.1	0.0206
10	257.1288	11341	3887.9	46655	34.3	0.0227

#	m/z	Res.	S/N	I	I %	FWHM
1	81.0695	8873	329.0	5264	67.0	0.0091
2	91.0542	9202	456.2	7299	92.9	0.0099
3	93.0703	9153	249.9	3998	50.9	0.0102
4	95.0853	9529	358.4	5734	73.0	0.0100
5	105.0696	9983	490.8	7853	100.0	0.0105
6	107.0855	9220	320.5	5128	65.3	0.0116
7	147.1163	11096	277.8	4444	56.6	0.0133
8	163.1484	10078	299.7	4795	61.1	0.0162
9	203.1795	11642	429.4	6870	87.5	0.0175
10	222.1489	9855	260.3	4165	53.0	0.0225

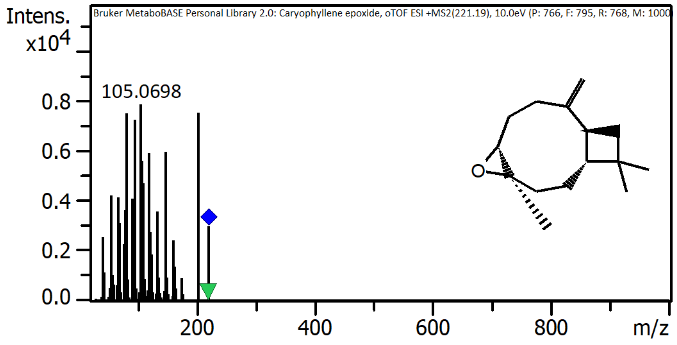

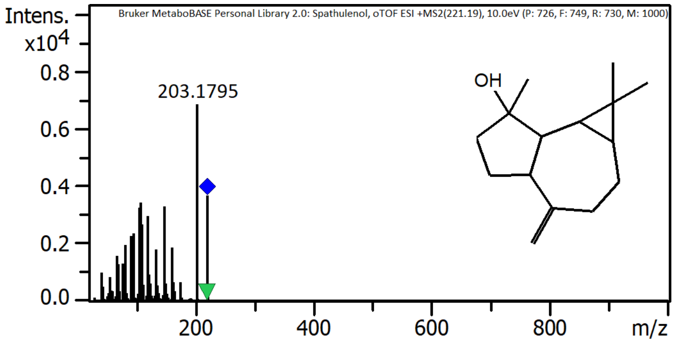

#	m/z	Res.	S/N	I	I %	FWHM
1	55.0555	5271	107.6	4225	53.9	0.0104
2	67.0549	6420	105.6	4146	52.9	0.0104
3	81.0703	7762	190.6	7484	95.4	0.0104
4	95.0856	9103	184.6	7248	92.4	0.0104
5	105.0698	10059	199.8	7845	100.0	0.0104
6	107.0856	10252	142.6	5599	71.4	0.0104
7	109.1009	10445	120.0	4712	60.1	0.0104
8	119.0852	11401	150.8	5921	75.5	0.0104
9	147.1165	14085	151.6	5952	75.9	0.0104
10	203.1790	19452	191.6	7523	95.9	0.0104

#	m/z	Res.	S/N	I	I %	FWHM
1	81.0695	7761	47.8	1972	28.7	0.0104
2	91.0542	8717	55.3	2281	33.2	0.0104
3	95.0845	9103	57.3	2363	34.4	0.0104
4	105.0706	10059	79.2	3263	47.5	0.0104
5	107.0856	10252	83.3	3435	50.1	0.0104
6	109.1016	10445	65.3	2693	39.2	0.0104
7	119.0845	11401	72.2	2975	43.3	0.0104
8	147.1157	14085	80.3	3311	48.2	0.0104
9	203.1795	19452	166.5	6863	100.0	0.0104
10	221.1894	21176	89.5	3689	53.8	0.0104

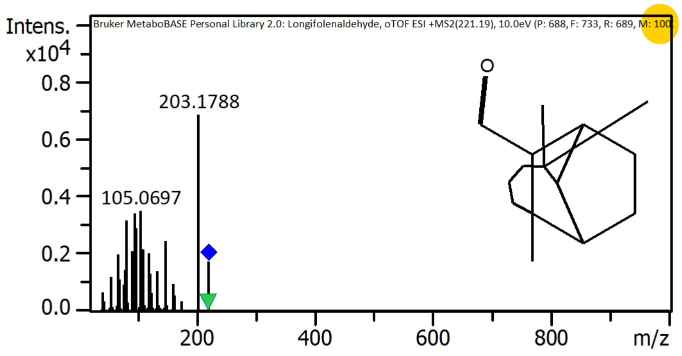

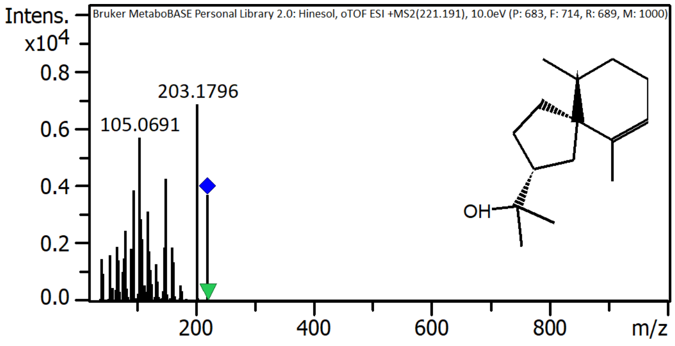

Compound Spectrum List Report

#	m/z	Res.	S/N	I	I %	FWHM
1	81.0701	7762	92.8	3188	46.4	0.0104
2	91.0540	8717	61.4	2109	30.7	0.0104
3	95.0852	9103	99.6	3421	49.8	0.0104
4	97.0647	9293	85.0	2920	42.5	0.0104
5	105.0697	10059	102.4	3517	51.3	0.0104
6	107.0854	10252	62.4	2143	31.2	0.0104
7	109.1011	10445	63.0	2164	31.5	0.0104
8	119.0852	11401	59.2	2034	29.6	0.0104
9	147.1158	14085	71.6	2459	35.8	0.0104
10	203.1788	19452	199.8	6863	100.0	0.0104

#	m/z	Res.	S/N	I	I %	FWHM
1	67.0552	6420	55.8	1917	27.9	0.0104
2	81.0694	7761	71.4	2453	35.7	0.0104
3	95.0848	9103	112.8	3875	56.5	0.0104
4	105.0691	10059	165.6	5688	82.9	0.0104
5	107.0855	10252	83.2	2858	41.6	0.0104
6	109.1013	10445	63.6	2185	31.8	0.0104
7	119.0850	11401	91.2	3133	45.6	0.0104
8	149.1311	14278	124.4	4273	62.3	0.0104
9	203.1796	19452	199.8	6863	100.0	0.0104
10	221.1887	21176	108.0	3710	54.1	0.0104

Cmpd 856, AutoMSn(335.2216), 10.03 min

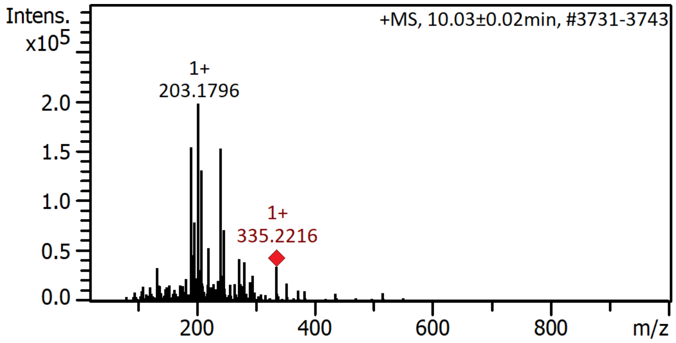

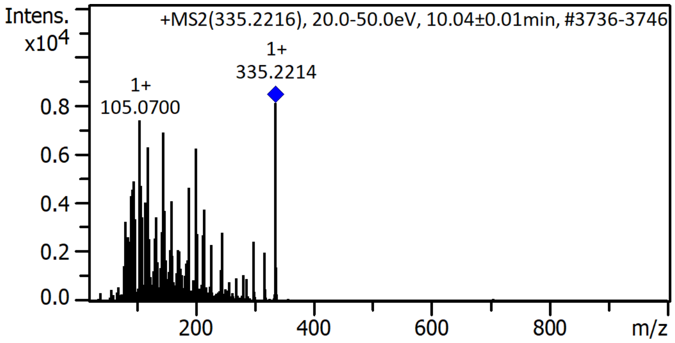

#	m/z	Res.	S/N	I	I %	FWHM
1	191.1433	11067	12854.0	154248	78.0	0.0173
2	195.1016	11465	3855.9	46271	23.4	0.0170
3	197.1174	10898	6617.9	79415	40.2	0.0181
4	203.1796	11559	16479.5	197754	100.0	0.0176
5	209.1538	11405	10936.1	131234	66.4	0.0183
6	221.1902	11436	4473.4	53681	27.1	0.0193
7	241.0975	12072	12740.4	152885	77.3	0.0200
8	241.1425	12116	9163.2	109959	55.6	0.0199
9	247.1330	12120	5962.8	71553	36.2	0.0204
10	273.1622	11721	3570.5	42846	21.7	0.0233

#	m/z	Res.	S/N	I	I %	FWHM
1	91.0526	8275	268.7	4299	53.1	0.0110
2	93.0698	8650	285.4	4567	56.4	0.0108
3	95.0855	9552	307.7	4923	60.8	0.0100
4	105.0700	9571	463.2	7411	91.6	0.0110
5	107.0854	10662	294.8	4716	58.3	0.0100
6	119.0853	10879	394.3	6308	77.9	0.0109
7	145.1001	10884	431.9	6910	85.4	0.0133
8	189.1637	10908	290.3	4645	57.4	0.0173
9	201.1643	11022	390.8	6252	77.2	0.0183
10	335.2214	10904	506.0	8095	100.0	0.0307

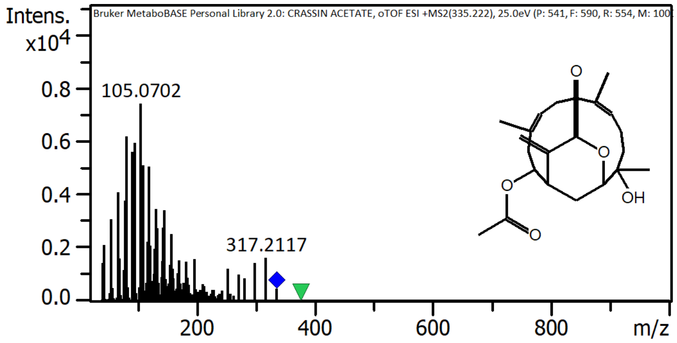

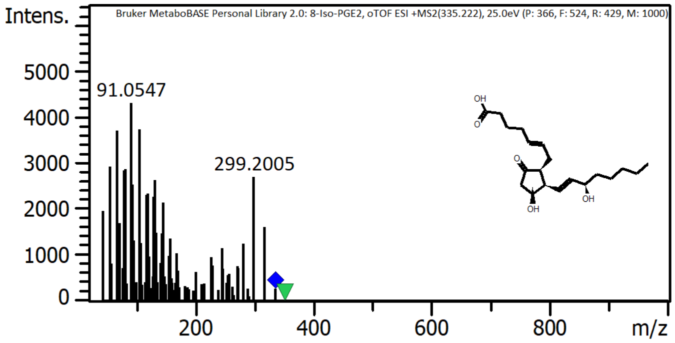

#	m/z	Res.	S/N	I	I %	FWHM
1	67.0555	2192	110.6	4098	55.4	0.0306
2	79.0556	2584	102.2	3787	51.2	0.0306
3	81.0704	2650	167.2	6196	83.7	0.0306
4	91.0547	2976	151.4	5610	75.8	0.0306
5	93.0704	3042	140.2	5195	70.2	0.0306
6	95.0862	3108	160.6	5951	80.4	0.0306
7	105.0702	3434	197.4	7315	98.8	0.0306
8	105.0702	3434	199.8	7404	100.0	0.0306
9	109.1016	3566	137.8	5106	69.0	0.0306
10	119.0853	3893	136.2	5047	68.2	0.0306

#	m/z	Res.	S/N	I	I %	FWHM
1	55.0547	1800	136.4	2932	68.3	0.0306
2	67.0550	2192	172.6	3710	86.4	0.0306
3	79.0544	2584	132.4	2846	66.3	0.0306
4	81.0705	2650	133.8	2876	67.0	0.0306
5	91.0547	2976	199.8	4294	100.0	0.0306
6	93.0704	3042	118.2	2541	59.2	0.0306
7	105.0698	3434	173.4	3727	86.8	0.0306
8	119.0851	3893	108.8	2338	54.5	0.0306
9	131.0852	4285	122.6	2635	61.4	0.0306
10	299.2005	9780	125.6	2700	62.9	0.0306

Compound Spectrum List Report

Cmpd 857, AutoMSn(203.1791), 10.04 min

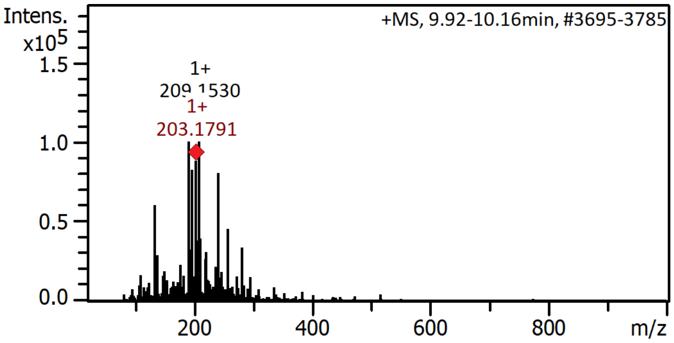

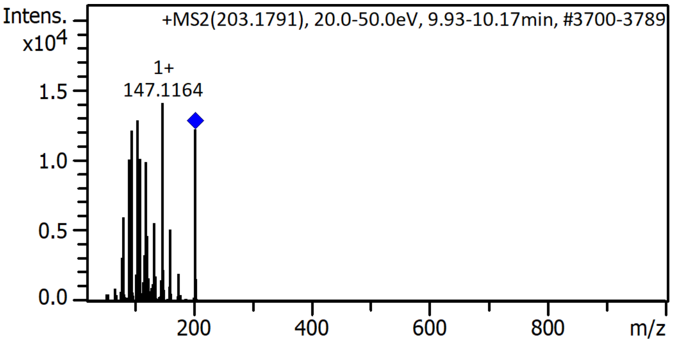

#	m/z	Res.	S/N	I	I %	FWHM
1	133.1008	10223	10085.7	60514	48.8	0.0130
2	191.1427	10933	20007.8	120047	96.7	0.0175
3	197.1170	11269	13815.4	82892	66.8	0.0175
4	203.1791	11344	14739.7	88438	71.3	0.0179
5	207.1376	11462	6436.7	38620	31.1	0.0181
6	209.1530	11341	20684.5	124107	100.0	0.0184
7	211.1682	10957	6612.9	39678	32.0	0.0193
8	241.1432	11784	13452.7	80716	65.0	0.0205
9	257.1280	11871	7594.2	45565	36.7	0.0217
10	281.1357	12073	5652.4	33915	27.3	0.0233

#	m/z	Res.	S/N	I	I %	FWHM
1	81.0699	8073	831.1	5956	42.4	0.0100
2	91.0542	9541	1403.5	10058	71.5	0.0095
3	95.0854	9482	1690.9	12118	86.2	0.0100
4	105.0696	9558	1793.6	12854	91.4	0.0110
5	107.0851	10131	1055.0	7561	53.8	0.0106
6	109.1010	9810	1410.4	10108	71.9	0.0111
7	119.0852	9473	1381.2	9898	70.4	0.0126
8	133.1009	10448	777.3	5571	39.6	0.0127
9	147.1164	10594	1961.7	14059	100.0	0.0139
10	203.1796	11404	1704.9	12218	86.9	0.0178

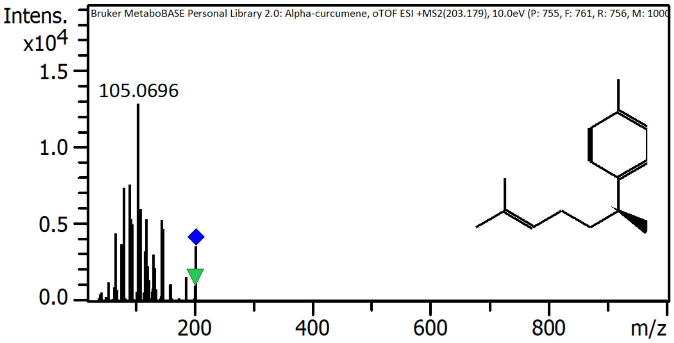

#	m/z	Res.	S/N	I	I %	FWHM
1	81.0697	5524	114.8	7378	57.5	0.0147
2	91.0545	6205	118.0	7584	59.1	0.0147
3	93.0690	6342	82.8	5321	41.4	0.0147
4	95.0851	6479	78.0	5013	39.0	0.0147
5	105.0696	7160	199.8	12841	100.0	0.0147
6	107.0844	7297	93.4	6003	46.7	0.0147
7	109.1014	7434	93.4	6003	46.7	0.0147
8	119.0845	8115	82.8	5321	41.4	0.0147
9	145.0992	9887	82.2	5283	41.1	0.0147
10	147.1150	10025	73.2	4705	36.6	0.0147

Cmpd 864, AutoMSn(265.1430), 10.12 min

Compound Spectrum List Report

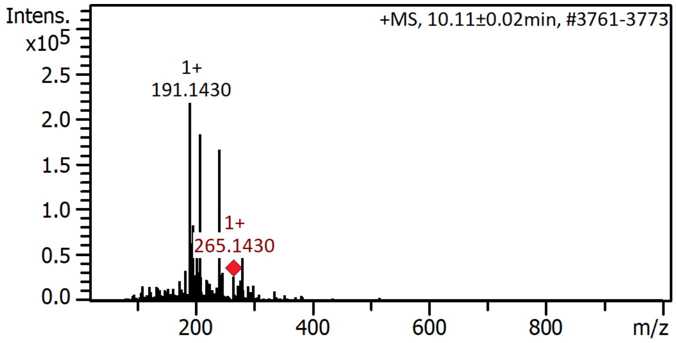

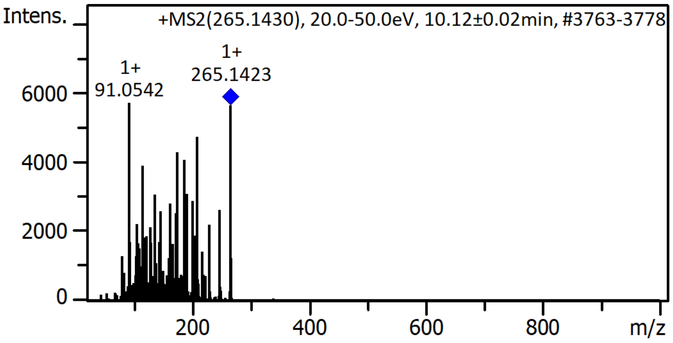

#	m/z	Res.	S/N	I	I %	FWHM
1	183.1016	11018	2731.8	32782	15.1	0.0166
2	191.1430	11337	18126.1	217513	100.0	0.0169
3	195.1015	11031	5321.4	63857	29.4	0.0177
4	197.1173	10662	6962.6	83552	38.4	0.0185
5	203.1795	11588	5817.9	69815	32.1	0.0175
6	207.1380	11271	2629.2	31551	14.5	0.0184
7	209.1537	11416	15251.4	183017	84.1	0.0183
8	241.0973	11676	9095.7	109149	50.2	0.0206
9	241.1427	11844	13880.0	166560	76.6	0.0204
10	281.1367	11840	5943.7	71324	32.8	0.0237

#	m/z	Res.	S/N	I	I %	FWHM
1	91.0542	9603	356.4	5703	100.0	0.0095
2	115.0550	10249	244.1	3906	68.5	0.0112
3	135.0801	11303	192.1	3073	53.9	0.0120
4	163.0743	12223	176.0	2817	49.4	0.0133
5	175.0391	13082	268.3	4293	75.3	0.0134
6	187.1107	11587	254.3	4068	71.3	0.0161
7	191.0703	13660	192.7	3083	54.1	0.0140
8	201.1290	11905	180.2	2884	50.6	0.0169
9	209.0811	9803	295.1	4722	82.8	0.0213
10	265.1423	11727	352.0	5632	98.7	0.0226

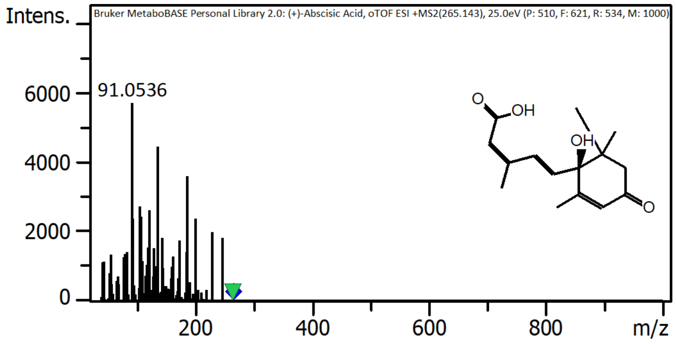

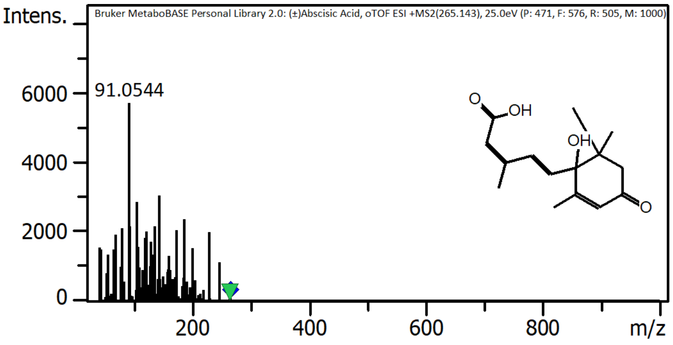

#	m/z	Res.	S/N	I	I %	FWHM
1	91.0536	11021	199.8	5697	100.0	0.0083
2	93.0697	11265	83.6	2384	41.8	0.0083
3	105.0699	12718	96.0	2737	48.0	0.0083
4	107.0853	12962	85.6	2441	42.8	0.0083
5	121.0639	14653	92.2	2629	46.1	0.0083
6	135.0798	16350	156.0	4448	78.1	0.0083
7	187.1108	22648	126.0	3593	63.1	0.0083
8	201.1268	24344	83.8	2390	41.9	0.0083
9	229.1222	27733	70.2	2002	35.1	0.0083
10	247.1327	29913	64.0	1825	32.0	0.0083

#	m/z	Res.	S/N	I	I %	FWHM
1	79.0548	9569	73.8	2104	36.9	0.0083
2	91.0544	11021	199.8	5697	100.0	0.0083
3	93.0706	11265	75.8	2161	37.9	0.0083
4	105.0702	12718	100.4	2863	50.3	0.0083
5	121.0635	14653	70.8	2019	35.4	0.0083
6	135.0806	16350	76.2	2173	38.1	0.0083
7	135.0822	16350	75.0	2139	37.5	0.0083
8	143.0826	17319	106.8	3045	53.5	0.0083
9	173.1307	20956	71.8	2047	35.9	0.0083
10	187.1118	22648	83.2	2372	41.6	0.0083

Cmpd 883, AutoMSn(203.1793), 10.34 min

Compound Spectrum List Report

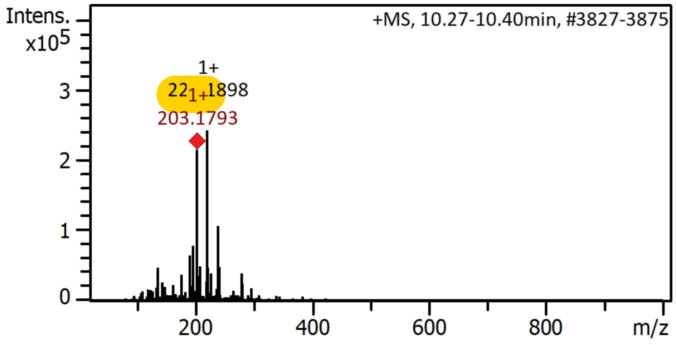

#	m/z	Res.	S/N	I	I %	FWHM
1	135.1166	10694	10463.0	47084	16.8	0.0126
2	191.1430	11342	14372.4	64676	23.0	0.0169
3	197.1172	11366	17550.1	78976	28.1	0.0173
4	203.1793	11107	47845.7	215306	76.6	0.0183
5	209.1534	11071	10886.5	48989	17.4	0.0189
6	221.1898	11026	62455.4	281049	100.0	0.0201
7	222.1930	11652	10498.5	47243	16.8	0.0191
8	239.2007	11858	23759.3	106917	38.0	0.0202
9	241.1438	11735	10667.5	48004	17.1	0.0205
10	279.1931	12330	8654.2	38944	13.9	0.0226

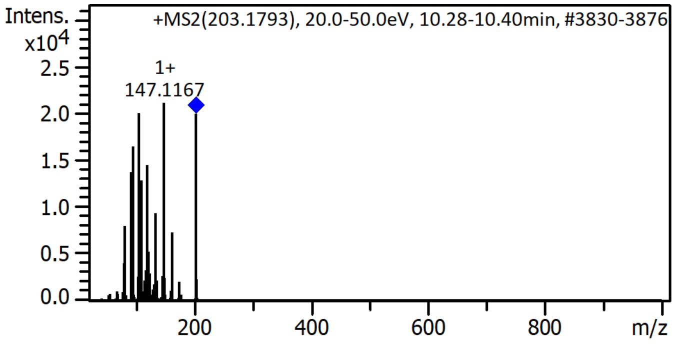

#	m/z	Res.	S/N	I	I %	FWHM
1	81.0700	8160	1648.7	8038	38.1	0.0099
2	91.0543	9187	2824.1	13768	65.3	0.0099
3	95.0857	9120	3371.1	16434	78.0	0.0104
4	105.0698	9495	4110.7	20040	95.1	0.0111
5	107.0854	9859	2206.9	10759	51.0	0.0109
6	109.1009	9831	2639.4	12867	61.1	0.0111
7	119.0852	10094	2973.1	14494	68.8	0.0118
8	133.1011	10941	1921.7	9369	44.5	0.0122
9	147.1167	10777	4323.3	21076	100.0	0.0137
10	203.1796	11714	4095.3	19965	94.7	0.0173

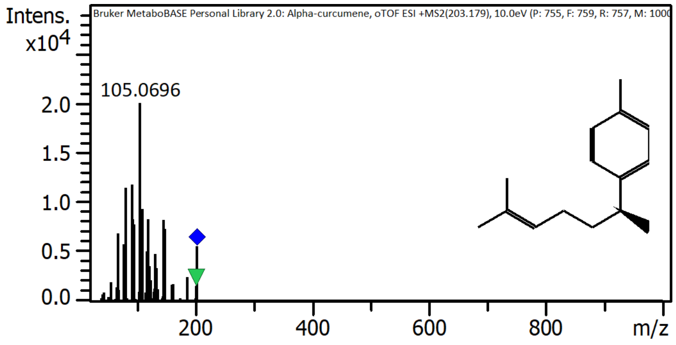

#	m/z	Res.	S/N	I	I %	FWHM
1	81.0697	5519	114.8	11503	57.5	0.0147
2	91.0545	6199	118.0	11823	59.1	0.0147
3	93.0690	6336	82.8	8296	41.4	0.0147
4	95.0851	6474	78.0	7816	39.0	0.0147
5	105.0696	7153	199.8	20020	100.0	0.0147
6	107.0844	7290	93.4	9359	46.7	0.0147
7	109.1014	7428	93.4	9359	46.7	0.0147
8	119.0845	8107	82.8	8296	41.4	0.0147
9	145.0992	9879	82.2	8236	41.1	0.0147
10	147.1150	10016	73.2	7335	36.6	0.0147

Cmpd 888, AutoMSn(191.1436), 10.40 min

Compound Spectrum List Report

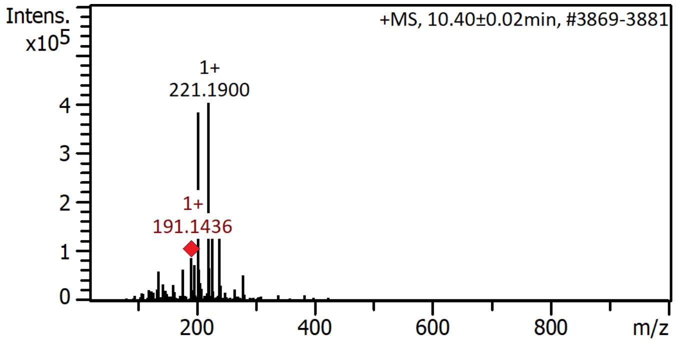

#	m/z	Res.	S/N	I	I %	FWHM
1	135.1168	10852	4978.2	59739	14.8	0.0125
2	177.0549	11455	5231.8	62782	15.6	0.0155
3	191.1436	11456	7353.7	88244	21.9	0.0167
4	197.1176	11369	6028.9	72347	18.0	0.0173
5	203.1795	10943	31959.6	383515	95.3	0.0186
6	204.1830	11515	5217.6	62612	15.6	0.0177
7	221.1900	10939	33548.4	402581	100.0	0.0202
8	222.1934	11660	5443.5	65322	16.2	0.0191
9	227.1284	11518	11676.8	140121	34.8	0.0197
10	239.2008	11905	11851.4	142217	35.3	0.0201

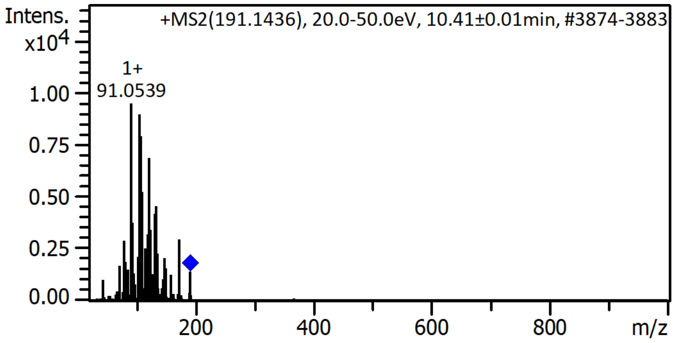

#	m/z	Res.	S/N	I	I %	FWHM
1	91.0539	9018	618.1	9478	100.0	0.0101
2	93.0688	10298	246.1	3774	39.8	0.0090
3	105.0685	10246	584.1	8956	94.5	0.0103
4	107.0853	10262	516.6	7921	83.6	0.0104
5	109.1008	11278	341.8	5241	55.3	0.0097
6	119.0854	9612	208.9	3203	33.8	0.0124
7	121.1003	10831	447.6	6863	72.4	0.0112
8	123.0798	10594	222.7	3414	36.0	0.0116
9	131.0841	11411	274.5	4210	44.4	0.0115
10	133.1004	10426	298.2	4572	48.2	0.0128

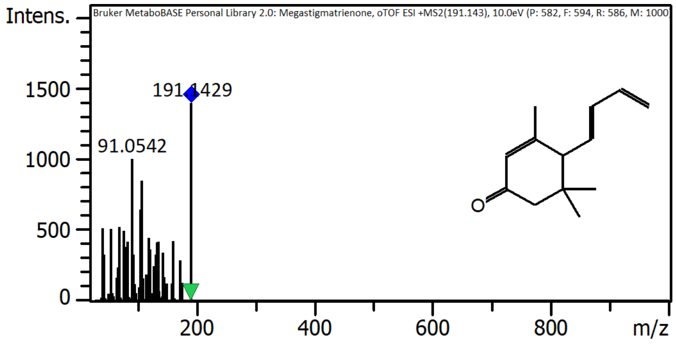

#	m/z	Res.	S/N	I	I %	FWHM
1	41.0394	4648	73.8	514	36.9	0.0088
2	55.0544	6235	73.4	511	36.7	0.0088
3	69.0337	7818	75.4	525	37.7	0.0088
4	77.0389	8725	71.2	496	35.6	0.0088
5	91.0542	10312	144.0	1003	72.1	0.0088
6	105.0700	11900	93.0	648	46.5	0.0088
7	107.0850	12128	122.0	850	61.1	0.0088
8	119.0855	13487	64.4	448	32.2	0.0088
9	161.0963	18245	60.8	423	30.4	0.0088
10	191.1429	21648	199.8	1391	100.0	0.0088

Cmpd 909, AutoMSn(203.1791), 10.65 min

Compound Spectrum List Report

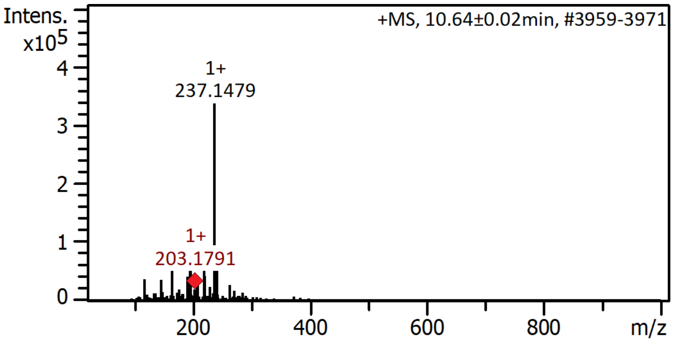

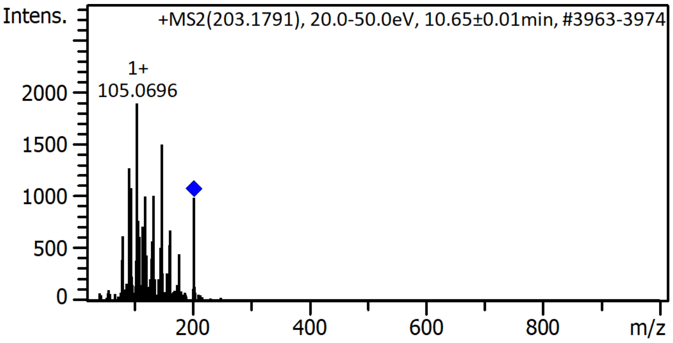

#	m/z	Res.	S/N	I	I %	FWHM
1	117.0543	10047	3061.8	36742	10.9	0.0117
2	165.1267	10879	4288.1	51457	15.2	0.0152
3	191.1426	11375	3386.0	40632	12.0	0.0168
4	195.1010	11135	4364.2	52370	15.5	0.0175
5	197.1167	10953	6394.3	76732	22.7	0.0180
6	219.1225	11626	4559.4	54713	16.2	0.0188
7	221.1891	11667	3482.6	41791	12.4	0.0190
8	237.1479	11372	28139.5	337674	100.0	0.0209
9	238.1511	11619	4274.3	51291	15.2	0.0205
10	241.1037	11581	7519.3	90232	26.7	0.0208

#	m/z	Res.	S/N	I	I %	FWHM
1	91.0547	10331	77.9	1272	67.2	0.0088
2	95.0843	10692	66.3	1084	57.3	0.0089
3	105.0696	9834	115.8	1892	100.0	0.0107
4	107.0844	12209	47.1	770	40.7	0.0088
5	115.0525	12482	43.8	715	37.8	0.0092
6	117.0683	11090	43.7	714	37.8	0.0106
7	119.0846	11027	61.1	999	52.8	0.0108
8	133.1003	11090	61.9	1010	53.4	0.0120
9	147.1180	10259	91.8	1500	79.3	0.0143
10	203.1806	11460	60.6	990	52.3	0.0177

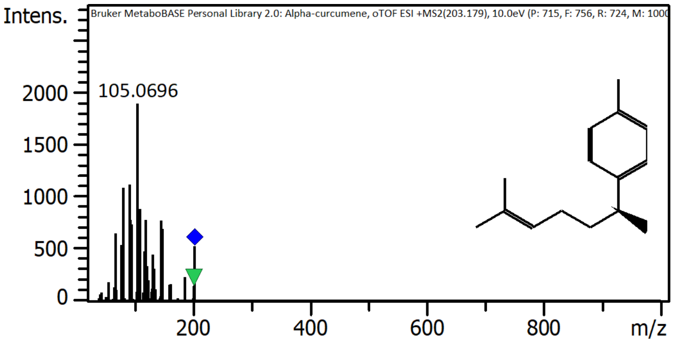

#	m/z	Res.	S/N	I	I %	FWHM
1	81.0697	8921	114.8	1086	57.5	0.0091
2	91.0545	10020	118.0	1116	59.1	0.0091
3	93.0690	10241	82.8	783	41.4	0.0091
4	95.0851	10463	78.0	738	39.0	0.0091
5	105.0696	11562	199.8	1890	100.0	0.0091
6	107.0844	11783	93.4	883	46.7	0.0091
7	109.1014	12005	93.4	883	46.7	0.0091
8	119.0845	13104	82.8	783	41.4	0.0091
9	145.0992	15967	82.2	777	41.1	0.0091
10	147.1150	16188	73.2	692	36.6	0.0091

Cmpd 912, AutoMSn(229.1422), 10.66 min

Compound Spectrum List Report

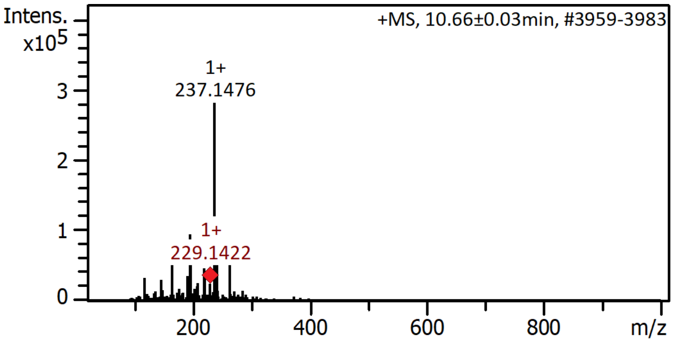

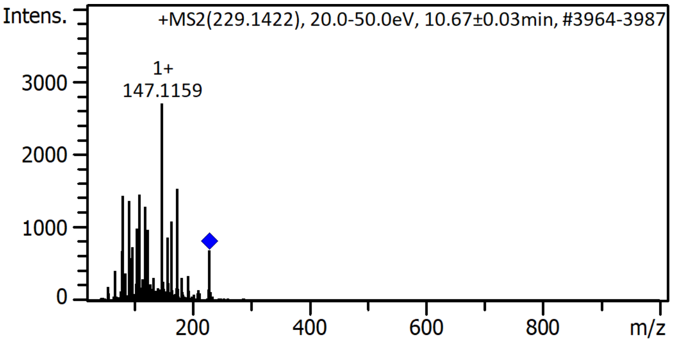

#	m/z	Res.	S/N	I	I %	FWHM
1	165.1266	10949	7650.0	55080	19.6	0.0151
2	191.1425	11314	4885.7	35177	12.5	0.0169
3	195.1006	11178	13198.1	95026	33.8	0.0175
4	197.1164	11192	11024.7	79378	28.2	0.0176
5	219.1222	11383	6343.2	45671	16.2	0.0193
6	237.1476	11420	39069.1	281298	100.0	0.0208
7	238.1509	11462	5955.9	42883	15.2	0.0208
8	241.1040	11043	10916.8	78601	27.9	0.0218
9	241.1406	11426	8010.4	57675	20.5	0.0211
10	263.1243	11957	8117.3	58445	20.8	0.0220

#	m/z	Res.	S/N	I	I %	FWHM
1	81.0701	9084	150.1	1441	53.3	0.0089
2	91.0542	8682	142.9	1372	50.8	0.0105
3	105.0699	9760	103.7	995	36.8	0.0108
4	109.1009	9041	151.3	1452	53.8	0.0121
5	119.0851	11587	134.3	1289	47.7	0.0103
6	123.1167	11657	101.7	976	36.1	0.0106
7	147.1159	10942	281.4	2702	100.0	0.0134
8	157.0994	11359	90.7	870	32.2	0.0138
9	165.1284	8847	113.0	1085	40.2	0.0187
10	175.1106	10708	159.8	1534	56.8	0.0164

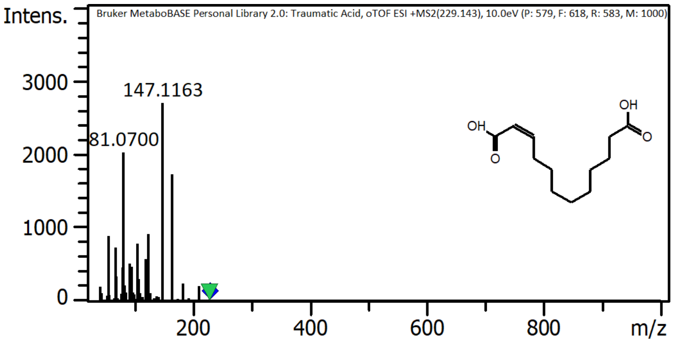

#	m/z	Res.	S/N	I	I %	FWHM
1	55.0187	4114	66.4	897	33.2	0.0134
2	55.0544	4117	46.4	627	23.2	0.0134
3	67.0543	5014	54.4	735	27.2	0.0134
4	81.0700	6062	150.0	2026	75.1	0.0134
5	91.0536	6809	38.6	521	19.3	0.0134
6	105.0696	7857	58.6	792	29.3	0.0134
7	119.0846	8905	43.2	584	21.6	0.0134
8	123.1163	9206	68.0	919	34.0	0.0134
9	147.1163	11001	199.8	2699	100.0	0.0134
10	165.1264	12347	128.0	1729	64.1	0.0134

Cmpd 916, AutoMSn(273.1836), 10.75 min

Compound Spectrum List Report

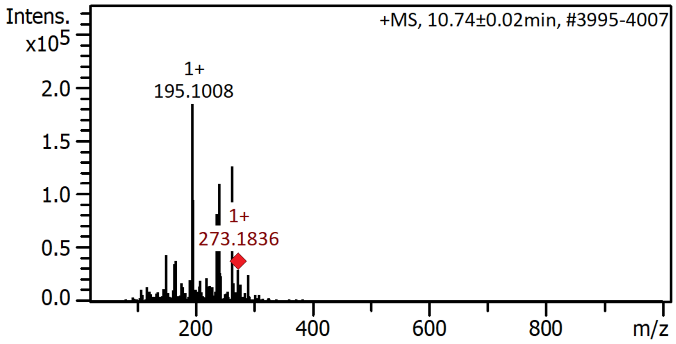

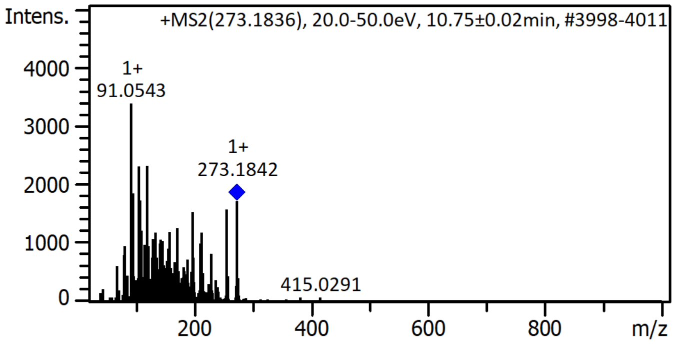

#	m/z	Res.	S/N	I	I %	FWHM
1	149.1317	11146	3644.0	43728	23.7	0.0134
2	165.1264	10696	2939.4	35273	19.1	0.0154
3	167.1060	11383	3210.9	38530	20.9	0.0147
4	195.1008	11444	15363.0	184356	100.0	0.0170
5	197.1167	11032	7962.3	95547	51.8	0.0179
6	237.1480	11747	6831.7	81980	44.5	0.0202
7	241.1424	11640	9164.3	109971	59.7	0.0207
8	242.1441	12212	2212.9	26555	14.4	0.0198
9	263.1245	12010	10510.1	126121	68.4	0.0219
10	273.1836	11798	2461.4	29537	16.0	0.0232

#	m/z	Res.	S/N	I	I %	FWHM
1	91.0543	9209	211.4	3383	100.0	0.0099
2	93.0702	11008	86.7	1387	41.0	0.0085
3	95.0860	8895	115.5	1848	54.6	0.0107
4	105.0689	8939	144.4	2310	68.3	0.0118
5	107.0842	9587	108.2	1731	51.2	0.0112
6	119.0856	10467	144.9	2318	68.5	0.0114
7	171.1153	11745	78.7	1259	37.2	0.0146
8	197.1338	12063	96.1	1538	45.5	0.0163
9	255.1780	10935	98.5	1576	46.6	0.0233
10	273.1842	9745	107.3	1716	50.7	0.0280

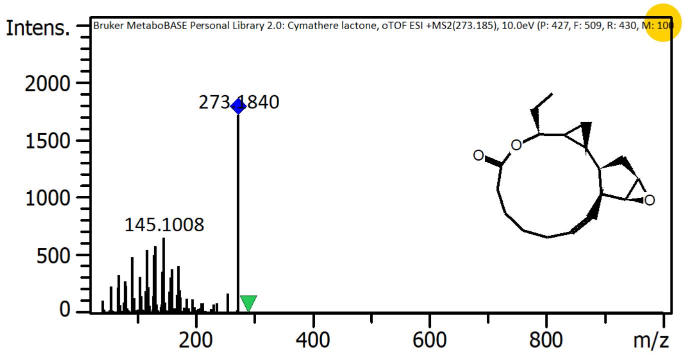

#	m/z	Res.	S/N	I	I %	FWHM
1	91.0539	11125	57.4	493	28.7	0.0082
2	117.0688	14303	48.4	415	24.2	0.0082
3	117.0694	14303	64.4	553	32.2	0.0082
4	129.0689	15769	59.0	506	29.5	0.0082
5	131.0847	16015	68.4	587	34.2	0.0082
6	143.0849	17481	42.0	360	21.0	0.0082
7	145.1008	17728	76.2	654	38.1	0.0082
8	159.1163	19440	45.0	386	22.5	0.0082
9	171.1161	20906	48.0	412	24.0	0.0082
10	273.1840	33376	199.8	1715	100.0	0.0082

Cmpd 925, AutoMSn(191.1423), 10.83 min

Compound Spectrum List Report

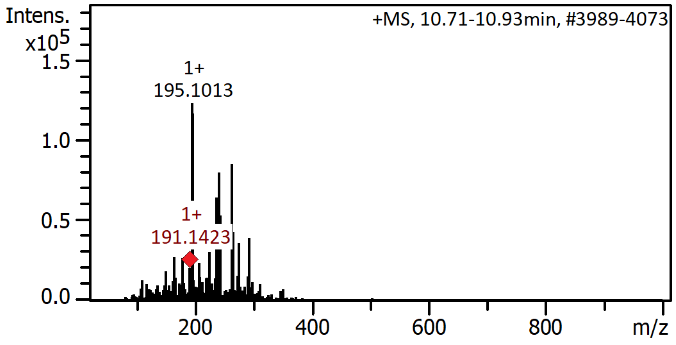

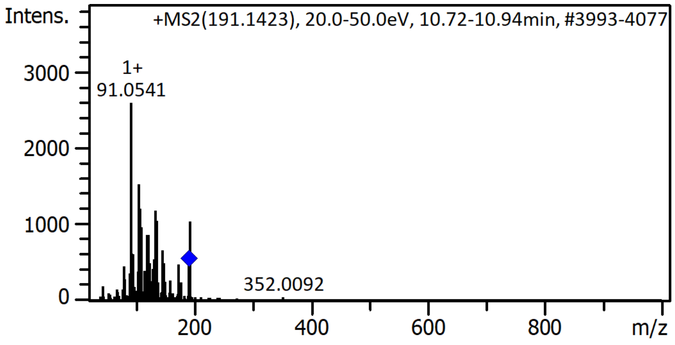

#	m/z	Res.	S/N	I	I %	FWHM
1	195.1013	11318	13647.9	122831	100.0	0.0172
2	197.1166	11240	12924.2	116318	94.7	0.0175
3	225.1478	12068	3371.7	30345	24.7	0.0187
4	237.1478	11533	7153.8	64384	52.4	0.0206
5	241.1425	11736	8854.3	79688	64.9	0.0205
6	243.1575	12362	5887.6	52989	43.1	0.0197
7	263.1245	12243	9456.2	85106	69.3	0.0215
8	265.1402	12065	4765.3	42888	34.9	0.0220
9	275.1992	12404	4009.8	36089	29.4	0.0222
10	293.2102	12264	4347.1	39124	31.9	0.0239

#	m/z	Res.	S/N	I	I %	FWHM
1	91.0541	9287	203.3	2592	100.0	0.0098
2	105.0707	10593	120.2	1532	59.1	0.0099
3	107.0853	11434	94.8	1209	46.6	0.0094
4	109.1016	12236	75.9	968	37.4	0.0089
5	119.0851	7646	67.8	865	33.3	0.0156
6	121.1008	10453	68.0	868	33.5	0.0116
7	133.1003	10956	93.1	1187	45.8	0.0121
8	135.0804	8297	82.5	1051	40.6	0.0163
9	135.1154	10330	53.4	681	26.3	0.0131
10	193.0510	14079	81.8	1043	40.2	0.0137

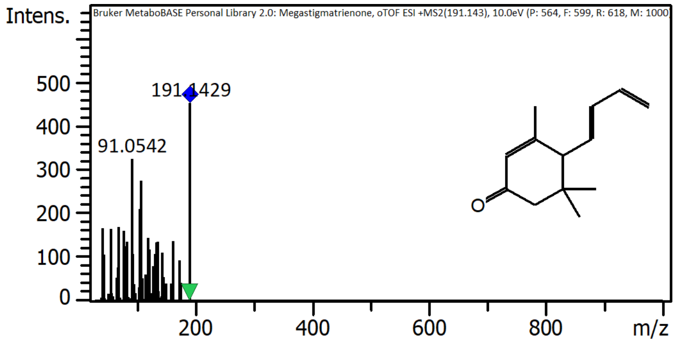

#	m/z	Res.	S/N	I	I %	FWHM
1	41.0394	4924	73.8	167	36.9	0.0083
2	55.0544	6606	73.4	166	36.7	0.0083
3	69.0337	8283	75.4	171	37.7	0.0083
4	77.0389	9244	71.2	161	35.6	0.0083
5	91.0542	10926	144.0	326	72.1	0.0083
6	105.0700	12607	93.0	210	46.5	0.0083
7	107.0850	12849	122.0	276	61.1	0.0083
8	119.0855	14289	64.4	146	32.2	0.0083
9	161.0963	19330	60.8	138	30.4	0.0083
10	191.1429	22935	199.8	452	100.0	0.0083

Cmpd 926, AutoMSn(293.2112), 10.84 min

Compound Spectrum List Report

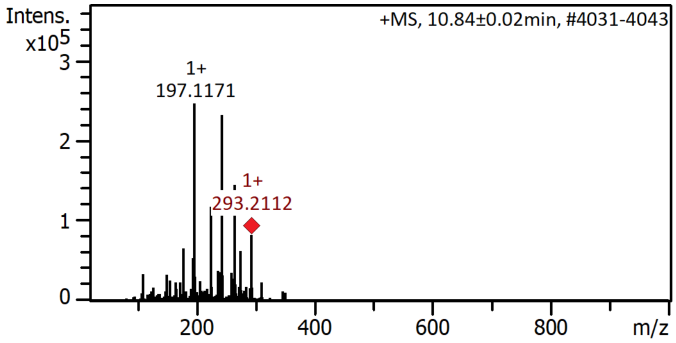

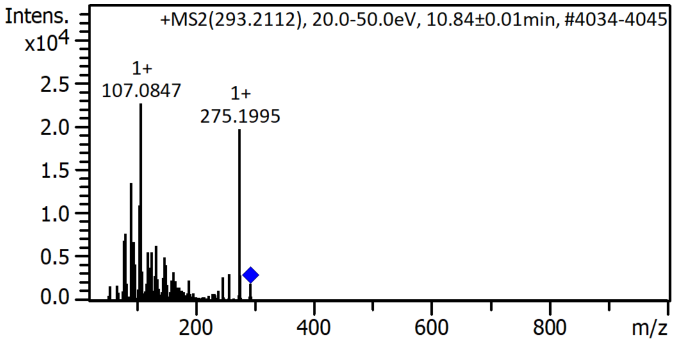

#	m/z	Res.	S/N	I	I %	FWHM
1	179.1064	10935	5478.5	65742	26.7	0.0164
2	195.1015	11341	4512.9	54155	22.0	0.0172
3	197.1171	11309	20557.1	246685	100.0	0.0174
4	225.1482	11820	9794.5	117534	47.6	0.0190
5	237.1483	11684	3112.2	37347	15.1	0.0203
6	241.1430	11727	2950.4	35405	14.4	0.0206
7	243.1589	11832	19304.0	231648	93.9	0.0206
8	265.1407	11957	12095.3	145143	58.8	0.0222
9	275.2003	11360	5217.0	62604	25.4	0.0242
10	293.2112	12408	6902.6	82831	33.6	0.0236

#	m/z	Res.	S/N	I	I %	FWHM
1	79.0534	8477	443.9	6954	30.7	0.0093
2	81.0696	8487	495.9	7769	34.3	0.0096
3	91.0539	9394	865.3	13556	59.8	0.0097
4	93.0698	8934	385.0	6031	26.6	0.0104
5	95.0850	9834	434.0	6800	30.0	0.0097
6	105.0690	9883	698.4	10942	48.3	0.0106
7	107.0847	9597	1445.8	22651	100.0	0.0112
8	125.0950	9576	360.5	5648	24.9	0.0131
9	133.0996	11827	403.0	6313	27.9	0.0113
10	275.1995	12560	1258.7	19719	87.1	0.0219

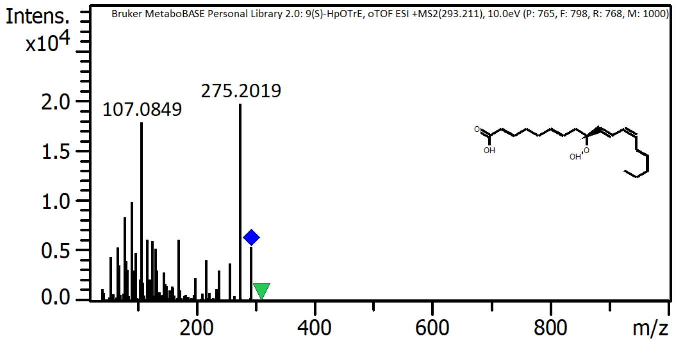

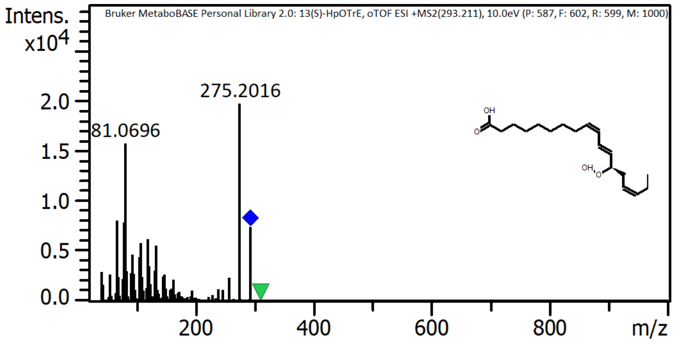

#	m/z	Res.	S/N	I	I %	FWHM
1	67.0547	6452	54.4	5364	27.2	0.0104
2	79.0548	7606	85.0	8381	42.5	0.0104
3	91.0538	8761	100.6	9919	50.4	0.0104
4	107.0849	10303	181.2	17866	90.7	0.0104
5	117.0699	11264	62.2	6133	31.1	0.0104
6	125.0948	12036	61.4	6054	30.7	0.0104
7	131.0845	12612	53.2	5245	26.6	0.0104
8	171.1027	16463	62.8	6192	31.4	0.0104
9	275.2019	26478	199.8	19700	100.0	0.0104
10	293.2087	28211	55.2	5443	27.6	0.0104

#	m/z	Res.	S/N	I	I %	FWHM
1	67.0547	6452	74.2	7316	37.1	0.0104
2	67.0547	6452	81.8	8065	40.9	0.0104
3	79.0542	7606	80.0	7888	40.0	0.0104
4	81.0696	7800	159.4	15716	79.8	0.0104
5	93.0695	8955	47.6	4693	23.8	0.0104
6	107.0848	10303	59.4	5857	29.7	0.0104
7	119.0851	11458	63.4	6251	31.7	0.0104
8	133.0999	12806	56.8	5600	28.4	0.0104
9	275.2016	26478	199.8	19700	100.0	0.0104
10	293.2108	28211	75.6	7454	37.8	0.0104

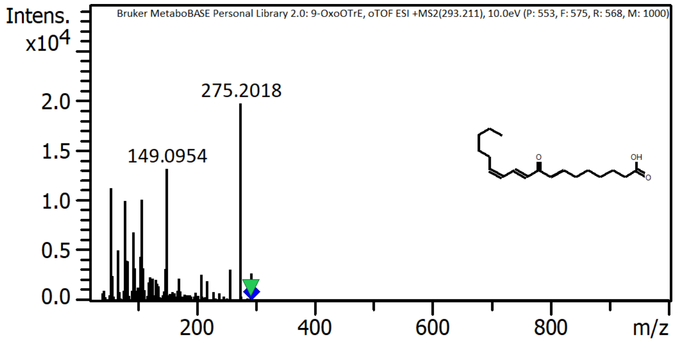

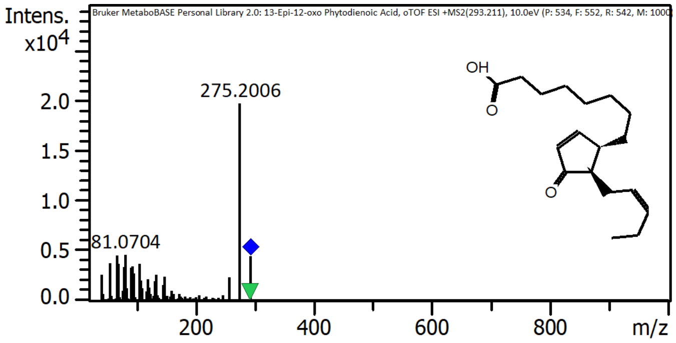

Compound Spectrum List Report

#	m/z	Res.	S/N	I	I %	FWHM
1	55.0546	5297	64.6	6369	32.3	0.0104
2	55.0549	5297	114.4	11279	57.3	0.0104
3	67.0538	6452	51.4	5068	25.7	0.0104
4	79.0545	7606	101.6	10017	50.9	0.0104
5	81.0694	7800	41.2	4062	20.6	0.0104
6	93.0698	8955	69.8	6882	34.9	0.0104
7	105.0688	10109	44.8	4417	22.4	0.0104
8	107.0854	10303	102.8	10136	51.5	0.0104
9	149.0954	14345	134.0	13212	67.1	0.0104
10	275.2018	26478	199.8	19700	100.0	0.0104

#	m/z	Res.	S/N	I	I %	FWHM
1	55.0548	5297	38.8	3826	19.4	0.0104
2	67.0547	6452	46.0	4535	23.0	0.0104
3	69.0703	6646	37.2	3668	18.6	0.0104
4	79.0545	7606	33.8	3333	16.9	0.0104
5	81.0704	7800	47.2	4654	23.6	0.0104
6	91.0541	8761	33.4	3293	16.7	0.0104
7	93.0702	8955	34.4	3392	17.2	0.0104
8	105.0699	10109	37.4	3688	18.7	0.0104
9	275.2006	26478	199.8	19700	100.0	0.0104
10	293.2097	28211	45.8	4516	22.9	0.0104

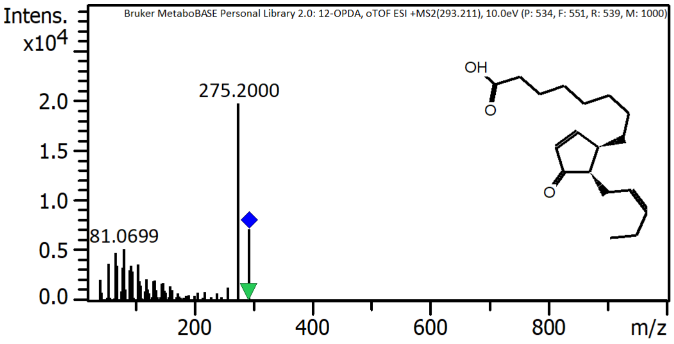

#	m/z	Res.	S/N	I	I %	FWHM
1	55.0545	5297	37.4	3688	18.7	0.0104
2	67.0538	6452	49.0	4831	24.5	0.0104
3	69.0702	6646	33.0	3254	16.5	0.0104
4	69.0703	6646	35.4	3490	17.7	0.0104
5	79.0545	7606	33.2	3273	16.6	0.0104
6	81.0699	7800	52.8	5206	26.4	0.0104
7	93.0697	8955	35.0	3451	17.5	0.0104
8	105.0693	10109	36.4	3589	18.2	0.0104
9	275.2000	26478	199.8	19700	100.0	0.0104
10	293.2110	28211	72.8	7178	36.4	0.0104

Cmpd 944, AutoMSn(181.1218), 11.07 min

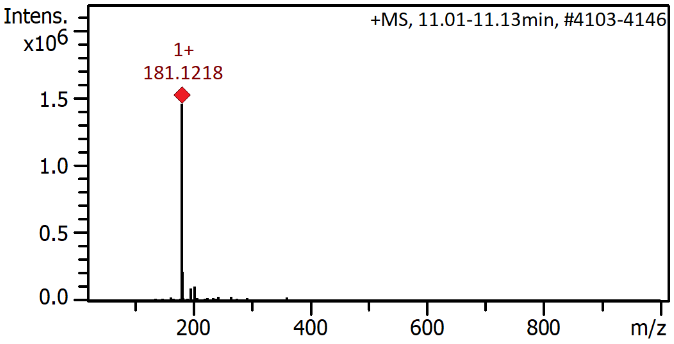

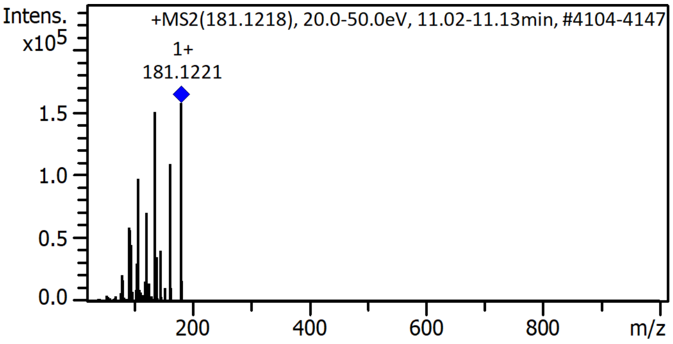

#	m/z	Res.	S/N	I	I %	FWHM
1	163.1116	11164	4753.9	24449	1.7	0.0146
2	181.1218	9914	282945.5	1455148	100.0	0.0183
3	182.1252	10724	42088.1	216453	14.9	0.0170
4	183.1281	10077	4307.6	22154	1.5	0.0182
5	197.1170	11053	17988.6	92513	6.4	0.0178
6	203.1037	11088	20394.7	104887	7.2	0.0183
7	225.1476	11751	4344.6	22343	1.5	0.0192
8	243.1586	11865	6247.9	32132	2.2	0.0205
9	265.1412	11878	5604.4	28822	2.0	0.0223
10	361.2372	12149	4648.9	23909	1.6	0.0297

#	m/z	Res.	S/N	I	I %	FWHM
1	91.0540	8957	17179.9	58902	37.5	0.0102
2	93.0698	8485	16577.5	56837	36.1	0.0110
3	95.0854	8941	13161.5	45125	28.7	0.0106
4	107.0852	8787	28445.9	97529	62.0	0.0122
5	121.1010	9342	20553.4	70469	44.8	0.0130
6	135.1166	8853	43772.0	150075	95.4	0.0153
7	139.0750	10249	10343.3	35463	22.6	0.0136
8	145.1009	10329	11868.4	40692	25.9	0.0140
9	163.1117	9816	31831.7	109137	69.4	0.0166
10	181.1221	9516	45864.9	157251	100.0	0.0190

Compound Spectrum List Report

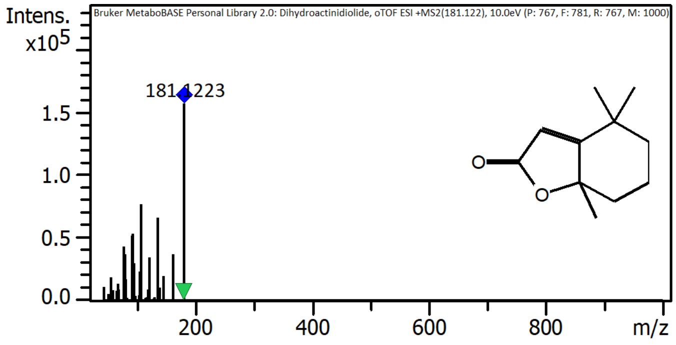

#	m/z	Res.	S/N	I	I %	FWHM
1	77.0390	3985	46.0	43401	27.6	0.0193
2	79.0547	4089	39.7	37426	23.8	0.0193
3	91.0545	4710	55.2	52050	33.1	0.0193
4	93.0701	4814	57.0	53780	34.2	0.0193
5	95.0855	4918	32.3	30507	19.4	0.0193
6	107.0854	5539	81.8	77210	49.1	0.0193
7	121.1003	6264	36.8	34752	22.1	0.0193
8	135.1165	6989	70.5	66517	42.3	0.0193
9	163.1114	8437	39.5	37268	23.7	0.0193
10	181.1223	9368	166.5	157094	100.0	0.0193

Cmpd 946, AutoMSn(191.1434), 11.08 min

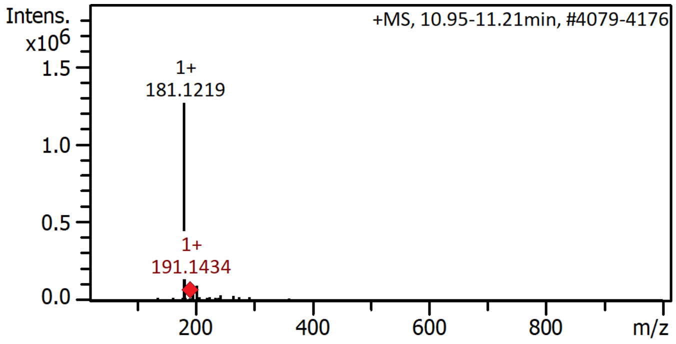

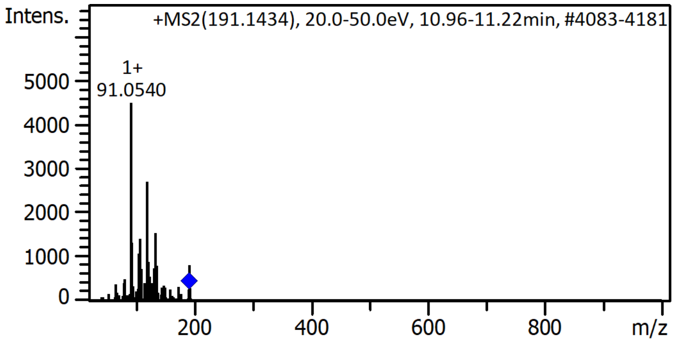

#	m/z	Res.	S/N	I	I %	FWHM
1	181.1219	9989	140668.7	1266018	100.0	0.0181
2	182.1253	10643	20777.9	187001	14.8	0.0171
3	197.1169	11176	10813.0	97317	7.7	0.0176
4	203.1035	11391	10821.3	97392	7.7	0.0178
5	207.1379	11295	2692.9	24236	1.9	0.0183
6	225.1478	11610	2722.9	24506	1.9	0.0194
7	243.1589	11407	3799.5	34196	2.7	0.0213
8	265.1406	12382	3254.4	29290	2.3	0.0214
9	275.2000	11622	2629.7	23667	1.9	0.0237
10	293.2098	12460	2537.9	22841	1.8	0.0235

#	m/z	Res.	S/N	I	I %	FWHM
1	91.0540	8845	373.9	4487	100.0	0.0103
2	93.0688	7995	110.5	1327	29.6	0.0116
3	105.0692	11006	90.5	1086	24.2	0.0095
4	107.0844	10961	118.3	1420	31.6	0.0098
5	119.0487	10660	225.0	2700	60.2	0.0112
6	121.1020	10719	74.1	889	19.8	0.0113
7	131.0847	9779	61.6	739	16.5	0.0134
8	133.1011	11233	128.5	1542	34.4	0.0118
9	135.1169	11305	65.8	790	17.6	0.0120
10	192.1376	7310	67.4	809	18.0	0.0263

Compound Spectrum List Report

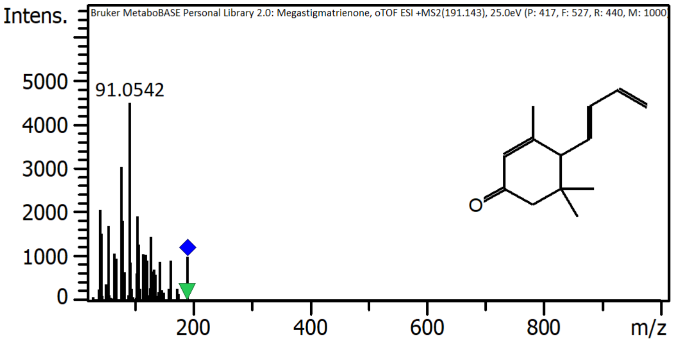

#	m/z	Res.	S/N	I	I %	FWHM
1	41.0395	4710	92.2	2069	46.1	0.0087
2	43.0190	4937	68.4	1535	34.2	0.0087
3	55.0545	6318	76.0	1705	38.0	0.0087
4	65.0393	7464	48.4	1086	24.2	0.0087
5	77.0390	8842	135.4	3038	67.8	0.0087
6	79.0548	9073	80.8	1813	40.4	0.0087
7	91.0542	10450	199.8	4483	100.0	0.0087
8	105.0698	12059	85.4	1916	42.7	0.0087
9	107.0851	12290	57.2	1283	28.6	0.0087
10	128.0610	14697	64.8	1454	32.4	0.0087

Cmpd 957, AutoMSn(265.1413), 11.22 min

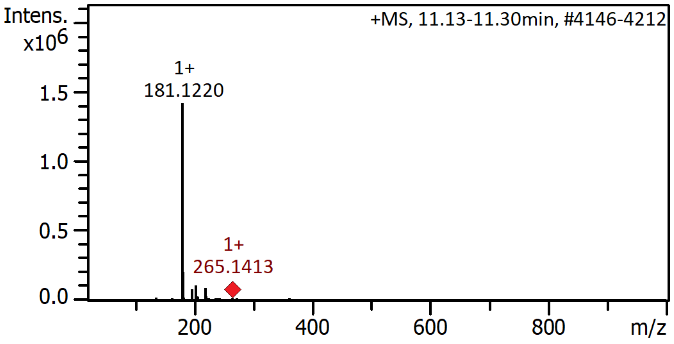

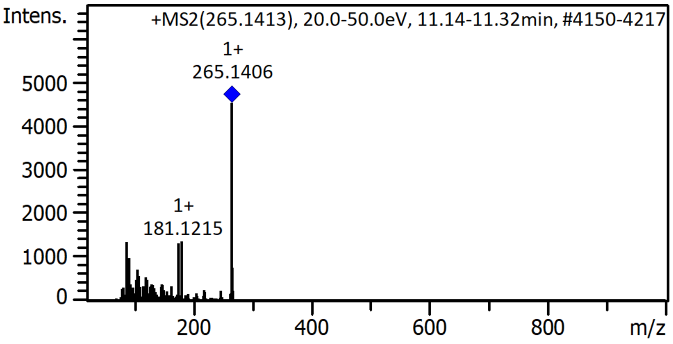

#	m/z	Res.	S/N	I	I %	FWHM
1	135.1166	10429	5890.3	21205	1.5	0.0130
2	181.1220	9893	393010.9	1414839	100.0	0.0183
3	182.1255	10772	56737.2	204254	14.4	0.0169
4	183.1285	10172	5411.9	19483	1.4	0.0180
5	197.1171	11349	22790.1	82044	5.8	0.0174
6	203.1039	11250	30159.0	108573	7.7	0.0181
7	205.1220	11381	8524.9	30690	2.2	0.0180
8	207.1380	11213	6643.8	23918	1.7	0.0185
9	220.1330	11578	25377.0	91357	6.5	0.0190
10	221.1251	9203	8276.9	29797	2.1	0.0240

#	m/z	Res.	S/N	I	I %	FWHM
1	86.0598	8767	281.4	1351	29.8	0.0098
2	91.0546	8238	206.3	990	21.9	0.0111
3	103.0534	10798	98.5	473	10.4	0.0095
4	105.0699	8612	148.6	713	15.7	0.0122
5	107.0861	9852	116.4	559	12.3	0.0109
6	119.0849	9635	112.2	539	11.9	0.0124
7	175.0385	11274	277.0	1330	29.4	0.0155
8	181.1215	12438	284.8	1367	30.2	0.0146
9	265.1406	12408	943.4	4528	100.0	0.0214
10	266.1444	13565	156.0	749	16.5	0.0196

Compound Spectrum List Report

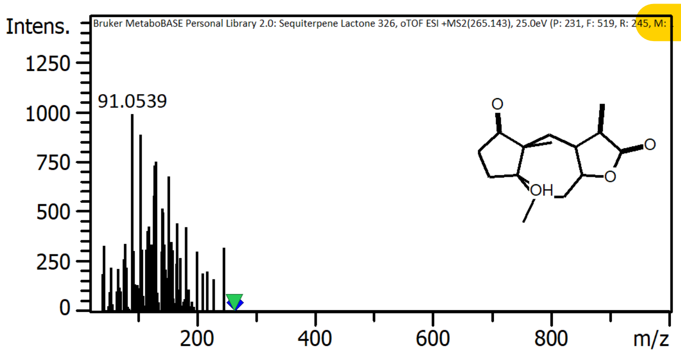

#	m/z	Res.	S/N	I	I %	FWHM
1	91.0539	3928	199.8	989	100.0	0.0232
2	105.0695	4533	179.0	886	89.6	0.0232
3	119.0851	5137	86.4	428	43.2	0.0232
4	128.0621	5525	117.6	582	58.9	0.0232
5	129.0688	5568	147.6	731	73.9	0.0232
6	131.0848	5655	151.8	751	76.0	0.0232
7	142.0777	6129	104.2	516	52.2	0.0232
8	143.0850	6173	100.8	499	50.5	0.0232
9	153.0701	6603	136.6	676	68.4	0.0232
10	168.0932	7252	89.6	444	44.8	0.0232

Cmpd 962, AutoMSn(295.2265), 11.34 min

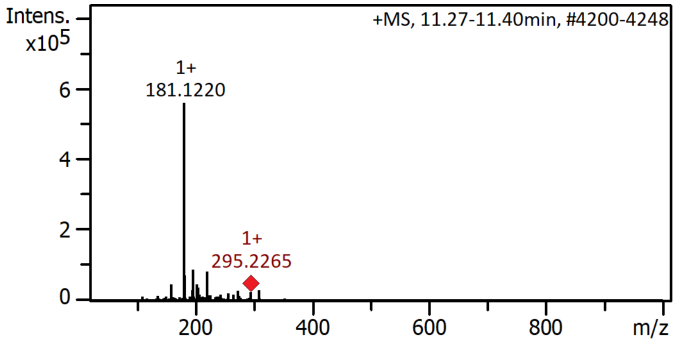

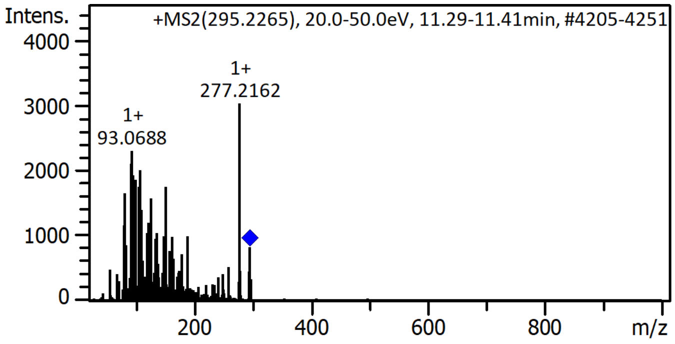

#	m/z	Res.	S/N	I	I %	FWHM
1	158.1534	10603	8830.2	45412	8.1	0.0149
2	181.1220	10153	108531.6	558163	100.0	0.0178
3	182.1255	11108	13731.7	70620	12.7	0.0164
4	195.1376	11178	5932.1	30508	5.5	0.0175
5	197.1168	11344	16886.6	86845	15.6	0.0174
6	203.1038	11269	8834.3	45434	8.1	0.0180
7	205.1223	11704	7228.4	37174	6.7	0.0175
8	220.1332	11495	16119.5	82900	14.9	0.0192
9	273.1619	10868	5515.3	28364	5.1	0.0251
10	309.0867	12448	5685.5	29240	5.2	0.0248

#	m/z	Res.	S/N	I	I %	FWHM
1	81.0702	8155	242.0	1660	54.7	0.0099
2	91.0537	8517	308.1	2113	69.6	0.0107
3	93.0688	9057	335.9	2303	75.9	0.0103
4	95.0860	9549	282.1	1935	63.7	0.0100
5	99.0801	8933	271.9	1865	61.4	0.0111
6	105.0688	8913	256.0	1755	57.8	0.0118
7	107.0854	9736	293.7	2014	66.3	0.0110
8	125.0952	10734	230.5	1581	52.1	0.0117
9	151.1113	10836	255.3	1751	57.7	0.0139
10	277.2162	12227	442.8	3036	100.0	0.0227

Compound Spectrum List Report

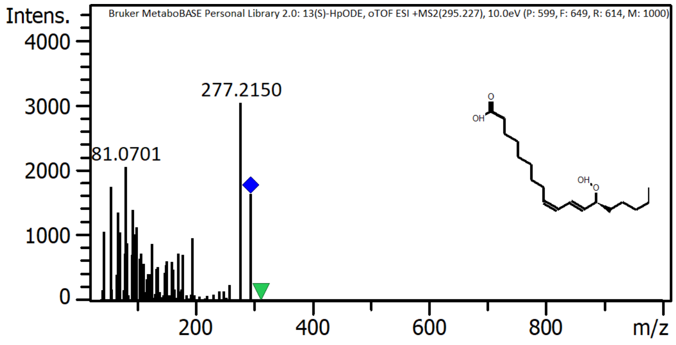

#	m/z	Res.	S/N	I	I %	FWHM
1	43.0547	1734	69.8	1060	34.9	0.0248
2	55.0548	2217	115.6	1755	57.9	0.0248
3	67.0549	2700	89.4	1357	44.7	0.0248
4	71.0856	2862	69.4	1054	34.7	0.0248
5	81.0701	3264	135.0	2049	67.6	0.0248
6	93.0694	3747	92.0	1397	46.0	0.0248
7	95.0860	3829	67.6	1026	33.8	0.0248
8	99.0797	3989	74.4	1129	37.2	0.0248
9	277.2150	11162	199.8	3033	100.0	0.0248
10	295.2250	11887	108.4	1646	54.3	0.0248

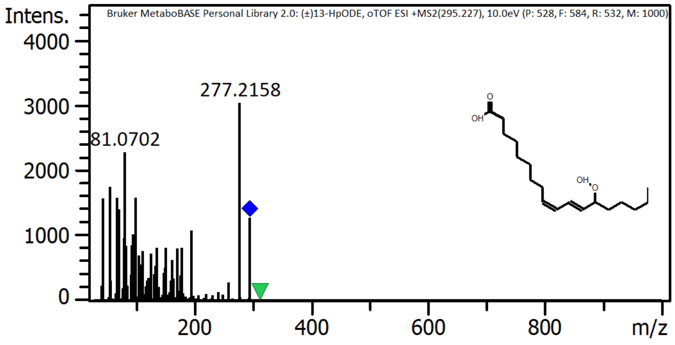

#	m/z	Res.	S/N	I	I %	FWHM
1	43.0552	1734	80.8	1227	40.4	0.0248
2	43.0554	1734	103.4	1570	51.8	0.0248
3	55.0549	2217	115.0	1746	57.6	0.0248
4	67.0551	2700	104.0	1579	52.1	0.0248
5	71.0861	2862	92.4	1403	46.2	0.0248
6	81.0702	3264	150.4	2283	75.3	0.0248
7	99.0805	3990	104.6	1588	52.4	0.0248
8	195.1366	7857	71.2	1081	35.6	0.0248
9	277.2158	11162	199.8	3033	100.0	0.0248
10	295.2274	11887	84.2	1278	42.1	0.0248

Cmpd 972, AutoMSn(365.2295), 11.49 min

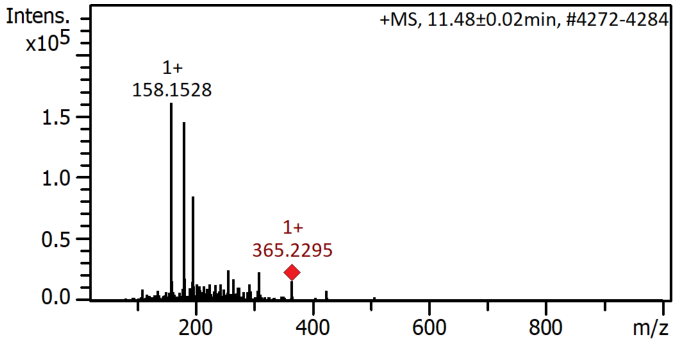

#	m/z	Res.	S/N	I	I %	FWHM
1	158.1528	10445	13383.9	160607	100.0	0.0151
2	159.1560	11020	1315.2	15783	9.8	0.0144
3	181.1211	10972	12090.7	145088	90.3	0.0165
4	182.1241	11289	1484.7	17816	11.1	0.0161
5	195.1359	11248	1270.1	15241	9.5	0.0173
6	197.1159	11251	7073.2	84878	52.8	0.0175
7	257.1263	11284	2039.3	24471	15.2	0.0228
8	265.1396	11713	1470.9	17651	11.0	0.0226
9	309.0851	11932	1906.4	22877	14.2	0.0259
10	365.2295	11807	1317.6	15811	9.8	0.0309

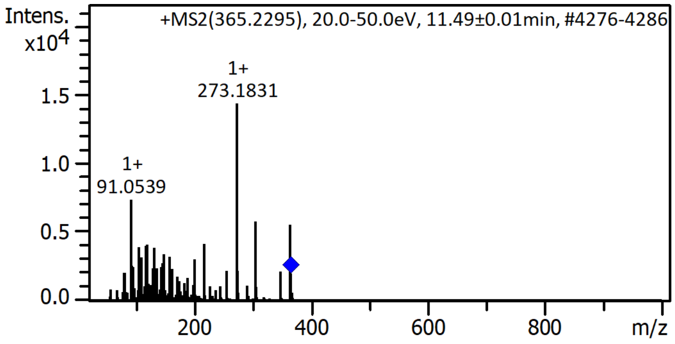

#	m/z	Res.	S/N	I	I %	FWHM
1	91.0539	9320	462.4	7398	51.6	0.0098
2	105.0693	10547	247.1	3953	27.6	0.0100
3	117.0683	10873	252.1	4034	28.1	0.0108
4	119.0841	9308	258.4	4135	28.8	0.0128
5	131.0835	10073	243.6	3897	27.2	0.0130
6	147.0795	10040	213.6	3418	23.8	0.0146
7	217.1562	10849	260.8	4173	29.1	0.0200
8	273.1831	11690	896.3	14340	100.0	0.0234
9	305.2461	13829	361.0	5776	40.3	0.0221
10	364.3189	14054	347.6	5562	38.8	0.0259

Compound Spectrum List Report

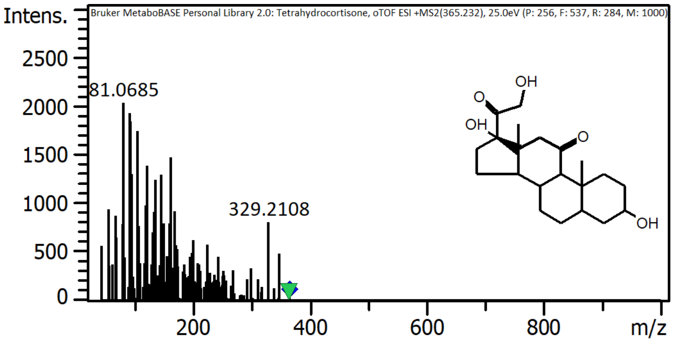

#	m/z	Res.	S/N	I	I %	FWHM
1	81.0685	3354	199.8	2027	100.0	0.0242
2	91.0520	3767	190.0	1928	95.1	0.0242
3	93.0696	3850	181.6	1843	90.9	0.0242
4	95.0827	3933	128.2	1301	64.2	0.0242
5	105.0722	4347	171.4	1739	85.8	0.0242
6	119.0862	4926	96.4	978	48.2	0.0242
7	121.0657	5008	136.6	1386	68.4	0.0242
8	135.0781	5588	122.2	1240	61.2	0.0242
9	145.1002	6002	127.8	1297	64.0	0.0242
10	161.0972	6664	145.2	1473	72.7	0.0242

Cmpd 973, AutoMSn(293.2100), 11.49 min

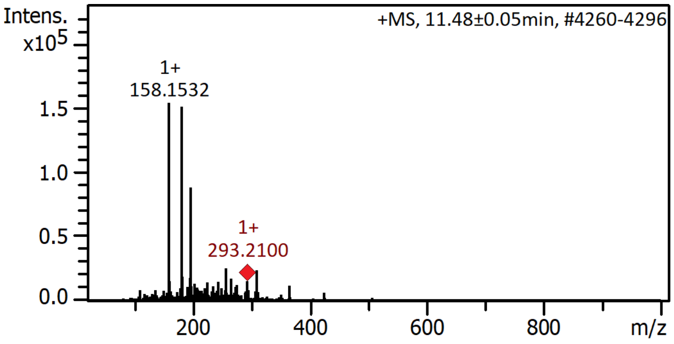

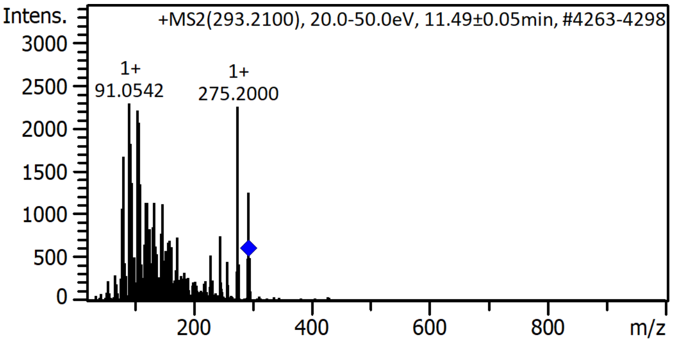

#	m/z	Res.	S/N	I	I %	FWHM
1	158.1532	10584	25676.7	154060	100.0	0.0149
2	159.1561	11481	2551.8	15311	9.9	0.0139
3	181.1216	10944	25142.5	150855	97.9	0.0165
4	182.1251	11149	3082.8	18497	12.0	0.0163
5	195.1367	11603	2969.5	17817	11.6	0.0168
6	197.1165	11133	14710.7	88264	57.3	0.0177
7	257.1274	11680	4182.5	25095	16.3	0.0220
8	265.1402	11800	2891.6	17349	11.3	0.0225
9	293.2100	11073	2505.6	15033	9.8	0.0265
10	309.0855	11904	3964.7	23788	15.4	0.0260

#	m/z	Res.	S/N	I	I %	FWHM
1	81.0702	8958	209.5	1676	73.1	0.0090
2	91.0542	8958	286.7	2293	100.0	0.0102
3	93.0698	8384	227.5	1820	79.4	0.0111
4	95.0853	9694	170.8	1366	59.6	0.0098
5	105.0691	9200	276.3	2210	96.4	0.0114
6	107.0848	9651	258.9	2071	90.3	0.0111
7	109.0652	8322	169.1	1353	59.0	0.0131
8	109.1005	8857	158.3	1266	55.2	0.0123
9	275.2000	12743	281.2	2250	98.1	0.0216
10	293.1192	12031	157.4	1259	54.9	0.0244

Compound Spectrum List Report

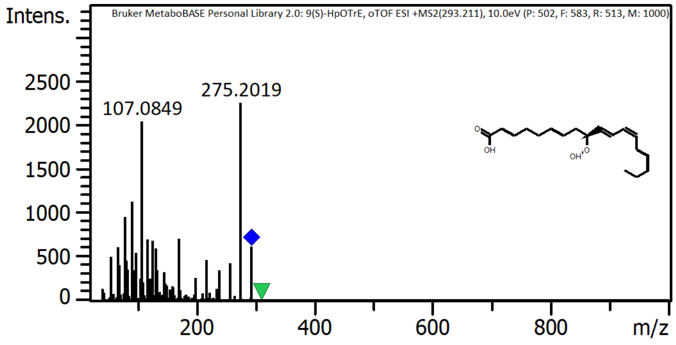

#	m/z	Res.	S/N	I	I %	FWHM
1	67.0547	8613	54.4	612	27.2	0.0078
2	79.0548	10155	85.0	956	42.5	0.0078
3	91.0538	11696	100.6	1132	50.4	0.0078
4	107.0849	13755	181.2	2038	90.7	0.0078
5	117.0699	15038	62.2	700	31.1	0.0078
6	125.0948	16068	61.4	691	30.7	0.0078
7	131.0845	16838	53.2	598	26.6	0.0078
8	171.1027	21978	62.8	706	31.4	0.0078
9	275.2019	35350	199.8	2247	100.0	0.0078
10	293.2087	37663	55.2	621	27.6	0.0078

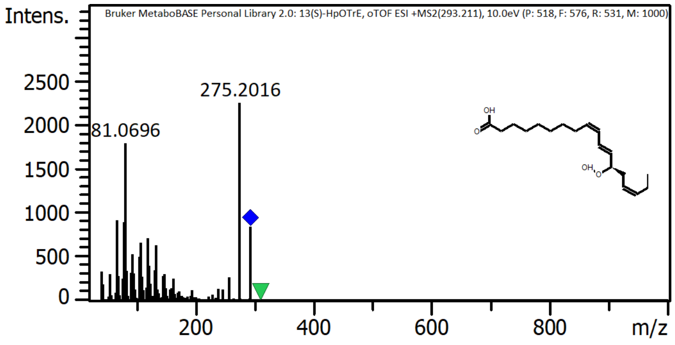

#	m/z	Res.	S/N	I	I %	FWHM
1	67.0547	8613	74.2	835	37.1	0.0078
2	67.0547	8613	81.8	920	40.9	0.0078
3	79.0542	10155	80.0	900	40.0	0.0078
4	81.0696	10413	159.4	1793	79.8	0.0078
5	93.0695	11955	47.6	535	23.8	0.0078
6	107.0848	13755	59.4	668	29.7	0.0078
7	119.0851	15297	63.4	713	31.7	0.0078
8	133.0999	17097	56.8	639	28.4	0.0078
9	275.2016	35350	199.8	2247	100.0	0.0078
10	293.2108	37663	75.6	850	37.8	0.0078

Cmpd 976, AutoMSn(181.1216), 11.55 min

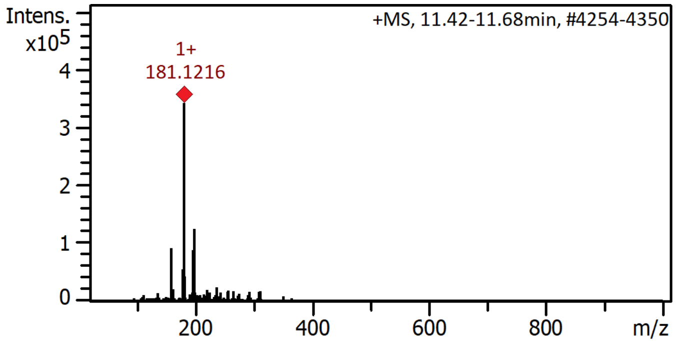

#	m/z	Res.	S/N	I	I %	FWHM
1	158.1531	10641	23125.4	92502	27.1	0.0149
2	161.1316	10635	5063.6	20254	5.9	0.0152
3	179.1423	10809	13798.1	55192	16.1	0.0166
4	181.1216	10467	85462.1	341848	100.0	0.0173
5	182.1252	10835	10607.3	42429	12.4	0.0168
6	197.1164	11031	22305.7	89223	26.1	0.0179
7	197.1517	10915	10410.8	41643	12.2	0.0181
8	199.1320	10748	31444.4	125777	36.8	0.0185
9	221.1139	11666	4789.4	19158	5.6	0.0190
10	237.1470	11172	5985.9	23943	7.0	0.0212

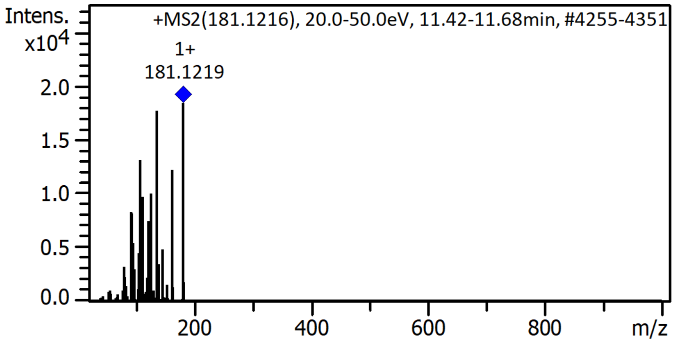

#	m/z	Res.	S/N	I	I %	FWHM
1	91.0540	9135	1961.9	8283	45.0	0.0100
2	93.0697	8947	1937.6	8181	44.4	0.0104
3	95.0852	9331	1294.2	5465	29.7	0.0102
4	107.0850	9347	3105.7	13113	71.2	0.0115
5	111.0440	9251	2311.4	9759	53.0	0.0120
6	121.1007	9929	1764.7	7451	40.5	0.0122
7	125.0595	10121	2381.4	10055	54.6	0.0124
8	135.1165	10304	4197.9	17725	96.3	0.0131
9	163.1113	10737	2891.9	12210	66.3	0.0152
10	181.1219	11042	4359.6	18407	100.0	0.0164

Compound Spectrum List Report

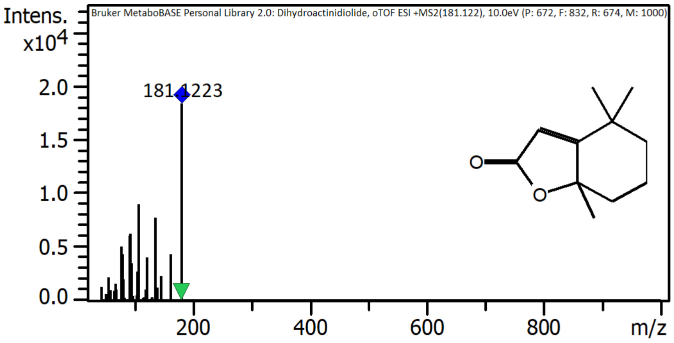

#	m/z	Res.	S/N	I	I %	FWHM
1	77.0390	4205	46.0	5080	27.6	0.0183
2	79.0547	4315	39.7	4381	23.8	0.0183
3	91.0545	4970	55.2	6093	33.1	0.0183
4	93.0701	5080	57.0	6295	34.2	0.0183
5	95.0855	5191	32.3	3571	19.4	0.0183
6	107.0854	5846	81.8	9038	49.1	0.0183
7	121.1003	6611	36.8	4068	22.1	0.0183
8	135.1165	7376	70.5	7786	42.3	0.0183
9	163.1114	8904	39.5	4362	23.7	0.0183
10	181.1223	9887	166.5	18389	100.0	0.0183

Cmpd 978, AutoMSn(265.1409), 11.57 min

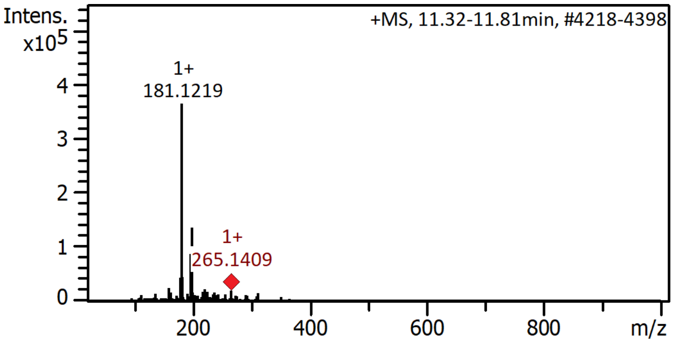

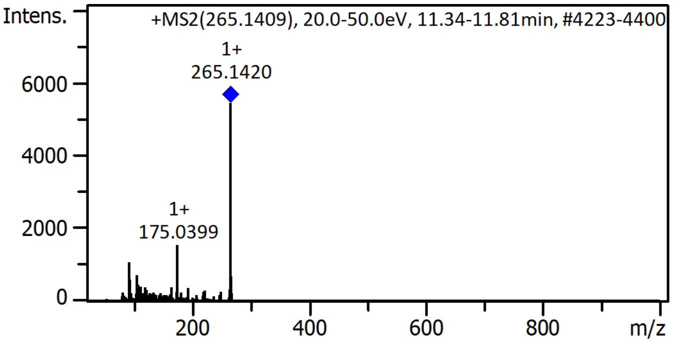

#	m/z	Res.	S/N	I	I %	FWHM
1	158.1538	10405	6680.8	24051	6.6	0.0152
2	179.1425	11085	12064.6	43433	11.9	0.0162
3	181.1219	10302	101437.2	365174	100.0	0.0176
4	182.1253	11096	12438.3	44778	12.3	0.0164
5	197.1167	11261	24531.1	88312	24.2	0.0175
6	197.1513	10674	6275.1	22591	6.2	0.0185
7	199.1322	10826	37777.8	136000	37.2	0.0184
8	221.1160	11444	6084.3	21904	6.0	0.0193
9	225.1478	11186	4700.6	16922	4.6	0.0201
10	265.1409	11905	5286.9	19033	5.2	0.0223

#	m/z	Res.	S/N	I	I %	FWHM
1	91.0543	8458	224.9	1079	19.9	0.0108
2	93.0705	9148	123.5	593	10.9	0.0102
3	105.0692	10582	149.0	715	13.2	0.0099
4	107.0851	11084	95.1	457	8.4	0.0097
5	111.0453	11812	81.1	389	7.2	0.0094
6	119.0856	12526	78.8	378	7.0	0.0095
7	165.1265	14328	79.8	383	7.1	0.0115
8	175.0399	11759	323.2	1552	28.6	0.0149
9	265.1420	11641	1132.0	5434	100.0	0.0228
10	266.1443	12464	142.0	682	12.5	0.0214

Compound Spectrum List Report

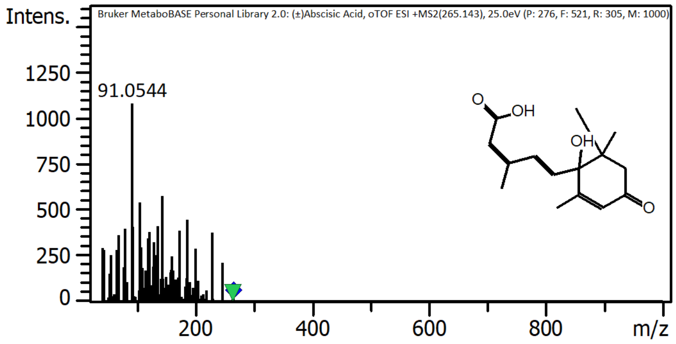

#	m/z	Res.	S/N	I	I %	FWHM
1	79.0548	3496	73.8	398	36.9	0.0226
2	91.0544	4026	199.8	1078	100.0	0.0226
3	93.0706	4115	75.8	409	37.9	0.0226
4	105.0702	4646	100.4	542	50.3	0.0226
5	121.0635	5353	70.8	382	35.4	0.0226
6	135.0806	5973	76.2	411	38.1	0.0226
7	135.0822	5973	75.0	405	37.5	0.0226
8	143.0826	6327	106.8	576	53.5	0.0226
9	173.1307	7655	71.8	388	35.9	0.0226
10	187.1118	8273	83.2	449	41.6	0.0226

Cmpd 982, AutoMSn(235.1679), 11.60 min

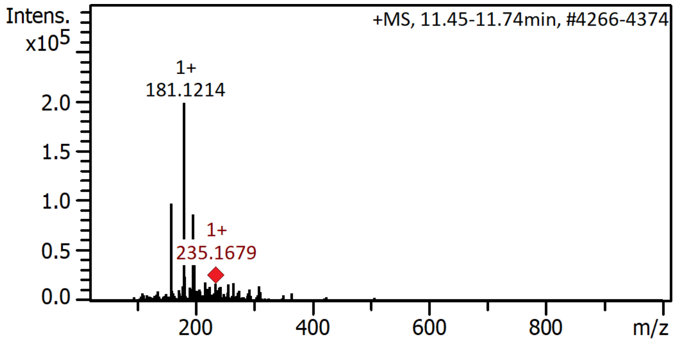

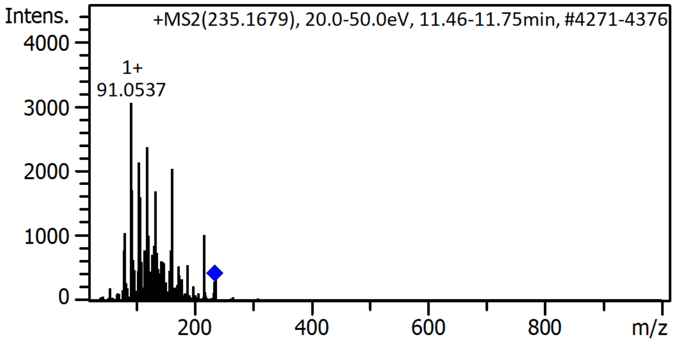

#	m/z	Res.	S/N	I	I %	FWHM
1	158.1529	10636	16311.3	97868	49.4	0.0149
2	181.1214	10850	32996.8	197981	100.0	0.0167
3	182.1247	11796	3952.2	23713	12.0	0.0154
4	197.1163	11253	14497.4	86985	43.9	0.0175
5	199.1317	10662	9122.1	54733	27.6	0.0187
6	217.1464	8367	3016.7	18100	9.1	0.0260
7	235.1679	11328	2825.6	16954	8.6	0.0208
8	257.1277	11229	2732.3	16394	8.3	0.0229
9	265.1403	11733	2957.9	17747	9.0	0.0226
10	309.0857	12285	2446.2	14677	7.4	0.0252

#	m/z	Res.	S/N	I	I %	FWHM
1	81.0693	9660	123.8	1052	34.4	0.0084
2	91.0537	8599	359.4	3055	100.0	0.0106
3	93.0691	9518	201.1	1710	56.0	0.0098
4	105.0693	10143	251.7	2139	70.0	0.0104
5	107.0850	10829	189.1	1608	52.6	0.0099
6	119.0850	9784	279.0	2372	77.6	0.0122
7	121.1010	9999	119.1	1012	33.1	0.0121
8	133.0998	10261	198.6	1688	55.3	0.0130
9	161.0943	11906	239.4	2035	66.6	0.0135
10	217.1514	9345	119.7	1017	33.3	0.0232

Compound Spectrum List Report

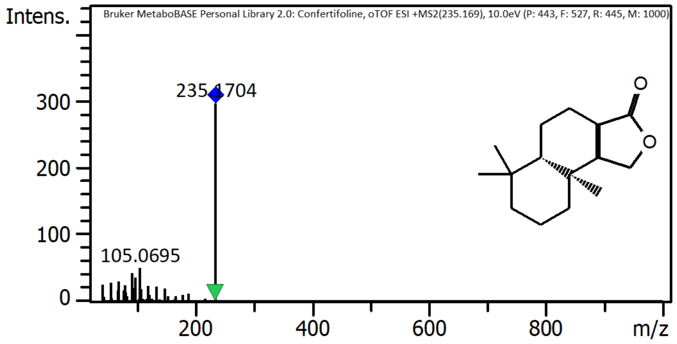

#	m/z	Res.	S/N	I	I %	FWHM
1	41.0399	4952	17.2	25	8.6	0.0083
2	55.0552	6644	19.0	28	9.5	0.0083
3	69.0704	8335	20.6	30	10.3	0.0083
4	79.0547	9540	16.4	24	8.2	0.0083
5	91.0546	10988	28.6	42	14.3	0.0083
6	97.0288	11709	24.0	36	12.0	0.0083
7	105.0695	12679	33.8	50	16.9	0.0083
8	119.0847	14370	15.6	23	7.8	0.0083
9	133.1008	16062	15.2	22	7.6	0.0083
10	235.1704	28379	199.8	296	100.0	0.0083

Cmpd 984, AutoMSn(191.1422), 11.63 min

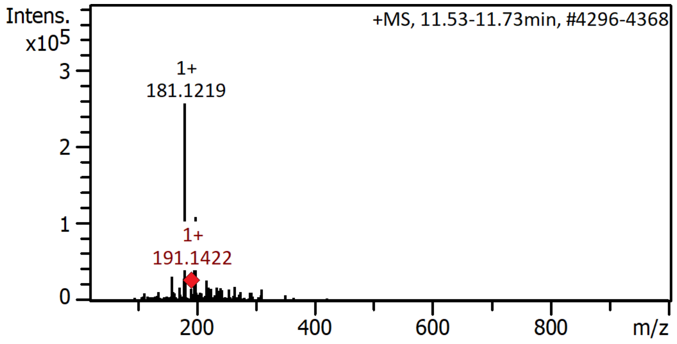

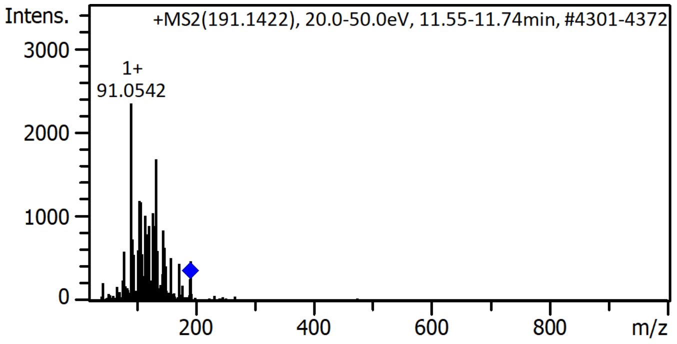

#	m/z	Res.	S/N	I	I %	FWHM
1	158.1536	10742	3417.3	30755	12.0	0.0147
2	172.1688	11553	1885.3	16968	6.6	0.0149
3	179.1423	10946	3185.1	28666	11.2	0.0164
4	181.1219	10535	28522.1	256699	100.0	0.0172
5	182.1252	11617	3490.6	31415	12.2	0.0157
6	197.1166	11267	9774.1	87967	34.3	0.0175
7	199.1322	10675	12121.2	109091	42.5	0.0187
8	217.1463	8526	2899.1	26092	10.2	0.0255
9	221.1141	11536	1904.4	17139	6.7	0.0192
10	265.1405	12053	1976.2	17786	6.9	0.0220

#	m/z	Res.	S/N	I	I %	FWHM
1	91.0542	10077	195.4	2345	100.0	0.0090
2	105.0686	9778	99.3	1192	50.8	0.0107
3	107.0841	9320	97.9	1175	50.1	0.0115
4	115.0548	10622	84.2	1011	43.1	0.0108
5	119.0867	10774	66.2	794	33.9	0.0111
6	121.1016	10163	74.7	896	38.2	0.0119
7	128.0610	12087	87.3	1047	44.7	0.0106
8	131.0850	11266	74.4	893	38.1	0.0116
9	133.1014	11473	140.3	1684	71.8	0.0116
10	145.0999	9100	70.1	842	35.9	0.0159

Compound Spectrum List Report

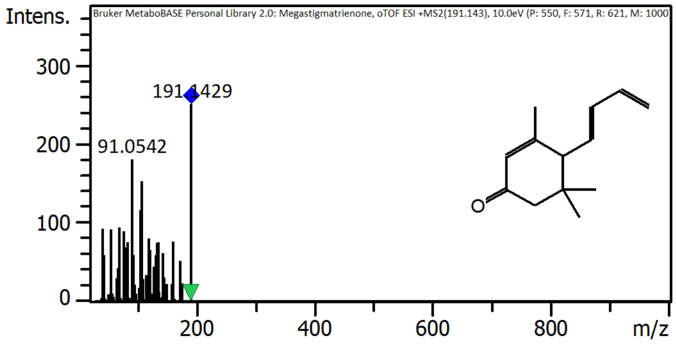

#	m/z	Res.	S/N	I	I %	FWHM
1	41.0394	4960	73.8	93	36.9	0.0083
2	55.0544	6654	73.4	92	36.7	0.0083
3	69.0337	8343	75.4	95	37.7	0.0083
4	77.0389	9311	71.2	89	35.6	0.0083
5	91.0542	11004	144.0	181	72.1	0.0083
6	105.0700	12698	93.0	117	46.5	0.0083
7	107.0850	12942	122.0	153	61.1	0.0083
8	119.0855	14392	64.4	81	32.2	0.0083
9	161.0963	19469	60.8	76	30.4	0.0083
10	191.1429	23101	199.8	251	100.0	0.0083

Cmpd 999, AutoMSn(229.1427), 11.89 min

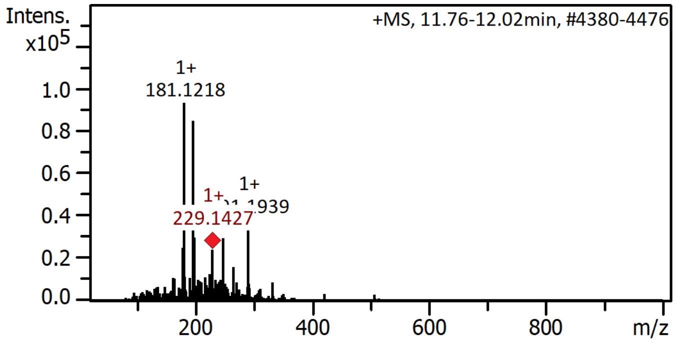

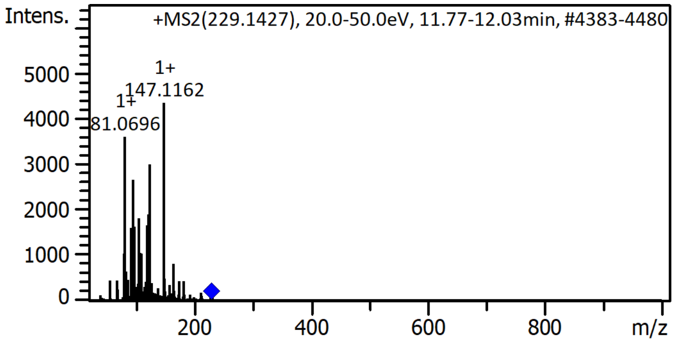

#	m/z	Res.	S/N	I	I %	FWHM
1	179.1420	11585	4177.2	25063	26.9	0.0155
2	181.1218	11112	15507.4	93045	100.0	0.0163
3	182.1247	11491	1885.8	11315	12.2	0.0158
4	197.1164	11568	14139.5	84837	91.2	0.0170
5	199.1323	10990	5006.3	30038	32.3	0.0181
6	225.1464	10809	2093.4	12561	13.5	0.0208
7	229.1427	11704	4015.2	24091	25.9	0.0196
8	248.1275	12285	4959.3	29756	32.0	0.0202
9	265.1404	12037	2644.3	15866	17.1	0.0220
10	291.1939	12473	6413.6	38482	41.4	0.0233

#	m/z	Res.	S/N	I	I %	FWHM
1	81.0696	8831	449.1	3593	82.8	0.0092
2	91.0531	7840	201.5	1612	37.2	0.0116
3	95.0855	8740	331.4	2651	61.1	0.0109
4	97.0642	9731	204.8	1639	37.8	0.0100
5	105.0694	9879	227.0	1816	41.9	0.0106
6	107.0848	10601	132.9	1064	24.5	0.0101
7	119.0851	9670	207.8	1663	38.3	0.0123
8	121.1012	9330	237.6	1901	43.8	0.0130
9	123.1166	10621	374.9	3000	69.2	0.0116
10	147.1162	10989	542.1	4337	100.0	0.0134

Compound Spectrum List Report

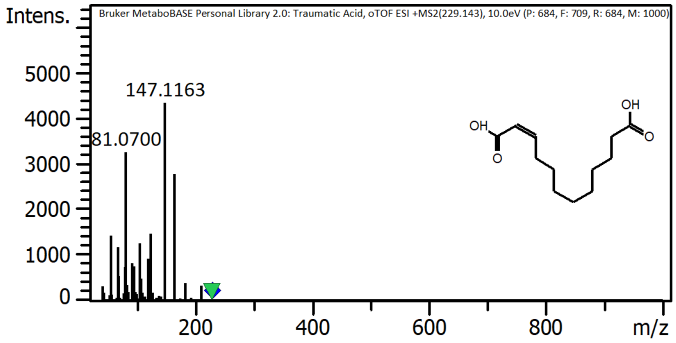

#	m/z	Res.	S/N	I	I %	FWHM
1	55.0187	3785	66.4	1440	33.2	0.0145
2	55.0544	3788	46.4	1006	23.2	0.0145
3	67.0543	4613	54.4	1180	27.2	0.0145
4	81.0700	5578	150.0	3253	75.1	0.0145
5	91.0536	6264	38.6	837	19.3	0.0145
6	105.0696	7229	58.6	1271	29.3	0.0145
7	119.0846	8193	43.2	937	21.6	0.0145
8	123.1163	8470	68.0	1475	34.0	0.0145
9	147.1163	10122	199.8	4332	100.0	0.0145
10	165.1264	11361	128.0	2776	64.1	0.0145

Cmpd 1000, AutoMSn(233.1533), 11.90 min

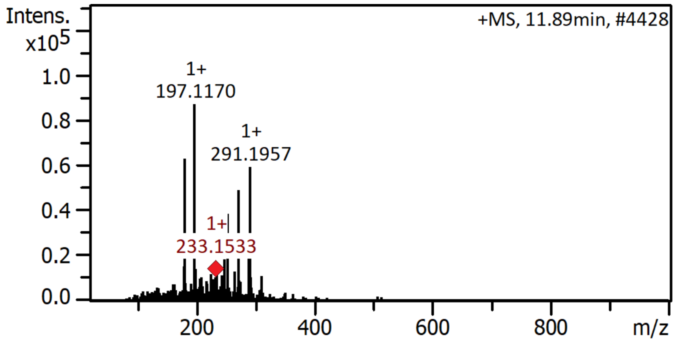

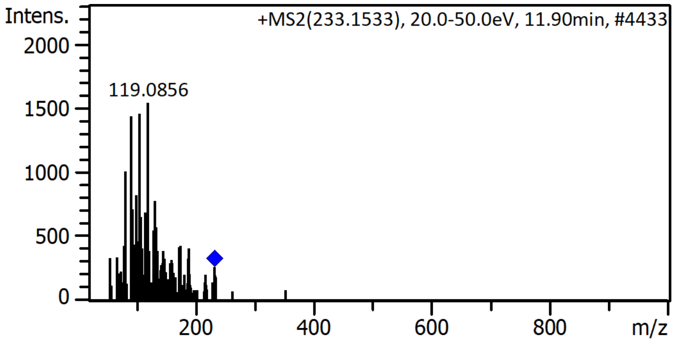

#	m/z	Res.	S/N	I	I %	FWHM
1	179.1420	11000	421.9	15189	17.4	0.0163
2	181.1225	11292	1751.0	63035	72.2	0.0160
3	197.1170	11769	2423.6	87249	100.0	0.0167
4	199.1315	12079	385.9	13894	15.9	0.0165
5	248.1285	10969	515.1	18543	21.3	0.0226
6	253.2161	11852	1072.3	38602	44.2	0.0214
7	265.1414	11217	355.0	12779	14.6	0.0236
8	271.2265	12298	1366.5	49195	56.4	0.0221
9	289.2363	11759	550.8	19829	22.7	0.0246
10	291.1957	12089	1646.2	59264	67.9	0.0241

#	m/z	Res.	S/N	I	I %	FWHM
1	81.0705	9050	20.5	1006	65.4	0.0090
2	91.0528	10017	29.3	1437	93.4	0.0091
3	93.0684	11304	14.6	713	46.3	0.0082
4	99.0796	8680	16.8	825	53.6	0.0114
5	105.0715	7265	29.7	1455	94.5	0.0145
6	107.0843	12442	13.3	654	42.5	0.0086
7	115.0583	10417	14.1	689	44.8	0.0110
8	119.0856	9986	31.4	1539	100.0	0.0119
9	131.0839	11120	15.9	778	50.6	0.0118
10	133.0989	13271	11.7	573	37.2	0.0100

Compound Spectrum List Report

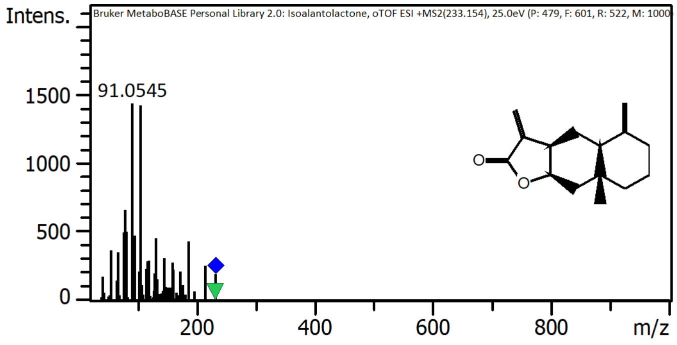

#	m/z	Res.	S/N	I	I %	FWHM
1	55.0546	6190	51.8	372	25.9	0.0089
2	67.0548	7539	49.4	355	24.7	0.0089
3	77.0383	8661	69.6	500	34.8	0.0089
4	79.0546	8888	92.6	665	46.3	0.0089
5	81.0698	9114	70.4	506	35.2	0.0089
6	91.0545	10237	199.8	1436	100.0	0.0089
7	95.0853	10690	66.4	477	33.2	0.0089
8	105.0699	11813	197.8	1421	99.0	0.0089
9	131.0852	14737	64.0	460	32.0	0.0089
10	187.1478	21040	60.4	434	30.2	0.0089

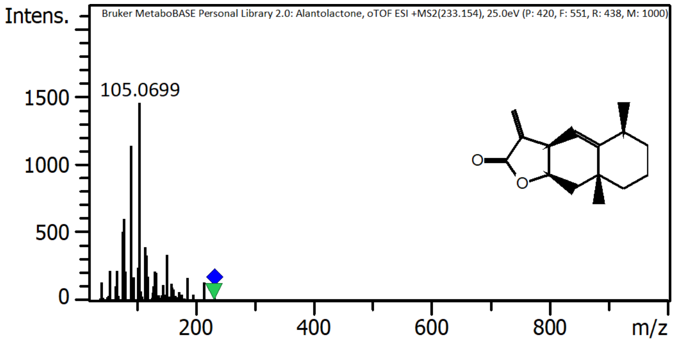

#	m/z	Res.	S/N	I	I %	FWHM
1	55.0553	6190	29.8	217	14.9	0.0089
2	67.0548	7539	30.2	220	15.1	0.0089
3	77.0392	8661	69.8	508	34.9	0.0089
4	79.0548	8888	83.0	604	41.5	0.0089
5	91.0545	10237	156.6	1139	78.4	0.0089
6	103.0545	11586	33.0	240	16.5	0.0089
7	105.0699	11813	199.8	1454	100.0	0.0089
8	115.0539	12935	54.6	397	27.3	0.0089
9	117.0694	13162	46.2	336	23.1	0.0089
10	151.0752	16985	47.0	342	23.5	0.0089

Cmpd 1008, AutoMSn(181.1218), 12.00 min

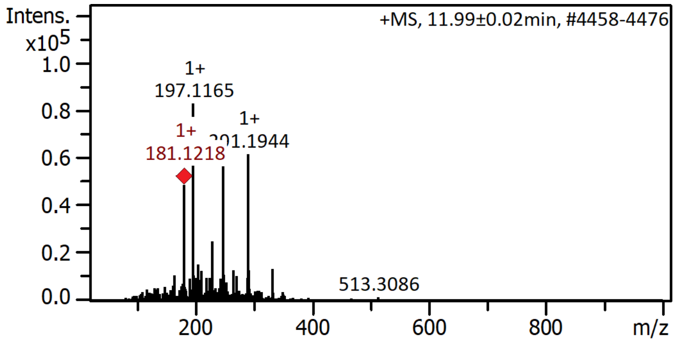

#	m/z	Res.	S/N	I	I %	FWHM
1	181.1218	11020	5421.9	48797	59.0	0.0164
2	197.1165	11456	9190.6	82716	100.0	0.0172
3	205.1222	10801	1697.6	15278	18.5	0.0190
4	211.0864	10937	1400.9	12608	15.2	0.0193
5	229.1429	11707	2782.9	25046	30.3	0.0196
6	248.1276	12452	6281.2	56531	68.3	0.0199
7	265.1410	12635	1418.6	12767	15.4	0.0210
8	291.1944	12424	6845.6	61611	74.5	0.0234
9	292.1982	11919	1408.1	12673	15.3	0.0245
10	332.3302	12488	1478.8	13309	16.1	0.0266

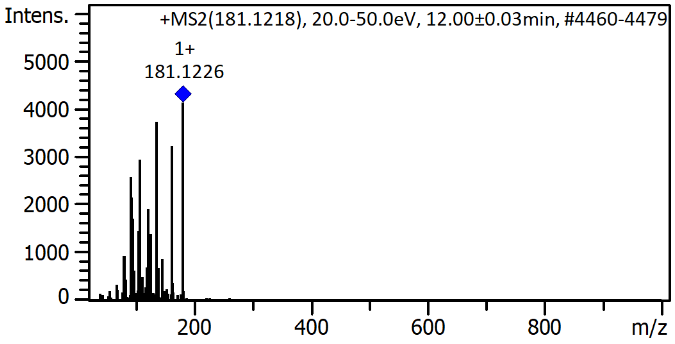

#	m/z	Res.	S/N	I	I %	FWHM
1	91.0542	8632	206.2	2577	62.5	0.0105
2	93.0693	9886	171.8	2147	52.1	0.0094
3	95.0854	9511	137.0	1712	41.5	0.0100
4	105.0690	10950	117.0	1463	35.5	0.0096
5	107.0844	10173	235.5	2944	71.4	0.0105
6	121.1007	10786	153.0	1913	46.4	0.0112
7	125.0586	11387	111.6	1396	33.9	0.0110
8	135.1169	8973	298.0	3725	90.4	0.0151
9	163.1108	11347	257.6	3220	78.1	0.0144
10	181.1226	11350	329.7	4122	100.0	0.0160

Compound Spectrum List Report

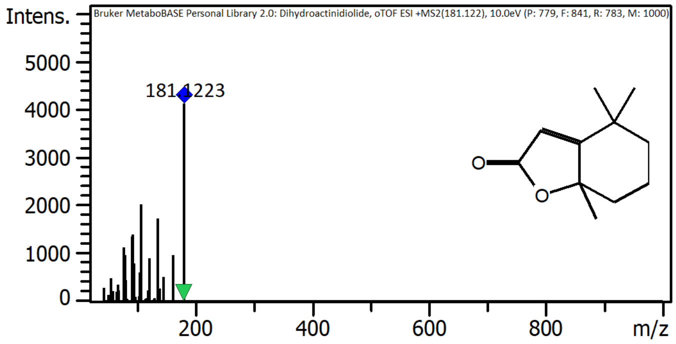

#	m/z	Res.	S/N	I	I %	FWHM
1	77.0390	4421	46.0	1138	27.6	0.0174
2	79.0547	4537	39.7	981	23.8	0.0174
3	91.0545	5226	55.2	1364	33.1	0.0174
4	93.0701	5341	57.0	1410	34.2	0.0174
5	95.0855	5457	32.3	800	19.4	0.0174
6	107.0854	6146	81.8	2024	49.1	0.0174
7	121.1003	6950	36.8	911	22.1	0.0174
8	135.1165	7754	70.5	1743	42.3	0.0174
9	163.1114	9361	39.5	977	23.7	0.0174
10	181.1223	10395	166.5	4117	100.0	0.0174

Cmpd 1018, AutoMSn(203.1788), 12.15 min

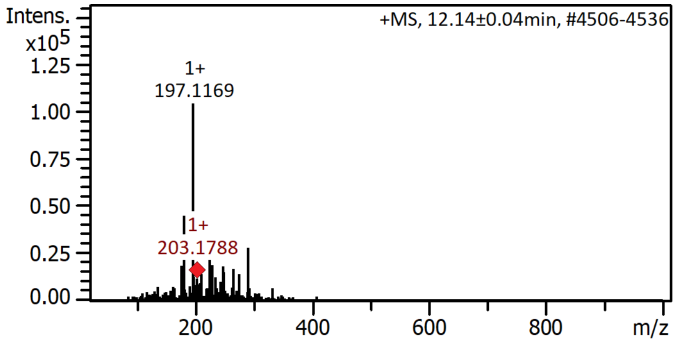

#	m/z	Res.	S/N	I	I %	FWHM
1	177.0541	10699	1541.6	18499	17.7	0.0165
2	181.1222	10728	3744.5	44934	43.1	0.0169
3	197.1169	11459	8686.8	104242	100.0	0.0172
4	225.1484	11817	2828.2	33938	32.6	0.0191
5	229.1431	12162	1553.6	18643	17.9	0.0188
6	248.1281	12307	1505.3	18064	17.3	0.0202
7	249.1463	10009	1266.2	15195	14.6	0.0249
8	265.1405	13108	1383.9	16607	15.9	0.0202
9	275.1998	12203	1163.9	13967	13.4	0.0226
10	291.1944	12410	2361.1	28333	27.2	0.0235

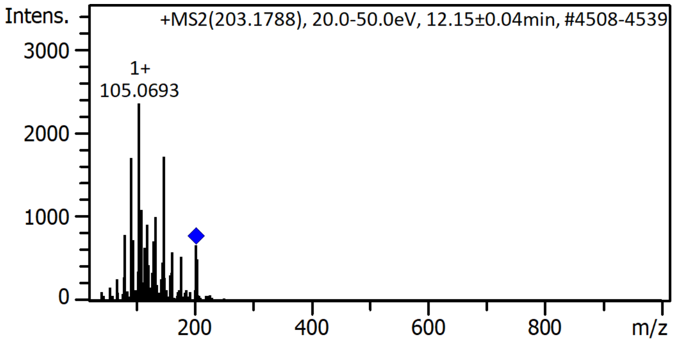

#	m/z	Res.	S/N	I	I %	FWHM
1	81.0702	10013	49.2	788	33.5	0.0081
2	91.0554	7275	106.5	1704	72.4	0.0125
3	95.0863	10426	45.4	727	30.9	0.0091
4	105.0693	10875	147.0	2352	100.0	0.0097
5	107.0853	10575	48.0	768	32.6	0.0101
6	109.1021	10816	67.9	1086	46.2	0.0101
7	119.0821	10397	57.1	914	38.8	0.0115
8	130.0695	9442	44.5	712	30.3	0.0138
9	133.1028	8512	62.7	1004	42.7	0.0156
10	147.1160	13159	107.5	1720	73.1	0.0112

Compound Spectrum List Report

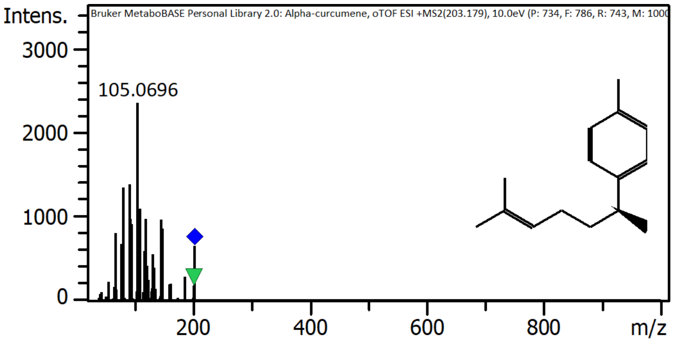

#	m/z	Res.	S/N	I	I %	FWHM
1	81.0697	8290	114.8	1350	57.5	0.0098
2	91.0545	9311	118.0	1388	59.1	0.0098
3	93.0690	9517	82.8	974	41.4	0.0098
4	95.0851	9723	78.0	917	39.0	0.0098
5	105.0696	10745	199.8	2350	100.0	0.0098
6	107.0844	10951	93.4	1099	46.7	0.0098
7	109.1014	11157	93.4	1099	46.7	0.0098
8	119.0845	12178	82.8	974	41.4	0.0098
9	145.0992	14838	82.2	967	41.1	0.0098
10	147.1150	15044	73.2	861	36.6	0.0098

Cmpd 1033, AutoMSn(191.1430), 12.33 min

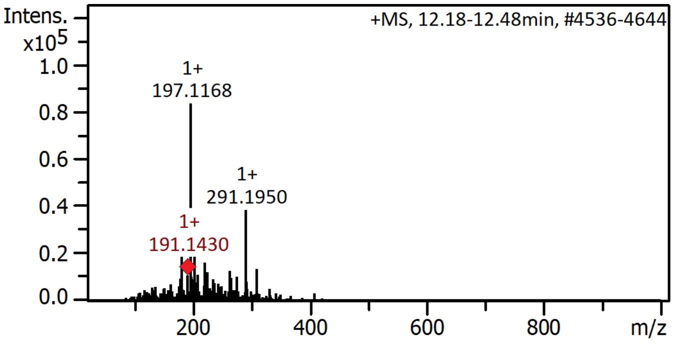

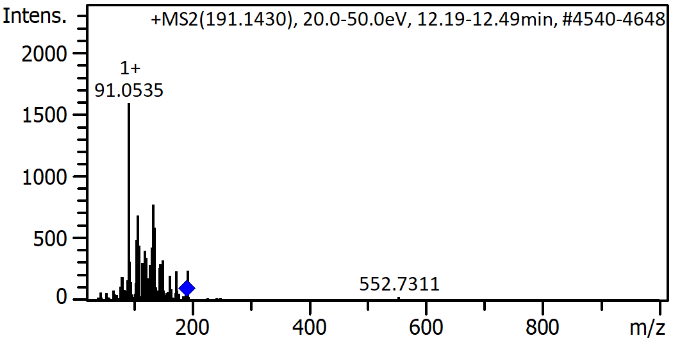

#	m/z	Res.	S/N	I	I %	FWHM
1	181.1222	11285	6889.2	35430	42.5	0.0160
2	191.1430	11786	2064.0	10615	12.7	0.0162
3	197.1168	11573	16220.4	83419	100.0	0.0170
4	203.1791	11840	5342.0	27473	32.9	0.0172
5	209.1534	11637	2146.3	11038	13.2	0.0180
6	221.1892	11945	3207.3	16494	19.8	0.0185
7	225.1482	11009	2354.7	12110	14.5	0.0205
8	263.1637	11476	2468.6	12696	15.2	0.0229
9	291.1950	12296	7519.4	38671	46.4	0.0237
10	309.2048	11789	2614.0	13443	16.1	0.0262

#	m/z	Res.	S/N	I	I %	FWHM
1	91.0535	9323	227.0	1589	100.0	0.0098
2	105.0703	9039	70.0	490	30.8	0.0116
3	107.0857	10875	98.5	689	43.4	0.0098
4	109.1006	10395	63.5	445	28.0	0.0105
5	119.0502	10029	57.5	402	25.3	0.0119
6	121.1000	11898	50.0	350	22.0	0.0102
7	131.0847	11858	61.3	429	27.0	0.0111
8	133.1006	10530	110.8	776	48.8	0.0126
9	135.1150	11340	84.1	589	37.1	0.0119
10	149.1338	12866	47.0	329	20.7	0.0116

Compound Spectrum List Report

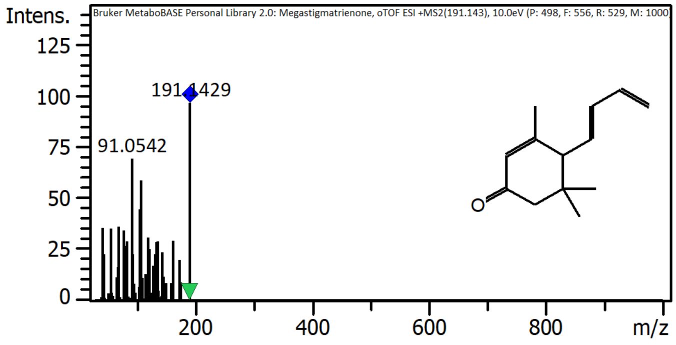

#	m/z	Res.	S/N	I	I %	FWHM
1	41.0394	5271	73.8	36	36.9	0.0078
2	55.0544	7071	73.4	35	36.7	0.0078
3	69.0337	8866	75.4	36	37.7	0.0078
4	77.0389	9894	71.2	34	35.6	0.0078
5	91.0542	11694	144.0	69	72.1	0.0078
6	105.0700	13494	93.0	45	46.5	0.0078
7	107.0850	13753	122.0	59	61.1	0.0078
8	119.0855	15294	64.4	31	32.2	0.0078
9	161.0963	20690	60.8	29	30.4	0.0078
10	191.1429	24549	199.8	96	100.0	0.0078

Cmpd 1037, AutoMSn(309.2050), 12.39 min

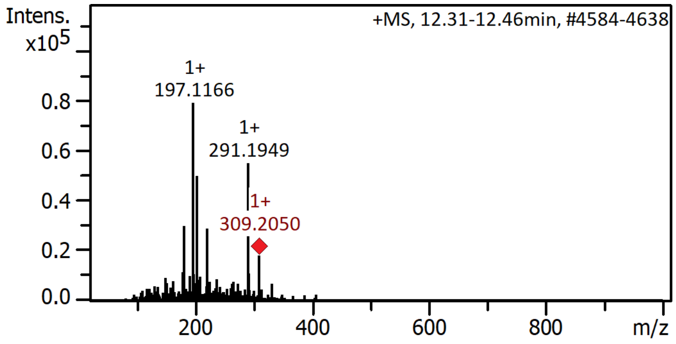

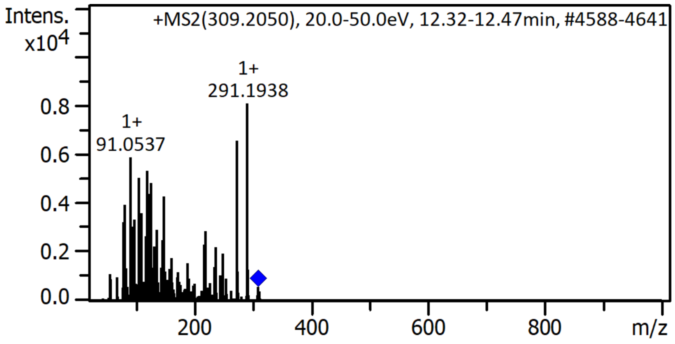

#	m/z	Res.	S/N	I	I %	FWHM
1	179.1059	10630	1578.2	11363	14.4	0.0168
2	181.1214	11188	4150.8	29886	37.8	0.0162
3	191.1425	11650	1366.6	9840	12.5	0.0164
4	197.1166	11087	10969.5	78980	100.0	0.0178
5	198.1194	11026	1480.6	10660	13.5	0.0180
6	203.1787	11316	6940.3	49970	63.3	0.0180
7	221.1889	11513	4036.8	29065	36.8	0.0192
8	291.1949	12707	7639.3	55003	69.6	0.0229
9	292.1982	11914	1504.1	10829	13.7	0.0245
10	309.2050	12280	2534.5	18248	23.1	0.0252

#	m/z	Res.	S/N	I	I %	FWHM
1	81.0694	8977	410.4	3940	48.8	0.0090
2	91.0537	8753	611.7	5872	72.8	0.0104
3	105.0699	10074	526.5	5054	62.6	0.0104
4	109.0634	10326	374.6	3597	44.6	0.0106
5	119.0848	10020	554.5	5323	66.0	0.0119
6	123.0801	10150	457.0	4388	54.4	0.0121
7	125.0950	10255	502.9	4828	59.8	0.0122
8	147.0797	10633	445.0	4272	52.9	0.0138
9	273.1847	11563	683.5	6561	81.3	0.0236
10	291.1938	12292	840.4	8068	100.0	0.0237

Compound Spectrum List Report

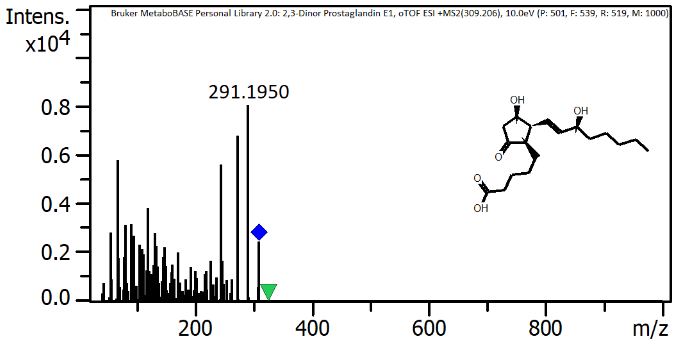

#	m/z	Res.	S/N	I	I %	FWHM
1	55.0548	2015	70.4	2840	35.2	0.0273
2	67.0545	2455	144.0	5809	72.1	0.0273
3	81.0703	2968	78.0	3146	39.0	0.0273
4	91.0541	3333	78.6	3171	39.3	0.0273
5	95.0848	3481	67.2	2711	33.6	0.0273
6	119.0845	4359	95.0	3832	47.5	0.0273
7	131.0850	4799	70.0	2824	35.0	0.0273
8	245.1892	8976	139.2	5615	69.7	0.0273
9	273.1840	10000	168.6	6801	84.4	0.0273
10	291.1950	10660	199.8	8060	100.0	0.0273

Cmpd 1038, AutoMSn(181.1222), 12.40 min

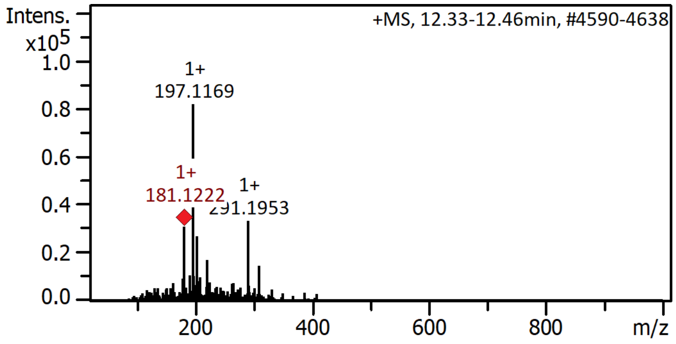

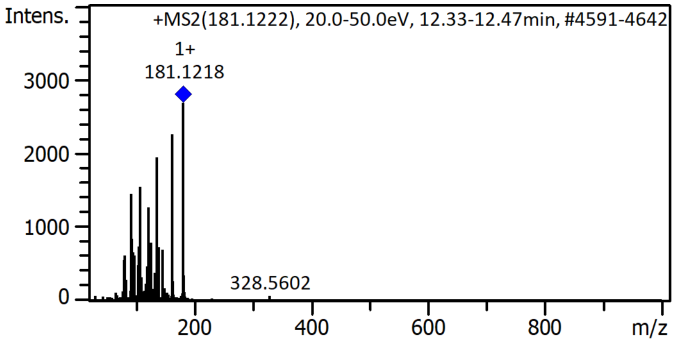

#	m/z	Res.	S/N	I	I %	FWHM
1	179.1056	10792	1020.5	9185	11.2	0.0166
2	181.1222	11056	3446.9	31022	37.9	0.0164
3	191.1433	11302	1179.8	10618	13.0	0.0169
4	197.1169	11344	9099.8	81899	100.0	0.0174
5	198.1208	10811	1144.9	10304	12.6	0.0183
6	203.1793	11195	2988.9	26901	32.8	0.0181
7	209.1526	11344	1078.1	9703	11.8	0.0184
8	221.1894	11398	1893.7	17043	20.8	0.0194
9	291.1953	13144	3714.4	33429	40.8	0.0222
10	309.2057	12089	1625.6	14630	17.9	0.0256

#	m/z	Res.	S/N	I	I %	FWHM
1	91.0533	8941	114.3	1458	54.3	0.0102
2	93.0709	7759	66.2	845	31.5	0.0120
3	105.0706	9897	58.4	745	27.7	0.0106
4	107.0847	11290	121.8	1554	57.9	0.0095
5	121.1004	11429	99.8	1273	47.4	0.0106
6	125.0597	11713	62.1	791	29.5	0.0107
7	135.1168	10863	152.9	1950	72.6	0.0124
8	139.0737	12357	57.3	731	27.2	0.0113
9	163.1117	11271	177.2	2259	84.1	0.0145
10	181.1218	10137	210.6	2685	100.0	0.0179

Compound Spectrum List Report

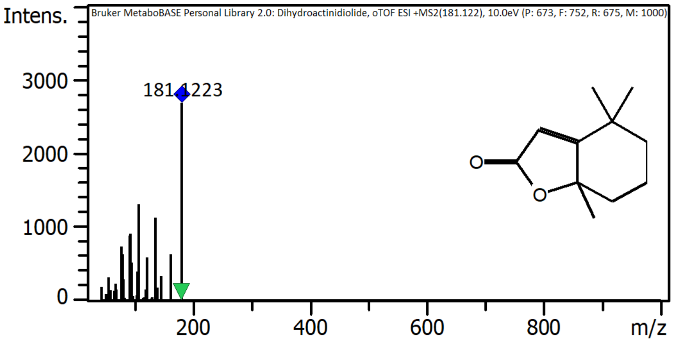

#	m/z	Res.	S/N	I	I %	FWHM
1	77.0390	4691	46.0	741	27.6	0.0164
2	79.0547	4814	39.7	639	23.8	0.0164
3	91.0545	5544	55.2	889	33.1	0.0164
4	93.0701	5667	57.0	918	34.2	0.0164
5	95.0855	5790	32.3	521	19.4	0.0164
6	107.0854	6520	81.8	1318	49.1	0.0164
7	121.1003	7374	36.8	593	22.1	0.0164
8	135.1165	8227	70.5	1136	42.3	0.0164
9	163.1114	9932	39.5	636	23.7	0.0164
10	181.1223	11028	166.5	2682	100.0	0.0164

Cmpd 1040, AutoMSn(203.1792), 12.42 min

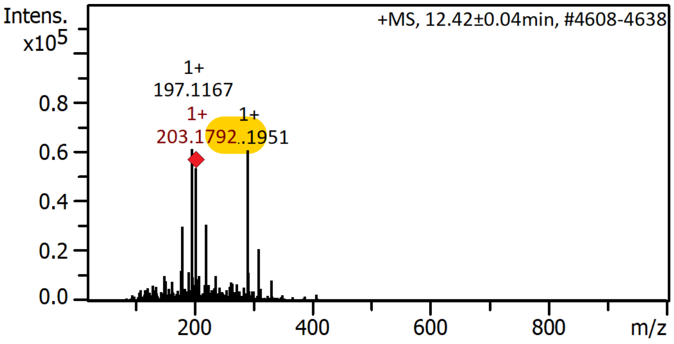

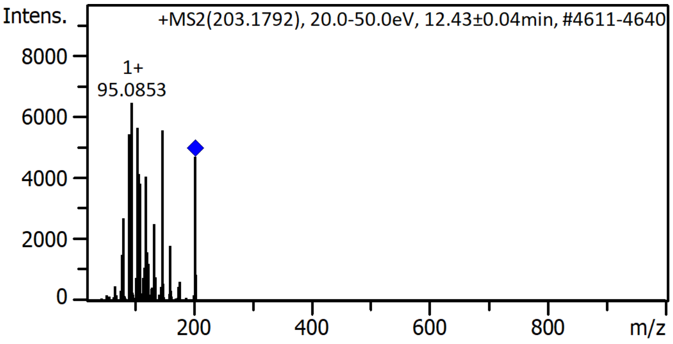

#	m/z	Res.	S/N	I	I %	FWHM
1	179.1060	10989	1675.8	12066	15.2	0.0163
2	181.1215	11212	4172.2	30040	37.7	0.0162
3	191.1427	11627	1576.4	11350	14.3	0.0164
4	197.1167	11331	11056.2	79605	100.0	0.0174
5	203.1792	11519	7406.4	53326	67.0	0.0176
6	221.1894	11874	4270.9	30751	38.6	0.0186
7	237.1096	10613	1390.6	10012	12.6	0.0223
8	291.1951	12909	8424.9	60659	76.2	0.0226
9	292.1994	12496	1556.2	11205	14.1	0.0234
10	309.2056	12309	2931.9	21110	26.5	0.0251

#	m/z	Res.	S/N	I	I %	FWHM
1	81.0689	8503	286.2	2690	41.8	0.0095
2	91.0535	9055	576.5	5419	84.2	0.0101
3	95.0853	10093	684.9	6438	100.0	0.0094
4	105.0694	9859	598.8	5629	87.4	0.0107
5	107.0844	9426	439.9	4135	64.2	0.0114
6	109.1003	10584	407.3	3828	59.5	0.0103
7	119.0850	9342	431.5	4056	63.0	0.0127
8	133.0997	11077	267.7	2517	39.1	0.0120
9	147.1159	10792	589.8	5544	86.1	0.0136
10	203.1784	11200	500.1	4701	73.0	0.0181

Compound Spectrum List Report

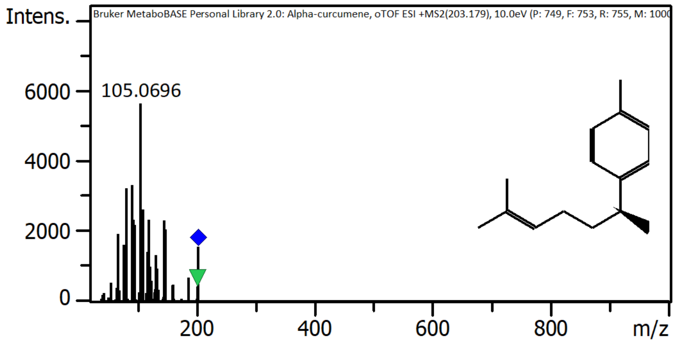

#	m/z	Res.	S/N	I	I %	FWHM
1	81.0697	8656	114.8	3231	57.5	0.0094
2	91.0545	9723	118.0	3321	59.1	0.0094
3	93.0690	9938	82.8	2330	41.4	0.0094
4	95.0851	10153	78.0	2195	39.0	0.0094
5	105.0696	11219	199.8	5623	100.0	0.0094
6	107.0844	11434	93.4	2629	46.7	0.0094
7	109.1014	11650	93.4	2629	46.7	0.0094
8	119.0845	12716	82.8	2330	41.4	0.0094
9	145.0992	15493	82.2	2314	41.1	0.0094
10	147.1150	15709	73.2	2060	36.6	0.0094

Cmpd 1045, AutoMSn(191.1420), 12.51 min

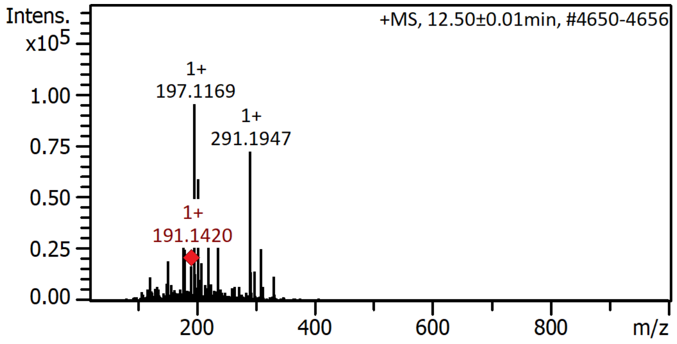

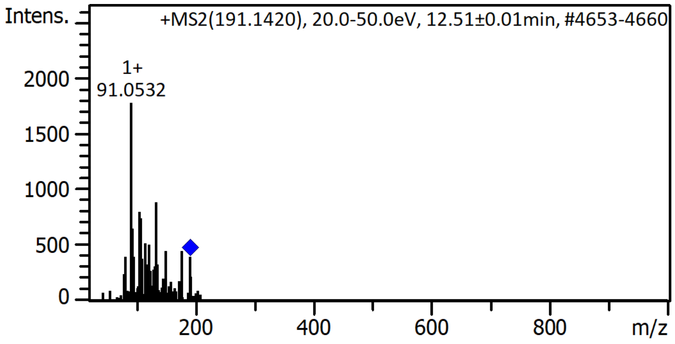

#	m/z	Res.	S/N	I	I %	FWHM
1	151.1115	9916	1070.4	19268	20.2	0.0152
2	179.1062	10992	1463.9	26350	27.7	0.0163
3	181.1220	11081	1389.8	25017	26.3	0.0163
4	197.1169	11131	5294.2	95296	100.0	0.0177
5	203.1785	11805	3276.6	58979	61.9	0.0172
6	209.1522	12671	1023.9	18430	19.3	0.0165
7	221.1895	11277	2065.4	37178	39.0	0.0196
8	237.1089	11987	1632.8	29390	30.8	0.0198
9	291.1947	12036	4009.3	72167	75.7	0.0242
10	309.2059	12629	1406.1	25309	26.6	0.0245

#	m/z	Res.	S/N	I	I %	FWHM
1	81.0685	8941	16.3	400	22.5	0.0091
2	91.0532	10316	72.3	1773	100.0	0.0088
3	93.0663	8722	26.6	652	36.8	0.0107
4	105.0707	12134	32.7	801	45.2	0.0087
5	107.0853	10463	30.2	741	41.8	0.0102
6	115.0527	12625	21.1	518	29.2	0.0091
7	121.0987	12559	20.7	507	28.6	0.0096
8	133.0981	9040	36.2	887	50.0	0.0147
9	149.1281	12658	18.4	450	25.4	0.0118
10	178.0202	16539	18.3	449	25.3	0.0108

Compound Spectrum List Report

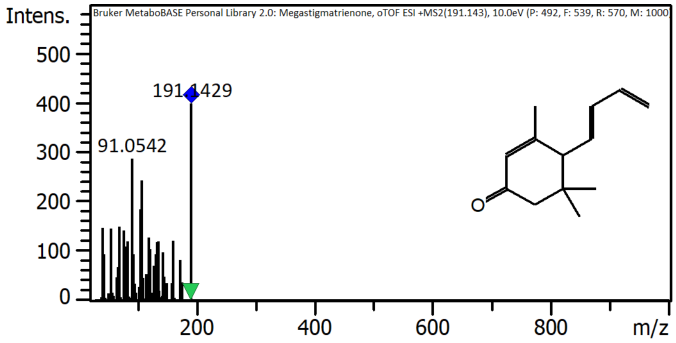

#	m/z	Res.	S/N	I	I %	FWHM
1	41.0394	5855	73.8	147	36.9	0.0070
2	55.0544	7855	73.4	146	36.7	0.0070
3	69.0337	9849	75.4	150	37.7	0.0070
4	77.0389	10991	71.2	142	35.6	0.0070
5	91.0542	12991	144.0	287	72.1	0.0070
6	105.0700	14990	93.0	186	46.5	0.0070
7	107.0850	15278	122.0	243	61.1	0.0070
8	119.0855	16990	64.4	128	32.2	0.0070
9	161.0963	22984	60.8	121	30.4	0.0070
10	191.1429	27270	199.8	399	100.0	0.0070

Cmpd 1047, AutoMSn(299.0909), 12.52 min

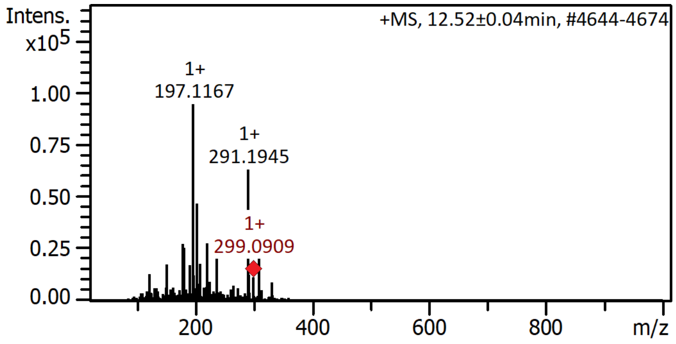

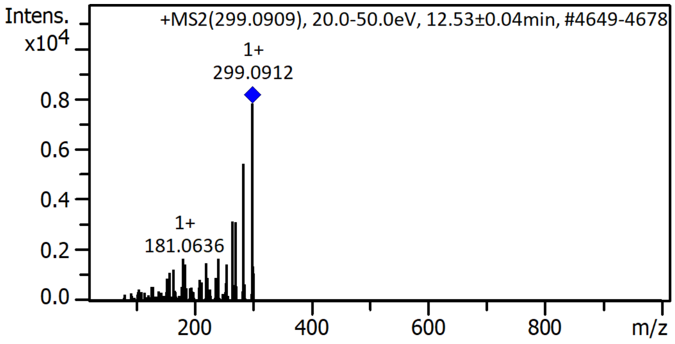

#	m/z	Res.	S/N	I	I %	FWHM
1	151.1106	10837	2413.6	17378	18.4	0.0139
2	179.1059	11387	3826.2	27549	29.1	0.0157
3	181.1214	11420	3570.8	25710	27.2	0.0159
4	197.1167	11235	13138.7	94598	100.0	0.0175
5	203.1786	11540	6512.5	46890	49.6	0.0176
6	209.1528	11222	2440.1	17569	18.6	0.0186
7	221.1892	11566	3851.0	27727	29.3	0.0191
8	237.1086	12126	4165.5	29992	31.7	0.0196
9	291.1945	12052	8772.6	63163	66.8	0.0242
10	309.2048	12400	3086.7	22224	23.5	0.0249

#	m/z	Res.	S/N	I	I %	FWHM
1	181.0636	12562	176.1	1691	21.6	0.0144
2	185.0592	11077	149.9	1439	18.4	0.0167
3	221.0604	12235	155.1	1489	19.0	0.0181
4	241.0475	10186	175.6	1685	21.5	0.0237
5	256.0716	14258	147.8	1419	18.1	0.0180
6	266.0551	13660	328.3	3152	40.3	0.0195
7	271.0972	12071	324.8	3118	39.9	0.0225
8	284.0666	12705	564.6	5421	69.3	0.0224
9	299.0912	10949	814.9	7823	100.0	0.0273
10	300.0926	12338	142.3	1366	17.5	0.0243

Compound Spectrum List Report

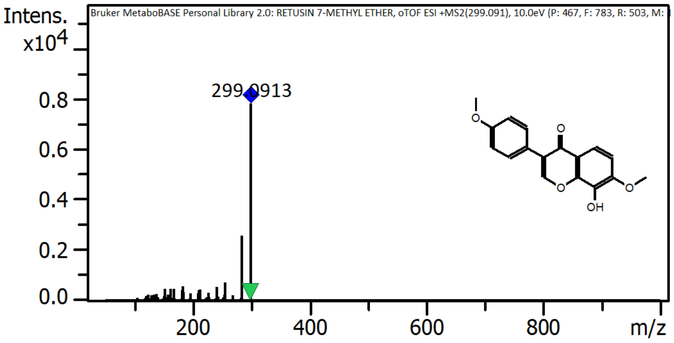

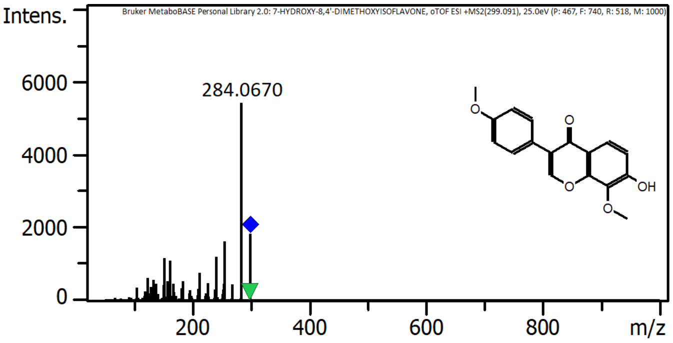

#	m/z	Res.	S/N	I	I %	FWHM
1	152.0088	6162	12.0	469	6.0	0.0247
2	161.0586	6528	11.8	462	5.9	0.0247
3	168.0553	6812	12.2	477	6.1	0.0247
4	183.0793	7421	14.4	563	7.2	0.0247
5	213.0530	8636	11.6	454	5.8	0.0247
6	241.0493	9771	14.0	548	7.0	0.0247
7	256.0728	10380	18.6	728	9.3	0.0247
8	284.0672	11515	66.6	2605	33.3	0.0247
9	299.0913	12124	199.8	7815	100.0	0.0247
10	299.0950	12124	83.2	3254	41.6	0.0247

#	m/z	Res.	S/N	I	I %	FWHM
1	123.0073	4986	23.0	623	11.5	0.0247
2	133.0637	5394	20.8	564	10.4	0.0247
3	152.0095	6162	43.6	1182	21.8	0.0247
4	161.0589	6528	41.2	1117	20.6	0.0247
5	213.0534	8636	28.2	764	14.1	0.0247
6	241.0472	9771	44.8	1214	22.4	0.0247
7	256.0722	10380	60.6	1642	30.3	0.0247
8	284.0670	11515	199.8	5415	100.0	0.0247
9	299.0875	12123	41.0	1111	20.5	0.0247
10	299.0901	12124	68.4	1854	34.2	0.0247

Cmpd 1076, AutoMSn(181.1211), 12.80 min

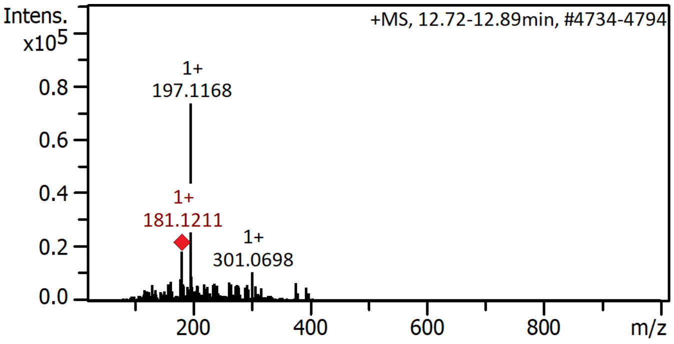

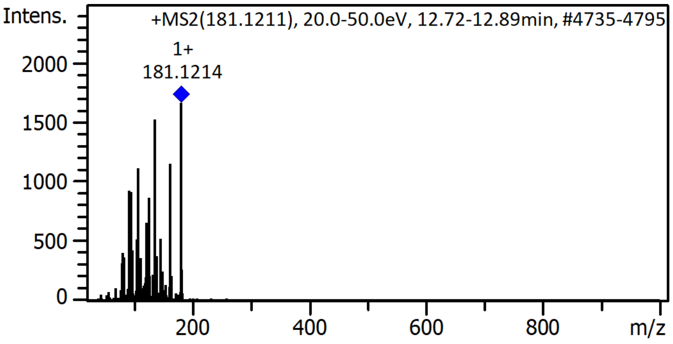

#	m/z	Res.	S/N	I	I %	FWHM
1	157.0832	10020	1183.8	6088	8.3	0.0157
2	163.0587	11054	1351.6	6951	9.5	0.0148
3	179.1060	11710	1530.6	7871	10.7	0.0153
4	181.1211	11263	3566.5	18342	25.0	0.0161
5	197.1168	11196	14282.1	73451	100.0	0.0176
6	198.1203	11118	1731.3	8904	12.1	0.0178
7	237.1089	11431	1214.3	6245	8.5	0.0207
8	262.1427	11456	1311.7	6746	9.2	0.0229
9	301.0698	12325	2061.8	10604	14.4	0.0244
10	376.2583	12669	1273.7	6550	8.9	0.0297

#	m/z	Res.	S/N	I	I %	FWHM
1	91.0545	8825	132.3	926	55.8	0.0103
2	93.0689	9307	95.1	665	40.1	0.0100
3	95.0851	10071	131.1	918	55.3	0.0094
4	107.0843	9707	159.1	1113	67.1	0.0110
5	121.1008	9544	94.2	660	39.8	0.0127
6	125.0587	11353	124.0	868	52.3	0.0110
7	135.1164	10027	217.4	1522	91.7	0.0135
8	145.0991	9980	74.6	522	31.5	0.0145
9	163.1102	12366	164.8	1154	69.5	0.0132
10	181.1214	11326	237.1	1659	100.0	0.0160

Compound Spectrum List Report

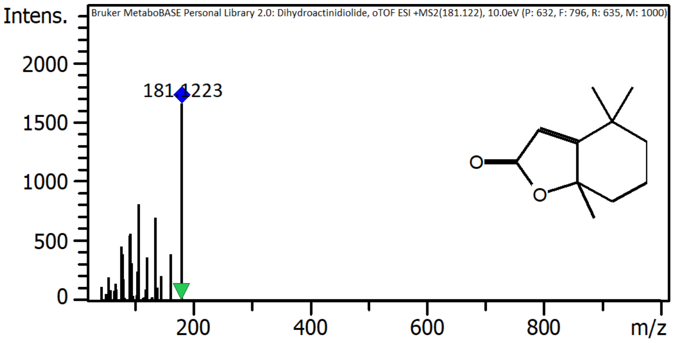

#	m/z	Res.	S/N	I	I %	FWHM
1	77.0390	4753	46.0	458	27.6	0.0162
2	79.0547	4878	39.7	395	23.8	0.0162
3	91.0545	5618	55.2	549	33.1	0.0162
4	93.0701	5742	57.0	568	34.2	0.0162
5	95.0855	5867	32.3	322	19.4	0.0162
6	107.0854	6607	81.8	815	49.1	0.0162
7	121.1003	7472	36.8	367	22.1	0.0162
8	135.1165	8337	70.5	702	42.3	0.0162
9	163.1114	10064	39.5	393	23.7	0.0162
10	181.1223	11175	166.5	1658	100.0	0.0162

Cmpd 1078, AutoMSn(317.0659), 12.82 min

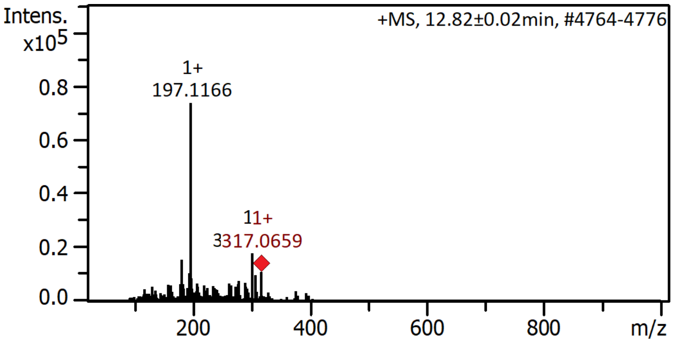

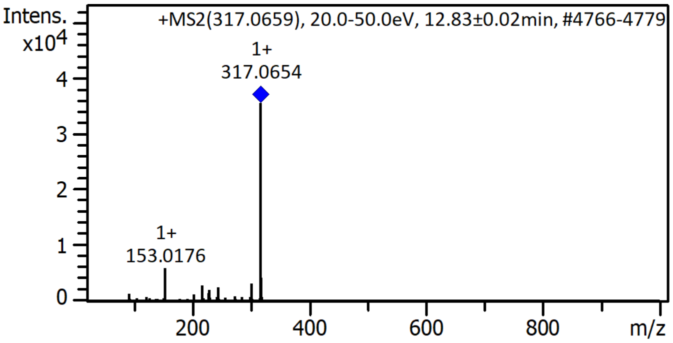

#	m/z	Res.	S/N	I	I %	FWHM
1	181.1217	10659	1303.2	15638	21.2	0.0170
2	194.1168	11946	860.7	10328	14.0	0.0162
3	197.1166	11141	6138.3	73659	100.0	0.0177
4	198.1199	11567	709.4	8513	11.6	0.0171
5	262.1435	12015	543.4	6521	8.9	0.0218
6	279.0760	12156	620.2	7442	10.1	0.0230
7	289.1783	12438	575.1	6901	9.4	0.0233
8	301.0704	12862	1491.8	17901	24.3	0.0234
9	307.1904	11699	813.1	9757	13.2	0.0263
10	317.0659	11872	905.8	10869	14.8	0.0267

#	m/z	Res.	S/N	I	I %	FWHM
1	92.0256	8767	82.1	1341	3.8	0.0105
2	153.0176	11825	360.8	5892	16.6	0.0129
3	203.0331	14082	77.0	1258	3.5	0.0144
4	217.0487	11670	173.0	2825	8.0	0.0186
5	228.0411	12690	87.8	1434	4.0	0.0180
6	229.0509	12353	123.8	2023	5.7	0.0185
7	245.0446	10611	154.2	2518	7.1	0.0231
8	302.0430	11109	192.2	3140	8.9	0.0272
9	317.0654	13031	2170.8	35456	100.0	0.0243
10	318.0670	14449	257.9	4212	11.9	0.0220

Compound Spectrum List Report

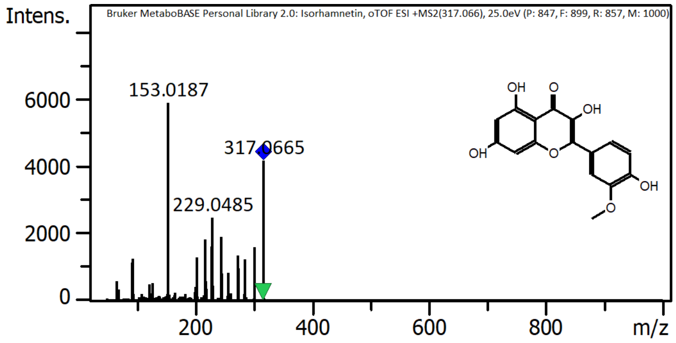

#	m/z	Res.	S/N	I	I %	FWHM
1	93.0348	3710	42.8	1261	21.4	0.0251
2	153.0187	6101	199.8	5886	100.0	0.0251
3	203.0337	8095	44.0	1296	22.0	0.0251
4	217.0493	8654	62.6	1844	31.3	0.0251
5	228.0398	9092	55.4	1632	27.7	0.0251
6	229.0485	9133	84.4	2487	42.2	0.0251
7	245.0434	9770	65.2	1921	32.6	0.0251
8	273.0431	10887	46.4	1367	23.2	0.0251
9	302.0443	12043	54.8	1614	27.4	0.0251
10	317.0665	12642	141.8	4178	71.0	0.0251

Cmpd 1080, AutoMSn(293.2097), 12.84 min

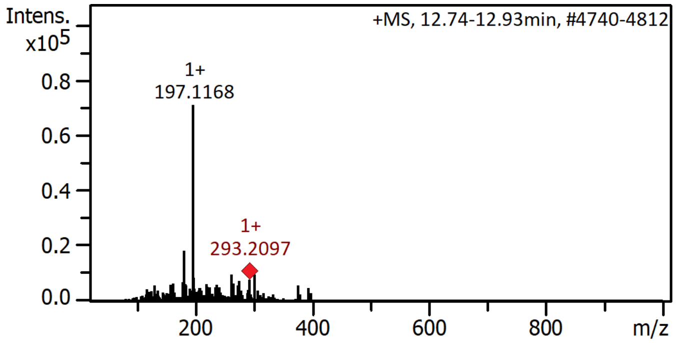

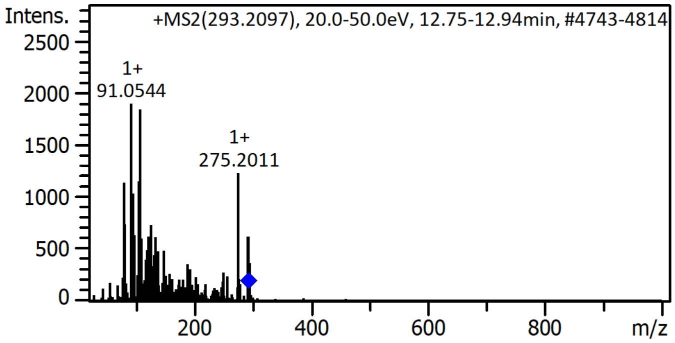

#	m/z	Res.	S/N	I	I %	FWHM
1	163.0581	10683	888.8	6399	9.0	0.0153
2	179.1061	11689	944.5	6801	9.6	0.0153
3	181.1214	11231	2558.1	18418	25.9	0.0161
4	197.1168	11340	9864.8	71026	100.0	0.0174
5	198.1205	11379	1169.3	8419	11.9	0.0174
6	262.1429	12419	1324.0	9533	13.4	0.0211
7	265.1409	11885	873.5	6289	8.9	0.0223
8	275.1990	11433	1011.8	7285	10.3	0.0241
9	293.2097	12761	1073.4	7728	10.9	0.0230
10	301.0697	12477	1319.9	9504	13.4	0.0241

#	m/z	Res.	S/N	I	I %	FWHM
1	79.0542	8609	116.5	1142	60.1	0.0092
2	81.0702	7428	75.4	739	38.9	0.0109
3	91.0544	10721	193.7	1898	100.0	0.0085
4	93.0698	8743	86.6	848	44.7	0.0106
5	95.0849	9530	105.8	1037	54.6	0.0100
6	97.1012	10725	65.0	637	33.5	0.0091
7	105.0689	10129	117.4	1151	60.6	0.0104
8	107.0844	10188	188.0	1843	97.1	0.0105
9	125.0962	12506	74.9	734	38.7	0.0100
10	275.2011	13805	125.9	1234	65.0	0.0199

Compound Spectrum List Report

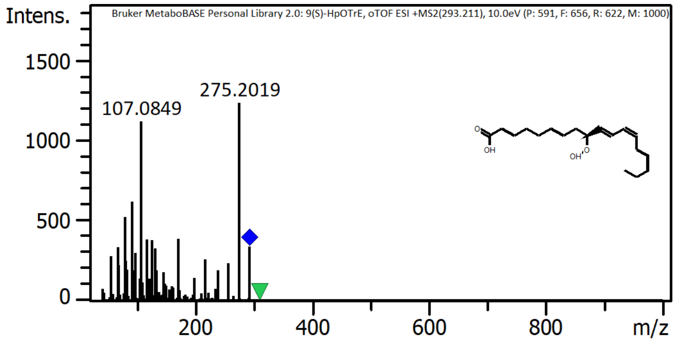

#	m/z	Res.	S/N	I	I %	FWHM
1	67.0547	9163	54.4	336	27.2	0.0073
2	79.0548	10803	85.0	524	42.5	0.0073
3	91.0538	12442	100.6	621	50.4	0.0073
4	107.0849	14633	181.2	1118	90.7	0.0073
5	117.0699	15997	62.2	384	31.1	0.0073
6	125.0948	17094	61.4	379	30.7	0.0073
7	131.0845	17913	53.2	328	26.6	0.0073
8	171.1027	23381	62.8	387	31.4	0.0073
9	275.2019	37606	199.8	1233	100.0	0.0073
10	293.2087	40067	55.2	341	27.6	0.0073

Cmpd 1101, AutoMSn(293.2089), 13.08 min

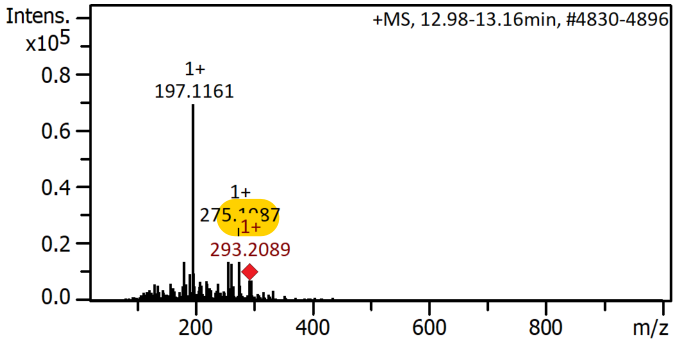

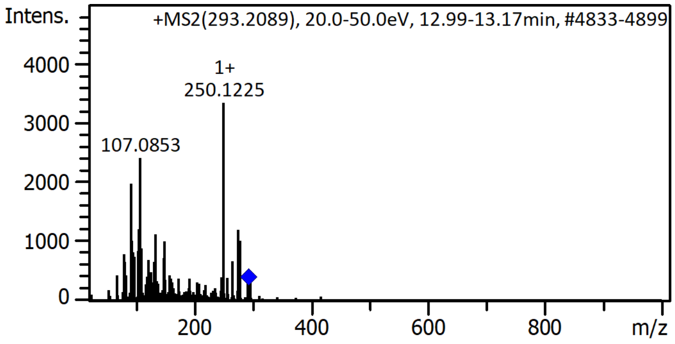

#	m/z	Res.	S/N	I	I %	FWHM
1	181.1213	11844	770.3	13866	20.0	0.0153
2	191.1425	12194	515.4	9278	13.4	0.0157
3	197.1161	11767	3845.0	69210	100.0	0.0168
4	198.1196	10357	531.0	9558	13.8	0.0191
5	219.0973	11617	375.3	6755	9.8	0.0189
6	257.1872	11914	1053.3	18960	27.4	0.0216
7	262.1416	13085	721.6	12988	18.8	0.0200
8	275.1987	11818	1427.7	25698	37.1	0.0233
9	293.2089	12527	398.4	7172	10.4	0.0234
10	296.1277	14214	388.8	6998	10.1	0.0208

#	m/z	Res.	S/N	I	I %	FWHM
1	91.0544	6509	79.2	1979	59.3	0.0140
2	93.0684	11224	40.6	1015	30.4	0.0083
3	105.0677	10324	48.4	1209	36.2	0.0102
4	107.0853	7393	96.4	2410	72.2	0.0145
5	109.0989	8992	35.7	893	26.8	0.0121
6	133.1003	9624	45.0	1126	33.8	0.0138
7	148.0769	12026	40.3	1008	30.2	0.0123
8	250.1225	14139	133.4	3336	100.0	0.0177
9	275.1996	15803	47.9	1199	35.9	0.0174
10	278.1198	15655	40.6	1014	30.4	0.0178

Compound Spectrum List Report

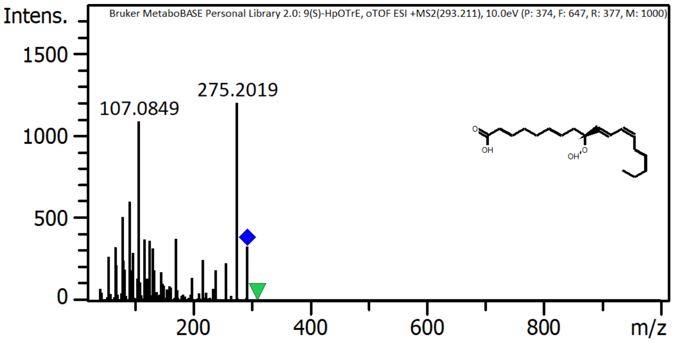

#	m/z	Res.	S/N	I	I %	FWHM
1	67.0547	3236	54.4	326	27.2	0.0207
2	79.0548	3815	85.0	509	42.5	0.0207
3	91.0538	4394	100.6	603	50.4	0.0207
4	107.0849	5168	181.2	1086	90.7	0.0207
5	117.0699	5650	62.2	373	31.1	0.0207
6	125.0948	6037	61.4	368	30.7	0.0207
7	131.0845	6326	53.2	319	26.6	0.0207
8	171.1027	8258	62.8	376	31.4	0.0207
9	275.2019	13282	199.8	1197	100.0	0.0207
10	293.2087	14151	55.2	331	27.6	0.0207

Cmpd 1107, AutoMSn(191.1418), 13.15 min

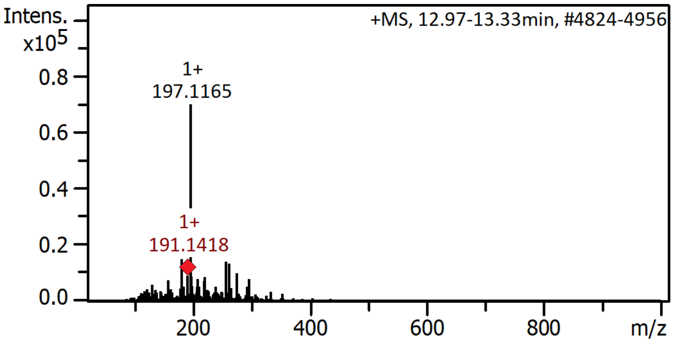

#	m/z	Res.	S/N	I	I %	FWHM
1	181.1220	11178	2503.1	15019	21.5	0.0162
2	191.1418	12214	1491.8	8951	12.8	0.0156
3	197.1165	11476	11644.9	69870	100.0	0.0172
4	198.1200	11288	1473.7	8842	12.7	0.0176
5	209.1527	11082	1290.5	7743	11.1	0.0189
6	221.1525	12187	1415.2	8491	12.2	0.0181
7	257.1880	11459	2383.2	14299	20.5	0.0224
8	262.1431	12181	2240.6	13443	19.2	0.0215
9	275.1995	11689	1644.2	9865	14.1	0.0235
10	296.1271	12903	1293.6	7762	11.1	0.0229

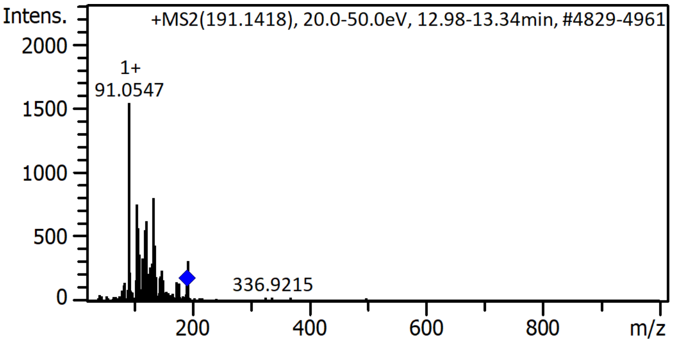

#	m/z	Res.	S/N	I	I %	FWHM
1	91.0547	8245	188.6	1540	100.0	0.0110
2	105.0688	9813	92.5	756	49.1	0.0107
3	107.0848	8878	70.1	573	37.2	0.0121
4	109.1012	11862	45.1	368	23.9	0.0092
5	115.0525	9920	40.9	334	21.7	0.0116
6	119.0839	12102	68.1	556	36.1	0.0098
7	121.1018	8522	76.7	626	40.7	0.0142
8	133.1015	10483	98.7	806	52.3	0.0127
9	135.0780	10449	53.6	437	28.4	0.0129
10	193.0502	14352	39.0	318	20.7	0.0135

Compound Spectrum List Report

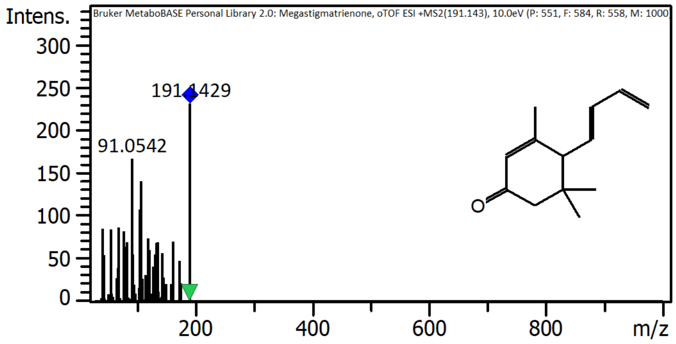

#	m/z	Res.	S/N	I	I %	FWHM
1	41.0394	5092	73.8	85	36.9	0.0081
2	55.0544	6830	73.4	85	36.7	0.0081
3	69.0337	8565	75.4	87	37.7	0.0081
4	77.0389	9558	71.2	82	35.6	0.0081
5	91.0542	11297	144.0	167	72.1	0.0081
6	105.0700	13035	93.0	108	46.5	0.0081
7	107.0850	13285	122.0	141	61.1	0.0081
8	119.0855	14774	64.4	74	32.2	0.0081
9	161.0963	19986	60.8	70	30.4	0.0081
10	191.1429	23714	199.8	231	100.0	0.0081

Cmpd 1109, AutoMSn(293.2099), 13.22 min

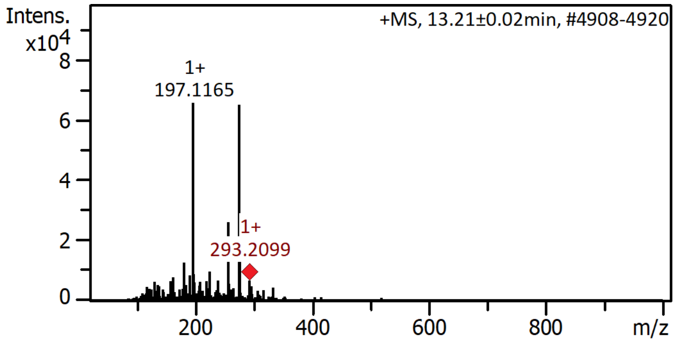

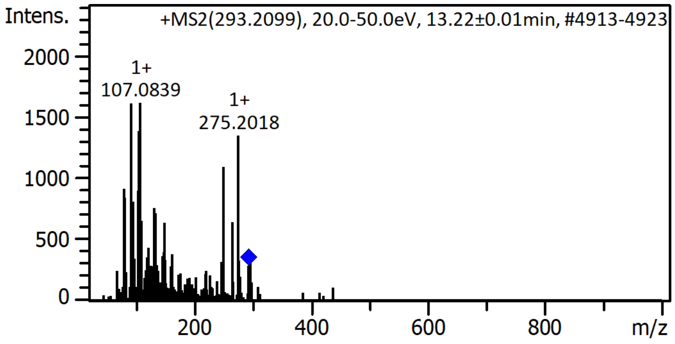

#	m/z	Res.	S/N	I	I %	FWHM
1	163.0549	9334	648.6	7783	11.9	0.0175
2	181.1216	11319	1065.9	12791	19.5	0.0160
3	191.1420	11198	705.2	8462	12.9	0.0171
4	197.1165	10985	5468.8	65625	100.0	0.0179
5	198.1205	11767	730.1	8761	13.4	0.0168
6	225.1014	11287	807.4	9689	14.8	0.0199
7	239.1707	6202	559.8	6717	10.2	0.0386
8	257.1892	12641	2180.7	26168	39.9	0.0203
9	275.1994	12425	5425.8	65109	99.2	0.0221
10	276.2020	12108	1102.4	13229	20.2	0.0228

#	m/z	Res.	S/N	I	I %	FWHM
1	79.0546	9718	56.0	914	56.7	0.0081
2	81.0680	7358	51.7	845	52.4	0.0110
3	91.0539	10548	98.5	1609	99.8	0.0086
4	93.0700	10861	48.2	787	48.8	0.0086
5	95.0858	8943	49.8	813	50.4	0.0106
6	103.0547	11740	55.1	900	55.8	0.0088
7	105.0698	10096	84.8	1385	85.9	0.0104
8	107.0839	8902	98.7	1612	100.0	0.0120
9	250.1202	12862	66.9	1093	67.8	0.0194
10	275.2018	12651	82.4	1346	83.5	0.0218

Compound Spectrum List Report

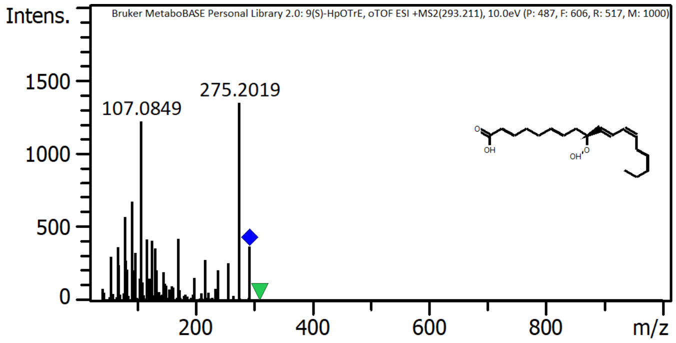

#	m/z	Res.	S/N	I	I %	FWHM
1	67.0547	8244	54.4	366	27.2	0.0081
2	79.0548	9719	85.0	572	42.5	0.0081
3	91.0538	11194	100.6	677	50.4	0.0081
4	107.0849	13165	181.2	1219	90.7	0.0081
5	117.0699	14392	62.2	419	31.1	0.0081
6	125.0948	15379	61.4	413	30.7	0.0081
7	131.0845	16115	53.2	358	26.6	0.0081
8	171.1027	21035	62.8	423	31.4	0.0081
9	275.2019	33833	199.8	1345	100.0	0.0081
10	293.2087	36046	55.2	371	27.6	0.0081

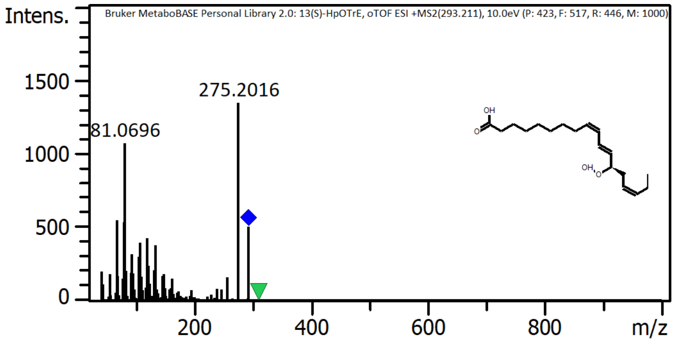

#	m/z	Res.	S/N	I	I %	FWHM
1	67.0547	8244	74.2	499	37.1	0.0081
2	67.0547	8244	81.8	551	40.9	0.0081
3	79.0542	9719	80.0	538	40.0	0.0081
4	81.0696	9967	159.4	1073	79.8	0.0081
5	93.0695	11442	47.6	320	23.8	0.0081
6	107.0848	13165	59.4	400	29.7	0.0081
7	119.0851	14640	63.4	427	31.7	0.0081
8	133.0999	16363	56.8	382	28.4	0.0081
9	275.2016	33833	199.8	1345	100.0	0.0081
10	293.2108	36047	75.6	509	37.8	0.0081

Cmpd 1114, AutoMSn(181.1216), 13.27 min

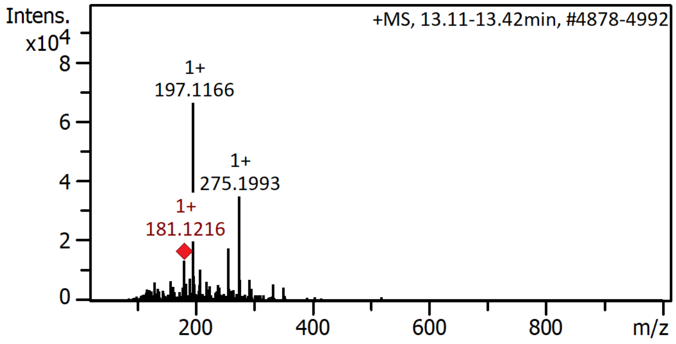

#	m/z	Res.	S/N	I	I %	FWHM
1	157.0831	10510	3296.0	6592	10.0	0.0149
2	181.1216	11863	6789.0	13578	20.5	0.0153
3	191.1420	11250	3685.6	7371	11.1	0.0170
4	197.1166	11401	33098.6	66197	100.0	0.0173
5	198.1199	11406	4131.4	8263	12.5	0.0174
6	209.1527	11561	5179.9	10360	15.6	0.0181
7	257.1889	12152	8821.5	17643	26.7	0.0212
8	275.1993	12081	17499.3	34999	52.9	0.0228
9	276.2025	12564	3498.5	6997	10.6	0.0220
10	293.2098	12712	3462.3	6925	10.5	0.0231

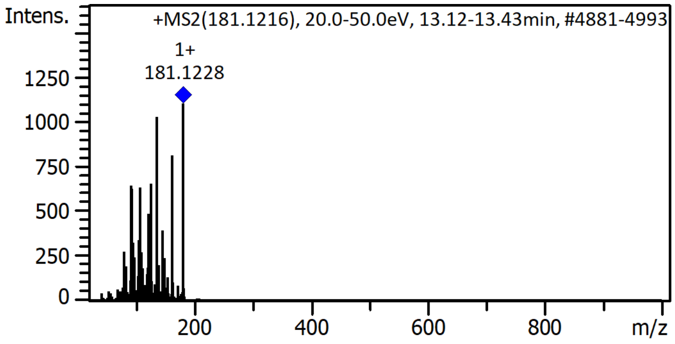

#	m/z	Res.	S/N	I	I %	FWHM
1	91.0539	8680	227.1	644	58.3	0.0105
2	93.0692	8555	221.6	628	56.9	0.0109
3	105.0690	11317	120.2	341	30.9	0.0093
4	107.0845	10272	224.2	635	57.6	0.0104
5	121.1007	10504	171.5	486	44.0	0.0115
6	125.0590	12327	230.9	654	59.3	0.0101
7	135.1163	10919	362.2	1026	93.0	0.0124
8	145.0995	10715	139.0	394	35.7	0.0135
9	163.1109	11022	287.1	813	73.7	0.0148
10	181.1228	11797	389.4	1103	100.0	0.0154

Compound Spectrum List Report

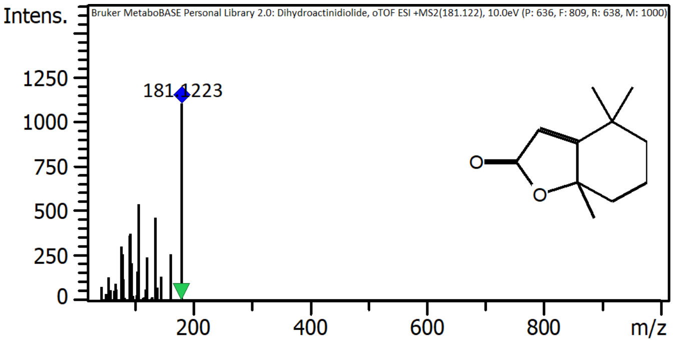

#	m/z	Res.	S/N	I	I %	FWHM
1	77.0390	4789	46.0	305	27.6	0.0161
2	79.0547	4914	39.7	263	23.8	0.0161
3	91.0545	5660	55.2	365	33.1	0.0161
4	93.0701	5785	57.0	377	34.2	0.0161
5	95.0855	5911	32.3	214	19.4	0.0161
6	107.0854	6656	81.8	542	49.1	0.0161
7	121.1003	7528	36.8	244	22.1	0.0161
8	135.1165	8399	70.5	467	42.3	0.0161
9	163.1114	10139	39.5	261	23.7	0.0161
10	181.1223	11259	166.5	1102	100.0	0.0161

Cmpd 1126, AutoMSn(285.0741), 13.39 min

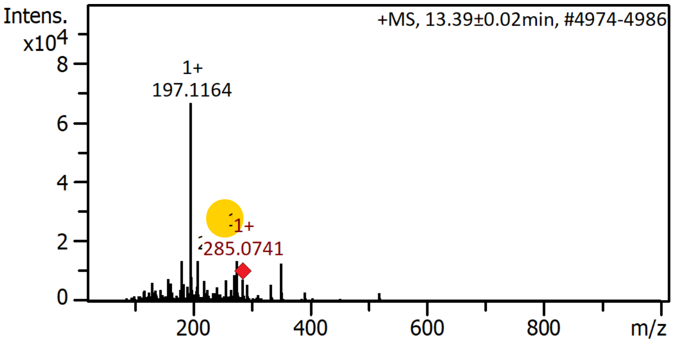

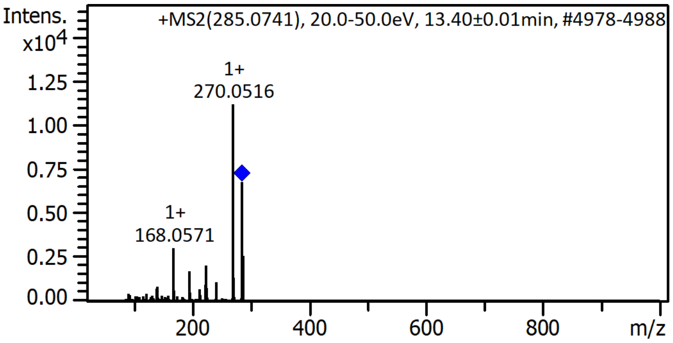

#	m/z	Res.	S/N	I	I %	FWHM
1	157.0836	11958	613.0	7356	11.1	0.0131
2	181.1215	12238	1135.7	13628	20.5	0.0148
3	197.1164	11522	5545.9	66551	100.0	0.0171
4	198.1202	10085	676.6	8119	12.2	0.0196
5	209.1520	11481	1131.5	13578	20.4	0.0182
6	257.1898	10683	575.2	6903	10.4	0.0241
7	271.1440	11320	730.8	8769	13.2	0.0240
8	275.1991	12421	1282.4	15389	23.1	0.0222
9	285.0741	13576	604.1	7250	10.9	0.0210
10	351.2522	12088	1069.9	12839	19.3	0.0291

#	m/z	Res.	S/N	I	I %	FWHM
1	168.0571	8884	186.5	3046	27.2	0.0189
2	196.0525	10111	104.1	1700	15.2	0.0194
3	223.0355	13438	57.3	936	8.4	0.0166
4	224.0466	12655	122.7	2004	17.9	0.0177
5	242.0573	14921	66.5	1086	9.7	0.0162
6	270.0516	11752	685.1	11190	100.0	0.0230
7	271.0554	14771	82.0	1339	12.0	0.0184
8	285.0764	13139	416.2	6798	60.8	0.0217
9	286.0798	9831	59.9	978	8.7	0.0291
10	287.0538	10830	158.1	2583	23.1	0.0265

Compound Spectrum List Report

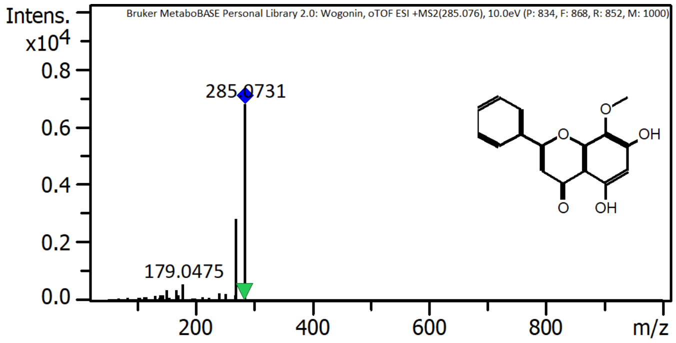

#	m/z	Res.	S/N	I	I %	FWHM
1	151.0528	6621	10.4	354	5.2	0.0228
2	168.0021	7364	8.6	292	4.3	0.0228
3	168.0535	7366	10.4	354	5.2	0.0228
4	179.0475	7848	16.4	557	8.2	0.0228
5	241.0475	10565	7.2	245	3.6	0.0228
6	270.0496	11836	15.4	523	7.7	0.0228
7	270.0499	11836	50.0	1700	25.0	0.0228
8	270.0556	11837	83.2	2828	41.6	0.0228
9	285.0731	12495	199.8	6792	100.0	0.0228
10	285.0788	12495	80.4	2733	40.2	0.0228

Cmpd 1131, AutoMSn(191.1445), 13.50 min

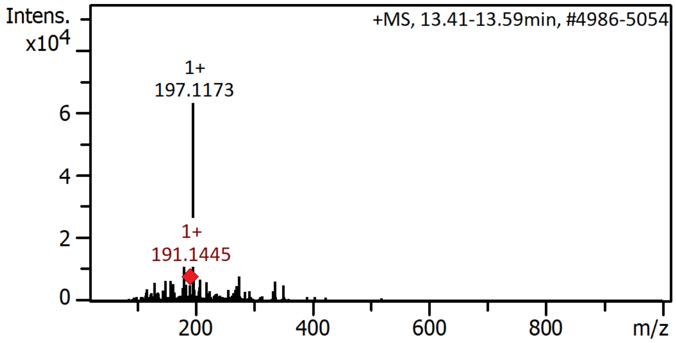

#	m/z	Res.	S/N	I	I %	FWHM
1	130.1592	9505	1282.9	5773	9.2	0.0137
2	149.0594	10141	1439.4	6477	10.3	0.0147
3	157.0831	9575	1431.4	6441	10.2	0.0164
4	181.1226	10125	2709.7	12194	19.3	0.0179
5	197.1173	10104	14020.5	63092	100.0	0.0195
6	198.1206	10227	1832.0	8244	13.1	0.0194
7	209.1537	10154	1523.5	6856	10.9	0.0206
8	219.0988	10391	1315.9	5922	9.4	0.0211
9	275.1995	11901	1745.4	7854	12.4	0.0231
10	337.1046	11291	1360.5	6122	9.7	0.0299

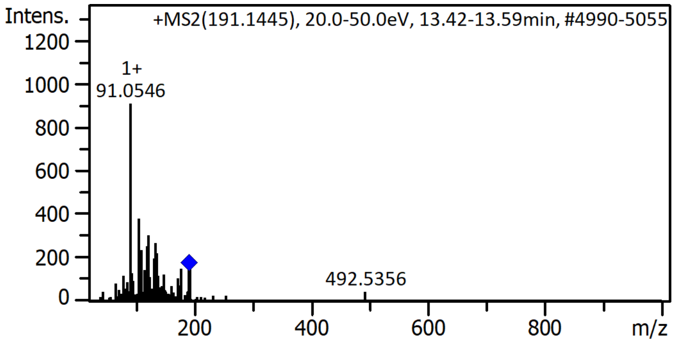

#	m/z	Res.	S/N	I	I %	FWHM
1	91.0546	9157	148.3	908	100.0	0.0099
2	105.0698	8703	62.2	381	42.0	0.0121
3	109.0628	11391	38.5	236	26.0	0.0096
4	109.1021	10222	32.5	199	21.9	0.0107
5	119.0868	11203	41.3	253	27.9	0.0106
6	121.1038	10411	49.5	303	33.4	0.0116
7	131.0851	12892	32.2	198	21.7	0.0102
8	133.0996	11217	44.0	269	29.6	0.0119
9	135.0807	9464	36.3	223	24.5	0.0143
10	193.0508	10831	32.0	196	21.6	0.0178

Compound Spectrum List Report

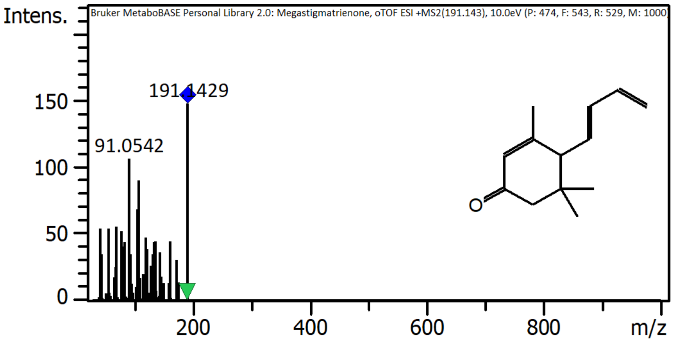

#	m/z	Res.	S/N	I	I %	FWHM
1	41.0394	5554	73.8	55	36.9	0.0074
2	55.0544	7450	73.4	54	36.7	0.0074
3	69.0337	9342	75.4	56	37.7	0.0074
4	77.0389	10425	71.2	53	35.6	0.0074
5	91.0542	12322	144.0	106	72.1	0.0074
6	105.0700	14218	93.0	69	46.5	0.0074
7	107.0850	14491	122.0	90	61.1	0.0074
8	119.0855	16115	64.4	48	32.2	0.0074
9	161.0963	21800	60.8	45	30.4	0.0074
10	191.1429	25866	199.8	148	100.0	0.0074

Cmpd 1135, AutoMSn(181.1225), 13.54 min

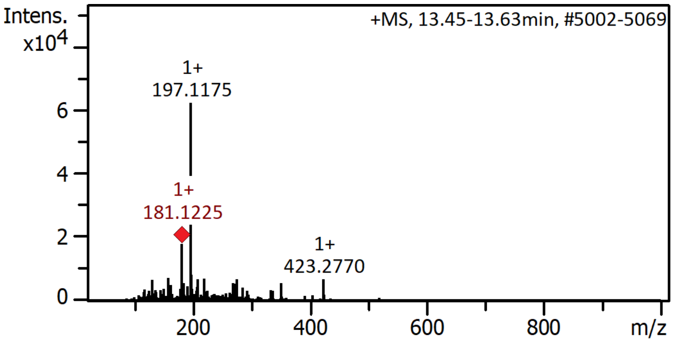

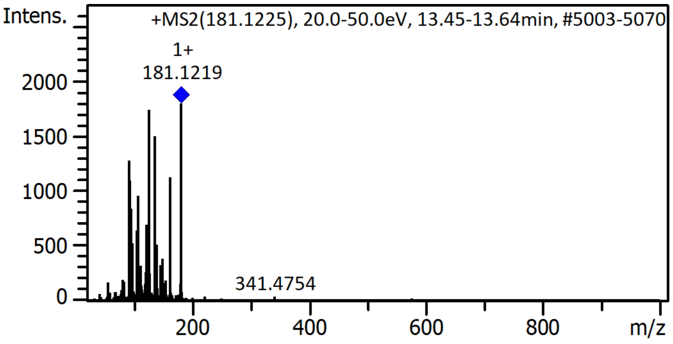

#	m/z	Res.	S/N	I	I %	FWHM
1	130.1595	9632	903.7	6506	10.5	0.0135
2	157.0836	9729	984.3	7087	11.4	0.0161
3	181.1225	9723	2505.0	18036	29.0	0.0186
4	185.0418	10995	774.7	5578	9.0	0.0168
5	197.1175	9598	8639.8	62207	100.0	0.0205
6	198.1191	9991	1143.8	8236	13.2	0.0198
7	209.1530	8725	946.9	6818	11.0	0.0240
8	219.0981	9539	951.8	6853	11.0	0.0230
9	275.1993	10795	941.4	6778	10.9	0.0255
10	423.2770	9721	945.9	6810	10.9	0.0435

#	m/z	Res.	S/N	I	I %	FWHM
1	91.0543	8502	132.9	1276	71.0	0.0107
2	93.0681	8376	114.3	1097	61.1	0.0111
3	95.0834	9780	87.6	841	46.8	0.0097
4	105.0693	10712	67.0	644	35.8	0.0098
5	107.0868	9072	99.6	956	53.2	0.0118
6	121.0988	8596	72.2	693	38.6	0.0141
7	125.0588	10549	181.0	1737	96.7	0.0119
8	135.1166	11334	155.9	1497	83.3	0.0119
9	163.1118	9508	117.2	1126	62.7	0.0172
10	181.1219	11597	187.1	1796	100.0	0.0156

Compound Spectrum List Report

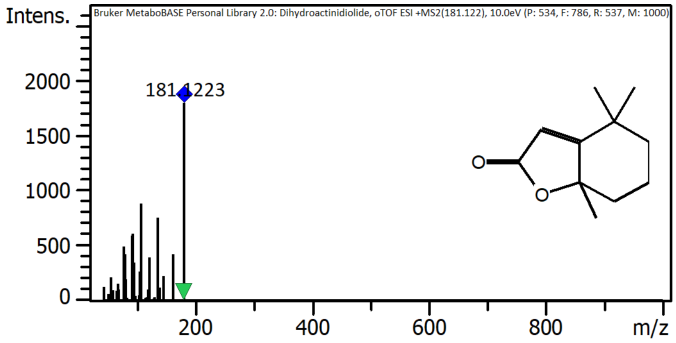

#	m/z	Res.	S/N	I	I %	FWHM
1	77.0390	4476	46.0	496	27.6	0.0172
2	79.0547	4593	39.7	428	23.8	0.0172
3	91.0545	5291	55.2	595	33.1	0.0172
4	93.0701	5408	57.0	614	34.2	0.0172
5	95.0855	5525	32.3	349	19.4	0.0172
6	107.0854	6222	81.8	882	49.1	0.0172
7	121.1003	7037	36.8	397	22.1	0.0172
8	135.1165	7851	70.5	760	42.3	0.0172
9	163.1114	9478	39.5	426	23.7	0.0172
10	181.1223	10524	166.5	1795	100.0	0.0172

Cmpd 1150, AutoMSn(124.0385), 13.76 min

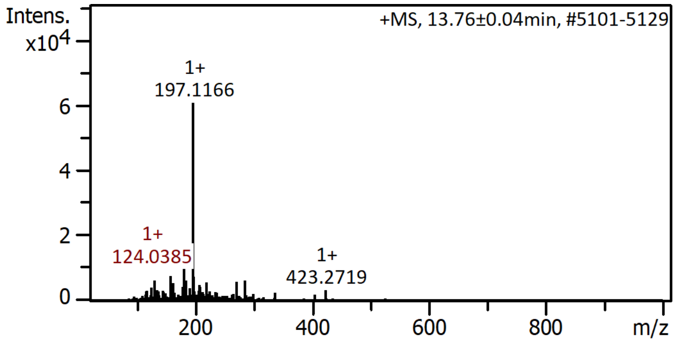

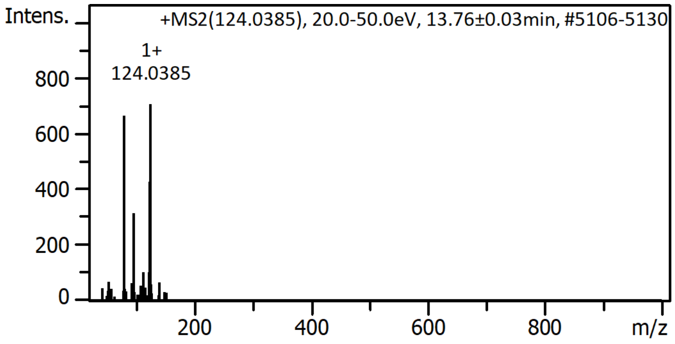

#	m/z	Res.	S/N	I	I %	FWHM
1	130.1586	11266	864.8	6226	10.3	0.0116
2	157.0832	10539	1040.8	7494	12.4	0.0149
3	163.0579	10100	743.6	5354	8.8	0.0161
4	181.1219	10952	1835.2	13214	21.8	0.0165
5	185.0420	10169	860.7	6197	10.2	0.0182
6	197.1166	11227	8421.8	60637	100.0	0.0176
7	198.1195	12184	1030.8	7422	12.2	0.0163
8	219.0987	11727	764.8	5507	9.1	0.0187
9	271.1681	11479	805.3	5798	9.6	0.0236
10	285.0862	11988	851.9	6133	10.1	0.0238

#	m/z	Res.	S/N	I	I %	FWHM
1	53.0360	7180	6.9	69	9.7	0.0074
2	80.0480	8002	66.4	664	94.0	0.0100
3	93.0699	13149	6.3	63	8.9	0.0071
4	96.0463	8570	31.5	315	44.6	0.0112
5	112.0375	10533	10.1	101	14.3	0.0106
6	122.0967	12571	10.1	101	14.3	0.0097
7	123.0546	13640	42.7	427	60.5	0.0090
8	124.0385	11698	70.6	706	100.0	0.0106
9	126.0552	14964	5.8	58	8.2	0.0084
10	140.0443	15024	6.4	64	9.1	0.0093

Compound Spectrum List Report

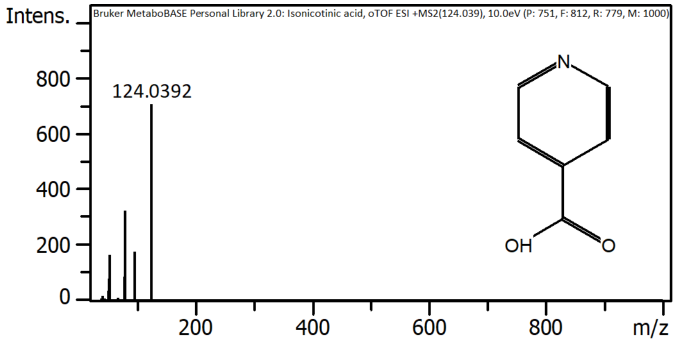

#	m/z	Res.	S/N	I	I %	FWHM
1	41.0392	3788	4.8	17	2.4	0.0108
2	51.0224	4709	10.2	36	5.1	0.0108
3	52.0307	4802	22.4	79	11.2	0.0108
4	53.0390	4895	46.8	165	23.4	0.0108
5	78.0343	7202	13.8	49	6.9	0.0108
6	78.0345	7202	6.8	24	3.4	0.0108
7	79.0421	7295	24.2	85	12.1	0.0108
8	80.0500	7388	92.0	325	46.0	0.0108
9	96.0449	8864	50.6	179	25.3	0.0108
10	124.0392	11448	199.8	705	100.0	0.0108

Cmpd 1152, AutoMSn(181.1213), 13.78 min

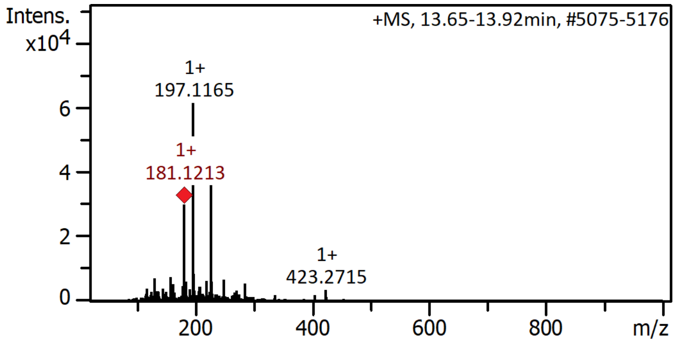

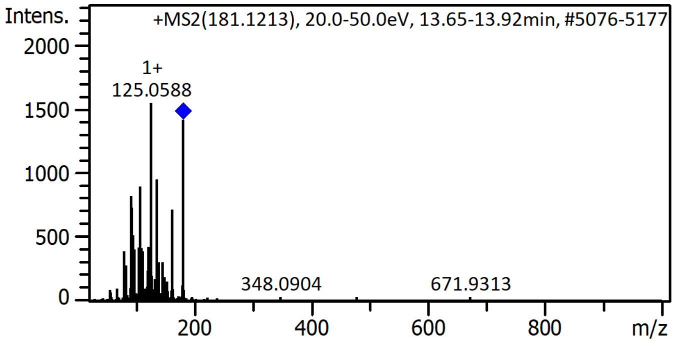

#	m/z	Res.	S/N	I	I %	FWHM
1	130.1581	10835	1381.2	7103	11.6	0.0120
2	157.0828	10763	1450.0	7457	12.1	0.0146
3	181.1213	11171	5857.5	30124	49.1	0.0162
4	185.0412	11230	1170.9	6022	9.8	0.0165
5	197.1165	10975	11934.7	61378	100.0	0.0180
6	198.1201	11426	1646.4	8467	13.8	0.0173
7	219.0976	12654	1233.4	6343	10.3	0.0173
8	227.1629	11815	7733.3	39771	64.8	0.0192
9	228.1665	11950	1161.1	5971	9.7	0.0191
10	249.1458	11948	1304.8	6710	10.9	0.0209

#	m/z	Res.	S/N	I	I %	FWHM
1	91.0536	8821	122.7	824	53.3	0.0103
2	93.0682	11539	108.8	730	47.2	0.0081
3	95.0858	12023	77.3	519	33.5	0.0079
4	105.0678	9046	63.0	423	27.4	0.0116
5	107.0842	10185	133.8	898	58.1	0.0105
6	121.0999	13356	63.9	429	27.7	0.0091
7	125.0588	9811	230.4	1547	100.0	0.0127
8	135.1160	8473	142.2	955	61.7	0.0159
9	163.1107	12513	106.8	717	46.4	0.0130
10	181.1213	10806	211.2	1418	91.7	0.0168

Compound Spectrum List Report

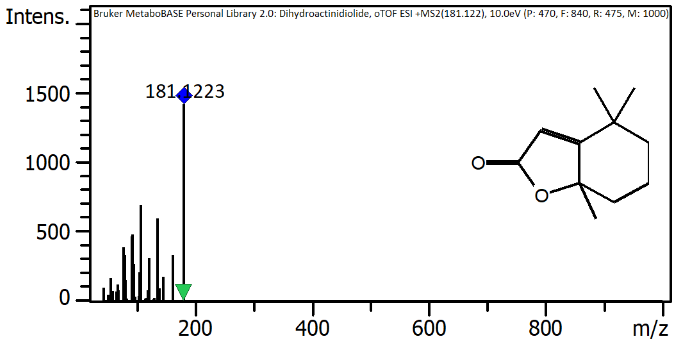

#	m/z	Res.	S/N	I	I %	FWHM
1	77.0390	6871	46.0	391	27.6	0.0112
2	79.0547	7051	39.7	338	23.8	0.0112
3	91.0545	8121	55.2	469	33.1	0.0112
4	93.0701	8301	57.0	485	34.2	0.0112
5	95.0855	8480	32.3	275	19.4	0.0112
6	107.0854	9551	81.8	696	49.1	0.0112
7	121.1003	10801	36.8	313	22.1	0.0112
8	135.1165	12051	70.5	600	42.3	0.0112
9	163.1114	14548	39.5	336	23.7	0.0112
10	181.1223	16154	166.5	1417	100.0	0.0112

Cmpd 1172, AutoMSn(331.2826), 14.09 min

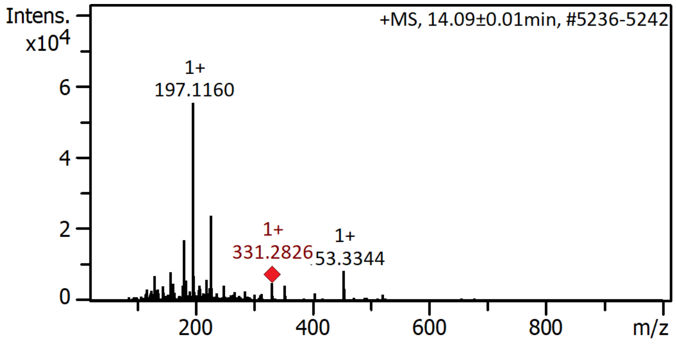

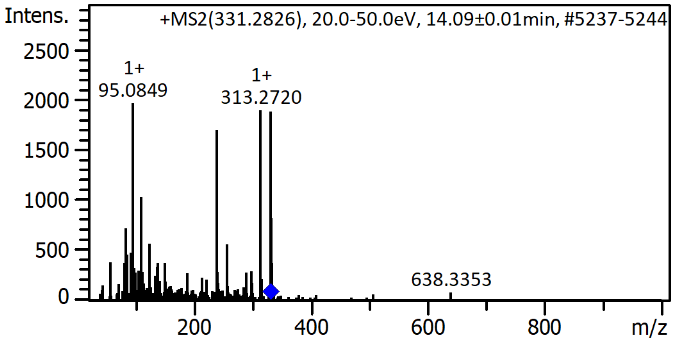

#	m/z	Res.	S/N	I	I %	FWHM
1	130.1583	9516	575.4	6905	12.5	0.0137
2	157.0825	10633	657.2	7887	14.3	0.0148
3	181.1218	10634	1424.9	17098	30.9	0.0170
4	185.0423	11559	462.1	5545	10.0	0.0160
5	197.1160	11522	4607.9	55295	100.0	0.0171
6	198.1188	11855	575.4	6904	12.5	0.0167
7	219.0980	12205	486.3	5836	10.6	0.0180
8	227.1625	12034	1995.3	23943	43.3	0.0189
9	331.2826	13073	411.1	4933	8.9	0.0253
10	453.3344	11975	699.6	8395	15.2	0.0379

#	m/z	Res.	S/N	I	I %	FWHM
1	83.0859	10171	44.1	720	36.7	0.0082
2	95.0849	9132	120.2	1964	100.0	0.0104
3	109.0999	10653	63.2	1032	52.6	0.0102
4	123.1173	9453	35.0	572	29.1	0.0130
5	239.2330	12355	103.8	1695	86.3	0.0194
6	257.2441	16860	34.4	563	28.6	0.0153
7	313.2720	14434	116.0	1894	96.5	0.0217
8	331.0807	15082	115.1	1880	95.7	0.0220
9	331.1965	11510	52.5	858	43.7	0.0288
10	332.3354	16075	50.6	827	42.1	0.0207

Compound Spectrum List Report

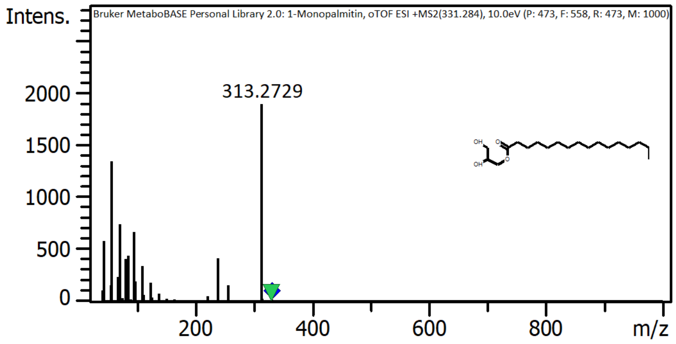

#	m/z	Res.	S/N	I	I %	FWHM
1	43.0545	6214	51.3	583	30.8	0.0069
2	57.0698	8237	118.3	1345	71.1	0.0069
3	71.0853	10259	65.7	746	39.4	0.0069
4	81.0695	11700	36.3	413	21.8	0.0069
5	83.0852	11991	31.7	360	19.0	0.0069
6	85.1007	12282	39.2	445	23.5	0.0069
7	95.0850	13723	59.0	671	35.4	0.0069
8	109.1007	15746	30.0	341	18.0	0.0069
9	239.2372	34528	37.2	422	22.3	0.0069
10	313.2729	45213	166.5	1892	100.0	0.0069

Cmpd 1175, AutoMSn(181.1216), 14.11 min

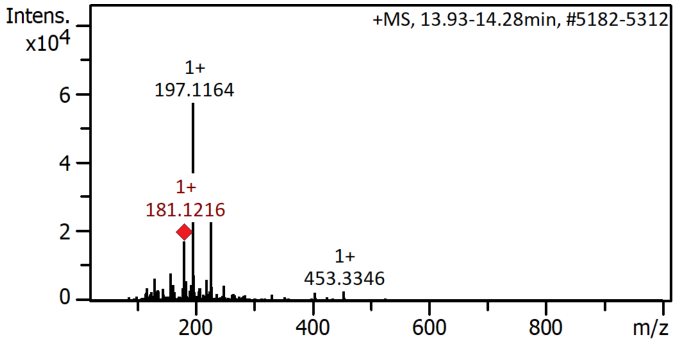

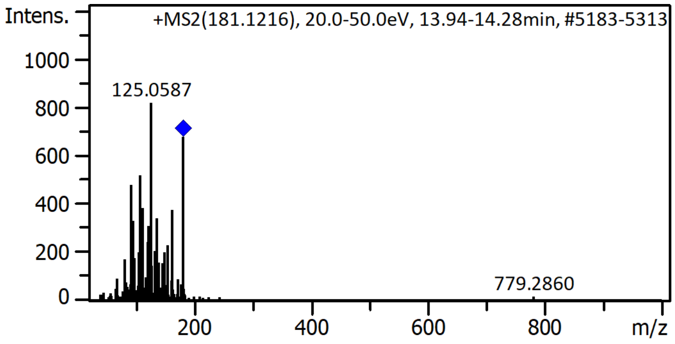

#	m/z	Res.	S/N	I	I %	FWHM
1	130.1581	10486	1587.3	6349	11.1	0.0124
2	157.0825	11194	1956.1	7824	13.6	0.0140
3	163.0583	10846	1145.4	4581	8.0	0.0150
4	181.1216	11461	4324.7	17299	30.2	0.0158
5	185.0410	11477	1399.0	5596	9.8	0.0161
6	193.1572	11643	1133.0	4532	7.9	0.0166
7	197.1164	11696	14333.6	57334	100.0	0.0169
8	198.1200	11304	1852.1	7409	12.9	0.0175
9	219.0985	12349	1489.3	5957	10.4	0.0177
10	227.1633	11649	6480.0	25920	45.2	0.0195

#	m/z	Res.	S/N	I	I %	FWHM
1	91.0537	10878	86.5	480	58.8	0.0084
2	93.0694	8623	59.4	330	40.4	0.0108
3	95.0849	9834	59.7	332	40.6	0.0097
4	107.0837	7842	93.2	518	63.4	0.0137
5	111.0421	12829	69.0	383	46.9	0.0087
6	121.0990	9636	55.7	309	37.9	0.0126
7	125.0587	11446	147.0	817	100.0	0.0109
8	135.1163	10757	61.7	343	41.9	0.0126
9	163.1130	13581	67.8	376	46.1	0.0120
10	181.1236	11487	122.1	678	83.0	0.0158

Compound Spectrum List Report

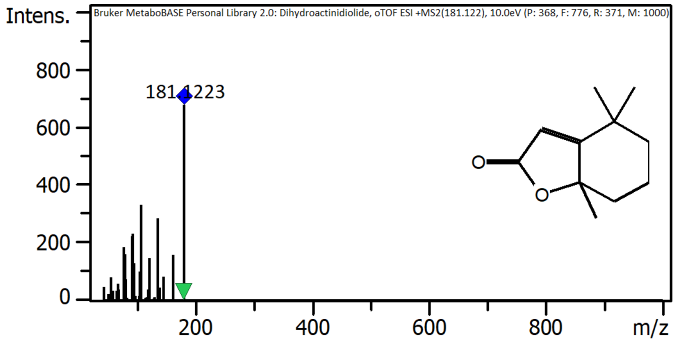

#	m/z	Res.	S/N	I	I %	FWHM
1	77.0390	7242	46.0	187	27.6	0.0106
2	79.0547	7431	39.7	161	23.8	0.0106
3	91.0545	8559	55.2	224	33.1	0.0106
4	93.0701	8748	57.0	232	34.2	0.0106
5	95.0855	8938	32.3	132	19.4	0.0106
6	107.0854	10066	81.8	333	49.1	0.0106
7	121.1003	11383	36.8	150	22.1	0.0106
8	135.1165	12701	70.5	287	42.3	0.0106
9	163.1114	15332	39.5	161	23.7	0.0106
10	181.1223	17025	166.5	678	100.0	0.0106

Cmpd 1194, AutoMSn(303.1933), 14.42 min

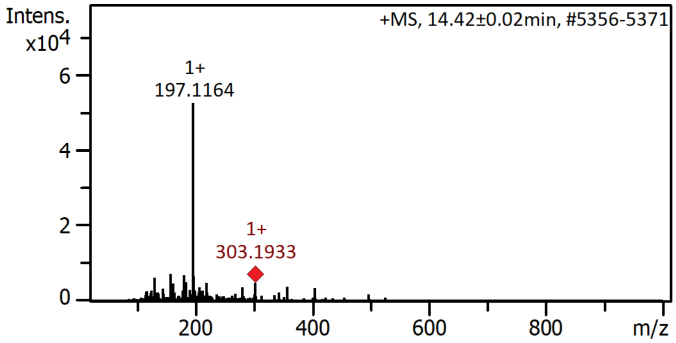

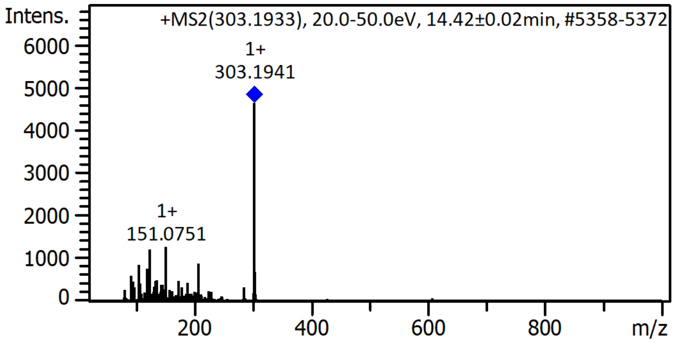

#	m/z	Res.	S/N	I	I %	FWHM
1	130.1583	10973	680.6	6126	11.7	0.0119
2	157.0829	10788	805.5	7250	13.8	0.0146
3	163.0589	9825	512.4	4612	8.8	0.0166
4	181.1213	11136	764.3	6879	13.1	0.0163
5	185.0412	11810	564.1	5077	9.7	0.0157
6	197.1164	11818	5829.5	52466	100.0	0.0167
7	198.1195	11772	720.0	6480	12.4	0.0168
8	219.0982	12538	541.8	4876	9.3	0.0175
9	303.1933	14135	540.1	4861	9.3	0.0214
10	357.2579	15137	427.1	3844	7.3	0.0236

#	m/z	Res.	S/N	I	I %	FWHM
1	91.0537	11268	48.7	597	12.9	0.0081
2	105.0686	9940	69.6	853	18.4	0.0106
3	119.0859	9155	62.2	762	16.4	0.0130
4	123.0803	10363	99.2	1216	26.2	0.0119
5	133.0999	13599	38.6	473	10.2	0.0098
6	135.0817	11527	39.7	486	10.5	0.0117
7	151.0751	11610	104.2	1276	27.5	0.0130
8	207.1339	13912	71.1	872	18.8	0.0149
9	303.1941	10907	378.1	4632	100.0	0.0278
10	304.1976	14107	55.8	684	14.8	0.0216

Compound Spectrum List Report

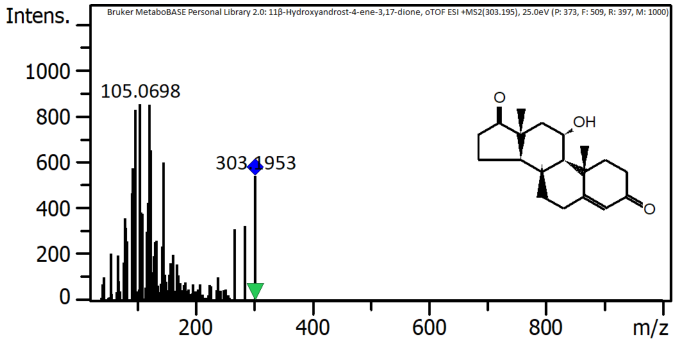

#	m/z	Res.	S/N	I	I %	FWHM
1	91.0544	3516	109.4	467	54.8	0.0259
2	93.0701	3594	135.0	576	67.6	0.0259
3	97.0651	3748	193.8	827	97.0	0.0259
4	105.0698	4057	199.8	852	100.0	0.0259
5	107.0854	4135	90.2	385	45.1	0.0259
6	119.0852	4598	100.0	427	50.1	0.0259
7	121.0648	4675	199.0	849	99.6	0.0259
8	123.0799	4753	153.0	653	76.6	0.0259
9	145.1008	5603	140.8	601	70.5	0.0259
10	303.1953	11707	127.0	542	63.6	0.0259

Cmpd 1205, AutoMSn(181.1209), 14.54 min

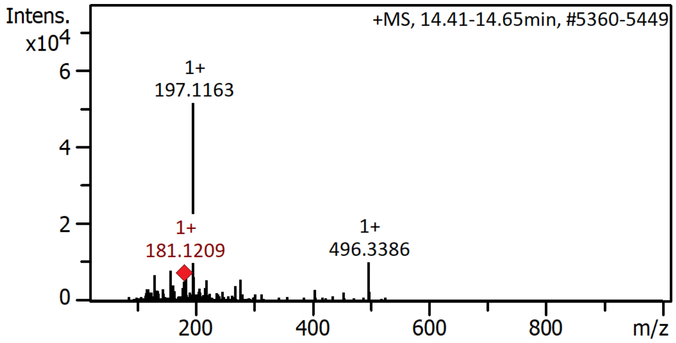

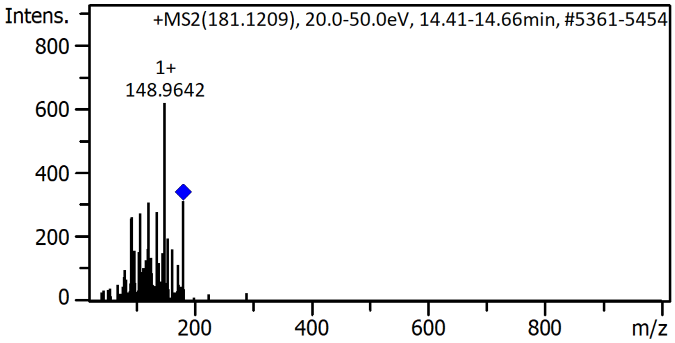

#	m/z	Res.	S/N	I	I %	FWHM
1	130.1588	10309	1117.0	6702	13.0	0.0126
2	157.0828	10707	1309.8	7859	15.3	0.0147
3	163.0589	10213	692.6	4156	8.1	0.0160
4	181.1209	11487	848.0	5088	9.9	0.0158
5	185.0415	12339	943.4	5661	11.0	0.0150
6	197.1163	11489	8565.1	51391	100.0	0.0172
7	198.1201	10049	1031.2	6187	12.0	0.0197
8	219.0992	10999	899.4	5397	10.5	0.0199
9	277.2153	11236	924.4	5546	10.8	0.0247
10	496.3386	12965	1694.3	10166	19.8	0.0383

#	m/z	Res.	S/N	I	I %	FWHM
1	91.0539	10904	30.5	260	42.0	0.0084
2	93.0669	8520	30.8	262	42.4	0.0109
3	107.0861	8751	32.3	275	44.4	0.0122
4	120.0823	8428	19.4	165	26.6	0.0142
5	121.0995	9951	36.4	310	50.1	0.0122
6	135.1144	12497	32.8	279	45.1	0.0108
7	148.9642	12626	72.7	618	100.0	0.0118
8	154.0861	15364	23.2	197	31.8	0.0100
9	163.1110	15149	19.2	164	26.4	0.0108
10	181.1171	12311	36.9	314	50.7	0.0147

Compound Spectrum List Report

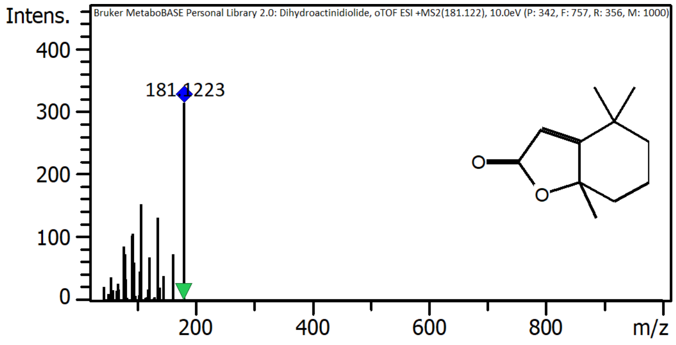

#	m/z	Res.	S/N	I	I %	FWHM
1	77.0390	6830	46.0	87	27.6	0.0113
2	79.0547	7009	39.7	75	23.8	0.0113
3	91.0545	8073	55.2	104	33.1	0.0113
4	93.0701	8252	57.0	107	34.2	0.0113
5	95.0855	8430	32.3	61	19.4	0.0113
6	107.0854	9494	81.8	154	49.1	0.0113
7	121.1003	10737	36.8	69	22.1	0.0113
8	135.1165	11980	70.5	133	42.3	0.0113
9	163.1114	14462	39.5	74	23.7	0.0113
10	181.1223	16058	166.5	313	100.0	0.0113

Cmpd 1208, AutoMSn(277.2147), 14.57 min

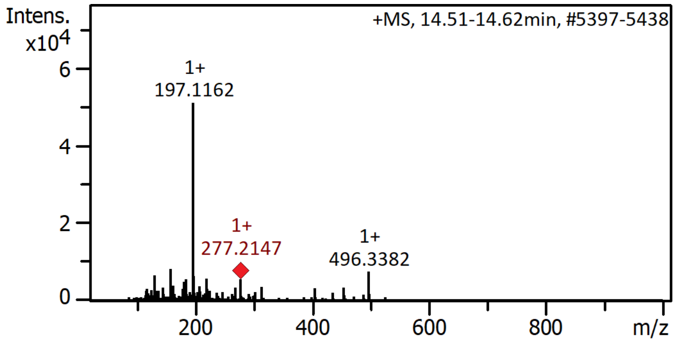

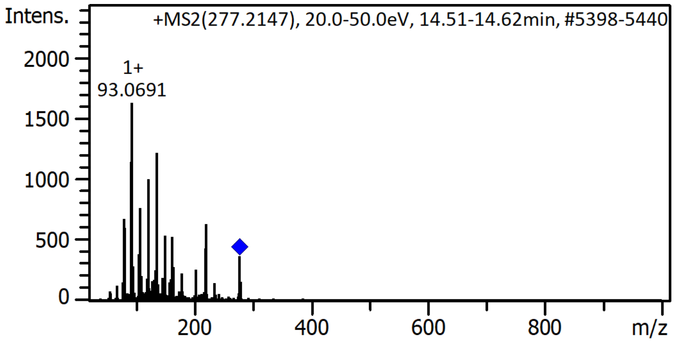

#	m/z	Res.	S/N	I	I %	FWHM
1	130.1582	10474	1271.9	6541	12.8	0.0124
2	157.0830	11366	1590.3	8179	16.0	0.0138
3	163.0594	9555	760.1	3909	7.7	0.0171
4	181.1216	11656	936.6	4817	9.4	0.0155
5	185.0408	12415	1084.5	5577	10.9	0.0149
6	197.1162	11515	9915.1	50992	100.0	0.0171
7	198.1188	11127	1246.3	6409	12.6	0.0178
8	219.0987	12155	1105.3	5684	11.1	0.0180
9	277.2147	13018	1078.4	5546	10.9	0.0213
10	496.3382	12351	1455.4	7485	14.7	0.0402

#	m/z	Res.	S/N	I	I %	FWHM
1	79.0536	9343	96.6	676	41.6	0.0085
2	81.0699	7719	85.7	600	36.9	0.0105
3	91.0534	10080	163.2	1143	70.3	0.0090
4	93.0691	10214	232.3	1626	100.0	0.0091
5	107.0866	9231	109.1	764	47.0	0.0116
6	121.1003	10291	143.5	1005	61.8	0.0118
7	135.1156	11811	174.1	1219	75.0	0.0114
8	149.0217	10702	77.1	540	33.2	0.0139
9	161.0945	10464	75.6	529	32.6	0.0154
10	221.1181	10300	90.4	633	38.9	0.0215

Compound Spectrum List Report

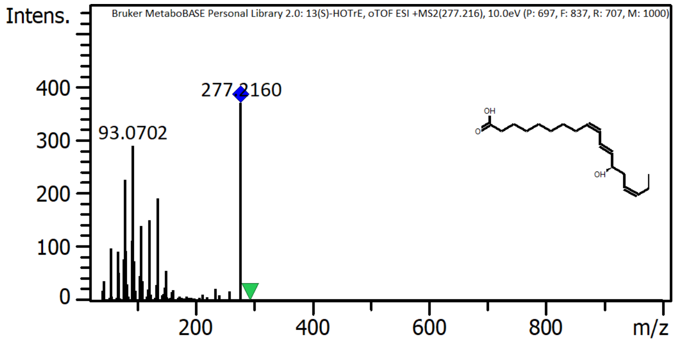

#	m/z	Res.	S/N	I	I %	FWHM
1	55.0552	6946	53.4	99	26.7	0.0079
2	67.0543	8460	50.2	93	25.1	0.0079
3	79.0545	9974	122.4	226	61.3	0.0079
4	81.0699	10228	51.0	94	25.5	0.0079
5	91.0543	11488	61.2	113	30.6	0.0079
6	93.0702	11742	156.4	289	78.3	0.0079
7	107.0851	13511	75.8	140	37.9	0.0079
8	121.1014	15279	81.8	151	40.9	0.0079
9	135.1165	17047	103.8	192	52.0	0.0079
10	277.2160	34975	199.8	369	100.0	0.0079

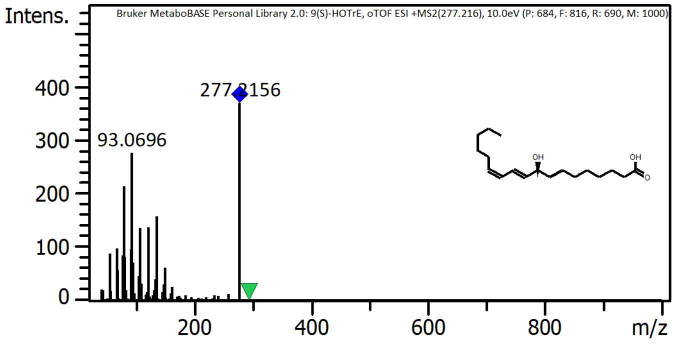

#	m/z	Res.	S/N	I	I %	FWHM
1	55.0550	6946	48.0	89	24.0	0.0079
2	67.0541	8460	53.4	99	26.7	0.0079
3	77.0384	9720	46.0	85	23.0	0.0079
4	79.0542	9974	116.0	215	58.1	0.0079
5	91.0543	11488	52.8	98	26.4	0.0079
6	93.0696	11742	149.6	277	74.9	0.0079
7	107.0852	13511	74.2	137	37.1	0.0079
8	121.1007	15279	74.8	138	37.4	0.0079
9	135.1163	17047	85.6	158	42.8	0.0079
10	277.2156	34975	199.8	369	100.0	0.0079

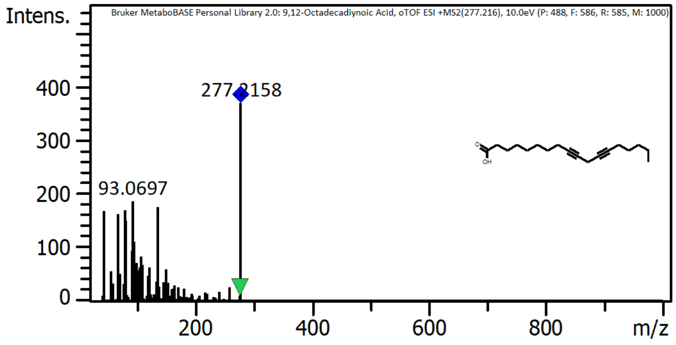

#	m/z	Res.	S/N	I	I %	FWHM
1	43.0550	5432	91.4	169	45.7	0.0079
2	67.0556	8460	88.0	163	44.0	0.0079
3	79.0544	9974	92.0	170	46.0	0.0079
4	81.0704	10228	81.8	151	40.9	0.0079
5	91.0537	11488	51.4	95	25.7	0.0079
6	93.0697	11742	101.0	187	50.6	0.0079
7	95.0855	11997	60.6	112	30.3	0.0079
8	107.0856	13511	45.6	84	22.8	0.0079
9	135.1169	17047	95.6	177	47.8	0.0079
10	277.2158	34975	199.8	369	100.0	0.0079

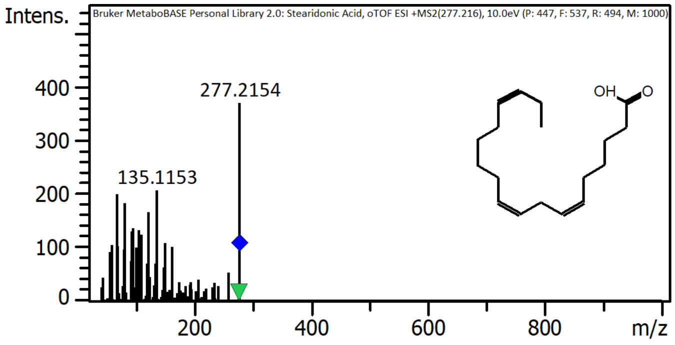

#	m/z	Res.	S/N	I	I %	FWHM
1	67.0544	8460	108.0	200	54.1	0.0079
2	81.0708	10228	99.4	184	49.7	0.0079
3	93.0682	11742	70.8	131	35.4	0.0079
4	95.0851	11997	74.0	137	37.0	0.0079
5	105.0691	13256	71.8	133	35.9	0.0079
6	107.0859	13511	61.6	114	30.8	0.0079
7	109.1002	13765	67.8	125	33.9	0.0079
8	121.1007	15279	90.2	167	45.1	0.0079
9	135.1153	17047	112.2	207	56.2	0.0079
10	277.2154	34975	199.8	369	100.0	0.0079

Cmpd 1211, AutoMSn(496.3380), 14.59 min

Compound Spectrum List Report

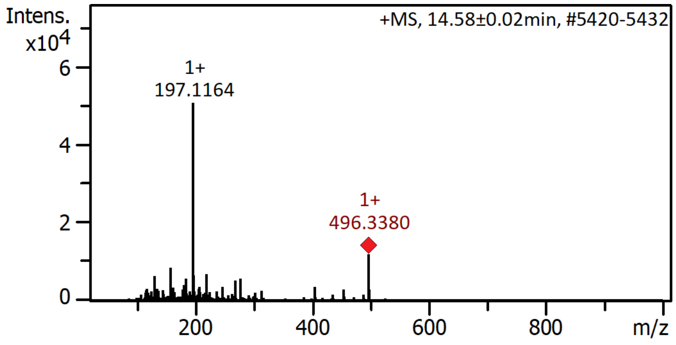

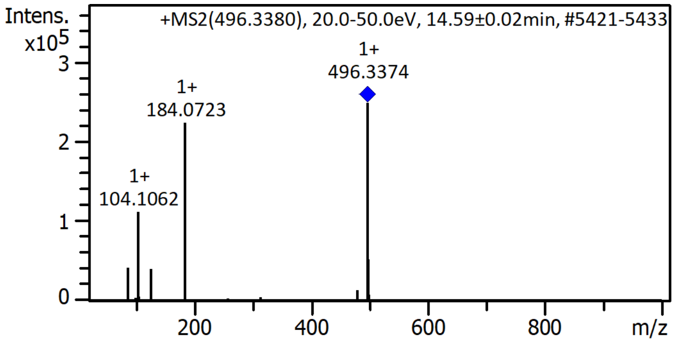

#	m/z	Res.	S/N	I	I %	FWHM
1	130.1586	10908	524.9	6299	12.4	0.0119
2	157.0823	11059	706.6	8479	16.7	0.0142
3	181.1208	12572	338.1	4058	8.0	0.0144
4	185.0404	12339	473.3	5680	11.2	0.0150
5	197.1164	11388	4224.0	50688	100.0	0.0173
6	198.1202	12181	535.8	6430	12.7	0.0163
7	219.0988	13076	571.1	6853	13.5	0.0168
8	269.2064	11109	431.7	5181	10.2	0.0242
9	277.2143	12108	473.5	5682	11.2	0.0229
10	496.3380	12408	998.4	11981	23.6	0.0400

#	m/z	Res.	S/N	I	I %	FWHM
1	86.0959	8893	2607.9	41727	16.8	0.0097
2	104.1062	9262	7005.5	112087	45.2	0.0112
3	105.1098	9025	306.9	4910	2.0	0.0116
4	124.9988	10628	2506.9	40111	16.2	0.0118
5	184.0723	11120	13989.1	223825	90.2	0.0166
6	185.0758	10886	639.2	10227	4.1	0.0170
7	478.3276	13246	817.5	13080	5.3	0.0361
8	496.3374	12768	15512.5	248200	100.0	0.0389
9	497.3408	12945	3296.2	52739	21.2	0.0384
10	498.3421	13462	492.5	7880	3.2	0.0370

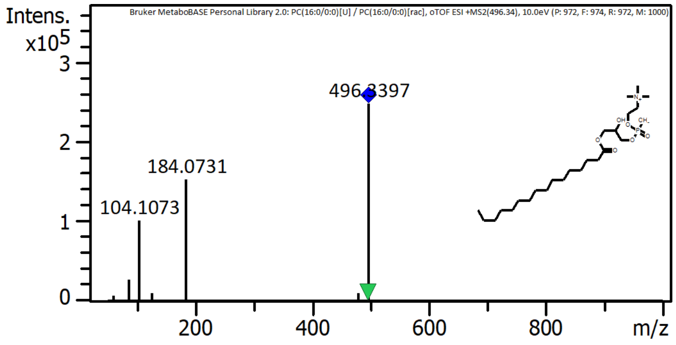

#	m/z	Res.	S/N	I	I %	FWHM
1	57.0723	1314	1.8	2234	0.9	0.0434
2	60.0822	1383	5.8	7198	2.9	0.0434
3	86.0970	1982	21.6	26806	10.8	0.0434
4	104.1072	2396	65.6	81410	32.8	0.0434
5	104.1073	2396	81.8	101514	40.9	0.0434
6	124.9992	2877	8.4	10424	4.2	0.0434
7	184.0731	4237	123.0	152643	61.6	0.0434
8	478.3286	11010	6.6	8191	3.3	0.0434
9	478.3301	11010	7.8	9680	3.9	0.0434
10	496.3397	11424	199.8	247952	100.0	0.0434

Cmpd 1214, AutoMSn(217.1574), 14.64 min

Compound Spectrum List Report

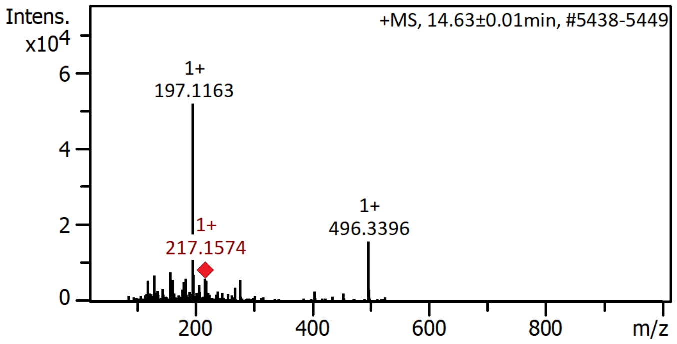

#	m/z	Res.	S/N	I	I %	FWHM
1	119.0850	8889	303.3	5459	10.5	0.0134
2	130.1587	10329	379.0	6822	13.2	0.0126
3	157.0831	11045	420.7	7572	14.6	0.0142
4	161.0949	10771	307.3	5532	10.7	0.0150
5	185.0420	12730	333.9	6011	11.6	0.0145
6	197.1163	11533	2878.4	51811	100.0	0.0171
7	198.1198	11881	387.6	6976	13.5	0.0167
8	217.1574	11620	328.6	5915	11.4	0.0187
9	277.2162	10456	313.9	5650	10.9	0.0265
10	496.3396	12580	882.2	15880	30.6	0.0395

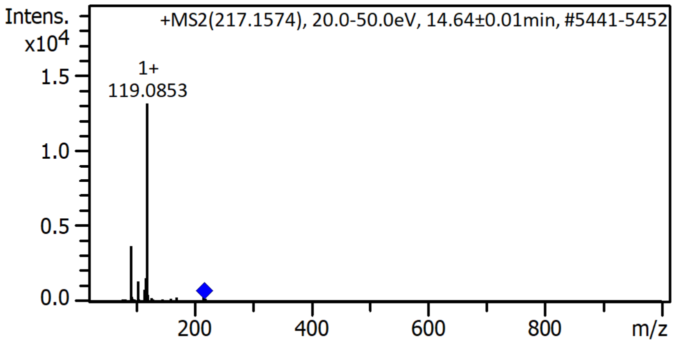

#	m/z	Res.	S/N	I	I %	FWHM
1	91.0554	7919	147.7	3693	28.2	0.0115
2	93.0680	8956	13.1	329	2.5	0.0104
3	103.0542	11253	40.7	1017	7.8	0.0092
4	104.0614	9242	53.2	1331	10.2	0.0113
5	115.0537	13177	31.4	786	6.0	0.0087
6	117.0695	8076	61.8	1544	11.8	0.0145
7	118.0755	10864	11.8	295	2.3	0.0109
8	119.0853	10078	524.4	13110	100.0	0.0118
9	120.0896	12213	17.8	445	3.4	0.0098
10	216.1194	17976	13.5	337	2.6	0.0120

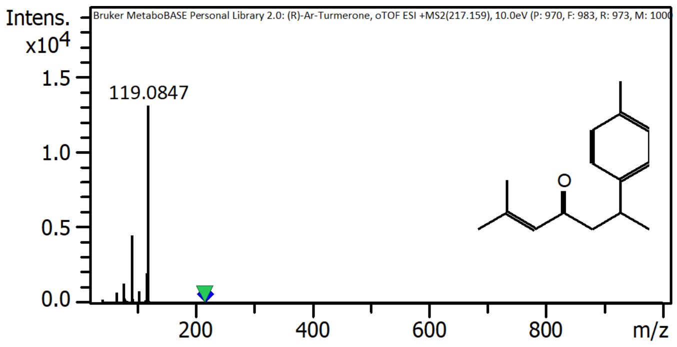

#	m/z	Res.	S/N	I	I %	FWHM
1	65.0384	5315	5.3	695	5.3	0.0122
2	77.0381	6296	10.0	1311	10.0	0.0122
3	77.0382	6296	9.2	1206	9.2	0.0122
4	78.0459	6378	2.4	315	2.4	0.0122
5	79.0543	6460	1.8	236	1.8	0.0122
6	91.0537	7441	34.4	4510	34.4	0.0122
7	93.0694	7606	2.0	262	2.0	0.0122
8	103.0533	8422	5.9	773	5.9	0.0122
9	117.0690	9567	15.0	1967	15.0	0.0122
10	119.0847	9732	99.9	13097	100.0	0.0122

Cmpd 1230, AutoMSn(291.2291), 14.85 min

Compound Spectrum List Report

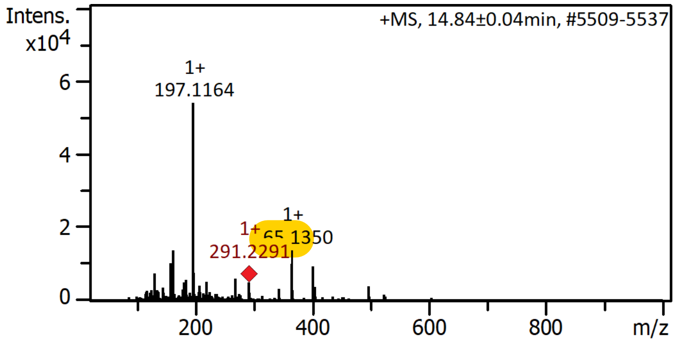

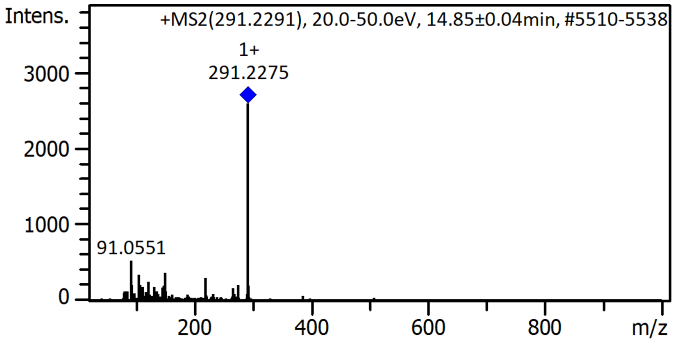

#	m/z	Res.	S/N	I	I %	FWHM
1	130.1582	10788	1029.4	7411	13.7	0.0121
2	157.0831	10903	1430.1	10297	19.1	0.0144
3	163.0733	9780	1934.1	13925	25.8	0.0167
4	185.0417	10598	799.4	5755	10.7	0.0175
5	197.1164	11373	7499.5	53996	100.0	0.0173
6	198.1196	11068	1046.2	7532	13.9	0.0179
7	219.0976	12049	721.8	5197	9.6	0.0182
8	269.2475	10099	847.8	6104	11.3	0.0267
9	365.1350	12843	1933.7	13922	25.8	0.0284
10	401.2895	12817	1292.4	9305	17.2	0.0313

#	m/z	Res.	S/N	I	I %	FWHM
1	91.0551	9171	54.0	529	20.4	0.0099
2	93.0699	12348	20.9	205	7.9	0.0075
3	105.0683	11425	35.1	344	13.2	0.0092
4	107.0844	11834	21.1	207	8.0	0.0090
5	121.1019	9309	25.1	246	9.5	0.0130
6	149.0940	12737	37.0	363	14.0	0.0117
7	219.1747	18063	30.3	297	11.4	0.0121
8	275.1993	18149	21.0	206	7.9	0.0152
9	291.2275	12572	264.8	2595	100.0	0.0232
10	292.2358	15681	54.1	530	20.4	0.0186

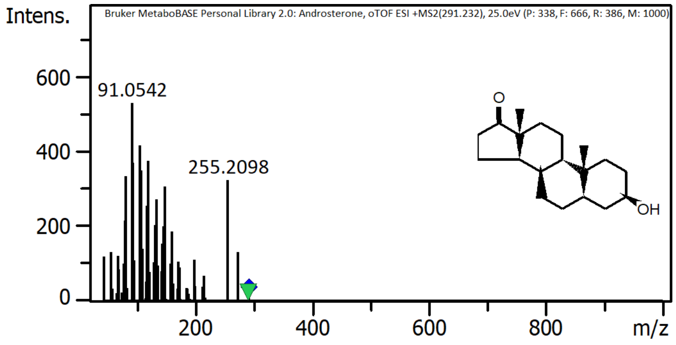

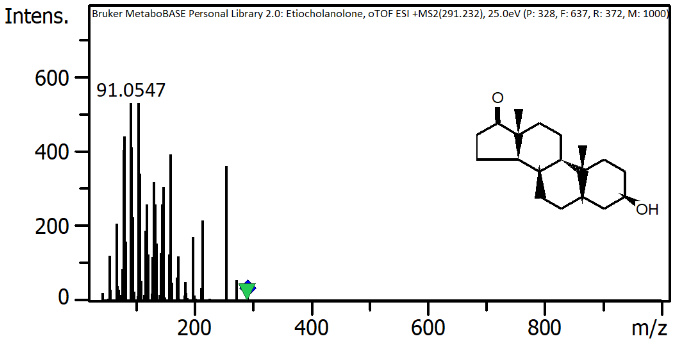

#	m/z	Res.	S/N	I	I %	FWHM
1	81.0704	3591	126.4	334	63.3	0.0226
2	91.0542	4034	199.8	529	100.0	0.0226
3	93.0708	4123	140.2	371	70.2	0.0226
4	105.0695	4655	157.2	416	78.7	0.0226
5	107.0850	4744	132.2	350	66.2	0.0226
6	117.0691	5186	96.4	255	48.2	0.0226
7	119.0843	5275	141.8	375	71.0	0.0226
8	133.1012	5896	102.8	272	51.5	0.0226
9	147.1174	6517	116.2	307	58.2	0.0226
10	255.2098	11306	122.4	324	61.3	0.0226

#	m/z	Res.	S/N	I	I %	FWHM
1	79.0542	3502	152.6	404	76.4	0.0226
2	81.0695	3591	166.4	440	83.3	0.0226
3	91.0547	4034	199.8	529	100.0	0.0226
4	93.0699	4123	155.2	411	77.7	0.0226
5	105.0691	4655	199.8	529	100.0	0.0226
6	107.0853	4744	128.8	341	64.5	0.0226
7	131.0843	5807	120.4	319	60.3	0.0226
8	147.1156	6517	115.6	306	57.9	0.0226
9	159.1156	7049	148.2	392	74.2	0.0226
10	255.2099	11306	137.0	363	68.6	0.0226

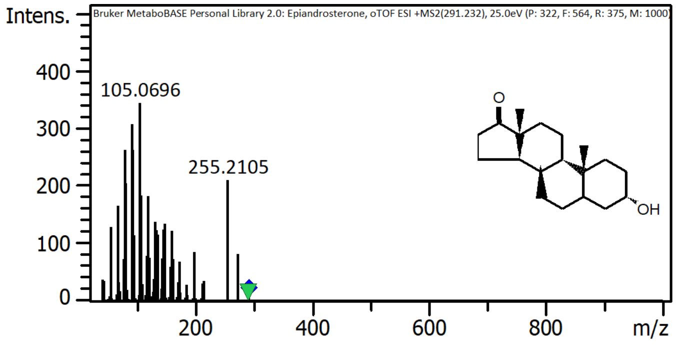

Compound Spectrum List Report

#	m/z	Res.	S/N	I	I %	FWHM
1	67.0545	2970	96.4	166	48.2	0.0226
2	79.0545	3502	152.8	263	76.5	0.0226
3	81.0698	3591	119.0	204	59.6	0.0226
4	91.0540	4034	179.0	308	89.6	0.0226
5	93.0696	4123	152.6	262	76.4	0.0226
6	105.0696	4655	199.8	343	100.0	0.0226
7	107.0854	4744	107.2	184	53.7	0.0226
8	119.0849	5275	106.0	182	53.1	0.0226
9	131.0848	5807	80.4	138	40.2	0.0226
10	255.2105	11306	122.2	210	61.2	0.0226

Cmpd 1251, AutoMSn(124.0379), 15.17 min

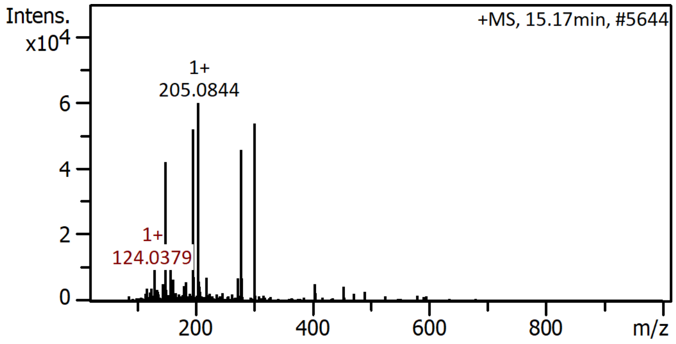

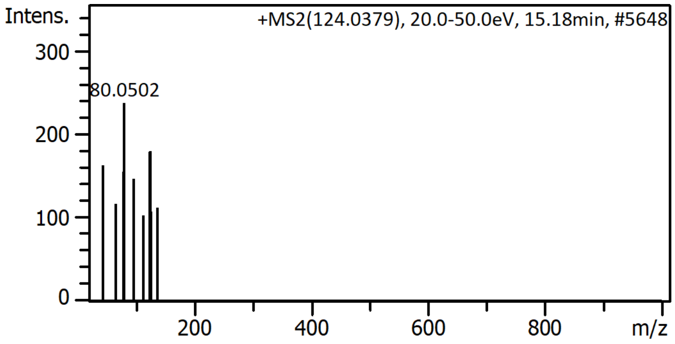

#	m/z	Res.	S/N	I	I %	FWHM
1	130.1577	11501	273.6	9850	16.5	0.0113
2	149.0222	10644	1170.4	42135	70.5	0.0140
3	157.0821	10828	363.1	13072	21.9	0.0145
4	197.1165	11595	1443.5	51965	86.9	0.0170
5	198.1185	11036	199.8	7192	12.0	0.0180
6	205.0844	11032	1660.6	59780	100.0	0.0186
7	219.0999	8815	196.1	7058	11.8	0.0249
8	279.1568	12335	1271.3	45766	76.6	0.0226
9	301.1383	12308	1491.8	53705	89.8	0.0245
10	302.1421	12631	304.8	10972	18.4	0.0239

#	m/z	Res.	S/N	I	I %	FWHM
1	44.0477	8214	1.6	163	68.8	0.0054
2	64.9255	11208	1.1	117	49.4	0.0058
3	78.0394	11521	1.5	155	65.4	0.0068
4	80.0502	5047	2.3	237	100.0	0.0159
5	96.0419	12491	1.4	147	62.0	0.0077
6	123.0569	11724	1.7	179	75.5	0.0105
7	124.0378	15025	1.7	180	75.9	0.0083
8	124.0594	15336	1.3	132	55.7	0.0081
9	126.0560	15470	1.0	108	45.6	0.0081
10	135.9644	15464	1.1	112	47.3	0.0088

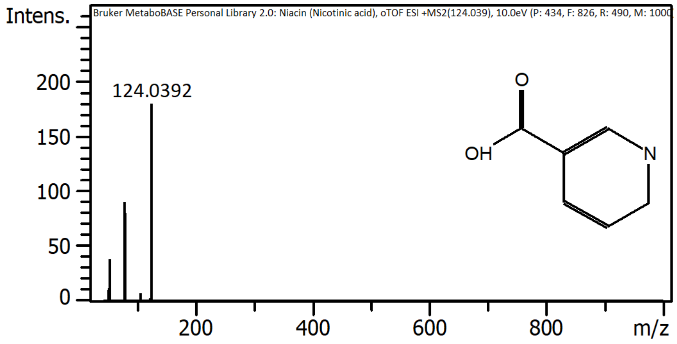

#	m/z	Res.	S/N	I	I %	FWHM
1	44.9973	6601	1.4	1	0.7	0.0068
2	51.0229	7485	11.4	10	5.7	0.0068
3	52.0246	7632	13.2	12	6.6	0.0068
4	53.0389	7781	43.0	39	21.5	0.0068
5	78.0344	11448	100.4	90	50.3	0.0068
6	79.0422	11596	10.2	9	5.1	0.0068
7	80.0499	11744	89.6	81	44.8	0.0068
8	106.0288	15555	8.0	7	4.0	0.0068
9	122.0236	17902	2.6	2	1.3	0.0068
10	124.0392	18197	199.8	180	100.0	0.0068

Compound Spectrum List Report

Cmpd 1279, AutoMSn(181.1217), 15.56 min

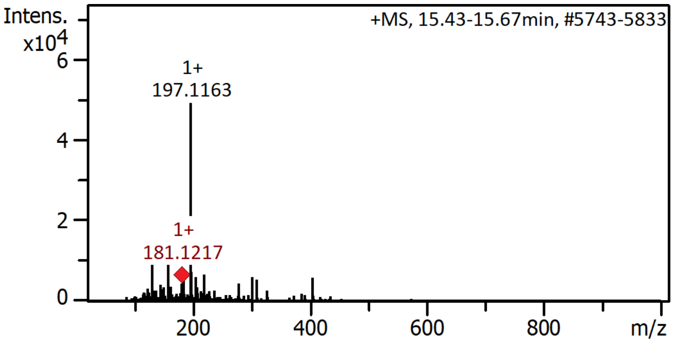

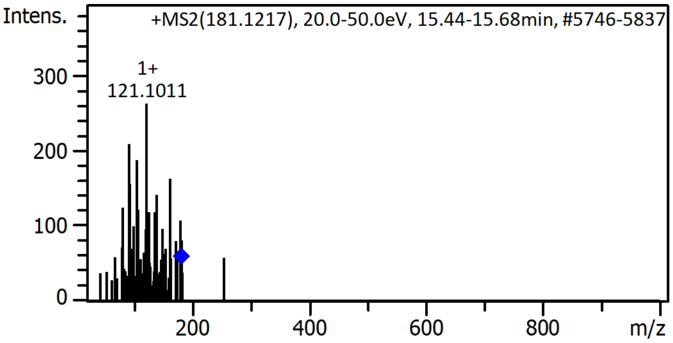

#	m/z	Res.	S/N	I	I %	FWHM
1	130.1583	10890	990.0	8910	18.2	0.0120
2	157.0829	11620	1353.4	12181	24.8	0.0135
3	185.0417	11440	750.1	6751	13.8	0.0162
4	197.1163	11219	5449.1	49042	100.0	0.0176
5	198.1188	9830	803.8	7234	14.8	0.0202
6	205.0851	11343	656.3	5907	12.0	0.0181
7	219.0973	12361	737.6	6638	13.5	0.0177
8	301.1402	13393	663.6	5973	12.2	0.0225
9	309.2106	10532	590.5	5314	10.8	0.0294
10	404.3167	12746	651.0	5859	11.9	0.0317

#	m/z	Res.	S/N	I	I %	FWHM
1	81.0315	11950	8.5	125	47.5	0.0068
2	91.0528	10472	14.2	209	79.6	0.0087
3	93.0698	12528	10.6	156	59.5	0.0074
4	105.0692	13303	12.7	188	71.4	0.0079
5	107.0854	13667	8.3	122	46.3	0.0078
6	121.1011	10591	17.8	263	100.0	0.0114
7	125.0571	14647	8.0	118	45.0	0.0085
8	135.1192	13071	8.1	119	45.3	0.0103
9	138.0621	13168	9.6	142	54.0	0.0105
10	163.1080	11713	11.1	163	62.0	0.0139

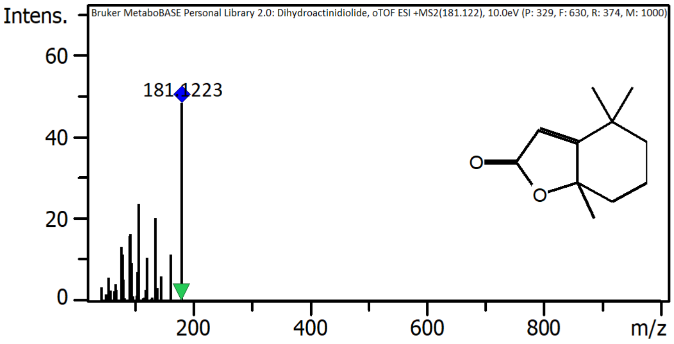

#	m/z	Res.	S/N	I	I %	FWHM
1	77.0390	9041	46.0	13	27.6	0.0085
2	79.0547	9277	39.7	11	23.8	0.0085
3	91.0545	10685	55.2	16	33.1	0.0085
4	93.0701	10922	57.0	17	34.2	0.0085
5	95.0855	11158	32.3	9	19.4	0.0085
6	107.0854	12567	81.8	24	49.1	0.0085
7	121.1003	14211	36.8	11	22.1	0.0085
8	135.1165	15856	70.5	20	42.3	0.0085
9	163.1114	19141	39.5	11	23.7	0.0085
10	181.1223	21255	166.5	48	100.0	0.0085

Cmpd 1283, AutoMSn(124.0378), 15.60 min

Compound Spectrum List Report

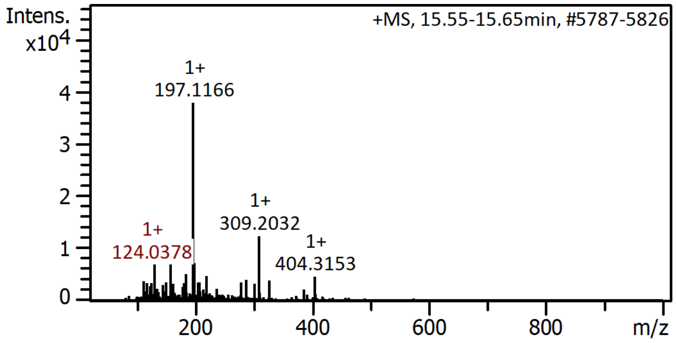

#	m/z	Res.	S/N	I	I %	FWHM
1	130.1585	9122	446.6	8040	21.3	0.0143
2	157.0828	10609	595.3	10716	28.4	0.0148
3	185.0416	11022	281.9	5075	13.4	0.0168
4	197.1166	10881	2099.5	37791	100.0	0.0181
5	198.1208	10931	313.6	5644	14.9	0.0181
6	199.1692	11700	394.5	7101	18.8	0.0170
7	219.0996	10598	263.5	4743	12.5	0.0207
8	287.2195	13530	219.0	3942	10.4	0.0212
9	309.2032	11421	691.6	12450	32.9	0.0271
10	404.3153	12446	257.9	4643	12.3	0.0325

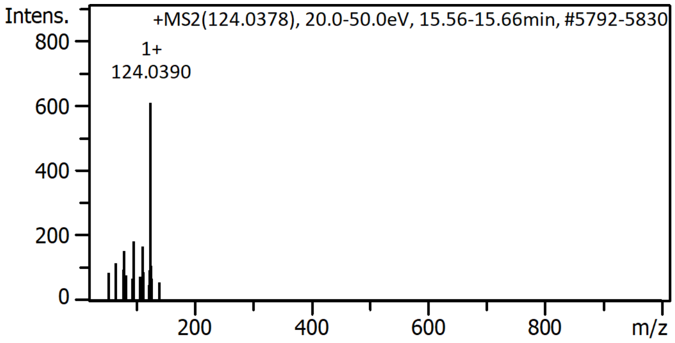

#	m/z	Res.	S/N	I	I %	FWHM
1	64.9260	10886	2.4	115	18.9	0.0060
2	78.0450	12000	2.0	96	15.8	0.0065
3	80.0492	7904	3.2	154	25.4	0.0101
4	96.0482	11249	3.8	184	30.2	0.0085
5	111.0339	13592	3.5	167	27.5	0.0082
6	112.0354	13815	1.8	88	14.4	0.0081
7	123.0410	14882	1.9	93	15.3	0.0083
8	124.0390	11614	12.6	607	100.0	0.0107
9	124.0634	14226	7.5	361	59.5	0.0087
10	125.0395	14823	2.2	107	17.6	0.0084

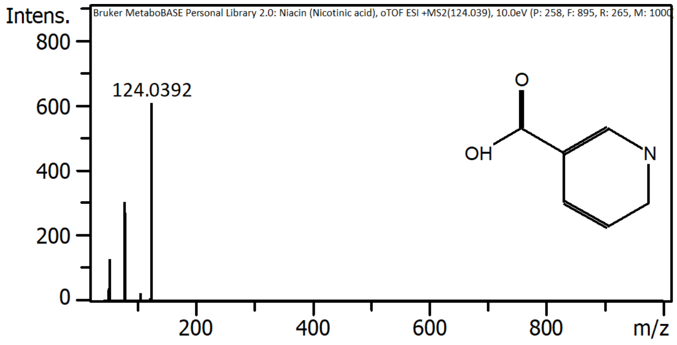

#	m/z	Res.	S/N	I	I %	FWHM
1	44.9973	4588	1.4	4	0.7	0.0098
2	51.0229	5203	11.4	35	5.7	0.0098
3	52.0246	5305	13.2	40	6.6	0.0098
4	53.0389	5408	43.0	131	21.5	0.0098
5	78.0344	7957	100.4	305	50.3	0.0098
6	79.0422	8060	10.2	31	5.1	0.0098
7	80.0499	8162	89.6	272	44.8	0.0098
8	106.0288	10811	8.0	24	4.0	0.0098
9	122.0236	12442	2.6	8	1.3	0.0098
10	124.0392	12648	199.8	606	100.0	0.0098

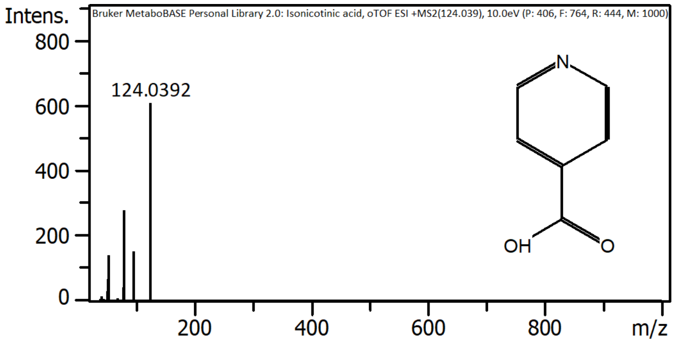

#	m/z	Res.	S/N	I	I %	FWHM
1	41.0392	4185	4.8	15	2.4	0.0098
2	51.0224	5203	10.2	31	5.1	0.0098
3	52.0307	5305	22.4	68	11.2	0.0098
4	53.0390	5408	46.8	142	23.4	0.0098
5	78.0343	7957	13.8	42	6.9	0.0098
6	78.0345	7957	6.8	21	3.4	0.0098
7	79.0421	8060	24.2	73	12.1	0.0098
8	80.0500	8162	92.0	279	46.0	0.0098
9	96.0449	9793	50.6	154	25.3	0.0098
10	124.0392	12648	199.8	606	100.0	0.0098

Cmpd 1284, AutoMSn(327.2306), 15.61 min

Compound Spectrum List Report

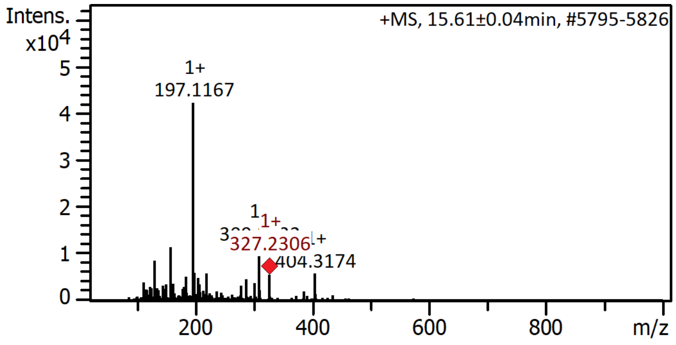

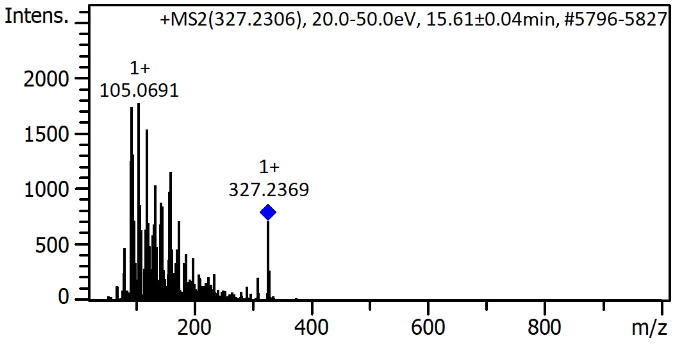

#	m/z	Res.	S/N	I	I %	FWHM
1	130.1580	10662	1435.8	8615	20.4	0.0122
2	157.0831	10579	1929.6	11578	27.4	0.0148
3	185.0412	11417	860.1	5161	12.2	0.0162
4	197.1167	11165	7032.1	42193	100.0	0.0177
5	198.1197	12191	889.3	5336	12.6	0.0163
6	199.1694	11893	985.6	5914	14.0	0.0167
7	219.0986	11789	967.7	5806	13.8	0.0186
8	309.2032	12337	1927.4	11564	27.4	0.0251
9	327.2306	12652	913.1	5478	13.0	0.0259
10	404.3174	12837	961.9	5771	13.7	0.0315

#	m/z	Res.	S/N	I	I %	FWHM
1	91.0533	9238	153.6	1255	70.9	0.0099
2	93.0692	10516	212.5	1735	98.0	0.0089
3	95.0486	9588	145.9	1191	67.3	0.0099
4	95.0846	9117	160.5	1311	74.1	0.0104
5	105.0691	8793	216.7	1770	100.0	0.0119
6	119.0843	9662	188.2	1537	86.8	0.0123
7	133.1001	9505	126.8	1036	58.5	0.0140
8	143.0832	11050	107.7	880	49.7	0.0129
9	157.1007	12040	120.0	980	55.4	0.0130
10	159.1156	13145	141.4	1155	65.2	0.0121

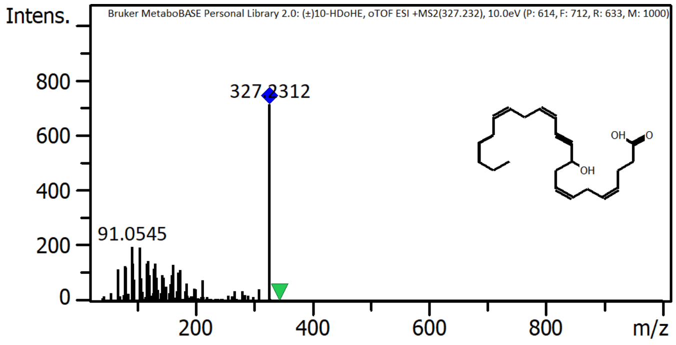

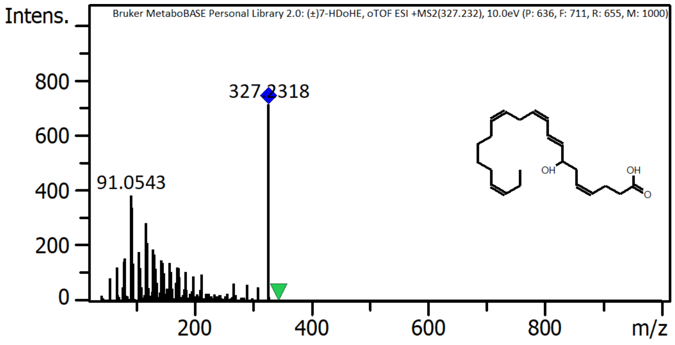

#	m/z	Res.	S/N	I	I %	FWHM
1	79.0544	8834	35.2	125	17.6	0.0089
2	81.0699	9059	33.8	120	16.9	0.0089
3	91.0545	10175	55.8	199	27.9	0.0089
4	93.0698	10400	38.8	138	19.4	0.0089
5	105.0696	11741	54.6	195	27.3	0.0089
6	117.0700	13082	38.6	138	19.3	0.0089
7	119.0858	13307	41.2	147	20.6	0.0089
8	131.0855	14648	38.4	137	19.2	0.0089
9	161.1310	18005	37.0	132	18.5	0.0089
10	327.2312	36566	199.8	712	100.0	0.0089

#	m/z	Res.	S/N	I	I %	FWHM
1	81.0699	9059	43.6	155	21.8	0.0089
2	91.0543	10175	107.8	384	54.0	0.0089
3	93.0699	10400	95.0	339	47.5	0.0089
4	105.0695	11741	50.6	180	25.3	0.0089
5	117.0698	13082	79.8	284	39.9	0.0089
6	119.0856	13307	59.6	212	29.8	0.0089
7	129.0689	14423	52.8	188	26.4	0.0089
8	131.0852	14648	47.8	170	23.9	0.0089
9	327.2318	36566	199.8	712	100.0	0.0089
10	327.2336	36566	83.2	297	41.6	0.0089

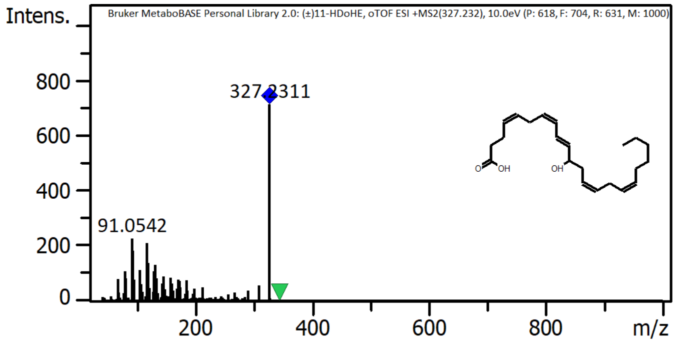

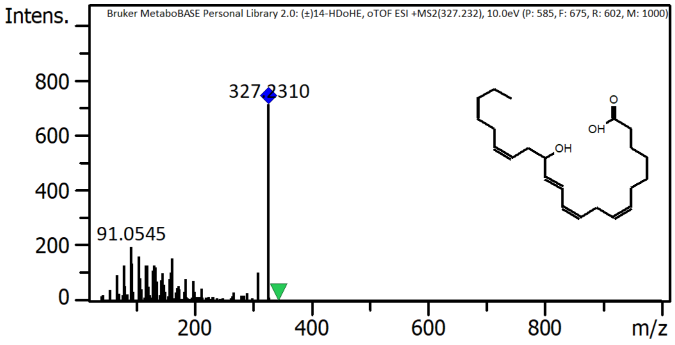

Compound Spectrum List Report

#	m/z	Res.	S/N	I	I %	FWHM
1	79.0545	8834	29.0	103	14.5	0.0089
2	79.0545	8834	30.4	108	15.2	0.0089
3	91.0542	10175	64.0	228	32.0	0.0089
4	93.0698	10400	51.6	184	25.8	0.0089
5	105.0694	11741	31.8	113	15.9	0.0089
6	117.0693	13082	59.4	212	29.7	0.0089
7	119.0849	13307	39.6	141	19.8	0.0089
8	129.0697	14423	30.4	108	15.2	0.0089
9	131.0848	14648	36.4	130	18.2	0.0089
10	327.2311	36566	199.8	712	100.0	0.0089

#	m/z	Res.	S/N	I	I %	FWHM
1	79.0549	8834	36.2	129	18.1	0.0089
2	91.0545	10175	55.6	198	27.8	0.0089
3	93.0695	10400	37.8	135	18.9	0.0089
4	105.0701	11741	45.8	163	22.9	0.0089
5	117.0696	13082	35.8	128	17.9	0.0089
6	119.0847	13307	36.2	129	18.1	0.0089
7	131.0852	14648	35.8	128	17.9	0.0089
8	133.1006	14873	34.4	123	17.2	0.0089
9	161.1318	18005	44.0	157	22.0	0.0089
10	327.2310	36566	199.8	712	100.0	0.0089

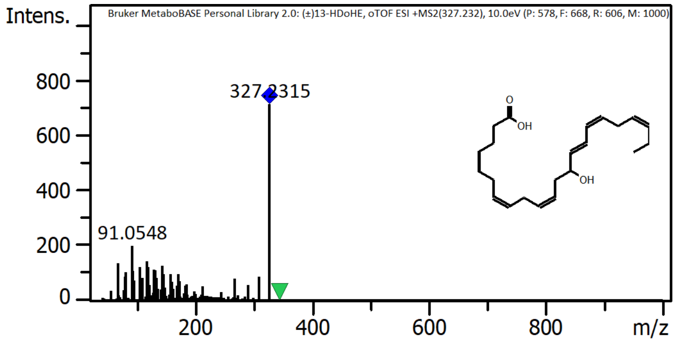

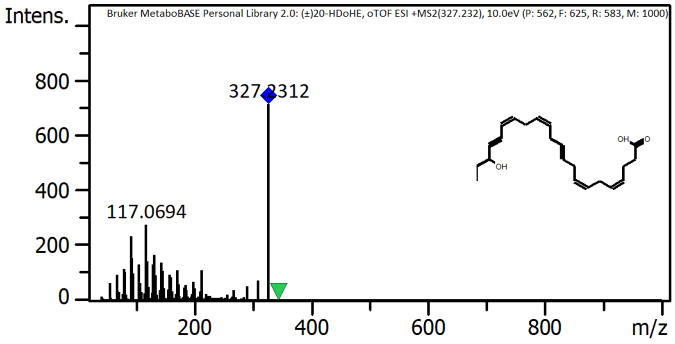

#	m/z	Res.	S/N	I	I %	FWHM
1	67.0546	7493	37.8	135	18.9	0.0089
2	91.0548	10175	56.2	200	28.1	0.0089
3	93.0697	10400	30.2	108	15.1	0.0089
4	105.0690	11741	34.2	122	17.1	0.0089
5	117.0696	13082	40.6	145	20.3	0.0089
6	119.0848	13307	34.4	123	17.2	0.0089
7	129.0697	14423	31.2	111	15.6	0.0089
8	131.0860	14648	31.0	111	15.5	0.0089
9	143.0846	15989	35.6	127	17.8	0.0089
10	327.2315	36566	199.8	712	100.0	0.0089

#	m/z	Res.	S/N	I	I %	FWHM
1	79.0545	8834	32.4	116	16.2	0.0089
2	91.0542	10175	66.0	235	33.0	0.0089
3	93.0701	10400	43.6	155	21.8	0.0089
4	105.0697	11741	36.8	131	18.4	0.0089
5	117.0694	13082	77.8	277	38.9	0.0089
6	119.0849	13307	40.4	144	20.2	0.0089
7	129.0685	14422	36.8	131	18.4	0.0089
8	131.0854	14648	47.0	168	23.5	0.0089
9	143.0852	15989	39.4	140	19.7	0.0089
10	327.2312	36566	199.8	712	100.0	0.0089

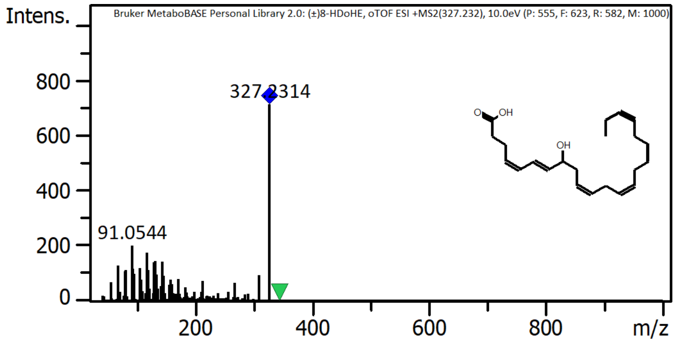

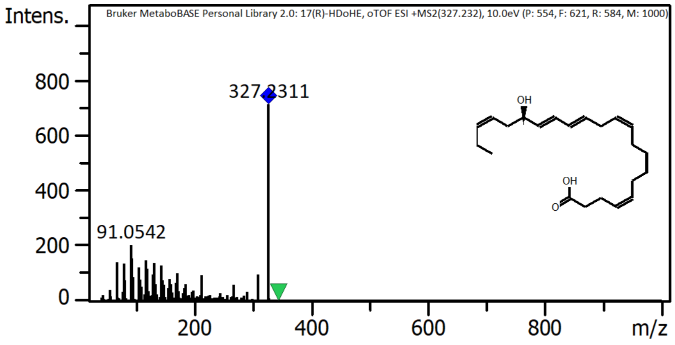

#	m/z	Res.	S/N	I	I %	FWHM
1	67.0548	7493	36.2	129	18.1	0.0089
2	81.0703	9059	31.8	113	15.9	0.0089
3	91.0544	10175	56.8	202	28.4	0.0089
4	93.0696	10400	32.8	117	16.4	0.0089
5	105.0697	11741	33.4	119	16.7	0.0089
6	117.0697	13082	49.8	178	24.9	0.0089
7	129.0691	14423	40.0	143	20.0	0.0089
8	131.0848	14648	41.0	146	20.5	0.0089
9	143.0847	15989	40.4	144	20.2	0.0089
10	327.2314	36566	199.8	712	100.0	0.0089

#	m/z	Res.	S/N	I	I %	FWHM
1	67.0546	7493	40.0	143	20.0	0.0089
2	79.0544	8834	38.8	138	19.4	0.0089
3	91.0542	10175	57.4	205	28.7	0.0089
4	93.0697	10400	43.8	156	21.9	0.0089
5	105.0697	11741	34.0	121	17.0	0.0089
6	117.0699	13082	42.0	150	21.0	0.0089
7	119.0849	13307	32.6	116	16.3	0.0089
8	131.0846	14648	39.4	140	19.7	0.0089
9	143.0846	15989	36.2	129	18.1	0.0089
10	327.2311	36566	199.8	712	100.0	0.0089

Compound Spectrum List Report

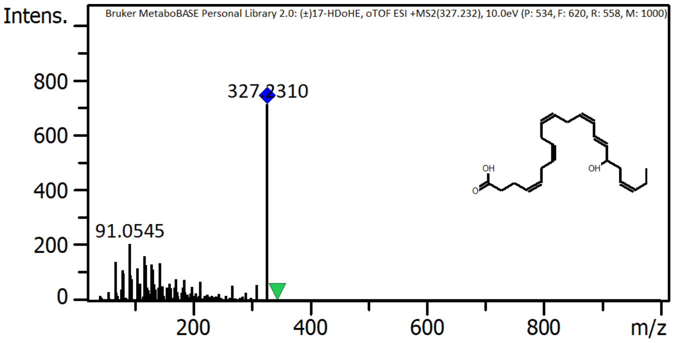

#	m/z	Res.	S/N	I	I %	FWHM
1	67.0549	7493	39.8	142	19.9	0.0089
2	79.0546	8834	30.8	110	15.4	0.0089
3	91.0545	10175	58.2	207	29.1	0.0089
4	105.0698	11741	32.6	116	16.3	0.0089
5	117.0697	13082	46.0	164	23.0	0.0089
6	119.0852	13307	36.0	128	18.0	0.0089
7	129.0693	14423	36.6	130	18.3	0.0089
8	131.0853	14648	31.2	111	15.6	0.0089
9	143.0847	15989	38.8	138	19.4	0.0089
10	327.2310	36566	199.8	712	100.0	0.0089

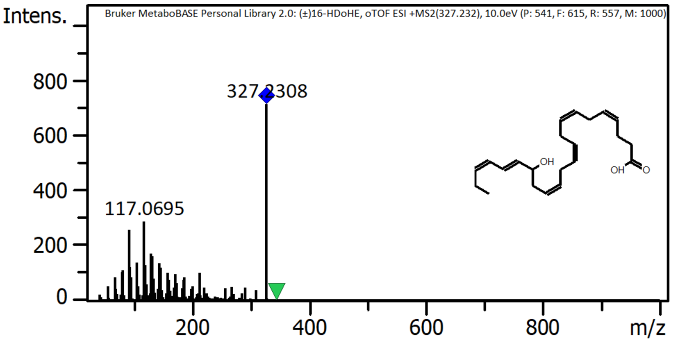

#	m/z	Res.	S/N	I	I %	FWHM
1	91.0542	10175	72.2	257	36.1	0.0089
2	93.0701	10400	34.2	122	17.1	0.0089
3	105.0691	11741	39.2	140	19.6	0.0089
4	117.0695	13082	81.2	289	40.6	0.0089
5	119.0848	13307	36.2	129	18.1	0.0089
6	129.0693	14423	48.6	173	24.3	0.0089
7	131.0850	14648	45.6	163	22.8	0.0089
8	143.0851	15989	38.4	137	19.2	0.0089
9	145.1009	16214	33.6	120	16.8	0.0089
10	327.2308	36566	199.8	712	100.0	0.0089

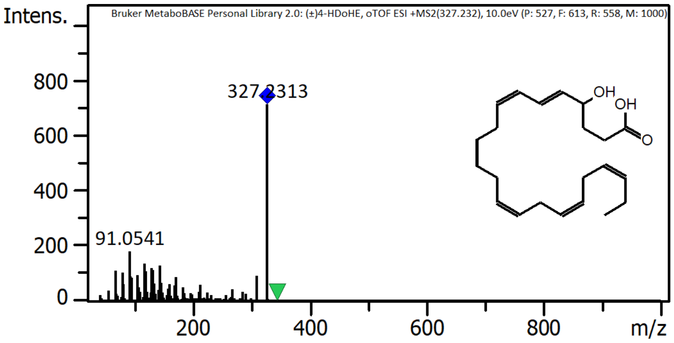

#	m/z	Res.	S/N	I	I %	FWHM
1	67.0545	7493	28.8	103	14.4	0.0089
2	67.0546	7493	30.6	109	15.3	0.0089
3	79.0544	8834	29.0	103	14.5	0.0089
4	91.0541	10175	50.8	181	25.4	0.0089
5	117.0692	13082	38.2	136	19.1	0.0089
6	119.0851	13307	30.2	108	15.1	0.0089
7	129.0696	14423	33.6	120	16.8	0.0089
8	131.0850	14648	31.6	113	15.8	0.0089
9	143.0844	15989	36.0	128	18.0	0.0089
10	327.2313	36566	199.8	712	100.0	0.0089

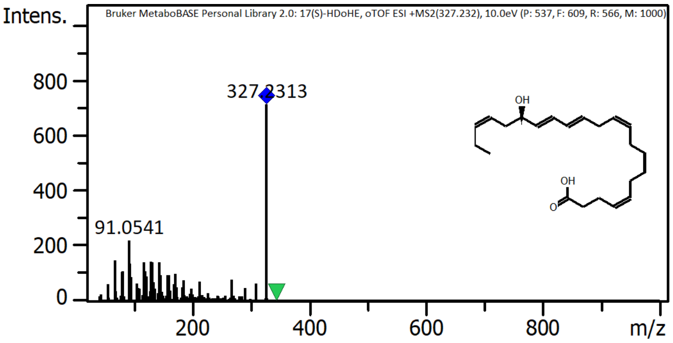

#	m/z	Res.	S/N	I	I %	FWHM
1	67.0543	7493	41.6	148	20.8	0.0089
2	81.0702	9059	30.4	108	15.2	0.0089
3	91.0539	10175	40.4	144	20.2	0.0089
4	91.0541	10175	62.0	221	31.0	0.0089
5	93.0699	10400	38.6	138	19.3	0.0089
6	117.0698	13082	40.0	143	20.0	0.0089
7	129.0692	14423	40.6	145	20.3	0.0089
8	131.0848	14648	40.0	143	20.0	0.0089
9	143.0844	15989	40.0	143	20.0	0.0089
10	327.2313	36566	199.8	712	100.0	0.0089

Cmpd 1347, AutoMSn(455.3507), 16.59 min

Compound Spectrum List Report

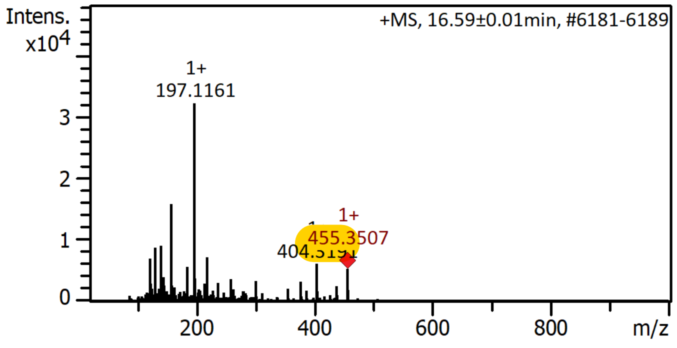

#	m/z	Res.	S/N	I	I %	FWHM
1	121.0282	11112	585.3	7024	21.8	0.0109
2	130.1581	10748	732.4	8789	27.3	0.0121
3	139.0377	10209	756.3	9076	28.2	0.0136
4	144.1017	10243	323.8	3885	12.1	0.0141
5	157.0827	10920	1321.1	15853	49.2	0.0144
6	185.0422	11828	461.1	5533	17.2	0.0156
7	197.1161	10999	2684.9	32219	100.0	0.0179
8	219.0988	10983	599.6	7195	22.3	0.0199
9	404.3191	14619	508.5	6102	18.9	0.0277
10	455.3507	11541	435.7	5229	16.2	0.0395

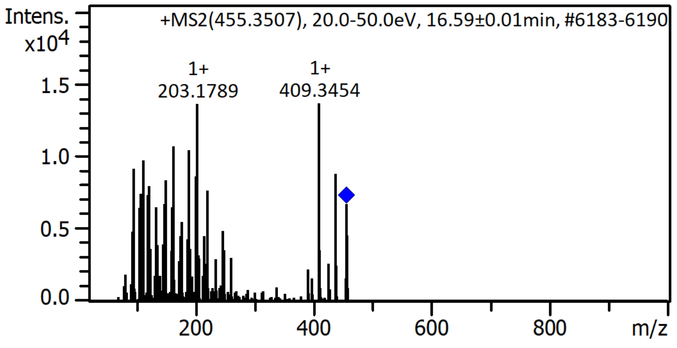

#	m/z	Res.	S/N	I	I %	FWHM
1	95.0850	8513	562.2	9183	67.2	0.0112
2	111.0796	9759	595.3	9723	71.2	0.0114
3	121.1001	10372	486.7	7949	58.2	0.0117
4	149.1321	9823	511.3	8352	61.1	0.0152
5	163.1470	10801	656.6	10724	78.5	0.0151
6	189.1626	10548	640.1	10455	76.5	0.0179
7	201.1627	11768	528.0	8624	63.1	0.0171
8	203.1789	11930	834.4	13628	99.7	0.0170
9	409.3454	12960	836.6	13665	100.0	0.0316
10	437.3378	12280	538.7	8798	64.4	0.0356

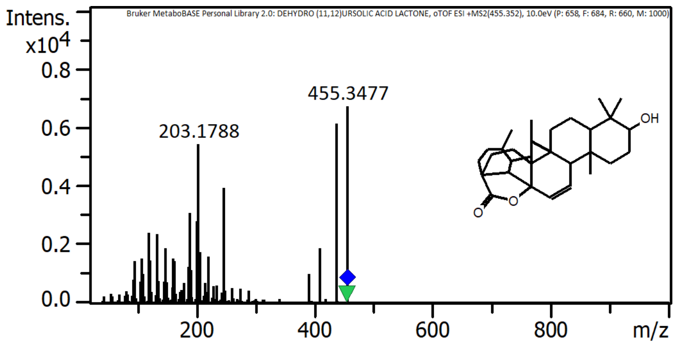

#	m/z	Res.	S/N	I	I %	FWHM
1	119.0855	3319	72.2	2425	36.1	0.0359
2	133.1012	3710	70.4	2365	35.2	0.0359
3	189.1629	5272	92.0	3091	46.0	0.0359
4	201.1636	5607	83.6	2808	41.8	0.0359
5	203.1788	5663	162.0	5442	81.1	0.0359
6	247.1660	6889	117.6	3951	58.9	0.0359
7	409.3431	11409	56.6	1901	28.3	0.0359
8	437.3370	12189	182.4	6127	91.3	0.0359
9	437.3400	12189	56.4	1895	28.2	0.0359
10	455.3477	12691	199.8	6712	100.0	0.0359

Cmpd 1378, AutoMSn(279.2306), 17.18 min

Compound Spectrum List Report

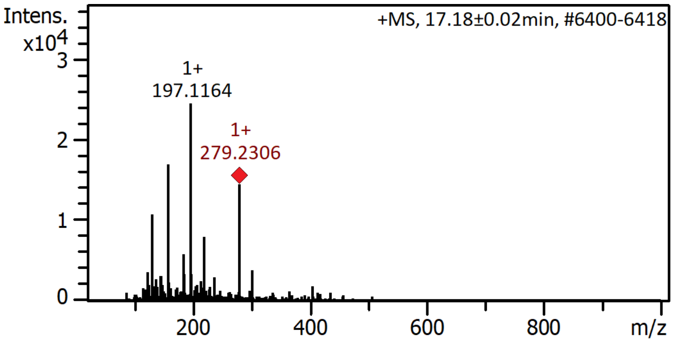

#	m/z	Res.	S/N	I	I %	FWHM
1	121.9654	10265	584.0	3504	14.3	0.0119
2	130.1585	10566	1790.9	10746	43.9	0.0123
3	157.0831	10766	2817.9	16907	69.1	0.0146
4	185.0414	10759	975.9	5855	23.9	0.0172
5	185.1137	10632	545.8	3275	13.4	0.0174
6	197.1164	11737	4079.0	24474	100.0	0.0168
7	198.1191	11999	543.3	3260	13.3	0.0165
8	219.0980	11316	1324.5	7947	32.5	0.0194
9	279.2306	12216	2410.6	14464	59.1	0.0229
10	301.1388	12014	624.7	3748	15.3	0.0251

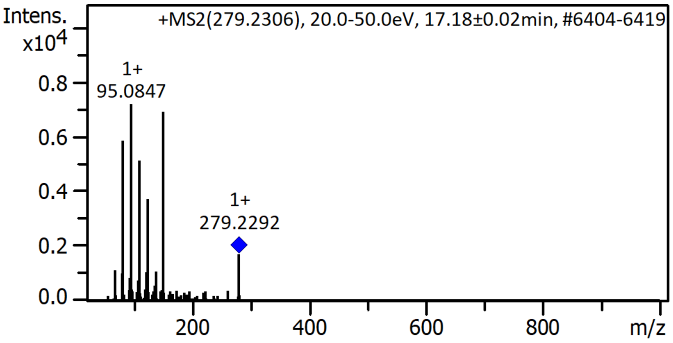

#	m/z	Res.	S/N	I	I %	FWHM
1	67.0539	8277	139.1	1113	15.5	0.0081
2	79.0540	7354	125.2	1002	13.9	0.0107
3	81.0688	8766	731.6	5853	81.3	0.0092
4	95.0847	9751	899.8	7198	100.0	0.0098
5	109.0998	9919	642.8	5142	71.4	0.0110
6	121.0294	8598	129.0	1032	14.3	0.0141
7	123.1156	10562	464.6	3717	51.6	0.0117
8	137.1321	10409	131.6	1053	14.6	0.0132
9	149.0227	10398	864.3	6914	96.1	0.0143
10	279.2292	11912	214.7	1718	23.9	0.0234

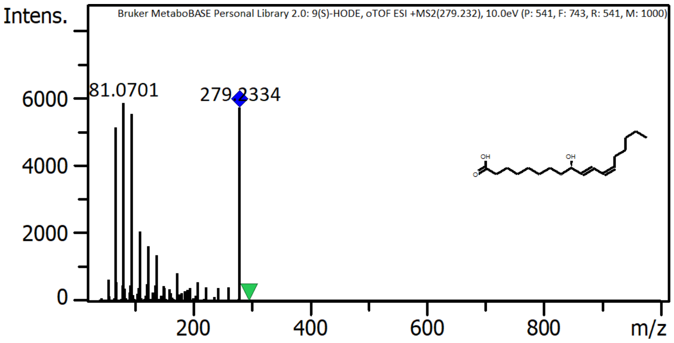

#	m/z	Res.	S/N	I	I %	FWHM
1	55.0550	5884	21.6	632	10.8	0.0094
2	67.0548	7167	175.6	5139	87.9	0.0094
3	69.0700	7382	18.8	550	9.4	0.0094
4	81.0701	8665	199.8	5847	100.0	0.0094
5	95.0855	10163	188.6	5519	94.4	0.0094
6	109.1006	11661	71.0	2078	35.5	0.0094
7	123.1165	13159	56.0	1639	28.0	0.0094
8	137.1314	14657	46.6	1364	23.3	0.0094
9	173.1314	18505	28.2	825	14.1	0.0094
10	279.2334	29845	195.2	5713	97.7	0.0094

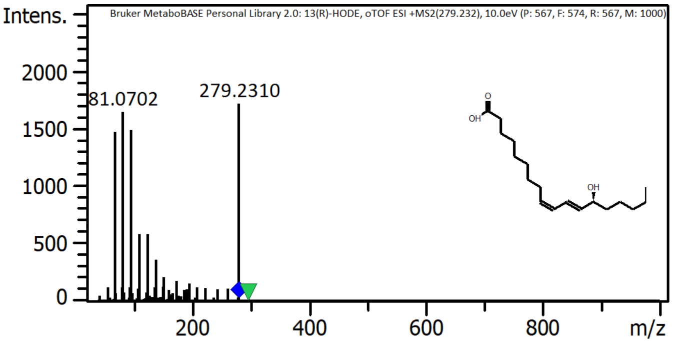

#	m/z	Res.	S/N	I	I %	FWHM
1	67.0546	7167	171.6	1474	85.9	0.0094
2	81.0702	8665	191.4	1644	95.8	0.0094
3	95.0854	10163	173.2	1487	86.7	0.0094
4	109.1003	11661	68.2	586	34.1	0.0094
5	123.1167	13159	68.6	589	34.3	0.0094
6	137.1317	14657	42.6	366	21.3	0.0094
7	151.1465	16155	24.6	211	12.3	0.0094
8	173.1313	18505	20.4	175	10.2	0.0094
9	195.1353	20857	17.6	151	8.8	0.0094
10	279.2310	29845	199.8	1716	100.0	0.0094

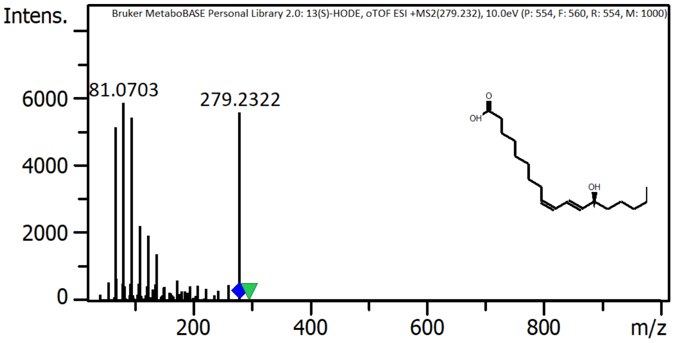

Compound Spectrum List Report

#	m/z	Res.	S/N	I	I %	FWHM
1	55.0546	5884	18.2	533	9.1	0.0094
2	67.0548	7167	175.6	5139	87.9	0.0094
3	69.0705	7382	22.0	644	11.0	0.0094
4	81.0703	8665	199.8	5847	100.0	0.0094
5	95.0856	10163	184.8	5408	92.5	0.0094
6	109.1011	11661	75.8	2218	37.9	0.0094
7	123.1164	13159	66.4	1943	33.2	0.0094
8	137.1321	14657	47.6	1393	23.8	0.0094
9	173.1332	18505	20.6	603	10.3	0.0094
10	279.2322	29845	190.0	5560	95.1	0.0094

Cmpd 1386, AutoMSn(331.2837), 17.37 min

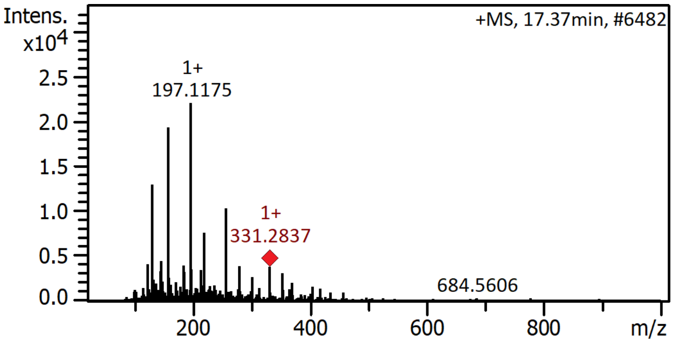

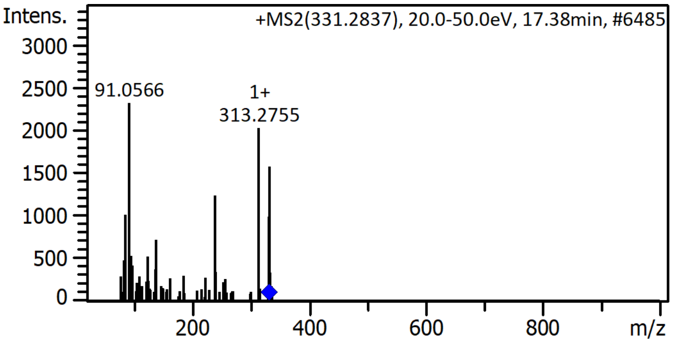

#	m/z	Res.	S/N	I	I %	FWHM
1	121.9646	10242	113.8	4097	18.6	0.0119
2	130.1581	10811	361.6	13018	59.1	0.0120
3	144.9845	10088	125.7	4525	20.6	0.0144
4	157.0839	9996	537.3	19341	87.8	0.0157
5	185.0418	14070	110.5	3979	18.1	0.0132
6	197.1175	10509	611.6	22016	100.0	0.0188
7	219.0998	14005	212.7	7657	34.8	0.0156
8	256.2626	12566	288.4	10383	47.2	0.0204
9	279.2306	15817	107.9	3885	17.6	0.0177
10	331.2837	15730	105.6	3803	17.3	0.0211

#	m/z	Res.	S/N	I	I %	FWHM
1	83.0881	8845	9.5	482	20.8	0.0094
2	85.1022	9134	20.0	1018	44.0	0.0093
3	91.0566	8229	45.4	2315	100.0	0.0111
4	95.0859	11826	10.5	533	23.0	0.0080
5	123.1220	5743	10.4	531	22.9	0.0214
6	137.1330	11439	14.2	726	31.4	0.0120
7	239.2325	12004	24.3	1239	53.5	0.0199
8	313.2755	13278	39.7	2027	87.6	0.0236
9	331.2088	15323	19.4	989	42.7	0.0216
10	332.3360	14905	30.8	1573	67.9	0.0223

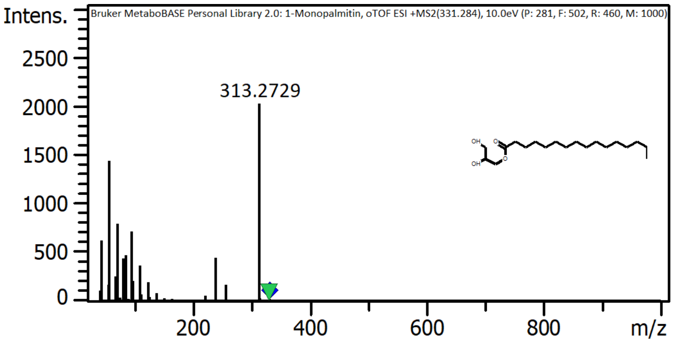

#	m/z	Res.	S/N	I	I %	FWHM
1	43.0545	7306	51.3	624	30.8	0.0059
2	57.0698	9684	118.3	1439	71.1	0.0059
3	71.0853	12063	65.7	799	39.4	0.0059
4	81.0695	13757	36.3	442	21.8	0.0059
5	83.0852	14099	31.7	385	19.0	0.0059
6	85.1007	14441	39.2	476	23.5	0.0059
7	95.0850	16135	59.0	718	35.4	0.0059
8	109.1007	18514	30.0	365	18.0	0.0059
9	239.2372	40597	37.2	452	22.3	0.0059
10	313.2729	53160	166.5	2025	100.0	0.0059

Compound Spectrum List Report

Cmpd 1410, AutoMSn(455.3507), 17.80 min

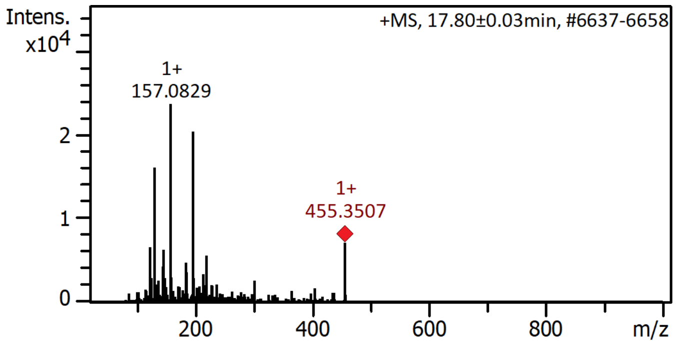

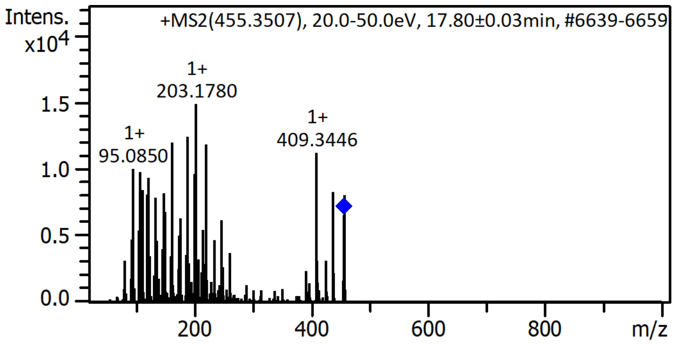

#	m/z	Res.	S/N	I	I %	FWHM
1	121.9648	9916	912.8	6572	27.8	0.0123
2	130.1576	9191	2233.4	16080	68.0	0.0142
3	144.1012	9124	591.1	4256	18.0	0.0158
4	144.9813	9947	879.3	6331	26.8	0.0146
5	157.0829	9722	3282.9	23637	100.0	0.0162
6	185.0417	10790	664.0	4781	20.2	0.0171
7	185.1146	8640	490.4	3531	14.9	0.0214
8	197.1162	9650	2832.3	20392	86.3	0.0204
9	219.0987	9797	779.9	5616	23.8	0.0224
10	455.3507	9386	987.1	7107	30.1	0.0485

#	m/z	Res.	S/N	I	I %	FWHM
1	95.0850	8898	1020.4	10000	67.3	0.0107
2	107.0846	8938	998.8	9788	65.9	0.0120
3	111.0801	9437	862.4	8452	56.9	0.0118
4	121.1007	9332	954.3	9352	62.9	0.0130
5	163.1471	9900	1223.9	11994	80.7	0.0165
6	189.1631	10516	1269.8	12444	83.8	0.0180
7	201.1627	10243	983.7	9641	64.9	0.0196
8	203.1780	10457	1516.1	14857	100.0	0.0194
9	221.1880	10417	1210.4	11862	79.8	0.0212
10	409.3446	10294	1142.7	11198	75.4	0.0398

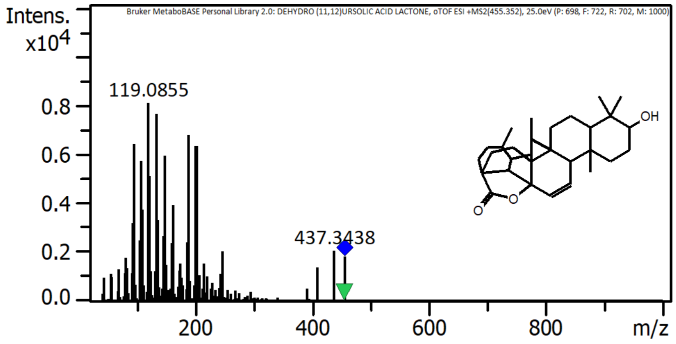

#	m/z	Res.	S/N	I	I %	FWHM
1	95.0855	4767	143.8	5832	72.0	0.0199
2	95.0856	4767	158.8	6441	79.5	0.0199
3	107.0848	5368	141.6	5743	70.9	0.0199
4	119.0855	5970	199.8	8104	100.0	0.0199
5	121.1019	6071	126.4	5127	63.3	0.0199
6	133.1011	6673	189.2	7674	94.7	0.0199
7	147.1168	7375	146.8	5954	73.5	0.0199
8	189.1624	9483	167.6	6798	83.9	0.0199
9	201.1637	10085	156.6	6352	78.4	0.0199
10	203.1793	10186	156.4	6343	78.3	0.0199

Cmpd 1415, AutoMSn(281.2459), 17.99 min

Compound Spectrum List Report

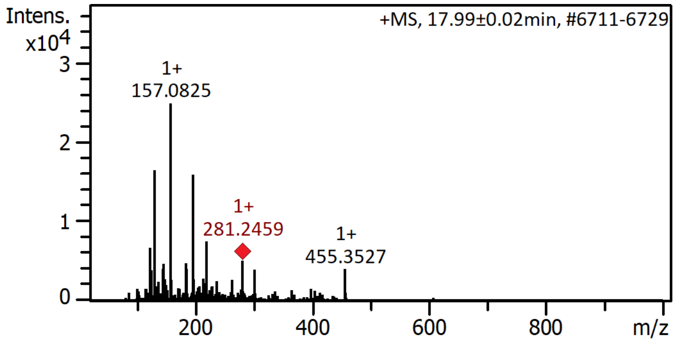

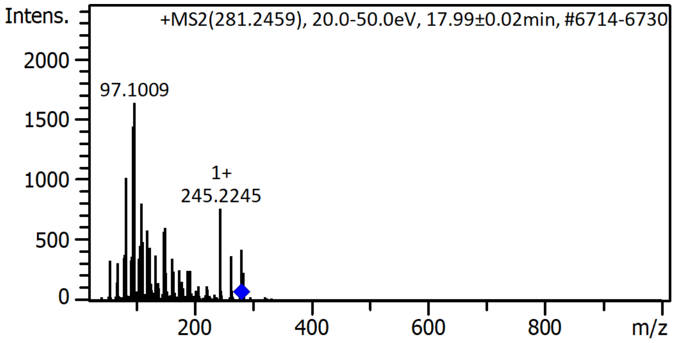

#	m/z	Res.	S/N	I	I %	FWHM
1	121.9645	9437	937.6	6751	27.1	0.0129
2	130.1583	9643	2282.1	16431	66.1	0.0135
3	144.9812	10528	641.8	4621	18.6	0.0138
4	157.0825	10108	3453.6	24866	100.0	0.0155
5	185.0406	10577	658.5	4741	19.1	0.0175
6	185.1131	11548	556.6	4008	16.1	0.0160
7	197.1168	10740	2206.5	15887	63.9	0.0184
8	219.0966	10052	1086.0	7819	31.4	0.0218
9	281.2459	12970	706.1	5084	20.4	0.0217
10	455.3527	8545	551.3	3969	16.0	0.0533

#	m/z	Res.	S/N	I	I %	FWHM
1	83.0845	7778	92.3	1015	62.1	0.0107
2	95.0843	10040	131.1	1442	88.3	0.0095
3	97.1009	8974	148.5	1633	100.0	0.0108
4	107.0849	11603	41.5	456	27.9	0.0092
5	109.1010	10295	73.1	804	49.2	0.0106
6	111.1156	11957	44.3	487	29.8	0.0093
7	119.0828	11124	53.1	584	35.8	0.0107
8	147.1185	8414	52.3	575	35.2	0.0175
9	149.0235	11086	54.8	603	36.9	0.0134
10	245.2245	11896	69.5	765	46.8	0.0206

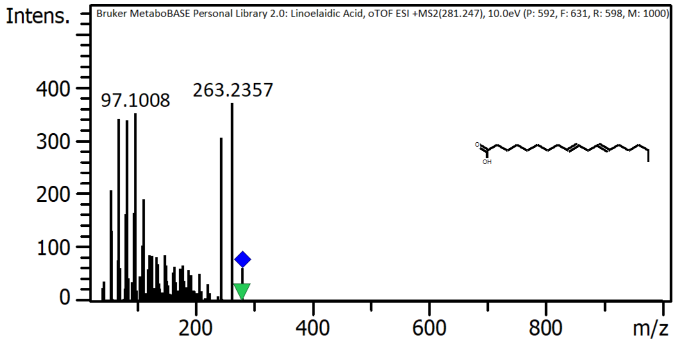

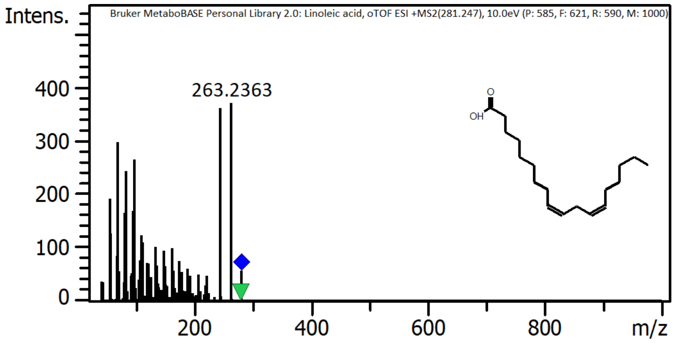

#	m/z	Res.	S/N	I	I %	FWHM
1	55.0551	6664	112.2	208	56.2	0.0083
2	57.0700	6908	71.6	133	35.8	0.0083
3	69.0704	8361	183.6	340	91.9	0.0083
4	81.0702	9813	88.0	163	44.0	0.0083
5	83.0855	10057	182.4	338	91.3	0.0083
6	95.0855	11510	89.2	165	44.6	0.0083
7	97.1008	11754	189.8	352	95.0	0.0083
8	111.1165	13450	103.2	191	51.7	0.0083
9	245.2259	29684	165.2	306	82.7	0.0083
10	263.2357	31864	199.8	370	100.0	0.0083

#	m/z	Res.	S/N	I	I %	FWHM
1	55.0540	6664	104.0	193	52.1	0.0083
2	57.0695	6908	68.6	127	34.3	0.0083
3	69.0694	8361	160.4	297	80.3	0.0083
4	81.0694	9813	89.8	166	44.9	0.0083
5	83.0853	10057	131.8	244	66.0	0.0083
6	95.0849	11510	91.6	170	45.8	0.0083
7	97.1002	11754	143.2	265	71.7	0.0083
8	109.1006	13206	66.8	124	33.4	0.0083
9	245.2255	29684	195.0	361	97.6	0.0083
10	263.2363	31864	199.8	370	100.0	0.0083

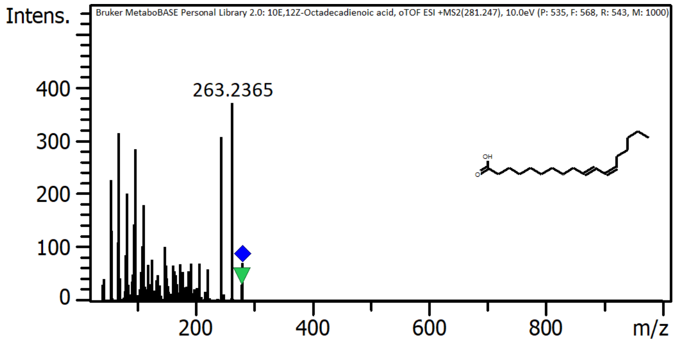

Compound Spectrum List Report

#	m/z	Res.	S/N	I	I %	FWHM
1	55.0545	6664	87.6	162	43.8	0.0083
2	55.0546	6664	122.6	227	61.4	0.0083
3	57.0702	6908	71.4	132	35.7	0.0083
4	69.0704	8361	169.6	314	84.9	0.0083
5	83.0865	10057	108.6	201	54.4	0.0083
6	95.0861	11510	78.2	145	39.1	0.0083
7	97.1003	11754	153.8	285	77.0	0.0083
8	111.1168	13450	97.2	180	48.6	0.0083
9	245.2280	29684	165.8	307	83.0	0.0083
10	263.2365	31864	199.8	370	100.0	0.0083

Cmpd 1441, AutoMSn(391.2828), 18.52 min

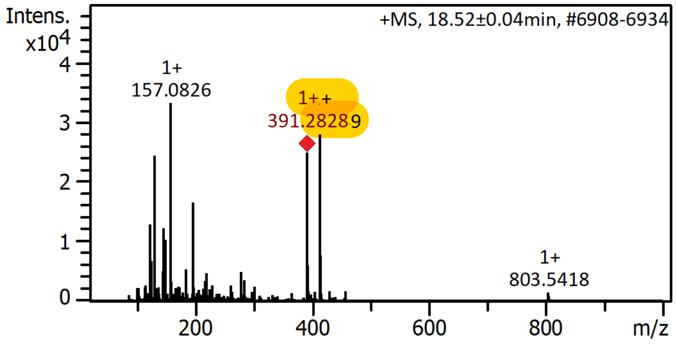

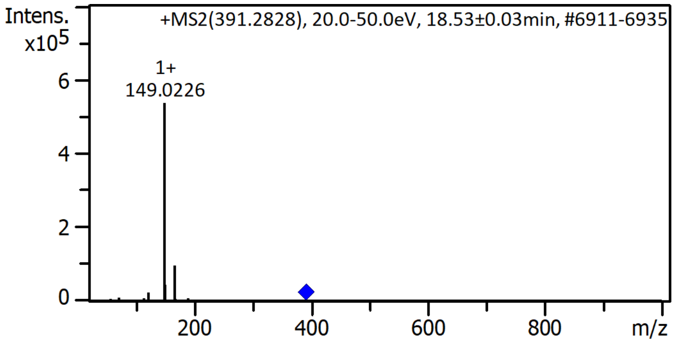

#	m/z	Res.	S/N	I	I %	FWHM
1	121.9651	10576	1801.5	12971	39.1	0.0115
2	130.1580	10354	3397.5	24462	73.7	0.0126
3	144.9813	11005	1707.4	12294	37.0	0.0132
4	146.9790	11526	985.0	7092	21.4	0.0128
5	149.0223	10815	1433.4	10320	31.1	0.0138
6	157.0826	10895	4613.0	33214	100.0	0.0144
7	197.1160	11479	2307.5	16614	50.0	0.0172
8	391.2828	12786	3472.0	24999	75.3	0.0306
9	413.2649	12260	3878.8	27927	84.1	0.0337
10	414.2683	12433	1086.9	7826	23.6	0.0333

#	m/z	Res.	S/N	I	I %	FWHM
1	57.0700	7109	587.4	5639	1.1	0.0080
2	71.0857	7008	1055.4	10131	1.9	0.0101
3	113.1317	9682	844.1	8104	1.5	0.0117
4	121.0281	9802	2445.5	23477	4.4	0.0123
5	149.0226	9648	55856.3	536221	100.0	0.0154
6	150.0262	11261	4521.0	43402	8.1	0.0133
7	151.0281	10522	338.4	3249	0.6	0.0144
8	167.0334	10779	10053.0	96509	18.0	0.0155
9	168.0371	10507	671.7	6448	1.2	0.0160
10	190.0494	11725	752.7	7226	1.3	0.0162

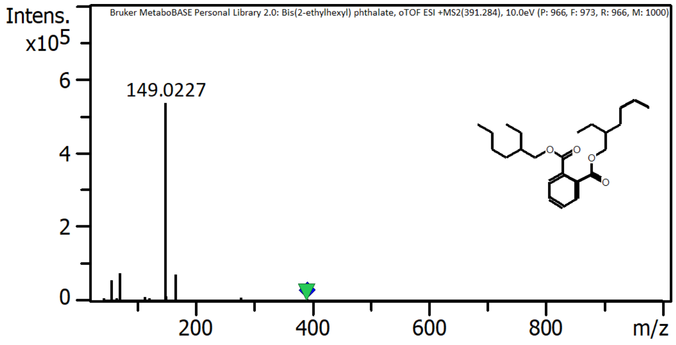

#	m/z	Res.	S/N	I	I %	FWHM
1	43.0545	2863	2.5	8043	1.5	0.0150
2	57.0704	3795	17.5	56303	10.5	0.0150
3	65.0384	4325	2.3	7507	1.4	0.0150
4	71.0856	4727	23.7	76143	14.2	0.0150
5	113.1324	7523	3.7	11797	2.2	0.0150
6	121.0266	8048	2.3	7507	1.4	0.0150
7	149.0227	9910	166.5	535684	100.0	0.0150
8	149.1343	9917	4.2	13406	2.5	0.0150
9	167.0338	11107	22.3	71854	13.4	0.0150
10	279.1597	18563	2.8	9116	1.7	0.0150

Compound Spectrum List Report

Cmpd 1457, AutoMSn(118.0846), 18.73 min

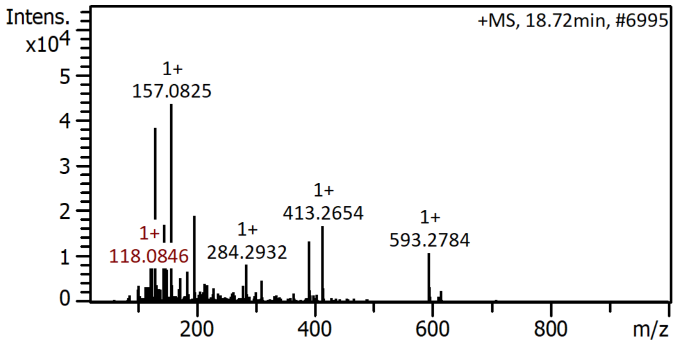

#	m/z	Res.	S/N	I	I %	FWHM
1	121.9651	10865	460.1	16562	38.0	0.0112
2	123.9633	10412	236.3	8508	19.5	0.0119
3	130.1574	10148	1063.3	38277	87.9	0.0128
4	144.9811	12006	473.6	17050	39.2	0.0121
5	146.9793	11340	283.9	10222	23.5	0.0130
6	157.0825	11108	1209.1	43528	100.0	0.0141
7	197.1170	12035	529.1	19047	43.8	0.0164
8	391.2828	13361	371.6	13379	30.7	0.0293
9	413.2654	13432	467.9	16843	38.7	0.0308
10	593.2784	13914	303.5	10925	25.1	0.0426

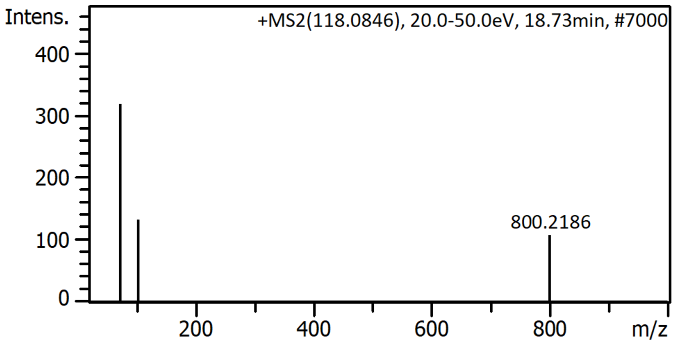

#	m/z	Res.	S/N	I	I %	FWHM
1	72.0816	11618	2.9	318	100.0	0.0062
2	103.0490	13081	1.2	133	41.8	0.0079
3	800.2186	41175	1.0	108	34.0	0.0194

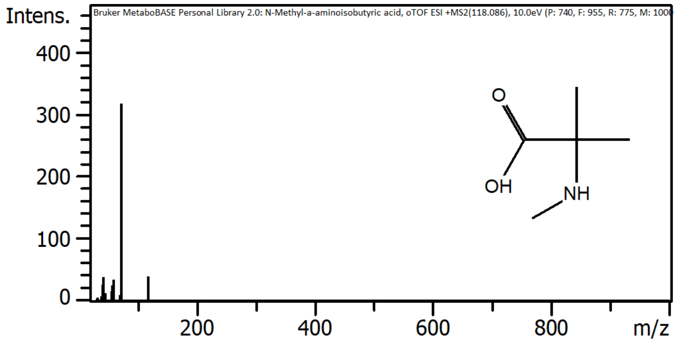

#	m/z	Res.	S/N	I	I %	FWHM
1	41.0391	10064	11.7	26	8.2	0.0041
2	42.0346	10308	17.3	38	12.1	0.0041
3	43.0185	10549	3.6	8	2.5	0.0041
4	45.0340	11044	5.6	12	3.9	0.0041
5	56.0494	13745	7.1	16	5.0	0.0041
6	57.0578	13992	11.3	25	7.9	0.0041
7	59.0497	14481	15.3	34	10.7	0.0041
8	70.0648	17182	4.6	10	3.2	0.0041
9	72.0812	17676	142.7	318	100.0	0.0041
10	118.0865	28958	17.9	40	12.5	0.0041

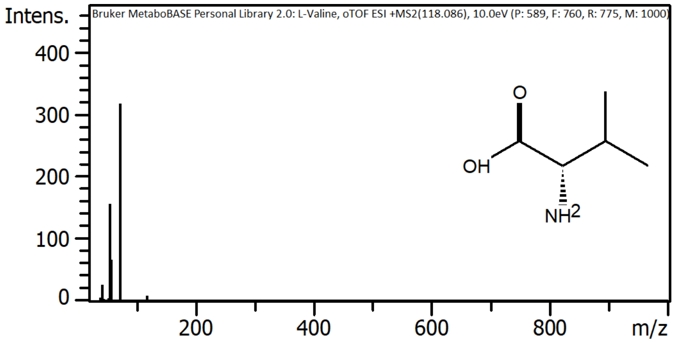

#	m/z	Res.	S/N	I	I %	FWHM
1	39.0231	9570	3.0	6	1.8	0.0041
2	41.0393	10064	1.7	3	1.0	0.0041
3	42.0336	10308	14.0	27	8.4	0.0041
4	44.0514	10803	1.8	3	1.1	0.0041
5	53.0392	13007	2.7	5	1.6	0.0041
6	55.0546	13501	82.0	156	49.2	0.0041
7	56.0504	13745	23.5	45	14.1	0.0041
8	57.0579	13992	35.5	68	21.3	0.0041
9	72.0815	17677	166.5	318	100.0	0.0041
10	118.0864	28958	4.8	9	2.9	0.0041

Cmpd 1465, AutoMSn(391.2817), 18.81 min

Compound Spectrum List Report

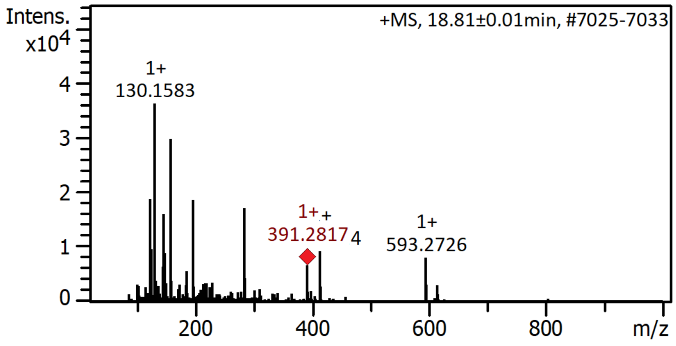

#	m/z	Res.	S/N	I	I %	FWHM
1	121.9657	10467	1570.7	18848	52.0	0.0117
2	123.9636	10521	800.3	9604	26.5	0.0118
3	130.1583	10589	3019.1	36230	100.0	0.0123
4	144.9816	10905	1342.8	16113	44.5	0.0133
5	146.9795	10533	747.0	8964	24.7	0.0140
6	157.0826	10957	2478.7	29745	82.1	0.0143
7	197.1157	12052	1558.7	18704	51.6	0.0164
8	284.2932	12930	1432.8	17194	47.5	0.0220
9	413.2624	13624	771.6	9259	25.6	0.0303
10	593.2726	13836	677.7	8132	22.4	0.0429

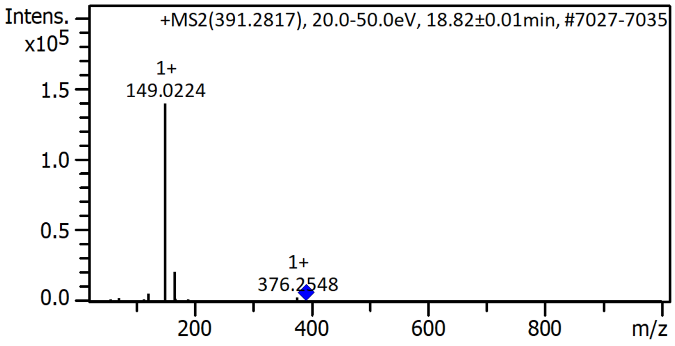

#	m/z	Res.	S/N	I	I %	FWHM
1	57.0705	6135	90.3	1475	1.1	0.0093
2	71.0852	7362	156.9	2562	1.8	0.0097
3	113.1316	9381	88.1	1439	1.0	0.0121
4	121.0277	9830	348.0	5684	4.1	0.0123
5	149.0224	10675	8526.2	139261	100.0	0.0140
6	150.0261	10501	663.0	10829	7.8	0.0143
7	167.0328	11079	1295.4	21158	15.2	0.0151
8	168.0365	10601	131.0	2140	1.5	0.0159
9	190.0493	10818	96.5	1577	1.1	0.0176
10	376.2548	15968	179.6	2933	2.1	0.0236

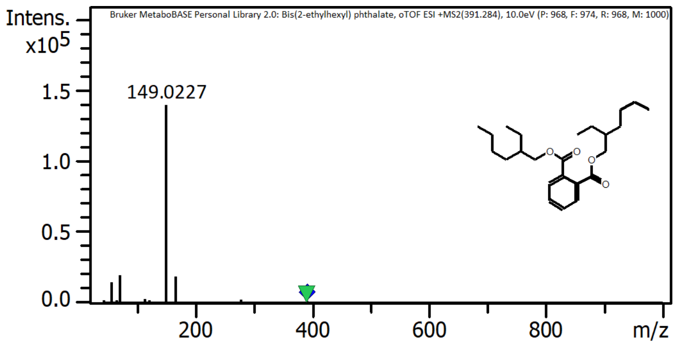

#	m/z	Res.	S/N	I	I %	FWHM
1	43.0545	3087	2.5	2089	1.5	0.0139
2	57.0704	4092	17.5	14622	10.5	0.0139
3	65.0384	4663	2.3	1950	1.4	0.0139
4	71.0856	5097	23.7	19775	14.2	0.0139
5	113.1324	8112	3.7	3064	2.2	0.0139
6	121.0266	8678	2.3	1950	1.4	0.0139
7	149.0227	10685	166.5	139122	100.0	0.0139
8	149.1343	10693	4.2	3482	2.5	0.0139
9	167.0338	11977	22.3	18661	13.4	0.0139
10	279.1597	20017	2.8	2367	1.7	0.0139

Cmpd 1495, AutoMSn(391.2832), 19.25 min

Compound Spectrum List Report

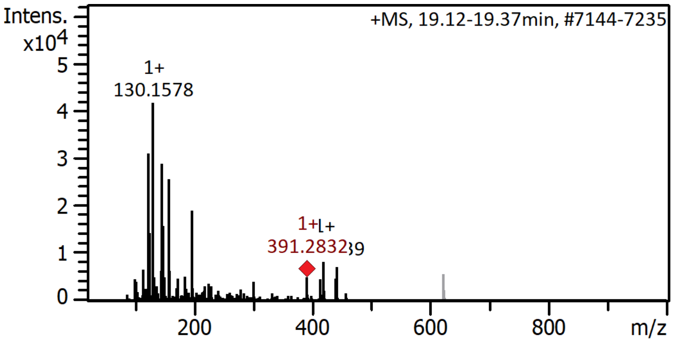

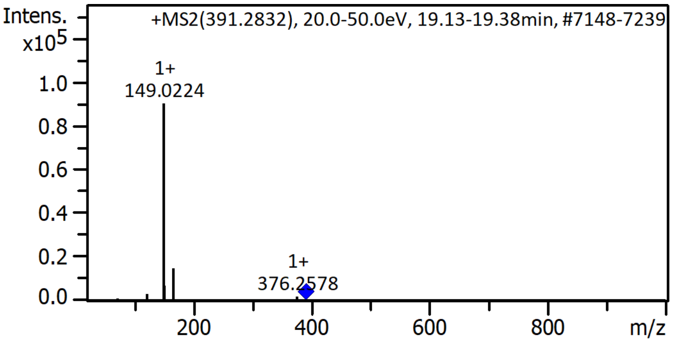

#	m/z	Res.	S/N	I	I %	FWHM
1	114.0905	9819	723.2	6509	15.6	0.0116
2	121.9648	10036	3445.0	31005	74.5	0.0122
3	123.9632	9641	1596.1	14365	34.5	0.0129
4	130.1578	10230	4624.5	41621	100.0	0.0127
5	144.9809	10593	3202.0	28818	69.2	0.0137
6	146.9792	10302	1757.1	15814	38.0	0.0143
7	157.0821	10857	2851.8	25667	61.7	0.0145
8	197.1164	11409	2122.6	19104	45.9	0.0173
9	419.3139	12275	923.4	8311	20.0	0.0342
10	441.2959	13315	779.9	7019	16.9	0.0331

#	m/z	Res.	S/N	I	I %	FWHM
1	57.0711	7525	67.1	822	0.9	0.0076
2	71.0845	8047	85.2	1044	1.2	0.0088
3	113.1313	10437	66.7	817	0.9	0.0108
4	121.0280	11230	259.7	3181	3.5	0.0108
5	149.0224	10912	7362.4	90189	100.0	0.0137
6	150.0253	12170	561.3	6876	7.6	0.0123
7	151.0279	9997	39.4	483	0.5	0.0151
8	167.0334	10882	1212.7	14855	16.5	0.0153
9	190.0469	14147	55.1	675	0.7	0.0134
10	376.2578	12911	163.5	2003	2.2	0.0291

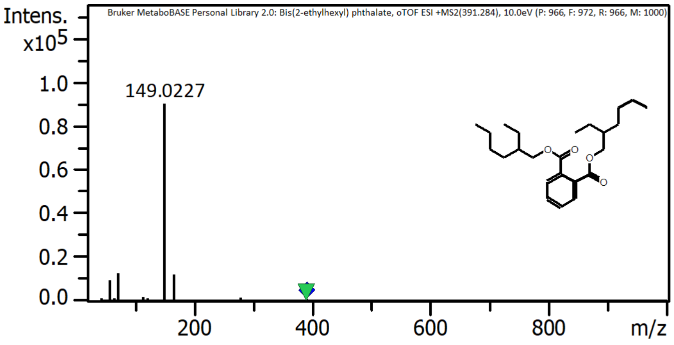

#	m/z	Res.	S/N	I	I %	FWHM
1	43.0545	3176	2.5	1353	1.5	0.0136
2	57.0704	4210	17.5	9470	10.5	0.0136
3	65.0384	4797	2.3	1263	1.4	0.0136
4	71.0856	5243	23.7	12807	14.2	0.0136
5	113.1324	8345	3.7	1984	2.2	0.0136
6	121.0266	8927	2.3	1263	1.4	0.0136
7	149.0227	10992	166.5	90099	100.0	0.0136
8	149.1343	11000	4.2	2255	2.5	0.0136
9	167.0338	12320	22.3	12085	13.4	0.0136
10	279.1597	20591	2.8	1533	1.7	0.0136

Cmpd 1540, AutoMSn(338.3405), 20.02 min

Compound Spectrum List Report

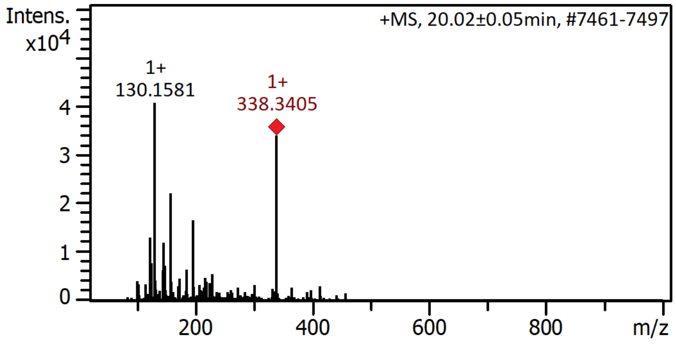

#	m/z	Res.	S/N	I	I %	FWHM
1	121.9654	10675	2174.7	13048	32.1	0.0114
2	123.9637	10654	1272.2	7633	18.8	0.0116
3	130.1581	10524	6775.2	40651	100.0	0.0124
4	144.9816	11086	2013.9	12083	29.7	0.0131
5	146.9795	11527	1188.4	7130	17.5	0.0128
6	157.0825	11131	3698.1	22188	54.6	0.0141
7	185.1143	12314	1072.3	6434	15.8	0.0150
8	197.1163	11109	2764.4	16586	40.8	0.0177
9	338.3405	12578	5664.7	33988	83.6	0.0269
10	339.3426	14392	1244.4	7467	18.4	0.0236

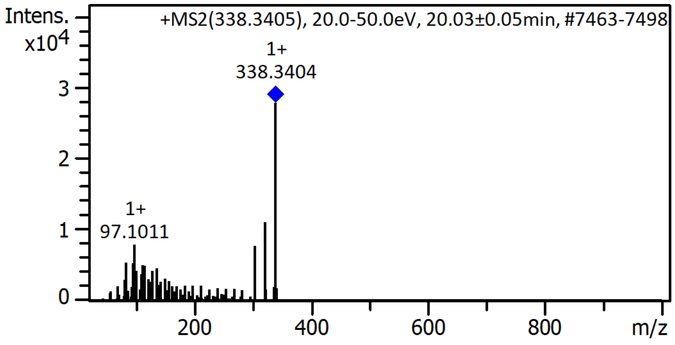

#	m/z	Res.	S/N	I	I %	FWHM
1	83.0853	8926	678.0	5424	19.5	0.0093
2	95.0847	9349	656.7	5253	18.9	0.0102
3	97.1011	9103	990.6	7925	28.5	0.0107
4	111.1159	10640	626.6	5013	18.0	0.0104
5	114.0908	10345	607.3	4858	17.5	0.0110
6	135.1156	10828	567.0	4536	16.3	0.0125
7	303.3023	12761	972.1	7777	28.0	0.0238
8	321.3142	12759	1390.0	11120	40.0	0.0252
9	338.3404	12365	3473.9	27791	100.0	0.0274
10	339.3425	14054	592.0	4736	17.0	0.0241

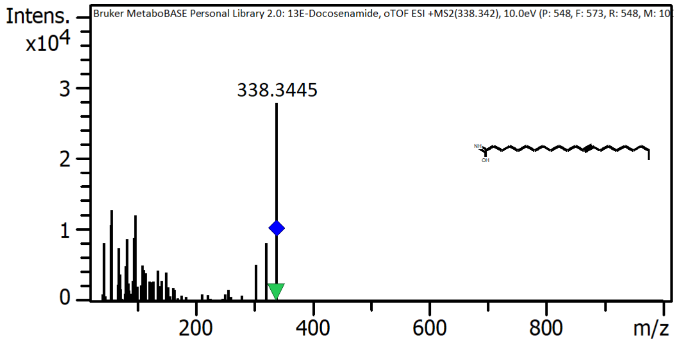

#	m/z	Res.	S/N	I	I %	FWHM
1	43.0563	1411	59.0	8198	29.5	0.0305
2	55.0554	1804	77.4	10755	38.7	0.0305
3	57.0708	1870	92.0	12784	46.0	0.0305
4	69.0704	2263	54.2	7531	27.1	0.0305
5	83.0866	2723	63.2	8782	31.6	0.0305
6	95.0854	3116	64.4	8949	32.2	0.0305
7	97.1004	3182	87.2	12117	43.6	0.0305
8	321.3132	10529	59.0	8198	29.5	0.0305
9	338.3412	11087	64.8	9004	32.4	0.0305
10	338.3445	11087	199.8	27763	100.0	0.0305

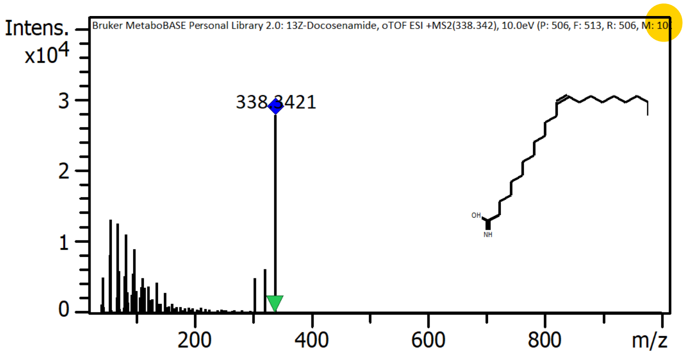

#	m/z	Res.	S/N	I	I %	FWHM
1	55.0550	1804	59.2	8226	29.6	0.0305
2	57.0708	1870	95.0	13201	47.5	0.0305
3	69.0709	2263	90.8	12617	45.4	0.0305
4	71.0863	2329	43.2	6003	21.6	0.0305
5	81.0703	2657	37.2	5169	18.6	0.0305
6	83.0861	2723	80.0	11116	40.0	0.0305
7	95.0860	3116	40.6	5642	20.3	0.0305
8	97.1014	3182	65.2	9060	32.6	0.0305
9	321.3154	10529	45.2	6281	22.6	0.0305
10	338.3421	11087	199.8	27763	100.0	0.0305

Cmpd 1652, AutoMSn(152.0562), 21.70 min

Compound Spectrum List Report

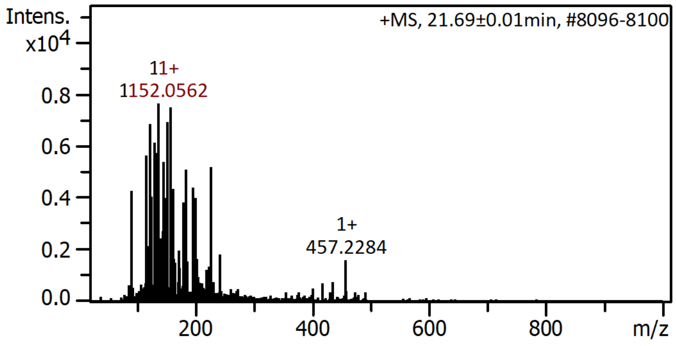

#	m/z	Res.	S/N	I	I %	FWHM
1	115.9630	10886	312.8	5631	73.7	0.0107
2	121.9657	10685	380.8	6854	89.8	0.0114
3	130.1580	10580	340.0	6121	80.2	0.0123
4	133.9733	10576	318.2	5728	75.0	0.0127
5	136.0610	10862	424.2	7636	100.0	0.0125
6	144.9817	9573	300.1	5402	70.7	0.0151
7	152.0562	11151	385.1	6933	90.8	0.0136
8	157.0831	11501	416.0	7488	98.1	0.0137
9	184.9851	9580	283.4	5101	66.8	0.0193
10	226.9502	11355	287.9	5182	67.9	0.0200

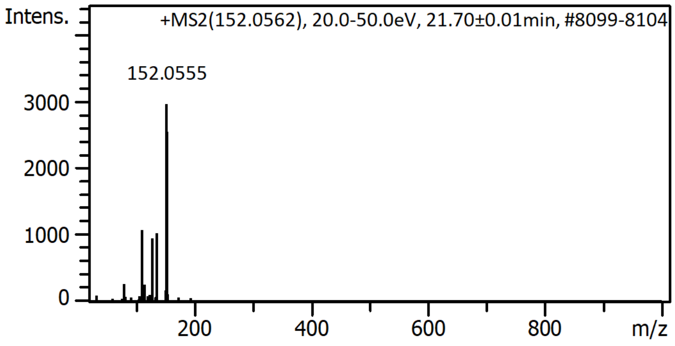

#	m/z	Res.	S/N	I	I %	FWHM
1	80.0244	6068	9.2	263	8.9	0.0132
2	110.0339	10438	37.9	1081	36.5	0.0105
3	115.0030	12297	9.0	258	8.7	0.0094
4	123.0738	13656	3.7	105	3.5	0.0090
5	128.0449	11169	33.4	951	32.1	0.0115
6	135.0297	12522	36.1	1029	34.7	0.0108
7	150.9522	15868	5.7	164	5.5	0.0095
8	152.0555	8302	103.9	2963	100.0	0.0183
9	153.0402	12784	89.5	2550	86.1	0.0120
10	154.0330	16753	3.8	108	3.6	0.0092

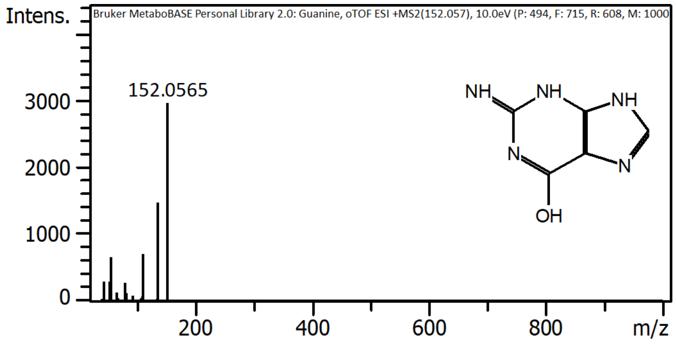

#	m/z	Res.	S/N	I	I %	FWHM
1	43.0292	3087	8.2	293	9.9	0.0139
2	53.0137	3804	8.2	293	9.9	0.0139
3	55.0294	3948	18.7	667	22.5	0.0139
4	65.0142	4665	3.7	133	4.5	0.0139
5	80.0248	5742	7.7	276	9.3	0.0139
6	82.0406	5886	3.4	121	4.1	0.0139
7	93.0083	6673	2.3	83	2.8	0.0139
8	110.0350	7895	20.1	714	24.1	0.0139
9	135.0299	9688	41.6	1478	49.9	0.0139
10	152.0565	10910	83.2	2960	100.0	0.0139

Cmpd 1673, AutoMSn(181.1209), 21.99 min

Compound Spectrum List Report

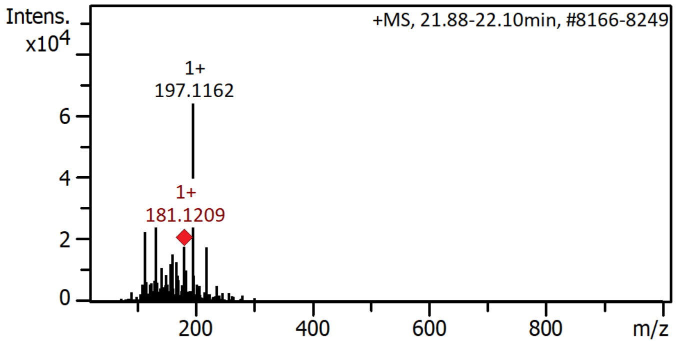

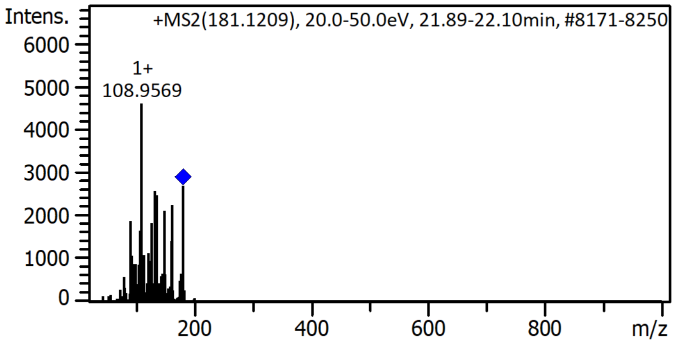

#	m/z	Res.	S/N	I	I %	FWHM
1	113.9625	9977	5008.5	22538	35.3	0.0114
2	131.9731	10528	5665.6	25495	39.9	0.0125
3	141.9584	9838	2409.3	10842	17.0	0.0144
4	157.0826	11003	2702.2	12160	19.0	0.0143
5	159.9683	10414	3407.1	15332	24.0	0.0154
6	167.9622	10627	2872.4	12926	20.2	0.0158
7	181.1209	11064	3956.9	17806	27.9	0.0164
8	185.0413	11421	2212.5	9956	15.6	0.0162
9	197.1162	11336	14200.6	63903	100.0	0.0174
10	219.0979	11631	3914.8	17617	27.6	0.0188

#	m/z	Res.	S/N	I	I %	FWHM
1	90.9471	9884	307.3	1882	40.9	0.0092
2	107.0842	10453	270.2	1655	36.0	0.0102
3	108.9569	10559	751.3	4602	100.0	0.0103
4	126.9681	11324	300.9	1843	40.1	0.0112
5	131.9735	10731	422.4	2587	56.2	0.0123
6	135.1154	10433	404.1	2475	53.8	0.0130
7	148.9762	10235	346.3	2121	46.1	0.0146
8	159.9677	10576	231.4	1418	30.8	0.0151
9	163.1102	10165	368.4	2257	49.0	0.0160
10	181.1205	11143	440.2	2696	58.6	0.0163

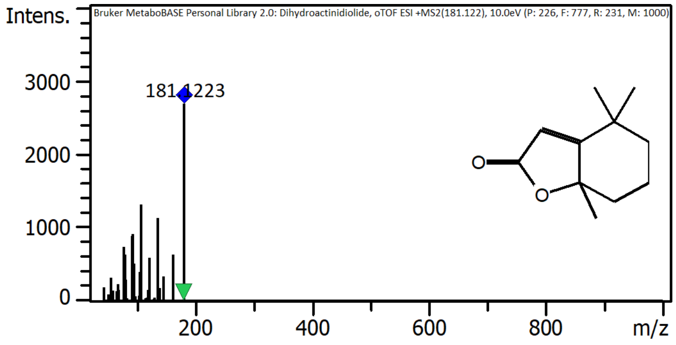

#	m/z	Res.	S/N	I	I %	FWHM
1	77.0390	7655	46.0	744	27.6	0.0101
2	79.0547	7855	39.7	642	23.8	0.0101
3	91.0545	9047	55.2	892	33.1	0.0101
4	93.0701	9248	57.0	922	34.2	0.0101
5	95.0855	9448	32.3	523	19.4	0.0101
6	107.0854	10640	81.8	1324	49.1	0.0101
7	121.1003	12033	36.8	596	22.1	0.0101
8	135.1165	13425	70.5	1141	42.3	0.0101
9	163.1114	16207	39.5	639	23.7	0.0101
10	181.1223	17997	166.5	2694	100.0	0.0101

Cmpd 1691, AutoMSn(181.1210), 22.26 min

Compound Spectrum List Report

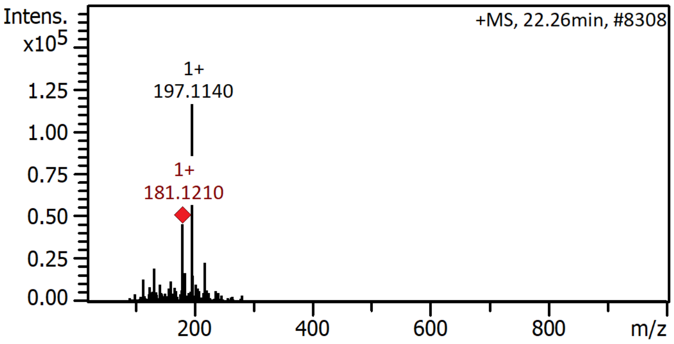

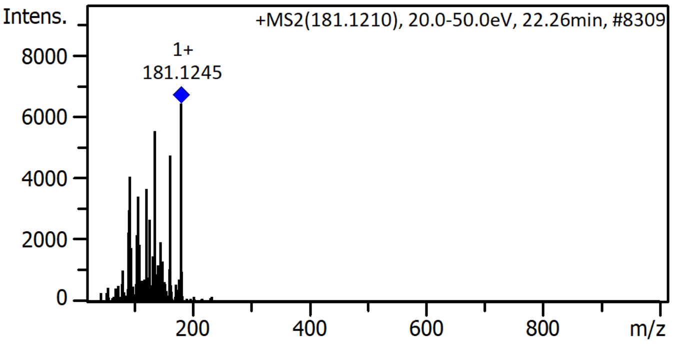

#	m/z	Res.	S/N	I	I %	FWHM
1	113.9615	8428	364.6	13127	11.3	0.0135
2	131.9731	9074	537.0	19332	16.6	0.0145
3	141.9575	7076	276.8	9964	8.6	0.0201
4	159.9667	10931	325.8	11728	10.1	0.0146
5	181.1210	8679	1269.7	45708	39.3	0.0209
6	185.0397	9873	468.6	16869	14.5	0.0187
7	197.1140	9781	3232.1	116356	100.0	0.0202
8	198.1181	10123	422.9	15223	13.1	0.0196
9	203.1023	7445	272.5	9811	8.4	0.0273
10	219.0960	9170	646.5	23275	20.0	0.0239

#	m/z	Res.	S/N	I	I %	FWHM
1	90.9484	11117	45.9	2247	35.0	0.0082
2	91.0544	8627	60.6	2969	46.3	0.0106
3	93.0708	9885	82.8	4059	63.2	0.0094
4	105.0709	8898	44.2	2165	33.7	0.0118
5	107.0862	9236	69.6	3408	53.1	0.0116
6	121.1004	8702	74.9	3669	57.2	0.0139
7	126.9729	9758	54.2	2657	41.4	0.0130
8	135.1173	9316	112.7	5524	86.1	0.0145
9	163.1128	11823	96.7	4737	73.8	0.0138
10	181.1245	11058	131.0	6419	100.0	0.0164

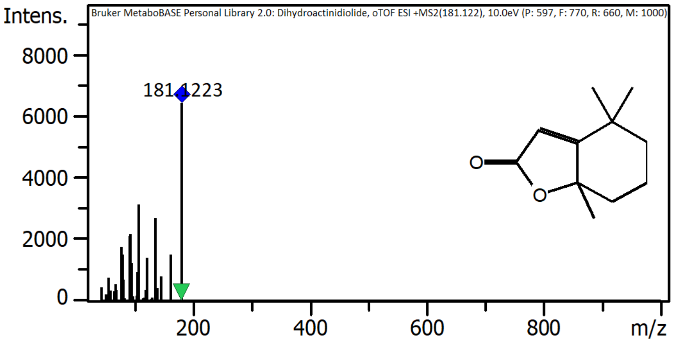

#	m/z	Res.	S/N	I	I %	FWHM
1	77.0390	4569	46.0	1772	27.6	0.0169
2	79.0547	4688	39.7	1528	23.8	0.0169
3	91.0545	5400	55.2	2125	33.1	0.0169
4	93.0701	5520	57.0	2195	34.2	0.0169
5	95.0855	5639	32.3	1245	19.4	0.0169
6	107.0854	6351	81.8	3152	49.1	0.0169
7	121.1003	7182	36.8	1419	22.1	0.0169
8	135.1165	8013	70.5	2715	42.3	0.0169
9	163.1114	9674	39.5	1521	23.7	0.0169
10	181.1223	10742	166.5	6413	100.0	0.0169

Cmpd 1695, AutoMSn(181.1208), 22.31 min

Compound Spectrum List Report

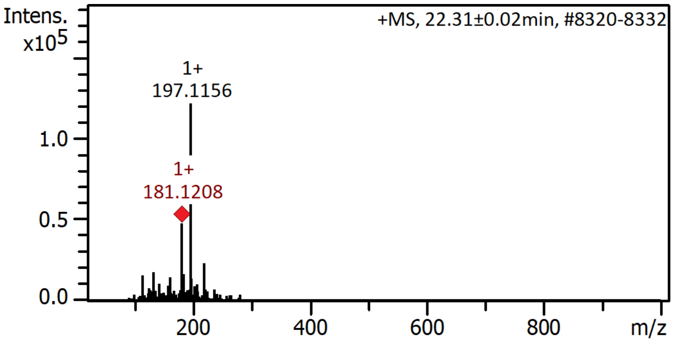

#	m/z	Res.	S/N	I	I %	FWHM
1	113.9625	9447	1291.0	15492	12.7	0.0121
2	131.9725	10382	1451.6	17420	14.3	0.0127
3	141.9577	8784	883.8	10606	8.7	0.0162
4	159.9682	9564	1197.9	14375	11.8	0.0167
5	181.1208	10409	3997.6	47971	39.5	0.0174
6	185.0406	9422	1363.9	16367	13.5	0.0196
7	197.1156	10671	10128.4	121541	100.0	0.0185
8	198.1185	10489	1148.7	13785	11.3	0.0189
9	207.1365	10124	823.6	9883	8.1	0.0205
10	219.0973	10290	1945.7	23348	19.2	0.0213

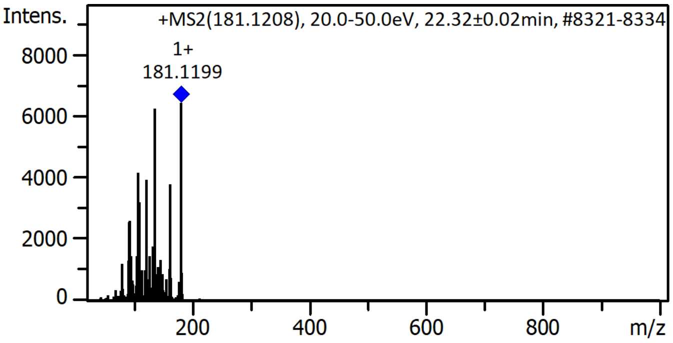

#	m/z	Res.	S/N	I	I %	FWHM
1	91.0538	8322	160.4	2566	40.0	0.0109
2	93.0686	9200	162.4	2598	40.5	0.0101
3	107.0842	8017	259.6	4154	64.7	0.0134
4	108.9566	8554	200.9	3214	50.1	0.0127
5	121.1008	8580	245.3	3925	61.1	0.0141
6	126.9673	10777	92.0	1471	22.9	0.0118
7	131.9719	11837	111.3	1780	27.7	0.0111
8	135.1149	10497	390.5	6248	97.3	0.0129
9	163.1090	10411	236.3	3781	58.9	0.0157
10	181.1199	11661	401.3	6421	100.0	0.0155

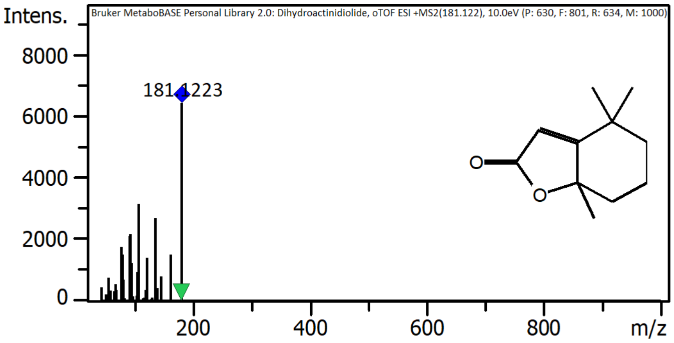

#	m/z	Res.	S/N	I	I %	FWHM
1	77.0390	4370	46.0	1772	27.6	0.0176
2	79.0547	4484	39.7	1528	23.8	0.0176
3	91.0545	5165	55.2	2125	33.1	0.0176
4	93.0701	5279	57.0	2196	34.2	0.0176
5	95.0855	5394	32.3	1246	19.4	0.0176
6	107.0854	6074	81.8	3153	49.1	0.0176
7	121.1003	6869	36.8	1419	22.1	0.0176
8	135.1165	7664	70.5	2716	42.3	0.0176
9	163.1114	9252	39.5	1522	23.7	0.0176
10	181.1223	10274	166.5	6415	100.0	0.0176

Cmpd 1710, AutoMSn(153.1264), 22.51 min

Compound Spectrum List Report

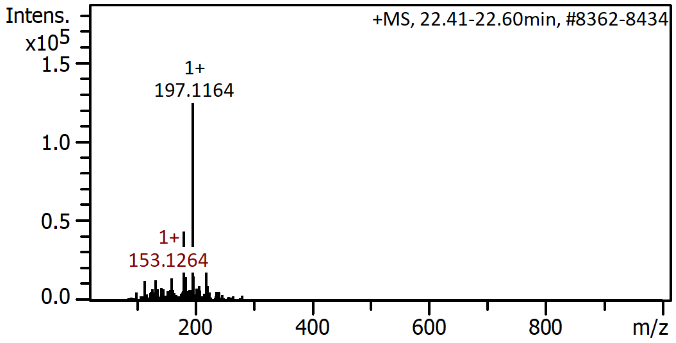

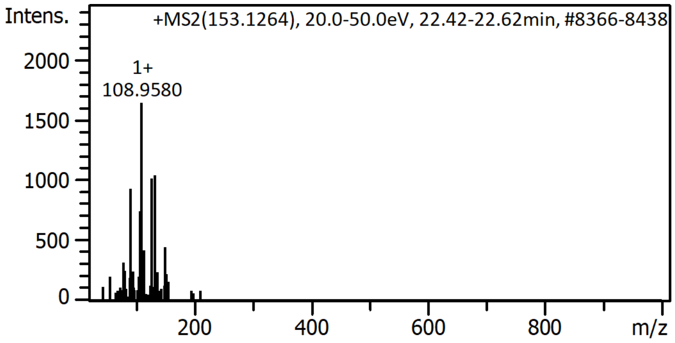

#	m/z	Res.	S/N	I	I %	FWHM
1	113.9628	10636	671.8	12092	9.7	0.0107
2	131.9729	11117	695.3	12515	10.1	0.0119
3	159.9679	11620	776.1	13969	11.2	0.0138
4	181.1211	11236	2416.9	43504	35.0	0.0161
5	185.0410	11406	821.0	14778	11.9	0.0162
6	197.1164	11470	6902.3	124242	100.0	0.0172
7	198.1192	11442	833.4	15002	12.1	0.0173
8	207.1376	12339	511.4	9206	7.4	0.0168
9	219.0973	11520	1088.9	19600	15.8	0.0190
10	222.0172	12574	508.7	9157	7.4	0.0177

#	m/z	Res.	S/N	I	I %	FWHM
1	79.0557	6383	12.4	322	19.6	0.0124
2	81.0691	11411	9.5	248	15.1	0.0071
3	90.9484	9293	35.8	931	56.8	0.0098
4	91.0550	11647	30.6	797	48.6	0.0078
5	107.0854	10594	28.8	748	45.6	0.0101
6	108.9580	10340	63.1	1641	100.0	0.0105
7	113.9599	7699	16.3	423	25.8	0.0148
8	126.9684	12824	39.1	1016	61.9	0.0099
9	131.9730	12219	40.0	1040	63.4	0.0108
10	149.9890	8426	17.2	448	27.3	0.0178

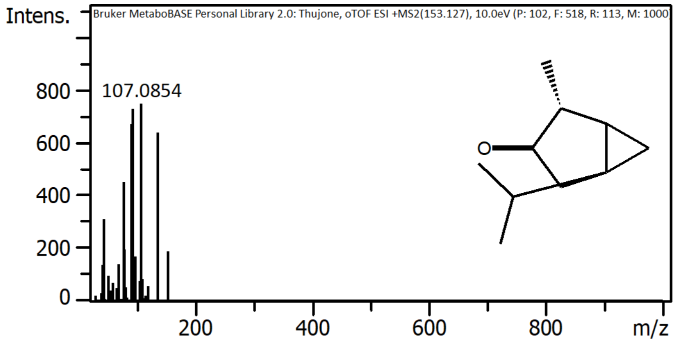

#	m/z	Res.	S/N	I	I %	FWHM
1	43.0183	4593	25.8	173	23.2	0.0094
2	43.0554	4597	46.4	312	41.8	0.0094
3	77.0392	8226	67.3	453	60.7	0.0094
4	79.0547	8441	29.3	197	26.4	0.0094
5	91.0543	9722	99.7	671	89.8	0.0094
6	93.0699	9937	108.2	728	97.5	0.0094
7	97.0654	10364	25.4	171	22.9	0.0094
8	107.0854	11434	111.0	747	100.0	0.0094
9	135.1174	14427	95.1	640	85.7	0.0094
10	153.1277	16350	28.2	190	25.4	0.0094

Cmpd 1717, AutoMSn(181.1217), 22.64 min

Compound Spectrum List Report

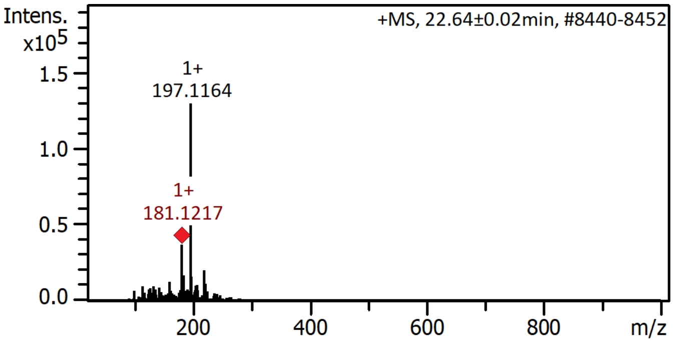

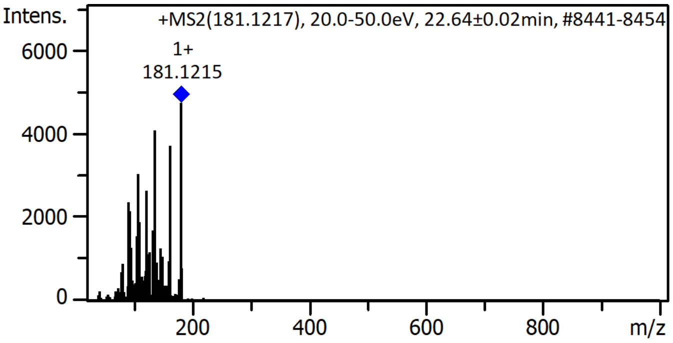

#	m/z	Res.	S/N	I	I %	FWHM
1	131.9736	10839	801.8	9622	7.4	0.0122
2	159.9684	10846	1037.9	12455	9.6	0.0147
3	181.1217	11496	3105.3	37264	28.8	0.0158
4	185.0418	11039	1382.1	16585	12.8	0.0168
5	197.1164	11150	10785.4	129425	100.0	0.0177
6	198.1195	11467	1326.3	15916	12.3	0.0173
7	205.0022	10491	812.8	9753	7.5	0.0195
8	207.1371	11371	873.1	10477	8.1	0.0182
9	219.0984	11932	1669.2	20030	15.5	0.0184
10	222.0176	11299	915.1	10981	8.5	0.0196

#	m/z	Res.	S/N	I	I %	FWHM
1	90.9457	9105	113.5	1817	38.4	0.0100
2	91.0531	10471	147.9	2367	50.0	0.0087
3	93.0698	10078	134.7	2155	45.5	0.0092
4	107.0847	9568	190.2	3044	64.3	0.0112
5	108.9579	10795	118.5	1897	40.1	0.0101
6	121.0996	9590	165.1	2641	55.8	0.0126
7	131.9734	12397	105.5	1688	35.6	0.0106
8	135.1158	10471	255.0	4079	86.2	0.0129
9	163.1106	10254	231.7	3707	78.3	0.0159
10	181.1215	10727	295.9	4734	100.0	0.0169

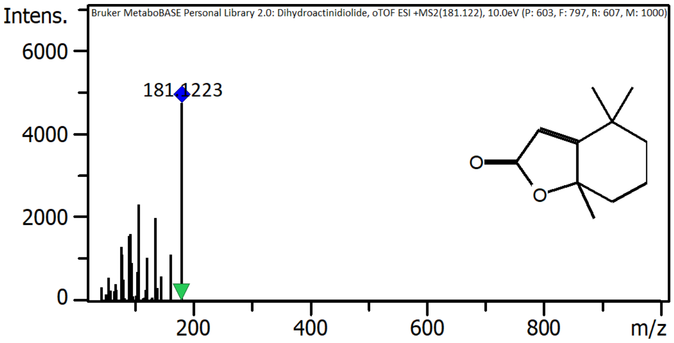

#	m/z	Res.	S/N	I	I %	FWHM
1	77.0390	4377	46.0	1307	27.6	0.0176
2	79.0547	4491	39.7	1127	23.8	0.0176
3	91.0545	5173	55.2	1567	33.1	0.0176
4	93.0701	5287	57.0	1619	34.2	0.0176
5	95.0855	5402	32.3	918	19.4	0.0176
6	107.0854	6084	81.8	2325	49.1	0.0176
7	121.1003	6880	36.8	1046	22.1	0.0176
8	135.1165	7676	70.5	2003	42.3	0.0176
9	163.1114	9267	39.5	1122	23.7	0.0176
10	181.1223	10290	166.5	4730	100.0	0.0176

Cmpd 1724, AutoMSn(124.0383), 22.77 min

Compound Spectrum List Report

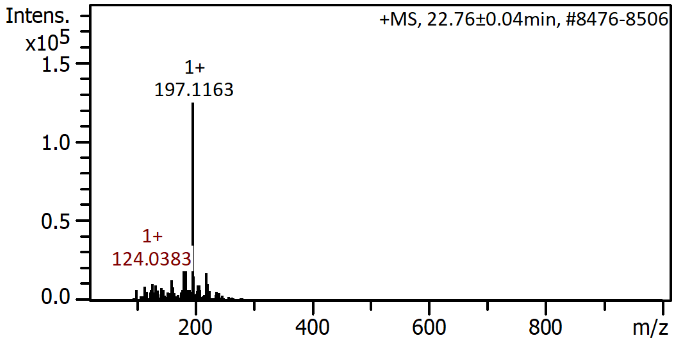

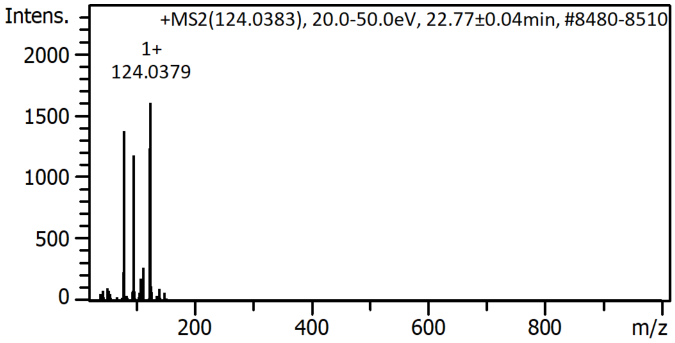

#	m/z	Res.	S/N	I	I %	FWHM
1	127.0382	9865	1444.3	10399	8.4	0.0129
2	159.9683	11458	1754.6	12633	10.1	0.0140
3	181.1216	10928	4427.2	31876	25.6	0.0166
4	185.0415	11128	2590.8	18654	15.0	0.0166
5	197.1163	11418	17294.2	124518	100.0	0.0173
6	198.1201	10753	2088.1	15034	12.1	0.0184
7	205.0023	12300	1341.0	9655	7.8	0.0167
8	207.1366	11859	1330.7	9581	7.7	0.0175
9	219.0987	11541	2352.8	16940	13.6	0.0190
10	222.0177	12315	1425.4	10263	8.2	0.0180

#	m/z	Res.	S/N	I	I %	FWHM
1	78.0344	9464	19.7	228	14.2	0.0082
2	80.0485	9292	118.5	1375	85.9	0.0086
3	96.0431	8696	101.4	1177	73.5	0.0110
4	108.0437	13694	15.4	179	11.2	0.0079
5	111.0314	13422	11.6	135	8.4	0.0083
6	112.0371	12228	23.1	267	16.7	0.0092
7	123.0546	10867	106.4	1235	77.1	0.0113
8	124.0379	10825	138.0	1600	100.0	0.0115
9	124.0759	12636	9.3	108	6.7	0.0098
10	125.0435	14161	10.2	118	7.4	0.0088

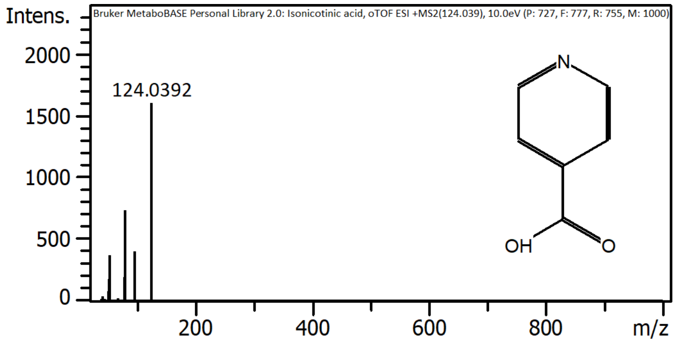

#	m/z	Res.	S/N	I	I %	FWHM
1	41.0392	3492	4.8	38	2.4	0.0118
2	51.0224	4341	10.2	82	5.1	0.0118
3	52.0307	4427	22.4	179	11.2	0.0118
4	53.0390	4513	46.8	374	23.4	0.0118
5	78.0343	6640	13.8	110	6.9	0.0118
6	78.0345	6640	6.8	54	3.4	0.0118
7	79.0421	6726	24.2	194	12.1	0.0118
8	80.0500	6811	92.0	736	46.0	0.0118
9	96.0449	8172	50.6	405	25.3	0.0118
10	124.0392	10554	199.8	1599	100.0	0.0118

Cmpd 1743, AutoMSn(181.1206), 23.05 min

Compound Spectrum List Report

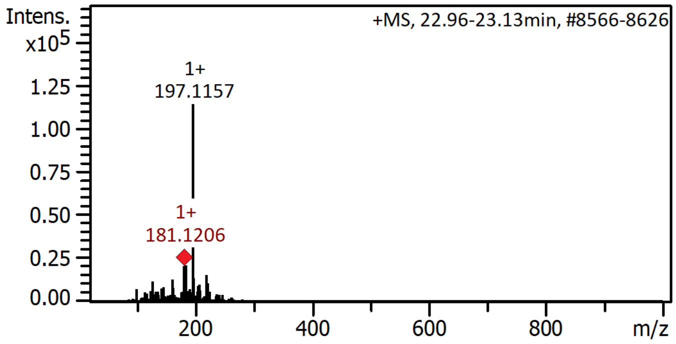

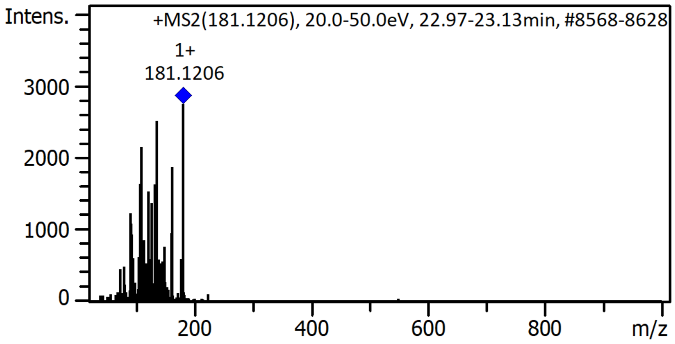

#	m/z	Res.	S/N	I	I %	FWHM
1	127.0383	10351	1292.6	11633	10.2	0.0123
2	159.9684	11361	1402.7	12625	11.0	0.0141
3	181.1206	11004	2295.4	20658	18.1	0.0165
4	185.0409	11652	2344.0	21096	18.4	0.0159
5	197.1157	11540	12707.2	114365	100.0	0.0171
6	198.1190	10590	1510.1	13591	11.9	0.0187
7	205.0018	12016	998.5	8987	7.9	0.0171
8	207.1368	11396	1094.1	9847	8.6	0.0182
9	219.0982	11583	1703.3	15329	13.4	0.0189
10	222.0175	12169	1184.0	10656	9.3	0.0182

#	m/z	Res.	S/N	I	I %	FWHM
1	90.9465	9549	102.4	1229	44.7	0.0095
2	91.0525	9753	90.5	1086	39.5	0.0093
3	107.0847	10652	136.5	1638	59.6	0.0101
4	108.9564	10138	179.0	2148	78.2	0.0107
5	121.1006	10305	127.8	1533	55.8	0.0118
6	126.9675	11634	114.6	1376	50.1	0.0109
7	131.9735	10307	136.3	1636	59.5	0.0128
8	135.1156	10425	209.7	2517	91.6	0.0130
9	163.1104	10175	156.0	1872	68.1	0.0160
10	181.1206	12672	228.9	2747	100.0	0.0143

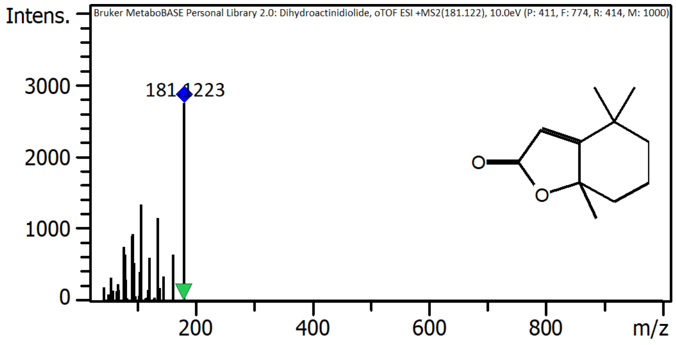

#	m/z	Res.	S/N	I	I %	FWHM
1	77.0390	4652	46.0	758	27.6	0.0166
2	79.0547	4774	39.7	654	23.8	0.0166
3	91.0545	5498	55.2	909	33.1	0.0166
4	93.0701	5620	57.0	940	34.2	0.0166
5	95.0855	5742	32.3	533	19.4	0.0166
6	107.0854	6467	81.8	1349	49.1	0.0166
7	121.1003	7313	36.8	607	22.1	0.0166
8	135.1165	8159	70.5	1162	42.3	0.0166
9	163.1114	9850	39.5	651	23.7	0.0166
10	181.1223	10937	166.5	2745	100.0	0.0166

Cmpd 1747, AutoMSn(191.1420), 23.12 min

Compound Spectrum List Report

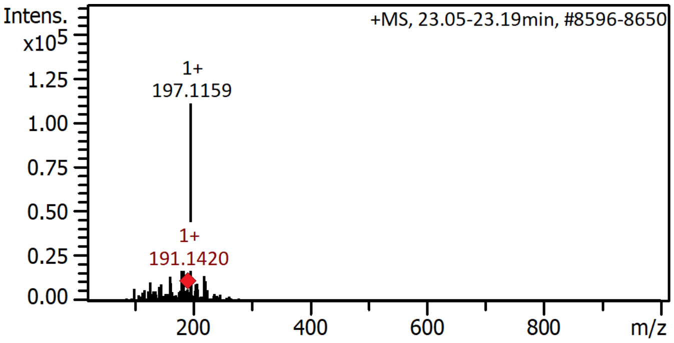

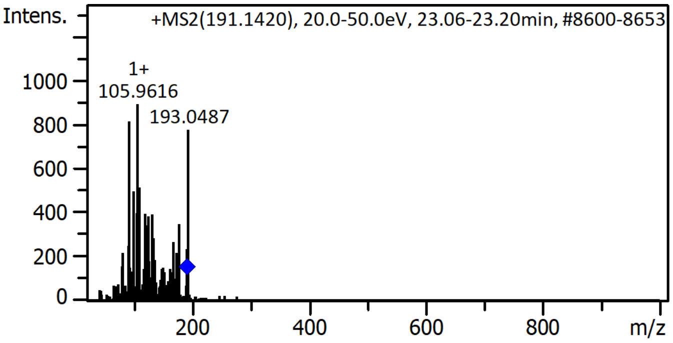

#	m/z	Res.	S/N	I	I %	FWHM
1	127.0373	10312	2002.8	10300	9.3	0.0123
2	159.9675	11235	2587.3	13306	12.0	0.0142
3	163.0584	11068	1896.3	9752	8.8	0.0147
4	181.1207	11030	3659.2	18819	17.0	0.0164
5	185.0408	11186	4138.2	21282	19.2	0.0165
6	197.1159	11331	21519.7	110673	100.0	0.0174
7	198.1194	10520	2574.9	13242	12.0	0.0188
8	207.1365	11382	1874.1	9638	8.7	0.0182
9	219.0975	11918	2660.6	13683	12.4	0.0184
10	222.0166	12290	2151.9	11067	10.0	0.0181

#	m/z	Res.	S/N	I	I %	FWHM
1	91.0536	10335	116.4	815	91.2	0.0088
2	98.9836	10453	70.9	497	55.6	0.0095
3	105.0688	12086	57.2	400	44.8	0.0087
4	105.9616	11196	127.6	893	100.0	0.0095
5	109.1030	9389	73.3	513	57.5	0.0116
6	119.0842	12043	56.5	396	44.3	0.0099
7	123.9725	9926	55.0	385	43.1	0.0125
8	131.0839	7823	55.9	392	43.8	0.0168
9	178.0256	10472	49.8	349	39.0	0.0170
10	193.0487	11827	110.7	775	86.8	0.0163

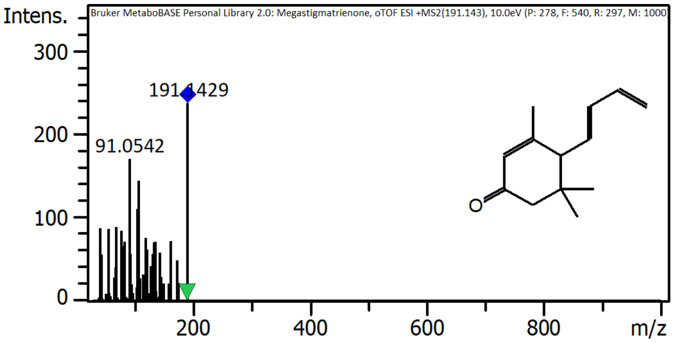

#	m/z	Res.	S/N	I	I %	FWHM
1	41.0394	4754	73.8	88	36.9	0.0086
2	55.0544	6377	73.4	87	36.7	0.0086
3	69.0337	7996	75.4	89	37.7	0.0086
4	77.0389	8923	71.2	84	35.6	0.0086
5	91.0542	10547	144.0	171	72.1	0.0086
6	105.0700	12170	93.0	110	46.5	0.0086
7	107.0850	12404	122.0	145	61.1	0.0086
8	119.0855	13794	64.4	76	32.2	0.0086
9	161.0963	18660	60.8	72	30.4	0.0086
10	191.1429	22140	199.8	237	100.0	0.0086

Cmpd 1750, AutoMSn(124.0397), 23.16 min

Compound Spectrum List Report

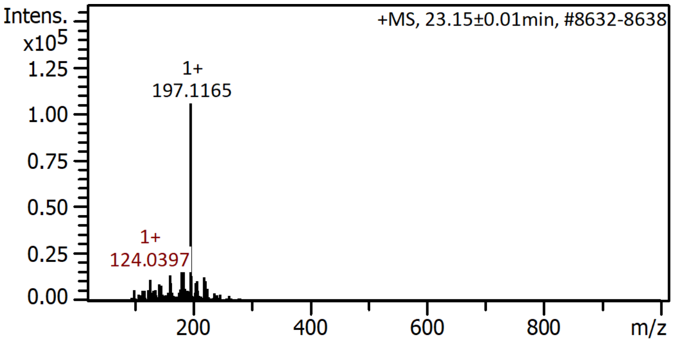

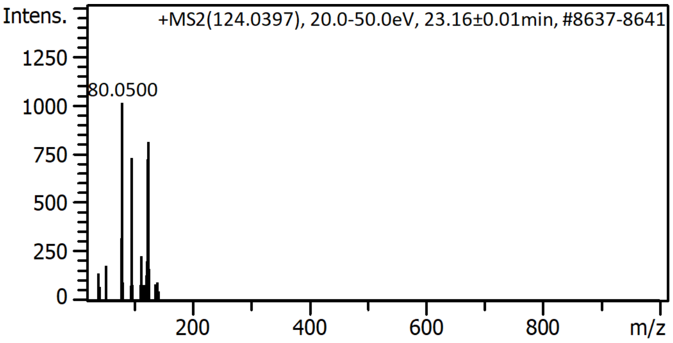

#	m/z	Res.	S/N	I	I %	FWHM
1	127.0379	11080	612.6	11026	10.5	0.0115
2	159.9684	10722	742.0	13357	12.7	0.0149
3	163.0589	11451	531.4	9566	9.1	0.0142
4	181.1209	11527	950.6	17112	16.2	0.0157
5	185.0411	10707	1252.0	22536	21.4	0.0173
6	197.1165	11000	5855.8	105405	100.0	0.0179
7	198.1200	11716	732.8	13191	12.5	0.0169
8	207.1373	11738	570.6	10271	9.7	0.0176
9	219.0990	12170	702.8	12651	12.0	0.0180
10	222.0171	12715	588.5	10593	10.0	0.0175

#	m/z	Res.	S/N	I	I %	FWHM
1	39.0208	5997	5.2	139	13.7	0.0065
2	52.0227	5563	6.7	178	17.6	0.0094
3	78.0340	11079	12.1	322	31.8	0.0070
4	80.0500	7817	38.1	1011	100.0	0.0102
5	96.0435	11503	27.6	731	72.3	0.0083
6	112.0418	12834	8.7	231	22.9	0.0087
7	122.0875	14281	7.7	205	20.3	0.0085
8	123.0610	10111	27.3	723	71.5	0.0122
9	124.0380	13815	30.7	814	80.5	0.0090
10	125.0542	12345	6.1	162	16.0	0.0101

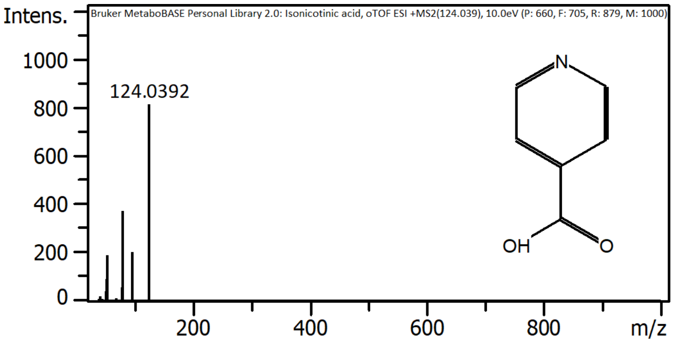

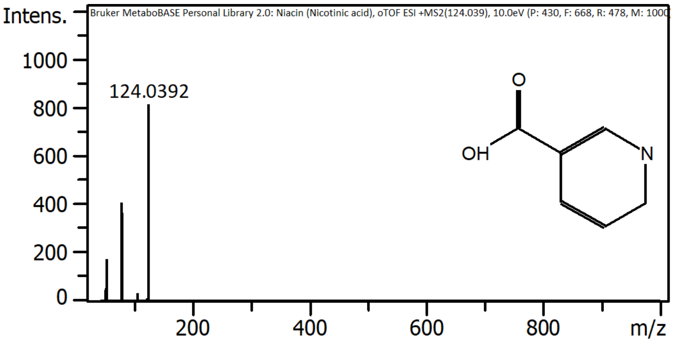

#	m/z	Res.	S/N	I	I %	FWHM
1	41.0392	5900	4.8	20	2.4	0.0070
2	51.0224	7335	10.2	41	5.1	0.0070
3	52.0307	7480	22.4	91	11.2	0.0070
4	53.0390	7625	46.8	190	23.4	0.0070
5	78.0343	11218	13.8	56	6.9	0.0070
6	78.0345	11218	6.8	28	3.4	0.0070
7	79.0421	11363	24.2	98	12.1	0.0070
8	80.0500	11508	92.0	374	46.0	0.0070
9	96.0449	13808	50.6	206	25.3	0.0070
10	124.0392	17832	199.8	813	100.0	0.0070

#	m/z	Res.	S/N	I	I %	FWHM
1	44.9973	6469	1.4	6	0.7	0.0070
2	51.0229	7335	11.4	46	5.7	0.0070
3	52.0246	7479	13.2	54	6.6	0.0070
4	53.0389	7625	43.0	175	21.5	0.0070
5	78.0344	11218	100.4	408	50.3	0.0070
6	79.0422	11363	10.2	41	5.1	0.0070
7	80.0499	11508	89.6	364	44.8	0.0070
8	106.0288	15243	8.0	33	4.0	0.0070
9	122.0236	17542	2.6	11	1.3	0.0070
10	124.0392	17832	199.8	813	100.0	0.0070

Cmpd 1760, AutoMSn(124.0386), 23.32 min

Compound Spectrum List Report

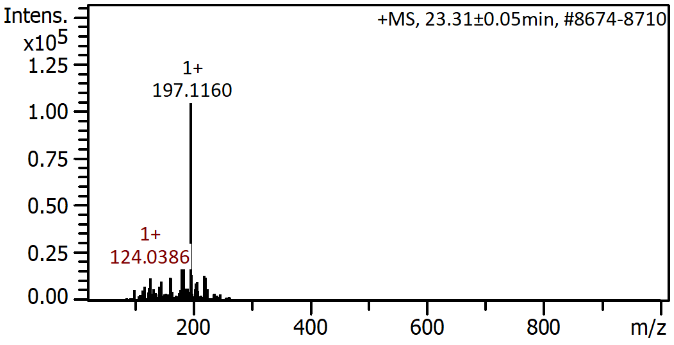

#	m/z	Res.	S/N	I	I %	FWHM
1	127.0376	9465	1294.9	11654	11.2	0.0134
2	145.0484	11195	1089.8	9809	9.4	0.0130
3	159.9677	10890	1318.7	11868	11.4	0.0147
4	163.0582	10418	1292.7	11634	11.2	0.0157
5	181.1212	11632	1837.4	16537	15.9	0.0156
6	185.0408	11252	2414.7	21732	20.9	0.0164
7	197.1160	11069	11559.8	104038	100.0	0.0178
8	198.1182	10970	1494.7	13452	12.9	0.0181
9	219.0979	11842	1431.8	12886	12.4	0.0185
10	222.0161	12819	1317.1	11854	11.4	0.0173

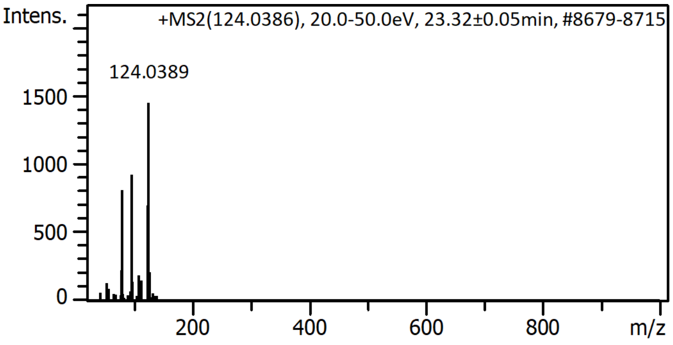

#	m/z	Res.	S/N	I	I %	FWHM
1	53.0409	7827	9.5	126	8.7	0.0068
2	78.0323	9103	16.7	221	15.3	0.0086
3	80.0493	7746	61.2	811	56.1	0.0103
4	96.0448	9915	69.6	923	63.8	0.0097
5	97.0407	13144	10.5	139	9.6	0.0074
6	108.0433	9586	14.0	185	12.8	0.0113
7	112.0376	11670	11.1	147	10.2	0.0096
8	123.0524	10013	52.7	698	48.2	0.0123
9	124.0389	10501	109.2	1447	100.0	0.0118
10	126.0534	9960	15.5	206	14.2	0.0127

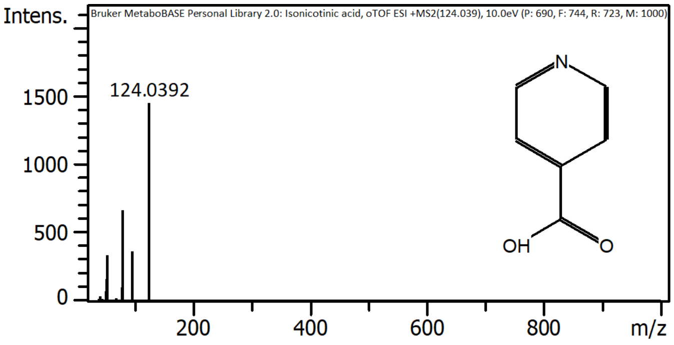

#	m/z	Res.	S/N	I	I %	FWHM
1	41.0392	3331	4.8	35	2.4	0.0123
2	51.0224	4141	10.2	74	5.1	0.0123
3	52.0307	4223	22.4	162	11.2	0.0123
4	53.0390	4305	46.8	338	23.4	0.0123
5	78.0343	6334	13.8	100	6.9	0.0123
6	78.0345	6334	6.8	49	3.4	0.0123
7	79.0421	6416	24.2	175	12.1	0.0123
8	80.0500	6497	92.0	665	46.0	0.0123
9	96.0449	7796	50.6	366	25.3	0.0123
10	124.0392	10068	199.8	1445	100.0	0.0123

Cmpd 1767, AutoMSn(153.1254), 23.40 min

Compound Spectrum List Report

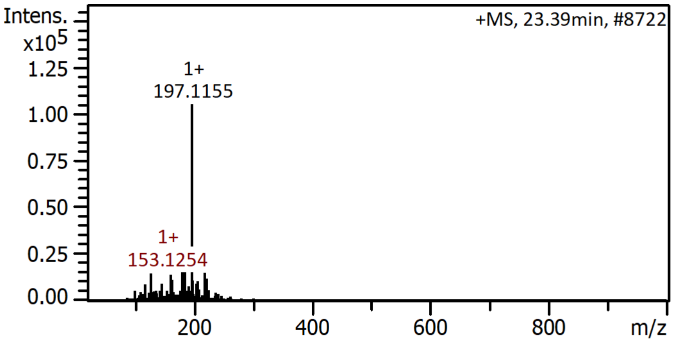

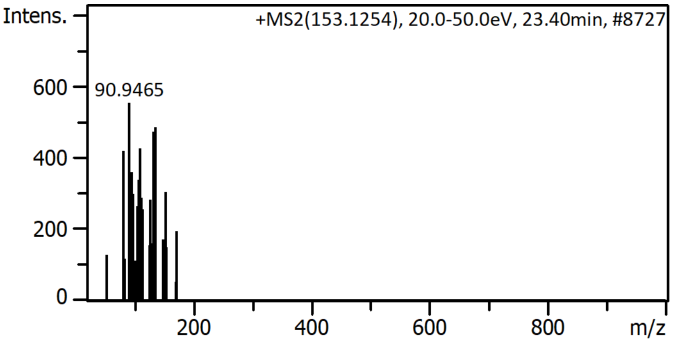

#	m/z	Res.	S/N	I	I %	FWHM
1	127.0371	9899	403.3	14517	13.8	0.0128
2	159.9681	11168	386.7	13920	13.2	0.0143
3	163.0581	11893	312.1	11236	10.7	0.0137
4	181.1201	11741	424.2	15270	14.5	0.0154
5	185.0412	11987	700.8	25229	24.0	0.0154
6	197.1155	11679	2923.2	105235	100.0	0.0169
7	198.1182	11730	301.3	10846	10.3	0.0169
8	207.1359	12884	287.1	10335	9.8	0.0161
9	219.0969	12479	410.4	14774	14.0	0.0176
10	222.0168	13540	330.0	11879	11.3	0.0164

#	m/z	Res.	S/N	I	I %	FWHM
1	81.0664	9511	8.1	420	75.9	0.0085
2	90.9465	12726	10.6	553	100.0	0.0071
3	95.0850	12389	6.9	361	65.3	0.0077
4	97.0671	11099	5.8	299	54.1	0.0087
5	107.0832	12062	6.5	339	61.3	0.0089
6	108.9577	12993	8.2	426	77.0	0.0084
7	111.0418	12873	5.5	288	52.1	0.0086
8	131.9737	7630	9.1	473	85.5	0.0173
9	135.1196	11032	9.3	485	87.7	0.0122
10	153.0325	13700	5.8	304	55.0	0.0112

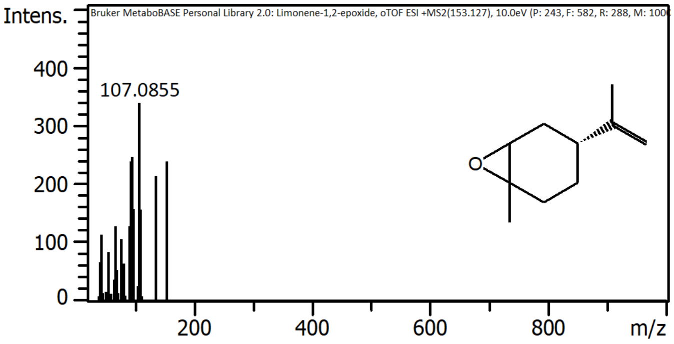

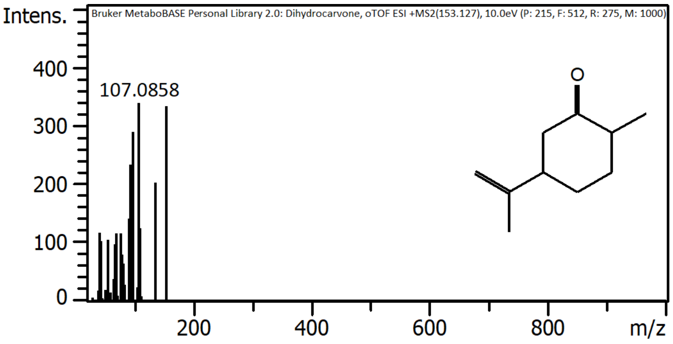

#	m/z	Res.	S/N	I	I %	FWHM
1	43.0190	6098	67.8	115	33.9	0.0071
2	67.0548	9504	76.2	129	38.1	0.0071
3	91.0542	12906	75.8	128	37.9	0.0071
4	93.0702	13192	141.2	239	70.7	0.0071
5	95.0856	13478	145.8	247	73.0	0.0071
6	97.0651	13758	93.6	159	46.8	0.0071
7	107.0855	15178	199.8	339	100.0	0.0071
8	109.1017	15464	92.8	157	46.4	0.0071
9	135.1167	19152	126.4	214	63.3	0.0071
10	153.1271	21704	141.0	239	70.6	0.0071

#	m/z	Res.	S/N	I	I %	FWHM
1	41.0400	5817	69.2	117	34.6	0.0071
2	69.0705	9790	68.8	117	34.4	0.0071
3	91.0547	12906	84.0	142	42.0	0.0071
4	93.0704	13192	137.8	234	69.0	0.0071
5	95.0860	13478	88.6	150	44.3	0.0071
6	97.0651	13758	170.6	289	85.4	0.0071
7	107.0858	15178	199.8	339	100.0	0.0071
8	109.1011	15464	73.8	125	36.9	0.0071
9	135.1169	19152	119.8	203	60.0	0.0071
10	153.1274	21704	197.0	334	98.6	0.0071

Cmpd 1783, AutoMSn(191.1423), 23.63 min

Compound Spectrum List Report

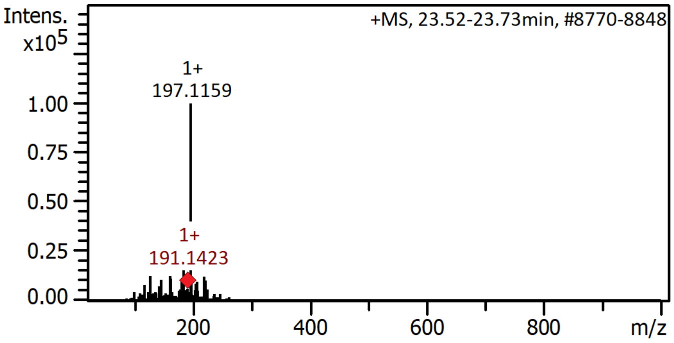

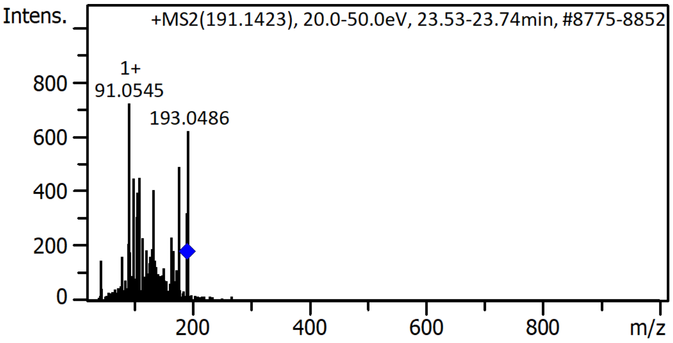

#	m/z	Res.	S/N	I	I %	FWHM
1	127.0381	10112	3447.3	12410	12.5	0.0126
2	145.0483	10521	2938.1	10577	10.6	0.0138
3	159.9682	10889	3460.9	12459	12.5	0.0147
4	163.0588	10954	3060.7	11019	11.1	0.0149
5	181.1211	11554	3070.5	11054	11.1	0.0157
6	185.0410	11832	6534.1	23523	23.6	0.0156
7	197.1159	11343	27678.8	99644	100.0	0.0174
8	198.1191	10827	3339.4	12022	12.1	0.0183
9	219.0978	11359	3341.1	12028	12.1	0.0193
10	222.0169	11661	2832.3	10196	10.2	0.0190

#	m/z	Res.	S/N	I	I %	FWHM
1	91.0545	7819	133.4	721	100.0	0.0116
2	98.9823	8825	83.1	449	62.3	0.0112
3	105.0691	11046	57.1	309	42.8	0.0095
4	105.9615	10620	73.2	395	54.8	0.0100
5	109.1009	9775	83.3	450	62.5	0.0112
6	133.0276	10197	75.2	406	56.3	0.0130
7	133.1010	9865	59.2	320	44.3	0.0135
8	178.0229	13791	90.9	491	68.1	0.0129
9	191.0692	13164	59.3	320	44.5	0.0145
10	193.0486	12305	114.8	620	86.0	0.0157

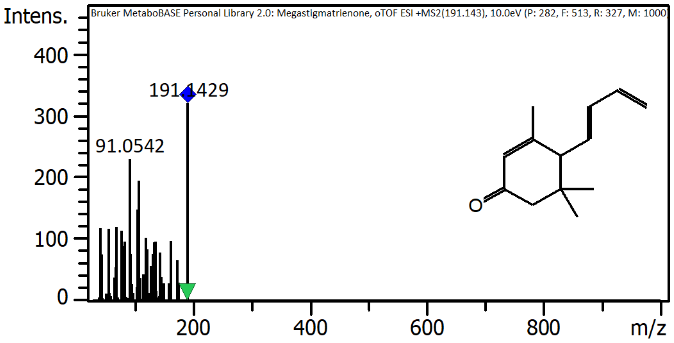

#	m/z	Res.	S/N	I	I %	FWHM
1	41.0394	5603	73.8	118	36.9	0.0073
2	55.0544	7516	73.4	118	36.7	0.0073
3	69.0337	9425	75.4	121	37.7	0.0073
4	77.0389	10518	71.2	114	35.6	0.0073
5	91.0542	12431	144.0	231	72.1	0.0073
6	105.0700	14344	93.0	149	46.5	0.0073
7	107.0850	14619	122.0	195	61.1	0.0073
8	119.0855	16258	64.4	103	32.2	0.0073
9	161.0963	21993	60.8	97	30.4	0.0073
10	191.1429	26095	199.8	320	100.0	0.0073

Cmpd 1784, AutoMSn(124.0390), 23.63 min

Compound Spectrum List Report

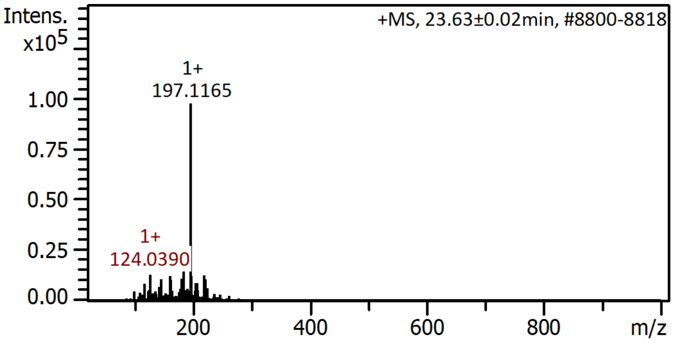

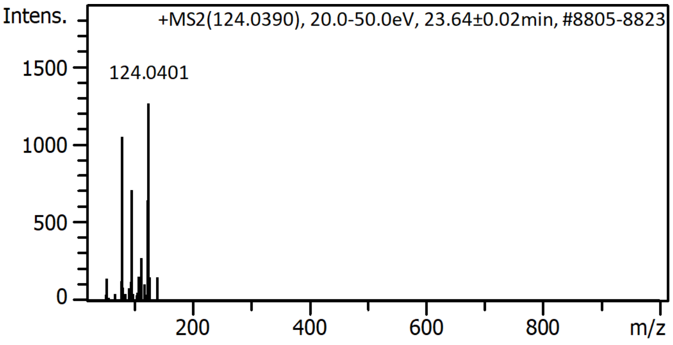

#	m/z	Res.	S/N	I	I %	FWHM
1	127.0383	10123	1058.2	12698	13.0	0.0125
2	145.0485	11402	877.0	10524	10.8	0.0127
3	159.9682	10529	1002.5	12030	12.3	0.0152
4	163.0592	11227	851.2	10214	10.5	0.0145
5	181.1210	12321	909.7	10916	11.2	0.0147
6	185.0413	11898	2049.3	24591	25.2	0.0156
7	197.1165	11204	8118.1	97417	100.0	0.0176
8	198.1196	11091	1024.8	12298	12.6	0.0179
9	219.0978	11704	1034.9	12418	12.7	0.0187
10	222.0171	11451	875.7	10508	10.8	0.0194

#	m/z	Res.	S/N	I	I %	FWHM
1	53.0383	8888	7.5	140	11.1	0.0060
2	78.0290	12030	6.7	126	10.0	0.0065
3	80.0495	8927	56.3	1051	83.4	0.0090
4	96.0434	9362	38.0	710	56.3	0.0103
5	108.0426	10845	8.2	153	12.1	0.0100
6	112.0397	13624	14.8	275	21.9	0.0082
7	123.0564	11386	34.5	645	51.2	0.0108
8	124.0401	10432	67.5	1260	100.0	0.0119
9	126.0509	14621	8.0	150	11.9	0.0086
10	140.0280	16206	8.1	151	12.0	0.0086

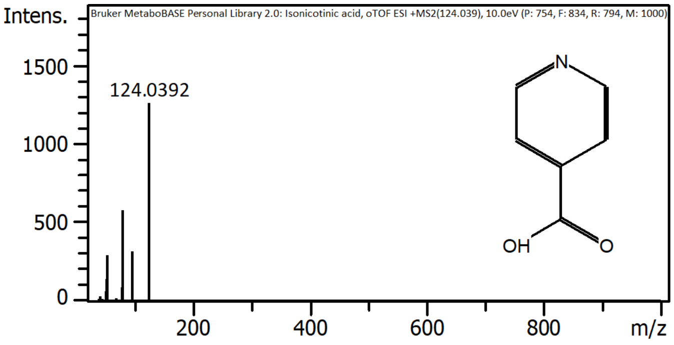

#	m/z	Res.	S/N	I	I %	FWHM
1	41.0392	3708	4.8	30	2.4	0.0111
2	51.0224	4610	10.2	64	5.1	0.0111
3	52.0307	4701	22.4	141	11.2	0.0111
4	53.0390	4793	46.8	295	23.4	0.0111
5	78.0343	7051	13.8	87	6.9	0.0111
6	78.0345	7051	6.8	43	3.4	0.0111
7	79.0421	7142	24.2	152	12.1	0.0111
8	80.0500	7233	92.0	580	46.0	0.0111
9	96.0449	8679	50.6	319	25.3	0.0111
10	124.0392	11208	199.8	1259	100.0	0.0111

Cmpd 1806, AutoMSn(127.0377), 23.93 min

Compound Spectrum List Report

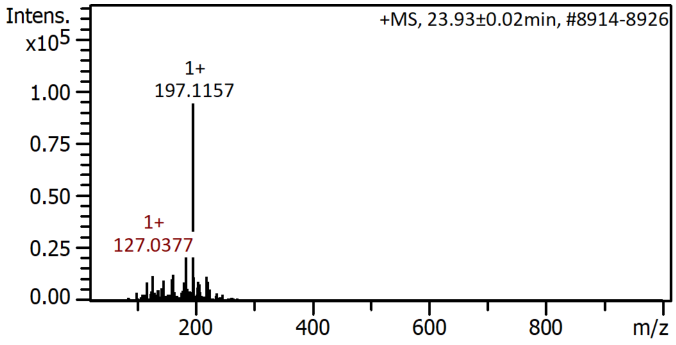

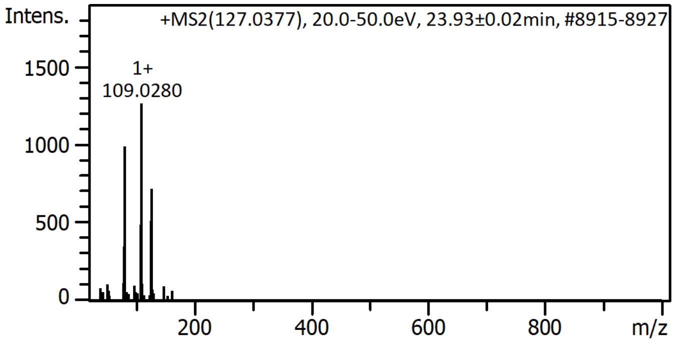

#	m/z	Res.	S/N	I	I %	FWHM
1	127.0377	10724	973.2	11679	12.4	0.0118
2	145.0482	10761	800.3	9604	10.2	0.0135
3	159.9681	10539	853.6	10244	10.9	0.0152
4	163.0586	10291	1040.4	12484	13.3	0.0158
5	185.0411	11674	2055.3	24663	26.2	0.0159
6	197.1157	11132	7847.1	94165	100.0	0.0177
7	198.1197	11156	920.0	11040	11.7	0.0178
8	205.0023	11617	756.9	9083	9.6	0.0176
9	219.0979	12214	958.9	11507	12.2	0.0179
10	222.0178	13416	739.8	8877	9.4	0.0165

#	m/z	Res.	S/N	I	I %	FWHM
1	51.0195	9425	5.3	102	8.1	0.0054
2	79.0550	11818	5.8	111	8.8	0.0067
3	80.0503	9236	18.2	352	27.8	0.0087
4	81.0327	9710	51.0	987	78.1	0.0083
5	108.0435	9266	25.4	491	38.8	0.0117
6	109.0280	10482	65.4	1264	100.0	0.0104
7	110.0338	12652	5.6	109	8.6	0.0087
8	126.0551	12514	26.6	514	40.7	0.0101
9	127.0404	9101	37.1	718	56.8	0.0140
10	127.0825	22510	6.5	125	9.9	0.0056

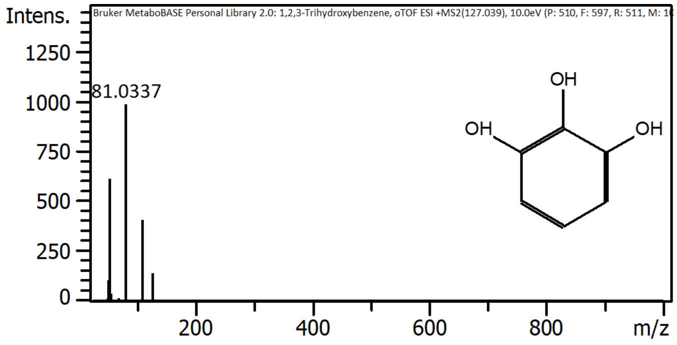

#	m/z	Res.	S/N	I	I %	FWHM
1	50.0138	5126	3.2	16	1.6	0.0098
2	51.0231	5230	21.2	105	10.6	0.0098
3	52.0328	5333	2.2	11	1.1	0.0098
4	53.0388	5437	124.4	614	62.3	0.0098
5	55.0182	5639	7.6	37	3.8	0.0098
6	68.9975	7072	2.8	14	1.4	0.0098
7	81.0337	8306	199.8	986	100.0	0.0098
8	81.1130	8314	6.6	33	3.3	0.0098
9	109.0274	11175	82.8	408	41.4	0.0098
10	127.0386	13022	28.4	140	14.2	0.0098

Cmpd 1814, AutoMSn(191.1427), 24.02 min

Compound Spectrum List Report

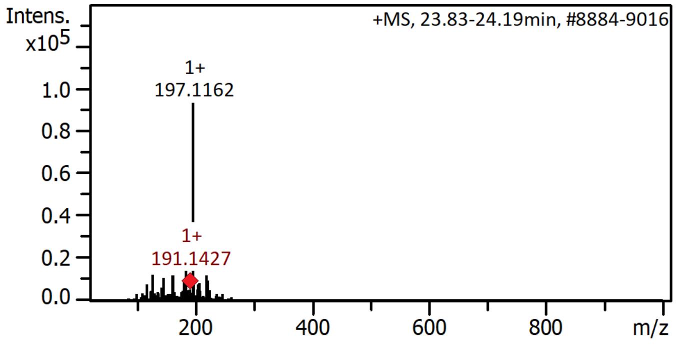

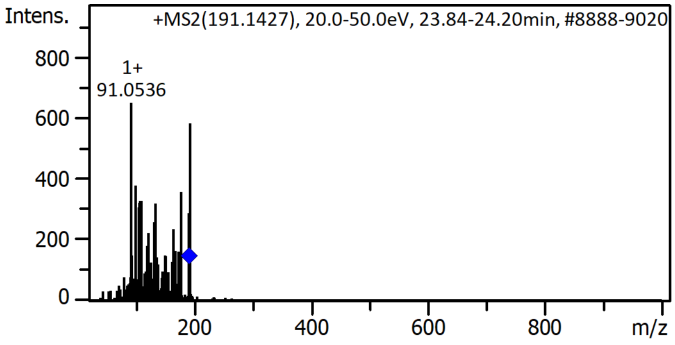

#	m/z	Res.	S/N	I	I %	FWHM
1	127.0380	10616	4021.7	12065	13.0	0.0120
2	145.0484	10608	3510.8	10532	11.3	0.0137
3	159.9684	10984	3959.0	11877	12.8	0.0146
4	163.0590	10528	3965.4	11896	12.8	0.0155
5	181.1211	10932	2907.2	8722	9.4	0.0166
6	185.0413	11642	8243.5	24730	26.6	0.0159
7	197.1162	11428	31022.0	93066	100.0	0.0172
8	198.1194	11420	3744.3	11233	12.1	0.0173
9	219.0976	12293	3975.0	11925	12.8	0.0178
10	222.0172	11932	3180.5	9541	10.3	0.0186

#	m/z	Res.	S/N	I	I %	FWHM
1	91.0536	10323	158.9	649	100.0	0.0088
2	98.9843	9246	92.6	378	58.3	0.0107
3	105.0675	10414	75.2	307	47.3	0.0101
4	105.9612	8508	78.8	322	49.6	0.0125
5	107.0845	7793	80.2	328	50.5	0.0137
6	109.1002	10986	80.6	329	50.8	0.0099
7	133.1007	12622	78.4	320	49.3	0.0105
8	178.0269	14308	87.4	357	55.0	0.0124
9	191.0708	11115	70.7	289	44.5	0.0172
10	193.0491	13511	142.7	583	89.8	0.0143

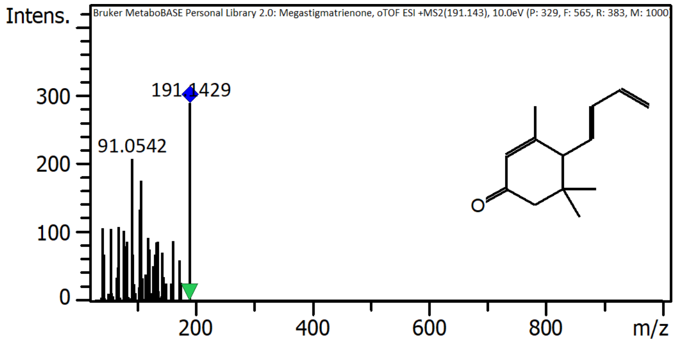

#	m/z	Res.	S/N	I	I %	FWHM
1	41.0394	5704	73.8	107	36.9	0.0072
2	55.0544	7652	73.4	106	36.7	0.0072
3	69.0337	9594	75.4	109	37.7	0.0072
4	77.0389	10707	71.2	103	35.6	0.0072
5	91.0542	12655	144.0	208	72.1	0.0072
6	105.0700	14603	93.0	134	46.5	0.0072
7	107.0850	14883	122.0	176	61.1	0.0072
8	119.0855	16551	64.4	93	32.2	0.0072
9	161.0963	22389	60.8	88	30.4	0.0072
10	191.1429	26565	199.8	289	100.0	0.0072

Cmpd 1848, AutoMSn(191.1422), 24.50 min

Compound Spectrum List Report

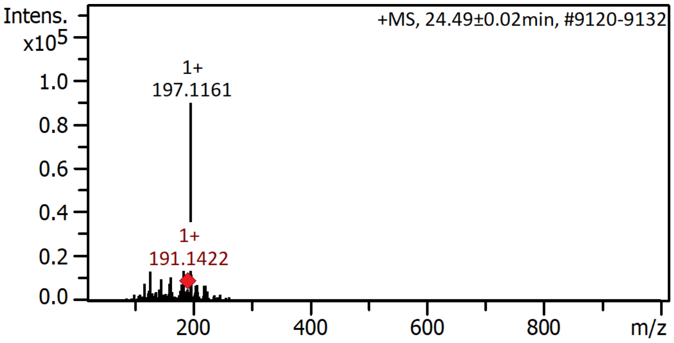

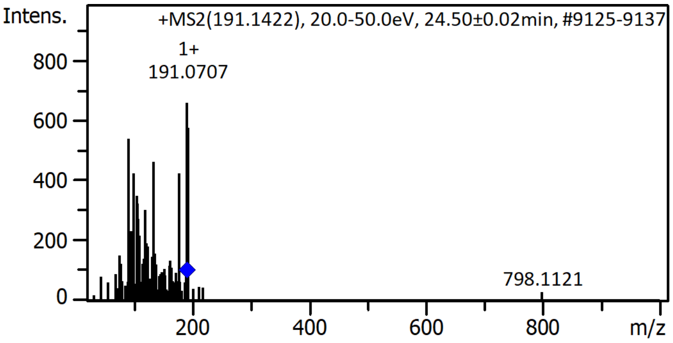

#	m/z	Res.	S/N	I	I %	FWHM
1	117.0540	9326	629.9	7559	8.4	0.0126
2	127.0379	9864	1099.3	13191	14.7	0.0129
3	145.0486	10309	813.6	9763	10.9	0.0141
4	159.9685	11815	651.4	7817	8.7	0.0135
5	163.0591	10473	890.7	10689	11.9	0.0156
6	181.1208	11295	616.3	7396	8.2	0.0160
7	185.0407	11556	1876.0	22512	25.1	0.0160
8	197.1161	11607	7471.7	89660	100.0	0.0170
9	198.1191	11114	979.0	11748	13.1	0.0178
10	207.1368	12718	600.4	7205	8.0	0.0163

#	m/z	Res.	S/N	I	I %	FWHM
1	91.0523	8637	33.0	539	81.9	0.0105
2	98.9818	10438	26.0	424	64.5	0.0095
3	105.0692	10623	21.4	350	53.3	0.0099
4	105.9609	7003	19.8	324	49.2	0.0151
5	119.0838	12550	18.6	303	46.1	0.0095
6	133.0323	11907	28.3	462	70.3	0.0112
7	133.1021	14068	23.2	379	57.6	0.0095
8	178.0226	12175	26.0	425	64.6	0.0146
9	191.0707	12718	40.3	658	100.0	0.0150
10	193.0505	12437	35.2	576	87.5	0.0155

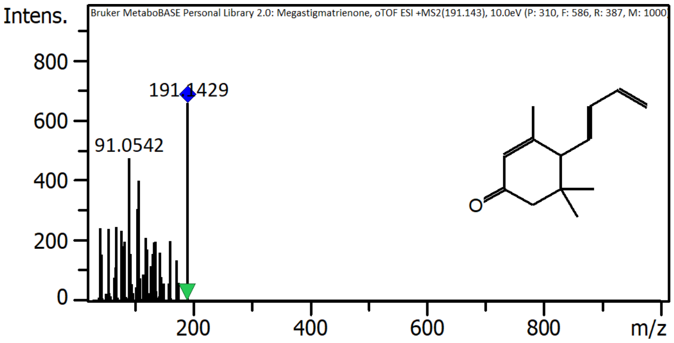

#	m/z	Res.	S/N	I	I %	FWHM
1	41.0394	2889	73.8	243	36.9	0.0142
2	55.0544	3875	73.4	241	36.7	0.0142
3	69.0337	4859	75.4	248	37.7	0.0142
4	77.0389	5423	71.2	234	35.6	0.0142
5	91.0542	6409	144.0	474	72.1	0.0142
6	105.0700	7396	93.0	306	46.5	0.0142
7	107.0850	7538	122.0	401	61.1	0.0142
8	119.0855	8383	64.4	212	32.2	0.0142
9	161.0963	11340	60.8	200	30.4	0.0142
10	191.1429	13455	199.8	657	100.0	0.0142

Cmpd 1856, AutoMSn(153.1277), 24.61 min

Compound Spectrum List Report

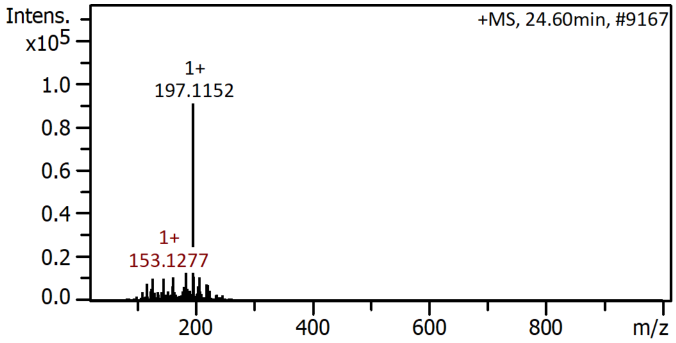

#	m/z	Res.	S/N	I	I %	FWHM
1	117.0530	8859	214.0	7705	8.5	0.0132
2	127.0378	9092	280.3	10090	11.1	0.0140
3	145.0477	10796	283.2	10196	11.2	0.0134
4	163.0570	10638	297.3	10702	11.8	0.0153
5	185.0406	11253	680.8	24509	27.0	0.0164
6	197.1152	11101	2523.8	90856	100.0	0.0178
7	198.1174	11419	307.3	11061	12.2	0.0174
8	207.1368	12437	300.6	10821	11.9	0.0167
9	219.0967	11864	210.7	7584	8.3	0.0185
10	222.0171	12113	203.4	7324	8.1	0.0183

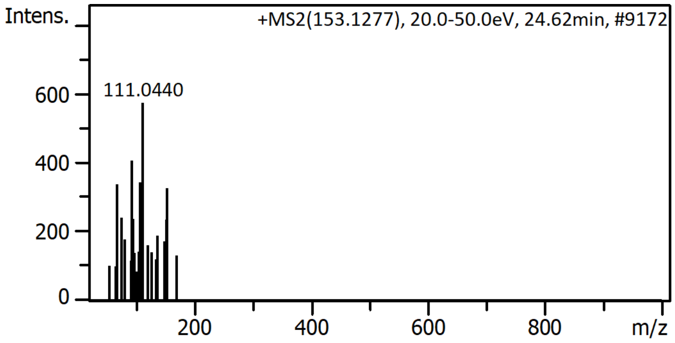

#	m/z	Res.	S/N	I	I %	FWHM
1	67.0564	11176	4.0	339	59.2	0.0060
2	75.0433	11090	2.9	242	42.2	0.0068
3	93.0716	7119	4.8	407	71.0	0.0131
4	95.0844	11998	2.8	238	41.5	0.0079
5	107.0892	11911	4.1	343	59.9	0.0090
6	109.0961	11704	3.4	286	49.9	0.0093
7	111.0440	12355	6.8	573	100.0	0.0090
8	152.0554	16866	2.8	236	41.2	0.0090
9	153.0357	10461	3.9	327	57.1	0.0146
10	153.0888	16850	2.9	246	42.9	0.0091

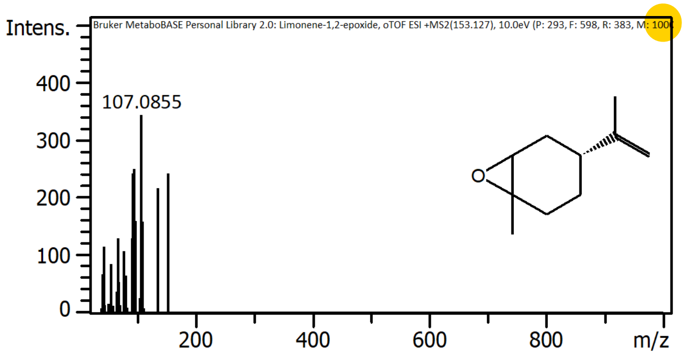

#	m/z	Res.	S/N	I	I %	FWHM
1	43.0190	4947	67.8	116	33.9	0.0087
2	67.0548	7711	76.2	131	38.1	0.0087
3	91.0542	10471	75.8	130	37.9	0.0087
4	93.0702	10703	141.2	242	70.7	0.0087
5	95.0856	10934	145.8	250	73.0	0.0087
6	97.0651	11162	93.6	161	46.8	0.0087
7	107.0855	12314	199.8	343	100.0	0.0087
8	109.1017	12546	92.8	159	46.4	0.0087
9	135.1167	15538	126.4	217	63.3	0.0087
10	153.1271	17609	141.0	242	70.6	0.0087

Cmpd 1894, AutoMSn(124.0382), 25.12 min

Compound Spectrum List Report

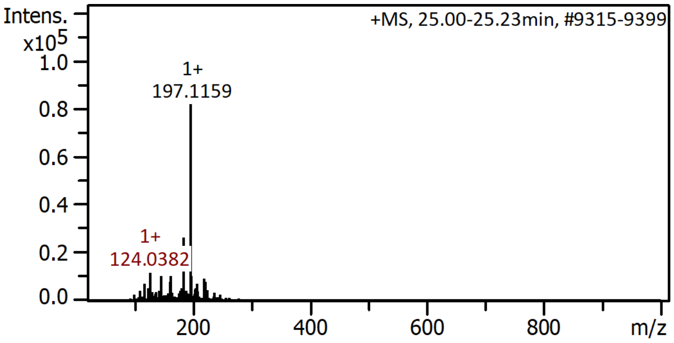

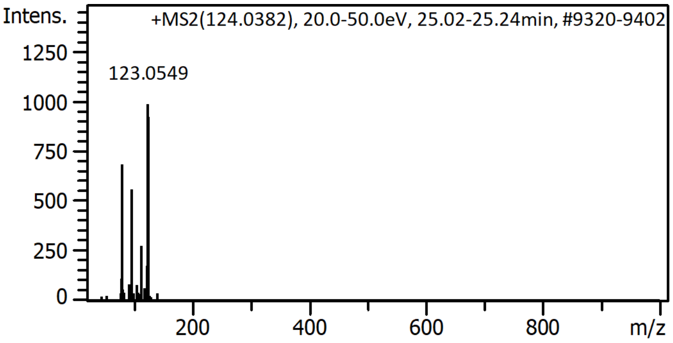

#	m/z	Res.	S/N	I	I %	FWHM
1	127.0377	10088	1613.2	11615	14.2	0.0126
2	145.0480	10269	1420.8	10230	12.5	0.0141
3	159.9677	11195	1083.3	7800	9.5	0.0143
4	163.0590	10498	1438.4	10357	12.6	0.0155
5	185.0406	11600	3675.5	26464	32.3	0.0160
6	197.1159	11580	11381.8	81949	100.0	0.0170
7	198.1192	11444	1438.9	10360	12.6	0.0173
8	207.1363	10207	994.4	7160	8.7	0.0203
9	219.0973	11219	1294.1	9318	11.4	0.0195
10	222.0178	12474	1099.0	7913	9.7	0.0178

#	m/z	Res.	S/N	I	I %	FWHM
1	78.0343	11627	9.4	111	11.3	0.0067
2	80.0478	9857	57.9	683	69.4	0.0081
3	92.0465	12763	6.9	81	8.2	0.0072
4	96.0445	8269	47.4	559	56.8	0.0116
5	105.0481	13503	6.8	80	8.1	0.0078
6	112.0391	7932	23.4	276	28.0	0.0141
7	118.0559	14025	5.3	63	6.4	0.0084
8	122.0970	13113	14.7	174	17.6	0.0093
9	123.0549	6513	83.5	985	100.0	0.0189
10	124.0376	9238	78.1	922	93.6	0.0134

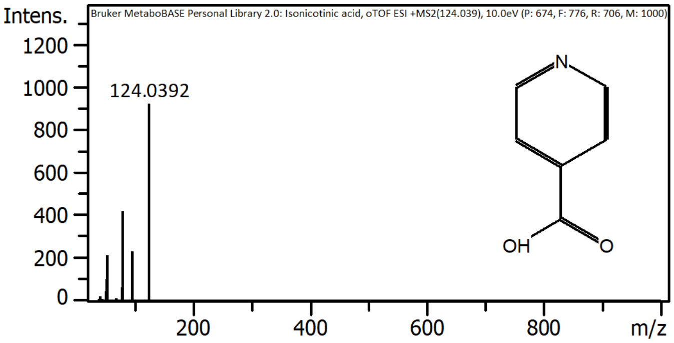

#	m/z	Res.	S/N	I	I %	FWHM
1	41.0392	3131	4.8	22	2.4	0.0131
2	51.0224	3893	10.2	47	5.1	0.0131
3	52.0307	3970	22.4	103	11.2	0.0131
4	53.0390	4047	46.8	216	23.4	0.0131
5	78.0343	5954	13.8	64	6.9	0.0131
6	78.0345	5954	6.8	31	3.4	0.0131
7	79.0421	6031	24.2	112	12.1	0.0131
8	80.0500	6108	92.0	424	46.0	0.0131
9	96.0449	7328	50.6	233	25.3	0.0131
10	124.0392	9464	199.8	921	100.0	0.0131

Cmpd 1936, AutoMSn(219.0979), 25.66 min

Compound Spectrum List Report

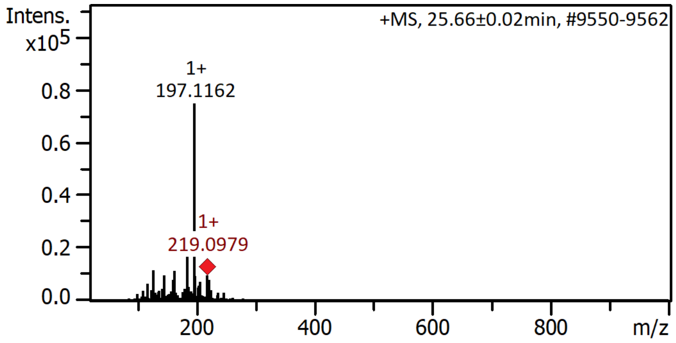

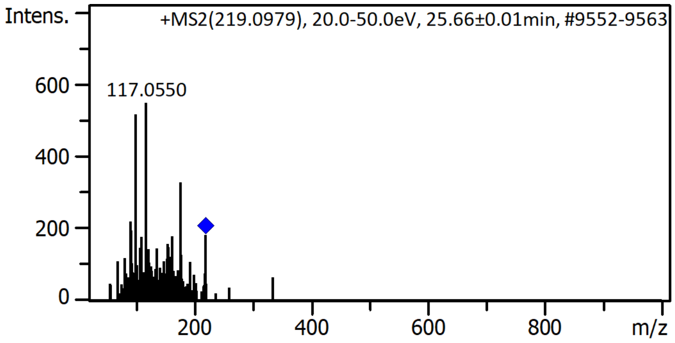

#	m/z	Res.	S/N	I	I %	FWHM
1	127.0379	10800	959.2	11510	15.4	0.0118
2	145.0484	9957	792.7	9512	12.7	0.0146
3	159.9685	10547	655.9	7870	10.5	0.0152
4	163.0588	10549	947.1	11365	15.2	0.0155
5	185.0413	11513	2061.6	24739	33.1	0.0161
6	197.1162	10934	6231.4	74776	100.0	0.0180
7	198.1195	11180	785.8	9429	12.6	0.0177
8	207.1369	11981	586.7	7040	9.4	0.0173
9	219.0979	10669	790.8	9489	12.7	0.0205
10	222.0174	12370	649.6	7796	10.4	0.0179

#	m/z	Res.	S/N	I	I %	FWHM
1	90.9473	11646	13.2	220	40.3	0.0078
2	91.0529	12424	11.7	195	35.7	0.0073
3	99.0430	8194	31.0	516	94.3	0.0121
4	108.9628	6781	10.7	178	32.6	0.0161
5	117.0550	11094	32.8	547	100.0	0.0106
6	153.9583	11417	9.5	159	29.1	0.0135
7	154.9546	14428	9.0	150	27.4	0.0107
8	163.0559	15704	10.8	180	32.8	0.0104
9	176.9849	10468	19.7	328	60.0	0.0169
10	219.1021	18401	11.0	183	33.5	0.0119

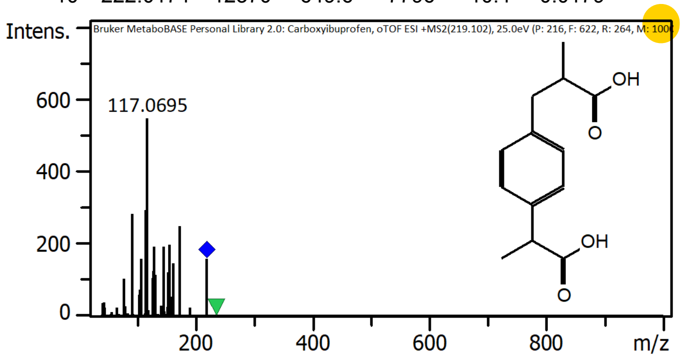

#	m/z	Res.	S/N	I	I %	FWHM
1	91.0544	11305	103.6	283	51.9	0.0081
2	107.0486	13290	58.4	160	29.2	0.0081
3	115.0544	14284	107.4	294	53.8	0.0081
4	117.0695	14534	199.8	546	100.0	0.0081
5	129.0685	16024	70.8	194	35.4	0.0081
6	145.1004	18014	70.6	193	35.3	0.0081
7	155.0845	19254	72.4	198	36.2	0.0081
8	163.0746	20246	54.2	148	27.1	0.0081
9	173.0948	21490	91.6	251	45.8	0.0081
10	219.1013	27202	58.4	160	29.2	0.0081

Cmpd 1969, AutoMSn(124.0392), 26.02 min

Compound Spectrum List Report

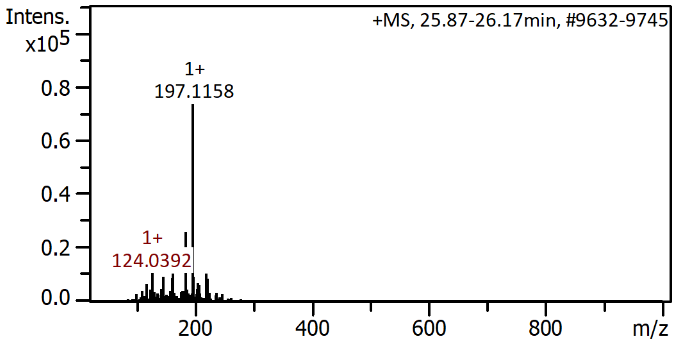

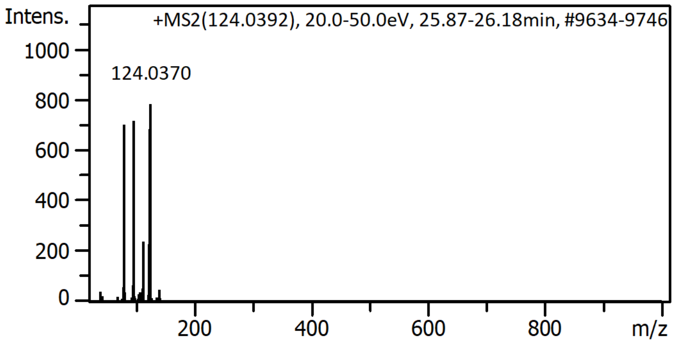

#	m/z	Res.	S/N	I	I %	FWHM
1	127.0375	10229	2742.7	10971	14.9	0.0124
2	145.0478	10589	2298.9	9196	12.5	0.0137
3	159.9678	11254	2165.2	8661	11.8	0.0142
4	163.0595	10432	2574.6	10299	14.0	0.0156
5	185.0408	11193	6520.8	26083	35.5	0.0165
6	197.1158	11577	18361.8	73447	100.0	0.0170
7	198.1190	11473	2293.0	9172	12.5	0.0173
8	205.0020	11473	1680.6	6723	9.2	0.0179
9	219.0975	11498	2584.3	10337	14.1	0.0191
10	222.0169	11472	2132.4	8530	11.6	0.0194

#	m/z	Res.	S/N	I	I %	FWHM
1	78.0352	11254	10.0	57	7.3	0.0069
2	79.0551	12282	16.3	92	11.8	0.0064
3	80.0491	8720	123.8	701	89.9	0.0092
4	95.0506	13196	11.4	65	8.3	0.0072
5	96.0434	9893	126.3	716	91.7	0.0097
6	111.0246	13907	9.2	52	6.7	0.0080
7	112.0383	11400	42.4	240	30.8	0.0098
8	122.0975	10924	40.3	228	29.3	0.0112
9	123.0541	11275	120.4	682	87.4	0.0109
10	124.0370	9562	137.7	780	100.0	0.0130

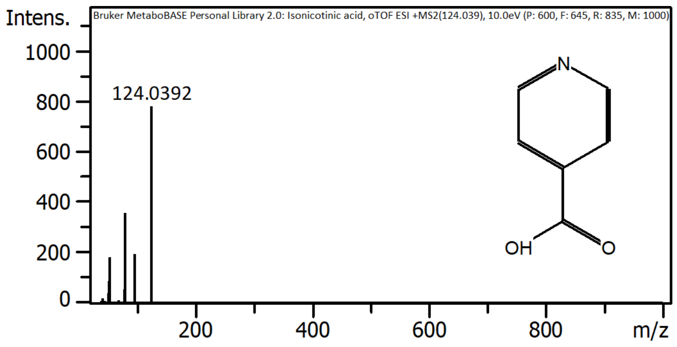

#	m/z	Res.	S/N	I	I %	FWHM
1	41.0392	3435	4.8	19	2.4	0.0119
2	51.0224	4271	10.2	40	5.1	0.0119
3	52.0307	4355	22.4	87	11.2	0.0119
4	53.0390	4439	46.8	183	23.4	0.0119
5	78.0343	6531	13.8	54	6.9	0.0119
6	78.0345	6531	6.8	27	3.4	0.0119
7	79.0421	6616	24.2	94	12.1	0.0119
8	80.0500	6700	92.0	359	46.0	0.0119
9	96.0449	8039	50.6	197	25.3	0.0119
10	124.0392	10382	199.8	779	100.0	0.0119

Cmpd 1989, AutoMSn(124.0374), 26.31 min

Compound Spectrum List Report

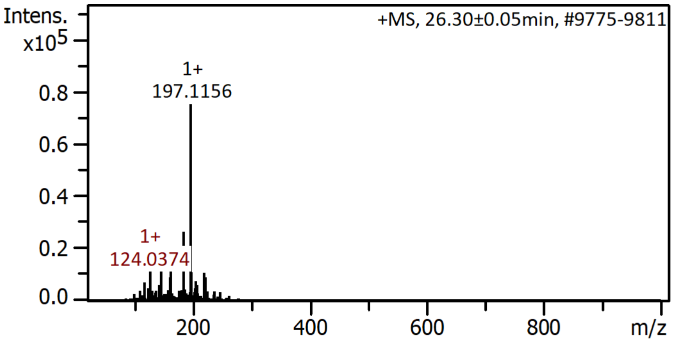

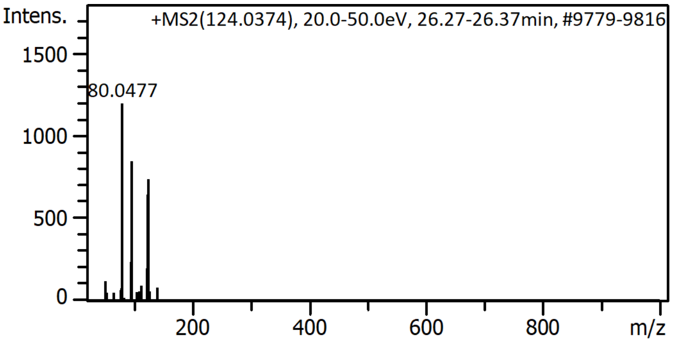

#	m/z	Res.	S/N	I	I %	FWHM
1	127.0381	10272	989.1	11869	15.8	0.0124
2	145.0483	11143	986.8	11842	15.7	0.0130
3	159.9677	10788	735.4	8824	11.7	0.0148
4	163.0585	11276	941.1	11293	15.0	0.0145
5	185.0409	11709	2206.2	26474	35.2	0.0158
6	197.1156	11354	6269.7	75237	100.0	0.0174
7	198.1195	12722	909.4	10912	14.5	0.0156
8	205.0017	11905	612.0	7344	9.8	0.0172
9	219.0968	12660	887.3	10647	14.2	0.0173
10	222.0169	11786	743.0	8916	11.9	0.0188

#	m/z	Res.	S/N	I	I %	FWHM
1	51.0234	9961	7.2	118	9.8	0.0051
2	78.0517	9684	4.6	75	6.3	0.0081
3	80.0477	9434	73.1	1195	100.0	0.0085
4	95.0495	9770	14.6	239	20.0	0.0097
5	96.0445	8718	51.9	847	70.9	0.0110
6	112.0408	14710	5.5	89	7.5	0.0076
7	122.0955	12680	11.9	194	16.2	0.0096
8	123.0536	11754	39.4	644	53.9	0.0105
9	124.0376	9589	45.3	740	61.9	0.0129
10	140.0301	15324	4.9	80	6.7	0.0091

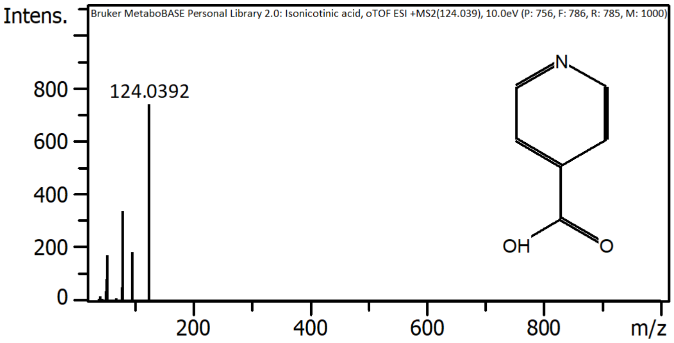

#	m/z	Res.	S/N	I	I %	FWHM
1	41.0392	5407	4.8	18	2.4	0.0076
2	51.0224	6722	10.2	38	5.1	0.0076
3	52.0307	6855	22.4	83	11.2	0.0076
4	53.0390	6988	46.8	173	23.4	0.0076
5	78.0343	10281	13.8	51	6.9	0.0076
6	78.0345	10281	6.8	25	3.4	0.0076
7	79.0421	10414	24.2	89	12.1	0.0076
8	80.0500	10546	92.0	340	46.0	0.0076
9	96.0449	12654	50.6	187	25.3	0.0076
10	124.0392	16342	199.8	739	100.0	0.0076

Cmpd 2008, AutoMSn(127.0389), 26.57 min

Compound Spectrum List Report

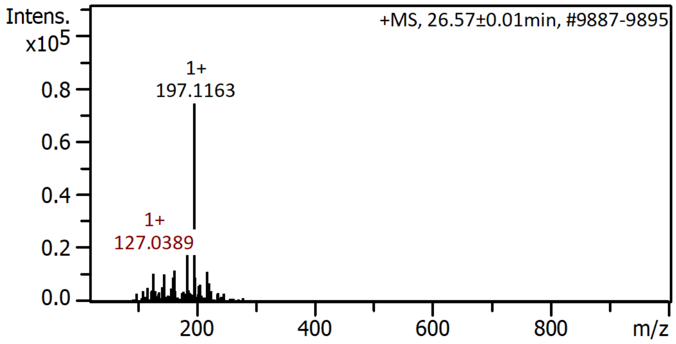

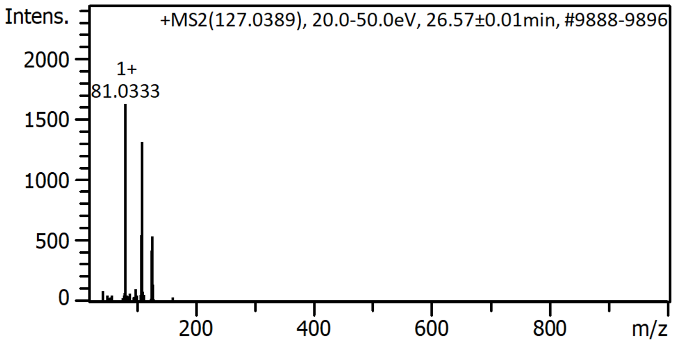

#	m/z	Res.	S/N	I	I %	FWHM
1	127.0389	9435	875.1	10501	14.1	0.0135
2	145.0479	9323	848.7	10184	13.7	0.0156
3	159.9686	10273	755.8	9069	12.2	0.0156
4	163.0588	9988	977.3	11727	15.8	0.0163
5	185.0413	10642	1950.9	23411	31.5	0.0174
6	197.1163	10048	6189.9	74279	100.0	0.0196
7	198.1200	10764	759.9	9118	12.3	0.0184
8	207.1364	10602	526.6	6320	8.5	0.0195
9	219.0990	10247	935.9	11231	15.1	0.0214
10	222.0187	11854	574.9	6899	9.3	0.0187

#	m/z	Res.	S/N	I	I %	FWHM
1	43.0160	8527	5.2	89	5.5	0.0050
2	81.0333	7995	95.4	1622	100.0	0.0101
3	99.0465	13596	6.0	101	6.2	0.0073
4	108.0459	10037	32.3	549	33.9	0.0108
5	109.0264	8248	77.1	1311	80.8	0.0132
6	110.0564	13286	4.6	79	4.9	0.0083
7	126.0527	8247	24.9	424	26.1	0.0153
8	127.0382	8324	31.6	538	33.1	0.0153
9	128.0430	15520	8.3	141	8.7	0.0083
10	128.1075	15237	4.4	75	4.6	0.0084

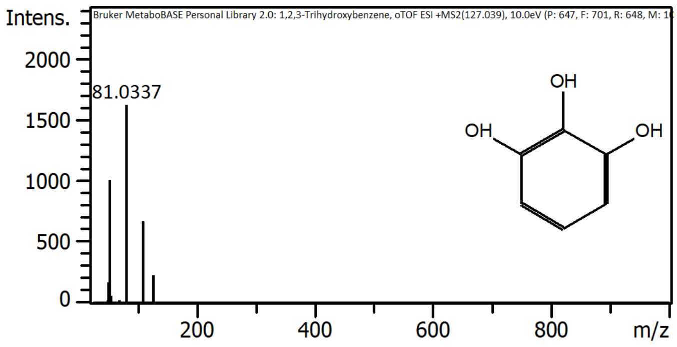

#	m/z	Res.	S/N	I	I %	FWHM
1	50.0138	5665	3.2	26	1.6	0.0088
2	51.0231	5779	21.2	172	10.6	0.0088
3	52.0328	5894	2.2	18	1.1	0.0088
4	53.0388	6008	124.4	1009	62.3	0.0088
5	55.0182	6232	7.6	62	3.8	0.0088
6	68.9975	7815	2.8	23	1.4	0.0088
7	81.0337	9179	199.8	1620	100.0	0.0088
8	81.1130	9188	6.6	54	3.3	0.0088
9	109.0274	12350	82.8	672	41.4	0.0088
10	127.0386	14390	28.4	230	14.2	0.0088

Cmpd 2013, AutoMSn(124.0389), 26.63 min

Compound Spectrum List Report

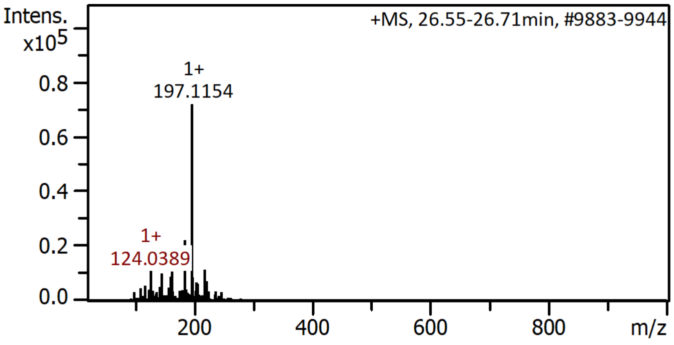

#	m/z	Res.	S/N	I	I %	FWHM
1	127.0382	9215	1218.1	10963	15.2	0.0138
2	145.0475	9714	1097.9	9881	13.7	0.0149
3	159.9674	10402	986.5	8878	12.3	0.0154
4	163.0584	10660	1191.5	10724	14.9	0.0153
5	185.0404	10904	2470.7	22237	30.9	0.0170
6	197.1154	10823	7994.8	71953	100.0	0.0182
7	198.1194	11054	935.6	8421	11.7	0.0179
8	205.0016	10943	748.7	6738	9.4	0.0187
9	219.0971	11293	1269.7	11428	15.9	0.0194
10	222.0169	12090	798.0	7182	10.0	0.0184

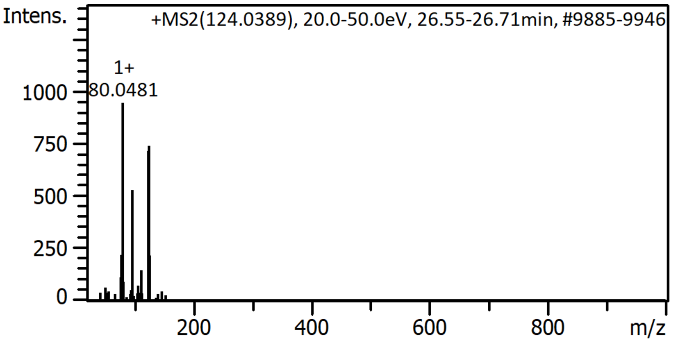

#	m/z	Res.	S/N	I	I %	FWHM
1	77.0339	10536	8.6	110	11.7	0.0073
2	78.0365	7907	17.3	220	23.3	0.0099
3	79.0483	11702	6.2	79	8.3	0.0068
4	80.0481	8699	74.0	944	100.0	0.0092
5	81.0414	34318	7.1	90	9.6	0.0024
6	96.0422	9724	41.5	530	56.1	0.0099
7	112.0352	13701	11.3	145	15.3	0.0082
8	123.0538	11508	56.1	716	75.8	0.0107
9	124.0364	9756	58.1	741	78.5	0.0127
10	126.0539	11608	17.1	218	23.0	0.0109

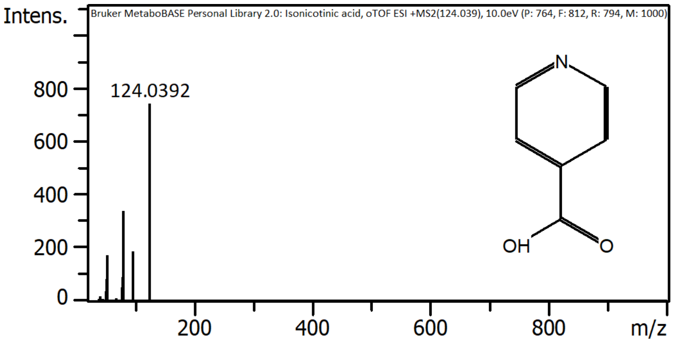

#	m/z	Res.	S/N	I	I %	FWHM
1	41.0392	5660	4.8	18	2.4	0.0073
2	51.0224	7037	10.2	38	5.1	0.0073
3	52.0307	7176	22.4	83	11.2	0.0073
4	53.0390	7316	46.8	173	23.4	0.0073
5	78.0343	10763	13.8	51	6.9	0.0073
6	78.0345	10763	6.8	25	3.4	0.0073
7	79.0421	10902	24.2	90	12.1	0.0073
8	80.0500	11041	92.0	341	46.0	0.0073
9	96.0449	13247	50.6	187	25.3	0.0073
10	124.0392	17108	199.8	740	100.0	0.0073

Cmpd 2035, AutoMSn(124.0388), 26.91 min

Compound Spectrum List Report

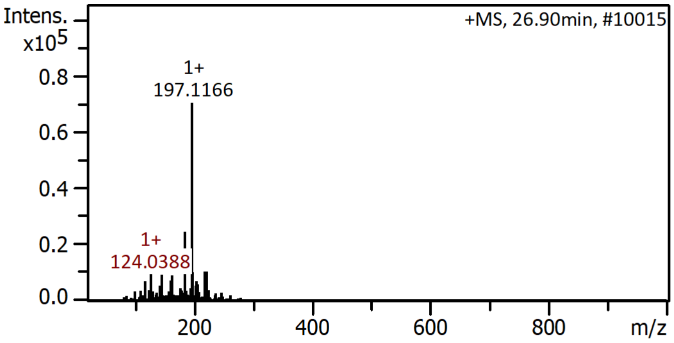

#	m/z	Res.	S/N	I	I %	FWHM
1	117.0535	9796	190.8	6869	9.8	0.0119
2	127.0389	9062	266.8	9605	13.7	0.0140
3	145.0485	11290	252.8	9100	13.0	0.0128
4	159.9681	10538	201.8	7263	10.3	0.0152
5	163.0593	12076	248.9	8962	12.8	0.0135
6	185.0419	10904	682.3	24564	35.0	0.0170
7	197.1166	11634	1951.4	70251	100.0	0.0169
8	198.1179	10471	278.8	10037	14.3	0.0189
9	219.0963	10051	288.8	10395	14.8	0.0218
10	222.0174	12273	288.7	10393	14.8	0.0181

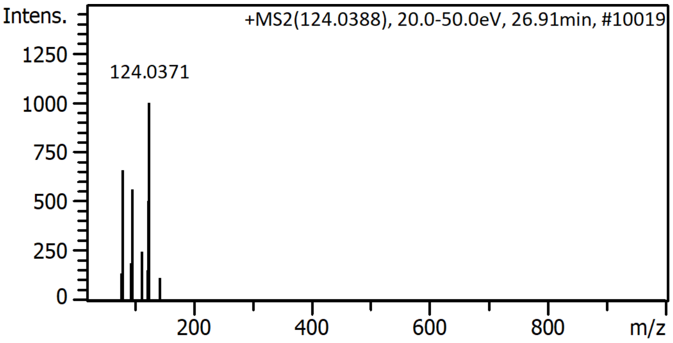

#	m/z	Res.	S/N	I	I %	FWHM
1	78.0340	11908	1.9	136	13.6	0.0066
2	80.0499	9118	9.2	661	66.2	0.0088
3	94.0315	12979	2.6	190	19.0	0.0072
4	94.0624	14187	2.2	160	16.0	0.0066
5	96.0456	12922	7.8	564	56.5	0.0074
6	112.0271	5796	3.5	251	25.2	0.0193
7	122.0550	14540	2.1	153	15.3	0.0084
8	122.0959	14626	2.0	145	14.5	0.0083
9	123.0523	7485	7.0	506	50.7	0.0164
10	124.0371	11705	13.9	998	100.0	0.0106

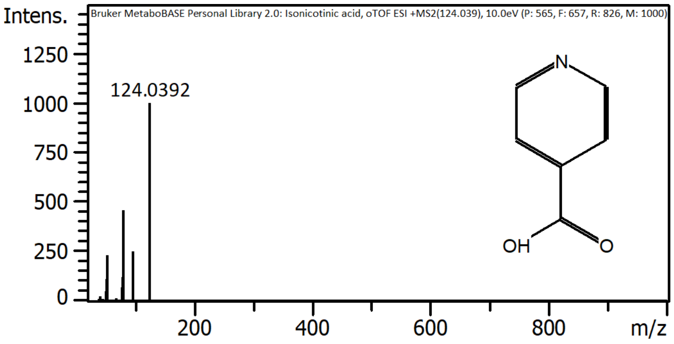

#	m/z	Res.	S/N	I	I %	FWHM
1	41.0392	3680	4.8	24	2.4	0.0112
2	51.0224	4575	10.2	51	5.1	0.0112
3	52.0307	4665	22.4	112	11.2	0.0112
4	53.0390	4756	46.8	234	23.4	0.0112
5	78.0343	6997	13.8	69	6.9	0.0112
6	78.0345	6997	6.8	34	3.4	0.0112
7	79.0421	7087	24.2	121	12.1	0.0112
8	80.0500	7178	92.0	459	46.0	0.0112
9	96.0449	8612	50.6	252	25.3	0.0112
10	124.0392	11122	199.8	997	100.0	0.0112

Cmpd 2076, AutoMSn(124.0391), 27.46 min

Compound Spectrum List Report

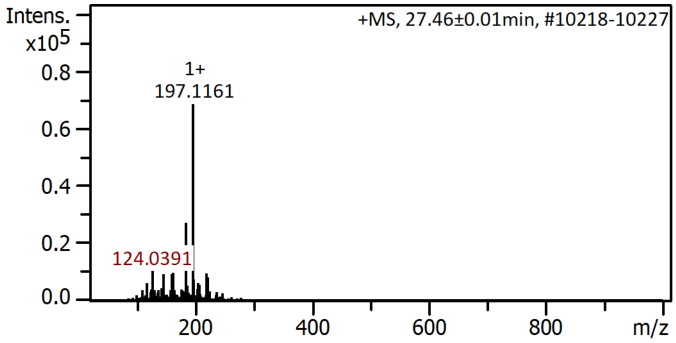

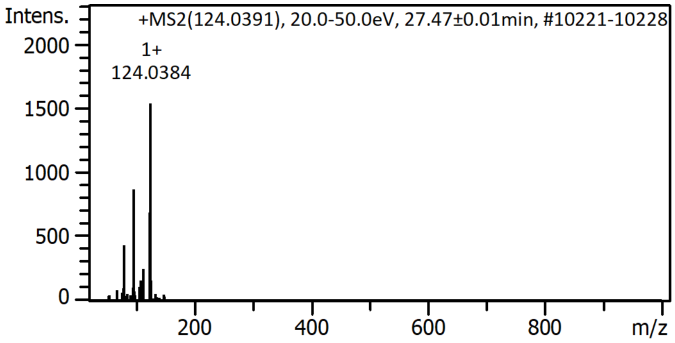

#	m/z	Res.	S/N	I	I %	FWHM
1	117.0535	9522	504.8	6058	8.8	0.0123
2	127.0382	10255	990.9	11890	17.4	0.0124
3	145.0478	10721	772.2	9266	13.5	0.0135
4	159.9677	10979	767.8	9214	13.5	0.0146
5	163.0588	11196	801.9	9623	14.1	0.0146
6	185.0408	11730	2281.6	27379	40.0	0.0158
7	197.1161	11523	5707.4	68488	100.0	0.0171
8	198.1189	11429	624.2	7490	10.9	0.0173
9	219.0986	11888	780.5	9366	13.7	0.0184
10	222.0177	12120	676.8	8121	11.9	0.0183

#	m/z	Res.	S/N	I	I %	FWHM
1	80.0473	9455	25.0	434	28.3	0.0085
2	96.0437	9589	50.0	866	56.4	0.0100
3	96.0825	13132	7.0	122	7.9	0.0073
4	106.0278	13537	6.2	107	7.0	0.0078
5	108.0432	10241	9.1	158	10.3	0.0106
6	112.0392	13237	14.3	247	16.1	0.0085
7	123.0562	10739	39.7	688	44.8	0.0115
8	124.0384	12252	88.6	1535	100.0	0.0101
9	125.0416	13408	7.3	126	8.2	0.0093
10	126.0509	14413	8.9	155	10.1	0.0087

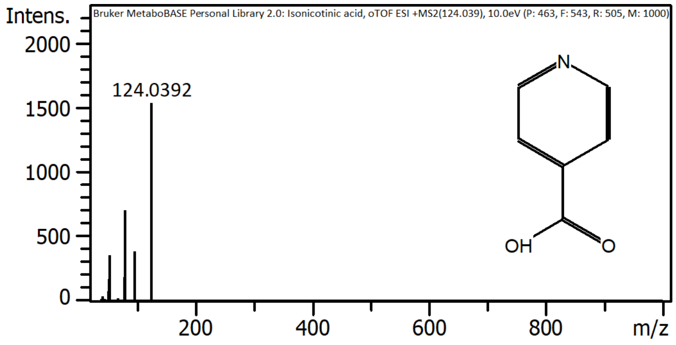

#	m/z	Res.	S/N	I	I %	FWHM
1	41.0392	3841	4.8	37	2.4	0.0107
2	51.0224	4775	10.2	78	5.1	0.0107
3	52.0307	4869	22.4	172	11.2	0.0107
4	53.0390	4964	46.8	359	23.4	0.0107
5	78.0343	7303	13.8	106	6.9	0.0107
6	78.0345	7303	6.8	52	3.4	0.0107
7	79.0421	7397	24.2	186	12.1	0.0107
8	80.0500	7492	92.0	706	46.0	0.0107
9	96.0449	8989	50.6	388	25.3	0.0107
10	124.0392	11609	199.8	1533	100.0	0.0107

Cmpd 2167, AutoMSn(124.0383), 28.71 min

Compound Spectrum List Report

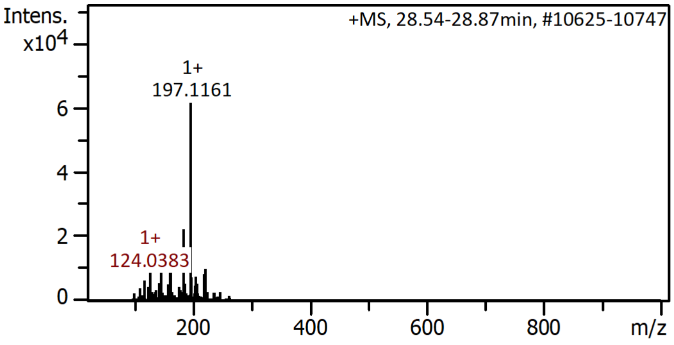

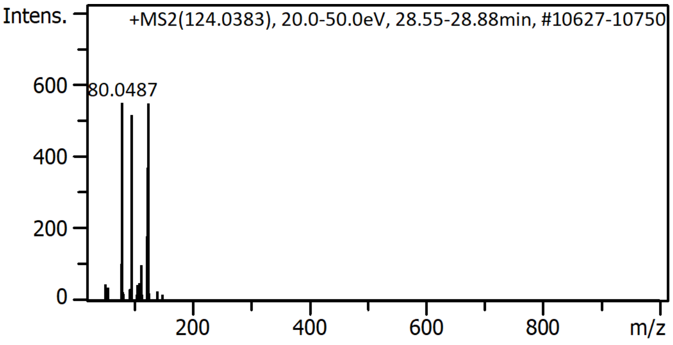

#	m/z	Res.	S/N	I	I %	FWHM
1	127.0378	9977	1982.9	10198	16.6	0.0127
2	145.0483	10694	2038.8	10485	17.0	0.0136
3	159.9681	11426	1974.4	10154	16.5	0.0140
4	163.0590	10484	2054.6	10566	17.2	0.0156
5	185.0407	11514	4355.6	22400	36.4	0.0161
6	197.1161	11207	11969.3	61556	100.0	0.0176
7	198.1190	10699	1407.9	7240	11.8	0.0185
8	205.0022	11585	1440.6	7409	12.0	0.0177
9	219.0976	10708	1596.4	8210	13.3	0.0205
10	222.0175	11840	1923.0	9890	16.1	0.0188

#	m/z	Res.	S/N	I	I %	FWHM
1	51.0236	9437	6.1	45	8.2	0.0054
2	78.0322	11601	13.9	101	18.5	0.0067
3	80.0487	9488	75.2	548	100.0	0.0084
4	96.0437	9013	70.6	514	93.9	0.0107
5	109.0250	14445	6.6	48	8.8	0.0075
6	112.0411	14142	13.6	99	18.0	0.0079
7	122.0988	8305	24.7	180	32.9	0.0147
8	123.0536	11513	50.7	370	67.5	0.0107
9	124.0400	8781	75.0	546	99.7	0.0141
10	124.0542	11681	13.5	98	17.9	0.0106

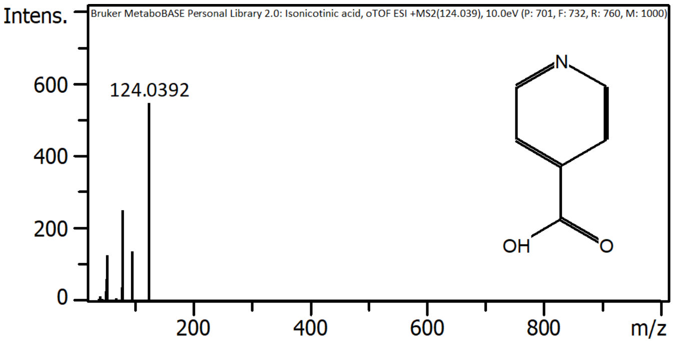

#	m/z	Res.	S/N	I	I %	FWHM
1	41.0392	5330	4.8	13	2.4	0.0077
2	51.0224	6627	10.2	28	5.1	0.0077
3	52.0307	6758	22.4	61	11.2	0.0077
4	53.0390	6889	46.8	128	23.4	0.0077
5	78.0343	10135	13.8	38	6.9	0.0077
6	78.0345	10135	6.8	19	3.4	0.0077
7	79.0421	10266	24.2	66	12.1	0.0077
8	80.0500	10397	92.0	251	46.0	0.0077
9	96.0449	12474	50.6	138	25.3	0.0077
10	124.0392	16110	199.8	546	100.0	0.0077

Cmpd 2175, AutoMSn(127.0374), 28.80 min

Compound Spectrum List Report

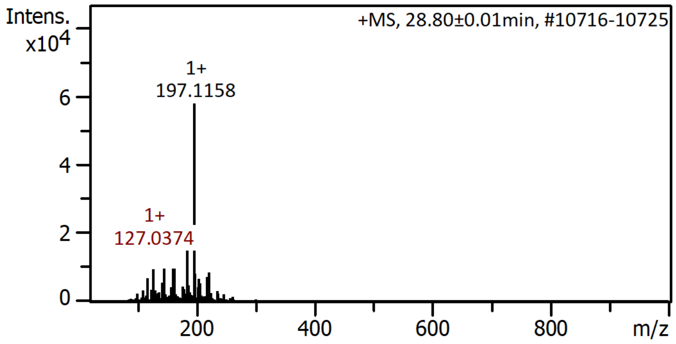

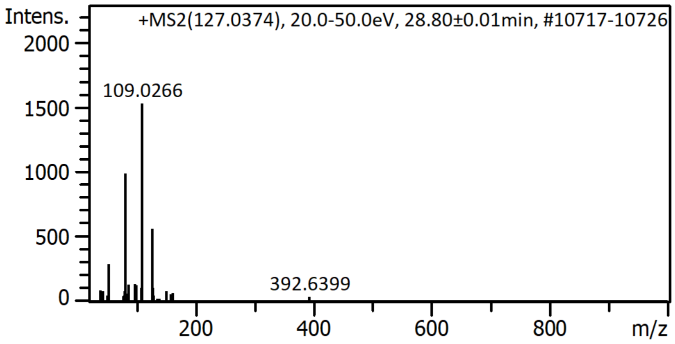

#	m/z	Res.	S/N	I	I %	FWHM
1	117.0536	9806	567.6	6811	11.8	0.0119
2	127.0374	9959	781.8	9381	16.2	0.0128
3	145.0482	10100	808.8	9706	16.8	0.0144
4	159.9677	11525	800.7	9609	16.6	0.0139
5	163.0599	9978	804.1	9650	16.7	0.0163
6	185.0403	10646	1793.2	21518	37.2	0.0174
7	197.1158	11154	4816.6	57800	100.0	0.0177
8	198.1189	10965	682.4	8189	14.2	0.0181
9	219.0973	11088	592.6	7111	12.3	0.0198
10	222.0168	11336	708.2	8498	14.7	0.0196

#	m/z	Res.	S/N	I	I %	FWHM
1	53.0404	5567	17.4	290	19.0	0.0095
2	81.0330	7511	59.2	987	64.7	0.0108
3	86.0589	12315	7.7	129	8.4	0.0070
4	97.0257	12983	8.0	133	8.7	0.0075
5	99.0423	13136	7.7	128	8.4	0.0075
6	108.0483	14108	6.3	106	6.9	0.0077
7	109.0266	10328	91.5	1525	100.0	0.0106
8	126.0519	9842	33.9	565	37.0	0.0128
9	127.0389	10424	31.8	530	34.7	0.0122
10	128.1068	14970	6.3	105	6.9	0.0086

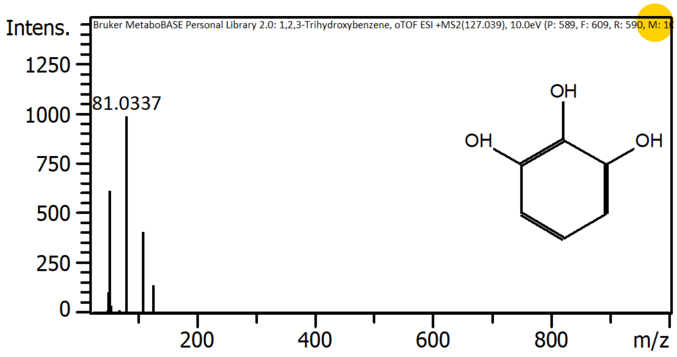

#	m/z	Res.	S/N	I	I %	FWHM
1	50.0138	4677	3.2	16	1.6	0.0107
2	51.0231	4771	21.2	105	10.6	0.0107
3	52.0328	4865	2.2	11	1.1	0.0107
4	53.0388	4959	124.4	614	62.3	0.0107
5	55.0182	5144	7.6	38	3.8	0.0107
6	68.9975	6452	2.8	14	1.4	0.0107
7	81.0337	7577	199.8	986	100.0	0.0107
8	81.1130	7584	6.6	33	3.3	0.0107
9	109.0274	10195	82.8	409	41.4	0.0107
10	127.0386	11879	28.4	140	14.2	0.0107

Cmpd 2193, AutoMSn(124.0389), 29.09 min

Compound Spectrum List Report

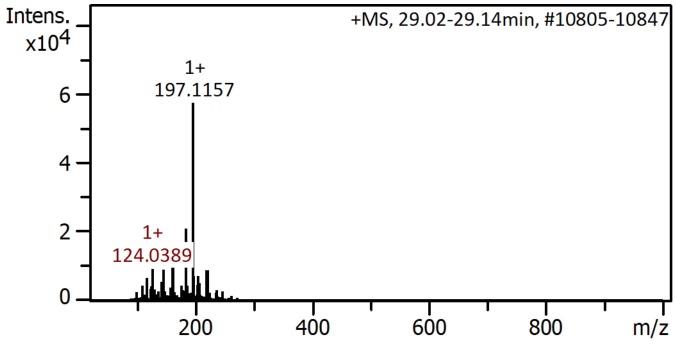

#	m/z	Res.	S/N	I	I %	FWHM
1	127.0379	9768	1266.6	9120	15.9	0.0130
2	145.0480	10873	1241.2	8936	15.6	0.0133
3	159.9678	10267	1353.0	9742	17.0	0.0156
4	163.0584	10975	1387.3	9989	17.4	0.0149
5	185.0404	10728	2935.7	21137	36.8	0.0172
6	197.1157	11079	7970.3	57386	100.0	0.0178
7	198.1181	12614	981.4	7066	12.3	0.0157
8	205.0016	11009	980.9	7063	12.3	0.0186
9	219.0967	10878	1227.6	8839	15.4	0.0201
10	222.0168	12129	1219.5	8781	15.3	0.0183

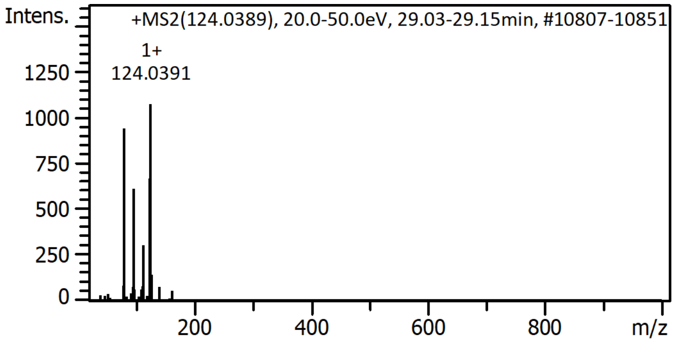

#	m/z	Res.	S/N	I	I %	FWHM
1	78.0311	11936	7.7	83	7.7	0.0065
2	80.0493	8363	87.0	939	87.5	0.0096
3	95.0504	13233	7.0	75	7.0	0.0072
4	96.0438	10117	56.8	613	57.1	0.0095
5	111.0315	14438	7.2	78	7.2	0.0077
6	112.0415	12889	28.2	305	28.4	0.0087
7	123.0532	11242	61.8	668	62.2	0.0109
8	124.0391	9309	99.4	1073	100.0	0.0133
9	126.0536	10849	13.0	140	13.1	0.0116
10	140.0378	9055	7.1	76	7.1	0.0155

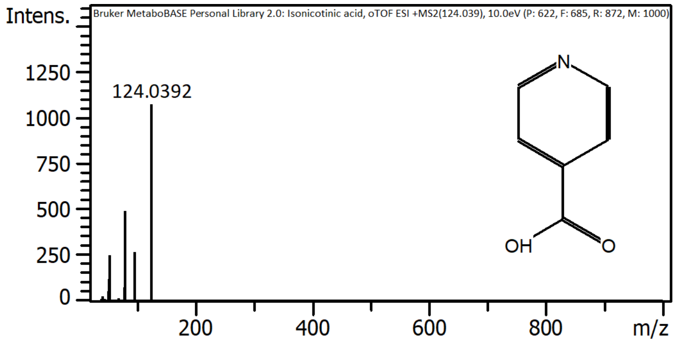

#	m/z	Res.	S/N	I	I %	FWHM
1	41.0392	3376	4.8	26	2.4	0.0122
2	51.0224	4198	10.2	55	5.1	0.0122
3	52.0307	4281	22.4	120	11.2	0.0122
4	53.0390	4364	46.8	251	23.4	0.0122
5	78.0343	6420	13.8	74	6.9	0.0122
6	78.0345	6420	6.8	36	3.4	0.0122
7	79.0421	6503	24.2	130	12.1	0.0122
8	80.0500	6586	92.0	494	46.0	0.0122
9	96.0449	7902	50.6	272	25.3	0.0122
10	124.0392	10205	199.8	1072	100.0	0.0122

Cmpd 2216, AutoMSn(127.0379), 29.43 min

Compound Spectrum List Report

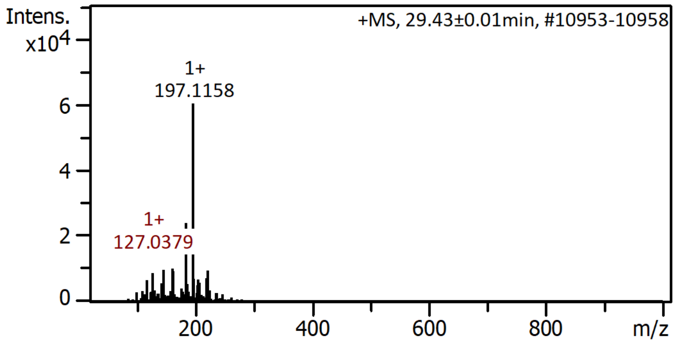

#	m/z	Res.	S/N	I	I %	FWHM
1	127.0379	10137	723.8	8686	14.4	0.0125
2	145.0482	10423	809.8	9717	16.1	0.0139
3	159.9678	10842	840.3	10084	16.7	0.0148
4	163.0590	10039	776.6	9319	15.5	0.0162
5	185.0410	11624	2009.1	24109	40.0	0.0159
6	197.1158	11472	5025.2	60302	100.0	0.0172
7	198.1197	10865	582.0	6984	11.6	0.0182
8	205.0019	10519	561.1	6733	11.2	0.0195
9	219.0978	10404	597.0	7164	11.9	0.0211
10	222.0174	12495	788.6	9463	15.7	0.0178

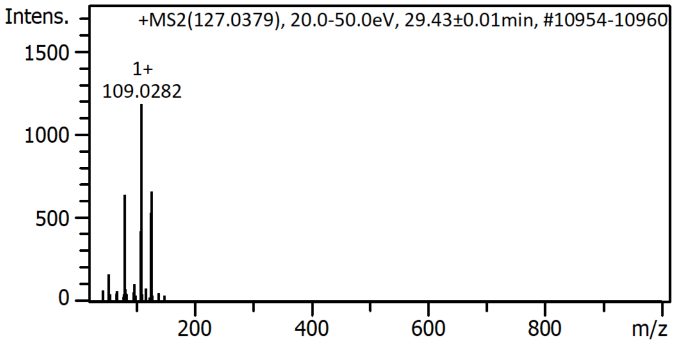

#	m/z	Res.	S/N	I	I %	FWHM
1	53.0381	9735	9.1	163	13.8	0.0054
2	81.0348	7335	35.6	642	54.3	0.0110
3	82.0388	7464	4.1	74	6.2	0.0110
4	97.0218	13157	5.9	107	9.0	0.0074
5	108.0443	10117	23.6	424	35.9	0.0107
6	109.0282	7888	65.6	1181	100.0	0.0138
7	117.0091	13777	4.3	77	6.5	0.0085
8	126.0531	9663	29.6	533	45.2	0.0130
9	126.0866	15706	5.4	96	8.2	0.0080
10	127.0373	9868	36.7	661	56.0	0.0129

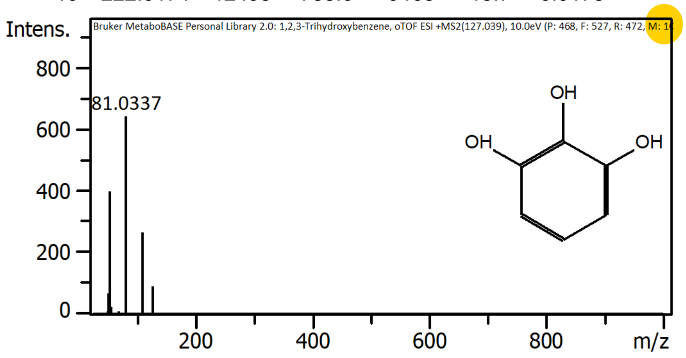

#	m/z	Res.	S/N	I	I %	FWHM
1	50.0138	4454	3.2	10	1.6	0.0112
2	51.0231	4544	21.2	68	10.6	0.0112
3	52.0328	4634	2.2	7	1.1	0.0112
4	53.0388	4723	124.4	399	62.3	0.0112
5	55.0182	4900	7.6	24	3.8	0.0112
6	68.9975	6145	2.8	9	1.4	0.0112
7	81.0337	7217	199.8	641	100.0	0.0112
8	81.1130	7224	6.6	21	3.3	0.0112
9	109.0274	9710	82.8	266	41.4	0.0112
10	127.0386	11314	28.4	91	14.2	0.0112

Cmpd 2222, AutoMSn(124.0379), 29.51 min

Compound Spectrum List Report

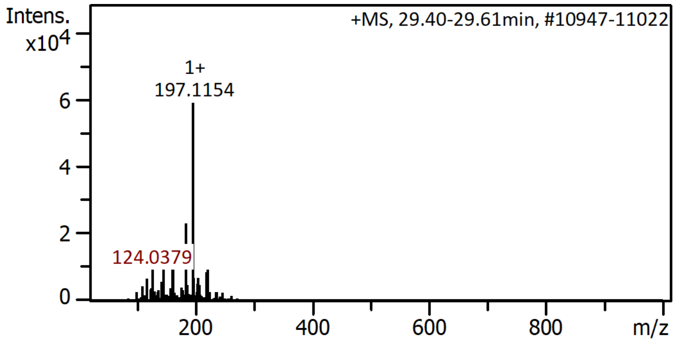

#	m/z	Res.	S/N	I	I %	FWHM
1	127.0378	9874	1836.7	9446	16.0	0.0129
2	145.0474	10685	1968.4	10123	17.2	0.0136
3	159.9680	10181	2002.1	10296	17.5	0.0157
4	163.0583	11055	1953.3	10045	17.1	0.0147
5	185.0401	11240	4517.2	23231	39.4	0.0165
6	197.1154	11406	11453.1	58901	100.0	0.0173
7	198.1183	10806	1332.6	6853	11.6	0.0183
8	205.0024	11218	1308.4	6729	11.4	0.0183
9	219.0972	12270	1660.3	8539	14.5	0.0179
10	222.0164	11665	1789.9	9205	15.6	0.0190

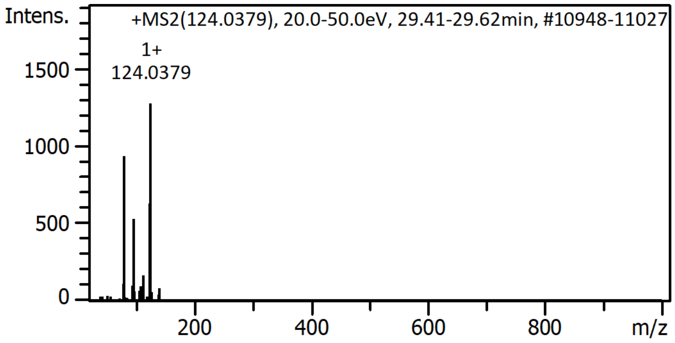

#	m/z	Res.	S/N	I	I %	FWHM
1	78.0332	6910	15.0	109	8.6	0.0113
2	80.0489	8796	128.7	937	73.5	0.0091
3	94.0279	12548	13.3	97	7.6	0.0075
4	96.0440	7698	72.8	531	41.6	0.0125
5	108.0435	12693	13.0	95	7.4	0.0085
6	111.0278	13656	9.3	68	5.3	0.0081
7	112.0380	12373	22.7	165	13.0	0.0091
8	123.0535	11343	86.5	630	49.4	0.0108
9	124.0379	10707	175.0	1275	100.0	0.0116
10	140.0344	12183	11.2	82	6.4	0.0115

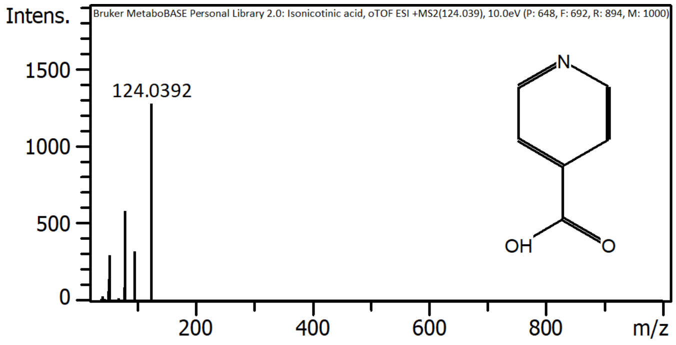

#	m/z	Res.	S/N	I	I %	FWHM
1	41.0392	3437	4.8	31	2.4	0.0119
2	51.0224	4273	10.2	65	5.1	0.0119
3	52.0307	4358	22.4	143	11.2	0.0119
4	53.0390	4442	46.8	298	23.4	0.0119
5	78.0343	6536	13.8	88	6.9	0.0119
6	78.0345	6536	6.8	43	3.4	0.0119
7	79.0421	6620	24.2	154	12.1	0.0119
8	80.0500	6705	92.0	586	46.0	0.0119
9	96.0449	8044	50.6	323	25.3	0.0119
10	124.0392	10389	199.8	1273	100.0	0.0119

Cmpd 2249, AutoMSn(124.0381), 29.82 min

Compound Spectrum List Report

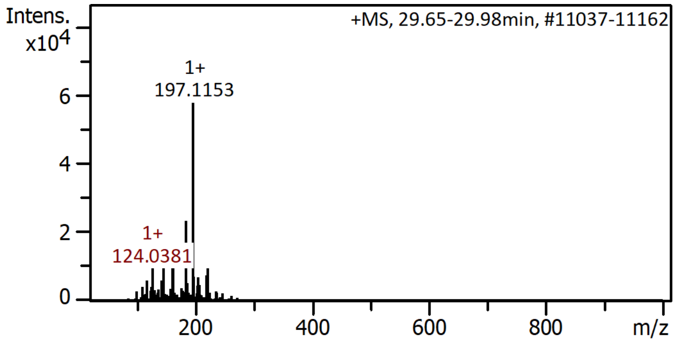

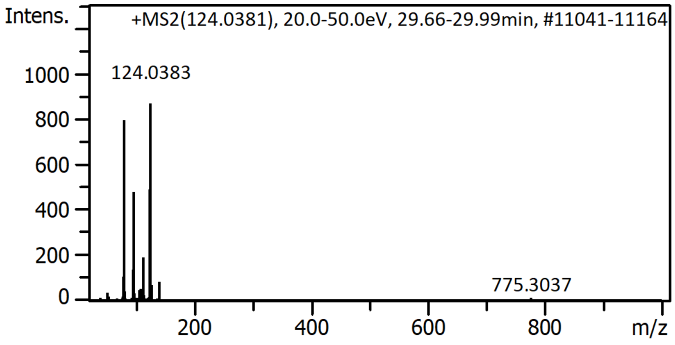

#	m/z	Res.	S/N	I	I %	FWHM
1	127.0376	10241	3564.6	9871	17.1	0.0124
2	145.0473	10343	3463.6	9592	16.6	0.0140
3	159.9678	10965	3498.3	9688	16.8	0.0146
4	163.0576	10507	3544.0	9814	17.0	0.0155
5	185.0403	11206	8448.9	23397	40.5	0.0165
6	197.1153	11589	20838.4	57706	100.0	0.0170
7	198.1181	10852	2558.9	7086	12.3	0.0183
8	205.0014	11211	2476.3	6857	11.9	0.0183
9	219.0969	12030	2668.0	7388	12.8	0.0182
10	222.0167	12116	3382.6	9367	16.2	0.0183

#	m/z	Res.	S/N	I	I %	FWHM
1	78.0322	9606	27.1	106	12.2	0.0081
2	80.0489	7677	202.8	796	91.6	0.0104
3	95.0474	9871	34.4	135	15.6	0.0096
4	96.0450	8883	122.6	481	55.4	0.0108
5	112.0368	10144	48.9	192	22.1	0.0110
6	123.0532	10889	125.5	492	56.7	0.0113
7	124.0383	10439	221.4	868	100.0	0.0119
8	124.0758	15049	20.7	81	9.4	0.0082
9	126.0531	13648	17.5	69	7.9	0.0092
10	140.0318	14978	21.5	84	9.7	0.0093

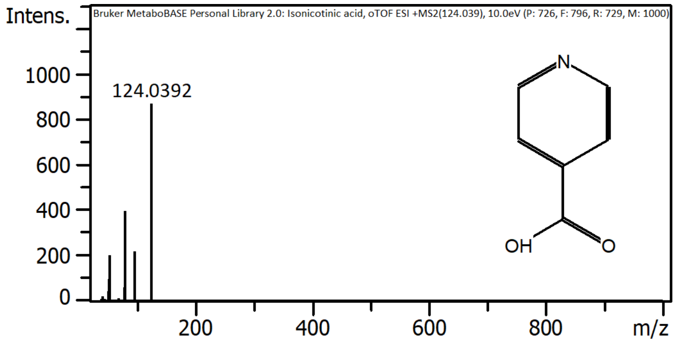

#	m/z	Res.	S/N	I	I %	FWHM
1	41.0392	3307	4.8	21	2.4	0.0124
2	51.0224	4112	10.2	44	5.1	0.0124
3	52.0307	4193	22.4	97	11.2	0.0124
4	53.0390	4274	46.8	203	23.4	0.0124
5	78.0343	6289	13.8	60	6.9	0.0124
6	78.0345	6289	6.8	30	3.4	0.0124
7	79.0421	6370	24.2	105	12.1	0.0124
8	80.0500	6451	92.0	399	46.0	0.0124
9	96.0449	7740	50.6	220	25.3	0.0124
10	124.0392	9996	199.8	868	100.0	0.0124
